# Supplementary material for: Multiomics analysis of serial PARP inhibitor treated metastatic TNBC inform on rational combination therapies
Source: NPJ Precis Oncol. 2021 Oct 19;5:92. doi: 10.1038/s41698-021-00232-w (PMC8526613; doi:10.1038/s41698-021-00232-w)
Supplement: Supplementary file 1 — Supplementary Information [file 41698_2021_232_MOESM1_ESM.pdf]

**Supplementary method:** GeneTrails© Comprehensive Solid Tumor Panel, Multiplex Immunohistochemistry, Image Acquisition and Processing, Cyclic-Immunofluorescence, Image processing and analysis for Cyclic-Immunofluorescence.

*Supplementary Method: GeneTrails© Comprehensive Solid Tumor Panel.*

The test is performed on DNA and RNA extracted from macro-dissected, tumor-rich regions of formalin-fixed/paraffin-embedded tissue. The DNA and RNA (cDNA) NGS library preparations are both based on custom QiaSeq chemistry (Qiagen) that includes unique molecular indices for the assessment of library complexity. Sequencing is performed on Illumina NextSeq500/550 instruments and the data are analyzed through custom bioinformatics pipelines. The DNA library is generated by 9,229 custom-designed primer extension assays covering 613,343 base pairs across 124 cancer-related genes. This panel is routinely sequenced to an average read depth of >2,000, providing high sensitivity for SNVs, in/dels and copy number alterations. Variants are identified using both FreeBayes and MuTect2 algorithms in a custom sequencing analysis pipeline. Included in the panel are 227 short repeats that are used to assess microsatellite instability. TMB is estimated by determining the number of likely somatic mutations and normalizing this to mutations per megabase of sequence.

DNA Sequencing Panel: AKT1, AKT2, AKT3, ALK, APC, AR, ARAF, ARID1A, ATM, ATR, BAP1, BARD1, BRAF, BRCA1, BRCA2, BRIP1, CASP8, CCND1, CCNE1, CHEK1, CD274, CDK12, CDKN1B, CDKN2A, CHEK2, CTNNB1, DDR2, DDX11, EGFR, ERBB2, ERBB3, ERBB4, ERCC2, FANCM, ERCC5, ESR1, FAM175A, FANCA, FANCC, FANCD2, FANCE, FANCF, FANCG, FGF3, FGF4, FGF18, FGF19, FGFR1, FGFR2, FGFR3, FGFR4, GNA11, GNAQ , GNAS, HIST1H3B, HRAS, IDH1, IDH2, IDO1, IDO2, INPP4B, NBN, JAK2, KDR, KIT, KRAS, MAP2K1, MAP2K2, MAP2K4, MAPK1, MDC1, MDM2, MDM4, MET, MLH1, MLH3, MRE11, MSH2, MSH6, MTOR, MUTYH, MYC, NF1, NRAS, NTRK1, NTRK2, NTRK3, PALB2, PDCD1LG2, PDGFRA, PIK3CA, PI3CB, PIK3R1, PMS1, PMS2, PLE, PPP2R1A, PPP6C, PTCH1, PTEN, RAC1, RAD50, RAD51, RAD51B, RAD51C, RAD51D, RAD52, RAD54L, RAF1, RASA1, RB1, RET, PICTOR, RIT1, ROS1, RPTOR, STAG2, STAT3, STK11, TOP1, TP53, TSC1, TSC2, XRCC1

*Supplementary Method: Multiplex Immunohistochemistry, Image Acquisition and Processing*

Multiplex IHC was performed on 5 μm FFPE sections using an adapted protocol based on methodology described previously [25, 26]. Briefly, slides were deparaffinized and stained with hematoxylin (S3301, Dako), followed by whole-slide scanning at 20X magnification on an Aperio AT2 (Leica Biosystems). Tissues then underwent 15 minutes of heat-mediated antigen retrieval in pH 6.0 Citra solution (BioGenex), 10 minutes of blocking in Dako Dual Endogenous Enzyme Block (S2003, Dako), then 10 minutes of protein blocking with 5% normal goat serum and 2.5% BSA in TBST. Primary antibody details, dilution, and incubation times are listed in **Suppl. Table 1.** After washing off unbound primary antibody in TBST, either anti-rat, anti-mouse, or anti-rabbit Histofine Simple Stain MAX PO horseradish peroxidase (HRP)-conjugated polymer (Nichirei Biosciences) was applied for 30 minutes at room temperature, followed by AEC chromogen (Vector Laboratories). Slides were digitally scanned following each chromogen development.

Scanned images were registered in MATLAB version R2018b using the SURF algorithm in the Computer Vision Toolbox (The MathWorks, Inc). Image processing and cell quantification were performed using FIJI (FIJI Is Just ImageJ) [27], CellProfiler Version 3.5.1 [28], and FCS Express 6 Image Cytometry RUO (De Novo Software). AEC signal was extracted for quantification and visualization in FIJI using a custom macro for color deconvolution. Briefly, the FIJI plugin Color_Deconvolution [H AEC] was used to separate hematoxylin, followed by postprocessing steps for signal cleaning and background elimination. AEC signal was extracted in FIJI using the NIH plugin RGB_to_CMYK. Color de-convoluted images were processed in CellProfiler to quantify single cell mean intensity signal measurements for each stained marker. Cells were classified based on hierarchical gating (image cytometry) and defined as described in **Fig. 2**. For visualization, signal-extracted images were overlaid in pseudo-color in FIJI.

*Supplementary Method: Cyclic-Immunofluorescence*

Cyclic-Immunofluorescence (Cyc-IF) allows the detection of more than 40 proteins on a single FFPE slide. Multiple sequential rounds of immunofluorescence staining and quenching were performed on each patient sample as previously described [30-32]. A control tissue microarray (TMA) composed of normal and cancer tissue samples as well as endometrium, spleen, breast, colorectal, ovary, and fallopian tube cancer cell lines was incorporated into each step of the experiment for quality control and data scaling. This TMA includes a positive and negative control for each antibody incorporated in the assay. The samples were processed as follow: FFPE slides were baked overnight at 55°C followed by 30 minutes at 65°C, before deparaffination. Antigen retrieval was performed using a Cuisinart pressure cooker (model CPC-600). Briefly, slides were emerged in a coplin jar filled with pH6 Citrate buffer. The pressure cooker was set on high for 4 minutes, and slides were incubated in the pressure cooker for 20 minutes before releasing the pressure. Slides were then rinsed once into warm deionized water and incubated for 15 minutes in warm pH9 Tris/EDTA antigen retrieval buffer. After blocking the slides for 30 minutes at room temperature in a solution of PBS containing 10% NGS and 1% BSA, coverslips were mounted using slowfade Gold DAPI mounting media and slides were scanned at 10X magnification using the Axioscan fluorescence slide scanner (Zeiss). Autofluorescence of each tissue was acquired. After imaging, the coverslips were removed by soaking the slides in a vertical position within a staining dish filled with PBS. Slides were then incubated with the first round of four antibodies (**Suppl. Table 3**), directly labeled with different Alexa-Fluor (AF) molecules (AF488, AF555, AF647 and AF750) for 2 hours at room temperature. After acquiring an image of the staining, the fluorescence molecules were quenched using a solution of 3% peroxide and 20 mM NaOH in PBS. Subsequent rounds of staining and quenching were then performed on each slide until all antibodies were probed.

*Supplementary Method: Image processing and analysis for Cyclic-Immunofluorescence*

Each image acquired during the Cyc-IF assay was registered based on DAPI features acquired from each round of staining, using custom Matlab scripts (version 2018b) [33]. Segmentation of cells and single-cell feature extraction was then performed using QI Software (Quantitative Imaging Systems). Extracted features included nucleus size and mean intensity for each marker and autofluorescence in each wavelength used for the staining process. Nucleus size and autofluorescence in the AF555 channel were used to filter out cells that appeared to be outliers, and only cells expressing epithelial markers (cancer cells) were selected for downstream analysis.

To subtract autofluorescence background, all autofluorescence and protein mean intensities were divided by their respective exposure time. Autofluorescence values from each cell were then subtracted from each protein value, based on their respective wavelength. To normalize and scale the data, all background subtracted data from the samples and the control TMA were incorporated into a single table, and values were z-scored using median and standard deviation across all markers for each individual cell and across cells for each marker. Heat maps were then constructed using unsupervised hierarchical clustering and K-means clustering of the protein and cells from each sample using custom Python scripts.

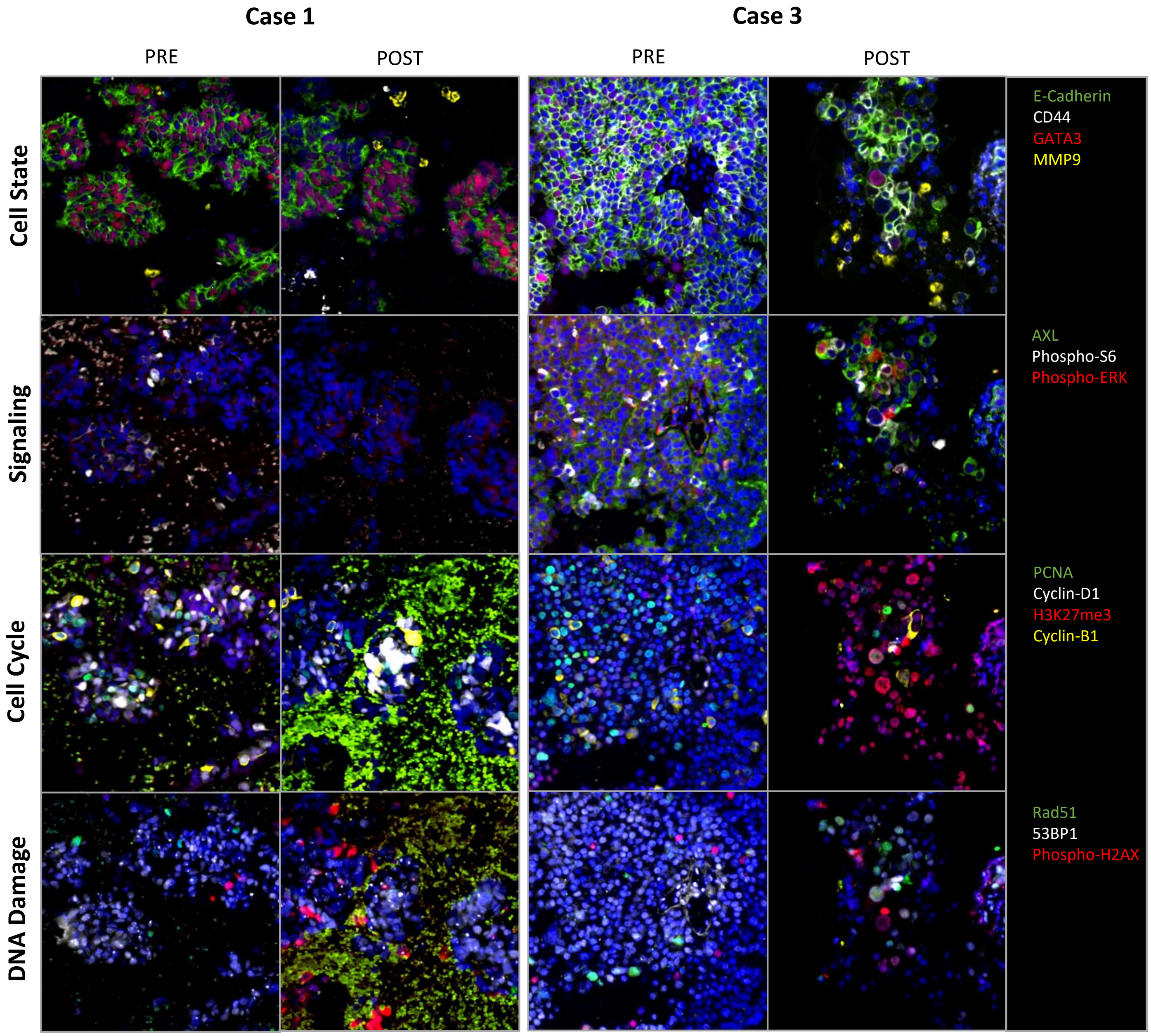


**Supplementary Figure 1: Cyc-IF staining example.**

**Supplementary Table 1: mIHC antibody panel.**

| Cycle | Cycle 0 | Cycle 1 | Cycle 2 | Cycle 3 | Cycle 4 | Cycle 5 | Cycle 6 | Cycle 7 |
|---|---|---|---|---|---|---|---|---|
| **Round** | **Round 1** | **Round 1** | **Round 1** | **Round 1** | **Round 1** | **Round 1** | **Round 1** | **Round 1** |
| **Primary** | Hematoxylin | M aPD1 | M aHLA-DR/DP/DQ | R aCD3 | M aCD8 | R aGrzB Ab-1 | M aCD163 | R aPanCK |
| **Vendor** | Agilent tech | Abcam | LS bio | Thermo Sci | Invitrogen/Thermo | Abcam | Thermo Sci | Cell Signaling |
| **Clone** | | NAT105 | WR18 | SP7 | SP16 | polyclonal | 10D6 | AE1/AE3 |
| **Catalog Number** | S330130-2 | ab52587 | LS-B10162-100 | RM-9107-S | MA5-14548 | ab4059 | MS-1103-S1 | 5153 |
| **Dilution** | | 1/50 | 1/500 | 1:150 | 1:100 | 1:100 | 1:100 | 1:2000 |
| **Reaction** | 2min @RT | 30min @ RT | 30min @ RT | 30min @ RT | 30min @ RT | 30min @ RT | 30min @ RT | 30min @ RT |
| **AEC** | NA | **30 min** | **10 min** | **25 min** | **20 min** | **20 min** | **20 min** | **10 min** |
| **Round** | | **Round 2** | | **Round 2** | **Round 2** | **Round 2** | **Round 2** | **Round 2** |
| **Primary** | | R aPDL1 | | M aCD45 | M aCD20 | Ms aCD68 | EOMES(Tbr2) | R aKi67 |
| **Vendor** | | Cell Signaling | | EBio | Abcam | Abcam | EMD Millipore | Cell Marque |
| **Clone** | | E1L3N | | H130 | L26 | PG-M1 | AB2283 | SP6 |
| **Catalog Number** | | 1684S | | 14-0459-82 | ab9645 | AB783 | AB2283 | 275R-14 |
| **Conc/Ratio 1µL per** | | 1:100 | | 1:100 | 1:100 | 1:50 | 1:1000 | 1:500 |
| **Reaction** | | 4C ON | | 30min @ RT | 30min @ RT | 30min @ RT | 30min @ RT | 30min @ RT |
| **AEC** | | **50 min** | | **30 min** | **30 min** | **20 min** | **10 min** | **10 min** |
| **Round** | | **Round 3** | | | | | | **Round 3** |
| **Primary** | | Hematoxylin | | | | | | Hematoxylin |
| **Vendor** | | Agilent tech | | | | | | Agilent tech |
| **Clone** | | | | | | | | |
| **Catalog Number** | | S330130-2 | | | | | | S330130-2 |
| **Conc/Ratio 1µL per** | | | | | | | | |
| **Reaction** | | 2min @RT | | | | | | 10min @RT |
| **AEC** | | NA | | | | | | NA |

**Supplementary Table 2: RPPA pathway score predictors.**

| Pathway | Predictor | Weight |
|---|---|---|
| Apoptosis | Caspase-7 -cleaved | 1 |
| | Caspase-8 -cleaved | 1 |
| AR | AR | 1 |
| BH3 Balance | BAK | 1 |
| | BAX | 1 |
| | BID | 1 |
| | BIM | 1 |
| | MCL-1 | -1 |
| | BADPS112 | -1 |
| | BCL2 | -1 |
| | BCLXL | -1 |
| | CIAP2 | -1 |
| Cell cycle progression | Cyclin-B1 | 1 |
| | PLK1 | 1 |
| | CDK1_pT14 | 1 |
| | Chk1 | 1 |
| | cdc25C | 1 |
| | Rb_pS807_S811 | 1 |
| | p21 | -1 |
| | p27_pT198 | -1 |
| | CyclinD1 | -1 |
| | 14-3-3-beta | -1 |
| DNA Damage Checkpoint | ATR_pS428 | 1 |
| | cdc2_pY15 | 1 |
| | Chk1_pS296 | 1 |
| | Chk2_pT68 | 1 |
| | Wee1_pS642 | 1 |
| G0-G1 | 14-3-3-beta | 1 |
| | 53BP1 | 1 |
| | BRD4 | -1 |
| | Cyclin-D1 | 1 |
| | Cyclin-B1 | -1 |
| | p27_pT198 | 1 |
| | p21 | 1 |
| G1-S | 53BP1 | 1 |
| | BRD4 | 1 |
| | Cyclin-E1 | 1 |
| G2-M | Cyclin-B1 | 1 |
| | PLK1 | 1 |
| | CDK1_pT14 | 1 |
| | cdc25C | 1 |
| | Rb_pS807_S811 | 1 |
| Immune | Lck | 1 |
| | ZAP-70 | 1 |
| | CD4 | 1 |

| | | |
|---|---|---|
| | CD45 | 1 |
| Immune checkpoint | B7-H4 | 1 |
| | PD-L1 | 1 |
| Notch | Jagged1 | 1 |
| | Notch1 | 1 |
| | Notch3 | 1 |
| | TAZ | 1 |
| | YAP | 1 |
| | YAP_pS127 | -1 |
| PARylation | PAR | 1 |
| PI3K-Akt | Akt_pS473 | 0.5 |
| | Akt_pT308 | 0.5 |
| | GSK-3a-b_pS21_S9 | 1 |
| | INPP4b | -1 |
| | p27_pT198 | 1 |
| | PRAS40_pT246 | 1 |
| | PTEN | -1 |
| | Tuberin_pT1462 | 1 |
| RAS-MAPK | B-Raf_pS445 | 1 |
| | c-Jun_pS73 | 1 |
| | C-Raf_pS338 | 1 |
| | JNK_pT183_Y185 | 1 |
| | MAPK_pT202-Y204 | 1 |
| | MEK1_p_S217-S221 | 1 |
| | p38_pT180_Y182 | 1 |
| | p38-MAPK | 1 |
| | p90RSK_pT573 | 1 |
| | YB1_pS102 | 1 |
| RTK | CMET_pY1235 | 1 |
| | EGFR_pY1173 | 1 |
| | HER2_pY1248 | 1 |
| | HER3_pY1289 | 1 |
| | IGF1R_pY1135_Y1136 | 1 |
| | IRS1 | 1 |
| | Shc_pY317 | 1 |
| | Src_pY416 | 0.5 |
| | Src_pY527 | 0.5 |
| | SHP-2_pY542 | 1 |
| TSC-mTOR | 4E-BP1_pS65 | 1 |
| | mTOR_pS2448 | 1 |
| | p70-S6K_pT389 | 1 |
| | Rb_pS807_S811 | 1 |
| | Rictor_pT1135 | 1 |
| | S6_pS235_S236 | 0.5 |
| | S6_pS240_S244 | 0.5 |
| Tumor Content | betacatenin | 1 |

| | |
|---|---|
| Claudin-7 | 1 |
| E-Cadherin | 1 |
| RBM15 | 1 |
| EPPK1 | 1 |
| Caveolin-1 | -1 |
| Collagen-VI | -1 |
| Lck | -1 |
| MMP2 | -1 |
| PAI-1 | -1 |

**Supplementary Table 3: Cyc-IF antibody panel.**

| Protein | Fluorescence | Company | Cat.No. | Name |
|---|---|---|---|---|
| 53BP1 | AF750 | Abcam | ab222232 | Anti-53BP1 antibody [EPR2172(2)] (ab175933) |
| AXL | AF555 | R&D | AF154 | Human Axl Antibody |
| BRCA1 | AF555 | Abcam | ab215988 | Anti-BRCA1 antibody [EPR19433] - BSA and Azide free |
| CA9 | AF555 | Abcam | ab15086 | Anti-Carbonic Anhydrase IX antibody (ab15086) |
| CCNA2 | AF555 | Abcam | ab217731 | Anti-Cyclin A2 antibody [EPR17351] (Alexa Fluor® 555) (ab217731) |
| CCNB1 | AF555 | Abcam | ab214381 | Anti-Cyclin B1 antibody [Y106] (Alexa Fluor® 555) |
| CCND1 | AF647 | Abcam | ab190563 | Anti-Cyclin D1 antibody [EPR2241] (Alexa Fluor® 647) |
| CCNE | AF647 | lifetech | 50-9714-80 | Cyclin E Antibody, eFluor® 660 |
| CD44 | AF647 | abcam | ab194988 | Anti-CD44 antibody [EPR1013Y] (Alexa Fluor® 647) |
| CDK1 | AF488 | Abcam | ab203852 | Anti-CDK1 antibody [A17] (Alexa Fluor® 488) |
| CK19 (CKs) | AF488 | Biolegend | 628502 | Purified anti-Cytokeratin 19 Antibody |
| CK5 (CKs) | AF488 | Biolegend | 905501 | Keratin 5 Polyclonal Antibody, Purified (1 mg/ml) 0.03% Thimerosal |
| CK7 (CKs) | AF488 | abcam | ab185048 | Anti-Cytokeratin 7 antibody [EPR1619Y] - Cytoskeleton Marker (Alexa Fluor 488) (ab185048) |
| CK8 (CKs) | AF488 | abcam | ab192467 | Anti-Cytokeratin 8 antibody [EP1628Y] (Alexa Fluor 488) (ab192467) |
| E-cadherin | AF555 | Abcam | ab206878 | Anti-E Cadherin antibody [EP700Y] - Intercellular Junction Marker (Alexa Fluor® 555) |
| GATA3 | AF750 | CST | 5852s | GATA-3 (D13C9) XP® Rabbit mAb #5852 |
| H3K27Me3 | AF488 | CST | 5499 | Tri-Methyl-Histone H3 (Lys27) (C36B11) Rabbit mAb (Alexa Fluor 488 Conjugate) #5499 |
| Ki67 | AF647 | CST | 12075S | Ki-67 (D3B5) Rabbit mAb (Alexa Fluor 647 Conjugate) #12075 |
| MMP9 | AF647 | R&D | AF911 | Human MMP-9 Antibody |
| p21 | AF647 | CST | 8587S | CST - p21 Waf1/Cip1 (12D1) Rabbit mAb (Alexa Fluor® 647 Conjugate) |
| p-4EBP1 | AF750 | Abnova | PAB16946 | EIF4EBP1 (phospho S65) polyclonal antibody |
| p-ATM | AF750 | Abcam | ab81292 | Anti-ATM (phospho S1981) antibody [EP1890Y] |
| p-ATR | AF488 | Abcam | ab230831 | Anti-ATR (phospho S428) antibody [EPR2184] - BSA and Azide free (ab230831) |
| p-CHK2 | AF750 | R&D | AF1626 | CHEK2 / CHK2 Antibody (phospho-Thr68) LS-C177890 |
| PCNA | AF488 | CST | 8580 | PCNA (PC10) Mouse mAb (Alexa Fluor 488 Conjugate) #8580 |
| PDGFR | AF555 | R&D | AF385 | Human PDGF R beta Antibody |
| p-EGFR | AF647 | R&D | AF1095 | Human Phospho-EGFR (Y1173) Antibody |

| | | | | |
|---|---|---|---|---|
| p-ERK | AF647 | R&D | AF1018 | Human/Mouse/Rat Phospho-ERK1(T202/Y204)/ERK2 (T185/Y187) Antibody |
| p-GSK3b | AF750 | CST | 9323S | Phospho-GSK-3ß (Ser9) (5B3) Rabbit mAb #9323 |
| p-H2AX | AF647 | CST | 9720S | Phospho-Histone H2A.X (Ser139) (20E3) Rabbit mAb (Alexa Fluor® 647 Conjugate) |
| p-HH3 | AF488 | CST | 3465S | Phospho-Histone H3 (Ser10) (D2C8) XP Rabbit mAb (Alexa Fluor 488 Conjugate) #3465 |
| p-P38 | AF750 | CST | 4511T | CST - Phospho-p38 MAPK (Thr180/Tyr182) (D3F9) XP® Rabbit mAb |
| p-PDK1 | AF750 | Thermofisher | PA5-37679 | Phospho-PDPK1 (Ser241) Antibody |
| p-RB | AF647 | abcam | ab215947 | Anti-Rb (phospho S807) antibody [EPR17732] (Alexa Fluor® 647) (ab215947) |
| p-S6 | AF555 | CST | 3985S | Phospho-S6 Ribosomal Protein (Ser235/236) (D57.2.2E) XP Rabbit mAb (Alexa Fluor 555 Conjugate) #3985 |
| PTEN | AF555 | R&D | AF847 | Human/Mouse/Rat PTEN Antibody |
| Rad51 | AF488 | abcam | ab196449 | Anti-Rad51 antibody [EPR4030(3)] (Alexa Fluor® 488) |
| RRM2 | AF488 | Abcam | ab215941 | Anti-RRM2 antibody [EPR11820] (Alexa Fluor® 488) |
| SMAD3 | AF555 | R&D | MAB4038 | Human Smad3 Antibody |
| Sting | AF647 | Abcam | ab198952 | Anti-TMEM173 antibody [EPR13130] (Alexa Fluor® 647) |
| TP53 | AF488 | Bioss | bs-8687R-A488 | p53 (FL-393) Polyclonal Antibody, ALEXA FLUOR® 488 Conjugated |
| TSC1 | AF750 | Abcam | ab40872 | Anti-Hamartin antibody [EP318Y] KO Tested Recombinant |
| Vimentin | AF488 | CST | 9854 | Vimentin (D21H3) XP Rabbit mAb (Alexa Fluor 488 Conjugate) #9854 |
| YAP1 | AF750 | R&D | MAB8094 | Human/Mouse/Rat YAP1 Antibody |

**Supplementary Table 4: Time of completion of the CLIA assays.** The time of completion was measured from the time of sample collection to the time of clinical report completion. N/a: no data available due to low tumor content.

| Patient | Treatment | Report Name | Time |
|---|---|---|---|
| Case 1 | Pre | GeneTrails | 106 |
| | | MSI | 14 |
| | | IHC | 12 |
| | On | GeneTrails | 9 |
| | | MSI | 8 |
| | | IHC | 11 |
| Case 2 | Pre | GeneTrails | 14 |
| | | MSI | 19 |
| | | IHC | 7 |
| | On | GeneTrails | 15 |
| | | MSI | 11 |
| | | IHC | 14 |
| Case 3 | Pre | GeneTrails | 10 |
| | | MSI | 9 |
| | | IHC | 29 |
| | On | GeneTrails | n/a |
| | | MSI | n/a |
| | | IHC | 14 |

n/a: no data available. The assay were not performed due to low tumor content.

**Supplementary Table 5: List of SNVs detected through WES.**

| Patient | Hugo_Symbol | Description | Protein_Change | cDNA_Change | t_FA | Variant_Classification |
|---|---|---|---|---|---|---|
| 1 | ZSWIM8 | zinc finger, SWIM-type containing 8 | p.P1495A | c.4483C>G | 0.776296296 | Missense_Mutation |
| 1 | ZSWIM8 | zinc finger, SWIM-type containing 8 | p.H303L | c.908A>T | 0.743089431 | Missense_Mutation |
| 1 | TP53 | tumor protein p53 | p.M237I | c.711G>A | 0.456896552 | Missense_Mutation |
| 1 | GRIN2B | glutamate receptor, ionotropic, N-methyl D-aspartate 2B | p.V1438M | c.4312G>A | 0.43231441 | Missense_Mutation |
| 1 | KCNA4 | potassium voltage-gated channel, shaker-related subfamily, member 4 | p.A46V | c.137C>T | 0.348837209 | Missense_Mutation |
| 1 | LDB2 | LIM domain binding 2 | p.P351S | c.1051C>T | 0.346153846 | Missense_Mutation |
| 1 | RALGAPA2 | Ral GTPase activating protein, alpha subunit 2 (catalytic) | p.Q1658* | c.4972C>T | 0.318302387 | Nonsense_Mutation |
| 1 | SCN1A | sodium channel, voltage-gated, type I, alpha subunit | p.I1701L | c.5101A>C | 0.313901345 | Missense_Mutation |
| 1 | DOCK2 | dedicator of cytokinesis 2 | p.G987R | c.2959G>A | 0.313868613 | Missense_Mutation |
| 1 | F13A1 | coagulation factor XIII, A1 polypeptide | p.R704W | c.2110C>T | 0.286419753 | Missense_Mutation |
| 1 | VPS13D | vacuolar protein sorting 13 homolog D (S. cerevisiae) | p.S596C | c.1786A>T | 0.23125 | Missense_Mutation |
| 1 | LOXL3 | lysyl oxidase-like 3 | p.A360T | c.1078G>A | 0.217213115 | Missense_Mutation |
| 1 | NEB | nebulin | p.R6535Q | c.19604G>A | 0.187878788 | Missense_Mutation |
| 1 | ALPPL2 | alkaline phosphatase, placental-like 2 | p.P509R | c.1526C>G | 0.162162162 | Missense_Mutation |
| 1 | SRCIN1 | SRC kinase signaling inhibitor 1 | p.P475L | c.1424C>T | 0.15625 | Missense_Mutation |
| 1 | SPACA1 | sperm acrosome associated 1 | p.R28H | c.83G>A | 0.144144144 | Missense_Mutation |
| 1 | USP21 | ubiquitin specific peptidase 21 | p.N533K | c.1599T>A | 0.141666667 | Missense_Mutation |
| 1 | REPS2 | RALBP1 associated Eps domain containing 2 | p.R655C | c.1963C>T | 0.141304348 | Missense_Mutation |
| 1 | ZFHX4 | zinc finger homeobox 4 | p.E130* | c.388G>T | 0.131455399 | Nonsense_Mutation |
| 1 | PUS10 | pseudouridylate synthase 10 | p.E517K | c.1549G>A | 0.125827815 | Missense_Mutation |
| 1 | RHPN2 | rhophilin, Rho GTPase binding protein 2 | p.R406Q | c.1217G>A | 0.125 | Missense_Mutation |
| 1 | SUV420H2 | suppressor of variegation 4-20 homolog 2 (Drosophila) | p.E212K | c.634G>A | 0.1234375 | Missense_Mutation |
| 1 | PIK3CG | phosphatidylinositol-4,5-bisphosphate 3-kinase, catalytic subunit gamma | p.G790A | c.2369G>C | 0.112068966 | Missense_Mutation |
| 1 | PDCD5 | programmed cell death 5 | p.D26V | c.77A>T | 0.104477612 | Missense_Mutation |
| 1 | KMT2C | lysine (K)-specific methyltransferase 2C | p.S764F | c.2291C>T | 0.082474227 | Missense_Mutation |
| 1 | PASD1 | PAS domain containing 1 | p.D83H | c.247G>C | 0.08 | Missense_Mutation |
| 1 | SDC4 | syndecan 4 | p.D67G | c.200A>G | 0.076923077 | Splice_Site |
| 1 | KIF18B | kinesin family member 18B | p.A524E | c.1571C>A | 0.068965517 | Missense_Mutation |
| 1 | USO1 | USO1 vesicle transport factor | p.S422L | c.1265C>T | 0.068627451 | Missense_Mutation |
| 1 | SLC25A5 | solute carrier family 25 (mitochondrial carrier; adenine nucleotide translocator), member 5 | p.R236P | c.707G>C | 0.034482759 | Missense_Mutation |
| 2 | OSGEPL1 | O-sialoglycoprotein endopeptidase-like 1 | p.E268* | c.802G>T | 0.384615385 | Nonsense_Mutation |
| 2 | TLL2 | tolloid-like 2 | p.E822Q | c.2464G>C | 0.305555556 | Missense_Mutation |
| 2 | CATSPER2 | cation channel, sperm associated 2 | p.R513H | c.1538G>A | 0.294642857 | Missense_Mutation |
| 2 | ADAMTS4 | ADAM metallopeptidase with thrombospondin type 1 motif, 4 | p.K598R | c.1793A>G | 0.291390728 | Missense_Mutation |
| 2 | TFDP2 | transcription factor Dp-2 (E2F dimerization partner 2) | p.E118Q | c.352G>C | 0.277777778 | Missense_Mutation |
| 2 | CCDC74A | coiled-coil domain containing 74A | p.R294Q | c.881G>A | 0.268656716 | Missense_Mutation |
| 2 | IQSEC2 | IQ motif and Sec7 domain 2 | p.M947K | c.2840T>A | 0.247787611 | Missense_Mutation |
| 2 | SDK2 | sidekick cell adhesion molecule 2 | p.N1603K | c.4809C>A | 0.215517241 | Missense_Mutation |
| 2 | FRY | furry homolog (Drosophila) | p.K1020R | c.3059A>G | 0.214285714 | Missense_Mutation |
| 2 | KDR | kinase insert domain receptor (a type III receptor tyrosine kinase) | p.E1240Q | c.3718G>C | 0.203389831 | Missense_Mutation |
| 2 | TOM1L2 | target of myb1-like 2 (chicken) | p.N329K | c.987C>A | 0.2 | Missense_Mutation |
| 2 | TTN | titin | p.V13878D | c.41633T>A | 0.192513369 | Missense_Mutation |
| 2 | PRPF4B | pre-mRNA processing factor 4B | p.R464T | c.1391G>C | 0.19047619 | Missense_Mutation |
| 2 | HLA-B | major histocompatibility complex, class I, B | p.Y140F | c.419A>T | 0.189655172 | Missense_Mutation |
| 2 | RIOK3 | RIO kinase 3 | p.Y88F | c.263A>T | 0.181818182 | Missense_Mutation |
| 2 | CCDC180 | coiled-coil domain containing 180 | p.N1237H | c.3709A>C | 0.177835052 | Missense_Mutation |

| | | | | | | | |
|---|---|---|---|---|---|---|---|
| 2 | TENM2 | teneurin transmembrane protein 2 | p.V1105L | c.3313G>C | 0.174863388 | Missense_Mutation |
| 2 | ANGEL2 | angel homolog 2 (Drosophila) | p.H493Y | c.1477C>T | 0.174757282 | Missense_Mutation |
| 2 | PCNXL4 | pecanex-like 4 (Drosophila) | p.D342N | c.1024G>A | 0.173913043 | Missense_Mutation |
| 2 | C3orf20 | chromosome 3 open reading frame 20 | p.L885I | c.2653C>A | 0.172413793 | Missense_Mutation |
| 2 | SEC23B | Sec23 homolog B (S. cerevisiae) | p.S115F | c.344C>T | 0.165413534 | Missense_Mutation |
| 2 | ACACB | acetyl-CoA carboxylase beta | p.M2218I | c.6654G>C | 0.163265306 | Missense_Mutation |
| 2 | RASAL2 | RAS protein activator like 2 | p.Q81* | c.241C>T | 0.16224649 | Nonsense_Mutation |
| 2 | MMP1 | matrix metallopeptidase 1 (interstitial collagenase) | p.G225A | c.674G>C | 0.160583942 | Missense_Mutation |
| 2 | PYGO1 | pygopus family PHD finger 1 | p.R278* | c.832C>T | 0.158536585 | Nonsense_Mutation |
| 2 | CCDC33 | coiled-coil domain containing 33 | p.V200F | c.598G>T | 0.146067416 | Missense_Mutation |
| 2 | NACAD | NAC alpha domain containing | p.E530* | c.1588G>T | 0.144736842 | Nonsense_Mutation |
| 2 | TMBIM6 | transmembrane BAX inhibitor motif containing 6 | p.K232N | c.696G>C | 0.139534884 | Missense_Mutation |
| 2 | LCMT1 | leucine carboxyl methyltransferase 1 | p.A72V | c.215C>T | 0.139240506 | Missense_Mutation |
| 2 | DNAJC30 | DnaJ (Hsp40) homolog, subfamily C, member 30 | p.D205N | c.613G>A | 0.138461538 | Missense_Mutation |
| 2 | HLA-B | major histocompatibility complex, class I, B | p.H137Y | c.409C>T | 0.137931034 | Missense_Mutation |
| 2 | ASAH1 | N-acylsphingosine amidohydrolase (acid ceramidase) 1 | p.E290Q | c.868G>C | 0.1375 | Missense_Mutation |
| 2 | SORBS2 | sorbin and SH3 domain containing 2 | p.E729Q | c.2185G>C | 0.132978723 | Missense_Mutation |
| 2 | CAND2 | cullin-associated and neddylation-dissociated 2 (putative) | p.LIRE1067del | c.3199_3210de | 0.132183908 | Splice_Site |
| 2 | MYO3A | myosin IIIA | p.Y929* | c.2787T>A | 0.131578947 | Nonsense_Mutation |
| 2 | GRM8 | glutamate receptor, metabotropic 8 | p.M412V | c.1234A>G | 0.128888889 | Missense_Mutation |
| 2 | EXOC6 | exocyst complex component 6 | p.E611Q | c.1831G>C | 0.126126126 | Missense_Mutation |
| 2 | FEM1B | fem-1 homolog b (C. elegans) | p.S454Y | c.1361C>A | 0.125 | Missense_Mutation |
| 2 | OGDHL | oxoglutarate dehydrogenase-like | p.D113Y | c.337G>T | 0.124378109 | Missense_Mutation |
| 2 | PLCH1 | phospholipase C, eta 1 | p.F281I | c.841T>A | 0.122807018 | Missense_Mutation |
| 2 | BPIFB6 | BPI fold containing family B, member 6 | p.P425Q | c.1274C>A | 0.117647059 | Missense_Mutation |
| 2 | ELMO1 | engulfment and cell motility 1 | p.E661K | c.1981G>A | 0.115207373 | Missense_Mutation |
| 2 | PATZ1 | POZ (BTB) and AT hook containing zinc finger 1 | p.R456L | c.1367G>T | 0.1125 | Missense_Mutation |
| 2 | DNAH9 | dynein, axonemal, heavy chain 9 | p.E466* | c.1396G>T | 0.107526882 | Nonsense_Mutation |
| 2 | SLC7A10 | solute carrier family 7 (neutral amino acid transporter light chain, asc system), member 10 | p.L128V | c.382C>G | 0.106060606 | Missense_Mutation |
| 2 | DGUOK | deoxyguanosine kinase | p.R12* | c.34C>T | 0.102076125 | Nonsense_Mutation |
| 2 | MIA3 | melanoma inhibitory activity family, member 3 | p.K674N | c.2022G>C | 0.099236641 | Missense_Mutation |
| 2 | C19orf44 | chromosome 19 open reading frame 44 | p.S78L | c.233C>T | 0.09765625 | Missense_Mutation |
| 2 | PNPLA1 | patatin-like phospholipase domain containing 1 | p.S267C | c.800C>G | 0.096969697 | Missense_Mutation |
| 2 | GPR139 | G protein-coupled receptor 139 | p.A6D | c.17C>A | 0.096774194 | Missense_Mutation |
| 2 | IBSP | integrin-binding sialoprotein | p.D97N | c.289G>A | 0.096153846 | Missense_Mutation |
| 2 | SNAPC4 | small nuclear RNA activating complex, polypeptide 4, 190kDa | p.S836* | c.2507C>A | 0.095808383 | Nonsense_Mutation |
| 2 | KIAA1377 | KIAA1377 | p.N638K | c.1914T>A | 0.095238095 | Missense_Mutation |
| 2 | LIPE | lipase, hormone-sensitive | p.L758V | c.2272C>G | 0.092783505 | Missense_Mutation |
| 2 | EIF3M | eukaryotic translation initiation factor 3, subunit M | p.V245G | c.734T>G | 0.090909091 | Missense_Mutation |
| 2 | KCNK10 | potassium channel, subfamily K, member 10 | p.G448R | c.1342G>C | 0.089686099 | Missense_Mutation |
| 2 | CCNF | cyclin F | p.T181S | c.542C>G | 0.088050314 | Splice_Site |
| 2 | PURB | purine-rich element binding protein B | p.L75V | c.223C>G | 0.085192698 | Missense_Mutation |
| 2 | CHPF | chondroitin polymerizing factor | p.D193H | c.577G>C | 0.083591331 | Missense_Mutation |
| 2 | F5 | coagulation factor V (proaccelerin, labile factor) | p.T199A | c.595A>G | 0.079051383 | Missense_Mutation |
| 2 | PLCB2 | phospholipase C, beta 2 | p.E1028Q | c.3082G>C | 0.07518797 | Missense_Mutation |
| 2 | LIG1 | ligase I, DNA, ATP-dependent | p.P52A | c.154C>G | 0.074906367 | Missense_Mutation |
| 2 | FBN3 | fibrillin 3 | p.C1221S | c.3662G>C | 0.071794872 | Missense_Mutation |

| | | | | | | |
|---|---|---|---|---|---|---|
| 2 | ADAMTS16 | ADAM metallopeptidase with thrombospondin type 1 motif, 16 | p.C518S | c.1553G>C | 0.071428571 | Missense_Mutation |
| 2 | RBAK | RB-associated KRAB zinc finger | p.E342D | c.1026G>C | 0.07079646 | Missense_Mutation |
| 2 | LGALS14 | lectin, galactoside-binding, soluble, 14 | p.I130M | c.390C>G | 0.0703125 | Missense_Mutation |
| 2 | NOS1 | nitric oxide synthase 1 (neuronal) | p.E1414V | c.4241A>T | 0.068965517 | Missense_Mutation |
| 2 | LRGUK | leucine-rich repeats and guanylate kinase domain containing | p.439_445STYF | c.1317_1334de | 0.06185567 | Splice_Site |
| 2 | FGFRL1 | fibroblast growth factor receptor-like 1 | p.E289K | c.865G>A | 0.061403509 | Missense_Mutation |
| 2 | GLYATL1 | glycine-N-acyltransferase-like 1 | p.C287* | c.861T>A | 0.058252427 | Nonsense_Mutation |
| 2 | HSH2D | hematopoietic SH2 domain containing | p.L230F | c.690G>T | 0.057803468 | Missense_Mutation |
| 2 | CDH3 | cadherin 3, type 1, P-cadherin (placental) | p.E399Q | c.1195G>C | 0.057522124 | Missense_Mutation |
| 2 | FOXP1 | forkhead box P1 | p.R544* | c.1630C>T | 0.056818182 | Nonsense_Mutation |
| 2 | CLASP1 | cytoplasmic linker associated protein 1 | p.S37C | c.110C>G | 0.052083333 | Missense_Mutation |
| 2 | CPSF1 | cleavage and polyadenylation specific factor 1, 160kDa | p.N24S | c.71A>G | 0.050793651 | Missense_Mutation |
| 2 | CARD6 | caspase recruitment domain family, member 6 | p.D31N | c.91G>A | 0.047368421 | Missense_Mutation |
| 2 | GRTP1 | growth hormone regulated TBC protein 1 | p.E21Q | c.61G>C | 0.045283019 | Missense_Mutation |
| 2 | SLC46A2 | solute carrier family 46, member 2 | p.V303L | c.907G>C | 0.039735099 | Missense_Mutation |
| 2 | TMEM184B | transmembrane protein 184B | p.D296G | c.887A>G | 0.037815126 | Missense_Mutation |
| 2 | C5orf60 | chromosome 5 open reading frame 60 | p.V44I | c.129_130TG>( | 0.037800687 | Missense_Mutation |
| 2 | FLG2 | filaggrin family member 2 | p.S743A | c.2227T>G | 0.026350461 | Missense_Mutation |
| 3 | ADRA1B | adrenoceptor alpha 1B | p.L68M | c.202T>A | 0.828897338 | Missense_Mutation |
| 3 | ZFYVE1 | zinc finger, FYVE domain containing 1 | p.Y523* | c.1569C>G | 0.827411168 | Nonsense_Mutation |
| 3 | ZBTB48 | zinc finger and BTB domain containing 48 | p.C324R | c.970T>C | 0.818791946 | Missense_Mutation |
| 3 | LZTR1 | leucine-zipper-like transcription regulator 1 | p.P308S | c.922C>T | 0.807909605 | Missense_Mutation |
| 3 | KRT79 | keratin 79 | p.T475I | c.1424C>T | 0.762376238 | Missense_Mutation |
| 3 | BCAT2 | branched chain amino-acid transaminase 2, mitochondrial | p.I152M | c.456C>G | 0.708994709 | Missense_Mutation |
| 3 | PELP1 | proline, glutamate and leucine rich protein 1 | p.L369Q | c.1106T>A | 0.696629213 | Missense_Mutation |
| 3 | HPS4 | Hermansky-Pudlak syndrome 4 | p.S526T | c.1577G>C | 0.693661972 | Missense_Mutation |
| 3 | FAM65A | family with sequence similarity 65, member A | p.L78V | c.232C>G | 0.691358025 | Missense_Mutation |
| 3 | KMT2B | lysine (K)-specific methyltransferase 2B | p.A1541V | c.4622C>T | 0.666666667 | Missense_Mutation |
| 3 | OXGR1 | oxoglutarate (alpha-ketoglutarate) receptor 1 | p.L184V | c.550C>G | 0.66025641 | Missense_Mutation |
| 3 | IL1RAPL2 | interleukin 1 receptor accessory protein-like 2 | p.E605D | c.1815A>T | 0.637096774 | Missense_Mutation |
| 3 | RAPGEF1 | Rap guanine nucleotide exchange factor (GEF) 1 | p.Q660R | c.1979A>G | 0.623376623 | Missense_Mutation |
| 3 | TNNT1 | troponin T type 1 (skeletal, slow) | p.W277S | c.830G>C | 0.617283951 | Missense_Mutation |
| 3 | EFNB3 | ephrin-B3 | p.G173V | c.518G>T | 0.591549296 | Missense_Mutation |
| 3 | DCLK1 | doublecortin-like kinase 1 | p.D79E | c.237C>A | 0.557377049 | Missense_Mutation |
| 3 | KRTAP13-3 | keratin associated protein 13-3 | p.S141R | c.423T>A | 0.543478261 | Missense_Mutation |
| 3 | SIGLEC12 | sialic acid binding Ig-like lectin 12 (gene/pseudogene) | p.R92* | c.274C>T | 0.518731988 | Nonsense_Mutation |
| 3 | SF3B5 | splicing factor 3b, subunit 5, 10kDa | p.S9R | c.27C>G | 0.512776831 | Missense_Mutation |
| 3 | LDLRAD3 | low density lipoprotein receptor class A domain containing 3 | p.P77A | c.229C>G | 0.487603306 | Missense_Mutation |
| 3 | PSMD3 | proteasome (prosome, macropain) 26S subunit, non-ATPase, 3 | p.L191V | c.571C>G | 0.46875 | Missense_Mutation |
| 3 | KIAA0368 | KIAA0368 | p.L318F | c.954G>T | 0.435483871 | Missense_Mutation |
| 3 | APBA1 | amyloid beta (A4) precursor protein-binding, family A, member 1 | p.V763I | c.2287G>A | 0.430379747 | Missense_Mutation |
| 3 | KIF1A | kinesin family member 1A | p.N1054S | c.3161A>G | 0.425287356 | Splice_Site |
| 3 | STEAP1B | STEAP family member 1B | p.V122L | c.364G>C | 0.421052632 | Missense_Mutation |
| 3 | CDK5RAP2 | CDK5 regulatory subunit associated protein 2 | p.I539M | c.1617C>G | 0.417021277 | Missense_Mutation |

| | | | | | | |
|---|---|---|---|---|---|---|
| 3 | NDUFB2 | NADH dehydrogenase (ubiquinone) 1 beta subcomplex, 2, 8kDa | p.H75N | c.223C>A | 0.415254237 | Missense_Mutation |
| 3 | MVB12A | multivesicular body subunit 12A | p.D116E | c.348C>G | 0.412811388 | Missense_Mutation |
| 3 | RALGAPA2 | Ral GTPase activating protein, alpha subunit 2 (catalytic) | p.E1184D | c.3552G>C | 0.409090909 | Missense_Mutation |
| 3 | MBTD1 | mbt domain containing 1 | p.K576N | c.1728G>C | 0.407407407 | Missense_Mutation |
| 3 | CTNNA3 | catenin (cadherin-associated protein), alpha 3 | p.S743* | c.2228C>A | 0.402985075 | Nonsense_Mutation |
| 3 | ASCL1 | achaete-scute family bHLH transcription factor 1 | p.K100Q | c.298A>C | 0.402684564 | Missense_Mutation |
| 3 | ANKEF1 | ankyrin repeat and EF-hand domain containing 1 | p.D464E | c.1392T>G | 0.396551724 | Missense_Mutation |
| 3 | NEB | nebulin | p.Y4561* | c.13683C>G | 0.395833333 | Nonsense_Mutation |
| 3 | GIGYF1 | GRB10 interacting GYF protein 1 | p.S412C | c.1235C>G | 0.395061728 | Missense_Mutation |
| 3 | PHF21B | PHD finger protein 21B | p.K177Q | c.529A>C | 0.394285714 | Missense_Mutation |
| 3 | SLC3A1 | solute carrier family 3 (amino acid transporter heavy chain), member 1 | p.R83H | c.248G>A | 0.392592593 | Missense_Mutation |
| 3 | YTHDF1 | YTH domain family, member 1 | p.S555R | c.1663A>C | 0.389671362 | Missense_Mutation |
| 3 | TRIM23 | tripartite motif containing 23 | p.R289* | c.865C>T | 0.388535032 | Nonsense_Mutation |
| 3 | TBX3 | T-box 3 | p.D27E | c.81C>G | 0.385964912 | Missense_Mutation |
| 3 | POLK | polymerase (DNA directed) kappa | p.Y50S | c.149A>C | 0.383458647 | Missense_Mutation |
| 3 | AP000783.1 | | p.R104G | c.310C>G | 0.379790941 | Missense_Mutation |
| 3 | LINGO2 | leucine rich repeat and Ig domain containing 2 | p.S137I | c.410G>T | 0.37944664 | Missense_Mutation |
| 3 | NLRP8 | NLR family, pyrin domain containing 8 | p.Q254K | c.760C>A | 0.371702638 | Missense_Mutation |
| 3 | NEB | nebulin | p.R4376K | c.13127G>A | 0.371134021 | Missense_Mutation |
| 3 | CDC42BPG | CDC42 binding protein kinase gamma (DMPK-like) | p.S1154T | c.3461G>C | 0.365591398 | Missense_Mutation |
| 3 | RUFY4 | RUN and FYVE domain containing 4 | p.L543M | c.1627C>A | 0.360655738 | Missense_Mutation |
| 3 | AOX1 | aldehyde oxidase 1 | p.V1090A | c.3269T>C | 0.354166667 | Missense_Mutation |
| 3 | RPN2 | ribophorin II | p.S121P | c.361T>C | 0.350993377 | Missense_Mutation |
| 3 | TTC30A | tetratricopeptide repeat domain 30A | p.D648E | c.1944T>G | 0.349056604 | Missense_Mutation |
| 3 | KDM4B | lysine (K)-specific demethylase 4B | p.A827V | c.2480C>T | 0.333333333 | Missense_Mutation |
| 3 | ZHX3 | zinc fingers and homeoboxes 3 | p.G915R | c.2743G>A | 0.333333333 | Missense_Mutation |
| 3 | NEDD4 | neural precursor cell expressed, developmentally down-regulated 4, E3 ubiquitin protein ligase | p.T645fs | c.1932_1934de | 0.305263158 | Splice_Site |
| 3 | PCLO | piccolo presynaptic cytomatrix protein | p.P1768T | c.5302C>A | 0.303278689 | Missense_Mutation |
| 3 | RAB11FIP3 | RAB11 family interacting protein 3 (class II) | p.R155P | c.464G>C | 0.3 | Missense_Mutation |
| 3 | ALKBH7 | alkB, alkylation repair homolog 7 (E. coli) | p.A94S | c.280G>T | 0.294964029 | Missense_Mutation |
| 3 | ADAR | adenosine deaminase, RNA-specific | p.V97M | c.289G>A | 0.291497976 | Missense_Mutation |
| 3 | TTC17 | tetratricopeptide repeat domain 17 | p.M797I | c.2391G>A | 0.287037037 | Missense_Mutation |
| 3 | SUCNR1 | succinate receptor 1 | p.P156H | c.467C>A | 0.286919831 | Missense_Mutation |
| 3 | HS3ST2 | heparan sulfate (glucosamine) 3-O-sulfotransferase 2 | p.R350G | c.1048A>G | 0.283687943 | Missense_Mutation |
| 3 | ITPKB | inositol-trisphosphate 3-kinase B | p.M704V | c.2110A>G | 0.27294686 | Missense_Mutation |
| 3 | NCDN | neurochondrin | p.D459H | c.1375G>C | 0.263736264 | Missense_Mutation |
| 3 | HNRNPU | heterogeneous nuclear ribonucleoprotein U (scaffold attachment factor A) | p.S326T | c.976T>A | 0.262068966 | Missense_Mutation |
| 3 | IL6R | interleukin 6 receptor | p.T57S | c.170C>G | 0.258992806 | Missense_Mutation |
| 3 | LRRC53 | leucine rich repeat containing 53 | p.G352A | c.1055G>C | 0.25 | Missense_Mutation |
| 3 | SLC41A1 | solute carrier family 41 (magnesium transporter), member 1 | p.R407H | c.1220G>A | 0.246753247 | Missense_Mutation |
| 3 | RCSD1 | RCSD domain containing 1 | p.G328A | c.983G>C | 0.245398773 | Missense_Mutation |
| 3 | PKLR | pyruvate kinase, liver and RBC | p.V280M | c.838G>A | 0.243678161 | Missense_Mutation |
| 3 | C1orf147 | chromosome 1 open reading frame 147 | p.L265V | c.793C>G | 0.233333333 | Missense_Mutation |
| 3 | ARHGAP30 | Rho GTPase activating protein 30 | p.R167C | c.499C>T | 0.229437229 | Missense_Mutation |
| 3 | OR2A5 | olfactory receptor, family 2, subfamily A, member 5 | p.L154P | c.461T>C | 0.229299363 | Missense_Mutation |

| | | | | | | | |
|---|---|---|---|---|---|---|---|
| 3 | MYH7 | myosin, heavy chain 7, cardiac muscle, beta | p.SGHPDSAGR1 | c.4954_4980de | 0.226890756 | Splice_Site |
| 3 | CYB5RL | cytochrome b5 reductase-like | p.D297H | c.889G>C | 0.218045113 | Missense_Mutation |
| 3 | SYNE1 | spectrin repeat containing, nuclear envelope 1 | p.S157G | c.469A>G | 0.206060606 | Missense_Mutation |
| 3 | DMRTA2 | DMRT-like family A2 | p.D164Y | c.490G>T | 0.201342282 | Missense_Mutation |
| 3 | ZNF141 | zinc finger protein 141 | p.K353V | c.1057_1059AA | 0.158940397 | Missense_Mutation |
| 3 | BTNL9 | butyrophilin-like 9 | p.H389Y | c.1165C>T | 0.133333333 | Missense_Mutation |
| 3 | CNOT4 | CCR4-NOT transcription complex, subunit 4 | p.S280A | c.838T>G | 0.113636364 | Missense_Mutation |
| 3 | EPHB2 | EPH receptor B2 | p.D397V | c.1190A>T | 0.108108108 | Missense_Mutation |
| 3 | DOLPP1 | dolichyldiphosphatase 1 | | | 0.098214286 | Splice_Site |
| 3 | WDFY3 | WD repeat and FYVE domain containing 3 | p.D1484N | c.4450G>A | 0.08045977 | Missense_Mutation |
| 3 | WNK2 | WNK lysine deficient protein kinase 2 | p.L1024V | c.3070C>G | 0.079207921 | Missense_Mutation |
| 3 | RANBP6 | RAN binding protein 6 | p.A3S | c.6_7GG>TT | 0.067961165 | Missense_Mutation |
| 3 | ZNF609 | zinc finger protein 609 | p.R1410M | c.4229G>T | 0.067567568 | Missense_Mutation |
| 3 | NTN1 | netrin 1 | p.R582L | c.1745G>T | 0.066037736 | Missense_Mutation |
| 3 | PLD4 | phospholipase D family, member 4 | p.S76C | c.227C>G | 0.064220183 | Missense_Mutation |
| 3 | PEX16 | peroxisomal biogenesis factor 16 | p.P152L | c.455C>T | 0.058823529 | Missense_Mutation |
| 3 | EMR2 | egf-like module containing, mucin-like, hormone receptor-like 2 | p.K154I | c.461A>T | 0.056 | Missense_Mutation |
| 3 | EMR2 | egf-like module containing, mucin-like, hormone receptor-like 2 | p.L153F | c.457C>T | 0.049180328 | Missense_Mutation |
| 3 | PYGL | phosphorylase, glycogen, liver | p.K796E | c.2386A>G | 0.047619048 | Missense_Mutation |
| 3 | ANKRD36 | ankyrin repeat domain 36 | p.K1149N | c.3447G>T | 0.044117647 | Missense_Mutation |
| 3 | PABPC3 | poly(A) binding protein, cytoplasmic 3 | p.C132G | c.394T>G | 0.043956044 | Missense_Mutation |
| 3 | ARHGEF18 | Rho/Rac guanine nucleotide exchange factor (GEF) 18 | p.G233C | c.697G>T | 0.043859649 | Missense_Mutation |
| 3 | RP11-817J15.3 | | p.R48G | c.142A>G | 0.043859649 | Missense_Mutation |
| 3 | CBX8 | chromobox homolog 8 | p.D149H | c.445G>C | 0.037735849 | Missense_Mutation |
| 3 | PRSS1 | protease, serine, 1 (trypsin 1) | p.D218Y | c.651_652TG>C | 0.03649635 | Missense_Mutation |
| 3 | TOP2A | topoisomerase (DNA) II alpha 170kDa | p.E203D | c.609G>C | 0.035714286 | Missense_Mutation |
| 3 | NACC1 | nucleus accumbens associated 1, BEN and BTB (POZ) domain containing | p.R165L | c.494G>T | 0.033783784 | Missense_Mutation |
| 3 | CDC34 | cell division cycle 34 | p.R159L | c.476G>T | 0.032258065 | Missense_Mutation |
| 3 | SMARCA2 | SWI/SNF related, matrix associated, actin dependent regulator of chromatin, subfamily a, member 2 | p.E1224Q | c.3670G>C | 0.029585799 | Missense_Mutation |
| 3 | SIGLEC10 | sialic acid binding Ig-like lectin 10 | p.P647Q | c.1940C>A | 0.02955665 | Missense_Mutation |
| 3 | FAM78B | family with sequence similarity 78, member B | p.R54S | c.160C>A | 0.028037383 | Missense_Mutation |
| 3 | SACS | sacsin molecular chaperone | p.P45Q | c.134C>A | 0.02617801 | Missense_Mutation |
| 3 | SEMG1 | semenogelin I | p.R372S | c.1114C>A | 0.026086957 | Missense_Mutation |
| 3 | DLL4 | delta-like 4 (Drosophila) | p.G47W | c.139G>T | 0.025069638 | Missense_Mutation |
| 3 | HIVEP2 | human immunodeficiency virus type I enhancer binding protein 2 | p.P1145Q | c.3434C>A | 0.0243309 | Missense_Mutation |
| 3 | FN3K | fructosamine 3 kinase | p.R294L | c.881G>T | 0.024193548 | Missense_Mutation |
| 3 | GOLGB1 | golgin B1 | p.H1361N | c.4081C>A | 0.023121387 | Missense_Mutation |
| 3 | SRRM2 | serine/arginine repetitive matrix 2 | p.R1809L | c.5426G>T | 0.02283105 | Missense_Mutation |
| 3 | SMIM11 | small integral membrane protein 11 | p.G42W | c.124G>T | 0.021472393 | Missense_Mutation |
| 3 | MRC2 | mannose receptor, C type 2 | p.W1047L | c.3140G>T | 0.018480493 | Missense_Mutation |
| 3 | ANO1 | anoctamin 1, calcium activated chloride channel | p.W910L | c.2729G>T | 0.018306636 | Missense_Mutation |
| 3 | KIAA0195 | KIAA0195 | p.R953L | c.2858G>T | 0.018087855 | Missense_Mutation |
| 3 | MCM5 | minichromosome maintenance complex component 5 | p.G239W | c.715G>T | 0.016666667 | Missense_Mutation |
| 3 | SPRTN | SprT-like N-terminal domain | p.W36L | c.107G>T | 0.01510574 | Missense_Mutation |
| 3 | TULP4 | tubby like protein 4 | p.M1187I | c.3561G>T | 0.014376997 | Missense_Mutation |
| 3 | SOX9 | SRY (sex determining region Y)-box 9 | p.S228S | c.684G>T | 0.012805588 | Splice_Site |
| 3 | FLG2 | filaggrin family member 2 | p.H1632N | c.4894C>A | 0.011904762 | Missense_Mutation |

**Supplementary Table 6: List of CNVs detected through the CNV kit.**

| Patient | chromosome | start | end | gene | log2 | depth | probes | weight |
|---|---|---|---|---|---|---|---|---|
| 1 | 11 | 72291930 | 72354068 | PDE2A,PDE2A,MIR139 | 3.88971 | 3992.55 | 26 | 16.2983 |
| 1 | 11 | 68855924 | 69513518 | MYEOV,CCND1,ORAOV1 | 3.77607 | 3547.24 | 29 | 18.3142 |
| 1 | 11 | 70858931 | 71156031 | DHCR7 | 3.64678 | 3253.88 | 13 | 8.62797 |
| 1 | 11 | 72146022 | 72282263 | LINC01537 | 2.76665 | 1544.34 | 3 | 2.12059 |
| 1 | 11 | 69590404 | 69948650 | FGF3,ANO1 | 2.65215 | 1819.78 | 13 | 7.50079 |
| 1 | 10 | 63519734 | 63964818 | CABCOCO1,ARID5B,RTKN2 | 2.53355 | 1625.93 | 33 | 20.6256 |
| 1 | 10 | 79814446 | 81106855 | RPS24,ZMIZ1-AS1,ZMIZ1 | 2.21377 | 1149.52 | 45 | 28.2523 |
| 1 | 10 | 75523254 | 75560545 | SEC24C,FUT11,CHCHD1,ZSWIM8,ZSWIM8,ZSWIM8-AS1 | 2.04835 | 1242.65 | 57 | 38.5376 |
| 1 | 10 | 61828752 | 61836227 | ANK3 | 1.99357 | 1184.44 | 29 | 20.9377 |
| 1 | 19 | 56113435 | 56218448 | ZNF524,ZNF865,ZNF784,ZNF580,ZNF581,CCDC106,U2AF2,EPN1 | 1.97103 | 1243.96 | 57 | 36.5328 |
| 1 | 13 | 33284754 | 34013214 | PDS5B,KL,STARD13 | 1.83466 | 1069.26 | 62 | 38.8785 |
| 1 | 19 | 58639511 | 59069711 | ZNF329,ZNF274,ZNF544,ZNF8,ZNF8-ERVK3-1,ZSCAN22,A1BG,A1BG,A1BG-AS1,ZNF497,ZNF837,MIR4754,RPS5,RNF225,ZNF584,ZNF132,ZNF324B,ZNF324,ZNF446,SLC27A5,ZBTB45,TRIM28,TRIM28,MIR6807,CHMP2A,UBE2M | 1.82927 | 998.429 | 172 | 111.481 |
| 1 | 19 | 57835073 | 58374056 | ZNF543,ZNF304,TRAPPC2B,ZNF547,ZNF547,ZNF548,ZNF17,ZNF749,VN1R1,ZNF772,ZNF419,ZNF773,ZNF549,ZNF550,ZNF416,ZIK1,ZNF530,ZNF134,ZNF211,ZSCAN4,ZNF551,ZNF154,ZNF671,ZNF776,ZNF586,ZNF552,ZNF587B,ZNF587 | 1.82583 | 1113.89 | 253 | 157.531 |
| 1 | 19 | 49982178 | 50433568 | FLT3LG,RPL13A,RPL13AP5,RPL13A,RPL13AP5,SNORD32A,RPL13A,RPL13AP5,SNORD33,RPL13A,RPL13AP5,SNORD34,RPL13A,RPL13AP5,SNORD35A,RPS11,RPS11,SNORD35B,MIR150,FCGRT,RCN3,NOSIP,PRRG2,PRR12,RRAS,SCAF1,IRF3,IRF3,BCL2L12,BCL2L12,PRMT1,PRMT1,MIR5088,ADM5,CPT1C,TSKS,AP2A1,AP2A1,MIR6799,AP2A1,FUZ,FUZ,MED25,MED25,MIR6800,PTOV1-AS1,PTOV1-AS1,PTOV1,PTOV1,MIR4749,PTOV1,PTOV1,PTOV1-AS2,PNKP,AKT1S1,AKT1S1,TBC1D17,TBC1D17,TBC1D17,MIR4750,IL4I1,IL4I1,NUP62 | 1.81429 | 1067.56 | 285 | 180.602 |
| 1 | 10 | 76996200 | 77795175 | ZNF503,ZNF503,ZNF503-AS2,ZNF503-AS2,LRMDA,LRMDA,MIR606 | 1.78193 | 788.765 | 23 | 14.8592 |
| 1 | 19 | 54682514 | 54974573 | MBOAT7,TSEN34,RPS9,LILRB3,LILRB3,LILRA6,LILRB5,LILRB2,MIR4752,LILRA3,LILRA5,LILRA4,LAIR1,TTYH1,LENG8,LENG8,LENG9,LENG9 | 1.74043 | 1079.88 | 157 | 86.6146 |
| 1 | 10 | 63974694 | 65928219 | RTKN2,ZNF365,ADO,EGR2,NRBF2,JMJD1C,JMJD1C,MIR1296,JMJD1C,JMJD1C-AS1,JMJD1C,REEP3,REEP3 | 1.67414 | 1015.87 | 121 | 71.2761 |
| 1 | 19 | 55686575 | 56109255 | SYT5,PTPRH,TMEM86B,PPP6R1,MIR6804,PPP6R1,PPP6R1,MIR6802,PPP6R1,MIR6803,HSPBP1,BRSK1,TMEM150B,KMT5C,COX6B2,FAM71E2,IL11,TMEM190,TMEM238,RPL28,RPL28,MIR6805,UBE2S,SHISA7,ISOC2,ZNF628,NAT14,SSC5D,SBK2,SBK3,ZNF579,FIZ1 | 1.63621 | 822.377 | 217 | 133.534 |
| 1 | 11 | 70026033 | 70196228 | ANO1,FADD,PPFIA1,PPFIA1,MIR548K | 1.62734 | 912.553 | 36 | 20.7815 |
| 1 | 19 | 58374101 | 58639446 | ZNF587,ZNF814,ZNF417,ZNF418,ZNF256,C19orf18,ZNF606,ZNF606,LOC100128398,LOC100128398,ZSCAN1,ZNF135,ZSCAN18,ZNF329 | 1.44295 | 904.772 | 108 | 65.5683 |

| | | | | | | | | |
|---|---|---|---|---|---|---|---|---|
| 1 | 19 | 54974578 | 55686374 | LENG9,CDC42EP5,LAIR2,KIR3DX1,LILRA2,LILRA1,LILRB1,LILRB4,KIR3DL3,KIR2DL3,LOC101928804,KIR2DL1,KIR2DL1,KIR2DL4,KIR3DL1,KIR2DS4,KIR3DL2,FCAR,NCR1,NLRP7,NLRP2,GP6,RDH13,EPS8L1,PPP1R12C,TNNT1,TNNI3,DNAAF3,SYT5 | 1.37698 | 766.518 | 277 | 155.928 |
| 1 | 10 | 72973426 | 73269275 | UNC5B,SLC29A3,CDH23 | 1.37582 | 732.757 | 35 | 21.0572 |
| 1 | 19 | 56218948 | 57832303 | NLRP9,RFPL4A,RFPL4AL1,NLRP11,NLRP4,NLRP13,NLRP8,NLRP5,ZNF787,ZNF444,GALP,ZSCAN5B,ZSCAN5C,ZSCAN5A,ZNF542P,ZNF582,ZNF583,ZNF667,ZNF667-AS1,ZNF471,ZFP28,ZNF470,ZNF71,SMIM17,ZNF835,ZIM2-AS1,ZIM2,ZIM2,PEG3,PEG3-AS1,ZIM2,PEG3,USP29,ZIM3,DUXA,ZNF264,AURKC,ZNF805,ZNF460,ZNF543 | 1.36647 | 819.826 | 370 | 227.256 |
| 1 | 10 | 76075067 | 76910850 | ADK,ADK,LOC102723439,KAT6B,DUPD1,DUSP13,SAMD8 | 1.35302 | 839.918 | 64 | 38.882 |
| 1 | 10 | 61412499 | 61828678 | SLC16A9,CCDC6,LINC01553,ANK3 | 1.31364 | 729.01 | 39 | 24.1765 |
| 1 | 19 | 50434068 | 50971077 | ATF5,ATF5,MIR4751,SIGLEC11,SIGLEC16,VRK3,ZNF473,LOC400710,IZUMO2,MYH14,KCNC3,NAPSB,NAPSA,NR1H2,POLD1,SPIB,MYBPC2,FAM71E1 | 1.30904 | 747.276 | 202 | 124.127 |
| 1 | 20 | 59830508 | 61040588 | CDH4,CDH4,LOC100128310,MIR1257,TAF4,LSM14B,PSMA7,SS18L1,MTG2,HRH3,OSBPL2,ADRM1,LAMA5,LAMA5,MIR4758,LAMA5,LAMA5-AS1,RPS21,CABLES2,RBBP8NL,GATA5 | 1.22795 | 674.596 | 240 | 146.165 |
| 1 | 10 | 61840241 | 63519234 | ANK3,CDK1,RHOBTB1,TMEM26,TMEM26,TMEM26-AS1,CABCOCO1 | 1.21915 | 701.053 | 101 | 61.7376 |
| 1 | 10 | 1.23E+08 | 123549200 | FGFR2,ATE1 | 1.17383 | 528.534 | 39 | 22.6083 |
| 1 | 1 | 1.51E+08 | 151263693 | LOC100507670,ZNF687,ZNF687 | 1.16338 | 652.333 | 20 | 14.0234 |
| 1 | 1 | 1.61E+08 | 161643044 | HSPA6,FCGR3A,FCGR2C,HSPA7,FCGR3B,FCGR2B | 1.11982 | 644.98 | 49 | 25.5361 |
| 1 | 7 | 1.44E+08 | 144072748 | LOC101928605,OR2A1-AS1,OR2A1,OR2A42,LOC101928605,OR2A1-AS1,OR2A9P,OR2A20P,LOC101928605,OR2A1-AS1,OR2A7,LOC101928605,OR2A1-AS1,OR2A7,ARHGEF34P,LOC101928605,OR2A1-AS1,ARHGEF34P,OR2A1-AS1,OR2A20P,OR2A9P,OR2A1-AS1,OR2A1,OR2A42,ARHGEF5 | 1.05622 | 839.32 | 68 | 29.9706 |
| 1 | 10 | 1.24E+08 | 124189427 | PLEKHA1,PLEKHA1,MIR3941 | 0.954815 | 476.341 | 15 | 8.87181 |
| 1 | 20 | 44468941 | 44643184 | SNX21,SNX21,ACOT8,ACOT8,ZSWIM3,ZSWIM1,SPATA25,NEURL2,NEURL2,CTSA,CTSA,PLTP,PCIF1,ZNF335,MMP9,MMP9,SLC12A5-AS1 | 0.941746 | 556.986 | 114 | 78.1929 |
| 1 | 20 | 39752343 | 40052321 | PLCG1-AS1,PLCG1,PLCG1,ZHX3,LPIN3,EMILIN3,CHD6 | 0.925334 | 544.418 | 95 | 63.827 |
| 1 | 10 | 78977108 | 79814443 | KCNMA1,DLG5,DLG5-AS1,POLR3A,RPS24 | 0.922044 | 556.333 | 105 | 63.267 |
| 1 | 13 | 26108294 | 26933840 | ATP8A2,SHISA2,RNF6,CDK8 | 0.918697 | 529.541 | 79 | 45.9245 |
| 1 | 5 | 66458893 | 66480417 | MAST4,CD180 | 0.915104 | 528.83 | 22 | 16.0649 |
| 1 | 16 | 2811496 | 2820900 | SRRM2 | 0.878739 | 606.538 | 32 | 22.3591 |
| 1 | 10 | 86131221 | 88196728 | CCSER2,GRID1-AS1,GRID1,GRID1,GRID1,MIR346 | 0.870984 | 442.327 | 60 | 36.3087 |
| 1 | 2 | 2.28E+08 | 227663418 | IRS1 | 0.865521 | 491.889 | 14 | 10.522 |
| 1 | 12 | 7032551 | 7080297 | ATN1,C12orf57,PTPN6,MIR200CHG,MIR200C,MIR200CHG,MIR141,PHB2,PHB2,SCARNA12,EMG1 | 0.851998 | 532.738 | 53 | 34.2571 |
| 1 | 10 | 82192281 | 82558150 | TSPAN14,SH2D4B | 0.82169 | 390.042 | 30 | 17.0448 |
| 1 | 10 | 81107355 | 81521061 | PPIF,ZCCHC24,LOC729815,EIF5AL1,SFTPA2,SFTPA1 | 0.78866 | 482.874 | 49 | 26.1312 |

| | | | | | | | | |
|---|---|---|---|---|---|---|---|---|
| 1 | 10 | 74776504 | 75521934 | P4HA1,NUDT13,ECD,FAM149B1,DNAJC9,DNAJC9,DNAJC9-AS1,DNAJC9-AS1,DNAJC9-AS1,MRPS16,DNAJC9-AS1,CFAP70,CFAP70,ANXA7,MSS51,PPP3CB,PPP3CB,PPP3CB-AS1,PPP3CB-AS1,USP54,USP54,MYOZ1,SYNPO2L,GLUD1P3,SEC24C | 0.778779 | 494.073 | 212 | 125.934 |
| 1 | 11 | 70788658 | 70858431 | SHANK2 | 0.770369 | 668.295 | 17 | 10.1975 |
| 1 | 1 | 1.55E+08 | 155296977 | ADAR,KCNN3,PMVK,PBXIP1,PYGO2,PYGO2,LOC101928120,SHC1,CKS1B,CKS1B,MIR4258,FLAD1,LENEP,ZBTB7B,ZBTB7B,DCST2,DCST2,DCST1,DCST1,DCST1-AS1,DCST1-AS1,ADAM15,DCST1-AS1,EFNA4,EFNA4,EFNA3,EFNA1,SLC50A1,DPM3,KRTCAP2,TRIM46,MUC1,MIR92B,THBS3,MTX1,MTX1,GBAP1,GBAP1,GBA,FAM189B,SCAMP3,CLK2,HCN3,PKLR,FDPS,FDPS,RUSC1-AS1,RUSC1-AS1,RUSC1,RUSC1 | 0.767753 | 482.147 | 346 | 221.786 |
| 1 | 12 | 49419911 | 49448558 | KMT2D | 0.758638 | 444.844 | 79 | 53.5986 |
| 1 | 20 | 61040771 | 62959313 | GATA5,MIR1-1HG-AS1,MIR1-1HG,MIR1-1HG,MIR1-1,MIR1-1HG,MIR133A2,SLCO4A1,SLCO4A1,SLCO4A1-AS1,NTSR1,MRGBP,OGFR-AS1,OGFR,OGFR,COL9A3,TCFL5,TCFL5,DPH3P1,DIDO1,GID8,SLC17A9,BHLHE23,LINC01749,MIR124-3,YTHDF1,BIRC7,BIRC7,MIR3196,NKAIN4,NKAIN4,FLJ16779,ARFGAP1,ARFGAP1,MIR4326,COL20A1,CHRNA4,CHRNA4,LOC100130587,KCNQ2,EEF1A2,PPDPF,PTK6,SRMS,FNDC11,HELZ2,GMEB2,STMN3,RTEL1,RTEL1-TNFRSF6B,RTEL1-TNFRSF6B,TNFRSF6B,ARFRP1,ZGPAT,ZGPAT,LIME1,LIME1,SLC2A4RG,ZBTB46,ABHD16B,TPD52L2,DNAJC5,UCKL1,UCKL1,MIR1914,UCKL1,MIR647,UCKL1,UCKL1-AS1,ZNF512B,SAMD10,PRPF6,C20orf204,SOX18,TCEA2,RGS19,RGS19,MIR6813,OPRL1,LKAAEAR1,OPRL1,NPBWR2,MYT1,PCMTD2 | 0.715444 | 496.762 | 614 | 380.374 |
| 1 | 20 | 33328142 | 34288865 | NCOA6,GGT7,ACSS2,GSS,MYH7B,MYH7B,MIR499A,MIR499B,TRPC4AP,EDEM2,MMP24-AS1-EDEM2,MMP24-AS1-EDEM2,MMP24-AS1-EDEM2,PROCR,MMP24-AS1-EDEM2,MMP24,EIF6,FAM83C,UQCC1,GDF5OS,GDF5OS,GDF5,GDF5,GDF5,MIR1289-1,CEP250,C20orf173,ERGIC3,FER1L4,SPAG4,CPNE1,CPNE1,RBM12,NFS1,NFS1,ROMO1,ROMO1 | 0.714737 | 456.342 | 375 | 233.84 |
| 1 | 20 | 29890959 | 31291344 | DEFB116,DEFB118,DEFB119,DEFB121,DEFB123,DEFB124,REM1,HM13,HM13,HM13-AS1,ID1,MIR3193,COX4I2,BCL2L1,BCL2L1,ABALON,TPX2,MYLK2,FOXS1,DUSP15,DUSP15,TTLL9,TTLL9,PDRG1,XKR7,CCM2L,HCK,TM9SF4,TSPY26P,PLAGL2,POFUT1,POFUT1,MIR1825,KIF3B,ASXL1,NOL4L,NOL4L,LOC101929698,NOL4L-DT,C20orf203,COMMD7 | 0.693332 | 444.698 | 289 | 178.151 |
| 1 | 20 | 54972217 | 55905066 | CSTF1,CASS4,RTFDC1,RTFDC1,GCNT7,GCNT7,FAM209A,FAM209B,TFAP2C,BMP7,MIR4325,SPO11 | 0.689346 | 459.526 | 69 | 43.7206 |
| 1 | 3 | 39184694 | 39322479 | CSRNP1,XIRP1,CX3CR1 | 0.670266 | 354.737 | 37 | 25.511 |

| | | | | | | | | |
|---|---|---|---|---|---|---|---|---|
| 1 | 20 | 47733581 | 52824694 | STAU1,DDX27,ZNFX1,ZFAS1,SNORD12C,ZFAS1,SNORD12B,ZFAS1,SNORD12,KCNB1,PTGIS,B4GALT5,SLC9A8,SPATA2,RNF114,SNAI1,UBE2V1,TMEM189-UBE2V1,TMEM189-UBE2V1,TMEM189,CEBPB-AS1,CEBPB,SMIM25,PTPN1,MIR645,RIPOR3,RIPOR3,MIR1302-5,PARD6B,BCAS4,ADNP,ADNP-AS1,DPM1,DPM1,MOCS3,KCNG1,NFATC2,NFATC2,MIR3194,ATP9A,SALL4,ZFP64,TSHZ2,LOC101927770,ZNF217,ZNF217,BCAS1,BCAS1,MIR4756,CYP24A1,PFDN4 | 0.669271 | 465.183 | 478 | 292.956 |
| 1 | 13 | 32365866 | 33284254 | RXFP2,FRY,ZAR1L,BRCA2,N4BP2L1,N4BP2L2,PDS5B | 0.667494 | 528.123 | 191 | 119.891 |
| 1 | 5 | 82815127 | 82876268 | VCAN,VCAN,VCAN-AS1 | 0.63989 | 449.262 | 42 | 29.4987 |
| 1 | 10 | 67829086 | 68688268 | CTNNA3,CTNNA3,LOC101928961,LRRTM3 | 0.632163 | 488.473 | 25 | 16.2202 |
| 1 | 16 | 711380 | 779712 | WDR90,RHOT2,RHBDL1,STUB1,STUB1,JMJD8,JMJD8,WDR24,FBXL16,METRN,FAM173A,FAM173A,CCDC78,CCDC78,HAGHL | 0.627325 | 492.381 | 106 | 71.6497 |
| 1 | 16 | 72821224 | 73126340 | ZFHX3 | 0.619104 | 367.135 | 47 | 32.409 |
| 1 | 20 | 44644803 | 47675165 | MMP9,SLC12A5-AS1,SLC12A5-AS1,SLC12A5,SLC12A5,NCOA5,CD40,CDH22,SLC35C2,ELMO2,ZNF334,OCSTAMP,SLC13A3,TP53RK,SLC2A10,EYA2,EYA2,MIR3616,ZMYND8,ZMYND8,LOC100131496,NCOA3,SULF2,PREX1,ARFGEF2,CSE1L | 0.617858 | 428.524 | 370 | 228.731 |
| 1 | 1 | 1.61E+08 | 161487984 | ARHGAP30,NECTIN4,KLHDC9,PFDN2,NIT1,NIT1,DEDD,DEDD,UFC1,USP21,PPOX,B4GALT3,ADAMTS4,NDUFS2,FCER1G,APOA2,TOMM40L,TOMM40L,MIR5187,TOMM40L,NR1I3,NR1I3,PCP4L1,MPZ,SDHC,CFAP126,FCGR2A | 0.612494 | 459.36 | 181 | 113.595 |
| 1 | 1 | 1.59E+08 | 160106160 | ACKR1,FCER1A,OR10J3,OR10J4,OR10J1,OR10J5,APCS,CRP,DUSP23,FCRL6,SLAMF8,SLAMF8,SNHG28,SNHG28,SNHG28,VSIG8,VSIG8,CFAP45,CFAP45,MIR4259,TAGLN2,IGSF9,SLAMF9,LINC01133,PIGM,KCNJ10,KCNJ9,IGSF8,ATP1A2 | 0.606346 | 450.929 | 180 | 113.707 |
| 1 | 8 | 77616293 | 77776514 | ZFHX4 | 0.589454 | 490.777 | 48 | 34.4926 |
| 1 | 20 | 55919010 | 58422336 | RAE1,LOC100291105,RBM38,RBM38,CTCFL,PCK1,ZBP1,PMEPA1,PMEPA1,NKILA,MIR4532,C20orf85,ANKRD60,PPP4R1L,RAB22A,VAPB,APCDD1L,LINC01711,STX16,STX16-NPEPL1,STX16-NPEPL1,NPEPL1,MIR296,MIR298,GNAS-AS1,GNAS,GNAS,NELFCD,CTSZ,TUBB1,ATP5F1E,SLMO2-ATP5E,SLMO2-ATP5E,PRELID3B,ZNF831,EDN3,PHACTR3 | 0.584679 | 431.227 | 310 | 183.6 |
| 1 | 1 | 2.28E+08 | 229385621 | SNAP47,JMJD4,SNAP47,PRSS38,WNT9A,WNT9A,MIR5008,WNT3A,ARF1,MIR3620,ARF1,C1orf35,MRPL55,GUK1,GJC2,IBA57-AS1,IBA57,OBSCN-AS1,OBSCN-AS1,OBSCN,OBSCN,TRIM11,TRIM11,MIR6742,TRIM17,HIST3H3,HIST3H2A,HIST3H2BB,MIR4666A,RNF187,BTNL10,RHOU,DUSP5P1,RHOU | 0.583646 | 460.33 | 296 | 186.128 |
| 1 | 4 | 47033873 | 47939555 | GABRB1,COMMD8,ATP10D,CORIN,CORIN,LOC101927179,LOC101927179,NFXL1,NFXL1,NFXL1,LOC101927157,LOC101927157,CNGA1 | 0.57621 | 487.288 | 102 | 63.0973 |
| 1 | 12 | 9446447 | 9632653 | LOC642846,LINC02367,LOC728715,LOC728715,DDX12P | 0.576049 | 428.749 | 55 | 25.8405 |

| | | | | | | | | |
|---|---|---|---|---|---|---|---|---|
| 1 | 1 | 2.47E+08 | 249240121 | ZNF669,C1orf229,ZNF124,MIR3916,VN1R5,ZNF496,NLRP3,OR2B11,OR2W5,GCSAML,GCSAML-AS1,GCSAML,OR2C3,GCSAML,OR2G2,OR2G3,OR13G1,OR6F1,OR14A2,OR14A2,OR14K1,OR1C1,OR14A16,OR11L1,TRIM58,OR2W3,OR2T8,OR2AJ1,OR2L13,OR2L8,OR2L13,OR2AK2,OR2L13,OR2L1P,OR2L13,OR2L13,OR2L5,OR2L13,OR2L2,OR2L13,OR2L3,OR2M1P,OR2M5,OR2M2,OR2M3,OR2M4,OR2T33,OR2T12,OR2M7,OR14C36,OR2T4,OR2T6,OR2T1,OR2T7,OR2T2,OR2T3,OR2T5,OR2G6,OR2T29,OR2T34,OR2T10,OR2T11,OR2T35,OR2T27,OR14I1,LYPD8,SH3BP5L,MIR3124,ZNF672,ZNF692,PGBD2 | 0.573688 | 533.109 | 330 | 190.292 |
| 1 | 20 | 34309627 | 39751843 | RBM39,PHF20,SCAND1,CNBD2,NORAD,EPB41L1,EPB41L1,LOC100130373,AAR2,DLGAP4,DLGAP4,DLGAP4-AS1,DLGAP4-AS1,MYL9,TGIF2,TGIF2-C20orf24,TGIF2-C20orf24,C20orf24,SLA2,NDRG3,DSN1,SOGA1,TLDC2,TLDC2,SAMHD1,SAMHD1,RBL1,MROH8,MROH8,RPN2,RPN2,GHRH,MANBAL,SRC,BLCAP,BLCAP,NNAT,CTNNBL1,VSTM2L,TTI1,RPRD1B,TGM2,KIAA1755,KIAA1755,LOC149684,BPI,LBP,SNHG17,SNORA71B,SNHG17,SNORA71A,SNHG17,SNORA71C,SNHG17,SNORA71D,SNHG11,SNORA71E,SNHG11,SNORA60,RALGAPB,RALGAPB,MIR548O2,ADIG,ARHGAP40,SLC32A1,ACTR5,PPP1R16B,FAM83D,DHX35,MAFB,LOC100128988,TOP1,TOP1,PLCG1-AS1 | 0.569341 | 425.92 | 669 | 410.305 |
| 1 | 1 | 1.55E+08 | 156900414 | RUSC1,ASH1L,ASH1L,MIR555,ASH1L,POU5F1P4,ASH1L,ASH1L-AS1,ASH1L-AS1,MSTO1,MSTO2P,YY1AP1,DAP3,MSTO2P,MSTO2P,GON4L,GON4L,SYT11,RIT1,KHDC4,KHDC4,SNORA80E,KHDC4,SCARNA4,RXFP4,ARHGEF2,ARHGEF2,MIR6738,SSR2,UBQLN4,LAMTOR2,RAB25,MEX3A,LMNA,SEMA4A,SLC25A44,PMF1,PMF1-BGLAP,PMF1-BGLAP,BGLAP,PAQR6,SMG5,SMG5,TMEM79,GLMP,VHLL,CCT3,CCT3,TSACC,TSACC,RHBG,C1orf61,C1orf61,MIR9-1,MEF2D,IQGAP3,TTC24,NAXE,GPATCH4,HAPLN2,BCAN,NES,CRABP2,ISG20L2,RRNAD1,MRPL24,HDGF,PRCC,SH2D2A,SH2D2A,NTRK1,NTRK1,INSRR,NTRK1,PEAR1,LRRC71 | 0.565376 | 446.951 | 695 | 430.245 |

| | | | | | | | | |
|---|---|---|---|---|---|---|---|---|
| 1 | 20 | 40053797 | 44467047 | CHD6,PTPRT,SRSF6,L3MBTL1,SGK2,IFT52,MYBL2,GTSF1L,TOX2,JPH2,OSER1,GDAP1L1,FITM2,R3HDML,HNF4A,HNF4A,HNF4A-AS1,HNF4A,MIR3646,LINC01620,TTPAL,SERINC3,PKIG,ADA,KCNK15-AS1,WISP2,KCNK15-AS1,KCNK15,KCNK15,RIMS4,YWHAB,PABPC1L,PABPC1L,TOMM34,TOMM34,STK4,KCNS1,WFDC5,WFDC12,PI3,SEMG1,SEMG2,SLPI,MATN4,MATN4,RBPJL,RBPJL,SDC4,SYS1,SYS1-DBNDD2,SYS1,SYS1-DBNDD2,TP53TG5,SYS1-DBNDD2,TP53TG5,SYS1-DBNDD2,DBNDD2,PIGT,PIGT,MIR6812,WFDC2,SPINT3,WFDC6,WFDC6,EPPIN-WFDC6,EPPIN-WFDC6,EPPIN,WFDC8,WFDC9,WFDC9,WFDC10A,WFDC11,WFDC10B,WFDC10B,WFDC13,WFDC10B,WFDC13,MIR3617,WFDC13,SPINT4,WFDC3,WFDC3,DNTTIP1,DNTTIP1,UBE2C,TNNC2,SNX21 | 0.564381 | 416.646 | 553 | 335.114 |
| 1 | 10 | 77795675 | 78317088 | LRMDA | 0.562072 | 341.008 | 13 | 6.6867 |
| 1 | 8 | 1.45E+08 | 146303522 | SCRIB,MIR937,SCRIB,PUF60,NRBP2,NRBP2,MIR6845,EPPK1,PLEC,PLEC,MIR661,PARP10,GRINA,SPATC1,OPLAH,OPLAH,MIR6846,EXOSC4,EXOSC4,MIR6847,GPAA1,CYC1,SHARPIN,MAF1,WDR97,HGH1,MROH1,MIR7112,SCX,BOP1,BOP1,MIR7112,BOP1,SCX,HSF1,DGAT1,DGAT1,MIR6848,SCRT1,TMEM249,TMEM249,FBXL6,FBXL6,SLC52A2,ADCK5,CPSF1,CPSF1,MIR939,CPSF1,MIR6849,SLC39A4,VPS28,TONSL,TONSL,TONSL-AS1,MIR6893,TONSL,TONSL-AS1,CYHR1,KIFC2,FOXH1,PPP1R16A,GPT,MFSD3,RECQL4,LRRC14,LRRC14,LRRC24,C8orf82,ARHGAP39,ZNF251,ZNF34,RPL8,RPL8,MIR6850,ZNF517,ZNF517,LOC100130027,ZNF7,COMMD5,ZNF250,ZNF16,ZNF252P,ZNF252P,TMED10P1,ZNF252P-AS1,C8orf33 | 0.551675 | 387.293 | 735 | 465.504 |
| 1 | 12 | 1.22E+08 | 122285126 | KDM2B,KDM2B,MIR7107,ORAI1,MORN3,TMEM120B,TMEM120B,RHOF,RHOF,LINC01089,SETD1B,HPD | 0.550248 | 450.236 | 104 | 64.0247 |
| 1 | 12 | 60500 | 4609494 | IQSEC3,IQSEC3,LOC574538,SLC6A12,SLC6A12,LOC101929384,SLC6A13,KDM5A,CCDC77,B4GALNT3,NINJ2,NINJ2,LOC100049716,WNK1,RAD52,ERC1,FBXL14,WNT5B,MIR3649,ADIPOR2,CACNA2D4,CACNA2D4,LRTM2,LINC00940,DCP1B,CACNA1C,CACNA1C,CACNA1C-AS2,CACNA1C,CACNA1C-AS1,ITFG2-AS1,ITFG2-AS1,FKBP4,ITFG2-AS1,ITFG2,ITFG2,ITFG2,NRIP2,ITFG2,FOXM1,FOXM1,RHNO1,TULP3,TEAD4,TSPAN9,LOC100128253,PRMT8,PRMT8,THCAT155,CRACR2A,PARP11,CCND2-AS1,CCND2,CCND2,TIGAR,FGF23,FGF6,C12orf4 | 0.536934 | 412.572 | 640 | 386.784 |
| 1 | 19 | 42381287 | 42931295 | CD79A,ARHGEF1,LOC100505585,RABAC1,ATP1A3,GRIK5,ZNF574,POU2F2,LOC100505622,MIR4323,DEDD2,ZNF526,GSK3A,ERF,CIC,PAFAH1B3,PAFAH1B3,PRR19,PRR19,TMEM145,MEGF8,MEGF8,MIR8077,CNFN,LOC101930071,LIPE-AS1,LIPE,LIPE-AS1,LIPE | 0.522353 | 420.74 | 277 | 176.95 |

| | | | | | | | | |
|---|---|---|---|---|---|---|---|---|
| 1 | 16 | 66885348 | 68344972 | CA7,PDP2,CDH16,RRAD,FAM96B,CES2,CES3,CES4A,CBFB,C16orf70,B3GNT9,TRADD,FBXL8,FBXL8,HSF4,HSF4,NOL3,KIAA0895L,EXOC3L1,E2F4,ELMO3,ELMO3,MIR328,LRRC29,LRRC29,TMEM208,TMEM208,FHOD1,SLC9A5,PLEKHG4,PLEKHG4,KCTD19,KCTD19,LRRC36,TPPP3,ZDHHC1,HSD11B2,ATP6V0D1,AGRP,RIPOR1,CTCF,CARMIL2,CARMIL2,ACD,ACD,PARD6A,ENKD1,C16orf86,GFOD2,RANBP10,TSNAXIP1,CENPT,CENPT,THAP11,NUTF2,EDC4,NRN1L,PSKH1,CTRL,PSMB10,LCAT,LCAT,SLC12A4,SLC12A4,DPEP3,DPEP2,DDX28,DDX28,DUS2,DUS2,NFATC3,ESRP2,ESRP2,MIR6773,PLA2G15,SLC7A6,SLC7A6,SLC7A6OS,SLC7A6OS,SLC7A6OS,PRMT7 | 0.519873 | 433.407 | 690 | 452.505 |
| 1 | 1 | 1.1E+08 | 109816179 | CELSR2 | 0.519498 | 521.08 | 47 | 32.3239 |
| 1 | 4 | 1.24E+08 | 126412947 | SPATA5,SPRY1,ANKRD50,FAT4 | 0.50729 | 480.467 | 107 | 72.6865 |
| 1 | 1 | 1.43E+08 | 150667365 | NBPF20,NBPF19,NBPF8,NBPF9,NBPF20,NBPF19,NBPF9,LOC100996724,PDE4DIP,NBPF20,NBPF19,NBPF9,PDE4DIP,NBPF20,NBPF19,NBPF9,SEC22B,NBPF20,NBPF19,NBPF9,NBPF20,NBPF19,NBPF9,NOTCH2NL,NBPF20,NBPF19,NBPF10,HFE2,NBPF20,NBPF19,NBPF10,TXNIP,NBPF20,NBPF19,NBPF10,POLR3GL,NBPF20,NBPF19,NBPF10,ANKRD34A,NBPF20,NBPF19,NBPF10,LIX1L,NBPF20,NBPF19,NBPF10,LIX1L,LIX1L-AS1,NBPF20,NBPF19,NBPF10,LIX1L-AS1,RBM8A,NBPF20,NBPF19,NBPF10,RBM8A,NBPF20,NBPF19,NBPF10,RBM8A,GNRHR2,NBPF20,NBPF19,NBPF10,GNRHR2,NBPF20,NBPF19,NBPF10,PEX11B,NBPF20,NBPF19,NBPF10,ITGA10,NBPF20,NBPF19,NBPF10,ANKRD35,NBPF20,NBPF19,NBPF10,PIAS3,NBPF20,NBPF19,NBPF10,PIAS3,MIR6736,NBPF20,NBPF19,NBPF10,PIAS3,NUDT17,NBPF20,NBPF19,NBPF10,NUDT17,NBPF20,NBPF19,NBPF10,POLR3C,NBPF20,NBPF19,NBPF10,RNF115,NBPF20,NBPF19,NBPF10,CD160,NBPF20,NBPF19,NBPF10,PDZK1,NBPF20,NBPF19,NBPF10,GPR89A,NBPF20,NBPF19,NBPF10,NBPF25P,NBPF20,NBPF19,NBPF10,NBPF25P,PDE4DIPP1,NBPF19,LOC728989,NBPF19,PRKAB2,NBPF19,CHD1L,PDIA3P1,NBPF19,CHD1L,FMO5,NBPF19,CHD1L,NBPF19,BCL9,NBPF19,ACP6,NBPF19,GJA5,NBPF19,GJA8,NBPF19,GPR89B,NBPF19,PDE4DIPP1,NBPF19,MIR5087,NBPF25P,FAM231D,LOC388692,LOC100996741,FCGR1CP,HIST2H3PS2,LOC101929798,HIST2H2BF,FCGR1A,HIST2H2BF,HIST2H4A,HIST2H4B,HIST2H3C,HIST2H3A,HIST2H2AA3,HIST2H2AA4,HIST2H2BC,HIST2H2BE,HIST2H2AC,HIST2H2AB,BOLA1,SV2A,SF3B4,MTMR11,OTUD7B,VPS45,PLEKHO1,ANP32E,CA14,APH1A,C1orf54,CIART,MRPS21,PRPF3,RPRD2,TARS2,TARS2,MIR6878,ECM1,ADAMTSL4,ADAMTSL4,MIR4257,ADAMTSL4-AS1,MCL1,ENSA,GOLPH3L | 0.50569 | 446.443 | 730 | 425.263 |

| | | | | | | | | |
|---|---|---|---|---|---|---|---|---|
| 1 | 16 | 28332751 | 31510777 | EIF3CL,EIF3C,EIF3C,CLN3,APOBR,IL27,NUPR1,SGF29,SULT1A2,SULT1A1,EIF3C,EIF3CL,ATXN2L,TUFM,TUFM,MIR4721,SH2B1,ATP2A1,ATP2A1,ATP2A1-AS1,ATP2A1,RABEP2,RABEP2,CD19,NFATC2IP,NFATC2IP,MIR4517,SPNS1,LAT,RRN3P2,SNX29P2,BOLA2-SMG1P6,LOC606724,BOLA2-SMG1P6,BOLA2,BOLA2B,BOLA2-SMG1P6,BOLA2B,BOLA2,SLX1B,SLX1A,SLX1B,SLX1A,SLX1A-SULT1A3,SLX1B-SULT1A4,SLX1A-SULT1A3,SLX1B-SULT1A4,SLX1A-SULT1A3,SLX1B-SULT1A4,SULT1A3,SULT1A4,LOC388242,LOC613038,SMG1P2,SMG1P2,MIR3680-2,MIR3680-1,SPN,QPRT,C16orf54,ZG16,KIF22,MAZ,PRRT2,PAGR1,MVP,CDIPT,CDIPT-AS1,SEZ6L2,ASPHD1,KCTD13,TMEM219,TAOK2,HIRIP3,INO80E,DOC2A,C16orf92,C16orf92,FAM57B,FAM57B,ALDOA,PPP4C,TBX6,YPEL3,LOC101928595,GDPD3,GDPD3,MAPK3,CORO1A,CORO1A,LOC606724,BOLA2,BOLA2B,BOLA2B,BOLA2,SLX1B,SLX1A,CD2BP2,TBC1D10B,MYLPF,SEPT1,SEPT1,ZNF48,ZNF48,ZNF771,DCTPP1,SEPHS2,ITGAL,ITGAL,MIR4518,ZNF768,ZNF747,ZNF764,ZNF688,ZNF785,ZNF689,PRR14,FBRS,SRCAP,SRCAP,SNORA30,PHKG2,PHKG2,CCDC189,CCDC189,RNF40,RNF40,ZNF629,BCL7C,BCL7C,MIR4519,BCL7C,MIR762HG,BCL7C,MIR762HG,MIR762,CTF1,FBXL19-AS1,FBXL19,FBXL19,ORAI3,SETD1A,HSD3B7,STX1B,STX4,ZNF668,ZNF646,PRSS53,VKORC1,BCKDK,KAT8,PRSS8,PRSS36,FUS,PYCARD,PYCARD,PYCARD-AS1,TRIM72,TRIM72,PYDC1,ITGAM,ITGAX,ITGAD,COX6A2,ZNF843,ARMC5,TGFB1I1,SLC5A2,SLC5A2,C16orf58,C16orf58 | 0.504401 | 426.541 | 1214 | 755.825 |
| 1 | 12 | 47181652 | 48056723 | SLC38A4,AMIGO2,PCED1B,MIR4698,PCED1B,MIR4494 | 0.497995 | 446.505 | 24 | 15.8179 |
| 1 | 3 | 49020247 | 49226806 | ARIH2,P4HTM,WDR6,WDR6,DALRD3,DALRD3,DALRD3,MIR425,DALRD3,NDUFAF3,MIR191,NDUFAF3,IMPDH2,QRICH1,QARS,QARS,MIR6890,USP19,LAMB2,CCDC71,KLHDC8B,C3orf84 | 0.495346 | 407.508 | 166 | 114.919 |

| | | | | | | | | |
|---|---|---|---|---|---|---|---|---|
| 1 | 1 | 1.51E+08 | 154571089 | PI4KB,RFX5,SELENBP1,PSMB4,POGZ,CGN,TUFT1,TUFT1,MIR554,SNX27,CELF3,RIIAD1,MRPL9,MRPL9,OAZ3,OAZ3,TDRKH,LINGO4,RORC,C2CD4D,C2CD4D,LOC100132111,THEM5,THEM4,S100A10,S100A11,TCHHL1,TCHH,RPTN,HRNR,FLG,FLG,FLG-AS1,FLG-AS1,FLG2,CRNN,LCE5A,CRCT1,LCE3E,LCE3D,LCE3A,LCE2D,LCE2C,LCE2B,LCE2A,LCE4A,C1orf68,KPRP,LCE1F,LCE1E,LCE1C,LCE1B,LCE1A,LCE6A,SMCP,IVL,SPRR4,SPRR1A,SPRR3,SPRR1B,SPRR2C,SPRR2G,LELP1,PRR9,LOR,PGLYRP3,PGLYRP4,S100A9,S100A12,S100A8,S100A8,S100A7A,S100A7L2,S100A7,S100A6,S100A5,S100A4,LOC101928034,S100A3,S100A2,S100A16,S100A14,S100A13,S100A13,S100A1,CHTOP,SNAPIN,ILF2,NPR1,INTS3,SLC27A3,GATAD2B,DENND4B,CRTC2,SLC39A1,SLC39A1,MIR6737,CREB3L4,JTB,RAB13,RPS27,NUP210L,NUP210L,MIR5698,TPM3,MIR190B,C1orf189,C1orf43,C1orf43,UBAP2L,UBAP2L,HAX1,AQP10,ATP8B2,LOC101928101,IL6R,IL6R,SHE,TDRD10,UBE2Q1,UBE2Q1,UBE2Q1-AS1,CHRNB2,ADAR | 0.485025 | 450.084 | 934 | 557.169 |
| 1 | 20 | 31291619 | 33326863 | COMMD7,DNMT3B,MAPRE1,EFCAB8,SUN5,BPIFB2,BPIFB6,BPIFB3,BPIFB4,BPIFA2,BPIFA3,BPIFA1,BPIFB1,CDK5RAP1,SNTA1,CBFA2T2,NECAB3,NECAB3,C20orf144,NECAB3,ACTL10,E2F1,PXMP4,ZNF341,ZNF341,ZNF341-AS1,CHMP4B,RALY,MIR4755,RALY,EIF2S2,ASIP,AHCY,ITCH,ITCH,MIR644A,DYNLRB1,MAP1LC3A,PIGU,TP53INP2,NCOA6 | 0.480738 | 418.996 | 416 | 248.894 |
| 1 | 9 | 33290480 | 33528853 | NFX1,AQP7,AQP3,NOL6,ANKRD18B | 0.479011 | 374.5 | 74 | 45.9625 |
| 1 | 10 | 1.17E+08 | 117823375 | ATRNL1 | 0.47779 | 229.59 | 14 | 7.006 |
| 1 | 12 | 4667962 | 7032051 | RAD51AP1,DYRK4,AKAP3,AKAP3,NDUFA9,NDUFA9,GALNT8,KCNA6,KCNA1,KCNA5,NTF3,ANO2,VWF,CD9,PLEKHG6,TNFRSF1A,SCNN1A,SCNN1A,LTBR,LTBR,CD27-AS1,CD27-AS1,CD27,TAPBPL,TAPBPL,VAMP1,VAMP1,MRPL51,NCAPD2,NCAPD2,SCARNA10,GAPDH,IFFO1,NOP2,CHD4,CHD4,SCARNA11,LPAR5,ACRBP,ING4,ZNF384,PIANP,COPS7A,MLF2,PTMS,LAG3,CD4,GPR162,P3H3,GNB3,GNB3,CDCA3,CDCA3,USP5,TPI1,SPSB2,LRRC23,ENO2 | 0.461386 | 392.483 | 617 | 381.691 |

| | | | | | | | | |
|---|---|---|---|---|---|---|---|---|
| 1 | 1 | 2.01E+08 | 211836862 | CAMSAP2,GPR25,C1orf106,KIF21B,CACNA1S,TMEM9,IGFN1,PKP1,TNNT2,LAD1,TNNI1,PHLDA3,CSRP1,NAV1,NAV1,IPO9-AS1,NAV1,IPO9-AS1,MIR5191,NAV1,IPO9-AS1,MIR1231,IPO9-AS1,IPO9,IPO9,SHISA4,LMOD1,TIMM17A,RNPEP,RNPEP,ELF3-AS1,ELF3,GPR37L1,ARL8A,PTPN7,PTPRVP,LGR6,UBE2T,PPP1R12B,SYT2,KDM5B,MGAT4EP,RABIF,KLHL12,ADIPOR1,CYB5R1,LOC100506747,TMEM183A,TMEM183B,PPFIA4,MYOG,ADORA1,MYBPH,CHI3L1,CHIT1,BTG2,FMOD,PRELP,OPTC,ATP2B4,ATP2B4,SNORA77,LAX1,ZC3H11A,ZBED6,ZC3H11A,SNRPE,LINC00303,SOX13,ETNK2,ETNK2,ERLNC1,REN,KISS1,GOLT1A,PLEKHA6,PPP1R15B,PIK3C2B,MDM4,LRRN2,NFASC,CNTN2,TMEM81,RBBP5,DSTYK,TMCC2,NUAK2,KLHDC8A,LEMD1-AS1,LEMD1,LEMD1,BLACAT1,MIR135B,CDK18,MFSD4A,ELK4,SLC45A3,NUCKS1,RAB29,SLC41A1,PM20D1,LOC284581,SLC26A9,AVPR1B,RHEX,CTSE,SRGAP2,SRGAP2,SRGAP2D,IKBKE,IKBKE,MIR6769B,RASSF5,EIF2D,DYRK3,MAPKAPK2,IL10,IL19,IL20,IL24,FCMR,PIGR,FCAMR,C1orf116,YOD1,PFKFB2,C4BPB,C4BPA,CD55,CR2,CR1,CR1L,CD46,MIR29B2CHG,MIR29C,MIR29B2CHG,MIR29B2,MIR29B2CHG,MIR29B2CHG,LOC148696,CD34,PLXNA2,MIR205HG,MIR205HG,MIR205,CAMK1G,LAMB3,LAMB3,MIR4260,LOC101930114,G0S2,LOC101930114,HSD11B1,TRAF3IP3,TRAF3IP3,C1orf74,C1orf74,IRF6,DIEXF,SYT14,SERTAD4-AS1,SERTAD4-AS1,SERTAD4,SERTAD4,HHAT,KCNH1,RCOR3,TRAF5,LINC00467,RD3,SLC30A1,NEK2 | 0.459979 | 395.231 | 1841 | 1115.05 |
| 1 | 9 | 1.39E+08 | 140242039 | [illegible] 4,EGFL7,EGFL7,MIR126,AGPAT2,FAM69B,SNHG7,SNORA17B,SNHG7,SNORA17A,LCN10,LCN6,LCN6,LOC100128593,LCN8,LCN15,TMEM141,CCDC183,CCDC183,CCDC183-AS1,CCDC183-AS1,RABL6,RABL6,RABL6,MIR4292,AJM1,PHPT1,MAMDC4,EDF1,TRAF2,TRAF2,MIR4479,FBXW5,C8G,LCN12,PRR31,PTGDS,LCNL1,PAXX,CLIC3,ABCA2,ABCA2,C9orf139,C9orf139,FUT7,C9orf139,NPDC1,ENTPD2,SAPCD2,UAP1L1,MAN1B1-AS1,MAN1B1,DPP7,GRIN1,LRRC26,LRRC26,MIR3621,TMEM210,ANAPC2,SSNA1,TPRN,TMEM203,NDOR1,RNF208,CYSRT1,RNF224,SLC34A3,TUBB4B,TUBB4B,FAM166A,FAM166A,STPG3-AS1,STPG3,NELFB,TOR4A,NRARP,EXD3 | 0.454376 | 406.533 | 539 | 341.988 |

| | | | | | | | | |
|---|---|---|---|---|---|---|---|---|
| 1 | 4 | 71067907 | 83033382 | ODAM,FDCSP,CSN3,CABS1,SMR3A,SMR3B,OPRPN,MUC7,AMTN,AMBN,ENAM,JCHAIN,UTP3,RUFY3,GRSF1,MOB1B,DCK,SLC4A4,GC,NPFFR2,ADAMTS3,COX18,ANKRD17,ALB,AFP,AFM,RASSF6,CXCL8,CXCL6,PF4V1,CXCL1,PF4,PPBP,CXCL5,CXCL3,CXCL2,MTHFD2L,EPGN,EREG,AREG,BTC,PARM1,RCHY1,THAP6,ODAPH,CDKL2,G3BP2,USO1,PPEF2,NAAA,SDAD1,SDAD1,LOC101928809,LOC101928809,CXCL9,CXCL9,ART3,CXCL10,ART3,CXCL11,ART3,NUP54,SCARB2,FAM47E,FAM47E,FAM47E-STBD1,FAM47E-STBD1,FAM47E-STBD1,STBD1,CCDC158,SHROOM3,SHROOM3,MIR4450,SOWAHB,SEPT11,CCNI,CCNG2,CXCL13,CNOT6L,MRPL1,FRAS1,ANXA3,BMP2K,PAQR3,NAA11,GK2,ANTXR2,PRDM8,FGF5,C4orf22,BMP3,PRKG2,PRKG2,LOC101928942,RASGEF1B | 0.448996 | 411.605 | 1146 | 673.139 |
| 1 | 12 | 7083475 | 7344375 | EMG1,EMG1,LPCAT3,LPCAT3,C1S,C1R,C1RL,C1RL,C1RL-AS1,C1RL-AS1,RBP5,CLSTN3,PEX5 | 0.447468 | 400.163 | 100 | 64.8375 |
| 1 | 1 | 1.51E+08 | 151252700 | CTSS,CTSK,ARNT,SETDB1,CERS2,ANXA9,MINDY1,PRUNE1,BNIPL,C1orf56,CDC42SE1,MLLT11,GABPB2,SEMA6C,TNFAIP8L2,TNFAIP8L2-SCNM1,TNFAIP8L2-SCNM1,LYSMD1,TNFAIP8L2-SCNM1,SCNM1,TNFAIP8L2-SCNM1,SCNM1,TMOD4,TMOD4,VPS72,PIP5K1A,PSMD4,LOC100507670 | 0.441 | 396.352 | 238 | 145.34 |
| 1 | 2 | 1.79E+08 | 179500083 | MIR548N,TTN-AS1,TTN,MIR548N,TTN | 0.436298 | 462.261 | 285 | 204.971 |
| 1 | 1 | 1.62E+08 | 162367183 | FCGR2B,FCRLA,FCRLB,DUSP12,ATF6,OLFML2B,NOS1AP,NOS1AP,MIR4654,NOS1AP,MIR556,SPATA46,C1orf226,SH2D1B | 0.43411 | 398.876 | 100 | 58.5976 |
| 1 | 19 | 35249862 | 36429874 | ZNF599,ZNF30,ZNF792,GRAMD1A,SCN1B,HPN,HPN,HPN-AS1,HPN-AS1,FXYD3,LGI4,FXYD1,FXYD7,FXYD5,FAM187B,LSR,USF2,HAMP,MAG,CD22,CD22,MIR5196,FFAR1,FFAR3,GPR42,LINC01531,FFAR2,KRTDAP,DMKN,SBSN,GAPDHS,GAPDHS,TMEM147-AS1,TMEM147-AS1,TMEM147,TMEM147,ATP4A,HAUS5,RBM42,ETV2,COX6B1,UPK1A,UPK1A,UPK1A-AS1,ZBTB32,KMT2B,IGFLR1,IGFLR1,U2AF1L4,U2AF1L4,PSENEN,LIN37,HSPB6,PROSER3,ARHGAP33,ARHGAP33,LINC01529,PRODH2,NPHS1,KIRREL2,APLP1,NFKBID,HCST,TYROBP | 0.429974 | 401.281 | 507 | 326.388 |
| 1 | 20 | 58498018 | 59830008 | FAM217B,PPP1R3D,FAM217B,CDH26,C20orf197,MIR646HG,MIR646,MIR4533,MIR548AG2,CDH4 | 0.427622 | 384.657 | 59 | 33.291 |
| 1 | 17 | 7734619 | 7762213 | DNAH2,KDM6B,TMEM88,NAA38,NAA38,CYB5D1 | 0.421889 | 401.886 | 40 | 26.5091 |
| 1 | X | 1.53E+08 | 153905854 | DUSP9,PNCK,SLC6A8,BCAP31,ABCD1,PLXNB3,SRPK3,IDH3G,IDH3G,SSR4,SSR4,PDZD4,L1CAM,L1CAM,LCA10,LCA10,AVPR2,ARHGAP4,NAA10,RENBP,HCFC1,TMEM187,MIR3202-1,MIR3202-2,TMEM187,IRAK1,MECP2,OPN1LW,OPN1MW2,OPN1MW,OPN1MW3,TKTL1,FLNA,EMD,RPL10,RPL10,SNORA70,DNASE1L1,DNASE1L1,TAZ,TAZ,ATP6AP1,GDI1,FAM50A,FAM50A,MIR6858,PLXNA3,LAGE3,UBL4A,SLC10A3,FAM3A,G6PD,G6PD,IKBKG,IKBKG,CTAG1B,CTAG1A,CTAG2 | 0.420362 | 401.736 | 534 | 336.306 |

| | | | | | | | | |
|---|---|---|---|---|---|---|---|---|
| 1 | 3 | 50155700 | 50677215 | RBM5,SEMA3F-AS1,SEMA3F-AS1,SEMA3F,SEMA3F,GNAT1,SLC38A3,GNAI2,SEMA3B,SEMA3B,MIR6872,LSMEM2,IFRD2,HYAL3,HYAL3,NAT6,HYAL1,HYAL2,TUSC2,RASSF1,RASSF1,RASSF1-AS1,ZMYND10,NPRL2,NPRL2,CYB561D2,CYB561D2,CYB561D2,TMEM115,CYB561D2,CACNA2D2,CACNA2D2,C3orf18,HEMK1,CISH,MAPKAPK3 | 0.417326 | 365.347 | 210 | 135.473 |
| 1 | 22 | 24237167 | 24342396 | MIF-AS1,MIF,MIF-AS1,GSTT2B,GSTT2,DDTL,DDTL,DDT,DDT,GSTT2,GSTT2B,GSTTP1 | 0.412725 | 266.709 | 29 | 14.0506 |
| 1 | 7 | 82538165 | 82791870 | PCLO | 0.412158 | 405.589 | 54 | 38.3412 |
| 1 | 1 | 1.85E+08 | 186316595 | SWT1,IVNS1ABP,HMCN1,PRG4,PRG4,TPR,TPR | 0.407104 | 437.948 | 190 | 119.268 |
| 1 | 13 | 34013714 | 35785324 | STARD13,RFC3,NBEA | 0.402054 | 369.845 | 68 | 40.0693 |
| 1 | 16 | 1918690 | 2810546 | HS3ST6,MSRB1,RPL3L,NDUFB10,RPS2,RPS2,SNORA10,RPS2,SNORA64,SNHG9,SNORA78,RNF151,TBL3,NOXO1,GFER,SYNGR3,ZNF598,NPW,SLC9A3R2,NTHL1,NTHL1,TSC2,TSC2,PKD1,PKD1,MIR1225,PKD1,LOC105371049,RAB26,SNHG19,SNORD60,TRAF7,TRAF7,CASKIN1,CASKIN1,MLST8,MLST8,BRICD5,BRICD5,PGP,E4F1,DNASE1L2,ECI1,RNPS1,LOC106660606,MIR3677,LOC106660606,MIR940,MIR4717,ABCA3,CCNF,CCNF,MIR6767,TEDC2,TEDC2,MIR6768,NTN3,TBC1D24,ATP6V0C,AMDHD2,AMDHD2,CEMP1,MIR3178,PDPK1,LOC652276,FLJ42627,ERVK13-1,KCTD5,PRSS27,SRRM2-AS1,SRRM2 | 0.397097 | 397.3 | 465 | 287.683 |
| 1 | 1 | 42045722 | 42334805 | HIVEP3 | 0.39177 | 282.887 | 21 | 14.2144 |
| 1 | 10 | 75560774 | 75561469 | ZSWIM8,ZSWIM8-AS1,ZSWIM8 | 0.388758 | 519.783 | 3 | 2.0854 |
| 1 | 9 | 37428505 | 38527753 | GRHPR,ZBTB5,POLR1E,FBXO10,TOMM5,FRMPD1,TRMT10B,EXOSC3,DCAF10,SLC25A51,SHB,ALDH1B1,IGFBPL1 | 0.388447 | 366.807 | 137 | 86.4237 |
| 1 | 3 | 49413546 | 50005951 | TCTA,AMT,AMT,NICN1,NICN1,DAG1,BSN,APEH,MST1,RNF123,RNF123,AMIGO3,RNF123,GMPPB,GMPPB,IP6K1,CDHR4,FAM212A,UBA7,UBA7,MIR5193,TRAIP,CAMKV,MST1R,MON1A,RBM6 | 0.387066 | 383.336 | 287 | 192.253 |
| 1 | 9 | 33568663 | 35956694 | ANKRD18B,ANXA2P2,PTENP1,PRSS3,UBE2R2,UBAP2,UBAP2,SNORD121B,UBAP2,SNORD121A,DCAF12,UBAP1,KIF24,NUDT2,MYORG,C9orf24,FAM219A,DNAI1,ENHO,CNTFR,CNTFR,CNTFR-AS1,RPP25L,DCTN3,ARID3C,SIGMAR1,GALT,IL11RA,IL11RA,CCL27,CCL27,LOC730098,CCL19,CCL21,FAM205A,FAM205BP,PHF24,DNAJB5,C9orf131,VCP,FANCG,PIGO,STOML2,FAM214B,UNC13B,RUSC2,RUSC2,FAM166B,FAM166B,TESK1,TESK1,MIR4667,CD72,SIT1,CCDC107,CCDC107,ARHGEF39,ARHGEF39,CA9,TPM2,TLN1,TLN1,MIR6852,CREB3,MIR6853,CREB3,GBA2,RGP1,RGP1,MSMP,MSMP,NPR2,NPR2,SPAG8,SPAG8,HINT2,FAM221B,TMEM8B,OR13J1,HRCT1,SPAAR | 0.386267 | 381.928 | 696 | 441.191 |

| | | | | | | | | |
|---|---|---|---|---|---|---|---|---|
| 1 | 8 | 1.42E+08 | 144894625 | DENND3,SLC45A4,SLC45A4,LOC105375787,LOC105375787,GPR20,PTP4A3,MROH5,MIR1302-7,MIR4472-1,TSNARE1,ADGRB1,ARC,JRK,PSCA,LY6K,LY6K,LOC100288181,THEM6,SLURP1,LYPD2,SLURP2,LYNX1-SLURP2,LYNX1-SLURP2,LYNX1,LY6D,GML,CYP11B1,CYP11B2,LY6E-DT,LY6E,C8orf31,LY6H,GPIHBP1,ZFP41,GLI4,ZNF696,TOP1MT,RHPN1,MAFA,ZC3H3,GSDMD,MROH6,NAPRT,EEF1D,TIGD5,PYCR3,TSTA3,ZNF623,ZNF707,CCDC166,MAPK15,FAM83H,FAM83H,MIR4664,FAM83H-AS1,SCRIB | 0.37612 | 345.12 | 495 | 307.086 |
| 1 | 9 | 79007303 | 79814290 | RFK,GCNT1,PRUNE2,FOXB2,VPS13A-AS1,VPS13A | 0.3739 | 383.188 | 76 | 50.7778 |
| 1 | 16 | 60500 | 711167 | POLR3K,SNRNP25,RHBDF1,MPG,MPG,NPRL3,NPRL3,HBZ,HBM,HBA2,HBA1,HBQ1,LUC7L,FAM234A,FAM234A,RGS11,RGS11,ARHGDIG,PDIA2,AXIN1,MRPL28,TMEM8A,LOC100134368,NME4,DECR2,RAB11FIP3,CAPN15,MIR5587,CAPN15,MIR3176,CAPN15,PRR35,NHLRC4,PIGQ,RAB40C,WFIKKN1,METTL26,LOC100287175,MCRIP2,LOC105371038,WDR90,WDR90 | 0.37294 | 381.495 | 315 | 199.355 |
| 1 | 12 | 49448609 | 50383590 | KMT2D,RHEBL1,DHH,LMBR1L,TUBA1B,TUBA1A,TUBA1C,LOC101927267,PRPH,LOC101927267,TROAP,TROAP,C1QL4,DNAJC22,SPATS2,KCNH3,MCRS1,FAM186B,PRPF40B,PRPF40B,FMNL3,FMNL3,TMBIM6,NCKAP5L,BCDIN3D-AS1,BCDIN3D,BCDIN3D,FAIM2,LINC02396,AQP2,AQP2,LOC101927318,LOC101927318,AQP5,AQP5,AQP6 | 0.371801 | 353.142 | 290 | 181.68 |
| 1 | 16 | 2821263 | 5041977 | SRRM2,ELOB,ELOB,PRSS33,PRSS41,PRSS21,ZG16B,PRSS30P,PRSS22,FLYWCH2,FLYWCH1,KREMEN2,PAQR4,PAQR4,PKMYT1,PKMYT1,CLDN9,CLDN6,TNFRSF12A,HCFC1R1,HCFC1R1,THOC6,THOC6,BICDL2,BICDL2,LOC100128770,LOC100128770,MMP25,MMP25,MMP25-AS1,IL32,ZSCAN10,ZNF205-AS1,ZNF205-AS1,ZNF205,ZNF205,ZNF213,CASP16P,OR1F1,OR1F2P,ZNF200,MEFV,ZNF263,TIGD7,ZNF75A,OR2C1,ZSCAN32,ZNF174,ZNF597,NAA60,NAA60,MIR6126,C16orf90,CLUAP1,NLRC3,SLX4,DNASE1,TRAP1,CREBBP,ADCY9,SRL,TFAP4,GLIS2,PAM16,CORO7-PAM16,CORO7-PAM16,CORO7,CORO7-PAM16,CORO7,VASN,DNAJA3,NMRAL1,HMOX2,CDIP1,C16orf96,UBALD1,MGRN1,MGRN1,MIR6769A,NUDT16L1,ANKS3,C16orf71,C16orf71,ZNF500,ZNF500,SEPT12,SMIM22,ROGDI,GLYR1,UBN1,PPL,SEC14L5 | 0.371605 | 372.688 | 752 | 475.095 |
| 1 | X | 73045726 | 75651199 | TSIX,XIST,XIST,FTX,FTX,MIR421,MIR374B,MIR374C,FTX,MIR545,MIR374A,ZCCHC13,SLC16A2,RLIM,NEXMIF,ABCB7,UPRT,ZDHHC15,TTC3P1,MAGEE2,PBDC1,MAGEE1 | 0.367721 | 379.405 | 205 | 127.76 |
| 1 | 12 | 1.23E+08 | 123636581 | HCAR2,HCAR3,HCAR1,DENR,CCDC62,HIP1R,VPS37B,ABCB9,OGFOD2,ARL6IP4,PITPNM2,PITPNM2,MIR4304 | 0.35475 | 358.287 | 144 | 89.7439 |

| | | | | | | | | |
|---|---|---|---|---|---|---|---|---|
| 1 | 1 | 1.6E+08 | 161016926 | ATP1A2,ATP1A4,CASQ1,LOC729867,PEA15,PEA15,DCAF8,PEX19,COPA,COPA,NCSTN,NCSTN,NHLH1,VANGL2,SLAMF6,CD84,SLAMF1,CD48,SLAMF7,LY9,CD244,ITLN1,LOC101928372,ITLN2,ITLN2,F11R,TSTD1,USF1,ARHGAP30 | 0.346366 | 393.64 | 263 | 162.242 |
| 1 | 1 | 1.57E+08 | 159174918 | LRRC71,ARHGEF11,MIR765,ARHGEF11,ETV3L,ETV3,FCRL5,FCRL4,FCRL3,FCRL2,FCRL1,CD5L,KIRREL1,CD1D,CD1A,CD1C,CD1B,CD1E,OR10T2,OR10K2,OR10K1,OR10R2,OR6Y1,OR6P1,OR10X1,OR10Z1,SPTA1,OR6K2,OR6K3,OR6K6,OR6N1,OR6N2,MNDA,PYHIN1,IFI16,AIM2,CADM3,CADM3,CADM3-AS1,ACKR1 | 0.343595 | 386.444 | 416 | 249.166 |
| 1 | 3 | 1.14E+08 | 114280999 | DRD3,ZNF80,TIGIT,ZBTB20,MIR568,ZBTB20,ZBTB20,ZBTB20-AS1 | 0.342382 | 381.658 | 30 | 18.1354 |
| 1 | 11 | 85374445 | 85445792 | CREBZF,CCDC89,SYTL2 | 0.338598 | 392.539 | 43 | 30.4102 |
| 1 | 1 | 1.11E+08 | 111216916 | KCNC4,RBM15,SLC16A4,LAMTOR5,LAMTOR5,LAMTOR5-AS1,PROK1,KCNA10,KCNA2,KCNA3 | 0.329066 | 369.811 | 66 | 42.5126 |
| 1 | 8 | 20083272 | 23538995 | LZTS1,GFRA2,DOK2,XPO7,NPM2,FGF17,DMTN,FAM160B2,NUDT18,HR,REEP4,LGI3,SFTPC,BMP1,PHYHIP,MIR320A,POLR3D,POLR3D,PIWIL2,SLC39A14,PPP3CC,SORBS3,PDLIM2,C8orf58,CCAR2,BIN3,EGR3,PEBP4,RHOBTB2,TNFRSF10B,TNFRSF10B,LOC286059,LOC254896,TNFRSF10C,TNFRSF10C,TNFRSF10D,TNFRSF10A,CHMP7,R3HCC1,LOXL2,LOXL2,LOC100507156,ENTPD4,SLC25A37,NKX3-1 | 0.328816 | 359.301 | 517 | 320.845 |

| | | | | | | | | |
|---|---|---|---|---|---|---|---|---|
| 1 | 12 | 50854882 | 59307196 | LARP4,DIP2B,ATF1,TMPRSS12,METTL7A,HIGD1C,SLC11A2,LETMD1,LETMD1,CSRNP2,CSRNP2,TFCP2,POU6F1,DAZAP2,DAZAP2,SMAGP,SMAGP,BIN2,CELA1,GALNT6,SLC4A8,SCN8A,FIGNL2,ANKRD33,ACVRL1,ACVR1B,GRASP-AS1,GRASP,GRASP,NR4A1,ATG101,OR7E47P,KRT80,C12orf80,KRT7,KRT7,KRT7-AS,KRT87P,KRT86,KRT81,KRT86,KRT83,KRT85,KRT84,KRT82,KRT75,KRT6B,KRT6C,KRT6A,KRT5,KRT71,KRT74,KRT72,KRT73,KRT73,KRT73-AS1,KRT2,KRT1,KRT77,KRT76,KRT3,KRT4,KRT79,KRT78,KRT8,KRT8,KRT18,KRT18,EIF4B,LOC283335,TNS2,TNS2,TNS2,MIR6757,SPRYD3,IGFBP6,SOAT2,CSAD,CSAD,ZNF740,ZNF740,ITGB7,RARG,MFSD5,ESPL1,PFDN5,C12orf10,AAAS,SP7,SP1,AMHR2,PRR13,PCBP2,PCBP2,PCBP2-OT1,PCBP2,MAP3K12,MAP3K12,TARBP2,NPFF,ATF7,LOC100652999,ATF7,ATP5MC2,CALCOCO1,HOXC13-AS,HOXC13,HOXC13,HOXC12,HOTAIR,HOXC11,HOXC11,HOXC-AS3,HOXC10,HOXC10,MIR196A2,HOXC9,HOXC8,HOXC6,HOXC5,HOXC4,HOXC5,HOXC4,HOXC5,HOXC4,MIR615,HOXC4,FLJ12825,LOC100240734,LINC02381,SMUG1,CBX5,MIR3198-2,CBX5,HNRNPA1,HNRNPA1P10,NFE2,COPZ1,COPZ1,MIR148B,LOC102724050,GPR84,LOC102724050,ZNF385A,LOC102724050,ITGA5,LOC102724050,GTSF1,GTSF1,NCKAP1L,PDE1B,PPP1R1A,GLYCAM1,LACRT,DCD,MUCL1,TESPA1,NEUROD4,OR9K2,OR10A7,OR6C74,OR6C6,OR6C1,OR6C3,OR6C75,OR6C65,OR6C76,OR6C2,OR6C70,OR6C68,OR6C4,OR2AP1,OR10P1,METTL7B,ITGA7,BLOC1S1,BLOC1S1-RDH5,BLOC1S1-RDH5,RDH5,CD63,GDF11,SARNP,ORMDL2,DNAJC14,T | 0.325181 | 372.112 | 2606 | 1605.03 |
| 1 | 16 | 84120922 | 90294253 | MBTPS1,HSDL1,DNAAF1,DNAAF1,TAF1C,TAF1C,ADAD2,ADAD2,LOC654780,KCNG4,WFDC1,ATP2C2,ATP2C2,ATP2C2-AS1,TLDC1,COTL1,KLHL36,USP10,CRISPLD2,ZDHHC7,KIAA0513,FAM92B,MIR5093,GSE1,GINS2,C16orf74,C16orf74,MIR1910,EMC8,COX4I1,IRF8,LINC00917,FOXF1,MTHFSD,FLJ30679,FOXC2-AS1,FOXC2,FOXC2,FOXL1,C16orf95,FBXO31,MAP1LC3B,ZCCHC14,JPH3,KLHDC4,SLC7A5,CA5A,BANP,LOC400553,ZNF469,ZFPM1,ZFPM1,MIR5189,ZFPM1,LOC100128882,ZC3H18,IL17C,CYBA,MVD,SNAI3-AS1,SNAI3,RNF166,CTU2,PIEZO1,PIEZO1,MIR4722,PIEZO1,LOC100289580,PIEZO1,LOC339059,CDT1,APRT,GALNS,GALNS,TRAPPC2L,TRAPPC2L,PABPN1L,CBFA2T3,CBFA2T3,LOC101927793,CBFA2T3,LOC100129697,ACSF3,LINC02138,CDH15,SLC22A31,ZNF778,ANKRD11,ANKRD11,LOC100287036,SPG7,RPL13,RPL13,SNORD68,CPNE7,DPEP1,CHMP1A,CHMP1A,SPATA33,SPATA33,CDK10,SPATA2L,VPS9D1,VPS9D1,VPS9D1-AS1,VPS9D1,ZNF276,ZNF276,ZNF276,FANCA,FANCA,SPIRE2,TCF25,MC1R,TUBB3,DEF8,CENPBD1,AFG3L1P,DBNDD1,DBNDD1,GAS8,GAS8,GAS8,GAS8-AS1,GAS8,URAHP,PRDM7 | 0.32286 | 351.033 | 1144 | 704.925 |

| | | | | | | | | |
|---|---|---|---|---|---|---|---|---|
| 1 | 8 | 10500 | 1808325 | ZNF596,FAM87A,FBXO25,TDRP,ERICH1,DLGAP2,DLGAP2,DLGAP2-AS1,CLN8,MIR3674,MIR596,ARHGEF10 | 0.322194 | 313.552 | 114 | 62.7286 |
| 1 | 3 | 48448362 | 48788858 | PLXNB1,CCDC51,TMA7,ATRIP,ATRIP,TREX1,TREX1,SHISA5,PFKFB4,PFKFB4,MIR6823,UCN2,COL7A1,COL7A1,MIR711,UQCRC1,TMEM89,SLC26A6,SLC26A6,MIR6824,CELSR3,CELSR3,MIR4793,CELSR3-AS1,NCKIPSD,IP6K2,PRKAR2A | 0.321446 | 363.782 | 298 | 198.459 |
| 1 | 1 | 1.76E+08 | 185069140 | RFWD2,PAPPA2,ASTN1,ASTN1,MIR488,BRINP2,SEC16B,CRYZL2P-SEC16B,RASAL2-AS1,RASAL2,TEX35,C1orf220,MIR4424,RALGPS2,RALGPS2,ANGPTL1,FAM20B,TOR3A,ABL2,SOAT1,AXDND1,AXDND1,NPHS2,NPHS2,TDRD5,FAM163A,TOR1AIP2,TOR1AIP1,CEP350,QSOX1,FLJ23867,LHX4,LHX4,LHX4-AS1,ACBD6,XPR1,KIAA1614,KIAA1614-AS1,STX6,MR1,IER5,CACNA1E,ZNF648,GLUL,TEDDM1,RGSL1,RNASEL,RGS16,RGS8,NPL,DHX9,SHCBP1L,LAMC1,LAMC1,LAMC1-AS1,LAMC2,NMNAT2,SMG7,NCF2,ARPC5,RGL1,APOBEC4,RGL1,COLGALT2,TSEN15,C1orf21,EDEM3,FAM129A,RNF2 | 0.318923 | 372.408 | 1017 | 611.682 |
| 1 | 1 | 1.74E+08 | 175956156 | SLC9C2,ANKRD45,ANKRD45,TEX50,KLHL20,CENPL,DARS2,GAS5,SNORD81,GAS5,SNORD47,GAS5,SNORD80,GAS5,SNORD79,GAS5,SNORD78,GAS5,SNORD44,GAS5,SNORA103,SNORD77,GAS5,SNORD76,GAS5,SNORD75,GAS5,SNORD74,ZBTB37,SERPINC1,RC3H1,RABGAP1L,RABGAP1L,GPR52,CACYBP,MRPS14,TNN,KIAA0040,TNR,RFWD2,RFWD2,SCARNA3 | 0.315752 | 397.262 | 237 | 141.241 |
| 1 | 19 | 37642967 | 41883656 | ZNF585A,ZNF585B,ZNF383,HKR1,ZNF527,ZNF569,ZNF570,ZNF793,ZNF571-AS1,ZNF571-AS1,ZNF540,ZNF571-AS1,ZNF540,ZNF571,ZNF540,ZFP30,ZNF781,ZNF607,ZNF573,LOC644554,LOC100631378,WDR87,SIPA1L3,DPF1,PPP1R14A,SPINT2,YIF1B,C19orf33,YIF1B,KCNK6,CATSPERG,PSMD8,GGN,SPRED3,FAM98C,RASGRP4,RYR1,MAP4K1,MAP4K1,LOC105372397,EIF3K,ACTN4,CAPN12,LGALS7,LGALS7B,LGALS4,ECH1,HNRNPL,RINL,SIRT2,NFKBIB,CCER2,SARS2,SARS2,MRPS12,MRPS12,FBXO17,FBXO27,ACP7,PAK4,NCCRP1,SYCN,IFNL3,IFNL2,IFNL1,LRFN1,GMFG,SAMD4B,PAF1,MED29,ZFP36,MIR4530,PLEKHG2,RPS16,SUPT5H,TIMM50,DLL3,SELENOV,EID2B,EID2,LGALS13,LGALS16,LGALS17A,LGALS14,CLC,LEUTX,DYRK1B,FBL,FCGBP,PSMC4,ZNF546,ZNF780B,ZNF780A,MAP3K10,TTC9B,CNTD2,AKT2,AKT2,MIR641,C19orf47,C19orf47,PLD3,PLD3,PLD3,MIR6796,HIPK4,PRX,SERTAD1,SERTAD3,BLVRB,SPTBN4,SHKBP1,LTBP4,NUMBL,COQ8B,ITPKC,C19orf54,SNRPA,MIA,MIA-RAB4B,MIA-RAB4B,RAB4B,RAB4B-EGLN2,RAB4B-EGLN2,EGLN2,CYP2T1P,CYP2A6,CYP2A7,CYP2G1P,CYP2B7P,CYP2B6,CYP2A13,CYP2F1,CYP2S1,AXL,HNRNPUL1,CCDC97,TGFB1,B9D2 | 0.308407 | 382.374 | 1475 | 901.207 |

| | | | | | | | | |
|---|---|---|---|---|---|---|---|---|
| 1 | 19 | 12768995 | 13316871 | MAN2B1,WDR83,WDR83OS,WDR83,WDR83,DHPS,DHPS,GNG14,FBXW9,TNPO2,TNPO2,SNORD135,TNPO2,SNORD41,TRIR,ASNA1,BEST2,HOOK2,JUNB,PRDX2,RNASEH2A,RTBDN,MAST1,MAST1,MIR6794,DNASE2,KLF1,GCDH,GCDH,SYCE2,SYCE2,FARSA,FARSA,FARSA-AS1,CALR,CALR,MIR6515,RAD23A,GADD45GIP1,DAND5,NFIX,LYL1,TRMT1,NACC1,STX10,IER2 | 0.301412 | 365.261 | 276 | 177.664 |
| 1 | 14 | 23979187 | 24912092 | THTPA,ZFHX2,THTPA,AP1G2,AP1G2,LOC102724814,JPH4,DHRS2,DHRS4-AS1,DHRS4-AS1,DHRS4,DHRS4,DHRS4L2,DHRS4L1,CARMIL3,CPNE6,NRL,NRL,PCK2,DCAF11,FITM1,PSME1,EMC9,PSME2,MIR7703,PSME2,RNF31,IRF9,REC8,REC8,IPO4,IPO4,TM9SF1,TSSK4,CHMP4A,MDP1,NEDD8-MDP1,NEDD8-MDP1,NEDD8,GMPR2,TINF2,TGM1,RABGGTA,DHRS1,NOP9,NOP9,CIDEB,CIDEB,LTB4R2,CIDEB,LTB4R2,LTB4R,LTB4R2,LTB4R,LTB4R,ADCY4,RIPK3,NFATC4,NYNRIN,CBLN3,CBLN3,KHNYN,KHNYN,KHNYN,SDR39U1,SDR39U1 | 0.298811 | 354.269 | 514 | 334.042 |
| 1 | 16 | 780360 | 1884403 | NARFL,MSLN,MIR662,RPUSD1,CHTF18,GNG13,PRR25,LMF1,LMF1,LMF1-AS1,SOX8,SSTR5-AS1,SSTR5,C1QTNF8,CACNA1H,CACNA1H,TPSG1,TPSG1,TPSB2,TPSAB1,TPSD1,UBE2I,BAIAP3,BAIAP3,TSR3,TSR3,GNPTG,UNKL,C16orf91,CCDC154,CLCN7,PTX4,TELO2,IFT140,IFT140,TMEM204,IFT140,LOC105371046,CRAMP1,JPT2,MAPK8IP3,MAPK8IP3,MIR3177,NME3,MRPS34,MRPS34,EME2,EME2,SPSB3,NUBP2,IGFALS,HAGH,FAHD1,FAHD1,MEIOB | 0.297166 | 370.725 | 530 | 334.491 |
| 1 | 12 | 50410438 | 50821753 | RACGAP1,ASIC1,SMARCD1,GPD1,COX14,CERS5,LIMA1,FAM186A,LARP4 | 0.294287 | 340.471 | 115 | 70.3939 |
| 1 | 20 | 21142469 | 26256532 | KIZ,KIZ,KIZ-AS1,XRN2,NKX2-4,NKX2-2,PAX1,FOXA2,SSTR4,THBD,CD93,NXT1,GZF1,NAPB,CSTL1,CST11,CST8,CST9L,CST9,CST3,CST4,CST1,CST2,CST5,GGTLC1,SYNDIG1,CST7,APMAP,ACSS1,VSX1,LOC101926889,ENTPD6,ENTPD6,PYGB,PYGB,ABHD12,ABHD12,GINS1,NINL,NANP,ZNF337-AS1,ZNF337,ZNF337,FAM182B,LOC101926935,FAM182A,NCOR1P1,MIR663AHG,MIR663A | 0.293488 | 367.446 | 371 | 220.978 |

| | | | | | | | | |
|---|---|---|---|---|---|---|---|---|
| 1 | 5 | 1.4E+08 | 141352597 | SRA1,APBB3,APBB3,MIR6831,SLC35A4,CD14,TMCO6,NDUFA2,IK,MIR3655,IK,WDR55,DND1,HARS,HARS,HARS2,HARS2,ZMAT2,PCDHA1,PCDHA1,PCDHA2,PCDHA1,PCDHA2,PCDHA3,PCDHA1,PCDHA2,PCDHA3,PCDHA4,PCDHA1,PCDHA2,PCDHA3,PCDHA4,PCDHA5,PCDHA1,PCDHA2,PCDHA3,PCDHA4,PCDHA5,PCDHA6,PCDHA1,PCDHA2,PCDHA3,PCDHA4,PCDHA5,PCDHA6,PCDHA7,PCDHA1,PCDHA2,PCDHA3,PCDHA4,PCDHA5,PCDHA6,PCDHA7,PCDHA8,PCDHA1,PCDHA2,PCDHA3,PCDHA4,PCDHA5,PCDHA6,PCDHA7,PCDHA8,PCDHA9,PCDHA1,PCDHA2,PCDHA3,PCDHA4,PCDHA5,PCDHA6,PCDHA7,PCDHA8,PCDHA9,PCDHA10,PCDHA1,PCDHA2,PCDHA3,PCDHA4,PCDHA5,PCDHA6,PCDHA7,PCDHA8,PCDHA9,PCDHA10,PCDHA11,PCDHA1,PCDHA2,PCDHA3,PCDHA4,PCDHA5,PCDHA6,PCDHA7,PCDHA8,PCDHA9,PCDHA10,PCDHA11,PCDHA12,PCDHA1,PCDHA2,PCDHA3,PCDHA4,PCDHA5,PCDHA6,PCDHA7,PCDHA8,PCDHA9,PCDHA10,PCDHA11,PCDHA12,PCDHA13,PCDHA1,PCDHA2,PCDHA3,PCDHA4,PCDHA5,PCDHA6,PCDHA7,PCDHA8,PCDHA9,PCDHA10,PCDHA11,PCDHA12,PCDHA13,PCDHAC1,PCDHA1,PCDHA2,PCDHA3,PCDHA4,PCDHA5,PCDHA6,PCDHA7,PCDHA8,PCDHA9,PCDHA10,PCDHA11,PCDHA12,PCDHA13,PCDHAC1,PCDHAC2,LOC101926905,PCDHB1,PCDHB2,PCDHB3,PCDHB4,PCDHB5,PCDHB6,PCDHB17P,PCDHB7,PCDHB8,PCDHB16,PCDHB9,PCDHB10,PCDHB11,PCDHB12,PCDHB13,PCDHB14,PCDHB18P,PCDHB19P,PCDHB15,SLC25A2,TAF7,PCDHGA1,PCDHGA1,PCDHGA2,PCDHGA1,PCDHGA2,PCDHGA3,PCDHGA1,PCDHGA2,PCDHGA3,PCDHGB1,PCDHGA1,PCDHGA2,PCDHGA3,PCDHGB1,PCDHGA4,PCDHGA1,PCDHGA2,PCDHGA3,PCDHGB1,PCDHGA4,PCDHGB2,PCDHGA1,PCDHGA2,PCDHGA3,PCDHGB1,PCDHGA4,PCDHGB2,PCDHGA5,PCDHGA1,PCDHGA2,PCDHGA3,PCDH | 0.289851 | 401.888 | 746 | 485.311 |
| 1 | 12 | 1.24E+08 | 133841395 | DNAH10,CCDC92,ZNF664-RFLNA,ZNF664,ZNF664-RFLNA,RFLNA,NCOR2,NCOR2,MIR6880,SCARB1,UBC,MIR5188,DHX37,BRI3BP,AACS,TMEM132B,LOC283435,LINC02347,LINC02347,LINC00944,LINC00943,TMEM132C,TMEM132C,MIR3612,SLC15A4,GLT1D1,TMEM132D,TMEM132D,TMEM132D-AS2,LINC02418,FZD10,PIWIL1,RIMBP2,STX2,RAN,ADGRD1,ADGRD1,ADGRD1-AS1,LINC01257,SFSWAP,MMP17,ULK1,PUS1,EP400,EP400,SNORA49,EP400P1,DDX51,NOC4L,GALNT9,GALNT9,LOC100130238,FBRSL1,LRCOL1,P2RX2,POLE,PXMP2,PGAM5,ANKLE2,GOLGA3,CHFR,ZNF605,ZNF26,ZNF84,ZNF140,ZNF891,ZNF10,ZNF268,ANHX | 0.287356 | 324.539 | 881 | 526.442 |

| | | | | | | | | |
|---|---|---|---|---|---|---|---|---|
| 1 | 8 | 6602679 | 15531391 | AGPAT5,MIR4659A,MIR4659B,AGPAT5,XKR5,XKR5,GS1-24F4.2,DEFB1,DEFA6,DEFA4,DEFA5,DEFB4B,DEFB103B,DEFB103A,SPAG11B,DEFB104A,DEFB104B,DEFB106B,DEFB106A,DEFB105B,DEFB105A,DEFB107A,DEFB107B,PRR23D1,PRR23D2,PRR23D2,PRR23D1,SPAG11B,SPAG11A,SPAG11A,DEFB4A,PRAG1,CLDN23,MFHAS1,ERI1,ERI1,MIR4660,PPP1R3B,TNKS,TNKS,MIR597,MIR124-1,MSRA,PRSS55,RP1L1,MIR4286,C8orf74,SOX7,SOX7,LOC102723313,LOC102723313,PINX1,PINX1,PINX1,MIR1322,XKR6,XKR6,MIR598,XKR6,LOC101929269,MTMR9,SLC35G5,TDH,LOC100129129,FAM167A-AS1,FAM167A,FAM167A,BLK,GATA4,C8orf49,NEIL2,FDFT1,CTSB,DEFB136,DEFB135,DEFB134,LOC729732,MIR5692A1,MIR5692A2,LONRF1,LONRF1,MIR3926-1,MIR3926-2,KIAA1456,DLC1,DLC1,C8orf48,SGCZ,SGCZ,MIR383,TUSC3 | 0.28286 | 334.258 | 547 | 325.026 |
| 1 | 7 | 10500 | 6821140 | FAM20C,PDGFA,PRKAR1B,PRKAR1B,LOC101926963,PRKAR1B,DNAAF5,DNAAF5,SUN1,GET4,ADAP1,COX19,CYP2W1,C7orf50,C7orf50,MIR339,C7orf50,GPR146,C7orf50,GPER1,ZFAND2A,UNCX,MICALL2,INTS1,MAFK,LOC100128653,MAFK,TMEM184A,PSMG3,PSMG3-AS1,ELFN1,MAD1L1,MAD1L1,MIR4655,MRM2,MRM2,NUDT1,NUDT1,SNX8,SNX8,MIR6836,EIF3B,CHST12,GRIFIN,LFNG,LFNG,MIR4648,BRAT1,IQCE,TTYH3,AMZ1,AMZ1,GNA12,GNA12,CARD11,LOC100129603,SDK1,FOXK1,AP5Z1,AP5Z1,MIR4656,RADIL,RADIL,PAPOLB,MMD2,RNF216P1,RBAK,RBAK-RBAKDN,RBAK-RBAKDN,RBAKDN,ZNF890P,WIPI2,SLC29A4,TNRC18,LOC100129484,FBXL18,FBXL18,MIR589,ACTB,FSCN1,RNF216,RNF216,MIR6874,ZNF815P,OCM,CCZ1,RSPH10B2,RSPH10B,PMS2,AIMP2,AIMP2,EIF2AK1,EIF2AK1,EIF2AK1,ANKRD61,USP42,CYTH3,FAM220A,RAC1,DAGLB,KDELR2,GRID2IP,ZDHHC4,C7orf26,ZNF853,ZNF12,PMS2CL,RSPH10B,RSPH10B2 | 0.282559 | 345.443 | 1150 | 677.153 |
| 1 | 1 | 2.47E+08 | 247265220 | AHCTF1,ZNF695,ZNF670-ZNF695,ZNF670-ZNF695,ZNF670,ZNF669 | 0.28152 | 357.063 | 30 | 16.7226 |
| 1 | 17 | 60679494 | 62850131 | [illegible]633,TANC2,CYB561,ACE,KCNH6,DCAF7,TACO1,MAP3K3,LIMD2,STRADA,CCDC47,DDX42,FTSJ3,FTSJ3,PSMC5,PSMC5,SMARCD2,CSH2,GH2,CSH1,CSHL1,GH1,CD79B,SCN4A,PRR29-AS1,PRR29,PRR29,PRR29,ICAM2,ICAM2,ERN1,SNHG25,SNORD104,SNORA50C,TEX2,PECAM1,MILR1,POLG2,DDX5,DDX5,MIR3064,DDX5,MIR5047,DDX5,CEP95,CEP95,SMURF2,ARHGAP27P1-BPTFP1-KPNA2P3,ARHGAP27P1-BPTFP1-KPNA2P3,ARHGAP27P1,ARHGAP27P1-BPTFP1-KPNA2P3,ARHGAP27P1,MIR6080,PLEKHM1P1,PLEKHM1P1,MIR4315-1,MIR4315-2 | 0.276481 | 383.591 | 536 | 326.246 |

| | | | | | | | | |
|---|---|---|---|---|---|---|---|---|
| 1 | 1 | 2.26E+08 | 227843566 | DNAH14,LBR,ENAH,SRP9,EPHX1,TMEM63A,LEFTY1,PYCR2,PYCR2,MIR6741,LEFTY2,SDE2,H3F3A,H3F3AP4,ACBD3,ACBD3,ACBD3-AS1,MIXL1,LIN9,PARP1,STUM,ITPKB,PSEN2,COQ8A,CDC42BPA,ZNF678 | 0.274846 | 355.806 | 335 | 194.213 |
| 1 | 9 | 1.24E+08 | 139357968 | CNTRL,RAB14,GSN,GSN-AS1,GSN,STOM,GGTA1P,DAB2IP,TTLL11,MIR4478,NDUFA8,MORN5,LHX6,RBM18,MRRF,PTGS1,OR1J1,OR1J2,OR1J4,OR1N1,OR1N2,OR1L8,OR1Q1,OR1B1,OR1L1,OR1L3,OR1L4,OR1L6,OR5C1,OR1K1,PDCL,RC3H2,RC3H2,SNORD90,ZBTB6,ZBTB26,RABGAP1,RABGAP1,GPR21,MIR600HG,MIR600HG,MIR600,STRBP,CRB2,DENND1A,DENND1A,MIR601,LOC100505588,LHX2,NEK6,PSMB7,LOC100129034,PSMB7,NR5A1,NR6A1,NR6A1,MIR181A2HG,MIR181A2,NR6A1,MIR181A2HG,MIR181B2,OLFML2A,WDR38,RPL35,ARPC5L,GOLGA1,SCAI,PPP6C,RABEPK,HSPA5,GAPVD1,MAPKAP1,PBX3,MVB12B,LMX1B,ZBTB43,ZBTB34,RALGPS1,RALGPS1,ANGPTL2,GARNL3,SLC2A8,ZNF79,RPL12,RPL12,SNORA65,LRSAM1,FAM129B,STXBP1,STXBP1,MIR3911,CFAP157,CFAP157,PTRH1,PTRH1,TTC16,TOR2A,SH2D3C,MIR3960,MIR2861,CDK9,CDK9,FPGS,ENG,ENG,LOC102723566,AK1,AK1,MIR4672,ST6GALNAC6,ST6GALNAC4,PIP5KL1,DPM2,FAM102A,NAIF1,SLC25A25,PTGES2,PTGES2-AS1,LCN2,C9orf16,CIZ1,CIZ1,DNM1,DNM1,DNM1,MIR199B,MIR3154,GOLGA2,GOLGA2,SWI5,SWI5,TRUB2,COQ4,SLC27A4,SLC27A4,MIR1268A,MIR1268A,URM1,MIR1268A,MIR219A2,MIR219B,MIR1268A,CERCAM,MIR1268A,ODF2,MIR1268A,GLE1,MIR1268A,SPTAN1,MIR1268A,WDR34,SET,PKN3,ZDHHC12,LOC100506100,ZER1,ZER1,TBC1D13,ENDOG,ENDOG,SPOUT1,SPOUT1,KYAT1,LRRC8A,PHYHD1,DOLK,DOLK,NUP188,NUP188,SH3GLB2,MIGA2,DOLPP1,CRAT,PTPA,IER5L,C9orf106,NTMT1,C9orf50,NTMT1,NTMT1,ASB6,ASB6,PRRX2,PTGES,TOR1B,TOR1A,C9orf78,USP20,USP20,MIR6855,FNBP1,GPR107,LOC401554,NCS1,HMCN2,ASS1,FUBP3,PRDM12,EXOSC2,ABL1,QRFP,FIBCD1,LAMC3,AIF1L,NUP214,FAM78A,PLPP7,PRRC2B,PRRC2B,SNORD62A,SN | 0.273719 | 351.234 | 3180 | 1944.92 |

| | | | | | | | | |
|---|---|---|---|---|---|---|---|---|
| 1 | 6 | 60500 | 58745455 | LOC285766,DUSP22,IRF4,EXOC2,EXOC2,HUS1B,LINC01622,FOXQ1,FOXF2,FOXF2,MIR6720,FOXC1,GMDS,LINC01600,MYLK4,MYLK4,WRNIP1,WRNIP1,SERPINB1,MIR4645,LOC101927730,SERPINB9,SERPINB6,NQO2,RIPK1,BPHL,TUBB2A,TUBB2B,LOC100422781,PSMG4,SLC22A23,SLC22A23,LOC643327,PXDC1,FAM50B,PRPF4B,FAM217A,C6orf201,C6orf201,ECI2,ECI2,CDYL,RPP40,RPP40,LYRM4-AS1,LYRM4-AS1,LYRM4,PPP1R3G,LYRM4-AS1,LYRM4,LYRM4-AS1,LYRM4,MIR3691,LYRM4,FARS2,NRN1,F13A1,F13A1,MIR7853,MIR5683,LY86-AS1,LY86,LY86,RREB1,SSR1,CAGE1,RIOK1,DSP,SNRNP48,BMP6,BLOC1S5-TXNDC5,TXNDC5,BLOC1S5-TXNDC5,PIP5K1P1,BLOC1S5-TXNDC5,BLOC1S5,EEF1E1-BLOC1S5,EEF1E1-BLOC1S5,EEF1E1,SLC35B3,TFAP2A,TFAP2A,TFAP2A-AS2,TFAP2A,TFAP2A-AS1,LINC00518,MIR5689HG,MIR5689,GCNT2,C6orf52,PAK1IP1,TMEM14C,TMEM14B,MAK,GCM2,SYCP2L,SYCP2L,LOC101928191,ELOVL2,ELOVL2,ELOVL2-AS1,SMIM13,SMIM13,ERVFRD-1,NEDD9,TMEM170B,ADTRP,HIVEP1,EDN1,PHACTR1,PHACTR1,TBC1D7-LOC100130357,PHACTR1,TBC1D7-LOC100130357,LOC100130357,TBC1D7-LOC100130357,LOC100130357,TBC1D7-LOC100130357,TBC1D7,GFOD1,SIRT5,NOL7,NOL7,RANBP9,RANBP9,MCUR1,RNF182,CD83,JARID2,DTNBP1,MYLIP,MYLIP,MIR4639,GMPR,ATXN1,ATXN1,LOC101928433,STMND1,RBM24,CAP2,CAP2,LOC101928491,FAM8A1,NUP153,NUP153,LOC105374952,KIF13A,NHLRC1,TPMT,KDM1B,DEK,RNF144B,MIR548A1HG,MIR548A1,ID4,MBOAT1,E2F3,CDKAL1,SOX4,CASC15,NBAT1,PRL,HDGFL1,NRSN1,DCDC2,DCDC2,KAAG1,MRS2,GPL | 0.272466 | 353.707 | 7563 | 4585.69 |
| 1 | 9 | 1.4E+08 | 141152931 | EXD3,NOXA1,NOXA1,ENTPD8,ENTPD8,NSMF,NSMF,MIR7114,PNPLA7,MRPL41,DPH7,ZMYND19,ARRDC1,ARRDC1-AS1,EHMT1,EHMT1,LOC651337,MIR602,LOC100133077,CACNA1B,CACNA1B,CACNA1B,LOC101928786,TUBBP5 | 0.272379 | 361.783 | 235 | 144.451 |
| 1 | X | 54259212 | 58505576 | WNK3,TSR2,TSR2,FGD1,FGD1,GNL3L,ITIH6,MAGED2,MAGED2,SNORA11,TRO,PFKFB1,APEX2,ALAS2,PAGE2B,PAGE2,FAM104B,MTRNR2L10,PAGE5,PAGE3,MIR4536-1,MIR4536-2,MAGEH1,USP51,FOXR2,RRAGB,KLF8,UBQLN2,SPIN3,SPIN2B,SPIN2A,FAAH2,ZXDB,ZXDA | 0.271825 | 362.774 | 334 | 201.428 |

| | | | | | | | | |
|---|---|---|---|---|---|---|---|---|
| 1 | X | 1.01E+08 | 105855993 | BTK,RPL36A,RPL36A-HNRNPH2,RPL36A-HNRNPH2,GLA,RPL36A-HNRNPH2,HNRNPH2,ARMCX4,ARMCX1,ARMCX6,ARMCX3,ARMCX2,NXF5,ZMAT1,TCEAL2,TCEAL6,BEX5,TCP11X2,NXF2B,NXF2,TMSB15A,ARMCX5,ARMCX5-GPRASP2,ARMCX5-GPRASP2,GPRASP1,ARMCX5-GPRASP2,GPRASP2,ARMCX5-GPRASP2,BHLHB9,ARMCX5-GPRASP2,LINC00630,LINC00630,LINC00630,RAB40AL,BEX1,NXF3,BEX4,TCEAL8,TCEAL5,BEX2,TCEAL7,TCEAL9,BEX3,RAB40A,TCEAL4,TCEAL3,TCEAL1,MORF4L2,GLRA4,GLRA4,TMEM31,RAB9B,PLP1,RAB9B,TMSB15B,H2BFXP,LOC100101478,H2BFWT,H2BFM,SLC25A53,SLC25A53,ZCCHC18,FAM199X,ESX1,IL1RAPL2,IL1RAPL2,TEX13A,NRK,SERPINA7,MUM1L1,CXorf57 | 0.271605 | 362.924 | 477 | 284.044 |
| 1 | 2 | 1.8E+08 | 179643869 | TTN,TTN,LOC101927055 | 0.266823 | 421.172 | 135 | 99.4331 |
| 1 | 12 | 48104507 | 49418703 | ENDOU,RAPGEF3,SLC48A1,HDAC7,VDR,TMEM106C,COL2A1,SENP1,SENP1,PFKM,PFKM,ASB8,CCDC184,OR10AD1,H1FNT,ZNF641,ANP32D,C12orf54,OR8S1,LALBA,KANSL2,KANSL2,SNORA2C,MIR1291,KANSL2,SNORA2A,KANSL2,SNORA2B,CCNT1,TEX49,ADCY6,ADCY6,MIR4701,CACNB3,DDX23,RND1,CCDC65,FKBP11,ARF3,WNT10B,WNT1,DDN,DDN,DDN-AS1,DDN-AS1,PRKAG1,KMT2D | 0.264697 | 347.654 | 431 | 268.43 |
| 1 | 16 | 9017048 | 10525187 | USP7,C16orf72,GRIN2A,ATF7IP2 | 0.258786 | 275.169 | 55 | 33.5499 |
| 1 | 1 | 2.12E+08 | 225117504 | NEK2,LPGAT1,INTS7,DTL,DTL,MIR3122,PPP2R5A,PPP2R5A,SNORA16B,TMEM206,NENF,ATF3,FAM71A,BATF3,NSL1,TATDN3,SPATA45,FLVCR1,VASH2,ANGEL2,RPS6KC1,PROX1,SMYD2,PTPN14,CENPF,KCNK2,KCTD3,USH2A,USH2A,LOC102723833,ESRRG,GPATCH2,SPATA17,SPATA17,SPATA17-AS1,RRP15,TGFB2,TGFB2,MIR548F3,MIR548F3,C1orf143,LYPLAL1,LOC102723886,ZC3H11B,RNU5F-1,SLC30A10,RNU5F-1,EPRS,RNU5F-1,BPNT1,RNU5F-1,IARS2,RNU5F-1,IARS2,MIR215,RNU5F-1,IARS2,MIR194-1,IARS2,RAB3GAP2,RAB3GAP2,MIR664A,SNORA36B,MARK1,C1orf115,MARC2,MARC1,HLX-AS1,HLX,HLX,C1orf140,DUSP10,HHIPL2,TAF1A,MIA3,AIDA,BROX,FAM177B,DISP1,TLR5,SUSD4,CCDC185,CAPN8,CAPN2,TP53BP2,LOC100287497,FBXO28,DEGS1,LOC101927164,NVL,NVL,NVL,MIR320B2,CNIH4,WDR26,WDR26,MIR4742,CNIH3,DNAH14 | 0.257637 | 350.487 | 1114 | 671.461 |

| | | | | | | | | |
|---|---|---|---|---|---|---|---|---|
| 1 | 4 | 517309 | 10503079 | PIGG,PDE6B,ATP5ME,MYL5,MYL5,SLC49A3,SLC49A3,PCGF3,LOC100129917,CPLX1,GAK,TMEM175,DGKQ,SLC26A1,SLC26A1,IDUA,IDUA,FGFRL1,RNF212,SPON2,SPON2,LOC100130872,CTBP1-AS,CTBP1,CTBP1,CTBP1-AS2,MAEA,UVSSA,CRIPAK,NKX1-1,FAM53A,SLBP,TMEM129,TACC3,FGFR3,LETM1,NSD2,NSD2,SCARNA22,NELFA,NELFA,MIR943,C4orf48,NAT8L,POLN,POLN,HAUS3,MXD4,MIR4800,MXD4,ZFYVE28,CFAP99,RNF4,FAM193A,TNIP2,SH3BP2,ADD1,MFSD10,NOP14-AS1,NOP14,NOP14,GRK4,HTT,MSANTD1,RGS12,HGFAC,DOK7,LRPAP1,LINC00955,ADRA2C,OTOP1,TMEM128,LYAR,ZBTB49,NSG1,STX18,MSX1,CYTL1,STK32B,LINC01587,EVC2,EVC,CRMP1,MIR378D1,JAKMIP1,WFS1,PPP2R2C,MAN2B2,MRFAP1,LOC93622,S100P,MRFAP1L1,BLOC1S4,KIAA0232,TBC1D14,LOC100129931,CCDC96,LOC100129931,TADA2B,TADA2B,GRPEL1,SORCS2,SORCS2,MIR4798,SORCS2,PSAPL1,SORCS2,MIR4274,AFAP1-AS1,AFAP1,AFAP1,AFAP1,LOC389199,ABLIM2,ABLIM2,MIR95,SH3TC1,HTRA3,ACOX3,TRMT44,GPR78,CPZ,HMX1,USP17L6P,DRD5,SLC2A9,WDR1,WDR1,MIR3138,ZNF518B,CLNK | 0.254073 | 345.995 | 1457 | 885.456 |
| 1 | X | 1.49E+08 | 152914764 | IDS,LINC00893,CXorf40A,CXorf40A,HSFX3,HSFX4,MAGEA9,MAGEA9B,HSFX2,HSFX1,TMEM185A,MAGEA11,HSFX1,HSFX2,MAGEA9B,MAGEA9,MAGEA8-AS1,MAGEA8,CXorf40A,HSFX4,CXorf40A,CXorf40B,LINC00894,MIR2114,MAMLD1,MTM1,MTMR1,CD99L2,HMGB3,MIR4330,GPR50-AS1,GPR50,GPR50,VMA21,PASD1,PRRG3,FATE1,CNGA2,MAGEA4,GABRE,GABRE,MIR224,GABRE,MIR452,MAGEA5,MAGEA10-MAGEA5,MAGEA10-MAGEA5,MAGEA10,GABRA3,GABRA3,MIR105-1,GABRA3,MIR767,GABRA3,MIR105-2,GABRQ,MAGEA6,CSAG3,MAGEA2,MAGEA2B,CSAG4,MAGEA12,CSAG1,MAGEA3,CETN2,NSDHL,ZNF185,PNMA5,PNMA3,MAGEA1,ZNF275,ZFP92,TREX2,HAUS7,BGN,ATP2B3,CCNQ,DUSP9 | 0.250575 | 372.469 | 471 | 281.002 |
| 1 | 9 | 1.15E+08 | 123785816 | HSDL2,C9orf147,C9orf147,C9orf147,KIAA1958,KIAA1958,INIP,SNX30,SLC46A2,ZNF883,ZFP37,FAM225B,FAM225A,SLC31A2,FKBP15,SLC31A1,CDC26,PRPF4,RNF183,WDR31,BSPRY,HDHD3,ALAD,POLE3,C9orf43,RGS3,ZNF618,AMBP,KIF12,COL27A1,COL27A1,MIR455,ORM1,ORM2,AKNA,WHRN,ATP6V1G1,TMEM268,TEX48,TNFSF15,TNFSF8,TNC,DEC1,PAPPA,PAPPA,PAPPA-AS1,ASTN2,ASTN2,TRIM32,ASTN2,SNORA70C,TLR4,BRINP1,MIR147A,CDK5RAP2,MEGF9,FBXW2,PSMD5,CUTALP,PHF19,TRAF1,C5-OT1,C5,C5 | 0.250298 | 321.599 | 805 | 488.747 |

| | | | | | | | | |
|---|---|---|---|---|---|---|---|---|
| 1 | 9 | 88937271 | 95048136 | ZCCHC6,GAS1,C9orf170,DAPK1,CTSL,CTSL3P,CTSLP8,LOC392364,LOC497256,SPATA31E1,LOC497256,SPATA31C1,CDK20,SPATA31C2,SPIN1,NXNL2,MIR4289,C9orf47,C9orf47,S1PR3,S1PR3,SHC3,CKS2,CKS2,MIR3153,SECISBP2,SEMA4D,GADD45G,MIR4290HG,MIR4290,DIRAS2,SYK,AUH,NFIL3,MIR3910-1,MIR3910-2,ROR2,SPTLC1,LINC00475,PRSS47,IARS | 0.247519 | 345.656 | 389 | 234.485 |
| 1 | 11 | 92085773 | 92715427 | FAT3,MTNR1B | 0.24671 | 389.125 | 75 | 50.2253 |
| 1 | 1 | 1.62E+08 | 173494116 | SH2D1B,UHMK1,UAP1,DDR2,HSD17B7,CCDC190,RGS4,RGS5,RGS5,LOC101928404,NUF2,PBX1,LMX1A,RXRG,LOC400794,LRRC52,MGST3,ALDH9A1,TMCO1,UCK2,UCK2,MIR3658,FAM78B,FAM78B,MIR921,FMO9P,POGK,TADA1,ILDR2,MAEL,GPA33,DUSP27,POU2F1,CD247,CREG1,RCSD1,MPZL1,ADCY10,MPC2,MPC2,DCAF6,DCAF6,DCAF6,MIR1255B2,GPR161,TIPRL,SFT2D2,ANKRD36BP1,TBX19,MIR557,XCL2,XCL1,DPT,ATP1B1,ATP1B1,NME7,NME7,BLZF1,BLZF1,CCDC181,CCDC181,SLC19A2,F5,SELP,SELL,SELE,METTL18,C1orf112,SCYL3,KIFAP3,METTL11B,METTL11B,MIR3119-1,MIR3119-2,LOC101928650,GORAB,GORAB,PRRX1,MROH9,FMO3,FMO3,MIR1295A,MIR1295B,FMO6P,FMO2,FMO1,FMO4,PRRC2C,MYOC,VAMP4,METTL13,DNM3,DNM3,DNM3OS,MIR214,MIR3120,DNM3,DNM3OS,MIR214,DNM3,DNM3OS,MIR199A2,C1orf105,C1orf105,PIGC,SUCO,FASLG,TNFSF18,TNFSF4,PRDX6,SLC9C2 | 0.24668 | 353.97 | 1042 | 621.484 |
| 1 | 11 | 72361966 | 73118793 | PDE2A,ARAP1,ARAP1,ARAP1-AS2,STARD10,STARD10,MIR4692,ATG16L2,FCHSD2,FCHSD2,MIR4459,P2RY2,P2RY6,LOC100287837,ARHGEF17,ARHGEF17,RELT,FAM168A | 0.246068 | 332.762 | 165 | 103.577 |
| 1 | 1 | 2.45E+08 | 247027474 | HNRNPU,LOC101928068,EFCAB2,EFCAB2,KIF26B,SMYD3,TFB2M,CNST,SCCPDH,AHCTF1 | 0.242513 | 349.802 | 170 | 102.408 |
| 1 | 2 | 1.29E+08 | 133543311 | UGGT1,HS6ST1,RAB6C-AS1,RAB6C,RAB6C,CCDC74B,SMPD4,SMPD4,MZT2B,MZT2B,TUBA3E,CCDC115,CCDC115,IMP4,IMP4,PTPN18,CFC1B,TISP43,LOC646743,LOC646743,TISP43,CFC1,CFC1B,GPR148,AMER3,ARHGEF4,FAM168B,PLEKHB2,RAB6D,NOC2LP2,TUBA3D,MZT2A,MZT2A,MIR4784,LOC150776,CCDC74A,C2orf27A,C2orf27B,ANKRD30BL,MIR663B,ZNF806,GPR39,GPR39,LYPD1,LYPD1,NCKAP5 | 0.240332 | 286.63 | 366 | 203.639 |
| 1 | 5 | 1.76E+08 | 177612228 | RNF44,CDHR2,GPRIN1,SNCB,SNCB,MIR4281,EIF4E1B,TSPAN17,UNC5A,HK3,UIMC1,ZNF346,FGFR4,NSD1,RAB24,PRELID1,PRELID1,MXD3,MXD3,LMAN2,RGS14,SLC34A1,PFN3,F12,GRK6,GRK6,PRR7-AS1,PRR7,DBN1,PDLIM7,DOK3,DDX41,FAM193B,TMED9,B4GALT7,LOC202181,FAM153A,LOC728554,PROP1,FAM153C,N4BP3,RMND5B,NHP2 | 0.233546 | 338.373 | 462 | 286.439 |

| | | | | | | | | |
|---|---|---|---|---|---|---|---|---|
| 1 | 20 | 68259 | 6759756 | DEFB125,DEFB126,DEFB127,DEFB128,DEFB129,DEFB132,C20orf96,ZCCHC3,SOX12,NRSN2,TRIB3,RBCK1,TBC1D20,CSNK2A1,TCF15,SRXN1,SCRT2,SLC52A3,FAM110A,ANGPT4,RSPO4,PSMF1,TMEM74B,C20orf202,RAD21L1,SNPH,SDCBP2,FKBP1A-SDCBP2,FKBP1A-SDCBP2,SDCBP2-AS1,FKBP1A-SDCBP2,SDCBP2-AS1,FKBP1A,FKBP1A-SDCBP2,FKBP1A,MIR6869,NSFL1C,SIRPB2,SIRPD,SIRPB1,SIRPG,SIRPG,SIRPG-AS1,SIRPA,PDYN-AS1,PDYN,STK35,TGM3,TGM6,SNRPB,SNRPB,SNORD119,ZNF343,TMC2,NOP56,NOP56,MIR1292,NOP56,SNORD110,NOP56,SNORA51,NOP56,SNORD86,NOP56,SNORD56,NOP56,SNORD57,NOP56,IDH3B,IDH3B,EBF4,CPXM1,C20orf141,TMEM239,PCED1A,PCED1A,VPS16,VPS16,VPS16,PTPRA,PTPRA,GNRH2,MRPS26,OXT,AVP,UBOX5-AS1,UBOX5,UBOX5-AS1,UBOX5,FASTKD5,LZTS3,DDRGK1,ITPA,SLC4A11,C20orf194,ATRN,GFRA4,ADAM33,SIGLEC1,HSPA12B,C20orf27,SPEF1,CENPB,CDC25B,AP5S1,MAVS,PANK2,PANK2,MIR103A2,MIR103B2,RNF24,SMOX,ADRA1D,PRNP,PRND,PRNT,RASSF2,SLC23A2,TMEM230,PCNA,PCNA,PCNA-AS1,CDS2,PROKR2,LOC643406,LINC00654,GPCPD1,C20orf196,CHGB,TRMT6,MCM8,MCM8,MCM8-AS1,CRLS1,LRRN4,FERMT1,BMP2 | 0.233383 | 350.883 | 1039 | 631.69 |
| 1 | 12 | 1.19E+08 | 121871062 | SUDS3,SRRM4,HSPB8,LINC00934,CCDC60,TMEM233,PRKAB1,CIT,CIT,MIR1178,BICDL1,RAB35,GCN1,GCN1,MIR4498,RPLP0,PXN-AS1,PXN,PXN,SIRT4,PLA2G1B,MSI1,COX6A1,TRIAP1,TRIAP1,GATC,GATC,GATC,SRSF9,SRSF9,DYNLL1,COQ5,RNF10,POP5,CABP1,MLEC,UNC119B,UNC119B,MIR4700,ACADS,SPPL3,HNF1A,HNF1A,C12orf43,C12orf43,OASL,P2RX7,P2RX4,CAMKK2,ANAPC5,RNF34,KDM2B | 0.231894 | 342.115 | 492 | 299.985 |
| 1 | 1 | 2.29E+08 | 244541975 | RAB4A,RAB4A,SPHAR,CCSAP,ACTA1,NUP133,ABCB10,TAF5L,URB2,GALNT2,PGBD5,COG2,AGT,CAPN9,C1orf198,TTC13,ARV1,FAM89A,MIR1182,FAM89A,TRIM67,C1orf131,GNPAT,EXOC8,SPRTN,EGLN1,TSNAX,TSNAX-DISC1,TSNAX-DISC1,TSNAX-DISC1,DISC1,TSNAX-DISC1,DISC1,DISC2,SIPA1L2,MAP10,NTPCR,PCNX2,MAP3K21,KCNK1,KCNK1,MIR4427,SLC35F3,SLC35F3,MIR4671,LOC101927765,COA6,COA6,TARBP1,IRF2BP2,TOMM20,TOMM20,SNORA14B,RBM34,ARID4B,ARID4B,MIR4753,GGPS1,TBCE,TBCE,B3GALNT2,B3GALNT2,MIR5096,GNG4,MIR5096,LYST,LYST,LYST,MIR1537,NID1,GPR137B,ERO1B,EDARADD,LGALS8,LGALS8-AS1,LGALS8,LGALS8,HEATR1,HEATR1,ACTN2,MTR,RYR2,RYR2,MIR4428,LOC100130331,ZP4,CHRM3,FMN2,MIR1273E,MIR1273E,GREM2,RGS7,RGS7,MIR3123,FH,KMO,KMO,OPN3,OPN3,OPN3,CHML,WDR64,EXO1,MAP1LC3C,PLD5,CEP170,SDCCAG8,SDCCAG8,MIR4677,SDCCAG8,AKT3,AKT3,ZBTB18,C1orf100 | 0.231353 | 352.773 | 1422 | 851.595 |

| | | | | | | | | |
|---|---|---|---|---|---|---|---|---|
| 1 | 16 | 10574694 | 11857517 | ATF7IP2,EMP2,TEKT5,NUBP1,NUBP1,TVP23A,TVP23A,CIITA,DEXI,CLEC16A,SOCS1,TNP2,PRM3,PRM2,PRM1,RMI2,LITAF,SNN,TXNDC11,ZC3H7A | 0.230777 | 320.716 | 182 | 107.611 |
| 1 | 21 | 42869951 | 48119395 | TMPRSS2,LINC00479,RIPK4,RIPK4,MIR6814,PRDM15,C2CD2,ZBTB21,ZNF295-AS1,UMODL1,UMODL1,UMODL1-AS1,ABCG1,TFF3,TFF2,TFF1,TMPRSS3,UBASH3A,RSPH1,SLC37A1,PDE9A,WDR4,NDUFV3,ERVH48-1,MIR5692B,PKNOX1,CBS,CBSL,CBSL,CBS,U2AF1,U2AF1L5,CRYAA,CRYAA2,SIK1B,SIK1,HSF2BP,HSF2BP,H2BFS,RRP1B,PDXK,CSTB,RRP1,AGPAT3,TRAPPC10,PWP2,C21orf33,ICOSLG,DNMT3L,DNMT3L,LOC105372833,AIRE,PFKL,C21orf2,TRPM2,TRPM2,TRPM2-AS,LRRC3,TSPEAR,TSPEAR,TSPEAR-AS1,TSPEAR,TSPEAR-AS2,TSPEAR,KRTAP10-1,TSPEAR,KRTAP10-2,TSPEAR,KRTAP10-3,TSPEAR,KRTAP10-4,TSPEAR,KRTAP10-5,TSPEAR,KRTAP10-6,TSPEAR,KRTAP10-7,TSPEAR,KRTAP10-8,TSPEAR,KRTAP10-9,TSPEAR,KRTAP10-10,TSPEAR,KRTAP10-11,TSPEAR,KRTAP12-4,TSPEAR,KRTAP12-3,TSPEAR,KRTAP12-2,TSPEAR,KRTAP12-1,TSPEAR,KRTAP10-12,UBE2G2,SUMO3,PTTG1IP,ITGB2,LINC01547,FAM207A,ADARB1,POFUT2,COL18A1,COL18A1,COL18A1-AS1,COL18A1,MIR6815,SLC19A1,PCBP3,COL6A1,COL6A2,FTCD,FTCD,FTCD-AS1,SPATC1L,LSS,MCM3AP-AS1,MCM3AP-AS1,MCM3AP,MCM3AP,YBEY,C21orf58,PCNT,DIP2A,S100B,PRMT2 | 0.228157 | 351.65 | 1146 | 689.398 |
| 1 | 16 | 56503821 | 58741650 | OGFOD1,BBS2,MT4,MT3,MT2A,MT1E,MT1M,MT1A,MT1B,MT1F,MT1G,MT1H,MT1IP,MT1X,NUP93,MIR138-2,SLC12A3,SLC12A3,MIR6863,HERPUD1,CETP,NLRC5,CPNE2,FAM192A,RSPRY1,ARL2BP,PLLP,CCL22,CX3CL1,CCL17,CIAPIN1,CIAPIN1,COQ9,COQ9,POLR2C,DOK4,CCDC102A,ADGRG5,ADGRG1,ADGRG3,DRC7,KATNB1,KIFC3,KIFC3,MIR6772,LOC388282,CNGB1,TEPP,ZNF319,USB1,MMP15,CFAP20,CSNK2A2,CCDC113,CCDC113,PRSS54,PRSS54,GINS3,NDRG4,SETD6,CNOT1,CNOT1,SNORA46,CNOT1,SNORA50A,SLC38A7 | 0.227519 | 361.827 | 665 | 410.64 |
| 1 | 15 | 43813472 | 44127389 | MAP1A,PPIP5K1,CKMT1B,STRC,CATSPER2,PPIP5K1P1-CATSPER2,PPIP5K1P1-CATSPER2,CKMT1A,STRCP1,CATSPER2P1,PDIA3,ELL3,ELL3,SERF2,SERF2,SERF2-C15ORF63,SERF2,SERF2-C15ORF63,MIR1282,SERF2,SERF2-C15ORF63,SERINC4,SERF2-C15ORF63,SERINC4,SERF2-C15ORF63,HYPK,MFAP1,WDR76 | 0.225685 | 355.508 | 302 | 174.733 |
| 1 | 19 | 48547101 | 48989168 | CABP5,PLA2G4C,PLA2G4C,PLA2G4C-AS1,LIG1,ZSWIM9,CARD8,ZNF114,CCDC114,EMP3,TMEM143,SYNGR4,KDELR1,GRIN2D,GRWD1,KCNJ14,CYTH2,LMTK3 | 0.223511 | 345.812 | 172 | 107.579 |

| | | | | | | | | |
|---|---|---|---|---|---|---|---|---|
| 1 | 4 | 1.87E+08 | 191025802 | SORBS2,TLR3,FAM149A,CYP4V2,KLKB1,F11,F11,F11-AS1,F11-AS1,MTNR1A,FAT1,ZFP42,TRIML2,TRIML1,FRG1,FRG2 | 0.221682 | 354.517 | 265 | 164.928 |
| 1 | 12 | 7351545 | 9440739 | PEX5,ACSM4,CD163L1,CD163,APOBEC1,GDF3,DPPA3,CLEC4C,NANOGNB,NANOG,SLC2A14,SLC2A3,FOXJ2,C3AR1,NECAP1,CLEC4A,CLEC4A,POU5F1P3,FAM90A1,LINC00937,CLEC6A,CLEC4D,CLEC4E,AICDA,MFAP5,RIMKLB,A2ML1,PHC1,PHC1,M6PR,M6PR,KLRG1,LINC00612,A2M-AS1,A2M,A2M,PZP,LOC642846 | 0.219359 | 360.169 | 424 | 248.098 |
| 1 | 9 | 1.03E+08 | 104500268 | ERP44,INVS,INVS,TEX10,TEX10,MSANTD3,MSANTD3-TMEFF1,MSANTD3-TMEFF1,TMEFF1,CAVIN4,PLPPR1,BAAT,MRPL50,ZNF189,ALDOB,TMEM246-AS1,TMEM246,RNF20,GRIN3A,GRIN3A,PPP3R2 | 0.21505 | 323.941 | 168 | 102.227 |
| 1 | 13 | 30525133 | 32363377 | KATNAL1,HMGB1,USPL1,ALOX5AP,TEX26-AS1,MEDAG,TEX26,HSPH1,B3GLCT,RXFP2 | 0.213479 | 342.005 | 123 | 73.0034 |
| 1 | X | 62042080 | 72783463 | SPIN4,ARHGEF9,MIR1468,AMER1,ASB12,MTMR8,ZC4H2,ZC3H12B,LAS1L,FRMD8P1,MSN,MIR223,VSIG4,HEPH,EDA2R,AR,OPHN1,YIPF6,STARD8,EFNB1,PJA1,LINC00269,FAM155B,EDA,EDA,MIR676,AWAT2,OTUD6A,IGBP1,DGAT2L6,AWAT1,P2RY4,ARR3,RAB41,PDZD11,KIF4A,GDPD2,DLG3,DLG3,DLG3-AS1,TEX11,SLC7A3,SNX12,FOXO4,CXorf65,IL2RG,MED12,NLGN3,BCYRN1,GJB1,BCYRN1,ZMYM3,BCYRN1,NONO,BCYRN1,ITGB1BP2,BCYRN1,BCYRN1,TAF1,BCYRN1,OGT,BCYRN1,GCNA,BCYRN1,CXCR3,BCYRN1,CXorf49B,CXorf49,CXorf49B,CXorf49,NHSL2,NHSL2,RTL5,FLJ44635,PIN4,PIN4,ERCC6L,RPS4X,CITED1,HDAC8,PHKA1,PHKA1,PHKA1-AS1,FAM236B,FAM236A,DMRTC1,DMRTC1B,FAM226B,FAM226A,PABPC1L2B,PABPC1L2A,NAP1L6,NAP1L2,CDX4,CHIC1 | 0.204384 | 331.39 | 1000 | 597.37 |
| 1 | 9 | 99525320 | 102676957 | ZNF510,ZNF782,NUTM2G,MFSD14C,CTSV,ANKRD18CP,LOC100499484,LOC100499484-C9ORF174,LOC100499484-C9ORF174,LOC100499484-C9ORF174,CCDC180,LOC100499484-C9ORF174,CCDC180,MIR1302-8,TDRD7,TMOD1,TMOD1,TSTD2,TSTD2,NCBP1,XPA,FOXE1,TRMO,HEMGN,ANP32B,NANS,TRIM14,CORO2A,TBC1D2,TBC1D2,MIR6854,GABBR2,ANKS6,GALNT12,COL15A1,TGFBR1,ALG2,SEC61B,NR4A3 | 0.203209 | 325.329 | 428 | 255.296 |

| | | | | | | | | |
|---|---|---|---|---|---|---|---|---|
| 1 | 12 | 1.12E+08 | 118650185 | ATXN2,BRAP,ACAD10,ALDH2,ALDH2,MIR6761,MAPKAPK5-AS1,MAPKAPK5,MAPKAPK5,ADAM1A,TMEM116,ERP29,NAA25,NAA25,MIR3657,TRAFD1,HECTD4,HECTD4,MIR6861,RPL6,PTPN11,RPH3A,MIR1302-1,RPH3A,OAS1,OAS3,OAS2,DTX1,RASAL1,CFAP73,CFAP73,DDX54,CFAP73,DDX54,MIR7106,DDX54,RITA1,IQCD,TPCN1,TPCN1,MIR6762,SLC8B1,PLBD2,SDS,SDSL,LHX5,RBM19,TBX5,TBX3,MED13L,MED13L,MIR620,LINC00173,MAP1LC3B2,C12orf49,RNFT2,HRK,FBXW8,FBXW8,LOC100506551,TESC,FBXO21,NOS1,KSR2,RFC5,WSB2,VSIG10,PEBP1,TAOK3 | 0.203006 | 326.096 | 835 | 504.562 |
| 1 | 12 | 1.06E+08 | 110566921 | APPL2,C12orf75,NUAK1,CKAP4,TCP11L2,POLR3B,POLR3B,LOC100287944,LOC100287944,RFX4,LOC100287944,RFX4,LOC100505978,LOC100287944,RIC8B,RIC8B,TMEM263,MTERF2,CRY1,BTBD11,PWP1,PRDM4,PRDM4,LOC101929162,ASCL4,WSCD2,CMKLR1,FICD,SART3,ISCU,TMEM119,SELPLG,MIR4496,CORO1C,SSH1,SSH1,MIR619,DAO,SVOP,USP30,USP30-AS1,USP30,ALKBH2,UNG,ACACB,FOXN4,MYO1H,KCTD10,UBE3B,MMAB,MVK,FAM222A,FAM222A-AS1,TRPV4,TRPV4,MIR4497,GLTP,TCHP,GIT2,ANKRD13A,C12orf76,IFT81 | 0.201892 | 325.982 | 709 | 430.356 |
| 1 | 21 | 38567913 | 40574470 | TTC3,DSCR9,DSCR3,DYRK1A,KCNJ6,DSCR4,DSCR8,DSCR10,KCNJ15,ERG,ETS2,LOC400867,PSMG1,BRWD1 | 0.198694 | 318.574 | 128 | 78.0678 |
| 1 | 11 | 71164316 | 72145522 | NADSYN1,KRTAP5-7,KRTAP5-8,KRTAP5-9,KRTAP5-10,KRTAP5-11,FAM86C1,ALG1L9P,LOC100133315,RNF121,IL18BP,NUMA1,NUMA1,LOC100128494,NUMA1,MIR3165,LRTOMT,LRTOMT,LAMTOR1,LRTOMT,ANAPC15,ANAPC15,FOLR3,FOLR1,FOLR2,INPPL1,PHOX2A,CLPB | 0.198363 | 323.43 | 220 | 131.262 |
| 1 | 12 | 1.22E+08 | 122958727 | HPD,PSMD9,WDR66,BCL7A,MLXIP,LRRC43,IL31,LRRC43,B3GNT4,B3GNT4,DIABLO,DIABLO,VPS33A,CLIP1,ZCCHC8 | 0.197737 | 359.135 | 158 | 98.1249 |
| 1 | X | 99551245 | 100265030 | PCDH19,TNMD,TSPAN6,SRPX2,SYTL4,CSTF2,NOX1,XKRX,ARL13A | 0.196987 | 350.009 | 111 | 68.9203 |
| 1 | 16 | 64981519 | 66789025 | CDH11,LINC00922,CDH5,BEAN1,TK2,CKLF,CKLF-CMTM1,CKLF-CMTM1,CMTM1,CMTM2,CMTM3,CMTM4,DYNC1LI2,TERB1 | 0.194714 | 310.388 | 118 | 70.7308 |
| 1 | 11 | 1.19E+08 | 120096521 | DDX6,CXCR5,BCL9L,BCL9L,MIR4492,UPK2,FOXR1,CCDC84,RPS25,TRAPPC4,MIR3656,TRAPPC4,SLC37A4,HYOU1,VPS11,HMBS,H2AFX,DPAGT1,C2CD2L,HINFP,ABCG4,NLRX1,PDZD3,CCDC153,CBL,MCAM,MCAM,MIR6756,RNF26,C1QTNF5,MFRP,MFRP,C1QTNF5,USP2,USP2-AS1,USP2-AS1,THY1,NECTIN1,TRIM29,OAF | 0.192944 | 327.839 | 349 | 224.186 |

| | | | | | | | | |
|---|---|---|---|---|---|---|---|---|
| 1 | 9 | 95274304 | 99286090 | CENPP,ECM2,CENPP,ECM2,MIR4670,CENPP,CENPP,IPPK,IPPK,IPPK,LOC100128361,BICD2,ANKRD19P,ZNF484,FGD3,FGD3,LOC101927954,SUSD3,CARD19,NINJ1,WNK2,WNK2,C9orf129,C9orf129,FAM120AOS,FAM120AOS,FAM120A,FAM120A,PHF2,PHF2,MIR548AU,MIR4291,BARX1,PTPDC1,MIRLET7A1,MIRLET7F1,MIRLET7DHG,MIRLET7D,ZNF169,NUTM2F,MFSD14B,PCAT7,FBP2,FBP2,FBP1,C9orf3,C9orf3,MIR2278,C9orf3,LOC101928119,C9orf3,MIR23B,MIR27B,C9orf3,MIR3074,MIR24-1,FANCC,PTCH1,PTCH1,LOC100507346,ERCC6L2,LINC00092,LOC158434,HSD17B3,HSD17B3,HSD17B3-AS1,SLC35D2,ZNF367,HABP4,CDC14B | 0.188998 | 308.683 | 521 | 305.15 |
| 1 | 8 | 1.26E+08 | 130853848 | TATDN1,NDUFB9,NDUFB9,MTSS1,MIR4662B,MIR4662A,ZNF572,SQLE,WASHC5,NSMCE2,TRIB1,FAM84B,PRNCR1,CASC8,POU5F1B,MYC,PVT1,MIR1204,PVT1,TMEM75,PVT1,MIR1205,PVT1,MIR1206,PVT1,MIR1207,MIR1208,CCDC26,MIR3686,GSDMC | 0.182223 | 281.052 | 179 | 106.31 |
| 1 | 4 | 52660617 | 55956344 | DCUN1D4,LRRC66,SGCB,SPATA18,USP46,DANCR,MIR4449,DANCR,SNORA26,ERVMER34-1,RASL11B,SCFD2,FIP1L1,LNX1,LNX1,LNX1-AS1,RPL21P44,CHIC2,GSX2,PDGFRA,KIT,KDR | 0.180231 | 312.173 | 236 | 142.41 |
| 1 | 17 | 57812662 | 58761478 | VMP1,VMP1,MIR21,TUBD1,RPS6KB1,RNFT1,RNFT1,TBC1D3P1-DHX40P1,TBC1D3P1-DHX40P1,RNFT1-DT,TBC1D3P1-DHX40P1,MIR4737,HEATR6,HEATR6,LOC653653,CA4,USP32,USP32,SCARNA20,C17orf64,APPBP2,PPM1D,BCAS3 | 0.171333 | 367.84 | 197 | 110.185 |
| 1 | 16 | 68349789 | 72821222 | PRMT7,SMPD3,ZFP90,CDH3,CDH1,TANGO6,HAS3,HAS3,CHTF8,CHTF8,CHTF8,UTP4,UTP4,SNTB2,VPS4A,PDF,COG8,COG8,COG8,NIP7,NIP7,TMED6,TERF2,CYB5B,MIR1538,NFAT5,NQO1,NOB1,WWP2,WWP2,MIR140,CLEC18A,PDXDC2P-NPIPB14P,PDXDC2P-NPIPB14P,MIR1972-1,MIR1972-2,PDPR,PDPR,LOC400541,CLEC18C,EXOSC6,AARS,DDX19B,DDX19B,LOC100506083,DDX19A,ST3GAL2,FUK,COG4,SF3B3,SF3B3,SNORD111B,SF3B3,SNORD111,IL34,MTSS1L,VAC14,VAC14,VAC14-AS1,HYDIN,CMTR2,CALB2,ZNF23,ZNF19,CHST4,TAT-AS1,TAT,TAT,MARVELD3,PHLPP2,PHLPP2,SNORA70D,AP1G1,AP1G1,SNORD71,ATXN1L,ZNF821,IST1,IST1,PKD1L3,PKD1L3,DHODH,HP,HPR,TXNL4B,DHX38,PMFBP1,ZFHX3 | 0.170474 | 338.025 | 990 | 596.894 |
| 1 | 15 | 42867891 | 43109384 | STARD9,CDAN1,TTBK2 | 0.161408 | 313.807 | 131 | 86.6047 |

| | | | | | | | | |
|---|---|---|---|---|---|---|---|---|
| 1 | 2 | 74005381 | 75874029 | DUSP11,C2orf78,STAMBP,ACTG2,DGUOK,DGUOK,DGUOK-AS1,TET3,BOLA3,MOB1A,MTHFD2,SLC4A5,DCTN1,DCTN1,DCTN1-AS1,C2orf81,WDR54,RTKN,INO80B,INO80B-WBP1,INO80B-WBP1,WBP1,MOGS,MRPL53,CCDC142,CCDC142,TTC31,TTC31,LBX2,LBX2,LBX2-AS1,PCGF1,TLX2,DQX1,AUP1,AUP1,HTRA2,HTRA2,HTRA2,LOXL3,LOXL3,LOXL3,DOK1,DOK1,M1AP,SEMA4F,HK2,POLE4,TACR1,TACR1,MIR5000,EVA1A,MRPL19 | 0.160964 | 323.461 | 407 | 255.371 |
| 1 | 8 | 32406154 | 38965410 | NRG1,FUT10,MAK16,MAK16,TTI2,TTI2,RNF122,DUSP26,UNC5D,UNC5D,LOC101929550,KCNU1,LINC01605,ZNF703,LOC102723701,ERLIN2,ERLIN2,LOC728024,PLPBP,ADGRA2,BRF2,RAB11FIP1,GOT1L1,ADRB3,EIF4EBP1,ASH2L,STAR,LSM1,BAG4,DDHD2,PLPP5,NSD3,LETM2,FGFR1,C8orf86,RNF5P1,TACC1,PLEKHA2,HTRA4,TM2D2,ADAM9,ADAM32 | 0.159002 | 319.76 | 528 | 318.46 |
| 1 | 7 | 99473903 | 102517422 | OR2AE1,TRIM4,GJC3,AZGP1,AZGP1P1,ZKSCAN1,ZSCAN21,ZSCAN21,ZNF3,ZNF3,COPS6,MCM7,MCM7,MIR25,MIR93,MCM7,MIR106B,MCM7,AP4M1,AP4M1,TAF6,TAF6,CNPY4,CNPY4,MBLAC1,LAMTOR4,C7orf43,C7orf43,MIR4658,GAL3ST4,GPC2,STAG3,STAG3,GATS,GATS,GATS,PVRIG,SPDYE3,STAG3L5P-PVRIG2P-PILRB,PVRIG2P,STAG3L5P-PVRIG2P-PILRB,MIR6840,STAG3L5P-PVRIG2P-PILRB,PILRB,PILRA,ZCWPW1,MEPCE,PPP1R35,C7orf61,TSC22D4,NYAP1,AGFG2,SAP25,LRCH4,ZASP,LRCH4,FBXO24,FBXO24,PCOLCE-AS1,PCOLCE-AS1,PCOLCE,PCOLCE,MOSPD3,TFR2,ACTL6B,GNB2,GIGYF1,POP7,EPO,ZAN,EPHB4,SLC12A9,TRIP6,TRIP6,MIR6875,SRRT,SRRT,UFSP1,UFSP1,ACHE,MUC3A,MUC12,MUC12,LOC102724094,MUC17,TRIM56,SERPINE1,AP1S1,AP1S1,MIR4653,VGF,NAT16,MOGAT3,PLOD3,ZNHIT1,CLDN15,FIS1,LOC101927746,IFT22,COL26A1,MYL10,CUX1,SH2B2,SH2B2,MIR4285,LOC100289561,LOC100630923,LOC100630923,LOC100630923,PRKRIP1,LOC100630923,PRKRIP1,MIR548O,ORAI2,ALKBH4,LRWD1,LRWD1,MIR5090,LRWD1,MIR4467,POLR2J,RASA4B,POLR2J3,RASA4,RASA4B,POLR2J2,UPK3BL1,POLR2J2,RASA4DP,FAM185A,FAM185A,FBXL13 | 0.157322 | 361.431 | 931 | 562.009 |

| | | | | | | | |
|---|---|---|---|---|---|---|---|
| 1 | 16 | 11992404 | 28332251 | GSPT1,NPIPB2,TNFRSF17,SNX29,CPPED1,CPPED1,MIR4718,SHISA9,ERCC4,MKL2,MIR193BHG,MIR193B,MIR193BHG,MIR365A,PARN,BFAR,PLA2G10,ABCC6P2,NOMO1,PDXDC1,PDXDC1,NTAN1,PDXDC1,RRN3,MPV17L,C16orf45,MARF1,MARF1,MIR6506,NDE1,MIR484,NDE1,NDE1,MYH11,MYH11,FOPNL,ABCC1,ABCC6,NOMO3,PKD1P4-NPIPA8,XYLT1,XYLT1,LOC102723692,PKD1P5-LOC105376752,NOMO2,ABCC6P1,RPS15A,ARL6IP1,SMG1,TMC7,LOC102723385,COQ7,COQ7,ITPRIPL2,SYT17,CLEC19A,TMC5,GDE1,CCP110,C16orf62,KNOP1,IQCK,GPRC5B,GPR139,GP2,UMOD,PDILT,ACSM5,ACSM2A,ACSM2B,ACSM1,THUMPD1,ACSM3,ACSM3,ERI2,ERI2,ERI2,REXO5,REXO5,DCUN1D3,LYRM1,DNAH3,DNAH3,TMEM159,TMEM159,ZP2,ANKS4B,CRYM,CRYM-AS1,SNX29P1,MIR3680-2,MIR3680-1,SLC7A5P2,LOC101927814,METTL9,METTL9,METTL9,IGSF6,OTOA,RRN3P1,UQCRC2,PDZD9,C16orf52,VWA3A,EEF2K,POLR3E,CDR2,RRN3P3,LOC653786,HS3ST2,USP31,SCNN1G,SCNN1B,COG7,GGA2,EARS2,UBFD1,NDUFAB1,PALB2,DCTN5,PLK1,ERN2,CHP2,PRKCB,CACNG3,RBBP6,TNRC6A,LINC01567,TNRC6A,SLC5A11,ARHGAP17,LOC554206,LCMT1,AQP8,ZKSCAN2,HS3ST4,HS3ST4,MIR548W,C16orf82,KDM8,NSMCE1,FLJ21408,IL4R,IL21R,IL21R,IL21R-AS1,GTF3C1,KIAA0556,KIAA0556,LOC100128079,GSG1L,XPO6,SBK1 | 0.1571 | 330.229 | 1954 | 1169.77 |
| 1 | 5 | 43556775 | 45907753 | PAIP1,NNT,FGF10,MRPS30,HCN1 | 0.154998 | 294.376 | 71 | 43.1263 |
| 1 | 15 | 40324360 | 40894484 | EIF2AK4,SRP14,BMF,BUB1B,BUB1B,BUB1B-PAK6,BUB1B-PAK6,PAK6,BUB1B-PAK6,PAK6,C15orf56,ANKRD63,PLCB2,CCDC9B,PHGR1,DISP2,KNSTRN,IVD,BAHD1,CHST14,CCDC32,MRPL42P5,CCDC32,RPUSD2 | 0.152134 | 329.993 | 205 | 130.505 |
| 1 | X | 1.06E+08 | 112211890 | TBC1D8B,RIPPLY1,CLDN2,CLDN2,MORC4,RBM41,NUP62CL,PIH1D3,FRMPD3-AS1,FRMPD3,FRMPD3,PRPS1,TSC22D3,NCBP2L,MID2,MID2,LOC101928335,TEX13B,VSIG1,PSMD10,ATG4A,COL4A6,COL4A5,IRS4,GUCY2F,NXT2,KCNE5,ACSL4,TMEM164,TMEM164,MIR652,TMEM164,MIR3978,AMMECR1,AMMECR1,SNORD96B,RTL9,TDGF1P3,CHRDL1,PAK3,CAPN6,DCX,ALG13,TRPC5,TRPC5,TRPC5OS,RTL4,LHFPL1,AMOT,AMOT,MIR4329 | 0.151627 | 302.42 | 585 | 345.874 |

| | | | | | | | |
|---|---|---|---|---|---|---|---|
| 1 | 11 | 55035775 | 68855424 | TRIM48,OR4A16,OR4A15,OR4C15,OR4C16,OR4C11,OR4P4,OR4S2,OR4C6,OR5D13,OR5D14,OR5L1,OR5D18,OR5L2,OR5D16,TRIM51,OR5W2,OR5I1,OR10AG1,OR7E5P,OR5F1,OR5AS1,OR8I2,OR8H2,OR8H3,OR8J3,OR8K5,OR5J2,OR5T2,OR5T3,OR5T1,OR8H1,OR8K3,OR8K1,OR8J1,OR8U8,OR8U1,OR5R1,OR5M9,OR5M3,OR5M8,OR5M11,OR5M10,OR5M1,OR5AP2,OR5AR1,OR9G9,OR9G1,OR9G4,OR5AK2,OR5AK4P,LRRC55,APLNR,TNKS1BP1,SSRP1,P2RX3,PRG3,PRG2,SLC43A3,RTN4RL2,SLC43A1,TIMM10,SMTNL1,UBE2L6,SERPING1,MIR130A,YPEL4,CLP1,ZDHHC5,MED19,TMX2,TMX2-CTNND1,TMX2-CTNND1,SELENOH,TMX2-CTNND1,BTBD18,TMX2-CTNND1,CTNND1,OR9Q1,OR6Q1,OR9Q1,OR9Q1,OR9I1,OR9Q2,OR1S2,OR1S1,OR10Q1,OR10W1,OR5B17,OR5B3,OR5B2,OR5B12,OR5B21,LPXN,ZFP91,ZFP91-CNTF,ZFP91-CNTF,CNTF,GLYAT,GLYATL2,GLYATL1,GLYATL1,LOC283194,FAM111B,FAM111A,DTX4,MPEG1,OR5AN1,OR5A2,OR5A1,OR4D6,OR4D10,OR4D11,OR4D9,OSBP,OSBP,MIR3162,PATL1,OR10V1,OR10V2P,STX3,MRPL16,GIF,TCN1,OOSP1,OOSP2,MS4A3,MS4A2,MS4A6A,MS4A4E,MS4A4A,MS4A6E,MS4A7,MS4A14,MS4A5,MS4A1,MS4A12,MS4A13,MS4A8,MS4A18,MS4A15,MS4A10,CCDC86,PTGDR2,ZP1,PRPF19,TMEM109,TMEM132A,SLC15A3,CD6,CD5,VPS37C,PGA3,PGA4,PGA5,VWCE,DDB1,TKFC,TKFC,CYB561A3,CYB561A3,TMEM138,TMEM216,CPSF7,SDHAF2,PPP1R32,MIR4488,LRRC10B,SYT7,RPLP0P2,DAGLA,MYRF,MYRF,DKFZP434K028,TMEM258,TMEM258,MIR611,FEN1,FADS1,FADS1,MIR1908,FADS1,FADS2,FADS2,FADS3,FADS3,MIR6746,RAB3IL1,BEST1,FTH1,INCENP,SCGB1D1,SCGB2A1,SCGB1D2,SCGB2A2,SCGB1D4,ASRGL1,SCGB1A1,AHNAK,EEF1G,EEF1G,MIR3654,EEF1G,MIR6747,TUT1,MTA2,EML3,ROM1,B3 | 0.146914 | 329.842 | 3971 | 2474.27 |
| 1 | 3 | 50677715 | 52570962 | MAPKAPK3,MIR4787,DOCK3,DOCK3,MANF,RBM15B,DCAF1,RAD54L2,TEX264,GRM2,IQCF6,IQCF3,IQCF2,IQCF5-AS1,IQCF5,IQCF1,RRP9,PARP3,GPR62,PCBP4,ABHD14B,ABHD14A,ABHD14A-ACY1,ABHD14A-ACY1,ACY1,RPL29,DUSP7,LINC00696,POC1A,ALAS1,TLR9,TWF2,PPM1M,WDR82,WDR82,MIRLET7G,GLYCTK,GLYCTK,MIR135A1,DNAH1,BAP1,PHF7,SEMA3G,TNNC1,NISCH,STAB1,STAB1,NT5DC2,NT5DC2,SMIM4 | 0.146231 | 332.235 | 606 | 387.034 |
| 1 | 4 | 1.46E+08 | 155508073 | SMAD1,SMAD1-AS1,SMAD1,MMAA,C4orf51,ZNF827,LSM6,REELD1,SLC10A7,POU4F2,TTC29,EDNRA,TMEM184C,PRMT9,ARHGAP10,ARHGAP10,MIR4799,NR3C2,DCLK2,LRBA,LRBA,LOC729558,LRBA,MAB21L2,RPS3A,RPS3A,SNORD73B,RPS3A,SNORD73A,SH3D19,PRSS48,FAM160A1,GATB,FBXW7,FBXW7,FBXW7-AS1,FBXW7,MIR3140,MIR4453HG,MIR4453,TMEM154,TIGD4,ARFIP1,FHDC1,TRIM2,TRIM2,ANXA2P1,MND1,TMEM131L,TLR2,RNF175,SFRP2,DCHS2,PLRG1,FGB,FGA | 0.145737 | 309.711 | 697 | 418.972 |

| | | | | | | | |
|---|---|---|---|---|---|---|---|
| 1 | 8 | 23538995 | 30464017 | NKX3-1,NKX2-6,STC1,ADAM28,ADAM28,LOC101929294,LOC101929294,ADAMDEC1,LOC101929294,ADAM7,LOC101929294,ADAM7,LOC101929315,LOC101929294,NEFM,NEFL,NEFL,MIR6841,DOCK5,DOCK5,MIR6876,GNRH1,KCTD9,CDCA2,EBF2,PPP2R2A,BNIP3L,PNMA2,DPYSL2,ADRA1A,STMN4,TRIM35,PTK2B,CHRNA2,EPHX2,CLU,CLU,MIR6843,SCARA3,MIR3622B,MIR3622A,CCDC25,ESCO2,PBK,SCARA5,SCARA5,MIR4287,NUGGC,ELP3,PNOC,ZNF395,FBXO16,FZD3,FZD3,MIR4288,EXTL3,INTS9,HMBOX1,KIF13B,DUSP4,MIR3148,SARAF,LEPROTL1,LEPROTL1,MBOAT4,MBOAT4,DCTN6,RBPMS-AS1,RBPMS,RBPMS,GTF2E2 | 0.145329 | 320.915 | 736 | 442.797 |
| 1 | 15 | 73925460 | 76136903 | NPTN,CD276,C15orf59,TBC1D21,LOXL1-AS1,LOXL1,LOXL1,STOML1,PML,LOC283731,ISLR2,ISLR,STRA6,CCDC33,CYP11A1,SEMA7A,SEMA7A,MIR6881,UBL7,ARID3B,CLK3,EDC3,CYP1A1,CYP1A2,CSK,MIR4513,CSK,LMAN1L,CPLX3,ULK3,SCAMP2,MPI,FAM219B,COX5A,RPP25,SCAMP5,PPCDC,C15orf39,LOC105376731,COMMD4,NEIL1,NEIL1,MIR631,MAN2C1,SIN3A,PTPN9,SNUPN,IMP3,SNX33,CSPG4,ODF3L1,DNM1P35,MIR4313,UBE2Q2 | 0.144612 | 290.2 | 551 | 333.148 |
| 1 | 19 | 42932988 | 43979781 | LIPE-AS1,CXCL17,LIPE-AS1,LIPE-AS1,CEACAM1,LIPE-AS1,CEACAM8,PSG3,PSG8,PSG10P,PSG1,PSG6,PSG7,PSG11,PSG2,PSG5,PSG4,PSG9,CD177,TEX101,LYPD3,PHLDB3 | 0.143964 | 362.368 | 115 | 59.7238 |
| 1 | 12 | 1.11E+08 | 111922500 | IFT81,ATP2A2,ANAPC7,ARPC3,GPN3,GPN3,FAM216A,FAM216A,VPS29,RAD9B,PPTC7,TCTN1,TCTN1,HVCN1,HVCN1,PPP1CC,CCDC63,MYL2,LINC01405,CUX2,FAM109A,SH2B3,ATXN2 | 0.140999 | 306.623 | 229 | 138.227 |
| 1 | 7 | 1.49E+08 | 151038352 | ZNF786,ZNF425,ZNF398,ZNF282,ZNF212,ZNF783,LOC155060,ZNF777,ZNF746,ZNF767P,KRBA1,ZNF467,SSPO,ZNF862,ATP6V0E2,ACTR3C,ACTR3C,LRRC61,LRRC61,ZBED6CL,LRRC61,RARRES2,REPIN1,ZNF775,GIMAP8,GIMAP7,GIMAP4,GIMAP6,GIMAP2,GIMAP1,GIMAP1-GIMAP5,GIMAP1-GIMAP5,GIMAP5,TMEM176B,TMEM176A,AOC1,KCNH2,NOS3,NOS3,ATG9B,ATG9B,ABCB8,ASIC3,CDK5,SLC4A2,FASTK,TMUB1,AGAP3,GBX1,ASB10,ABCF2,CHPF2,CHPF2,MIR671,SMARCD3 | 0.139334 | 314.088 | 629 | 401.228 |
| 1 | 9 | 1.07E+08 | 114146881 | SMC2,OR13F1,OR13C4,OR13C3,OR13C8,OR13C5,OR13C2,OR13C9,OR13D1,NIPSNAP3A,NIPSNAP3B,ABCA1,SLC44A1,FSD1L,FKTN,TAL2,TMEM38B,ZNF462,ZNF462,LOC340512,RAD23B,KLF4,ACTL7B,ACTL7A,ELP1,FAM206A,CTNNAL1,TMEM245,TMEM245,MIR32,FRRS1L,EPB41L4B,PTPN3,MIR3927,PALM2,PALM2,PALM2-AKAP2,PALM2-AKAP2,PALM2-AKAP2,AKAP2,C9orf152,TXN,TXNDC8,SVEP1,MUSK,LPAR1,OR2K2,KIAA0368 | 0.133553 | 327.217 | 657 | 397.642 |
| 1 | 4 | 1.86E+08 | 186357376 | CENPU,ACSL1,MIR3945HG,MIR3945,MIR4455,HELT,LINC02436,SLC25A4,CFAP97,SNX25,LRP2BP,ANKRD37,ANKRD37,UFSP2,UFSP2,C4orf47 | 0.133541 | 310.958 | 110 | 66.0681 |

| | | | | | | | |
|---|---|---|---|---|---|---|---|
| 1 | 19 | 28263182 | 34900409 | UQCRFS1,VSTM2B,POP4,PLEKHF1,C19orf12,CCNE1,URI1,ZNF536,TSHZ3,THEG5,ZNF507,DPY19L3,PDCD5,ANKRD27,RGS9BP,NUDT19,TDRD12,SLC7A9,CEP89,FAAP24,RHPN2,GPATCH1,WDR88,LRP3,LRP3,SLC7A10,SLC7A10,CEBPA,CEBPG,PEPD,CHST8,KCTD15,LSM14A,KIAA0355,GPI,PDCD2L | 0.129751 | 330.69 | 500 | 300.026 |
| 1 | 13 | 41656862 | 48878244 | WBP4,MIR3168,KBTBD6,LOC101929140,KBTBD7,LOC101929140,MTRF1,MTRF1,MTRF1,NAA16,NAA16,RGCC,VWA8,MIR5006,VWA8,DGKH,AKAP11,TNFSF11,FAM216B,EPSTI1,DNAJC15,ENOX1,CCDC122,LACC1,LINC00390,SMIM2-AS1,SMIM2,SMIM2,SERP2,TSC22D1,TSC22D1,TSC22D1-AS1,NUFIP1,GPALPP1,GTF2F2,GTF2F2,KCTD4,TPT1,SNORA31,TPT1,SLC25A30,COG3,ERICH6B,SPERT,SIAH3,ZC3H13,CPB2-AS1,CPB2,CPB2,LCP1,LRRC63,RUBCNL,LRCH1,ESD,HTR2A,SUCLA2,NUDT15,MED4,MED4-AS1,MED4,ITM2B,RB1 | 0.128696 | 319.557 | 621 | 363.64 |
| 1 | 2 | 2.19E+08 | 221227358 | TNS1,TNS1,MIR6809,LOC105373878,RUFY4,CXCR2P1,RUFY4,CXCR2,CXCR1,ARPC2,GPBAR1,AAMP,PNKD,PNKD,TMBIM1,PNKD,TMBIM1,MIR6513,PNKD,CATIP-AS2,PNKD,CATIP-AS2,MIR6810,CATIP-AS2,CATIP,CATIP,CATIP,CATIP-AS1,SLC11A1,CTDSP1,CTDSP1,MIR26B,VIL1,USP37,CNOT9,PLCD4,ZNF142,BCS1L,RNF25,RNF25,STK36,STK36,TTLL4,CYP27A1,PRKAG3,WNT6,WNT10A,CDK5R2,FEV,CRYBA2,MIR375,LOC100129175,CFAP65,CFAP65,IHH,IHH,MIR3131,NHEJ1,SLC23A3,CNPPD1,RETREG2,ZFAND2B,ABCB6,ATG9A,ANKZF1,GLB1L,STK16,STK16,TUBA4A,TUBA4A,TUBA4A,TUBA4B,TUBA4B,DNAJB2,PTPRN,PTPRN,MIR153-1,RESP18,DNPEP,DES,SPEG,LOC100996693,GMPPA,ASIC4,CHPF,TMEM198,TMEM198,MIR3132,OBSL1,INHA,STK11IP,SLC4A3,MIR4268 | 0.126805 | 311.726 | 860 | 542.831 |
| 1 | 10 | 60500 | 7786975 | TUBB8,ZMYND11,DIP2C,DIP2C,MIR5699,DIP2C,PRR26,LARP4B,GTPBP4,IDI2,IDI2,IDI2-AS1,IDI2-AS1,IDI2-AS1,IDI1,IDI1,WDR37,ADARB2,ADARB2,ADARB2-AS1,PFKP,PITRM1,PITRM1,PITRM1-AS1,KLF6,AKR1E2,AKR1C1,AKR1C2,AKR1C3,AKR1C8P,AKR1C4,UCN3,TUBAL3,NET1,CALML5,CALML3-AS1,CALML3-AS1,CALML3,ASB13,FAM208B,GDI2,ANKRD16,FBXO18,IL15RA,IL2RA,RBM17,PFKFB3,PFKFB3,MIR3155A,MIR3155B,LOC399715,LOC399716,PRKCQ,SFMBT2,ITIH5,ITIH2 | 0.126789 | 307.7 | 670 | 397.042 |
| 1 | 10 | 29821497 | 32096041 | SVIL,SVIL,MIR604,SVIL,MIR938,JCAD,MTPAP,GOLGA2P6,MAP3K8,LYZL2,SVILP1,ZNF438,ZEB1-AS1,ZEB1,ZEB1 | 0.126763 | 324.776 | 120 | 74.6551 |
| 1 | 20 | 52831756 | 54970834 | PFDN4,DOK5,CBLN4,MC3R,FAM210B,AURKA,CSTF1 | 0.126418 | 318.183 | 53 | 31.0081 |
| 1 | X | 1.23E+08 | 129338387 | STAG2,SH2D1A,TENM1,TEX13C,DCAF12L2,DCAF12L1,PRR32,ACTRT1,SMARCA1,OCRL,APLN,XPNPEP2,SASH3,ZDHHC9,UTP14A,BCORL1,ELF4,AIFM1,RAB33A | 0.125881 | 324.197 | 321 | 197.74 |

| | | | | | | | |
|---|---|---|---|---|---|---|---|
| 1 | 5 | 68709931 | 72144239 | RAD17,MARVELD2,OCLN,GTF2H2C_2,GTF2H2C,SERF1A,SERF1B,SMN1,SMN2,GUSBP3,GTF2H2B,GUSBP3,NAIP,GTF2H2,LOC647859,BDP1,MCCC2,CARTPT,MAP1B,MAP1B,MIR4803,MRPS27,PTCD2,ZNF366,TNPO1 | 0.125042 | 320.618 | 268 | 148.569 |
| 1 | 3 | 46874922 | 48448358 | PRSS42,MYL3,PTH1R,CCDC12,NBEAL2,NRADDP,SETD2,KIF9-AS1,KIF9,KIF9,KLHL18,PTPN23,SCAP,ELP6,CSPG5,SMARCC1,DHX30,DHX30,MIR1226,MAP4,CDC25A,MIR4443,CAMP,ZNF589,NME6,SPINK8,SPINK8,MIR2115,FBXW12,PLXNB1 | 0.123128 | 315.787 | 422 | 270.6 |
| 1 | 19 | 59073417 | 59118483 | MZF1-AS1,MZF1 | 0.123111 | 361.106 | 12 | 8.01516 |
| 1 | 7 | 74167958 | 76871162 | NCF1,GTF2IRD2,RCC1L,GTF2IRD2,GTF2IRD2B,NCF1C,GTF2IP4,GTF2IP1,GTF2IP1,PMS2P5,GTF2IP1,PMS2P5,CASTOR2,TRIM73,NSUN5P1,NSUN5P1,POM121C,POM121C,PMS2P3,HIP1,CCL26,CCL24,RHBDD2,POR,MIR4651,POR,SNORA14A,POR,TMEM120A,STYXL1,MDH2,SRRM3,HSPB1,YWHAG,SSC4D,SSC4D,ZP3,ZP3,DTX2,UPK3B,LOC100133091,POMZP3,DTX2P1-UPK3BP1-PMS2P11,FAM185BP,CCDC146,CCDC146,FGL2 | 0.122644 | 277.772 | 368 | 197.972 |
| 1 | 16 | 73126840 | 84118820 | HCCAT5,C16orf47,PSMD7,LOC283922,NPIPB15,CLEC18B,GLG1,RFWD3,MLKL,FA2H,WDR59,ZNRF1,LDHD,ZFP1,CTRB2,CTRB1,BCAR1,CFDP1,TMEM170A,CHST6,CHST5,TMEM231,GABARAPL2,ADAT1,KARS,TERF2IP,DUXB,CNTNAP4,LINC02125,MIR4719,MON1B,MON1B,SYCE1L,SYCE1L,ADAMTS18,NUDT7,VAT1L,CLEC3A,WWOX,MAF,LOC102724084,DYNLRB2,CDYL2,CMC2,CENPN,ATMIN,C16orf46,GCSH,PKD1L2,BCO1,GAN,MIR4720,CMIP,PLCG2,SDR42E1,HSD17B2,MPHOSPH6,CDH13,CDH13,MIR3182,HSBP1,MLYCD,OSGIN1,NECAB2,SLC38A8,MBTPS1 | 0.121034 | 298.501 | 881 | 519.047 |
| 1 | 16 | 5046243 | 8993698 | SEC14L5,NAGPA,NAGPA,NAGPA-AS1,C16orf89,ALG1,RBFOX1,TMEM114,METTL22,ABAT,TMEM186,TMEM186,PMM2,PMM2,LOC100130283,CARHSP1,CARHSP1,USP7 | 0.11936 | 258.129 | 160 | 97.6645 |
| 1 | 14 | 19057400 | 21678940 | DUXAP10,BMS1P22,OR4Q3,OR4M1,OR4N2,OR4K2,OR4K5,OR4K1,OR4K15,OR4Q2,OR4K14,OR4K13,OR4L1,OR4K17,OR4N5,OR11G2,OR11H6,OR11H7,OR11H4,TTC5,CCNB1IP1,CCNB1IP1,SNORD126,PARP2,TEP1,KLHL33,OSGEP,APEX1,PIP4P1,PNP,RNASE10,RNASE9,RNASE11,LOC254028,RNASE12,LOC254028,OR6S1,ANG,RNASE4,RNASE4,EDDM3A,EDDM3B,RNASE6,RNASE1,RNASE3,ECRP,RNASE2,METTL17,METTL17,LOC101929718,SLC39A2,NDRG2,NDRG2,MIR6717,NDRG2,TPPP2,NDRG2,RNASE13,NDRG2,RNASE7,NDRG2,RNASE8,NDRG2,ARHGEF40,ARHGEF40,ARHGEF40,ZNF219,ZNF219,ZNF219,TMEM253,OR5AU1 | 0.118991 | 331.333 | 399 | 239.433 |

| | | | | | | | | |
|---|---|---|---|---|---|---|---|---|
| 1 | 19 | 652767 | 11941280 | RNF126,FSTL3,PRSS57,PALM,MISP,PTBP1,PTBP1,MIR4745,PLPPR3,PLPPR3,MIR3187,AZU1,PRTN3,ELANE,CFD,MED16,R3HDM4,KISS1R,ARID3A,WDR18,GRIN3B,GRIN3B,TMEM259,TMEM259,CNN2,ABCA7,ARHGAP45,POLR2E,GPX4,SBNO2,STK11,CBARP,ATP5F1D,MIDN,CIRBP-AS1,CIRBP-AS1,CIRBP,CIRBP,C19orf24,EFNA2,MUM1,NDUFS7,GAMT,DAZAP1,RPS15,APC2,C19orf25,PCSK4,REEP6,ADAMTSL5,PLK5,MEX3D,MBD3,UQCR11,TCF3,ONECUT3,ATP8B3,REXO1,REXO1,MIR1909,REXO1,LOC100288123,KLF16,ABHD17A,ADAT3,SCAMP4,SCAMP4,CSNK1G2,CSNK1G2-AS1,CSNK1G2,BTBD2,MKNK2,MOB3A,IZUMO4,AP3D1,DOT1L,PLEKHJ1,MIR1227,PLEKHJ1,MIR6789,PLEKHJ1,SF3A2,AMH,AMH,MIR4321,JSRP1,OAZ1,PEAK3,LINGO3,LSM7,SPPL2B,TMPRSS9,TMPRSS9,TIMM13,TIMM13,LMNB2,LMNB2,MIR7108,GADD45B,GNG7,DIRAS1,SLC39A3,SGTA,THOP1,ZNF554,ZNF555,ZNF556,ZNF57,ZNF77,TLE6,TLE2,AES,GNA11,GNA15,GNA15,LOC100996351,S1PR4,NCLN,CELF5,NFIC,SMIM24,DOHH,FZR1,C19orf71,MFSD12,HMG20B,GIPC3,TBXA2R,CACTIN-AS1,CACTIN,CACTIN,PIP5K1C,TJP3,TJP3,MIR1268A,MIR1268A,APBA3,MIR1268A,MRPL54,MIR1268A,RAX2,MIR1268A,MATK,MIR1268A,ZFR2,MIR1268A,ATCAY,MIR1268A,NMRK2,MIR1268A,DAPK3,MIR1268A,DAPK3,MIR637,MIR1268A,EEF2,MIR1268A,EEF2,SNORD37,PIAS4,ZBTB7A,MAP2K2,CREB3L3,SIRT6,ANKRD24,EBI3,CCDC94,SHD,TMIGD2,FSD1,STAP2,MPND,SH3GL1,CHAF1A,UBXN6,UBXN6,MIR4746,HDGFL2,HDGFL2,PLIN4,PLIN4,PLIN5,LRG1,SEMA6B,TNFAIP8L1,MYDGF,DPP9,DPP9,DPP9-AS1,MIR7-3HG,MIR7-3HG,MIR7-3,FEM1A,TICAM1,PLIN3,ARRDC5,UHRF1,UHRF1,MIR4747,KDM4B,PTPRS,ZNRF4,SAFB2,SAFB,C19orf70,HSD1 | 0.117957 | 320.963 | 4059 | 2512.78 |
| 1 | X | 1.54E+08 | 155260060 | GAB3,DKC1,DKC1,SNORA36A,MIR664B,DKC1,SNORA56,MPP1,SMIM9,F8,FUNDC2,CMC4,CMC4,MTCP1,BRCC3,VBP1,RAB39B,CLIC2,TMLHE-AS1,LOC101927830,TMLHE-AS1,LOC101927830,TMLHE,TMLHE,SPRY3,VAMP7,IL9R | 0.11168 | 302.009 | 189 | 105.805 |
| 1 | 13 | 19020500 | 21142192 | TUBA3C,LOC101928697,TPTE2,MPHOSPH8,PSPC1,ZMYM5,ZMYM2,GJA3,GJB2,GJB6,CRYL1,CRYL1,MIR4499,IFT88 | 0.10906 | 324.6 | 170 | 90.9397 |
| 1 | 5 | 10500 | 10250499 | PLEKHG4B,LRRC14B,CCDC127,SDHA,PDCD6,PDCD6,AHRR,AHRR,EXOC3-AS1,EXOC3,PP7080,SLC9A3,SLC9A3,SLC9A3-AS1,SLC9A3,MIR4456,CEP72,TPPP,ZDHHC11B,ZDHHC11,BRD9,TRIP13,LOC100506688,NKD2,SLC12A7,SLC12A7,MIR4635,SLC6A19,SLC6A18,TERT,MIR4457,CLPTM1L,LINC01511,SLC6A3,LPCAT1,SDHAP3,LOC728613,MIR4277,MRPL36,NDUFS6,IRX4,IRX2,C5orf38,LINC01019,IRX1,ADAMTS16,ICE1,LINC02145,MED10,UBE2QL1,NSUN2,SRD5A1,PAPD7,MIR4278,LOC442132,ADCY2,C5orf49,FASTKD3,FASTKD3,MTRR,MTRR,MIR4458HG,MIR4458,SEMA5A,SEMA5A,MIR4636,SNHG18,SNORD123,TAS2R1,FAM173B,CCT5 | 0.109038 | 295.729 | 729 | 427.497 |

| | | | | | | | | |
|---|---|---|---|---|---|---|---|---|
| 1 | 8 | 39695533 | 43152541 | ADAM2,IDO1,IDO2,TCIM,ZMAT4,SFRP1,SFRP1,MIR548AO,GOLGA7,GINS4,GINS4,LOC102723729,GPAT4,NKX6-3,ANK1,ANK1,MIR486-1,MIR486-2,KAT6A,AP3M2,PLAT,IKBKB,POLB,DKK4,VDAC3,SLC20A2,SMIM19,CHRNB3,CHRNA6,THAP1,RNF170,RNF170,MIR4469,HOOK3,FNTA,POMK,HGSNAT,POTEA | 0.103737 | 308.35 | 417 | 251.54 |
| 1 | 14 | 21851714 | 23978687 | SUPT16H,CHD8,CHD8,SNORD9,CHD8,SNORD8,RAB2B,TOX4,TOX4,METTL3,METTL3,SALL2,OR10G3,OR10G2,OR4E2,LOC105370401,LINC02332,DAD1,ABHD4,OR6J1,OXA1L,SLC7A7,MRPL52,MMP14,LRP10,REM2,RBM23,PRMT5-AS1,PRMT5,PRMT5,LOC101926933,HAUS4,HAUS4,HAUS4,MIR4707,AJUBA,C14orf93,PSMB5,PSMB11,CDH24,ACIN1,C14orf119,LMLN2,CEBPE,SLC7A8,RNF212B,HOMEZ,PPP1R3E,BCL2L2,BCL2L2-PABPN1,BCL2L2-PABPN1,BCL2L2-PABPN1,PABPN1,SLC22A17,EFS,IL25,CMTM5,MYH6,MYH6,MIR208A,MYH7,MYH7,MHRT,MYH7,MIR208B,NGDN | 0.10364 | 306.542 | 770 | 468.327 |
| 1 | 6 | 61944508 | 70071390 | KHDRBS2,LGSN,PTP4A1,PHF3,EYS,EYS,LOC441155,ADGRB3 | 0.101726 | 293.914 | 241 | 142.21 |
| 1 | 2 | 96652782 | 97783970 | ANKRD36C,FAHD2CP,FAHD2CP,GPAT2,GPAT2,ADRA2B,ASTL,DUSP2,STARD7,STARD7,STARD7-AS1,STARD7-AS1,TMEM127,CIAO1,SNRNP200,ITPRIPL1,NCAPH,NEURL3,ARID5A,KANSL3,FER1L5,LMAN2L,CNNM4,CNNM4,MIR3127,CNNM3,ANKRD23,ANKRD39,SEMA4C,FAM178B,FAM178B,LOC101927053,FAHD2B,ANKRD36 | 0.0980658 | 306.439 | 364 | 223.955 |
| 1 | 8 | 1.31E+08 | 142199222 | FAM49B,FAM49B,MIR5194,ASAP1,ADCY8,EFR3A,OC90,HHLA1,KCNQ3,LRRC6,TMEM71,PHF20L1,TG,TG,SLA,WISP1,NDRG1,ST3GAL1,ZFAT,ZFAT,ZFAT-AS1,MIR30B,MIR30D,LINC01591,KHDRBS3,FAM135B,COL22A1,KCNK9,TRAPPC9,CHRAC1,AGO2,PTK2,DENND3 | 0.0963937 | 275.2 | 733 | 425.115 |

| | | | | | | | | |
|---|---|---|---|---|---|---|---|---|
| 1 | X | 46618219 | 54255283 | SLC9A7,RP2,LINC01545,JADE3,RGN,NDUFB11,RBM10,UBA1,UBA1,INE1,CDK16,USP11,ZNF157,ZNF157,SNORA11C,ZNF41,LINC01560,ARAF,SYN1,SYN1,TIMP1,SYN1,MIR4769,CFP,ELK1,UXT,UXT,UXT-AS1,UXT-AS1,ZNF81,ZNF182,SPACA5,SPACA5B,ZNF630-AS1,ZNF630-AS1,ZNF630,ZNF630,SSX6,SSX5,SSX1,SSX9,SSX3,SSX4B,SSX4,SSX4,SSX4B,SLC38A5,FTSJ1,PORCN,EBP,TBC1D25,RBM3,WDR13,WAS,SUV39H1,GLOD5,GATA1,HDAC6,ERAS,PCSK1N,TIMM17B,PQBP1,SLC35A2,PIM2,OTUD5,KCND1,GRIPAP1,TFE3,CCDC120,PRAF2,WDR45,GPKOW,MAGIX,PLP2,PRICKLE3,SYP,SYP,SYP-AS1,CACNA1F,CCDC22,CCDC22,FOXP3,FOXP3,PPP1R3F,GAGE10,GAGE1,PAGE1,PAGE4,USP27X-AS1,USP27X,CLCN5,CLCN5,MIR532,CLCN5,MIR188,CLCN5,MIR500A,CLCN5,MIR362,CLCN5,MIR501,CLCN5,MIR500B,CLCN5,MIR660,CLCN5,MIR502,AKAP4,CCNB3,DGKK,SHROOM4,BMP15,NUDT10,CXorf67,NUDT11,GSPT2,MAGED1,MAGED4B,MAGED4,MAGED4B,MAGED4,SNORA11D,SNORA11E,XAGE2,SSX8,SSX7,SSX2B,SSX2,SSX2,SSX2B,SPANXN5,XAGE5,XAGE3,FAM156B,FAM156A,GPR173,TSPYL2,KDM5C,KDM5C,MIR6895,KDM5C,MIR6894,IQSEC2,SMC1A,SMC1A,MIR6857,RIBC1,HSD17B10,HUWE1,HUWE1,MIR98,HUWE1,MIRLET7F2,PHF8,FAM120C,WNK3 | 0.0951354 | 318.714 | 1304 | 801.293 |
| 1 | 11 | 70197011 | 70782785 | PPFIA1,CTTN,SHANK2,SHANK2,SHANK2-AS3,SHANK2,MIR3664 | 0.0942756 | 303.235 | 97 | 59.1911 |

| | | | | | | | | |
|---|---|---|---|---|---|---|---|---|
| 1 | 19 | 13317371 | 19869274 | CACNA1A,CCDC130,MRI1,C19orf53,ZSWIM4,LOC284454,MIR24-2,MIR27A,MIR23A,NANOS3,MIR181C,MIR181D,NANOS3,C19orf57,CC2D1A,PODNL1,PODNL1,DCAF15,DCAF15,RFX1,RLN3,IL27RA,PALM3,MISP3,MISP3,MIR1199,C19orf67,SAMD1,PRKACA,ASF1B,LOC100507373,ADGRL1,ADGRL1,ADGRE5,DDX39A,PKN1,PTGER1,GIPC1,DNAJB1,MIR639,TECR,TECR,NDUFB7,CLEC17A,ADGRE3,ZNF333,ADGRE2,OR7C1,OR7A5,OR7A10,OR7A17,OR7C2,SLC1A6,CCDC105,CASP14,OR1I1,SYDE1,ILVBL,NOTCH3,NOTCH3,MIR6795,EPHX3,BRD4,AKAP8,AKAP8L,WIZ,RASAL3,PGLYRP2,CYP4F22,CYP4F8,CYP4F3,CYP4F12,OR10H2,OR10H3,CYP4F24P,OR10H5,OR10H1,CYP4F2,CYP4F11,OR10H4,LINC00661,LINC00905,TPM4,RAB8A,HSH2D,CIB3,FAM32A,AP1M1,KLF2,EPS15L1,CALR3,C19orf44,C19orf44,CHERP,CHERP,SLC35E1,MED26,SMIM7,TMEM38A,NWD1,SIN3B,F2RL3,CPAMD8,HAUS8,MYO9B,USE1,OCEL1,NR2F6,USHBP1,BABAM1,ANKLE1,ABHD8,MRPL34,DDA1,ANO8,GTPBP3,PLVAP,BST2,MVB12A,TMEM221,NXNL1,SLC27A1,SLC27A1,LOC100507551,LOC100507551,PGLS,PGLS,FAM129C,COLGALT1,UNC13A,MAP1S,FCHO1,B3GNT3,INSL3,JAK3,RPL18A,RPL18A,SNORA68,SLC5A5,CCDC124,KCNN1,ARRDC2,IL12RB1,IL12RB1,MAST3,MAST3,PIK3R2,IFI30,MPV17L2,RAB3A,PDE4C,IQCN,JUND,MIR3188,LSM4,PGPEP1,GDF15,GDF15,MIR3189,LRRC25,SSBP4,ISYNA1,ELL,FKBP8,KXD1,UBA52,REX1BD,CRLF1,TMEM59L,KLHL26,CRTC1,COMP,UPF1,CERS1,GDF1,COPE,DDX49,HOMER3,SUGP2,SUGP2,ARMC6,ARMC6,SLC25A42,TMEM161A,MEF2B,BORCS8-MEF2B,BORCS8-MEF2B,BORCS8,RFXANK,RFXANK,NR2C2AP,NR2C2AP,NCAN,HAPLN4,TM6SF2,SUGP1,MAU2,GATAD2A,MIR640,GATAD2A,TSSK6,NDUFA13,YJEFN3,CILP2,PBX4,LPA | 0.0869975 | 318.305 | 2177 | 1341.75 |
| 1 | 1 | 1.1E+08 | 110754866 | CELSR2,PSRC1,MYBPHL,SORT1,PSMA5,SYPL2,ATXN7L2,CYB561D1,AMIGO1,GPR61,GNAI3,MIR197,GNAT2,AMPD2,GSTM4,GSTM2,GSTM5,GSTM3,EPS8L3,CSF1,AHCYL1,STRIP1,ALX3,UBL4B,SLC6A17,KCNC4 | 0.0861688 | 324.492 | 261 | 161.697 |
| 1 | 8 | 17143858 | 20082772 | VPS37A,MTMR7,SLC7A2,PDGFRL,MTUS1,MTUS1,MIR548V,FGL1,PCM1,ASAH1,NAT1,NAT2,PSD3,SH2D4A,CSGALNACT1,INTS10,LPL,SLC18A1,ATP6V1B2 | 0.080966 | 314.798 | 302 | 183.553 |
| 1 | 8 | 94829255 | 95735247 | MIR378D2,PDP1,CDH17,GEM,RAD54B,RAD54B,FSBP,VIRMA,LOC100288748,ESRP1,DPY19L4 | 0.080322 | 331.232 | 113 | 70.9663 |
| 1 | 7 | 1.42E+08 | 143884193 | MGAM2,PRSS58,PRSS1,PRSS3P2,EPHB6,TRPV6,TRPV5,LLCFC1,KEL,OR9A2,OR6V1,OR6W1P,PIP,TAS2R39,TAS2R40,LOC105375545,LOC105375545,GSTK1,LOC105375545,TMEM139,TMEM139,CASP2,CLCN1,FAM131B,ZYX,ZYX,MIR6892,EPHA1,EPHA1,EPHA1-AS1,EPHA1-AS1,TAS2R60,EPHA1-AS1,TAS2R41,TCAF2,LOC154761,TCAF1,OR2F2,OR2F1,OR6B1,OR2A5,OR2A25,OR2A12,OR2A2,OR2A14,ARHGEF35 | 0.0797919 | 348.964 | 422 | 246.323 |
| 1 | 11 | 72287581 | 72291731 | PDE2A | 0.0792453 | 303.327 | 8 | 5.3739 |

| | | | | | | | | |
|---|---|---|---|---|---|---|---|---|
| 1 | 2 | 26532773 | 27891629 | ADGRF3,ADGRF3,SELENOI,SELENOI,DRC1,OTOF,C2orf70,CIB4,KCNK3,SLC35F6,CENPA,DPYSL5,MAPRE3,TMEM214,AGBL5,OST4,EMILIN1,KHK,KHK,CGREF1,CGREF1,ABHD1,PREB,PRR30,TCF23,SLC5A6,SLC5A6,ATRAID,ATRAID,CAD,SLC30A3,DNAJC5G,TRIM54,UCN,MPV17,GTF3C2,GTF3C2,GTF3C2-AS1,EIF2B4,SNX17,SNX17,ZNF513,ZNF513,PPM1G,NRBP1,KRTCAP3,KRTCAP3,IFT172,IFT172,FNDC4,GCKR,C2orf16,ZNF512,CCDC121,CCDC121,GPN1,GPN1,SUPT7L,SUPT7L,SLC4A1AP,SLC4A1AP | 0.078379 | 309.21 | 622 | 398.515 |
| 1 | 15 | 40951555 | 42453082 | KNL1,RAD51,RMDN3,GCHFR,DNAJC17,DNAJC17,C15orf62,DNAJC17,ZFYVE19,ZFYVE19,PPP1R14D,SPINT1-AS1,SPINT1,SPINT1,RHOV,VPS18,DLL4,CHAC1,INO80,EXD1,CHP1,OIP5,NUSAP1,NDUFAF1,RTF1,ITPKA,LTK,RPAP1,TYRO3,MGA,MGA,MIR626,MAPKBP1,JMJD7,JMJD7-PLA2G4B,JMJD7-PLA2G4B,PLA2G4B,SPTBN5,SPTBN5,MIR4310,SPTBN5,LOC105370792,LOC105370792,EHD4,PLA2G4E-AS1,PLA2G4E,PLA2G4E,PLA2G4D,PLA2G4F,VPS39 | 0.0774811 | 293.521 | 581 | 366.32 |
| 1 | X | 1.18E+08 | 122766902 | DOCK11,IL13RA1,ZCCHC12,LONRF3,KIAA1210,PGRMC1,SLC25A43,SLC25A5-AS1,SLC25A5,SLC25A5,CXorf56,UBE2A,NKRF,SEPT6,SEPT6,MIR766,SOWAHD,RPL39,RPL39,SNORA69,UPF3B,RNF113A,NDUFA1,AKAP14,NKAP,RHOXF1-AS1,RHOXF2B,RHOXF2,RHOXF1-AS1,RHOXF1,RHOXF2,RHOXF2B,ZBTB33,TMEM255A,ATP1B4,LAMP2,CUL4B,MCTS1,C1GALT1C1,CT47B1,GLUD2,MIR3672,GRIA3,THOC2 | 0.0761325 | 307.548 | 393 | 232.078 |
| 1 | 7 | 65139852 | 74103469 | LOC441242,INTS4P2,LOC441242,CCT6P1,SNORA22,LOC441242,VKORC1L1,GUSB,ASL,CRCP,TPST1,LINC00174,KCTD7,RABGEF1,TMEM248,SBDS,TYW1,TYW1,MIR4650-1,MIR4650-2,PMS2P4,STAG3L4,AUTS2,GALNT17,GALNT17,MIR3914-1,MIR3914-2,CALN1,TYW1B,TYW1B,MIR4650-1,MIR4650-2,SBDSP1,POM121,POM121,NSUN5P2,TRIM74,LOC541473,LOC100101148,GTF2IP4,GTF2IP1,NCF1B,NSUN5,TRIM50,FKBP6,FZD9,BAZ1B,BCL7B,TBL2,MLXIPL,VPS37D,DNAJC30,BUD23,STX1A,STX1A,MIR4284,ABHD11,CLDN3,CLDN4,METTL27,TMEM270,ELN,LIMK1,EIF4H,EIF4H,MIR590,LAT2,RFC2,CLIP2,GTF2IRD1,GTF2I,LOC101926943 | 0.0720584 | 280.751 | 653 | 372.272 |

| | | | | | | | | |
|---|---|---|---|---|---|---|---|---|
| 1 | 21 | 30445738 | 38459151 | CCT8,MAP3K7CL,BACH1,GRIK1,GRIK1,GRIK1-AS2,CLDN17,CLDN8,KRTAP24-1,KRTAP25-1,KRTAP26-1,KRTAP27-1,KRTAP23-1,KRTAP13-2,MIR4327,KRTAP13-1,KRTAP13-3,KRTAP13-4,KRTAP15-1,KRTAP19-1,KRTAP19-2,KRTAP19-3,KRTAP19-4,KRTAP19-5,KRTAP19-6,KRTAP19-7,KRTAP22-2,KRTAP6-3,KRTAP6-2,KRTAP22-1,KRTAP6-1,KRTAP20-1,KRTAP20-4,KRTAP20-2,KRTAP20-3,KRTAP21-3,KRTAP21-2,KRTAP21-1,KRTAP8-1,KRTAP7-1,KRTAP11-1,KRTAP19-8,TIAM1,LOC150051,SOD1,SCAF4,HUNK,MIS18A,MRAP,MRAP,URB1,URB1,URB1,SNORA80A,URB1-AS1,EVA1C,C21orf59-TCP10L,TCP10L,C21orf59-TCP10L,C21orf59-TCP10L,C21orf59,SYNJ1,SYNJ1,PAXBP1-AS1,PAXBP1-AS1,PAXBP1,PAXBP1,C21orf62-AS1,C21orf62-AS1,C21orf62,C21orf62,OLIG2,OLIG1,LINC01548,IFNAR2,IL10RB-AS1,IL10RB,IFNAR1,IFNGR2,IFNGR2,TMEM50B,TMEM50B,DNAJC28,GART,SON,SON,MIR6501,DONSON,CRYZL1,ITSN1,ATP5PO,SLC5A3,MRPS6,MRPS6,KCNE2,SMIM11A,SMIM11B,C21orf140,KCNE1,KCNE1B,RCAN1,CLIC6,RUNX1,RUNX1,RUNX1-IT1,MIR802,SETD4,LOC100133286,CBR1,CBR3-AS1,CBR3,DOPEY2,MORC3,CHAF1B,CLDN14,SIM2,HLCS,RIPPLY3,PIGP | 0.0702476 | 318.033 | 904 | 540.311 |
| 1 | 5 | 1.48E+08 | 151125400 | FBXO38,HTR4,ADRB2,SH3TC2,ABLIM3,AFAP1L1,GRPEL2,GRPEL2,GRPEL2-AS1,PCYOX1L,IL17B,CARMN,MIR143,CARMN,MIR145,CSNK1A1,ARHGEF37,PPARGC1B,PPARGC1B,MIR378A,PDE6A,SLC26A2,TIGD6,TIGD6,HMGXB3,HMGXB3,CSF1R,PDGFRB,CDX1,SLC6A7,CAMK2A,ARSI,TCOF1,CD74,RPS14,NDST1,SYNPO,MYOZ3,RBM22,DCTN4,SMIM3,IRGM,IRGM,ZNF300,ZNF300,ZNF300P1,GPX3,TNIP1,ANXA6,CCDC69,GM2A,SLC36A3,SLC36A2,SLC36A1,FAT2,SPARC | 0.0693765 | 299.8 | 694 | 436.709 |
| 1 | 9 | 65468179 | 72913113 | LINC01410,PTGER4P2-CDK2AP2P2,LOC403323,LINC00537,MIR4477A,MIR4477B,FRG1JP,MIR1299,PGM5P2,CBWD6,CBWD5,PGM5,TMEM252,PIP5K1B,FAM122A,PIP5K1B,PIP5K1B,LOC101927069,PRKACG,FXN,TJP2,FAM189A2,APBA1,PTAR1,C9orf135,MAMDC2,MAMDC2,MAMDC2-AS1,MAMDC2,SMC5-AS1,SMC5 | 0.0669578 | 295.41 | 227 | 127.528 |
| 1 | 14 | 97348071 | 101377664 | LINC00618,LINC02291,LINC02312,LINC01550,C14orf177,BCL11B,SETD3,CCNK,CCDC85C,HHIPL1,CYP46A1,EML1,EVL,EVL,MIR151B,MIR342,DEGS2,YY1,YY1,MIR6764,SLC25A29,MIR345,SLC25A47,WARS,WDR25,BEGAIN,LINC00523,DLK1,MIR2392,MEG3,MEG3,MIR770,MIR493,MIR337,MIR665,RTL1,RTL1,MIR431,RTL1,MIR433,RTL1,MIR127,RTL1,MIR432,RTL1,MIR136,MEG8,MEG8,MIR370 | 0.0598705 | 267.216 | 299 | 180.851 |
| 1 | 3 | 56716629 | 57231704 | FAM208A,ARHGEF3,ARHGEF3,ARHGEF3-AS1,ARHGEF3,SPATA12,IL17RD | 0.0565978 | 246.621 | 60 | 35.2414 |

| | | | | | | | | |
|---|---|---|---|---|---|---|---|---|
| 1 | X | 1.29E+08 | 148582684 | SLC25A14,GPR119,RBMX2,FAM45BP,ENOX2,ARHGAP36,IGSF1,OR13H1,STK26,FRMD7,RAP2C,RAP2C-AS1,MBNL3,MBNL3,HS6ST2,HS6ST2,HS6ST2-AS1,USP26,TFDP3,GPC4,GPC3,MIR363,MIR92A2,MIR19B2,MIR20B,MIR18B,MIR106A,CCDC160,PHF6,HPRT1,MIR450B,MIR450A1,MIR450A1,MIR450A2,MIR542,MIR503HG,MIR503,MIR503HG,MIR424,PLAC1,FAM122B,FAM122C,MOSPD1,SMIM10,RTL8B,RTL8C,RTL8A,SMIM10L2B-AS1,CT55,ZNF75D,ZNF449,INTS6L,CT45A10,SAGE1,MMGT1,SLC9A6,FHL1,MAP7D3,ADGRG4,BRS3,HTATSF1,VGLL1,VGLL1,MIR934,CD40LG,ARHGEF6,RBMX,RBMX,SNORD61,GPR101,ZIC3,FGF13,FGF13,MIR504,F9,MCF2,ATP11C,ATP11C,MIR505,CXorf66,LOC728660,SOX3,LINC00632,CDR1,MIR320D2,SPANXB1,LDOC1,SPANXA2-OT1,LOC645188,SPANXD,SPANXC,MAGEC3,MAGEC1,MAGEC2,SPANXN4,SPANXN3,SLITRK4,SPANXN2,UBE2NL,SPANXN1,SLITRK2,MIR890,MIR888,MIR892A,MIR892B,MIR891B,MIR891A,CXorf51A,CXorf51B,MIR513C,MIR513B,MIR513A1,MIR513A2,MIR506,MIR506,MIR507,MIR508,MIR514B,MIR509-1,MIR509-2,MIR509-3,MIR509-3,MIR510,FMR1,FMR1NB,AFF2,IDS | 0.054692 | 306.705 | 984 | 577.626 |
| 1 | 9 | 19102432 | 26982397 | HAUS6,PLIN2,DENND4C,RPS6,ACER2,SLC24A2,MLLT3,MLLT3,MIR4473,MLLT3,MIR4474,FOCAD,FOCAD,MIR491,HACD4,IFNB1,IFNW1,IFNA21,IFNA7,IFNA16,IFNA14,IFNA5,KLHL9,IFNA6,IFNA13,IFNA2,IFNA8,IFNA1,MIR31HG,IFNE,MIR31HG,MIR31,MTAP,CDKN2A-AS1,CDKN2A,CDKN2B-AS1,CDKN2B,CDKN2B-AS1,DMRTA1,ELAVL2,IZUMO3,TUSC1,LOC100506422,CAAP1,PLAA,PLAA,IFT74,IFT74 | 0.0512745 | 316.27 | 361 | 210.212 |
| 1 | 4 | 1.86E+08 | 186539885 | CCDC110,CCDC110,LOC105377590,LOC105377590,PDLIM3,SORBS2 | 0.0500662 | 334.635 | 26 | 15.8386 |
| 1 | 8 | 1.25E+08 | 125499850 | FAM91A1,FER1L6,FER1L6,FER1L6-AS1,FER1L6,FER1L6-AS2,TMEM65,TRMT12,RNF139 | 0.0496081 | 314.922 | 77 | 47.0163 |
| 1 | 4 | 10509495 | 17814018 | CLNK,HS3ST1,RAB28,LINC01097,NKX3-2,LINC01096,BOD1L1,MIR5091,CPEB2-AS1,CPEB2,CPEB2,LOC101929095,LOC101929095,C1QTNF7,C1QTNF7,CC2D2A,FBXL5,FAM200B,BST1,CD38,FGFBP1,FGFBP2,PROM1,TAPT1,TAPT1-AS1,LDB2,QDPR,CLRN2,LAP3,MED28,FAM184B,DCAF16,NCAPG | 0.0481884 | 276.766 | 412 | 248.798 |
| 1 | 4 | 1.69E+08 | 170482750 | PALLD,CBR4,SH3RF1,NEK1 | 0.047688 | 282.139 | 87 | 50.8567 |

| | | | | | | | | |
|---|---|---|---|---|---|---|---|---|
| 1 | 11 | 60500 | 51566742 | SCGB1C2,SCGB1C1,ODF3,BET1L,RIC8A,RIC8A,MIR6743,SIRT3,PSMD13,NLRP6,PGGHG,IFITM5,IFITM2,IFITM1,IFITM3,B4GALNT4,PKP3,SIGIRR,ANO9,PTDSS2,RNH1,HRAS,LRRC56,LMNTD2,LMNTD2,LOC692247,RASSF7,MIR210HG,MIR210,PHRF1,IRF7,CDHR5,SCT,DRD4,DEAF1,DEAF1,TMEM80,TMEM80,EPS8L2,TALDO1,GATD1,LOC171391,CEND1,SLC25A22,PIDD1,RPLP2,RPLP2,SNORA52,PNPLA2,CRACR2B,CD151,POLR2L,TSPAN4,CHID1,AP2A2,MUC6,MUC2,MUC5AC,MUC5B,MUC5B,MIR6744,TOLLIP,BRSK2,MOB2,DUSP8,KRTAP5-AS1,KRTAP5-1,KRTAP5-AS1,KRTAP5-2,KRTAP5-AS1,KRTAP5-3,KRTAP5-5,KRTAP5-6,IFITM10,CTSD,SYT8,TNNI2,LSP1,LSP1,MIR4298,LSP1,MIR7847,TNNT3,MRPL23,MRPL23-AS1,HOTS,H19,H19,H19,MIR675,IGF2,INS-IGF2,IGF2,INS-IGF2,MIR483,IGF2,INS-IGF2,IGF2-AS,INS-IGF2,INS-IGF2,INS,TH,MIR4686,ASCL2,C11orf21,C11orf21,TSPAN32,TSPAN32,CD81-AS1,CD81-AS1,CD81,CD81,TSSC4,TRPM5,KCNQ1,KCNQ1,KCNQ1OT1,KCNQ1,KCNQ1-AS1,CDKN1C,SLC22A18AS,SLC22A18AS,SLC22A18,SLC22A18,PHLDA2,NAP1L4,NAP1L4,SNORA54,CARS,CARS,CARS-AS1,OSBPL5,MRGPRG,MRGPRG,MRGPRG-AS1,MRGPRG-AS1,MRGPRE,ZNF195,ART5,ART1,CHRNA10,NUP98,PGAP2,RHOG,STIM1,MIR4687,STIM1,RRM1,OR52B4,TRIM21,OR52K2,OR52K1,OR52M1,C11orf40,OR52I2,OR52I1,TRIM68,OR51D1,OR51E1,OR51E2,OR51F1,OR52R1,OR51F2,OR51S1,OR51H1,OR51T1,OR51A7,OR51G2,OR51G1,OR51A4,OR51A2,MMP26,OR51L1,OR52J3,OR52E2,OR52A5,OR52A1,OR52Z1,OR51V1,HBB,HBD,HBBP1,HBG1,HBG2,HBE1,OR51B4,OR51B2,OR51B5,OR51 | 0.0462067 | 314.402 | 5841 | 3563.92 |
| 1 | 19 | 34960072 | 35249362 | UBA2,WTIP,ZNF807,SCGB1B2P,SCGB2B2,ZNF302,ZNF181 | 0.0454655 | 320.992 | 45 | 25.3518 |
| 1 | 14 | 94741693 | 96752411 | PPP4R4,SERPINA10,SERPINA6,SERPINA2,SERPINA1,SERPINA11,SERPINA9,SERPINA12,SERPINA4,SERPINA5,SERPINA3,SERPINA13P,GSC,DICER1,DICER1,MIR3173,CLMN,SYNE3,SNHG10,SCARNA13,GLRX5,TCL6,TCL1B,TCL1A,C14orf132,BDKRB2,BDKRB1,ATG2B | 0.0447208 | 284.002 | 219 | 140.334 |
| 1 | 7 | 39247729 | 47894838 | POU6F2,YAE1D1,RALA,CDK13,MPLKIP,SUGCT,INHBA,INHBA,INHBA-AS1,GLI3,C7orf25,PSMA2,MRPL32,HECW1,HECW1,HECW1-IT1,MIR3943,HECW1,LUARIS,STK17A,STK17A,COA1,COA1,BLVRA,MRPS24,URGCP-MRPS24,URGCP-MRPS24,URGCP,UBE2D4,UBE2D4,POLR2J4,POLR2J4,RASA4CP,LINC00957,DBNL,PGAM2,POLM,MIR6838,POLM,AEBP1,AEBP1,MIR4649,POLD2,MYL7,GCK,YKT6,CAMK2B,NUDCD3,NPC1L1,DDX56,TMED4,OGDH,ZMIZ2,PPIA,H2AFV,PURB,MIR4657,PURB,MYO1G,SNHG15,SNHG15,SNORA9,CCM2,NACAD,TBRG4,TBRG4,SNORA5A,TBRG4,SNORA5C,TBRG4,SNORA5B,RAMP3,ADCY1,SEPT7P2,IGFBP1,IGFBP3,LOC730338,TNS3,C7orf65,PKD1L1,PKD1L1,C7orf69 | 0.0387841 | 282.197 | 781 | 467.095 |

| | | | | | | | | |
|---|---|---|---|---|---|---|---|---|
| 1 | 7 | 1.26E+08 | 128864451 | GRM8,GRM8,MIR592,ZNF800,GCC1,ARF5,FSCN3,PAX4,SND1,SND1,SND1-IT1,SND1,LRRC4,SND1,MIR593,MIR129-1,LEP,RBM28,PRRT4,IMPDH1,HILPDA,METTL2B,LINC01000,FAM71F2,FAM71F1,CALU,CALU,OPN1SW,OPN1SW,CCDC136,FLNC,FLNC,FLNC-AS1,ATP6V1F,LOC100130705,KCP,IRF5,TNPO3,LOC407835,TSPAN33,SMO | 0.0387721 | 288.62 | 429 | 264.522 |
| 1 | 12 | 1.24E+08 | 124414301 | MPHOSPH9,C12orf65,CDK2AP1,SBNO1,KMT5A,RILPL2,SNRNP35,RILPL1,MIR3908,TMED2,DDX55,EIF2B1,GTF2H3,TCTN2,ATP6V0A2,DNAH10 | 0.0373253 | 337.516 | 262 | 162.148 |
| 1 | 3 | 39322979 | 44283963 | CX3CR1,CCR8,SLC25A38,RPSA,RPSA,SNORA6,RPSA,SNORA62,MOBP,MYRIP,MYRIP,EIF1B-AS1,EIF1B,ENTPD3,ENTPD3,ENTPD3-AS1,RPL14,ZNF619,ZNF620,ZNF621,CTNNB1,ULK4,TRAK1,CCK,LYZL4,VIPR1,VIPR1,VIPR1-AS1,SEC22C,SEC22C,SS18L2,SEC22C,NKTR,NKTR,LOC101928323,ZBTB47,KLHL40,HHATL,CCDC13,CCDC13,CCDC13-AS1,HIGD1A,ACKR2,CYP8B1,ZNF662,KRBOX1,FAM198A,POMGNT2,SNRK,SNRK,SNRK-AS1,ANO10,ANO10,ABHD5,ABHD5,MIR138-1,TOPAZ1 | 0.0352872 | 272.236 | 443 | 270.992 |
| 1 | 9 | 27062612 | 33280799 | IFT74,TEK,EQTN,MOB3B,MOB3B,IFNK,C9orf72,LINGO2,LINGO2,MIR876,LINGO2,MIR873,ACO1,DDX58,TOPORS,TOPORS,SMIM27,SMIM27,SMIM27,NDUFB6,NDUFB6,TAF1L,TMEM215,APTX,DNAJA1,SMU1,B4GALT1,B4GALT1,B4GALT1-AS1,SPINK4,BAG1,CHMP5 | 0.0339944 | 316.73 | 273 | 164.269 |
| 1 | 10 | 42379857 | 51877836 | CCNYL2,ZNF33B,BMS1,MIR5100,RET,CSGALNACT2,RASGEF1A,FXYD4,HNRNPF,ZNF487,ZNF239,ZNF485,ZNF32-AS3,ZNF32,ZNF32-AS1,ZNF32-AS3,ZNF32,ZNF32-AS2,HNRNPA3P1,LINC00619,CXCL12,TMEM72-AS1,TMEM72,RASSF4,RASSF4,DEPP1,C10orf25,C10orf25,ZNF22,ZNF22,RSU1P2,ANKRD30BP3,MIR3156-1,OR13A1,ALOX5,ALOX5,LOC102724323,MARCH8,ZFAND4,WASHC2C,PTPN20,FAM35BP,LOC102724593,LOC102724593,SYT15,SYT15,GPRIN2,NPY4R,NPY4R2,ANXA8,FAM35DP,ANTXRL,ANXA8L1,ZNF488,RBP3,GDF2,GDF10,FRMPD2,MAPK8,ARHGAP22,WDFY4,WDFY4,LRRC18,MIR4294,VSTM4,FAM170B-AS1,FAM170B,C10orf128,C10orf71,DRGX,ERCC6,ERCC6,PGBD3,CHAT,SLC18A3,CHAT,C10orf53,OGDHL,PARG,PARG,PARGP1,TIMM23B,TIMM23B,MSMB,TIMM23B,NCOA4,TIMM23B,TIMM23,WASHC2A | 0.0328457 | 303.747 | 829 | 475.871 |

| | | | | | | | | |
|---|---|---|---|---|---|---|---|---|
| 1 | 9 | 10500 | 15468751 | CBWD1,C9orf66,C9orf66,DOCK8,DOCK8,KANK1,DMRT1,DMRT3,DMRT2,SMARCA2,VLDLR-AS1,VLDLR,VLDLR,KCNV2,PUM3,RFX3,GLIS3,GLIS3,GLIS3-AS1,SLC1A1,SPATA6L,SPATA6L,PLPP6,CDC37L1,AK3,RCL1,RCL1,MIR101-2,JAK2,INSL6,INSL4,RLN2,RLN1,PLGRKT,CD274,PDCD1LG2,RIC1,ERMP1,MLANA,KIAA2026,KIAA2026,MIR4665,RANBP6,IL33,TPD52L3,UHRF2,GLDC,KDM4C,DMAC1,PTPRD,TYRP1,TYRP1,LURAP1L-AS1,LURAP1L-AS1,LURAP1L,LURAP1L,MPDZ,LINC01235,LINC00583,NFIB,ZDHHC21,CER1,FREM1,LOC389705,TTC39B,SNAPC3,PSIP1 | 0.0319924 | 292.328 | 945 | 558.946 |
| 1 | 9 | 35957194 | 37426597 | OR2S2,RECK,GLIPR2,CCIN,CLTA,GNE,RNF38,MELK,MIR4475,PAX5,PAX5,MIR4540,PAX5,MIR4476,ZCCHC7,GRHPR | 0.0315767 | 281.762 | 166 | 95.6 |
| 1 | 2 | 95326671 | 96504630 | LOC442028,TEKT4,MAL,MRPS5,ZNF514,ZNF2,PROM2,KCNIP3,FAHD2A,TRIM43B,TRIM43 | 0.0304425 | 277.173 | 134 | 74.6194 |
| 1 | 13 | 95673757 | 96212753 | ABCC4,CLDN10 | 0.0297579 | 238.896 | 50 | 29.3798 |
| 1 | 2 | 71649970 | 73956505 | ZNF638,DYSF,CYP26B1,EXOC6B,SPR,EMX1,SFXN5,RAB11FIP5,NOTO,SMYD5,PRADC1,CCT7,FBXO41,EGR4,ALMS1,NAT8,ALMS1P1,NAT8B | 0.0286075 | 297.703 | 328 | 199.97 |
| 1 | 4 | 68566691 | 71067325 | UBA6,UBA6-AS1,GNRHR,TMPRSS11D,TMPRSS11A,TMPRSS11F,LOC550113,TMPRSS11F,LOC550113,SYT14P1,TMPRSS11F,FTLP10,TMPRSS11BNL,TMPRSS11BNL,TMPRSS11B,YTHDC1,TMPRSS11E,UGT2B17,UGT2B15,UGT2B10,UGT2A3,UGT2B7,LOC105377267,UGT2B11,UGT2B28,UGT2B4,UGT2A2,UGT2A1,UGT2A1,SULT1B1,SULT1E1,CSN1S1,CSN2,STATH,HTN3,HTN1,CSN1S2AP,CSN1S2BP,PRR27,ODAM | 0.0285445 | 364.37 | 284 | 150.842 |
| 1 | 21 | 40652727 | 42866610 | BRWD1,BRWD1-AS2,HMGN1,WRB,WRB-SH3BGR,WRB-SH3BGR,WRB,LCA5L,WRB-SH3BGR,SH3BGR,B3GALT5-AS1,B3GALT5,IGSF5,PCP4,DSCAM,DSCAM,MIR4760,MIR3197,BACE2,BACE2,PLAC4,FAM3B,MX2,MX1,TMPRSS2 | 0.0282134 | 281.031 | 209 | 122.663 |
| 1 | 20 | 47679727 | 47732483 | CSE1L,STAU1 | 0.0240034 | 369.465 | 28 | 18.6923 |
| 1 | 8 | 1812464 | 6599342 | ARHGEF10,KBTBD11,MYOM2,CSMD1,MCPH1,MCPH1,ANGPT2,MCPH1,MCPH1-AS1,AGPAT5 | 0.022916 | 254.804 | 246 | 145.054 |
| 1 | 4 | 1.71E+08 | 185550705 | CLCN3,HPF1,MFAP3L,AADAT,GALNTL6,LOC101930370,GALNT7,GALNT7,HMGB2,SAP30,SCRG1,HAND2,HAND2,HAND2-AS1,HAND2-AS1,FBXO8,CEP44,MIR4276,HPGD,GLRA3,ADAM29,GPM6A,GPM6A,LOC101928590,WDR17,SPATA4,ASB5,SPCS3,VEGFC,NEIL3,AGA,LINC01098,MIR1305,TENM3,DCTD,WWC2-AS2,WWC2,WWC2,WWC2-AS1,WWC2,CLDN22,CLDN24,CDKN2AIP,ING2,RWDD4,TRAPPC11,STOX2,ENPP6,IRF2,CASP3 | 0.0229038 | 273.259 | 561 | 337.545 |
| 1 | 2 | 84921381 | 86334415 | DNAH6,TRABD2A,TMSB10,KCMF1,TCF7L1,TGOLN2,RETSAT,ELMOD3,CAPG,SH2D6,MAT2A,GGCX,VAMP8,VAMP5,RNF181,TMEM150A,USP39,C2orf68,USP39,SFTPB,GNLY,ATOH8,ST3GAL5,POLR1A,PTCD3 | 0.0228894 | 288.939 | 278 | 169.935 |

| | | | | | | | | |
|---|---|---|---|---|---|---|---|---|
| 1 | 11 | 1.12E+08 | 118622147 | BCO2,PTS,PLET1,PLET1,LOC100132686,LOC101928847,NCAM1,NCAM1,NCAM1,NCAM1-AS1,TTC12,ANKK1,DRD2,DRD2,MIR4301,TMPRSS5,ZW10,CLDN25,USP28,HTR3B,HTR3A,ZBTB16,NNMT,C11orf71,RBM7,REXO2,NXPE1,NXPE4,NXPE2,CADM1,BUD13,ZPR1,APOA5,APOA4,APOC3,APOA1,APOA1,APOA1-AS,APOA1-AS,SIK3,SIK3,PAFAH1B2,SIDT2,SIDT2,LOC100652768,TAGLN,TAGLN,PCSK7,PCSK7,RNF214,BACE1,BACE1,BACE1-AS,CEP164,DSCAML1,FXYD2,FXYD6-FXYD2,FXYD6-FXYD2,FXYD6-FXYD2,FXYD6,TMPRSS13,IL10RA,SMIM35,SMIM35,TMPRSS4,TMPRSS4,SCN4B,SCN2B,JAML,MPZL3,MPZL2,CD3E,CD3D,CD3G,UBE4A,UBE4A,LOC100131626,LOC100131626,ATP5MG,KMT2A,KMT2A,LOC101929089,LOC101929089,TTC36,TTC36,TMEM25,TMEM25,TMEM25,IFT46,IFT46,ARCN1,PHLDB1,PHLDB1,MIR6716,TREH | 0.0210453 | 287.856 | 920 | 566.507 |
| 1 | 2 | 24905844 | 26432874 | NCOA1,PTRHD1,PTRHD1,CENPO,CENPO,CENPO,ADCY3,ADCY3,DNAJC27,EFR3B,POMC,DNMT3A,DNMT3A,MIR1301,DTNB,ASXL2,KIF3C,RAB10,GAREM2,HADHA | 0.0200361 | 282.476 | 250 | 149.263 |
| 1 | 3 | 5220234 | 15252429 | ARL8B,EDEM1,MIR4790,GRM7-AS3,GRM7,LMCD1-AS1,LMCD1,SSUH2,CAV3,OXTR,RAD18,SRGAP3,THUMPD3,SETD5,LHFPL4,MTMR14,CPNE9,BRPF1,OGG1,OGG1,CAMK1,CAMK1,TADA3,TADA3,ARPC4,ARPC4-TTLL3,ARPC4,ARPC4-TTLL3,ARPC4-TTLL3,TTLL3,RPUSD3,CIDEC,JAGN1,IL17RE,IL17RC,CRELD1,PRRT3,PRRT3,PRRT3-AS1,EMC3,EMC3,EMC3-AS1,FANCD2,FANCD2,FANCD2OS,FANCD2OS,BRK1,VHL,IRAK2,TATDN2,GHRLOS,LINC00852,GHRL,GHRLOS,GHRL,SEC13,ATP2B2,ATP2B2,MIR378B,ATP2B2,MIR885,SLC6A11,SLC6A1,SLC6A1-AS1,SLC6A1,HRH1,ATG7,ATG7,VGLL4,VGLL4,TAMM41,SYN2,SYN2,TIMP4,PPARG,TSEN2,MKRN2OS,MKRN2,RAF1,TMEM40,CAND2,RPL32,RPL32,SNORA7A,IQSEC1,NUP210,HDAC11,FBLN2,WNT7A,TPRXL,CHCHD4,TMEM43,XPC,LSM3,SLC6A6,GRIP2,CCDC174,C3orf20,FGD5,NR2C2,NR2C2,MRPS25,MRPS25,RBSN,COL6A4P1,CAPN7 | 0.0194524 | 282.149 | 1182 | 720.175 |
| 1 | 5 | 1.78E+08 | 180904760 | GMCL2,GMCL1P1,HNRNPAB,HNRNPAB,PHYKPL,PHYKPL,COL23A1,CLK4,ZNF354A,ZNF354B,ZFP2,ZNF454,GRM6,ZNF879,ZNF354C,ADAMTS2,RUFY1,RUFY1,LOC101928445,HNRNPH1,C5orf60,CBY3,CANX,MAML1,LTC4S,MGAT4B,MIR1229,MGAT4B,MGAT4B,SQSTM1,SQSTM1,SQSTM1,MRNIP,MRNIP,LOC100996419,TBC1D9B,RNF130,RNF130,MIR340,RASGEF1C,MAPK9,GFPT2,CNOT6,SCGB3A1,FLT4,OR2Y1,MGAT1,ZFP62,BTNL8,BTNL3,BTNL9,OR2V1,OR2V2,TRIM7,MIR4638,TRIM41,RACK1,RACK1,SNORD96A,RACK1,SNORD95,CTC-338M12.4,CTC-338M12.4,TRIM52,TRIM52 | 0.0190021 | 292.902 | 618 | 369.014 |

| | | | | | | | | |
|---|---|---|---|---|---|---|---|---|
| 1 | 16 | 46597745 | 56501955 | ANKRD26P1,SHCBP1,VPS35,ORC6,MYLK3,C16orf87,GPT2,DNAJA2,NETO2,ITFG1-AS1,ITFG1,ITFG1,PHKB,ABCC12,ABCC11,LONP2,LONP2,MIR5095,LONP2,MIR5095,SIAH1,MIR5095,SIAH1,MIR5095,N4BP1,CBLN1,C16orf78,ZNF423,CNEP1R1,HEATR3,PAPD5,ADCY7,ADCY7,MIR6771,BRD7,NKD1,SNX20,SNX20,LOC101927272,NOD2,CYLD,MIR3181,CYLD,LINC02168,SALL1,C16orf97,LINC00919,TOX3,LOC105371267,CHD9,LOC643802,RBL2,AKTIP,RPGRIP1L,FTO,LINC02169,IRX3,IRX5,IRX6,MMP2,LPCAT2,LPCAT2,CAPNS2,SLC6A2,CES1P1,CES1,CES5A,GNAO1,GNAO1,DKFZP434H168,GNAO1,MIR3935,AMFR,NUDT21,OGFOD1 | 0.0142057 | 300.079 | 911 | 540.522 |
| 1 | 2 | 2.39E+08 | 243188873 | UBE2F,UBE2F-SCLY,UBE2F-SCLY,SCLY,ESPNL,KLHL30,ERFE,ILKAP,LOC151174,LOC643387,HES6,PER2,TRAF3IP1,ASB1,TWIST2,HDAC4,HDAC4,MIR4440,HDAC4,MIR4269,HDAC4,MIR2467,LOC101928111,LOC150935,NDUFA10,NDUFA10,MIR4786,OR6B2,OR6B3,COPS9,OTOS,GPC1,GPC1,PP14571,GPC1,PP14571,MIR149,ANKMY1,ANKMY1,DUSP28,DUSP28,RNPEPL1,CAPN10,GPR35,AQP12B,LOC285191,AQP12A,KIF1A,AGXT,C2orf54,CROCC2,CROCC2,LOC200772,SNED1,SNED1,MTERF4,MTERF4,PASK,PASK,PPP1R7,PPP1R7,ANO7,HDLBP,SEPT2,FARP2,FARP2,MIR3133,STK25,BOK,THAP4,ATG4B,DTYMK,ING5,D2HGDH,GAL3ST2,NEU4,PDCD1,RTP5,LINC01237,LOC285095,LINC01237,LOC285097,LINC01881 | 0.013399 | 281.871 | 784 | 479.917 |
| 1 | 5 | 1.71E+08 | 175934469 | RANBP17,TLX3,MIR3912,NPM1,FGF18,SMIM23,FBXW11,STK10,EFCAB9,UBTD2,SH3PXD2B,NEURL1B,NEURL1B,MIR5003,DUSP1,ERGIC1,LOC100268168,RPL26L1,ATP6V0E1,ATP6V0E1,SNORA74B,CREBRF,BNIP1,NKX2-5,STC2,BOD1,CPEB4,C5orf47,NSG2,MSX2,MIR4634,DRD1,SFXN1,HRH2,CPLX2,THOC3,LOC100996385,FAM153B,SIMC1,KIAA1191,ARL10,ARL10,MIR1271,NOP16,NOP16,HIGD2A,HIGD2A,CLTB,FAF2 | 0.00992089 | 284.116 | 417 | 240.598 |
| 1 | 18 | 14851828 | 15381134 | ANKRD30B | 0.00673159 | 173.328 | 13 | 6.61769 |
| 1 | 5 | 72359596 | 82808241 | FCHO2,TMEM171,TMEM174,FOXD1,BTF3,ANKRA2,UTP15,ARHGEF28,ENC1,HEXB,HEXB,GFM2,GFM2,GFM2,NSA2,NSA2,FAM169A,GCNT4,ANKRD31,HMGCR,COL4A3BP,COL4A3BP,POLK,POLK,ANKDD1B,POC5,SV2C,IQGAP2,IQGAP2,F2RL2,F2R,F2RL1,S100Z,CRHBP,AGGF1,ZBED3,ZBED3,SNORA47,PDE8B,WDR41,OTP,TBCA,AP3B1,SCAMP1,LHFPL2,ARSB,DMGDH,BHMT2,BHMT,JMY,HOMER1,PAPD4,CMYA5,MTX3,THBS4,THBS4,CTD-2201I18.1,SERINC5,LOC644936,SPZ1,ZFYVE16,FAM151B,ANKRD34B,DHFR,DHFR,MTRNR2L2,DHFR,MSH3,MSH3,RASGRF2-AS1,RASGRF2,RASGRF2,RASGRF2,RNU5E-1,RNU5D-1,RNU5E-1,RNU5D-1,CKMT2,CKMT2-AS1,RNU5E-1,RNU5D-1,ZCCHC9,RNU5E-1,RNU5D-1,ACOT12,RNU5E-1,RNU5D-1,SSBP2,SSBP2,ATG10,RPS23,ATP6AP1L,MIR3977,TMEM167A,TMEM167A,SCARNA18,TMEM167A,XRCC4,XRCC4,VCAN | 0.00348903 | 298.358 | 1030 | 611.534 |

| | | | | | | | | |
|---|---|---|---|---|---|---|---|---|
| 1 | 8 | 77776514 | 82583277 | ZFHX4,PEX2,PKIA,ZC2HC1A,IL7,STMN2,HEY1,MRPS28,TPD52,MIR5708,ZBTB10,ZNF704,PAG1,FABP5,PMP2,FABP9,FABP4,FABP12,IMPA1 | 0.00178505 | 264.471 | 170 | 98.1472 |
| 1 | 5 | 66481687 | 68485610 | CD180,LINC02219,PIK3R1,SLC30A5,CCNB1,CENPH | -0.00229052 | 255.378 | 67 | 40.505 |
| 1 | 14 | 1.01E+08 | 107283157 | MEG8,SNORD114-29,MEG8,SNORD114-30,MEG8,SNORD114-31,MIR379,MIR411,MIR299,MIR380,MIR1197,MIR323A,MIR758,MIR494,MIR1193,MIR543,MIR495,MIR376C,MIR376A2,MIR654,MIR376B,MIR300,MIR1185-1,MIR1185-2,MIR381HG,MIR381,MIR381HG,MIR487B,MIR381HG,MIR539,MIR381HG,MIR889,MIR381HG,MIR544A,MIR381HG,MIR655,MIR487A,MIR382,MIR134,MIR668,MIR485,MIR323B,MIR154,MIR496,MIR377,MIR541,MIR409,MIR412,MIR369,MIR410,MIR656,DIO3OS,MIR1247,DIO3,LINC00239,PPP2R5C,DYNC1H1,HSP90AA1,HSP90AA1,WDR20,WDR20,MOK,ZNF839,CINP,TECPR2,ANKRD9,MIR4309,RCOR1,TRAF3,AMN,CDC42BPB,EXOC3L4,LINC00677,TNFAIP2,EIF5,EIF5,SNORA28,MARK3,CKB,TRMT61A,BAG5,APOPT1,KLC1,KLC1,XRCC3,XRCC3,ZFYVE21,PPP1R13B,LINC00637,ATP5MPL,TDRD9,TDRD9,RD3L,ASPG,MIR203A,MIR203B,KIF26A,C14orf144,C14orf180,TMEM179,MIR4710,INF2,ADSSL1,SIVA1,AKT1,ZBTB42,CEP170B,PLD4,AHNAK2,CLBA1,CDCA4,GPR132,JAG2,JAG2,MIR6765,NUDT14,BRF1,BRF1,BTBD6,BRF1,PACS2,PACS2,TEX22,MTA1,CRIP2,CRIP1,TEDC1,TMEM121,MIR4537,FAM30A,ADAM6,LINC00221,MIR5195 | -0.00236168 | 305.031 | 1217 | 715.48 |
| 1 | 2 | 70143178 | 71577454 | MXD1,ASPRV1,PCBP1-AS1,PCBP1,C2orf42,TIA1,PCYOX1,SNRPG,FAM136A,TGFA,ADD2,FIGLA,CLEC4F,CD207,VAX2,ATP6V1B1,ATP6V1B1,ATP6V1B1-AS1,ANKRD53,TEX261,NAGK,MCEE,MPHOSPH10,PAIP2B,ZNF638 | -0.00292787 | 287.047 | 216 | 130.505 |
| 1 | 18 | 72109194 | 78016748 | FAM69C,CNDP2,CNDP1,LINC00909,ZNF407,ZADH2,TSHZ1,SMIM21,ZNF516,C18orf65,ZNF236,MBP,GALR1,SALL3,ATP9B,NFATC1,LOC284241,CTDP1,KCNG2,PQLC1,HSBP1L1,TXNL4A,RBFA,ADNP2,PARD6G-AS1,PARD6G-AS1,PARD6G,PARD6G | -0.00609036 | 275.229 | 395 | 237.314 |
| 1 | 16 | 31511889 | 35193080 | C16orf58,AHSP,FRG2KP,YBX3P1,ZNF720,ZNF267,SLC6A10P | -0.00693073 | 280.161 | 199 | 96.8549 |
| 1 | 3 | 37584658 | 39184691 | ITGA9,ITGA9,ITGA9-AS1,CTDSPL,CTDSPL,MIR26A1,VILL,PLCD1,DLEC1,ACAA1,MYD88,OXSR1,SLC22A13,SLC22A14,XYLB,ACVR2B-AS1,ACVR2B,ACVR2B,EXOG,SCN5A,SCN10A,SCN11A,SCN11A,WDR48,WDR48,GORASP1,GORASP1,TTC21A,TTC21A,TTC21A,MIR6822,CSRNP1 | -0.00793134 | 300 | 410 | 253.451 |

| | | | | | | | | |
|---|---|---|---|---|---|---|---|---|
| 1 | 2 | 2.32E+08 | 238721727 | CAB39,ITM2C,GPR55,SPATA3,C2orf72,PSMD1,PSMD1,HTR2B,ARMC9,ARMC9,MIR4777,B3GNT7,NCL,NCL,SNORA75,NCL,SNORD20,NCL,SNORD82,LINC00471,NMUR1,TEX44,PTMA,PDE6D,COPS7B,MIR1471,NPPC,DIS3L2,ALPP,ALPPL2,ALPI,ECEL1,PRSS56,CHRND,CHRNG,TIGD1,TIGD1,MIR5001,EIF4E2,EIF4E2,EFHD1,GIGYF2,GIGYF2,KCNJ13,SNORC,NGEF,NGEF,LOC101928881,LOC101928881,NEU2,INPP5D,ATG16L1,ATG16L1,SCARNA5,ATG16L1,SCARNA6,SAG,DGKD,USP40,UGT1A8,UGT1A8,UGT1A10,UGT1A8,UGT1A10,UGT1A9,UGT1A8,UGT1A10,UGT1A9,UGT1A7,UGT1A8,UGT1A10,UGT1A9,UGT1A7,UGT1A6,UGT1A8,UGT1A10,UGT1A9,UGT1A7,UGT1A6,UGT1A5,UGT1A8,UGT1A10,UGT1A9,UGT1A7,UGT1A6,UGT1A5,UGT1A4,UGT1A8,UGT1A10,UGT1A9,UGT1A7,UGT1A6,UGT1A5,UGT1A4,UGT1A3,UGT1A8,UGT1A10,UGT1A9,UGT1A7,UGT1A6,UGT1A5,UGT1A4,UGT1A3,DNAJB3,UGT1A8,UGT1A10,UGT1A9,UGT1A7,UGT1A6,UGT1A5,UGT1A4,UGT1A3,LOC100286922,UGT1A8,UGT1A10,UGT1A9,UGT1A7,UGT1A6,UGT1A5,UGT1A4,UGT1A3,UGT1A1,MROH2A,HJURP,MSL3P1,TRPM8,SPP2,ARL4C,SH3BP4,AGAP1,GBX2,ASB18,IQCA1,ACKR3,COPS8,COL6A3,MLPH,MLPH,MIR6811,PRLH,RAB17,LRRFIP1 | -0.00809083 | 288.033 | 1003 | 600.14 |
| 1 | 17 | 59161753 | 60678248 | BCAS3,TBX2,C17orf82,TBX4,NACA2,BRIP1,INTS2,MED13,TBC1D3P2,EFCAB3,METTL2A,TLK2 | -0.0113624 | 347.564 | 214 | 122.543 |

| | | | | | | | | |
|---|---|---|---|---|---|---|---|---|
| 1 | 8 | 95802701 | 124787860 | INTS8,INTS8,CCNE2,CCNE2,NDUFAF6,TP53INP1,NDUFAF6,NDUFAF6,MIR3150BHG,MIR3150B,MIR3150A,PLEKHF2,C8orf37,C8orf37,C8orf37-AS1,GDF6,UQCRB,MTERF3,PTDSS1,LOC102724804,SDC2,CPQ,CPQ,LOC101927066,LOC101927066,TSPYL5,MTDH,LAPTM4B,MATN2,RPL30,RPL30,SNORA72,ERICH5,RIDA,POP1,NIPAL2,KCNS2,STK3,OSR2,VPS13B,VPS13B,MIR599,MIR875,COX6C,RGS22,RGS22,MIR1273A,FBXO43,POLR2K,SPAG1,RNF19A,ANKRD46,SNX31,PABPC1,YWHAZ,ZNF706,NACAP1,GRHL2,NCALD,MIR5680,RRM2B,UBR5,ODF1,KLF10,GASAL1,AZIN1,AZIN1-AS1,ATP6V1C1,LINC01181,BAALC-AS2,BAALC-AS2,BAALC,BAALC,MIR3151,BAALC,FZD6,CTHRC1,SLC25A32,SLC25A32,DCAF13,DCAF13,LOC105375690,RIMS2,RIMS2,DCSTAMP,DPYS,LRP12,ZFPM2,ZFPM2,ZFPM2-AS1,OXR1,ABRA,ANGPT1,RSPO2,EIF3E,EMC2,TMEM74,TRHR,NUDCD1,ENY2,PKHD1L1,EBAG9,SYBU,SYBU,LOC100132813,KCNV1,CSMD3,CSMD3,MIR2053,TRPS1,EIF3H,UTP23,RAD21,RAD21,RAD21-AS1,MIR3610,RAD21-AS1,AARD,SLC30A8,MED30,EXT1,SAMD12,SAMD12,SAMD12-AS1,TNFRSF11B,COLEC10,LOC101927513,COLEC10,MAL2,MAL2,MAL2-AS1,NOV,ENPP2,TAF2,DSCC1,DEPTOR,COL14A1,MRPL13,MTBP,SNTB1,HAS2,LINC01151,ZHX2,DERL1,TBC1D31,FAM83A,MIR4663,C8orf76,C8orf76,ZHX1-C8orf76,ZHX1-C8orf76,ZHX1,ATAD2,ATAD2,MIR548D1,WDYHV1,FBXO32,KLHL38,ANXA13,FAM91A1 | -0.0138171 | 295.532 | 1867 | 1100.11 |
| 1 | 4 | 1.56E+08 | 168155290 | FGA,FGG,LRAT,RBM46,NPY2R,MAP9,MAP9,LOC102724776,GUCY1A1,GUCY1B1,ASIC5,TDO2,CTSO,PDGFC,GLRB,GRIA2,FAM198B,FAM198B,FAM198B-AS1,TMEM144,RXFP1,C4orf46,ETFDH,PPID,FNIP2,FNIP2,C4orf45,C4orf45,RAPGEF2,FSTL5,FSTL5,LOC101928052,NAF1,NPY1R,NPY5R,TKTL2,TMA16,MARCH1,MARCH1,ANP32C,TRIM61,FAM218A,TRIM61,TRIM60,TMEM192,KLHL2,KLHL2,GK3P,MSMO1,CPE,CPE,MIR578,TLL1,SPOCK3 | -0.0142386 | 279.37 | 604 | 359.552 |

| | | | | | | | | |
|---|---|---|---|---|---|---|---|---|
| 1 | 17 | 6909198 | 7606861 | ALOX12-AS1,ALOX12,ALOX12-AS1,RNASEK,RNASEK-C17orf49,RNASEK-C17orf49,C17orf49,RNASEK-C17orf49,C17orf49,MIR497HG,MIR497HG,MIR195,MIR497HG,MIR497,BCL6B,SLC16A13,SLC16A11,CLEC10A,ASGR2,ASGR1,DLG4,DLG4,ACADVL,ACADVL,ACADVL,MIR324,DVL2,PHF23,GABARAP,CTDNEP1,ELP5,CLDN7,SLC2A4,YBX2,EIF5A,GPS2,NEURL4,ACAP1,KCTD11,TMEM95,TNK1,PLSCR3,TMEM256-PLSCR3,TMEM256-PLSCR3,TMEM256,NLGN2,SPEM1,SPEM2,TMEM102,FGF11,CHRNB1,ZBTB4,ZBTB4,SLC35G6,POLR2A,TNFSF12,TNFSF12-TNFSF13,TNFSF12-TNFSF13,TNFSF13,SENP3,SENP3-EIF4A1,SENP3-EIF4A1,EIF4A1,SENP3-EIF4A1,EIF4A1,SNORA48,SENP3-EIF4A1,EIF4A1,SNORD10,SENP3-EIF4A1,EIF4A1,SNORA67,CD68,LOC100996842,MPDU1,MPDU1,SOX15,FXR2,FXR2,SHBG,SHBG,SAT2,SHBG,ATP1B2,TP53,WRAP53 | -0.0143232 | 297.364 | 487 | 312.238 |
| 1 | 2 | 17997785 | 21258658 | MSGN1,KCNS3,RDH14,NT5C1B-RDH14,NT5C1B-RDH14,NT5C1B,MIR4757,OSR1,TTC32,WDR35,LOC101928222,MATN3,MATN3,LAPTM4A,SDC1,PUM2,RHOB,HS1BP3,GDF7,LDAH,APOB | -0.0151048 | 287.137 | 241 | 149.432 |
| 1 | 14 | 64915006 | 80669684 | MTHFD1,MTHFD1,ZBTB25,ZBTB25,AKAP5,ZBTB25,ZBTB1,LOC102723809,HSPA2,PPP1R36,PLEKHG3,SPTB,SPTB,MIR7855,CHURC1,CHURC1-FNTB,CHURC1-FNTB,GPX2,CHURC1-FNTB,CHURC1-FNTB,RAB15,CHURC1-FNTB,FNTB,CHURC1-FNTB,FNTB,MAX,CHURC1-FNTB,FNTB,MAX,MIR4706,MAX,MAX,LOC100506321,MIR4708,FUT8,FUT8-AS1,FUT8,MIR625,FUT8,CCDC196,GPHN,FAM71D,MPP5,ATP6V1D,EIF2S1,PLEK2,MIR5694,TMEM229B,PLEKHH1,PIGH,ARG2,ARG2,VTI1B,VTI1B,RDH11,RDH12,ZFYVE26,RAD51B,ZFP36L1,ACTN1,DCAF5,EXD2,GALNT16,ERH,ERH,SLC39A9,SLC39A9,PLEKHD1,CCDC177,SUSD6,LOC100289511,SRSF5,SRSF5,SLC10A1,SMOC1,SLC8A3,LOC646548,ADAM21P1,COX16,SYNJ2BP-COX16,SYNJ2BP-COX16,SYNJ2BP,ADAM21,ADAM20P1,ADAM20,MED6,TTC9,MAP3K9,PCNX1,SIPA1L1,RGS6,DPF3,DCAF4,ZFYVE1,RBM25,PSEN1,PAPLN,PAPLN,LOC101928123,NUMB,HEATR4,HEATR4,RIOX1,HEATR4,ACOT1,ACOT2,ACOT4,ACOT6,DNAL1,PNMA1,ELMSAN1,ELMSAN1,MIR4505,PTGR2,ZNF410,FAM161B,FAM161B,COQ6,COQ6,COQ6,ENTPD5,ENTPD5,BBOF1,BBOF1,ALDH6A1,ALDH6A1,LIN52,VSX2,ABCD4,VRTN,SYNDIG1L,NPC2,MIR4709,NPC2,ISCA2,LTBP2,AREL1,AREL1,FCF1,FCF1,YLPM1,PROX2,DLST,RPS6KL1,PGF,EIF2B2,MLH3,ACYP1,ZC2HC1C,NEK9,TMED10,FOS,JDP2,BATF,LOC102724153,FLVCR2,FLVCR2,ERG28,TTLL5,TGFB3,IFT43,GPATCH2L,ESRRB,VASH1,ANGEL1,LRRC74A,IRF2BPL,CIPC,ZDHHC22,TMEM63C,NGB,MIR1260A,NGB,POMT2,GSTZ1,TMED8,SAMD15,NOXRED1,VIPAS39,AHSA1,ISM2,SPTLC2,ALKBH1,ALKBH1,SLIRP,SLIRP,SLIRP,SNW1,SNW1,SNW1,C14orf178,C14orf178,ADCK1,NRXN3,DIO2 | -0.017572 | 278.856 | 2008 | 1220.38 |

| | | | | | | | | |
|---|---|---|---|---|---|---|---|---|
| 1 | 11 | 1.2E+08 | 134946016 | OAF,POU2F3,TMEM136,ARHGEF12,GRIK4,GRIK4,LOC105369532,GRIK4,LOC101929227,GRIK4,LOC101929208,TBCEL,TECTA,SC5D,SORL1,MIR100HG,MIR125B1,MIR100HG,BLID,MIR100HG,MIRLET7A2,MIR100HG,MIR100,MIR100HG,UBASH3B,CRTAM,JHY,BSX,HSPA8,CLMP,MIR4493,LOC100128242,GRAMD1B,SCN3B,ZNF202,OR6X1,OR6M1,TMEM225,OR8D4,OR4D5,OR6T1,OR10S1,OR10G4,OR10G9,OR10G8,OR10G7,VWA5A,OR10D3,OR8G2P,OR8G1,OR8G1,OR8G5,OR8D1,OR8D2,OR8B2,OR8B3,OR8B4,OR8B8,OR8B12,OR8A1,PANX3,TBRG1,SIAE,SIAE,SPA17,SPA17,NRGN,VSIG2,ESAM,ESAM,LOC101929340,MSANTD2,ROBO3,ROBO4,HEPN1,HEPACAM,HEPACAM,CCDC15,SLC37A2,TMEM218,PKNOX2,FEZ1,FEZ1,LOC403312,LOC403312,EI24,STT3A-AS1,STT3A-AS1,STT3A,CHEK1,CHEK1,ACRV1,ACRV1,PATE1,PATE2,PATE3,PATE4,HYLS1,PUS3,DDX25,CDON,RPUSD4,FAM118B,SRPRA,FOXRED1,TIRAP,DCPS,DCPS,GSEC,ST3GAL4,KIRREL3,KIRREL3,LOC101929427,KIRREL3-AS3,LOC101929473,ETS1,ETS1,LOC101929517,LOC101929538,FLI1,FLI1,SENCR,FLI1,KCNJ1,KCNJ5,C11orf45,KCNJ5,TP53AIP1,ARHGAP32,BARX2,TMEM45B,NFRKB,PRDM10,LINC00167,APLP2,ST14,ZBTB44,LOC646383,ADAMTS8,ADAMTS15,C11orf44,LINC02551,SNX19,NTM,OPCML,SPATA19,MIR4697HG,MIR4697,IGSF9B,JAM3,NCAPD3,VPS26B,THYN1,ACAD8,GLB1L3,GLB1L2,B3GAT1,LOC100507548 | -0.0196294 | 284.007 | 1471 | 906.09 |
| 1 | 3 | 52574400 | 56591389 | SMIM4,PBRM1,GNL3,GNL3,SNORD136,SNORD19,GNL3,SNORD19B,GNL3,SNORD69,GLT8D1,GLT8D1,SPCS1,SPCS1,NEK4,ITIH1,ITIH3,ITIH4,ITIH4,ITIH4-AS1,MUSTN1,TMEM110-MUSTN1,TMEM110-MUSTN1,TMEM110-MUSTN1,STIMATE,SFMBT1,RFT1,PRKCD,TKT,DCP1A,CACNA1D,CHDH,IL17RB,ACTR8,SELENOK,CACNA2D3,CACNA2D3,CACNA2D3-AS1,CACNA2D3,LRTM1,WNT5A,ERC2,ERC2,MIR3938,CCDC66 | -0.0201057 | 283.236 | 479 | 292.29 |
| 1 | 15 | 43350536 | 43813295 | UBR1,TMEM62,CCNDBP1,EPB42,TGM5,TGM7,LCMT2,LCMT2,ADAL,ADAL,ZSCAN29,TUBGCP4,TUBGCP4,TP53BP1,TP53BP1,MAP1A | -0.021042 | 318.46 | 181 | 113.164 |
| 1 | 2 | 86406652 | 92267022 | IMMT,IMMT,MIR4779,MRPL35,REEP1,KDM3A,CHMP3,RNF103-CHMP3,RNF103-CHMP3,RNF103,RNF103-CHMP3,RMND5A,RMND5A,CD8A,CD8B,ANAPC1P1,LOC285074,MIR4771-1,MIR4771-2,MIR4435-1,MIR4435-2,RGPD1,KRCC1,SMYD1,SMYD1,MIR4780,FABP1,THNSL2,FOXI3,TEX37,LOC101928371,EIF2AK3,EIF2AK3,RPIA,ANKRD36BP2,MIR4436A | -0.0216983 | 297.963 | 411 | 215.992 |
| 1 | 19 | 60500 | 608042 | OR4F17,PLPP2,MIER2,THEG,C2CD4C,SHC2,ODF3L2,MADCAM1,TPGS1,CDC34,GZMM,BSG,HCN2 | -0.0264298 | 283.136 | 107 | 62.3454 |
| 1 | 9 | 15509991 | 19058795 | PSIP1,CCDC171,C9orf92,BNC2,CNTLN,SH3GL2,ADAMTSL1,ADAMTSL1,MIR3152,SAXO1,RRAGA,HAUS6 | -0.0273032 | 260.092 | 228 | 131.89 |

| | | | | | | | | |
|---|---|---|---|---|---|---|---|---|
| 1 | 3 | 44362506 | 46874766 | TOPAZ1,TCAIM,ZNF445,ZNF852,ZKSCAN7,ZNF660,ZNF660-ZNF197,ZNF660-ZNF197,ZNF197,ZNF35,ZNF502,ZNF501,KIAA1143,KIF15,MIR564,TMEM42,TMEM42,TGM4,ZDHHC3,EXOSC7,CLEC3B,CDCP1,TMEM158,LARS2,LARS2,LARS2-AS1,LIMD1,SACM1L,SLC6A20,LZTFL1,LZTFL1,CCR9,FYCO1,FYCO1,CXCR6,XCR1,CCR1,CCR3,CCR2,LOC102724297,CCR5,CCRL2,LTF,RTP3,LRRC2,TDGF1,FAM240A,ALS2CL,TMIE,PRSS50,PRSS46P,PRSS45,PRSS42 | -0.0284108 | 272.949 | 487 | 302.936 |
| 1 | 12 | 9660033 | 20968171 | KLRB1,CLEC2D,CLECL1,CD69,KLRF1,CLEC2B,KLRF2,CLEC2A,CLEC12A-AS1,CLEC12A,CLEC12A,CLEC1B,CLEC12B,CLEC12B,LOC102724020,CLEC9A,CLEC1A,CLEC7A,OLR1,OLR1,TMEM52B,TMEM52B,GABARAPL1,KLRD1,LOC101928100,KLRK1,KLRC4-KLRK1,KLRC4-KLRK1,KLRC4,KLRC3,KLRC2,KLRC1,EIF2S3L,KLRA1P,MAGOHB,STYK1,YBX3,TAS2R7,TAS2R8,TAS2R9,TAS2R10,PRR4,PRH1-PRR4,PRH1-PRR4,PRH1,PRH1-PRR4,PRH1,TAS2R13,PRH1-PRR4,PRH1,PRH2,PRH1-PRR4,PRH1,PRH1-TAS2R14,TAS2R14,PRH1-PRR4,PRH1,PRH1-TAS2R14,PRH1-PRR4,PRH1,PRH1-TAS2R14,TAS2R50,PRH1-PRR4,PRH1,PRH1-TAS2R14,TAS2R20,PRH1-PRR4,PRH1,PRH1-TAS2R14,TAS2R19,PRH1-PRR4,PRH1,PRH1-TAS2R14,TAS2R31,PRH1-PRR4,PRH1,PRH1-TAS2R14,TAS2R46,PRH1-PRR4,PRH1,PRH1-TAS2R14,TAS2R43,PRH1-PRR4,PRH1,PRH1-TAS2R14,TAS2R30,TAS2R42,PRB3,PRB4,PRB1,PRB2,ETV6,BCL2L14,MIR1244-1,MIR1244-2,MIR1244-3,MIR1244-4,LRP6,MANSC1,LOH12CR2,LOH12CR2,BORCS5,BORCS5,DUSP16,CREBL2,GPR19,CDKN1B,APOLD1,APOLD1,MIR613,DDX47,RPL13AP20,GPRC5A,MIR614,GPRC5D-AS1,GPRC5D,GPRC5D-AS1,HEBP1,GPRC5D-AS1,HTR7P1,FAM234B,GSG1,EMP1,LINC01559,GRIN2B,ATF7IP,PLBD1,PLBD1-AS1,GUCY2C,GUCY2C,HIST4H4,H2AFJ,WBP11,C12orf60,SMCO3,C12orf60,ART4,MGP,ERP27,ARHGDIB,PDE6H,RERG,PTPRO,EPS8,STRAP,DERA,SLC15A5,MGST1,LMO3,SKP1P2,MIR3974,RERGL,PIK3C2G,PLCZ1,CAPZA3,PLEKHA5,AEBP2,PDE3A,SLCO1C1 | -0.0296471 | 305.587 | 1038 | 597.324 |
| 1 | 4 | 1.15E+08 | 123977011 | CAMK2D,ARSJ,UGT8,UGT8,MIR577,NDST4,TRAM1L1,NDST3,SNHG8,SNORA24,PRSS12,CEP170P1,METTL14,SEC24D,SYNPO2,MYOZ2,LOC101929762,USP53,C4orf3,FABP2,LOC645513,PDE5A,PDE5A,MAD2L1,PRDM5,NDNF,TNIP3,QRFPR,ANXA5,TMEM155,PP12613,EXOSC9,EXOSC9,CCNA2,CCNA2,BBS7,TRPC3,KIAA1109,ADAD1,IL2,IL21,IL21,IL21-AS1,BBS12,FGF2,FGF2,NUDT6,NUDT6,SPATA5 | -0.0308436 | 301.069 | 608 | 367.726 |

| | | | | | | | | |
|---|---|---|---|---|---|---|---|---|
| 1 | 9 | 72967111 | 79003656 | SMC5,KLF9,TRPM3,TRPM3,MIR204,TMEM2,ABHD17B,C9orf85,C9orf57,GDA,ZFAND5,TMC1,ALDH1A1,ANXA1,RORB-AS1,RORB,RORB,TRPM6,C9orf40,C9orf41-AS1,CARNMT1,CARNMT1,NMRK1,OSTF1,PCSK5,RFK | -0.0318259 | 281.884 | 392 | 230.121 |
| 1 | 19 | 11941375 | 12768991 | ZNF440,ZNF439,ZNF69,ZNF700,ZNF763,ZNF433-AS1,ZNF433,ZNF433-AS1,ZNF878,ZNF878,ZNF844,ZNF788,ZNF20,ZNF625-ZNF20,ZNF625-ZNF20,ZNF625-ZNF20,ZNF625,ZNF136,ZNF44,ZNF563,ZNF442,ZNF799,ZNF443,ZNF709,ZNF564,ZNF490,ZNF791,MAN2B1 | -0.0346416 | 343.286 | 270 | 151.032 |
| 1 | 15 | 79189824 | 99816216 | CTSH,RASGRF1,RASGRF1,LOC100129540,ANKRD34C-AS1,MIR184,ANKRD34C,TMED3,KIAA1024,MTHFS,ST20-MTHFS,ST20-MTHFS,ST20,ST20-MTHFS,ST20,ST20-AS1,ST20,ST20-AS1,BCL2A1,ZFAND6,FAH,ARNT2,ARNT2,LOC101929586,ARNT2,MIR5572,ABHD17C,CEMIP,MIR549A,CEMIP,CEMIP,MESD,MESD,MIR4514,TLNRD1,CFAP161,IL16,STARD5,TMC3-AS1,TMC3,MEX3B,LINC01583,EFL1,SAXO2,ADAMTS7P1,GOLGA6L10,UBE2Q2P2,GOLGA6L10,GOLGA6L17P,GOLGA6L9,RPS17,GOLGA6L10,GOLGA6L17P,GOLGA6L9,GOLGA6L10,GOLGA6L17P,GOLGA6L9,ADAMTS7P1,GOLGA6L17P,GOLGA6L9,UBE2Q2P2,RPS17,CPEB1,CPEB1,CPEB1-AS1,CPEB1-AS1,AP3B2,AP3B2,ACTG1P17,SNHG21,FSD2,SCARNA15,FSD2,WHAMM,HOMER2,FAM103A1,C15orf40,C15orf40,BTBD1,BTBD1,MIR4515,TM6SF1,HDGFL3,BNC1,SH3GL3,ADAMTSL3,EFL1P1,LOC440300,GOLGA2P7,ZSCAN2,SCAND2P,WDR73,NMB,SEC11A,ZNF592,ALPK3,SLC28A1,PDE8A,AKAP13,KLHL25,KLHL25,MIR1276,MIR548AP,AGBL1,AGBL1,LOC102724452,NTRK3,NTRK3,NTRK3-AS1,MRPL46,MRPS11,DET1,MIR1179,MIR7-2,MIR3529,AEN,ISG20,ACAN,HAPLN3,MFGE8,ABHD2,RLBP1,FANCI,FANCI,POLG,POLG,POLG,MIR6766,MIR9-3HG,MIR9-3,RHCG,TICRR,KIF7,PLIN1,PEX11A,WDR93,MESP1,MESP2,ANPEP,AP3S2,C15orf38-AP3S2,AP3S2,C15orf38-AP3S2,MIR5094,AP3S2,C15orf38-AP3S2,MIR5009,C15orf38-AP3S2,ARPIN,ZNF710,MIR3174,ZNF710,ZNF710,ZNF710-AS1,IDH2,IDH2,IDH2-DT,SEMA4B,CIB1,CIB1,GDPGP1,CIB1,TTLL13P,CIB1,NGRN,NGRN,GABARAPL3,ZNF774,IQGAP1,CRTC3,CRTC3, | -0.0392158 | 277.944 | 1894 | 1155.88 |
| 1 | 17 | 4574955 | 5042902 | PELP1,PELP1,LOC101559451,ARRB2,MED11,CXCL16,ZMYND15,TM4SF5,VMO1,GLTPD2,PSMB6,PLD2,MINK1,CHRNE,CHRNE,C17orf107,GP1BA,SLC25A11,RNF167,PFN1,ENO3,SPAG7,CAMTA2,INCA1,KIF1C,KIF1C,LOC102724009,SLC52A1,ZFP3,ZNF232,USP6 | -0.0407987 | 291.71 | 283 | 181.194 |
| 1 | 18 | 9134687 | 9235760 | ANKRD12 | -0.0411175 | 232.67 | 11 | 5.95674 |

| | | | | | | | | |
|---|---|---|---|---|---|---|---|---|
| 1 | 1 | 1.86E+08 | 200817711 | TPR,ODR4,ODR4,OCLM,LOC102724919,PDC,PTGS2,PLA2G4A,BRINP3,RGS18,RGS21,RGS1,RGS13,RGS2,UCHL5,UCHL5,TROVE2,TROVE2,GLRX2,CDC73,CDC73,MIR1278,CDC73,B3GALT2,KCNT2,KCNT2,MIR4735,CFH,CFHR3,CFHR1,CFHR4,CFHR2,CFHR5,F13B,ASPM,ZBTB41,CRB1,DENND1B,C1orf53,LHX9,NEK7,ATP6V1G3,PTPRC,MIR181A1HG,MIR181B1,MIR181A1,NR5A2,LINC00862,ZNF281,KIF14,DDX59,CAMSAP2 | -0.0423045 | 311.101 | 696 | 409.948 |
| 1 | 14 | 89344258 | 91925161 | FOXN3,FOXN3,FOXN3-AS1,EFCAB11,TDP1,KCNK13,PSMC1,NRDE2,CALM1,TTC7B,TTC7B,LOC101928909,TTC7B,LOC105370622,RPS6KA5,RPS6KA5,DGLUCY,DGLUCY,SNORA11B,DGLUCY,GPR68,CCDC88C,PPP4R3A | -0.0462215 | 242.963 | 264 | 156.225 |
| 1 | 5 | 49555074 | 66458567 | EMB,PARP8,ISL1,LINC02118,PELO,ITGA1,ITGA1,ITGA2,MOCS2,FST,NDUFS4,ARL15,ARL15,MIR581,ARL15,MIR4459,HSPB3,SNX18,ESM1,GZMK,GZMA,CDC20B,CDC20B,GPX8,CDC20B,MIR449A,MIR449B,CDC20B,MIR449C,MCIDAS,CCNO,DHX29,MTREX,MTREX,PLPP1,PLPP1,PLPP1,MIR5687,PLPP1,RNF138P1,SLC38A9,DDX4,IL31RA,IL6ST,FLJ31104,ANKRD55,LINC01948,C5orf67,MAP3K1,SETD9,SETD9,MIER3,MIER3,GPBP1,ACTBL2,PLK2,GAPT,MIR548AE2,LINC02108,RAB3C,PDE4D,DEPDC1B,ELOVL7,ERCC8,NDUFAF2,SMIM15,ZSWIM6,C5orf64,C5orf64,LOC101928651,KIF2A,DIMT1,IPO11,IPO11,LRRC70,IPO11-LRRC70,IPO11,IPO11-LRRC70,HTR1A,RNF180,RGS7BP,SHISAL2B,SREK1IP1,CWC27,ADAMTS6,CENPK,PPWD1,TRIM23,TRAPPC13,SGTB,NLN,ERBIN,SREK1,MAST4 | -0.0508003 | 283.167 | 1122 | 651.185 |
| 1 | 11 | 73120456 | 82966983 | FAM168A,PLEKHB1,RAB6A,MRPL48,COA4,PAAF1,DNAJB13,UCP2,UCP3,C2CD3,PPME1,P4HA3,P4HA3,P4HA3-AS1,PGM2L1,MIR548AL,KCNE3,LIPT2,LIPT2,LOC100287896,LOC100287896,POLD3,CHRDL2,CHRDL2,MIR4696,RNF169,XRRA1,SPCS2,NEU3,OR2AT4,SLCO2B1,TPBGL,ARRB1,ARRB1,MIR326,RPS3,RPS3,SNORD15A,RPS3,SNORD15B,KLHL35,GDPD5,SERPINH1,LOC105369391,MAP6,MAP6,MOGAT2,DGAT2,UVRAG,WNT11,THAP12,GVQW3,EMSY,LRRC32,GUCY2EP,TSKU,ACER3,B3GNT6,CAPN5,CAPN5,OMP,MYO7A,GDPD4,PAK1,AQP11,CLNS1A,RSF1,AAMDC,AAMDC,INTS4,INTS4,KCTD14,NDUFC2-KCTD14,NDUFC2-KCTD14,THRSP,NDUFC2-KCTD14,NDUFC2,ALG8,KCTD21-AS1,KCTD21,KCTD21,USP35,GAB2,NARS2,TENM4,TENM4,MIR708,TENM4,MIR5579,MIR4300HG,MIR4300,FAM181B,PRCP,DDIAS,RAB30,RAB30,SNORA70E,PCF11,PCF11,LOC100506282,ANKRD42 | -0.0538995 | 262.611 | 1027 | 611.062 |
| 1 | 5 | 1.66E+08 | 170323223 | TENM2,WWC1,RARS,FBLL1,PANK3,PANK3,MIR103A1,MIR103B1,SLIT3,SLIT3,LOC101927969,SLIT3,MIR218-2,SLIT3,MIR585,SPDL1,DOCK2,DOCK2,FAM196B,DOCK2,MIR378E,FOXI1,C5orf58,C5orf58,LCP2,LCP2,KCNIP1,KCNIP1,KCNMB1,GABRP,RANBP17 | -0.0545448 | 262.584 | 348 | 208.729 |

| | | | | | | | | |
|---|---|---|---|---|---|---|---|---|
| 1 | 9 | 80030815 | 88934648 | VPS13A,GNA14,GNAQ,CEP78,PSAT1,TLE4,TLE1,SPATA31D5P,SPATA31D4,SPATA31D3,SPATA31D1,RASEF,FRMD3,IDNK,UBQLN1,LOC105376114,GKAP1,KIF27,C9orf64,HNRNPK,HNRNPK,MIR7-1,RMI1,SLC28A3,NTRK2,AGTPBP1,LOC389765,NAA35,GOLM1,C9orf153,ISCA1,ZCCHC6 | -0.0565552 | 297.734 | 533 | 303.411 |
| 1 | 12 | 1.01E+08 | 105570002 | SCYL2,SLC17A8,NR1H4,GAS2L3,ANO4,SLC5A8,UTP20,ARL1,SPIC,MYBPC1,CHPT1,CHPT1,SYCP3,SYCP3,GNPTAB,DRAM1,WASHC3,NUP37,PARPBP,PARPBP,PMCH,IGF1,PAH,PAH,ASCL1,ASCL1,LOC101929058,C12orf42,STAB2,NT5DC3,TTC41P,HSP90B1,MIR3652,HSP90B1,C12orf73,TDG,GLT8D2,HCFC2,NFYB,TXNRD1,TXNRD1,EID3,CHST11,CHST11,MIR3922,SLC41A2,C12orf45,ALDH1L2,WASHC4,APPL2 | -0.0566452 | 304.022 | 715 | 427.374 |
| 1 | 11 | 1.11E+08 | 112013780 | C11orf53,COLCA1,COLCA2,MIR4491,POU2AF1,BTG4,LOC728196,MIR34B,LOC728196,MIR34C,C11orf88,LAYN,SIK2,SIK2,PPP2R1B,PPP2R1B,ALG9,FDXACB1,C11orf1,CRYAB,CRYAB,HSPB2,HSPB2-C11orf52,HSPB2,HSPB2-C11orf52,HSPB2-C11orf52,HSPB2-C11orf52,C11orf52,DIXDC1,DLAT,DLAT,PIH1D2,PIH1D2,NKAPD1,TIMM8B,TIMM8B,SDHD,SDHD | -0.0578056 | 283.638 | 211 | 126.38 |
| 1 | 1 | 89435736 | 91406853 | KYAT3,RBMXL1,GBP3,GBP1,GBP2,GBP7,GBP4,GBP5,GBP6,GBP1P1,LRRC8B,LRRC8C,LRRC8D,ZNF326,BARHL2,ZNF644 | -0.0668798 | 313.162 | 196 | 117.646 |
| 1 | 1 | 94012376 | 94643709 | FNBP1L,BCAR3,BCAR3,LOC100129046,BCAR3,MIR760,DNTTIP2,GCLM,ABCA4,ARHGAP29 | -0.0677869 | 292.796 | 116 | 71.9143 |
| 1 | 12 | 88589908 | 100676960 | KITLG,DUSP6,POC1B,POC1B,GALNT4,POC1B-GALNT4,POC1B,POC1B-GALNT4,POC1B,POC1B-GALNT4,POC1B-AS1,ATP2B1,LINC00615,CCER1,EPYC,KERA,LUM,DCN,LINC01619,BTG1,CLLU1OS,CLLU1OS,CLLU1,C12orf74,PLEKHG7,EEA1,LOC643339,LOC643339,LINC02412,NUDT4,NUDT4P2,NUDT4B,UBE2N,MRPL42,SOCS2-AS1,SOCS2,SOCS2,CRADD,PLXNC1,CEP83,MIR5700,TMCC3,MIR492,MIR492,KRT19P2,NDUFA12,NR2C1,FGD6,VEZT,MIR331,MIR3685,METAP2,USP44,PGAM1P5,NTN4,NTN4,LOC105369921,LINC02410,SNRPF,CCDC38,AMDHD1,HAL,LTA4H,ELK3,CDK17,CFAP54,NEDD1,RMST,MIR1251,MIR135A2,MIR4495,MIR4303,SLC9A7P1,LINC02453,TMPO-AS1,TMPO,TMPO,SLC25A3,SLC25A3,SNORA53,IKBIP,APAF1,ANKS1B,ANKS1B,FAM71C,UHRF1BP1L,GOLGA2P5,ACTR6,ACTR6,DEPDC4,DEPDC4,SCYL2 | -0.0712737 | 277.364 | 898 | 530.548 |

| | | | | | | | | |
|---|---|---|---|---|---|---|---|---|
| 1 | 4 | 1.26E+08 | 145916699 | MIR2054,INTU,SLC25A31,HSPA4L,PLK4,MFSD8,ABHD18,LARP1B,PGRMC2,JADE1,SCLT1,C4orf33,PCDH10,PABPC4L,PCDH18,SLC7A11-AS1,SLC7A11,SLC7A11,NOCT,ELF2,MGARP,NDUFC1,NDUFC1,NAA15,NAA15,RAB33B,SETD7,MGST2,MGST2,MAML3,MAML3,MAML3,LOC101927516,SCOC,SCOC-AS1,SCOC,CLGN,MGAT4D,ELMOD2,UCP1,TBC1D9,RNF150,ZNF330,IL15,INPP4B,USP38,GAB1,GAB1,MIR3139,SMARCA5,SMARCA5-AS1,SMARCA5,FREM3,GYPE,GYPB,GYPA,HHIP,ANAPC10 | -0.0740438 | 268.92 | 793 | 464.929 |
| 1 | 15 | 25616042 | 34434095 | SNHG14,UBE3A,ATP10A,ATP10A,MIR4715,LINC02346,GABRB3,GABRA5,GABRG3,OCA2,HERC2,GOLGA8F,GOLGA8G,MIR4509-1,MIR4509-2,MIR4509-3,HERC2P9,GOLGA8M,WHAMMP2,PDCD6IPP2,APBA2,FAM189A1,FAM189A1,NSMCE3,TJP1,LINC02249,CHRFAM7A,GOLGA8R,ARHGAP11B,LOC100288637,HERC2P10,FAN1,FAN1,MTMR10,MTMR10,TRPM1,TRPM1,MIR211,LINC02352,LOC283710,LOC283710,KLF13,OTUD7A,CHRNA7,ARHGAP11A,SCG5,GREM1,LOC100131315,GREM1,FMN1,RYR3,AVEN,AVEN,CHRM5,CHRM5,EMC7,PGBD4 | -0.0747527 | 279.995 | 781 | 455.122 |
| 1 | 3 | 1.84E+08 | 184541859 | EIF2B5,DVL3,AP2M1,ABCF3,VWA5B2,VWA5B2,MIR1224,ALG3,EEF1AKMT4,EEF1AKMT4-ECE2,EEF1AKMT4-ECE2,CAMK2N2,EEF1AKMT4-ECE2,ECE2,PSMD2,EIF4G1,EIF4G1,SNORD66,FAM131A,CLCN2,POLR2H,THPO,CHRD,LINC02054,EPHB3,MAGEF1 | -0.076223 | 267.052 | 263 | 171.448 |
| 1 | 12 | 44197968 | 47178980 | TWF1,TMEM117,NELL2,DBX2,PLEKHA8P1,ANO6,LINC00938,ARID2,SCAF11,SLC38A1,SLC38A2,SLC38A4 | -0.0811378 | 288.844 | 229 | 136.318 |
| 1 | 5 | 10254219 | 37000711 | CCT5,CMBL,MARCH6,ROPN1L,LINC02213,ANKRD33B,DAP,CTNND2,DNAH5,TRIO,FAM105A,OTULIN,ANKH,ANKH,LOC100130744,ANKH,MIR4637,FBXL7,FBXL7,MIR887,MARCH11,ZNF622,RETREG1,RETREG1,LOC101929524,MYO10,BASP1,CDH18,GUSBP1,CDH12,PRDM9,C5orf17,CDH10,LINC02228,CDH9,CDH6,DROSHA,C5orf22,PDZD2,PDZD2,MIR4279,GOLPH3,MTMR12,ZFR,ZFR,MIR579,SUB1,NPR3,TARS,ADAMTS12,RXFP3,SLC45A2,AMACR,C1QTNF3-AMACR,C1QTNF3-AMACR,C1QTNF3,RAI14,TTC23L,RAD1,BRIX1,DNAJC21,AGXT2,PRLR,SPEF2,IL7R,CAPSL,UGT3A1,UGT3A2,LMBRD2,LMBRD2,MIR580,SKP2,NADK2,RANBP3L,SLC1A3,NIPBL | -0.0848532 | 259.252 | 1276 | 765.34 |
| 1 | 2 | 1.19E+08 | 120619558 | DDX18,CCDC93,INSIG2,EN1,MARCO,C1QL2,STEAP3,STEAP3,STEAP3-AS1,C2orf76,DBI,TMEM37,SCTR,SCTR,LOC107105282,CFAP221,TMEM177,PTPN4 | -0.0849435 | 255.795 | 170 | 101.686 |

| | | | | | | | | |
|---|---|---|---|---|---|---|---|---|
| 1 | 15 | 20044632 | 25583741 | CHEK2P2,HERC2P3,NBEAP1,POTEB,POTEB2,POTEB3,MIR5701-1,MIR5701-2,MIR5701-3,FAM30C,LOC101927079,LINC02203,LOC101927079,LINC02203,OR4M2,LOC101927079,OR4N4,OR4N3P,IGHV1OR15-1,LOC102724760,IGHV1OR15-3,LOC642131,REREP3,TUBGCP5,CYFIP1,NIPA2,NIPA1,WHAMMP3,GOLGA8IP,HERC2P2,GOLGA6L2,MIR4508,MKRN3,MAGEL2,NDN,NPAP1,SNRPN,SNHG14,SNURF,SNHG14,SNHG14,SNORD107,PWARSN,SNHG14,PWAR5,SNORD64,SNHG14,PWAR5,SNORD108,SNHG14,SNORD109B,SNORD109A,SNHG14,SNORD116-1,SNHG14,SNORD116-2,SNHG14,SNORD116-3,SNORD116-9,SNHG14,SNORD116-4,SNHG14,SNORD116-5,SNORD116-7,SNHG14,SNORD116-2,SNORD116-6,SNHG14,SNORD116-8,SNHG14,SNORD116-10,SNHG14,SNORD116-11,SNHG14,SNORD116-12,SNHG14,SNORD116-13,SNHG14,SNORD116-14,SNHG14,SNORD116-19,SNORD116-17,SNHG14,SNORD116-22,SNHG14,SNORD116-23,SNHG14,SNORD116-24,SNHG14,SNORD116-25,SNHG14,SNORD116-26,SNHG14,SNORD116-27,SNHG14,SNORD116-28,SNHG14,SNORD116-29,SNHG14,SNORD115-1,SNHG14,SNORD115-5,SNORD115-9,SNORD115-10,SNORD115-12,SNHG14,SNORD115-6,SNHG14,SNORD115-7,SNHG14,SNORD115-8,SNHG14,SNORD115-9,SNORD115-10,SNORD115-12,SNORD115-5,SNHG14,SNORD115-10,SNHG14,SNORD115-13,SNHG14,SNORD115-14,SNHG14,SNORD115-16,SNHG14,SNORD115-17,SNORD115-18,SNORD115-19,SNHG14,SNORD115-20,SNORD115-15,SNORD115-21,SNHG14,SNORD115-15,SNORD115- | -0.0857726 | 354.155 | 364 | 188.29 |
| 1 | 5 | 1.59E+08 | 161576345 | IL12B,ADRA1B,TTC1,PWWP2A,FABP6,CCNJL,C1QTNF2,ZBED8,SLU7,PTTG1,MIR3142HG,MIR3142HG,MIR146A,ATP10B,GABRB2,GABRA6,GABRA1,GABRG2 | -0.0863269 | 267.588 | 198 | 120.993 |
| 1 | 15 | 1E+08 | 102520892 | MEF2A,LYSMD4,DNM1P46,LOC400464,ADAMTS17,SPATA41,CERS3-AS1,CERS3,CERS3,LINS1,ASB7,ALDH1A3,ALDH1A3,LOC101927751,LRRK1,CHSY1,SELENOS,SNRPA1,PCSK6,PCSK6,LOC100507472,TM2D3,TARSL2,LOC100128108,OR4F6,OR4F15,OR4F13P,OR4F4 | -0.0878727 | 271.382 | 304 | 175.674 |
| 1 | 1 | 1.11E+08 | 115398253 | KCNA3,CD53,LRIF1,DRAM2,CEPT1,DENND2D,CHI3L2,CHIAP2,CHIA,PIFO,OVGP1,WDR77,WDR77,ATP5PB,ATP5PB,C1orf162,TMIGD3,TMIGD3,ADORA3,RAP1A,FAM212B,FAM212B,DDX20,DDX20,KCND3,LINC01750,CTTNBP2NL,MIR4256,WNT2B,ST7L,CAPZA1,MOV10,RHOC,PPM1J,FAM19A3,SLC16A1,LRIG2,MAGI3,PHTF1,RSBN1,AP4B1-AS1,PTPN22,AP4B1-AS1,BCL2L15,AP4B1-AS1,AP4B1,AP4B1,AP4B1,DCLRE1B,DCLRE1B,HIPK1-AS1,HIPK1,HIPK1,OLFML3,SYT6,TRIM33,BCAS2,DENND2C,AMPD1,NRAS,CSDE1,SIKE1,SYCP1 | -0.0879119 | 285.095 | 597 | 368.536 |

| | | | | | | | | |
|---|---|---|---|---|---|---|---|---|
| 1 | 17 | 7762706 | 8273523 | NAA38,CYB5D1,NAA38,CHD3,CHD3,SCARNA21,LINC02581,KCNAB3,TRAPPC1,CNTROB,GUCY2D,ALOX15B,ALOX12B,MIR4314,ALOXE3,HES7,PER1,VAMP2,TMEM107,MIR4521,BORCS6,AURKB,CTC1,PFAS,SLC25A35,RANGRF,SLC25A35,ARHGEF15,ODF4,LOC100128288,KRBA2 | -0.0893699 | 259.521 | 305 | 195.435 |
| 1 | 3 | 49227306 | 49413046 | C3orf84,CCDC36,C3orf62,C3orf62,MIR4271,USP4,GPX1,RHOA | -0.0910503 | 276.024 | 62 | 36.9533 |
| 1 | 12 | 59307696 | 76443125 | LRIG3,SLC16A7,FAM19A2,USP15,MIR6125,USP15,MON2,LINC01465,MIRLET7I,PPM1H,AVPR1A,DPY19L2,TMEM5,TMEM5,TMEM5-AS1,SRGAP1,C12orf66,C12orf56,XPOT,TBK1,RASSF3,RASSF3,MIR548C,MIR548Z,GNS,TBC1D30,WIF1,LEMD3,MSRB3,LOC100507065,RPSAP52,RPSAP52,HMGA2,HMGA2,HMGA2,LOC100129940,LLPH,TMBIM4,IRAK3,HELB,GRIP1,CAND1,DYRK2,IFNG,IL26,IL22,MDM1,RAP1B,SNORA70G,RAP1B,NUP107,SLC35E3,MDM2,CPM,CPSF6,CPSF6,MIR1279,LYZ,YEATS4,FRS2,CCT2,LRRC10,BEST3,RAB3IP,MYRFL,CNOT2,KCNMB4,PTPRB,PTPRR,TSPAN8,LGR5,ZFC3H1,THAP2,TMEM19,RAB21,TBC1D15,TPH2,TRHDE-AS1,TRHDE-AS1,TRHDE,TRHDE,ATXN7L3B,KCNC2,CAPS2,CAPS2,GLIPR1L1,GLIPR1L2,GLIPR1,GLIPR1,KRR1,KRR1,PHLDA1,NAP1L1 | -0.0935952 | 278.867 | 1156 | 682.745 |
| 1 | 14 | 92585248 | 94704007 | NDUFB1,CPSF2,SLC24A4,RIN3,LGMN,GOLGA5,CHGA,ITPK1,MOAP1,TMEM251,GON7,UBR7,BTBD7,UNC79,COX8C,UNC79,PRIMA1,FAM181A-AS1,FAM181A,FAM181A,ASB2,ASB2,MIR4506,CCDC197,OTUB2,DDX24,IFI27L1,IFI27,IFI27L2,PPP4R4 | -0.0940679 | 268.849 | 347 | 205.781 |
| 1 | 2 | 1.21E+08 | 128928941 | EPB41L5,TMEM185B,RALB,INHBB,LINC01101,GLI2,TFCP2L1,CLASP1,NIFK-AS1,NIFK,NIFK,TSN,LOC107985820,CNTNAP5,GYPC,TEX51,BIN1,CYP27C1,ERCC3,MAP3K2,PROC,PROC,MIR4783,IWS1,MYO7B,MYO7B,LOC105373609,LIMS2,LIMS2,GPR17,SFT2D3,WDR33,POLR2D,AMMECR1L,SAP130,UGGT1 | -0.0950283 | 257.808 | 535 | 323.926 |
| 1 | 15 | 76146675 | 79168994 | UBE2Q2,FBXO22,FBXO22,FBXO22-AS1,NRG4,TMEM266,TMEM266,LOC101929439,ETFA,ISL2,SCAPER,SCAPER,MIR3713,RCN2,PSTPIP1,TSPAN3,PEAK1,HMG20A,LINGO1,LINGO1,LINGO1-AS1,LOC645752,TBC1D2B,SH2D7,CIB2,IDH3A,ACSBG1,DNAJA4,WDR61,CRABP1,IREB2,HYKK,PSMA4,CHRNA5,CHRNA5,CHRNA3,CHRNA3,CHRNB4,LOC646938,ADAMTS7,MORF4L1 | -0.0965523 | 262.971 | 441 | 253.099 |
| 1 | 1 | 43887674 | 45811281 | SZT2,SZT2,SZT2-AS1,SZT2,SZT2-AS1,MIR6735,SZT2,HYI,PTPRF,KDM4A,KDM4A,KDM4A-AS1,ST3GAL3,ARTN,IPO13,DPH2,ATP6V0B,B4GALT2,CCDC24,SLC6A9,KLF17,DMAP1,ERI3,RNF220,RNF220,MIR5584,TMEM53,C1orf228,KIF2C,KIF2C,SNORD160,RPS8,RPS8,SNORD55,RPS8,SNORD46,RPS8,SNORD38A,RPS8,SNORD38B,BEST4,PLK3,PLK3,TCTEX1D4,TCTEX1D4,BTBD19,PTCH2,EIF2B3,HECTD3,UROD,ZSWIM5,HPDL,MUTYH,MUTYH,TOE1,TOE1,TESK2 | -0.0976626 | 278.319 | 485 | 310.587 |

| | | | | | | | | |
|---|---|---|---|---|---|---|---|---|
| 1 | 3 | 57902627 | 90311186 | SLMAP,FLNB,FLNB,FLNB-AS1,DNASE1L3,ABHD6,HTD2,RPP14,PXK,PDHB,KCTD6,ACOX2,FAM107A,FAM107A,FAM3D-AS1,FAM3D-AS1,FAM3D,FAM3D,C3orf67,C3orf67,C3orf67-AS1,FHIT,PTPRG,PTPRG,PTPRG-AS1,C3orf14,FEZF2,CADPS,SYNPR,SYNPR,SYNPR-AS1,SNTN,C3orf49,C3orf49,THOC7,THOC7,ATXN7,PSMD6-AS2,PSMD6,PSMD6,PRICKLE2-AS1,PRICKLE2,PRICKLE2,PRICKLE2,PRICKLE2-AS3,ADAMTS9,ADAMTS9,ADAMTS9-AS1,ADAMTS9,ADAMTS9-AS2,MAGI1,SLC25A26,LRIG1,KBTBD8,MIR4272,SUCLG2,FAM19A1,FAM19A4,EOGT,TMF1,TMF1,MIR3136,UBA3,ARL6IP5,LMOD3,FRMD4B,MITF,FOXP1,FOXP1,MIR1284,EIF4E3,EIF4E3,GPR27,GPR27,PROK2,RYBP,SHQ1,GXYLT2,PPP4R2,PPP4R2,EBLN2,PDZRN3,PDZRN3,LOC101927296,PDZRN3,PDZRN3-AS1,CNTN3,MIR1324,FRG2C,FRG2EP,ZNF717,ZNF717,MIR4273,ROBO2,ROBO1,ROBO1,MIR3923,GBE1,CADM2,CADM2,MIR5688,CADM2,CADM2-AS2,VGLL3,MIR4795,CHMP2B,POU1F1,HTR1F,CGGBP1,CGGBP1,ZNF654,C3orf38,EPHA3 | -0.0979219 | 237.505 | 1296 | 763.659 |
| 1 | 6 | 1.5E+08 | 171054567 | PPIL4,GINM1,KATNA1,LATS1,LATS1,LOC645967,NUP43,PCMT1,LRP11,LRP11,RAET1E-AS1,RAET1E-AS1,RAET1E,RAET1E-AS1,RAET1G,RAET1G,ULBP2,ULBP1,RAET1K,RAET1L,ULBP3,PPP1R14C,IYD,PLEKHG1,MTHFD1L,AKAP12,ZBTB2,RMND1,ARMT1,CCDC170,ESR1,ESR1,SYNE1,SYNE1,SYNE1,MIR3163,SYNE1,SYNE1-AS1,MYCT1,VIP,FBXO5,MTRF1L,RGS17,OPRM1,OPRM1,IPCEF1,IPCEF1,CNKSR3,SCAF8,MIR1273C,TIAM2,TIAM2,TFB1M,TFB1M,TFB1M,CLDN20,NOX3,MIR1202,ARID1B,ARID1B,MIR4466,TMEM242,ZDHHC14,ZDHHC14,MIR3692,SNX9,SYNJ2,SERAC1,GTF2H5,TULP4,TMEM181,DYNLT1,SYTL3,SYTL3,MIR3918,EZR,EZR,EZR-AS1,OSTCP1,C6orf99,RSPH3,TAGAP,LOC101929122,FNDC1,FNDC1,SOD2,SOD2,WTAP,SOD2,SOD2-OT1,SOD2,SOD2-OT1,ACAT2,ACAT2,ACAT2,TCP1,TCP1,TCP1,SNORA20,TCP1,SNORA29,MRPL18,PNLDC1,MAS1,IGF2R,SLC22A1,SLC22A2,SLC22A3,LPAL2,LPA,PLG,MAP3K4,AGPAT4,AGPAT4,AGPAT4-IT1,PRKN,PRKN,PACRG,PACRG,PACRG,PACRG-AS3,PACRG,PACRG-AS1,QKI,C6orf118,PDE10A,LINC00473,LINC00602,LINC00602,T,PRR18,SFT2D1,MPC1,RPS6KA2,RPS6KA2,MIR1913,RNASET2,MIR3939,FGFR1OP,CCR6,GPR31,TCP10L2,UNC93A,TTLL2,TCP10,LINC02538,LINC01558,AFDN-DT,AFDN,HGC6.3,KIF25,FRMD1,DACT2,SMOC2,LOC101929523,THBS2,THBS2,WDR27,WDR27,C6orf120,C6orf120,C6orf120,PHF10,PHF10,TCTE3,TCTE3,ERMARD,ERMARD,LINC00242,LINC00574,DLL1,FAM120B,FAM120B,MIR4644,PSMB1,TBP,PDCD2 | -0.0985517 | 266.885 | 1950 | 1162.03 |

| | | | | | | | | |
|---|---|---|---|---|---|---|---|---|
| 1 | 5 | 82876768 | 139931828 | HAPLN1,EDIL3,NBPF22P,COX7C,COX7C,SNORD138,MIR4280,LOC101929380,LINC01949,RASA1,CCNH,TMEM161B,LINC00461,MIR9-2,LINC00461,MEF2C-AS2,MEF2C,MEF2C,MEF2C-AS1,MIR3660,CETN3,LOC731157,MBLAC2,POLR3G,LYSMD3,ADGRV1,ARRDC3,ARRDC3,ARRDC3-AS1,NR2F1,FAM172A,MIR2277,FAM172A,FAM172A,POU5F2,KIAA0825,SLF1,MCTP1,FAM81B,TTC37,ARSK,GPR150,RFESD,SPATA9,SPATA9,SPATA9,RHOBTB3,RHOBTB3,GLRX,LINC01554,ELL2,LOC101929710,MIR583,LOC101929710,PCSK1,LOC101929710,CAST,CAST,ERAP1,ERAP1,ERAP2,LNPEP,LIX1,RIOK2,RGMB,RGMB-AS1,RGMB,CHD1,FAM174A,ST8SIA4,ST8SIA4,MIR548P,SLCO4C1,SLCO6A1,PAM,GIN1,PPIP5K2,C5orf30,NUDT12,EFNA5,FBXL17,FER,PJA2,MAN2A1,LINC01848,TMEM232,TMEM232,MIR548F3,SLC25A46,TSLP,WDR36,CAMK4,STARD4,STARD4-AS1,STARD4-AS1,NREP,NREP,NREP,NREP-AS1,EPB41L4A,EPB41L4A,EPB41L4A-AS1,EPB41L4A,EPB41L4A-AS1,SNORA13,EPB41L4A,LOC101927023,EPB41L4A-AS2,APC,SRP19,SRP19,REEP5,REEP5,DCP2,MCC,MCC,TSSK1B,YTHDC2,KCNN2,KCNN2,LOC101927078,TRIM36,PGGT1B,CCDC112,FEM1C,TICAM2,TMED7-TICAM2,TMED7-TICAM2,LOC101927100,TMED7,TMED7-TICAM2,TMED7,CDO1,ATG12,ATG12,AP3S1,AP3S1,LVRN,ARL14EPL,COMMD10,SEMA6A,SEMA6A,SEMA6A-AS1,LINC02147,LINC02208,LINC02215,DTWD2,DMXL1,DMXL1,MIR5706,TNFAIP8,HSD17B4,FAM170A,PRR16,FTMT,SRFBP1,LOX,ZNF474,LOC100505841,SNCAIP,SNCAIP,MGC32805,LINC02201,SNX2,SNX24,PPIC,LOC105379152,PRDM6,PRDM6,CEP120,CSNK1G3,ZNF608,GR | -0.100429 | 273.322 | 3670 | 2186.65 |
| 1 | 1 | 1.16E+08 | 121484934 | TSHB,TSPAN2,NGF,VANGL1,CASQ2,NHLH2,LOC101928977,SLC22A15,SLC22A15,MAB21L3,ATP1A1,ATP1A1,ATP1A1-AS1,ATP1A1-AS1,CD58,IGSF3,MIR320B1,C1orf137,CD2,PTGFRN,CD101,CD101,LOC101929099,TTF2,TTF2,MIR942,TRIM45,VTCN1,MAN1A2,FAM46C,GDAP2,WDR3,WDR3,SPAG17,SPAG17,TBX15,WARS2,WARS2,LOC101929147,HAO2,HSD3B2,HSD3B1,ZNF697,PHGDH,HMGCS2,REG4,NBPF7,ADAM30,NOTCH2,FCGR1B,SRGAP2-AS1,SRGAP2D,SRGAP2B,SRGAP2C,EMBP1 | -0.101152 | 276.264 | 568 | 341.627 |
| 1 | 5 | 1.51E+08 | 158586117 | ATOX1,G3BP1,GLRA1,NMUR2,GRIA1,FAM114A2,MFAP3,GALNT10,GALNT10,MIR1294,GALNT10,SAP30L-AS1,SAP30L,HAND1,MIR3141,MIR1303,LARP1,FAXDC2,FAXDC2,MIR378H,CNOT8,GEMIN5,MRPL22,KIF4B,SGCD,PPP1R2P3,TIMD4,HAVCR1,HAVCR2,MED7,FAM71B,ITK,CYFIP2,CYFIP2,FNDC9,CYFIP2,LOC102724404,NIPAL4,ADAM19,SOX30,C5orf52,THG1L,LSM11,CLINT1,EBF1,RNF145 | -0.101427 | 249.533 | 503 | 301.558 |

| | | | | | | | | |
|---|---|---|---|---|---|---|---|---|
| 1 | 7 | 1.51E+08 | 159128163 | NOB1,WDR86,WDR86,WDR86-AS1,WDR86-AS1,CRYGN,CRYGN,MIR3907,RHEB,PRKAG2,PRKAG2,LOC644090,GALNTL5,GALNT11,KMT2C,XRCC2,ACTR3B,DPP6,PAXIP1-AS2,PAXIP1-AS2,PAXIP1,PAXIP1,HTR5A-AS1,HTR5A-AS1,HTR5A,HTR5A,INSIG1,BLACE,LOC100286906,EN2,CNPY1,LOC100506302,RBM33,SHH,LOC389602,C7orf13,RNF32,RNF32,LMBR1,LMBR1,NOM1,MNX1,MNX1,MNX1-AS2,UBE3C,DNAJB6,LOC101927914,PTPRN2,PTPRN2,MIR153-2,PTPRN2,MIR595,LINC01022,MIR5707,NCAPG2,ESYT2,WDR60,VIPR2 | -0.102034 | 251.315 | 711 | 417.387 |
| 1 | 2 | 27917984 | 32093017 | LINC01460,MRPL33,RBKS,RBKS,BABAM2,BRE-AS1,BABAM2,BABAM2,MIR4263,BABAM2,LOC100505716,FLJ31356,FOSL2,FOSL2,PLB1,PPP1CB,SPDYA,SPDYA,TRMT61B,TRMT61B,WDR43,WDR43,SNORD92,WDR43,SNORD53,TOGARAM2,C2orf71,CLIP4,ALK,YPEL5,LBH,LCLAT1,CAPN13,GALNT14,CAPN14,EHD3,XDH,SRD5A2 | -0.102645 | 262.015 | 441 | 268.998 |
| 1 | X | 1.12E+08 | 117680028 | HTR2C,SNORA35,HTR2C,MIR764,HTR2C,MIR1912,HTR2C,MIR1264,HTR2C,MIR1298,HTR2C,HTR2C,MIR1911,HTR2C,MIR448,IL13RA2,LRCH2,LRCH2,RBMXL3,LUZP4,PLS3,AGTR2,SLC6A14,CT83,KLHL13,WDR44,WDR44,MIR1277,DOCK11 | -0.105858 | 273.036 | 199 | 116.402 |
| 1 | 4 | 47939555 | 49632814 | LOC101927157,CNGA1,NIPAL1,TXK,TEC,SLAIN2,SLC10A4,ZAR1,FRYL,OCIAD1,OCIAD1,OCIAD1-AS1,OCIAD2,CWH43 | -0.106959 | 287.139 | 194 | 114.316 |
| 1 | 3 | 60500 | 5211565 | CHL1,CHL1,CHL1-AS1,CNTN6,CNTN4,CNTN4,CNTN4-AS1,IL5RA,TRNT1,CRBN,LRRN1,SETMAR,SUMF1,ITPR1,BHLHE40-AS1,BHLHE40-AS1,BHLHE40,BHLHE40,ARL8B | -0.107592 | 259.767 | 288 | 175.887 |
| 1 | 2 | 2.14E+08 | 218679761 | SPAG16,SPAG16,MIR4438,VWC2L,BARD1,LOC101928103,ABCA12,ABCA12,ATIC,FN1,MREG,PECR,TMEM169,XRCC5,MARCH4,SMARCAL1,RPL37A,IGFBP2,IGFBP5,TNP1,LINC01921,DIRC3,TNS1 | -0.107884 | 247.981 | 323 | 193.924 |

| | | | | | | | | |
|---|---|---|---|---|---|---|---|---|
| 1 | 8 | 47458041 | 77615793 | LINC00293,LOC100287846,SPIDR,CEBPD,PRKDC,MCM4,UBE2V2,EFCAB1,SNAI2,PPDPFL,SNTG1,PXDNL,PCMTD1,ST18,ALKAL1,RB1CC1,NPBWR1,OPRK1,ATP6V1H,RGS20,TCEA1,LYPLA1,MRPL15,SOX17,RP1,XKR4,TMEM68,TGS1,LYN,RPS20,RPS20,SNORD54,MOS,PLAG1,CHCHD7,SDR16C5,SDR16C6P,PENK,PENK,LOC101929415,IMPAD1,FAM110B,UBXN2B,CYP7A1,SDCBP,NSMAF,TOX,CA8,RAB2A,CHD7,LOC100130298,CLVS1,ASPH,MIR4470,NKAIN3,NKAIN3,UG0898H09,GGH,TTPA,YTHDF3,MIR124-2HG,MIR124-2HG,MIR124-2,LOC401463,BHLHE22,CYP7B1,LINC01299,ARMC1,MTFR1,PDE7A,DNAJC5B,TRIM55,CRH,LINC00967,RRS1,ADHFE1,C8orf46,MYBL1,VCPIP1,C8orf44,C8orf44-SGK3,C8orf44-SGK3,SGK3,PTTG3P,C8orf44-SGK3,SGK3,MCMDC2,SNHG6,SNORD87,TCF24,PPP1R42,COPS5,CSPP1,ARFGEF1,LOC102724708,CPA6,CPA6,PREX2,C8orf34-AS1,C8orf34-AS1,C8orf34,C8orf34,SULF1,SLCO5A1,PRDM14,NCOA2,TRAM1,LACTB2-AS1,LACTB2,LACTB2,XKR9,EYA1,MSC,MSC,MSC-AS1,MSC-AS1,MSC-AS1,TRPA1,TRPA1,LOC392232,KCNB2,TERF1,SBSPON,C8orf89,RPL7,RDH10,RDH10,RDH10-AS1,STAU2-AS1,STAU2-AS1,STAU2,STAU2,UBE2W,ELOC,TMEM70,LY96,JPH1,GDAP1,MIR5681A,MIR5681B,MIR2052HG,MIR2052HG,MIR2052,PI15,CRISPLD1,HNF4G | -0.109708 | 267.704 | 1797 | 1055.36 |
| 1 | 4 | 55958723 | 68337860 | KDR,SRD5A3,SRD5A3,SRD5A3-AS1,TMEM165,CLOCK,PDCL2,NMU,EXOC1L,EXOC1,CEP135,KIAA1211,AASDH,PPAT,PAICS,SRP72,ARL9,THEGL,HOPX,SPINK2,REST,NOA1,POLR2B,IGFBP7,IGFBP7,IGFBP7-AS1,MIR548AG1,ADGRL3,TECRL,EPHA5,MIR1269A | -0.114296 | 251.579 | 466 | 280.306 |
| 1 | 11 | 69514018 | 69589904 | FGF19,FGF4 | -0.115436 | 253.86 | 7 | 4.6683 |
| 1 | 2 | 1.09E+08 | 114475550 | GCC2,GCC2,GCC2-AS1,LIMS1,RANBP2,CCDC138,EDAR,SH3RF3-AS1,SH3RF3-AS1,SH3RF3,SH3RF3,MIR4265,SH3RF3,MIR4266,SH3RF3,SEPT10,SOWAHC,RGPD5,RGPD6,LIMS3,LIMS4,LIMS3-LOC440895,MIR4267,MALL,MALL,MIR4436B1,MIR4436B2,NPHP1,MIR4436B1,MIR4436B2,LIMS3-LOC440895,LIMS3,LIMS4,RGPD6,RGPD5,BUB1,ACOXL,BCL2L11,MIR4435-2HG,MIR4435-2HG,MIR4435-1,MIR4435-2,ANAPC1,ANAPC1,MIR4771-1,MIR4771-2,MERTK,TMEM87B,FBLN7,ZC3H8,ZC3H6,RGPD8,RGPD5,TTL,LOC105373562,POLR1B,POLR1B,CHCHD5,SLC20A1,NT5DC4,NT5DC4,CKAP2L,CKAP2L,IL1A,IL1B,IL37,IL36G,IL36A,IL36B,IL36RN,IL1F10,IL1RN,PSD4,PAX8,PAX8,PAX8-AS1,CBWD2,RABL2A,SLC35F5 | -0.117482 | 277.067 | 595 | 346.097 |
| 1 | 4 | 13322 | 517304 | ZNF595,ZNF718,ZNF718,ZNF732,ZNF141,ZNF141,MIR571,ABCA11P,ABCA11P,ZNF721,PIGG | -0.11978 | 324.607 | 83 | 44.8735 |

| | | | | | | | | |
|---|---|---|---|---|---|---|---|---|
| 1 | 3 | 50005951 | 50154746 | RBM6,RBM5,RBM5,RBM5-AS1,RBM5,SEMA3F-AS1 | -0.12223 | 259.387 | 51 | 30.4071 |
| 1 | 6 | 1.37E+08 | 149826905 | MAP7,MAP3K5,MAP3K5,LOC101928461,PEX7,SLC35D3,IL20RA,IL22RA2,IFNGR1,OLIG3,TNFAIP3,PERP,ARFGEF3,ARFGEF3,PBOV1,HEBP2,NHSL1,NHSL1,MIR3145,FLJ46906,GVQW2,GVQW2,CCDC28A,CCDC28A,ECT2L,REPS1,ABRACL,HECA,TXLNB,CITED2,MIR3668,MIR4465,NMBR,NMBR,GJE1,VTA1,ADGRG6,HIVEP2,AIG1,ADAT2,PEX3,FUCA2,PHACTR2,LTV1,ZC2HC1B,PLAGL1,SF3B5,STX11,UTRN,EPM2A,EPM2A,LOC100507557,LOC100507557,LOC100507557,FBXO30,LOC100507557,SHPRH,SHPRH,GRM1,RAB32,ADGB,ADGB,KATNBL1P6,STXBP5-AS1,STXBP5,STXBP5,SAMD5,SASH1,UST,UST,UST-AS1,TAB2,TAB2,SUMO4,ZC3H12D,PPIL4 | -0.123627 | 264.87 | 938 | 564.557 |
| 1 | 9 | 38527768 | 47317179 | ANKRD18A,FAM201A,CNTNAP3,ZNF658B,ZNF658,CNTNAP3B,CNTNAP3P2 | -0.12439 | 185.559 | 100 | 51.7091 |
| 1 | 15 | 44128343 | 44614547 | WDR76,FRMD5,CASC4 | -0.125556 | 249.656 | 36 | 22.3861 |
| 1 | 2 | 1.71E+08 | 171627719 | UBR3,MYO3B,LOC101926913,LINC01124,LOC101926913,SP5,LOC101926913,ERICH2,ERICH2 | -0.127549 | 265.338 | 96 | 56.6379 |
| 1 | 14 | 24912592 | 26918192 | LOC101927045,CMA1,CTSG,GZMH,GZMB,STXBP6,NOVA1 | -0.128083 | 209.115 | 58 | 33.5764 |
| 1 | 2 | 2.04E+08 | 214149545 | CYP20A1,ABI2,RAPH1,CD28,CTLA4,ICOS,PARD3B,NRP2,INO80D,NDUFS1,EEF1B2,EEF1B2,SNORD51,EEF1B2,SNORA41,GPR1,ZDBF2,ADAM23,FAM237A,DYTN,MDH1B,FASTKD2,FASTKD2,MIR3130-1,MIR3130-2,CPO,KLF7,KLF7,MIR2355,LINC01802,MIR1302-4,CREB1,CREB1,METTL21A,METTL21A,CCNYL1,CCNYL1,MIR4775,FZD5,PLEKHM3,LOC100507443,CRYGD,LOC100507443,CRYGC,LOC100507443,CRYGB,CRYGA,C2orf80,IDH1,PIKFYVE,PTH2R,LOC101927960,MAP2,UNC80,RPE,KANSL1L,KANSL1L,LOC101928020,ACADL,MYL1,LANCL1-AS1,LANCL1,LANCL1,CPS1,ERBB4,ERBB4,MIR548F2,MIR4776-1,MIR4776-2,IKZF2,LOC100130451,SPAG16 | -0.131965 | 258.531 | 836 | 502.143 |

| | | | | | | | | |
|---|---|---|---|---|---|---|---|---|
| 1 | 7 | 1.29E+08 | 141919901 | AHCYL2,STRIP2,SMKR1,NRF1,MIR182,MIR96,MIR183,UBE2H,ZC3HC1,KLHDC10,TMEM209,SSMEM1,CPA2,CPA4,CPA5,CPA1,CEP41,MEST,MEST,MIR335,COPG2,COPG2,TSGA13,TSGA13,KLF14,MIR29A,MIR29B1,LINC-PINT,MKLN1,PODXL,PLXNA4,FLJ40288,CHCHD3,EXOC4,EXOC4,LOC101928861,LRGUK,SLC35B4,AKR1B1,AKR1B10,AKR1B15,BPGM,CALD1,AGBL3,AGBL3,C7orf49,C7orf49,C7orf49,TMEM140,WDR91,WDR91,MIR6509,STRA8,CNOT4,NUP205,STMP1,SLC13A4,FAM180A,LUZP6,MTPN,CHRM2,LOC349160,MIR490,CHRM2,LOC349160,PTN,DGKI,CREB3L2,CREB3L2,LOC100130880,AKR1D1,MIR4468,TRIM24,SVOPL,ATP6V0A4,ATP6V0A4,TMEM213,TMEM213,KIAA1549,ZC3HAV1L,ZC3HAV1,TTC26,UBN2,LUC7L2,FMC1,C7orf55-LUC7L2,LUC7L2,C7orf55-LUC7L2,LUC7L2,C7orf55-LUC7L2,LOC100129148,KLRG2,CLEC2L,HIPK2,TBXAS1,PARP12,KDM7A,SLC37A3,RAB19,MKRN1,DENND2A,ADCK2,NDUFB2-AS1,NDUFB2-AS1,NDUFB2,NDUFB2,BRAF,MRPS33,TMEM178B,AGK,KIAA1147,WEE2-AS1,WEE2,SSBP1,TAS2R3,TAS2R4,TAS2R5,PRSS37,OR9A4,CLEC5A,TAS2R38,MGAM,MGAM2 | -0.133078 | 261.634 | 1491 | 876.01 |
| 1 | 17 | 25268559 | 28088280 | [illegible],NLK,PPY2P,KRT18P55,TMEM97,TMEM97,IFT20,IFT20,TNFAIP1,POLDIP2,TMEM199,TMEM199,MIR4723,SEBOX,VTN,SARM1,SARM1,SLC46A1,SLC46A1,SLC13A2,FOXN1,UNC119,PIGS,ALDOC,SPAG5,SPAG5-AS1,SGK494,SPAG5-AS1,KIAA0100,KIAA0100,SDF2,SUPT6H,PROCA1,RAB34,RPL23A,RPL23A,SNORD42B,RPL23A,SNORD4A,RPL23A,SNORD42A,RPL23A,SNORD4B,TLCD1,NEK8,TRAF4,FAM222B,ERAL1,MIR451A,MIR451B,MIR144,MIR4732,FLOT2,DHRS13,PHF12,PHF12,LOC101927018,SEZ6,PIPOX,MYO18A,TIAF1,MYO18A,CRYBA1,NUFIP2,MIR4523,TAOK1,ABHD15,TP53I13,GIT1,ANKRD13B,CORO6,SSH2 | -0.133872 | 259.743 | 706 | 444.501 |

| | | | | | | | | |
|---|---|---|---|---|---|---|---|---|
| 1 | 15 | 62352431 | 73924960 | VPS13C,C2CD4A,C2CD4B,GOLGA2P11,MGC15885,TLN2,TLN2,MIR190A,TPM1,TPM1,TPM1-AS,LACTB,RPS27L,RAB8B,APH1B,CA12,USP3,USP3,USP3-AS1,USP3-AS1,FBXL22,FBXL22,HERC1,MIR422A,DAPK2,DAPK2,LOC101928988,FAM96A,SNX1,SNX22,SNX22,PPIB,PPIB,CSNK1G1,PCLAF,TRIP4,ZNF609,OAZ2,RBPMS2,RBPMS2,MIR1272,PIF1,PLEKHO2,ANKDD1A,SPG21,MTFMT,SLC51B,RASL12,KBTBD13,UBAP1L,PDCD7,CLPX,CILP,PARP16,IGDCC3,IGDCC4,DPP8,HACD3,INTS14,SLC24A1,SLC24A1,DENND4A,DENND4A,RAB11A,MEGF11,MEGF11,MIR4311,DIS3L,TIPIN,TIPIN,SCARNA14,MAP2K1,MAP2K1,SNAPC5,SNAPC5,RPL4,RPL4,SNORD18C,RPL4,SNORD18B,RPL4,SNORD16,RPL4,SNORD18A,ZWILCH,ZWILCH,LCTL,LCTL,LINC01169,SMAD6,LINC02206,LOC102723493,SMAD3,AAGAB,AAGAB,IQCH,IQCH,IQCH,IQCH-AS1,IQCH-AS1,C15orf61,C15orf61,MAP2K5,SKOR1,PIAS1,CALML4,CLN6,FEM1B,ITGA11,CORO2B,ANP32A,ANP32A,MIR4312,MIR548H4,SPESP1,NOX5,MIR548H4,NOX5,GLCE,PAQR5,PAQR5,LOC145694,LOC145694,KIF23,KIF23,RPLP1,DRAIC,TLE3,TLE3,MIR629,UACA,LARP6,LARP6,LRRC49,LRRC49,LRRC49,THAP10,CT62,THSD4,NR2E3,MYO9A,SENP8,GRAMD2A,PKM,PARP6,CELF6,HEXA,HEXA-AS1,TMEM202,TMEM202,TMEM202-AS1,TMEM202-AS1,ARIH1,MIR630,HIGD2B,BBS4,ADPGK,ADPGK,ADPGK-AS1,ADPGK-AS1,NEO1,HCN4,REC114,NPTN | -0.137342 | 267.614 | 1697 | 1015.08 |
| 1 | 18 | 40850364 | 48421761 | SYT4,SETBP1,SETBP1,MIR4319,SLC14A2,SLC14A1,SIGLEC15,EPG5,PSTPIP2,ATP5F1A,HAUS1,C18orf25,RNF165,LOXHD1,ST8SIA5,PIAS2,PIAS2,KATNAL2,KATNAL2,KATNAL2,ELOA2,HDHD2,IER3IP1,SKOR2,MIR4527HG,MIR4527,SMAD2,ZBTB7C,CTIF,CTIF,MIR4743,SMAD7,LOC100129878,DYM,DYM,MIR4744,DYM,C18orf32,RPL17-C18orf32,RPL17-C18orf32,MIR1539,RPL17-C18orf32,RPL17,RPL17-C18orf32,RPL17,SNORD58C,RPL17-C18orf32,RPL17,SNORD58A,RPL17-C18orf32,RPL17,SNORD58B,LIPG,ACAA2,SCARNA17,SNHG22,SNHG22,MYO5B,MYO5B,MYO5B,MIR4320,CFAP53,MBD1,CXXC1,SKA1,MAPK4,MRO | -0.142256 | 260.131 | 634 | 376.174 |
| 1 | 17 | 17067950 | 19436737 | MPRIP,PLD6,FLCN,COPS3,NT5M,MED9,RASD1,PEMT,RAI1,SMCR5,RAI1,RAI1,SREBF1,SREBF1,SREBF1,MIR6777,SREBF1,MIR33B,TOM1L2,DRC3,ATPAF2,GID4,DRG2,MYO15A,ALKBH5,LLGL1,FLII,MIEF2,TOP3A,SMCR8,SHMT1,EVPLL,LINC02076,LGALS9C,CCDC144B,TBC1D28,ZNF286B,ZNF286B,FOXO3B,TRIM16L,FBXW10,TVP23B,PRPSAP2,SLC5A10,SLC5A10,FAM83G,GRAP,LOC388436,LOC79999,GRAPL,GRAPL,LOC388436,LOC79999,EPN2,EPN2,B9D1,B9D1,B9D1,MIR1180,MAPK7,MFAP4,RNF112 | -0.144618 | 274.256 | 649 | 396.352 |

| | | | | | | | | |
|---|---|---|---|---|---|---|---|---|
| 1 | 18 | 31224776 | 35690905 | ASXL3,NOL4,DTNA,MAPRE2,ZNF397,ZNF397,ZSCAN30,ZSCAN30,ZNF271P,ZNF24,ZNF396,INO80C,MIR3975,GALNT1,MIR187,MIR3929,C18orf21,RPRD1A,SLC39A6,ELP2,MOCOS,FHOD3,TPGS2,TPGS2,KIAA1328,KIAA1328,CELF4,CELF4,LOC105372068,MIR4318 | -0.14523 | 259.31 | 362 | 216.203 |
| 1 | 2 | 10500 | 11312163 | FAM110C,SH3YL1,ACP1,ALKAL2,TMEM18,LOC101060391,SNTG2,SNTG2,TPO,PXDN,MYT1L,EIPR1,TRAPPC12,ADI1,RNASEH1,RPS7,COLEC11,ALLC,DCDC2C,SOX11,CMPK2,RSAD2,RNF144A,RNF144A,LOC101929452,LINC00299,ID2,KIDINS220,MBOAT2,ASAP2,ITGB1BP1,ITGB1BP1,CPSF3,CPSF3,IAH1,ADAM17,YWHAQ,TAF1B,GRHL1,KLF11,CYS1,RRM2,C2orf48,C2orf48,MIR4261,HPCAL1,ODC1,ODC1,SNORA80B,LOC101929715,NOL10,ATP6V1C2,ATP6V1C2,PDIA6,PDIA6,KCNF1,FLJ33534,C2orf50,PQLC3 | -0.146643 | 255.27 | 731 | 433.6 |
| 1 | 2 | 98201691 | 109085550 | ANKRD36B,COX5B,ACTR1B,ZAP70,TMEM131,VWA3B,CNGA3,INPP4A,COA5,UNC50,MGAT4A,KIAA1211L,TSGA10,TSGA10,C2orf15,TSGA10,LIPT1,LIPT1,MITD1,MRPL30,LYG2,LYG1,TXNDC9,EIF5B,REV1,AFF3,LONRF2,CHST10,NMS,PDCL3,NPAS2,NPAS2,LOC101927142,RPL31,RPL31,TBC1D8,TBC1D8,CNOT11,SNORD89,RNF149,MIR5696,CREG2,RFX8,MAP4K4,IL1R2,IL1R1,IL1RL2,IL1RL1,IL18R1,IL18RAP,IL18RAP,MIR4772,SLC9A4,SLC9A2,MFSD9,TMEM182,LOC100287010,POU3F3,LINC01159,LOC102724691,MRPS9,MRPS9,MRPS9,UTAT33,GPR45,TGFBRAP1,C2orf49,FHL2,NCK2,C2orf40,UXS1,PLGLA,CD8B2,ST6GAL2,SLC5A7,SULT1C3,SULT1C2,SULT1C4,GCC2 | -0.14674 | 264.547 | 924 | 554.224 |
| 1 | X | 75651699 | 99550745 | MIR325HG,MIR384,MIR325HG,MIR325,FGF16,ATRX,MAGT1,COX7B,ATP7A,ATP7A,PGAM4,PGK1,TAF9B,CYSLTR1,RTL3,LPAR4,MIR4328,P2RY10,GPR174,ITM2A,TBX22,FAM46D,BRWD3,HMGN5,SH3BGRL,POU3F4,CYLC1,RPS6KA6,MIR548I4,HDX,UBE2DNL,APOOL,APOOL,SATL1,SATL1,ZNF711,POF1B,MIR1321,CHM,CHM,MIR361,DACH2,KLHL4,CPXCR1,TGIF2LX,PABPC5,PCDH11X,NAP1L3,FAM133A,MIR548M,DIAPH2,DIAPH2,RPA4 | -0.146818 | 250.223 | 676 | 393.448 |
| 1 | 22 | 46805644 | 51244066 | CELSR1,GRAMD4,CERK,TBC1D22A,TBC1D22A,TBC1D22A-AS1,LINC01644,LINC00898,MIR3201,FAM19A5,FAM19A5,LOC284933,MIR4535,C22orf34,C22orf34,MIR3667,BRD1,ZBED4,ALG12,CRELD2,PIM3,PIM3,MIR6821,IL17REL,TTLL8,MLC1,MOV10L1,PANX2,TRABD,SELENOO,SELENOO,TUBGCP6,TUBGCP6,HDAC10,MAPK12,MAPK11,PLXNB2,DENND6B,PPP6R2,SBF1,ADM2,MIOX,LMF2,NCAPH2,NCAPH2,SCO2,SCO2,TYMP,TYMP,ODF3B,KLHDC7B,SYCE3,CPT1B,CHKB-CPT1B,CHKB-CPT1B,CHKB,MAPK8IP2,ARSA,SHANK3,ACR,RPL23AP82,RABL2B | -0.150997 | 258.559 | 688 | 430.932 |
| 1 | X | 1E+08 | 100617743 | TRMT2B,TMEM35A,CENPI,DRP2,TAF7L,TIMM8A,BTK | -0.153015 | 276.543 | 96 | 58.089 |

| | | | | | | | | |
|---|---|---|---|---|---|---|---|---|
| 1 | 12 | 76468534 | 85285886 | BBS10,OSBPL8,ZDHHC17,CSRP2,E2F7,NAV3,SYT1,SYT1,MIR1252,PAWR,PPP1R12A,OTOGL,PTPRQ,MYF6,MYF5,LIN7A,LIN7A,MIR617,LIN7A,MIR618,ACSS3,ACSS3,MIR4699,PPFIA2,PPFIA2,PPFIA2-AS1,CCDC59,CCDC59,METTL25,METTL25,TMTC2,SLC6A15 | -0.15302 | 267.479 | 546 | 323.577 |
| 1 | 1 | 1.09E+08 | 109780557 | AKNAD1,GPSM2,GPSM2,CLCC1,CLCC1,WDR47,TAF13,TMEM167B,SCARNA2,C1orf194,KIAA1324,SARS | -0.153529 | 260.821 | 122 | 76.0341 |
| 1 | 2 | 1.34E+08 | 138208587 | NCKAP5,NCKAP5,LOC101928185,MIR3679,MGAT5,TMEM163,ACMSD,ACMSD,CCNT2-AS1,CCNT2,MAP3K19,RAB3GAP1,ZRANB3,R3HDM1,R3HDM1,MIR128-1,UBXN4,LCT,LCT,LOC100507600,MCM6,DARS,DARS,DARS-AS1,CXCR4,THSD7B | -0.154172 | 257.879 | 335 | 204.239 |
| 1 | 5 | 1.41E+08 | 147793906 | RNF14,GNPDA1,NDFIP1,SPRY4,FGF1,ARHGAP26,NR3C1,MIR5197,HMHB1,YIPF5,KCTD16,PRELID2,GRXCR2,SH3RF2,PLAC8L1,LARS,RBM27,POU4F3,TCERG1,GPR151,PPP2R2B,STK32A,DPYSL3,JAKMIP2-AS1,JAKMIP2-AS1,JAKMIP2,JAKMIP2,SPINK1,SCGB3A2,C5orf46,SPINK5,SPINK14,SPINK6,LOC102546294,SPINK13,LOC102546294,SPINK7,LOC102546294,LOC102546294,SPINK9,FBXO38 | -0.159463 | 253.791 | 457 | 272.434 |
| 1 | 21 | 27141424 | 30444433 | GABPA,APP,CYYR1-AS1,CYYR1,CYYR1,ADAMTS1,ADAMTS5,ADAMTS5,MIR4759,N6AMT1,LTN1,RWDD2B,USP16,CCT8 | -0.161044 | 269.872 | 179 | 106.285 |
| 1 | 15 | 44816844 | 49304062 | EIF3J-AS1,EIF3J,SPG11,PATL2,B2M,TRIM69,SORD2P,TERB2,SORD,DUOX2,DUOXA2,DUOXA2,DUOXA1,DUOXA1,DUOX1,SHF,LOC101928414,SLC28A2,GATM,SPATA5L1,C15orf48,C15orf48,MIR147B,SLC30A4,SLC30A4,HMGN2P46,HMGN2P46,BLOC1S6,SQOR,SEMA6D,SLC24A5,SLC24A5,MYEF2,MYEF2,CTXN2,SLC12A1,DUT,FBN1,CEP152,SHC4,SHC4,EID1,SECISBP2L | -0.162986 | 280.446 | 563 | 340.616 |
| 1 | 12 | 21721767 | 34431528 | GYS2,LDHB,KCNJ8,ABCC9,CMAS,ST8SIA1,C2CD5,ETNK1,SOX5,SOX5,MIR920,LINC00477,BCAT1,C12orf77,LRMP,CASC1,ETFRF1,KRAS,LMNTD1,MIR4302,RASSF8,BHLHE41,SSPN,ITPR2,INTS13,FGFR1OP2,TM7SF3,MED21,C12orf71,STK38L,ARNTL2,ARNTL2,ARNTL2-AS1,SMCO2,PPFIBP1,REP15,MRPS35,MANSC4,KLHL42,PTHLH,CCDC91,FAR2,FAR2,LOC100506606,ERGIC2,OVCH1-AS1,OVCH1-AS1,OVCH1,OVCH1,TMTC1,IPO8,CAPRIN2,LINC00941,TSPAN11,DDX11,SINHCAF,SINHCAF,FLJ13224,DENND5B,DENND5B,DENND5B-AS1,ETFBKMT,AMN1,H3F3C,KIAA1551,BICD1,FGD4,DNM1L,YARS2,PKP2,SYT10,ALG10 | -0.168151 | 262.138 | 1058 | 617.581 |

| | | | | | | | | |
|---|---|---|---|---|---|---|---|---|
| 1 | 7 | 94257456 | 99473898 | SGCE,PEG10,PPP1R9A,PON1,PON3,PON2,ASB4,PDK4,DYNC1I1,SLC25A13,SLC25A13,MIR591,SEM1,DLX6-AS1,DLX6,DLX5,SDHAF3,TAC1,ASNS,CZ1P-ASNS,CZ1P-ASNS,MIR5692C2,CZ1P-ASNS,CCZ1P-OR7E38P,OCM2,LMTK2,BHLHA15,TECPR1,BRI3,BRI3,BAIAP2L1,BAIAP2L1,NPTX2,TMEM130,TRRAP,TRRAP,MIR3609,SCARNA28,TRRAP,SCARNA28,LOC101927550,SMURF1,SMURF1,KPNA7,MYH16,ARPC1A,ARPC1B,PDAP1,BUD31,BUD31,PTCD1,ATP5MF-PTCD1,PTCD1,ATP5MF-PTCD1,ATP5MF-PTCD1,CPSF4,ATP5MF-PTCD1,ATP5MF,ZNF789,ZNF789,ZNF394,ZNF394,ZKSCAN5,FAM200A,ZNF655,TMEM225B,ZSCAN25,ZSCAN25,CYP3A5,CYP3A5,CYP3A7-CYP3A51P,CYP3A7-CYP3A51P,CYP3A7,CYP3A4,CYP3A43,OR2AE1 | -0.168257 | 284.485 | 661 | 396.65 |
| 1 | 5 | 37049223 | 43479412 | NIPBL,C5orf42,NUP155,WDR70,GDNF,EGFLAM,LIFR,LIFR,LIFR-AS1,MIR3650,OSMR-AS1,OSMR,RICTOR,FYB1,C9,DAB2,PTGER4,TTC33,PRKAA1,RPL37,SNORD72,RPL37,CARD6,C7,MROH2B,C6,PLCXD3,OXCT1,OXCT1,OXCT1-AS1,C5orf51,FBXO4,GHR,CCDC152,CCDC152,SELENOP,SELENOP,LOC648987,ANXA2R,LOC648987,LOC100132356,ZNF131,NIM1K,HMGCS1,CCL28,TMEM267 | -0.176167 | 268.629 | 622 | 371.44 |
| 1 | 2 | 2.21E+08 | 227659142 | EPHA4,PAX3,PAX3,CCDC140,CCDC140,SGPP2,FARSB,MOGAT1,ACSL3,KCNE4,SCG2,AP1S3,WDFY1,MRPL44,SERPINE2,FAM124B,CUL3,DOCK10,DOCK10,MIR4439,NYAP2,MIR5702 | -0.177469 | 239.978 | 305 | 181.927 |
| 1 | 2 | 37450339 | 39222525 | CEBPZ,CEBPZ,NDUFAF7,NDUFAF7,NDUFAF7,PRKD3,PRKD3,QPCT,CDC42EP3,RMDN2,RMDN2,RMDN2-AS1,CYP1B1,ATL2,LOC101929596,HNRNPLL,GALM,SRSF7,GEMIN6,DHX57,MORN2,ARHGEF33,ARHGEF33,LOC375196,SOS1 | -0.184329 | 272.041 | 220 | 134.607 |
| 1 | 2 | 1.72E+08 | 171853213 | ERICH2,GAD1,GORASP2,TLK1 | -0.185216 | 275.298 | 46 | 26.7563 |
| 1 | 15 | 34456478 | 40322726 | EMC4,SLC12A6,NOP10,NUTM1,LPCAT4,GOLGA8A,GOLGA8A,MIR1233-2,MIR1233-1,GOLGA8B,GOLGA8B,MIR1233-2,MIR1233-1,GJD2,LOC101928174,ACTC1,AQR,ZNF770,DPH6,MIR3942,DPH6,MIR4510,C15orf41,C15orf41,CSNK1A1P1,CSNK1A1P1,LOC145845,MEIS2,TMCO5A,SPRED1,FAM98B,RASGRP1,C15orf53,C15orf54,THBS1,FSIP1,GPR176,EIF2AK4 | -0.189563 | 248.86 | 424 | 248.35 |
| 1 | 2 | 32429661 | 37310556 | SLC30A6,NLRC4,YIPF4,BIRC6,BIRC6,MIR558,TTC27,TTC27,MIR4765,LTBP1,MIR4430,RASGRP3,RASGRP3,LOC105374454,FAM98A,LINC01317,MYADML,CRIM1,FEZ2,VIT,STRN,HEATR5B | -0.189899 | 271.082 | 398 | 237.296 |
| 1 | 18 | 2796391 | 9104348 | SMCHD1,EMILIN2,LPIN2,LPIN2,LOC727896,MYOM1,MYL12A,MYL12A,LOC104968399,MYL12B,TGIF1,DLGAP1,DLGAP1,DLGAP1-AS1,DLGAP1,DLGAP1-AS2,DLGAP1,DLGAP1-AS3,AKAIN1,LINC00667,ZBTB14,EPB41L3,MIR3976,TMEM200C,L3MBTL4,L3MBTL4,MIR4317,LINC01387,LOC101927168,ARHGAP28,LINC00668,LAMA1,LAMA1,LOC101927188,LRRC30,PTPRM,RAB12,GACAT2,MTCL1,NDUFV2 | -0.192013 | 257.159 | 472 | 278.936 |

| | | | | | | | | |
|---|---|---|---|---|---|---|---|---|
| 1 | 7 | 47897151 | 57938684 | PKD1L1,HUS1,SUN3,C7orf57,UPP1,ABCA13,CDC14C,VWC2,ZPBP,SPATA48,IKZF1,FIGNL1,DDC,DDC,DDC-AS1,GRB10,COBL,POM121L12,VSTM2A,VSTM2A,VSTM2A-OT1,SEC61G,EGFR,EGFR,EGFR-AS1,LANCL2,VOPP1,FKBP9P1,SEPT14,ZNF713,MRPS17,NIPSNAP2,PSPH,CCT6A,CCT6A,SNORA15,SUMF2,PHKG1,CHCHD2,NUPR2,LOC401357,MIR4283-1,MIR4283-2,ZNF479,GUSBP10,MIR3147,ZNF716 | -0.192145 | 245.041 | 601 | 349.376 |
| 1 | 7 | 35058793 | 39247229 | DPY19L1,DPY19L2P1,TBX20,LOC401324,HERPUD2,SEPT7,EEPD1,KIAA0895,KIAA0895,ANLN,ANLN,AOAH,ELMO1,ELMO1,MIR1200,ELMO1,ELMO1-AS1,GPR141,NME8,SFRP4,EPDR1,STARD3NL,TARP,TRG-AS1,AMPH,FAM183BP,VPS41,POU6F2,POU6F2-AS2,POU6F2 | -0.196379 | 252.586 | 371 | 212.617 |
| 1 | 9 | 1.14E+08 | 115215741 | KIAA0368,ZNF483,ZNF483,PTGR1,PTGR1,LRRC37A5P,DNAJC25,DNAJC25-GNG10,DNAJC25-GNG10,GNG10,C9orf84,UGCG,UGCG,MIR4668,SUSD1,PTBP3,HSDL2 | -0.205374 | 246.105 | 194 | 113.753 |
| 1 | 21 | 9411693 | 19713843 | MIR3648-1,MIR3648-2,TEKT4P2,TPTE,BAGE2,BAGE4,BAGE3,BAGE5,BAGE2,BAGE4,BAGE3,BAGE5,BAGE,ANKRD20A11P,LIPI,RBM11,ABCC13,HSPA13,SAMSN1,SAMSN1,SAMSN1-AS1,SAMSN1-AS1,SAMSN1-AS1,LOC388813,LOC388813,NRIP1,USP25,MIR99AHG,MIR99AHG,MIR99A,MIR99AHG,MIRLET7C,MIR99AHG,MIR125B2,LINC01549,CXADR,BTG3,C21orf91,CHODL,TMPRSS15 | -0.206251 | 300.197 | 289 | 152.221 |
| 1 | 17 | 37563727 | 38547940 | MED1,CDK12,NEUROD2,PPP1R1B,STARD3,TCAP,PNMT,PGAP3,ERBB2,ERBB2,MIR4728,MIEN1,GRB7,IKZF3,ZPBP2,GSDMB,ORMDL3,LRRC3C,GSDMA,PSMD3,CSF3,MED24,MED24,MIR6884,MED24,SNORD124,THRA,THRA,NR1D1,NR1D1,MSL1,CASC3,CASC3,MIR6866,RAPGEFL1,RAPGEFL1,MIR6867,WIPF2,CDC6,RARA,RARA,RARA-AS1,GJD3,TOP2A | -0.207462 | 258.645 | 365 | 227.414 |

| | | | | | | | | |
|---|---|---|---|---|---|---|---|---|
| 1 | 17 | 38976439 | 44249208 | KRT10,TMEM99,TMEM99,KRT12,KRT20,KRT23,KRT39,KRT40,KRTAP3-3,KRTAP3-2,KRTAP3-1,KRTAP1-5,KRTAP1-4,KRTAP1-3,KRTAP1-1,KRTAP2-3,KRTAP2-4,KRTAP4-7,KRTAP4-8,KRTAP4-8,KRTAP4-9,KRTAP4-8,KRTAP4-11,KRTAP4-6,KRTAP4-5,KRTAP4-4,KRTAP4-3,KRTAP4-2,KRTAP4-1,KRTAP9-1,KRTAP9-3,KRTAP9-9,KRTAP9-9,KRTAP9-4,KRTAP9-9,KRTAP9-7,KRTAP29-1,KRTAP16-1,KRTAP17-1,KRT33A,KRT33B,KRT34,KRT31,KRT37,KRT38,KRT32,KRT35,KRT36,KRT13,KRT15,KRT19,KRT9,KRT14,KRT16,KRT17,EIF1,GAST,HAP1,JUP,P3H4,FKBP10,NT5C3B,NT5C3B,KLHL10,KLHL10,KLHL11,ACLY,TTC25,CNP,CNP,DNAJC7,DNAJC7,NKIRAS2,ZNF385C,DHX58,KAT2A,HSPB9,RAB5C,KCNH4,HCRT,GHDC,STAT5B,STAT5A,STAT3,CAVIN1,ATP6V0A1,ATP6V0A1,MIR548AT,ATP6V0A1,MIR5010,NAGLU,LOC108783654,HSD17B1,COASY,MLX,MLX,PSMC3IP,PSMC3IP,RETREG3,TUBG1,TUBG2,PLEKHH3,CCR10,CNTNAP1,EZH1,EZH1,MIR6780A,RAMP2-AS1,RAMP2-AS1,RAMP2,RAMP2,VPS25,WNK4,COA3,CNTD1,CNTD1,BECN1,BECN1,PSME3,AOC2,AOC3,AOC4P,LINC00671,G6PC,AARSD1,PTGES3L-AARSD1,PTGES3L-AARSD1,PTGES3L,RUNDC1,RPL27,IFI35,VAT1,RND2,BRCA1,NBR2,NBR1,TMEM106A,LINC00910,ARL4D,MIR2117HG,MIR2117,DHX8,DHX8,ETV4,ETV4,MEOX1,SOST,DUSP3,C17orf105,MPP3,CD300LG,MPP2,FAM215A,LINC01976,PPY,PYY,NAGS,TMEM101,LSM12,G6PC3,HDAC5,HDAC5,LOC105371789,C17orf53,ASB16,ASB16,ASB16-AS1,TMUB2,ATXN7L3,UBTF,UBTF,MIR6782,SLC4A1,RUNDC3A,SLC25A39,GRN,FAM171A2,ITGA2B,GPATCH8,FZD2,MEIOC,CCDC43,DBF4B,ADAM11,GJC1,HIGD1B,HIGD1B,EFTUD2,EFTUD2,CCDC103,FAM187A,GFAP,KI | -0.207521 | 257.715 | 1764 | 1082.25 |

| | | | | | | | | |
|---|---|---|---|---|---|---|---|---|
| 1 | X | 60500 | 46618215 | PLCXD1,GTPBP6,PPP2R3B,SHOX,CRLF2,CSF2RA,IL3RA,SLC25A6,ASMTL-AS1,ASMTL,ASMTL,P2RY8,AKAP17A,AKAP17A,ASMT,ASMT,DHRSX,DHRSX,ZBED1,CD99,XG,XG,XGY2,GYG2,ARSD,ARSD-AS1,ARSD,ARSE,ARSH,ARSF,LINC01546,MXRA5,PRKX,LOC389906,FAM239B,NLGN4X,MIR4770,PUDP,PUDP,STS,MIR4767,PUDP,STS,STS,PNPLA4,MIR651,VCX2,ANOS1,FAM9A,FAM9B,TBL1X,GPR143,SHROOM2,CLDN34,WWC3,CLCN4,MID1,HCCS,ARHGAP6,ARHGAP6,AMELX,ARHGAP6,MIR548AX,MSL3,FRMPD4,PRPS2,TLR7,TLR8-AS1,TLR8,TMSB4X,FAM9C,GS1-600G8.3,ATXN3L,EGFL6,TCEANC,RAB9A,TRAPPC2,OFD1,GPM6B,GEMIN8,UBE2E4P,GLRA2,FANCB,MOSPD2,ASB9,ASB11,PIGA,VEGFD,PIR-FIGF,PIR-FIGF,PIR,BMX,ACE2,TMEM27,CA5BP1,CA5B,ZRSR2,AP1S2,GRPR,MAGEB17,CTPS2,CTPS2,MIR548AM,CTPS2,S100G,SYAP1,TXLNG,RBBP7,REPS2,NHS,NHS,MIR4768,SCML1,RAI2,BEND2,SCML2,CDKL5,CDKL5,RS1,RS1,PPEF1,PHKA2-AS1,PHKA2,PHKA2,ADGRG2,PDHA1,PDHA1,MAP3K15,MAP3K15,SH3KBP1,BCLAF3,MAP7D2,MAP7D2,MIR23C,EIF1AX,RPS6KA3,CNKSR2,KLHL34,SMPX,MBTPS2,MBTPS2,YY2,SMS,PHEX,PHEX,PHEX-AS1,PHEX,PTCHD1-AS,PTCHD1-AS,ZNF645,PTCHD1-AS,DDX53,PTCHD1,PRDX4,ACOT9,SAT1,APOO,CXorf58,KLHL15,EIF2S3,ZFX,SUPT20HL2,SUPT20HL1,PDK3,PCYT1B,POLA1,POLA1,SCARNA23,ARX,MAGEB18,MAGEB6,MAGEB5,VENTXP1,PPP4R3C,DCAF8L2,MAGEB10,DCAF8L1,IL1RAPL1,IL1RAPL1,MIR4666B,MAGEB2,MAGEB3,MAGEB4,MAGEB1,NR0B1,CXorf21,GK,TAB3,FTHL17,DMD,DMD,MIR3915,DMD,MIR548F5,FAM47A,TMEM47,FAM47B,MAGEB16,CFAP47,CFAP47,LOC101928627, | -0.208503 | 251.183 | 2949 | 1699.72 |
| 1 | 2 | 39606761 | 55536143 | MAP4K3,TMEM178A,THUMPD2,SLC8A1-AS1,SLC8A1,SLC8A1,C2orf91,PKDCC,LOC102723824,EML4,EML4,COX7A2L,KCNG3,MTA3,OXER1,HAAO,ZFP36L2,LINC01126,THADA,PLEKHH2,PLEKHH2,C1GALT1C1L,DYNC2LI1,DYNC2LI1,ABCG5,ABCG5,ABCG8,LRPPRC,PPM1B,SLC3A1,SLC3A1,PREPL,PREPL,CAMKMT,SIX3,SIX2,SRBD1,PRKCE,EPAS1,TMEM247,ATP6V1E2,RHOQ,RHOQ,LOC100506142,PIGF,CRIPT,SOCS5,LINC01118,LINC01119,MCFD2,MCFD2,TTC7A,TTC7A,STPG4,CALM2,LOC101927043,EPCAM,EPCAM,MIR559,MSH2,KCNK12,MSH6,FBXO11,FOXN2,PPP1R21,STON1-GTF2A1L,STON1-GTF2A1L,STON1,STON1-GTF2A1L,GTF2A1L,STON1-GTF2A1L,LHCGR,FSHR,NRXN1,MIR4431,ASB3,GPR75-ASB3,ASB3,GPR75-ASB3,CHAC2,GPR75-ASB3,ERLEC1,GPR75-ASB3,MIR3682,GPR75-ASB3,GPR75,PSME4,ACYP2,ACYP2,TSPYL6,C2orf73,SPTBN1,SPTBN1,RPL23AP32,EML6,RTN4,CLHC1,RPS27A,RPS27A,MIR4426,MTIF2,CCDC88A | -0.209216 | 246.403 | 1191 | 716.02 |
| 1 | 2 | 2.28E+08 | 231678908 | RHBDD1,COL4A4,COL4A3,COL4A3,LOC654841,MFF,TM4SF20,MIR5703,AGFG1,AGFG1,C2orf83,SLC19A3,CCL20,DAW1,SPHKAP,PID1,DNER,TRIP12,TRIP12,FBXO36,FBXO36,SLC16A14,SP110,SP140,SP140L,SP100,CAB39 | -0.211518 | 237.36 | 493 | 286.176 |

| | | | | | | | | |
|---|---|---|---|---|---|---|---|---|
| 1 | 8 | 30464517 | 32405654 | GTF2E2,GTF2E2,SMIM18,GSR,UBXN8,PPP2CB,TEX15,PURG,WRN,NRG1 | -0.213198 | 274.866 | 149 | 91.6261 |
| 1 | 10 | 33197413 | 39076015 | ITGB1,NRP1,PARD3,CUL2,CUL2,MIR3611,CREM,CCNY,GJD4,FZD8,FZD8,MIR4683,ANKRD30A,ZNF248,ZNF25,ZNF33A,ZNF37A,HSD17B7P2,SEPT7P9,LINC00999 | -0.213341 | 243.051 | 299 | 168.646 |
| 1 | 13 | 84453605 | 88484001 | SLITRK1,SLITRK6,MIR4500HG,MIR4500,SLITRK5 | -0.213418 | 244.296 | 59 | 39.372 |
| 1 | 3 | 1.5E+08 | 153994677 | ERICH6,ERICH6,ERICH6-AS1,SIAH2,MINDY4B,CLRN1,CLRN1,CLRN1-AS1,MED12L,MED12L,GPR171,MED12L,P2RY14,MED12L,GPR87,MED12L,P2RY13,MED12L,P2RY12,IGSF10,MIR5186,MIR548H2,AADACL2,MIR548H2,AADACL2,AADACL2-AS1,MIR548H2,AADACL2-AS1,AADAC,AADACL2-AS1,AADAC,AADACL2-AS1,SUCNR1,MBNL1-AS1,MBNL1,MBNL1,MBNL1,TMEM14EP,P2RY1,RAP2B,LINC02006,C3orf79,ARHGEF26,DHX36 | -0.217052 | 266.052 | 246 | 152.175 |
| 1 | 10 | 7788511 | 27284161 | ITIH2,KIN,ATP5F1C,TAF3,GATA3-AS1,GATA3,LOC101928272,CELF2,CELF2,CELF2-AS1,USP6NL,ECHDC3,PROSER2,PROSER2-AS1,UPF2,DHTKD1,SEC61A2,SEC61A2,NUDT5,NUDT5,CDC123,CAMK1D,CAMK1D,MIR4480,CAMK1D,MIR4481,CAMK1D,MIR548Q,CCDC3,OPTN,MCM10,UCMA,PHYH,SEPHS1,BEND7,PRPF18,FRMD4A,MIR4293,FAM107B,CDNF,HSPA14,SUV39H2,SUV39H2,DCLRE1C,DCLRE1C,MEIG1,DCLRE1CP1,ACBD7-DCLRE1CP1,ACBD7-DCLRE1CP1,OLAH,ACBD7-DCLRE1CP1,ACBD7,C10orf111,RPP38,NMT2,NMT2,PPIAP30,FAM171A1,ITGA8,MINDY3,PTER,C1QL3,RSU1,CUBN,TRDMT1,VIM-AS1,VIM,VIM,ST8SIA6,ST8SIA6,ST8SIA6-AS1,HACD1,STAM,TMEM236,MRC1,MRC1,MIR511,MRC1,TMEM236,SLC39A12,SLC39A12,SLC39A12-AS1,CACNB2,NSUN6,ARL5B,MALRD1,PLXDC2,MIR4675,NEBL,NEBL,C10orf113,CASC10,CASC10,MIR1915,SKIDA1,MLLT10,DNAJC1,EBLN1,LOC100130992,COMMD3,COMMD3-BMI1,COMMD3-BMI1,BMI1,SPAG6,PIP4K2A,ARMC3,MSRB2,PTF1A,C10orf67,OTUD1,KIAA1217,KIAA1217,MIR603,ARHGAP21,PRTFDC1,ENKUR,ENKUR,THNSL1,GPR158-AS1,GPR158,GPR158,MYO3A,GAD2,APBB1IP,PDSS1,ABI1 | -0.220897 | 243.417 | 1551 | 900.579 |

| | | | | | | | | |
|---|---|---|---|---|---|---|---|---|
| 1 | 3 | 15253612 | 37584158 | CAPN7,SH3BP5-AS1,SH3BP5,SH3BP5,METTL6,EAF1,COLQ,COLQ,MIR4270,HACL1,HACL1,BTD,BTD,ANKRD28,ANKRD28,MIR3134,MIR563,GALNT15,DPH3,OXNAD1,OXNAD1,RFTN1,RFTN1,DAZL,PLCL2,PLCL2,MIR3714,TBC1D5,LOC339862,SATB1,KCNH8,KCNH8,MIR4791,EFHB,RAB5A,RAB5A,PP2D1,PP2D1,KAT2B,KAT2B,MIR3135A,SGO1,SGO1,SGO1-AS1,ZNF385D,ZNF385D,ZNF385D-AS1,UBE2E2,UBE2E2,MIR548AC,UBE2E1,NKIRAS1,RPL15,NR1D2,THRB,THRB,LOC101927854,MIR4792,RARB,TOP2B,MIR4442,NGLY1,OXSM,LINC00692,LRRC3B,NEK10,SLC4A7,EOMES,CMC1,AZI2,ZCWPW2,RBMS3,TGFBR2,GADL1,MIR466,STT3B,OSBPL10,ZNF860,GPD1L,CMTM8,CMTM7,CMTM6,DYNC1LI1,CNOT10,TRIM71,CCR4,GLB1,GLB1,TMPPE,CRTAP,SUSD5,FBXL2,FBXL2,UBP1,UBP1,CLASP2,PDCD6IP,ARPP21,ARPP21,MIR128-2,STAC,DCLK3,TRANK1,EPM2AIP1,MLH1,LRRFIP2,LOC152048,GOLGA4,GOLGA4,C3orf35,ITGA9 | -0.221206 | 236.068 | 1384 | 822.32 |
| 1 | 18 | 9256633 | 14763934 | ANKRD12,TWSG1,RALBP1,PPP4R1,RAB31,TXNDC2,VAPA,APCDD1,NAPG,PIEZO2,PIEZO2,MIR6788,SLC35G4,GNAL,GNAL,CHMP1B,GNAL,MPPE1,MPPE1,IMPA2,ANKRD62,CIDEA,TUBB6,TUBB6,AFG3L2,AFG3L2,PRELID3A,SPIRE1,PSMG2,CEP76,PSMG2,PTPN2,SEH1L,CEP192,LDLRAD4,LDLRAD4,MIR5190,LDLRAD4,MIR4526,FAM210A,RNMT,MC5R,MC2R,ZNF519,ANKRD20A5P,POTEC,ANKRD30B | -0.22472 | 255.937 | 520 | 300.137 |
| 1 | 22 | 20609931 | 24237162 | ZNF74,SCARF2,KLHL22,MED15,POM121L4P,TMEM191A,PI4KA,PI4KA,SERPIND1,SNAP29,CRKL,LINC01637,AIFM3,LZTR1,THAP7,THAP7,THAP7-AS1,THAP7-AS1,TUBA3FP,P2RX6,SLC7A4,MIR649,LRRC74B,GGT2,HIC2,TMEM191C,PI4KAP2,UBE2L3,YDJC,CCDC116,SDF2L1,MIR301B,MIR130B,PPIL2,YPEL1,MAPK1,PPM1F,PPM1F,LOC100286925,TOP3B,PRAMENP,VPREB1,BMS1P20,ZNF280B,ZNF280A,PRAME,PRAME,LL22NC03-63E9.3,LL22NC03-63E9.3,GGTLC2,MIR5571,IGLL5,RSPH14,RSPH14,GNAZ,RAB36,BCR,IGLL1,DRICH1,GUSBP11,GUSBP11,RGL4,ZNF70,VPREB3,C22orf15,C22orf15,CHCHD10,CHCHD10,MMP11,SMARCB1,SMARCB1,DERL3,DERL3,SLC2A11,MIF-AS1,MIF | -0.225025 | 247.947 | 754 | 433.321 |

| | | | | | | | | |
|---|---|---|---|---|---|---|---|---|
| 1 | 7 | 6825551 | 34981501 | RSPH10B,RSPH10B2,CCZ1B,MIR3683,LOC100131257,C1GALT1,COL28A1,MIOS,RPA3,RPA3,UMAD1,UMAD1,GLCCI1,ICA1,NXPH1,NDUFA4,PHF14,THSD7A,TMEM106B,VWDE,SCIN,ARL4A,ETV1,DGKB,AGMO,MEOX2,ISPD,ISPD,ISPD-AS1,SOSTDC1,LRRC72,ANKMY2,BZW2,TSPAN13,AGR2,AGR3,AHR,SNX13,PRPS1L1,HDAC9,MIR1302-6,HDAC9,TWIST1,FERD3L,TWISTNB,TWISTNB,MIR3146,TMEM196,MACC1,ITGB8,ABCB5,SP8,SP4,SP4,MIR1183,DNAH11,DNAH11,CDCA7L,CDCA7L,RAPGEF5,STEAP1B,LOC541472,IL6,IL6,TOMM7,SNHG26,SNORD93,FAM126A,KLHL7,NUPL2,GPNMB,MALSU1,IGF2BP3,RPS2P32,TRA2A,CLK2P1,CCDC126,FAM221A,STK31,NPY,MPP6,GSDME,OSBPL3,CYCS,C7orf31,NPVF,MIR148A,NFE2L3,HNRNPA2B1,CBX3,SNX10,SNX10,LOC105375304,LOC441204,KIAA0087,C7orf71,SKAP2,HOXA1,HOXA2,HOXA3,HOXA4,HOXA-AS3,HOXA5,HOXA-AS3,HOXA6,HOXA-AS3,HOXA-AS3,HOXA7,HOXA7,HOXA9,HOXA10-HOXA9,HOXA10-HOXA9,HOXA10-AS,HOXA10-HOXA9,HOXA10-AS,MIR196B,HOXA10-HOXA9,HOXA10-AS,HOXA10,HOXA10-HOXA9,HOXA10,HOXA11,HOXA13,EVX1-AS,EVX1,HIBADH,HIBADH,TSL,TAX1BP1,JAZF1,JAZF1,JAZF1-AS1,JAZF1-AS1,CREB5,TRIL,LOC100506497,CPVL,CPVL,CPVL,CHN2,CHN2,PRR15,LOC646762,LOC646762,MIR550A3,WIPF3,SCRN1,FKBP14,PLEKHA8,MTURN,LOC105375218,ZNRF2,ZNRF2,MIR550A1,MIR550B1,ZNRF2,NOD1,GGCT,GARS,CRHR2,INMT,INMT-MINDY4,INMT-MINDY4,INMT-MINDY4,MINDY4,AQP1,GHRHR,ADCYAP1R1,NEUROD6,CCDC129,PPP1R17,PDE1C,LSM5,AVL9,AVL9,DPY19L | -0.227575 | 247.025 | 1948 | 1130.39 |

| | | | | | | | | |
|---|---|---|---|---|---|---|---|---|
| 1 | 17 | 70118901 | 81194710 | SOX9,SLC39A11,SSTR2,COG1,COG1,FAM104A,FAM104A,FAM104A,C17orf80,C17orf80,C17orf80,CPSF4L,CPSF4L,CDC42EP4,SDK2,LOC100134391,LINC00469,LINC00469,RPL38,TTYH2,DNAI2,KIF19,BTBD17,GPR142,GPRC5C,CD300A,CD300LB,CD300C,CD300LD,CD300LD,C17orf77,C17orf77,CD300E,RAB37,RAB37,CD300LF,SLC9A3R1,MIR3615,SLC9A3R1,NAT9,TMEM104,GRIN2C,FDXR,FADS6,USH1G,OTOP2,OTOP3,HID1,HID1,HID1-AS1,CDR2L,MRPL58,KCTD2,KCTD2,ATP5PD,SLC16A5,ARMC7,NT5C,JPT1,SUMO2,NUP85,GGA3,GGA3,MRPS7,MRPS7,MIF4GD,LOC100287042,LOC100287042,SLC25A19,SLC25A19,GRB2,MIR3678,TMEM94,TMEM94,MIR6785,CASKIN2,TSEN54,LLGL2,MYO15B,RECQL5,RECQL5,SMIM5,RECQL5,SMIM6,SAP30BP,ITGB4,GALK1,H3F3B,MIR4738,UNK,UNC13D,WBP2,TRIM47,TRIM65,MRPL38,FBF1,ACOX1,TEN1,TEN1-CDK3,TEN1-CDK3,CDK3,EVPL,SRP68,GALR2,ZACN,ZACN,EXOC7,EXOC7,EXOC7,MIR6868,FOXJ1,RNF157-AS1,RNF157,RNF157,UBALD2,QRICH2,PRPSAP1,SPHK1,UBE2O,AANAT,RHBDF2,CYGB,PRCD,PRCD,SNHG16,SNORD1C,SNHG16,SNHG16,SNORD1B,SNHG16,SNORD1A,ST6GALNAC2,ST6GALNAC1,MXRA7,JMJD6,METTL23,SRSF2,SRSF2,MIR636,SRSF2,MFSD11,MFSD11,LINC00868,MGAT5B,SNHG20,SEC14L1,SCARNA16,SNHG20,SEC14L1,SCARNA16,MIR6516,SEC14L1,LOC105371907,SEPT9,SEPT9,SEPT9,MIR4316,LINC01973,TNRC6C,TNRC6C,TNRC6C-AS1,TNRC6C-AS1,TMC6,TMC6,TMC8,TMC8,C17orf99,SYNGR2,TK1,AFMID,BIRC5,TMEM235,LINC01993,SOCS3,PGS1,PGS1,DNAH17,DNAH17,DNAH17,DNAH17-AS1,CYTH1,USP36,TIMP2,TIMP2,CEP295NL,LGALS3BP,CANT1,C1QTNF1-AS1,C1QTNF1,ENGASE,RBFOX3,MIR4739,LINC02078,E | -0.22858 | 248.3 | 2812 | 1759.16 |
| 1 | 15 | 57967148 | 60720911 | MYZAP,GCOM1,GCOM1,GCOM1,POLR2M,ALDH1A2,ALDH1A2,LOC283665,AQP9,LIPC,LIPC,LIPC-AS1,ADAM10,ADAM10,HSP90AB4P,MINDY2,SLTM,RNF111,CCNB2,MYO1E,MYO1E,MIR2116,MYO1E,LDHAL6B,FAM81A,GCNT3,GTF2A2,BNIP2,FOXB1,ANXA2,ICE2 | -0.230881 | 252.962 | 285 | 168.459 |
| 1 | 3 | 48789041 | 49019190 | PRKAR2A,SLC25A20,ARIH2OS,ARIH2OS,ARIH2,ARIH2 | -0.231185 | 236.331 | 51 | 29.0691 |
| 1 | 10 | 1.24E+08 | 123971231 | TACC2 | -0.232892 | 196.063 | 42 | 26.6981 |
| 1 | 11 | 69949150 | 70017156 | ANO1 | -0.234313 | 242.758 | 25 | 15.3085 |
| 1 | 17 | 36398155 | 37417873 | LOC440434,MRPL45,GPR179,SOCS7,ARHGAP23,SRCIN1,EPOP,MIR4734,MLLT6,MLLT6,MIR4726,CISD3,CISD3,PCGF2,PCGF2,LOC100287808,PSMB3,PIP4K2B,CWC25,MIR4727,C17orf98,RPL23,RPL23,SNORA21,LASP1,LASP1,MIR6779,FBXO47,LOC100131347,LOC100131347,PLXDC1,PLXDC1,ARL5C,CACNB1,RPL19,STAC2,FBXL20 | -0.234795 | 244.919 | 310 | 182.691 |
| 1 | 11 | 92717956 | 94152776 | SLC36A4,DEUP1,SMCO4,CEP295,CEP295,SCARNA9,CEP295,TAF1D,TAF1D,SNORA25,TAF1D,SNORA32,TAF1D,SNORD6,TAF1D,SNORA1,TAF1D,SNORA8,TAF1D,SNORD5,TAF1D,SNORA18,TAF1D,TAF1D,SNORA40,C11orf54,MED17,VSTM5,HEPHL1,PANX1,IZUMO1R,GPR83 | -0.234887 | 260.033 | 169 | 104.002 |

| | | | | | | | | |
|---|---|---|---|---|---|---|---|---|
| 1 | 19 | 41884156 | 42375403 | TMEM91,EXOSC5,BCKDHA,B3GNT8,DMAC2,ERICH4,PCAT19,CEACAM21,CEACAM4,CEACAM7,CEACAM5,CEACAM6,CEACAM3,LYPD4,DMRTC2,RPS19,RPS19,MIR6797 | -0.236372 | 282.496 | 125 | 73.1898 |
| 1 | 19 | 48994271 | 49979912 | LMTK3,SULT2B1,FAM83E,FAM83E,SPACA4,RPL18,SPHK2,DBP,CA11,SEC1P,SEC1P,NTN5,SEC1P,FUT2,LOC105447645,MAMSTR,RASIP1,IZUMO1,FUT1,FGF21,BCAT2,HSD17B14,PLEKHA4,PPP1R15A,TULP2,NUCB1,NUCB1,NUCB1-AS1,DHDH,BAX,FTL,GYS1,RUVBL2,RUVBL2,MIR6798,RUVBL2,LHB,LHB,LOC101059948,CGB2,CGB1,CGB7,NTF4,KCNA7,SNRNP70,LIN7B,LIN7B,C19orf73,C19orf73,PPFIA3,HRC,TRPM4,SLC6A16,SLC6A16,MIR4324,CD37,TEAD2,DKKL1,DKKL1,LOC101928295,CCDC155,PTH2,GFY,SLC17A7,PIH1D1,ALDH16A1,FLT3LG | -0.237147 | 251.004 | 492 | 302.815 |
| 1 | 2 | 1.72E+08 | 179395177 | DCAF17,CYBRD1,DYNC1I2,SLC25A12,HAT1,METAP1D,DLX1,DLX2,ITGA6,PDK1,RAPGEF4-AS1,RAPGEF4,RAPGEF4,MAP3K20,MAP3K20,MAP3K20-AS1,CDCA7,SP3,OLA1,SP9,CIR1,CIR1,SCRN3,SCRN3,GPR155,WIPF1,CHRNA1,CHN1,ATF2,ATF2,MIR933,ATP5MC3,LNPK,EVX2,HOXD13,HOXD12,HOXD11,HOXD10,HOXD9,HOXD8,MIR10B,HOXD4,HOXD3,HAGLR,HOXD1,MIR7704,HOXD1,MTX2,MIR1246,HNRNPA3,NFE2L2,NFE2L2,MIR3128,AGPS,TTC30B,TTC30A,PDE11A,PDE11A,LOC105373764,RBM45,OSBPL6,OSBPL6,MIR548N,MIR548N,LOC101927027,PRKRA,MIR548N,PRKRA,MIR548N,PJVK,MIR548N,FKBP7,MIR548N,PLEKHA3,MIR548N,TTN-AS1,TTN | -0.24109 | 256.282 | 672 | 391.325 |
| 1 | 15 | 42453783 | 42858951 | VPS39,VPS39,MIR627,TMEM87A,GANC,CAPN3,ZNF106,SNAP23,LRRC57,HAUS2 | -0.244478 | 256.971 | 171 | 106.123 |
| 1 | 15 | 51250719 | 56657956 | AP4E1,MIR4713HG,TNFAIP8L3,MIR4713HG,CYP19A1,MIR4713HG,CYP19A1,MIR4713,GLDN,DMXL2,SCG3,LYSMD2,TMOD2,TMOD3,LEO1,MAPK6,BCL2L10,GNB5,GNB5,CERNA1,CERNA1,MYO5C,MYO5C,MYO5C,MIR1266,MYO5A,ARPP19,FAM214A,ONECUT1,WDR72,UNC13C,RSL24D1,RAB27A,PIGB,PIGB,CCPG1,DNAAF4-CCPG1,CCPG1,DNAAF4-CCPG1,CCPG1,DNAAF4-CCPG1,MIR628,DNAAF4-CCPG1,C15orf65,DNAAF4-CCPG1,C15orf65,DNAAF4,DNAAF4-CCPG1,DNAAF4,PYGO1,PRTG,NEDD4,RFX7,TEX9 | -0.251593 | 261.055 | 591 | 352.795 |

| | | | | | | | | |
|---|---|---|---|---|---|---|---|---|
| 1 | 17 | 45664545 | 49076008 | NPEPPS,KPNB1,TBKBP1,TBX21,OSBPL7,MRPL10,LRRC46,SCRN2,SP6,SP2,SP2,SP2-AS1,PNPO,PRR15L,CDK5RAP3,COPZ2,COPZ2,MIR152,NFE2L1,CBX1,SNX11,SKAP1,SKAP1,MIR1203,HOXB1,HOXB2,HOXB2,HOXB-AS1,HOXB-AS1,HOXB3,HOXB3,HOXB3,HOXB4,HOXB3,MIR10A,HOXB-AS3,HOXB5,HOXB-AS3,HOXB6,HOXB7,HOXB8,HOXB9,MIR196A1,PRAC1,PRAC2,MIR3185,HOXB13,TTLL6,CALCOCO2,LOC105371814,ATP5MC1,LOC105371814,UBE2Z,UBE2Z,SNF8,GIP,IGF2BP1,B4GALNT2,GNGT2,ABI3,PHOSPHO1,FLJ40194,ZNF652,PHB,NGFR,NGFR,LOC100288866,NXPH3,SPOP,SLC35B1,FAM117A,KAT7,TAC4,TAC4,FLJ45513,DLX4,DLX3,ITGA3,PDK2,SAMD14,PPP1R9B,SGCA,SGCA,HILS1,COL1A1,TMEM92,XYLT2,MRPL27,MRPL27,EME1,EME1,LRRC59,ACSF2,ACSF2,CHAD,RSAD1,MYCBPAP,EPN3,EPN3,LOC105371824,SPATA20,CACNA1G-AS1,CACNA1G,CACNA1G,ABCC3,ANKRD40,LUC7L3,LINC00483,WFIKKN2,TOB1,SPAG9 | -0.252608 | 245.724 | 902 | 555.104 |
| 1 | 19 | 43982192 | 48544923 | PHLDB3,ETHE1,ZNF575,XRCC1,PINLYP,IRGQ,ZNF576,ZNF428,ZNF428,SRRM5,CADM4,PLAUR,IRGC,SMG9,KCNN4,LYPD5,ZNF283,ZNF404,ZNF45,ZNF221,ZNF155,ZNF155,LOC101928063,LOC101928063,ZNF230,ZNF222,ZNF223,ZNF284,ZNF224,ZNF224,LOC100379224,ZNF225,ZNF234,ZNF226,ZNF227,ZNF233,ZNF235,ZNF112,ZNF285,ZNF229,ZNF180,CEACAM20,IGSF23,PVR,PVR,MIR4531,CEACAM19,CEACAM16,BCL3,BCL3,MIR8085,CBLC,BCAM,NECTIN2,TOMM40,APOE,APOC1,APOC1P1,APOC4,APOC4-APOC2,APOC4-APOC2,APOC2,CLPTM1,RELB,CLASRP,ZNF296,GEMIN7,GEMIN7-AS1,GEMIN7,PPP1R37,NKPD1,TRAPPC6A,BLOC1S3,EXOC3L2,MARK4,CKM,KLC3,ERCC2,PPP1R13L,CD3EAP,CD3EAP,ERCC1,ERCC1,FOSB,RTN2,PPM1N,VASP,OPA3,GPR4,EML2,EML2,MIR330,EML2,EML2-AS1,GIPR,GIPR,MIR642A,MIR642B,SNRPD2,QPCTL,FBXO46,BHMG1,SIX5,SIX5,DM1-AS,DM1-AS,DMPK,DMPK,DMWD,RSPH6A,SYMPK,FOXA3,IRF2BP1,MYPOP,NANOS2,NOVA2,CCDC61,MIR769,PGLYRP1,IGFL4,IGFL3,IGFL2,IGFL1,HIF3A,PPP5C,CCDC8,PNMA8A,PPP5D1,PPP5D1,PNMA8B,CALM3,PTGIR,GNG8,DACT3,DACT3,DACT3-AS1,DACT3-AS1,PRKD2,PRKD2,PRKD2,MIR320E,STRN4,STRN4,FKRP,FKRP,SLC1A5,AP2S1,ARHGAP35,NPAS1,TMEM160,ZC3H4,SAE1,BBC3,BBC3,MIR3190,MIR3191,CCDC9,INAFM1,C5AR1,C5AR2,DHX34,MEIS3,SLC8A2,KPTN,NAPA-AS1,NAPA,NAPA,ZNF541,BICRA,EHD2,NOP53,NOP53,SNORD23,NOP53,NOP53-AS1,SELENOW,TPRX1,CRX,SULT2A1,BSPH1,ELSPBP1,CABP5 | -0.252683 | 254.852 | 1434 | 880.14 |

| | | | | | | | | |
|---|---|---|---|---|---|---|---|---|
| 1 | 1 | 10500 | 35450977 | FAM87B,LINC00115,LINC01128,SAMD11,SAMD11,NOC2L,NOC2L,KLHL17,PLEKHN1,PERM1,HES4,ISG15,AGRN,RNF223,C1orf159,LINC01342,MIR200B,MIR200A,MIR429,TTLL10,TNFRSF18,TNFRSF4,SDF4,B3GALT6,C1QTNF12,UBE2J2,SCNN1D,ACAP3,ACAP3,MIR6726,PUSL1,PUSL1,INTS11,INTS11,INTS11,MIR6727,CPTP,TAS1R3,DVL1,DVL1,MIR6808,MXRA8,AURKAIP1,CCNL2,LOC148413,MRPL20,ANKRD65,TMEM88B,VWA1,ATAD3C,ATAD3B,ATAD3A,TMEM240,SSU72,FNDC10,LOC105378586,MIB2,MMP23B,MMP23B,MMP23A,CDK11B,CDK11B,SLC35E2B,CDK11B,MMP23A,CDK11B,CDK11A,SLC35E2,NADK,GNB1,CALML6,TMEM52,CFAP74,GABRD,PRKCZ,PRKCZ,PRKCZ-AS1,PRKCZ,FAAP20,FAAP20,SKI,MORN1,RER1,PEX10,PLCH2,PANK4,HES5,TNFRSF14-AS1,TNFRSF14,TNFRSF14,FAM213B,FAM213B,MMEL1,MMEL1,TTC34,ACTRT2,LINC00982,PRDM16,PRDM16,MIR4251,ARHGEF16,MEGF6,MEGF6,MIR551A,TPRG1L,WRAP73,TP73,TP73-AS1,CCDC27,SMIM1,LRRC47,CEP104,DFFB,C1orf174,AJAP1,MIR4417,MIR4689,NPHP4,NPHP4,KCNAB2,CHD5,RPL22,RNF207,ICMT,HES3,GPR153,ACOT7,HES2,ESPN,ESPN,MIR4252,TNFRSF25,TNFRSF25,PLEKHG5,PLEKHG5,NOL9,TAS1R1,ZBTB48,KLHL21,PHF13,THAP3,THAP3,DNAJC11,DNAJC11,CAMTA1,VAMP3,PER3,UTS2,TNFRSF9,PARK7,ERRFI1,SLC45A1,RERE,ENO1,ENO1,MIR6728,CA6,SLC2A7,SLC2A5,GPR157,MIR34AHG,MIR34A,MIR34AHG,LINC01759,H6PD,SPSB1,SLC25A33,TMEM201,PIK3CD,PIK3CD-AS1,PIK3CD,CLSTN1,CTNNBIP1,LZIC,NMNAT1,MIR5697,NMNAT1,RBP7,UBE4B,KIF1B,PGD,CENPS,CENPS-CORT,CENPS-CORT,CORT,DFFA,PEX14,CASZ1,C1orf127,TARDBP,MA | -0.254332 | 237.144 | 6415 | 3873.44 |
| 1 | 18 | 48452141 | 65181867 | ME2,ELAC1,SMAD4,MEX3C,LINC01630,DCC,DCC,MIR4528,MBD2,MBD2,SNORA37,POLI,STARD6,C18orf54,DYNAP,RAB27B,CCDC68,TCF4,TCF4,TCF4-AS1,TCF4,TCF4-AS1,MIR4529,LOC642484,LOC642484,LINC01905,TXNL1,WDR7,BOD1L2,ST8SIA3,ONECUT2,FECH,NARS,LOC100505549,LOC100505549,ATP8B1,ATP8B1,NEDD4L,MIR122,MIR3591,ALPK2,LOC101927322,MALT1,MALT1,ZNF532,OACYLP,SEC11C,GRP,RAX,CPLX4,LMAN1,CCBE1,PMAIP1,MC4R,CDH20,RNF152,PIGN,KIAA1468,TNFRSF11A,ZCCHC2,PHLPP1,BCL2,KDSR,VPS4B,SERPINB5,SERPINB12,SERPINB13,SERPINB4,SERPINB3,SERPINB11,SERPINB7,SERPINB2,SERPINB10,HMSD,SERPINB8,LINC00305,CDH7,CDH19,MIR5011,DSEL | -0.258165 | 250.465 | 940 | 563.312 |

| | | | | | | | | |
|---|---|---|---|---|---|---|---|---|
| 1 | 2 | 1.54E+08 | 170382259 | PRPF40A,ARL6IP6,RPRM,GALNT13,GALNT13,LOC100144595,KCNJ3,NR4A2,GPD2,GALNT5,ERMN,CYTIP,ACVR1C,ACVR1,UPP2,CCDC148-AS1,CCDC148,CCDC148,PKP4,PKP4,PKP4-AS1,DAPL1,TANC1,TANC1,MIR6888,WDSUB1,BAZ2B,MARCH7,CD302,LY75-CD302,LY75-CD302,LY75,PLA2R1,ITGB6,RBMS1,TANK,TANK,LOC101929512,PSMD14,TBR1,AHCTF1P1,SLC4A10,DPP4,LOC101929532,GCG,LOC101929532,FAP,FAP,IFIH1,GCA,KCNH7,FIGN,GRB14,COBLL1,COBLL1,SNORA70F,COBLL1,LOC101929633,SLC38A11,SCN3A,SCN2A,CSRNP3,GALNT3,TTC21B,TTC21B,TTC21B-AS1,LOC102724058,SCN1A,SCN1A,LOC101929680,SCN9A,SCN9A,SCN7A,XIRP2,XIRP2,XIRP2-AS1,LOC105616981,B3GALT1,STK39,CERS6,CERS6,MIR4774,NOSTRIN,SPC25,G6PC2,ABCB11,DHRS9,LRP2,BBS5,KLHL41 | -0.259524 | 264.029 | 1336 | 808.343 |
| 1 | 6 | 88332301 | 119532146 | ORC3,AKIRIN2,SPACA1,CNR1,RNGTT,PNRC1,SRSF12,PM20D2,GABRR1,GABRR2,UBE2J1,RRAGD,ANKRD6,LYRM2,LYRM2,LOC101929057,LOC101929057,MDN1,MDN1,CASP8AP2,GJA10,BACH2,MIR4464,MAP3K7,MIR4643,EPHA7,MANEA,FUT9,UFL1,FHL5,GPR63,NDUFAF4,KLHL32,KLHL32,MIR548H3,MIR548H3,MMS22L,MIR2113,POU3F2,FBXL4,MIR548AI,FAXC,COQ3,PNISR,USP45,TSTD3,CCNC,PRDM13,MCHR2,SIM1,ASCC3,GRIK2,HACE1,LIN28B-AS1,LIN28B,BVES,BVES-AS1,BVES-AS1,POPDC3,PREP,PRDM1,ATG5,LOC105377924,CRYBG1,RTN4IP1,RTN4IP1,QRSL1,QRSL1,LINC02532,MIR587,C6orf203,BEND3,PDSS2,SOBP,SCML4,SEC63,OSTM1,NR2E1,SNX3,AFG1L,FOXO3,ARMC2,ARMC2,ARMC2-AS1,SESN1,CEP57L1,CCDC162P,C6orf183,CCDC162P,CD164,PPIL6,PPIL6,SMPD2,SMPD2,MICAL1,MICAL1,ZBTB24,ZBTB24,AK9,FIG4,GPR6,WASF1,CDC40,METTL24,DDO,SLC22A16,CDK19,CDK19,AMD1,AMD1,GTF3C6,RPF2,GSTM2P1,SLC16A10,MFSD4B,REV3L,REV3L,TRAF3IP2-AS1,TRAF3IP2-AS1,TRAF3IP2-AS1,TRAF3IP2,FYN,WISP3,TUBE1,FAM229B,LAMA4,LAMA4,LOC101927640,RFPL4B,MARCKS,HDAC2,HDAC2,HDAC2-AS2,HDAC2-AS2,HS3ST5,FRK,NT5DC1,NT5DC1,COL10A1,TSPYL4,DSE,DSE,TSPYL1,DSE,LOC100287467,CALHM6,TRAPPC3L,TRAPPC3L,CALHM5,TRAPPC3L,CALHM4,CALHM4,RWDD1,RSPH4A,ZUFSP,KPNA5,FAM162B,GPRC6A,RFX6,VGLL2,ROS1,DCBLD1,DCBLD1,GOPC,GOPC,NUS1,SLC35F1,CEP85L,CEP85L,PLN,MCM9,MCM9,ASF1A,FAM184A,FAM184A,MIR548B,MAN1A1 | -0.261176 | 250.242 | 2100 | 1240.11 |
| 1 | 7 | 1.44E+08 | 148767771 | ARHGEF5,NOBOX,TPK1,CNTNAP2,CNTNAP2,MIR548F4,CNTNAP2,MIR548T,C7orf33,CUL1,EZH2,PDIA4,ZNF786 | -0.263661 | 209.449 | 195 | 112.797 |

| | | | | | | | | |
|---|---|---|---|---|---|---|---|---|
| 1 | 11 | 94229984 | 102933229 | ANKRD49,C11orf97,FUT4,FUT4,LOC105369438,LOC105369438,PIWIL4,LOC105369438,AMOTL1,CWC15,KDM4D,KDM4E,SRSF8,ENDOD1,SESN3,SESN3,LOC100129203,FAM76B,CEP57,MTMR2,MAML2,MAML2,MIR1260B,CCDC82,JRKL,CNTN5,LOC100128386,ARHGAP42,ARHGAP42,PGR,PGR,PGR-AS1,TRPC6,TRPC6,MIR3920,ANGPTL5,ANGPTL5,CEP126,CEP126,C11orf70,YAP1,BIRC3,BIRC2,TMEM123,LOC102723838,MMP7,MMP20,MMP27,MMP8,MMP10,WTAPP1,MMP1,WTAPP1,MMP3,MMP3,MMP12,MMP13,DCUN1D5 | -0.268217 | 249.261 | 552 | 328.35 |
| 1 | 10 | 51885769 | 55996729 | WASHC2A,ASAH2,SGMS1,SGMS1-AS1,ASAH2B,A1CF,PRKG1,PRKG1,MIR605,PRKG1,CSTF2T,DKK1,MBL2,PCDH15 | -0.269185 | 223.547 | 176 | 104.16 |
| 1 | 17 | 6003 | 4574883 | DOC2B,RPH3AL,RPH3AL,LOC100506388,LOC105371430,C17orf97,RFLNB,VPS53,FAM57A,GEMIN4,GLOD4,GLOD4,MRM3,MRM3,NXN,TIMM22,ABR,ABR,MIR3183,BHLHA9,TUSC5,YWHAE,CRK,MYO1C,INPP5K,PITPNA,SLC43A2,SCARF1,RILP,PRPF8,TLCD2,MIR22HG,MIR22HG,MIR22,WDR81,SERPINF2,SERPINF1,SMYD4,RPA1,RTN4RL1,DPH1,DPH1,OVCA2,HIC1,SMG6,SRR,SRR,TSR1,TSR1,TSR1,SNORD91B,TSR1,SNORD91A,SGSM2,MNT,LOC284009,METTL16,PAFAH1B1,CLUH,CLUH,MIR6776,CCDC92B,MIR1253,RAP1GAP2,RAP1GAP2,LOC101927911,OR1D5,OR1D2,OR1G1,OR1A2,OR1A1,OR1D4,OR3A2,OR3A1,OR3A4P,OR1E1,OR3A3,OR1E2,SPATA22,SPATA22,ASPA,TRPV3,TRPV1,SHPK,CTNS,TAX1BP3,P2RX5-TAX1BP3,P2RX5-TAX1BP3,EMC6,P2RX5-TAX1BP3,P2RX5-TAX1BP3,P2RX5,ITGAE,ITGAE,HASPIN,NCBP3,CAMKK1,P2RX1,ATP2A3,ZZEF1,CYB5D2,ANKFY1,UBE2G1,SPNS3,SPNS2,SPNS2,MYBBP1A,MYBBP1A,GGT6,SMTNL2,LINC01996,ALOX15,PELP1 | -0.270502 | 238.209 | 1096 | 672.736 |
| 1 | 10 | 27434367 | 29821058 | YME1L1,MASTL,MASTL,ACBD5,ACBD5,LRRC37A6P,ARMC4P1,PTCHD3,RAB18,MKX,ARMC4,MPP7,WAC,BAMBI,C10orf126,LYZL1,SVIL-AS1,SVIL,SVIL | -0.272719 | 255.094 | 215 | 124.463 |
| 1 | 6 | 1.25E+08 | 136681223 | RNF217-AS1,RNF217-AS1,RNF217,RNF217,TPD52L1,HDDC2,HEY2,NCOA7,NCOA7-AS1,NCOA7,HINT3,TRMT11,CENPW,RSPO3,RNF146,ECHDC1,KIAA0408,SOGA3,SOGA3,C6orf58,THEMIS,PTPRK,PTPRK,LOC101928140,LAMA2,ARHGAP18,TMEM244,L3MBTL3,SAMD3,TMEM200A,SMLR1,EPB41L2,AKAP7,ARG1,ARG1,MED23,MED23,ENPP3,ENPP3,OR2A4,MIR548H5,ENPP1,CTGF,MIR548AJ1,MOXD1,STX7,TAAR9,TAAR8,TAAR6,TAAR5,TAAR3P,TAAR2,TAAR1,VNN1,VNN3,VNN2,SLC18B1,RPS12,RPS12,SNORD101,RPS12,SNORD100,RPS12,SNORA33,EYA4,EYA4,TARID,TCF21,TBPL1,SLC2A12,SGK1,ALDH8A1,HBS1L,HBS1L,MIR3662,MYB,MIR548A2,AHI1,LINC00271,PDE7B,MTFR2,BCLAF1,MAP7 | -0.277429 | 265.867 | 942 | 562.011 |

| | | | | | | | | |
|---|---|---|---|---|---|---|---|---|
| 1 | 1 | 45811564 | 47748180 | TESK2,CCDC163,MMACHC,PRDX1,AKR1A1,NASP,CCDC17,GPBP1L1,TMEM69,TMEM69,IPP,IPP,MAST2,PIK3R3,LOC110117498-PIK3R3,LOC110117498-PIK3R3,TSPAN1,LOC110117498,TSPAN1,POMGNT1,POMGNT1,LURAP1,RAD54L,LRRC41,UQCRH,NSUN4,FAAH,DMBX1,MKNK1-AS1,KNCN,MKNK1-AS1,MKNK1,MKNK1,MOB3C,ATPAF1,TEX38,EFCAB14-AS1,EFCAB14,EFCAB14,CYP4B1,CYP4Z2P,CYP4A11,CYP4X1,CYP4Z1,CYP4A22,PDZK1IP1,TAL1,STIL | -0.278498 | 246.628 | 451 | 271.004 |
| 1 | 15 | 60770053 | 62212519 | ICE2,RORA-AS1,RORA,RORA,VPS13C | -0.280198 | 226.623 | 68 | 39.8137 |
| 1 | 2 | 1.38E+08 | 153514580 | THSD7B,HNMT,SPOPL,NXPH2,LRP1B,KYNU,ARHGAP15,LOC101928386,GTDC1,GTDC1,ZEB2,PABPC1P2,ACVR2A,ORC4,MBD5,EPC2,KIF5C,LYPD6B,LYPD6,MMADHC,RND3,RBM43,NMI,LOC101929319,TNFAIP6,LOC101929319,TNFAIP6,MIR4773-1,MIR4773-2,RIF1,NEB,ARL5A,CACNB4,STAM2,FMNL2,PRPF40A | -0.282976 | 248.209 | 811 | 485.593 |
| 1 | 9 | 95048771 | 95272410 | IARS,IARS,MIR3651,SNORA84,NOL8,CENPP,CENPP,OGN,CENPP,OMD,CENPP,ASPN,CENPP,ECM2 | -0.283026 | 299.447 | 73 | 42.8159 |
| 1 | 14 | 21679440 | 21841672 | HNRNPC,RPGRIP1,SUPT16H | -0.289017 | 248.409 | 70 | 41.7566 |
| 1 | 17 | 8273523 | 9153381 | KRBA2,RPL26,RNF222,NDEL1,MYH10,CCDC42,SPDYE4,MFSD6L,PIK3R6,PIK3R5,NTN1 | -0.290348 | 234.439 | 155 | 93.8351 |
| 1 | 22 | 24396201 | 45745685 | GSTTP2,CABIN1,SUSD2,GGT5,SPECC1L,SPECC1L-ADORA2A,SPECC1L-ADORA2A,ADORA2A,ADORA2A-AS1,UPB1,GUCD1,GUCD1,SNRPD3,SNRPD3,GGT1,GGT1,LRRC75B,BCRP3,PIWIL3,SGSM1,TMEM211,KIAA1671,CRYBB3,CRYBB2,IGLL3P,LRP5L,CRYBB2P1,GRK3,MYO18B,SEZ6L,ASPHD2,ASPHD2,HPS4,HPS4,SRRD,SRRD,TFIP11,TFIP11,TPST2,TPST2,MIR548J,CRYBB1,CRYBA4,LOC284898,MN1,PITPNB,TTC28-AS1,MIR3199-1,MIR3199-2,TTC28-AS1,TTC28,TTC28,CHEK2,HSCB,CCDC117,XBP1,ZNRF3,C22orf31,KREMEN1,EMID1,RHBDD3,EWSR1,GAS2L1,RASL10A,AP1B1,AP1B1,MIR3653,SNORD125,RFPL1S,RFPL1S,RFPL1,NEFH,THOC5,NIPSNAP1,NF2,CABP7,CABP7,ZMAT5,ZMAT5,UQCR10,ASCC2,MTMR3,MTMR3,HORMAD2-AS1,HORMAD2,LIF,LIF,LOC91370,OSM,CASTOR1,TBC1D10A,SF3A1,SF3A1,CCDC157,CCDC157,CCDC157,KIAA1656,RNF215,SEC14L2,MTFP1,LOC105372990,SEC14L3,SEC14L4,SEC14L6,GAL3ST1,PES1,TCN2,SLC35E4,SLC35E4,DUSP18,OSBP2,OSBP2,MIR3200,OSBP2,LOC107985544,MORC2-AS1,MORC2,SMTN,SELENOM,INPP5J,PLA2G3,MIR3928,RNF185,LIMK2,PIK3IP1,PATZ1,PATZ1,PIK3IP1-AS1,LINC01521,DRG1,EIF4ENIF1,SFI1,PISD,PISD,MIR7109,PRR14L,DEPDC5,C22orf24,C22orf24,YWHAH,YWHAH,SLC5A1,C22orf42,RFPL2,SLC5A4-AS1,SLC5A4,RFPL3,RFPL3,RFPL3S,RFPL3S,RTCB,BPIFC,FBXO7,SYN3,SYN3,TIMP3,LARGE1,LARGE1,MIR4764,ISX,HMGXB4,TOM1,TOM1,MIR3909,HMOX1,MCM5,RASD2,MB,APOL6,APOL5,RBFOX2,APOL3,APOL4,APOL2,APOL1,MYH9,TXN2,FOXRED2,EIF3D,CACNG2,LOC105373021,IFT27,IFT27,PVALB,NCF4-AS1,NCF4,NCF4,CSF2RB,TEX33,TST,TST,MPST,MPST,K | -0.291455 | 232.157 | 3761 | 2260.48 |

| | | | | | | | | |
|---|---|---|---|---|---|---|---|---|
| 1 | 1 | 35864462 | 42045722 | ZMYM4,KIAA0319L,NCDN,TFAP2E,PSMB2,C1orf216,CLSPN,AGO4,AGO1,AGO3,TEKT2,ADPRHL2,COL8A2,TRAPPC3,MAP7D1,THRAP3,SH3D21,EVA1B,STK40,LSM10,OSCP1,MRPS15,CSF3R,GRIK3,MIR4255,ZC3H12A,ZC3H12A,MIR6732,MEAF6,MEAF6,MIR5581,SNIP1,DNALI1,GNL2,RSPO1,C1orf109,CDCA8,EPHA10,MANEAL,YRDC,YRDC,C1orf122,C1orf122,MTF1,INPP5B,SF3A3,FHL3,UTP11,POU3F1,MIR3659,LINC01343,RRAGC,MYCBP,GJA9-MYCBP,MYCBP,GJA9-MYCBP,LOC105378663,GJA9-MYCBP,LOC105378663,GJA9-MYCBP,LOC105378663,GJA9,GJA9-MYCBP,GJA9,RHBDL2,AKIRIN1,NDUFS5,MACF1,MACF1,KIAA0754,BMP8A,BMP8A,OXCT2P1,BMP8A,PPIEL,PABPC4,PABPC4,LOC101929516,PABPC4,LOC101929516,SNORA55,HEYL,NT5C1A,HPCAL4,PPIE,PPIE,BMP8B,BMP8B,BMP8B,OXCT2,TRIT1,MYCL,MFSD2A,CAP1,PPT1,RLF,TMCO2,ZMPSTE24,COL9A2,SMAP2,ZFP69B,ZFP69,EXO5,ZNF684,RIMS3,NFYC-AS1,NFYC,NFYC,NFYC,MIR30E,NFYC,MIR30C1,KCNQ4,CITED4,CTPS1,SLFNL1-AS1,SLFNL1,SLFNL1-AS1,SCMH1,SCMH1,FOXO6,EDN2,HIVEP3 | -0.291764 | 226.269 | 1157 | 711.867 |
| 1 | 7 | 1.03E+08 | 126172518 | 2,PMPCB,PMPCB,DNAJC2,DNAJC2,PSMC2,PSMC2,SLC26A5,SLC26A5,LOC101927870,RELN,RELN,ORC5,LHFPL3,LHFPL3,LHFPL3-AS2,KMT2E,SRPK2,PUS7,RINT1,RINT1,EFCAB10,EFCAB10,ATXN7L1,CDHR3,SYPL1,NAMPT,CCDC71L,PIK3CG,PRKAR2B,HBP1,COG5,COG5,GPR22,COG5,DUS4L,DUS4L,BCAP29,SLC26A4-AS1,SLC26A4,SLC26A4,LOC101927974,CBLL1,CBLL1,SLC26A3,DLD,LAMB1,LAMB4,NRCAM,PNPLA8,THAP5,DNAJB9,C7orf66,EIF3IP1,IMMP2L,IMMP2L,LRRN3,DOCK4,DOCK4,DOCK4-AS1,ZNF277,IFRD1,LSMEM1,TMEM168,BMT2,GPR85,SMIM30,PPP1R3A,FOXP2,FOXP2,MIR3666,MDFIC,TFEC,TES,CAV2,CAV1,MET,CAPZA2,ST7-AS1,ST7,ST7,ST7-OT4,ST7,ST7,ST7-AS2,ST7,ST7-OT3,WNT2,ASZ1,CFTR,CFTR,CFTR-AS1,CTTNBP2,LSM8,ANKRD7,KCND2,TSPAN12,ING3,CPED1,WNT16,FAM3C,PTPRZ1,AASS,FEZF1,FEZF1,FEZF1-AS1,CADPS2,CADPS2,RNF133,CADPS2,RNF148,TAS2R16,SLC13A1,IQUB,NDUFA5,ASB15,LOC102724555,ASB15,LMOD2,WASL,HYAL4,SPAM1,TMEM229A,GPR37,C7orf77,POT1,GRM8 | -0.292529 | 258.529 | 1679 | 1008.76 |
| 1 | 19 | 36430374 | 37642956 | LRFN3,SDHAF1,SYNE4,ALKBH6,LOC101927572,CLIP3,LOC101927572,THAP8,THAP8,WDR62,OVOL3,OVOL3,POLR2I,POLR2I,TBCB,CAPNS1,COX7A1,ZNF565,ZNF146,ZFP14,ZFP82,ZNF566,ZNF566,LOC728752,LOC728752,ZNF260,ZNF529,ZNF529,ZNF529-AS1,ZNF382,ZNF461,ZNF567,ZNF850,ZNF790-AS1,ZNF790,ZNF345,ZNF345,ZNF829,ZNF829,ZNF568,ZNF420,ZNF585A | -0.297973 | 239.122 | 312 | 183.721 |
| 1 | 16 | 58742150 | 64981019 | GOT2,LOC729159,CDH8 | -0.298017 | 161.2 | 82 | 50.3834 |

| | | | | | | | | |
|---|---|---|---|---|---|---|---|---|
| 1 | 22 | 45809322 | 46805105 | SMC1B,RIBC2,FBLN1,ATXN10,ATXN10,MIR4762,WNT7B,LOC730668,PRR34,PRR34,PRR34-AS1,MIRLET7BHG,MIR3619,MIRLET7BHG,MIRLET7BHG,MIRLET7A3,MIRLET7BHG,MIR4763,MIRLET7B,PPARA,CDPF1,PKDREJ,TTC38,GTSE1,TRMU,CELSR1 | -0.300177 | 241.058 | 214 | 130.827 |
| 1 | 2 | 75929334 | 84915738 | GCFC2,LRRTM4,REG3G,REG1B,REG1A,REG3A,CTNNA2,CTNNA2,MIR4264,CTNNA2,LRRTM1,FUNDC2P2,SUCLG1,DNAH6 | -0.300226 | 191.985 | 200 | 123.285 |
| 1 | 3 | 1.21E+08 | 121509008 | STXBP5L,POLQ,ARGFX,FBXO40,HCLS1,GOLGB1,IQCB1 | -0.30173 | 269.619 | 154 | 97.9584 |
| 1 | 1 | 2.45E+08 | 245018287 | C1orf100,ADSS,CATSPERE,DESI2,COX20,HNRNPU | -0.30786 | 230.165 | 75 | 41.5947 |
| 1 | 2 | 55920195 | 70142602 | PNPT1,EFEMP1,MIR217HG,MIR217,MIR217HG,MIR216A,MIR217HG,MIR216B,LOC100129434,CCDC85A,CCDC85A,VRK2,VRK2,FANCL,FANCL,MIR4432HG,MIR4432,BCL11A,PAPOLG,REL,PUS10,PUS10,PEX13,PEX13,KIAA1841,C2orf74,AHSA2P,AHSA2P,USP34,USP34,USP34,SNORA70B,XPO1,FAM161A,CCT4,COMMD1,B3GNT2,MIR5192,B3GNT2,TMEM17,EHBP1,EHBP1,LOC100132215,OTX1,WDPCP,MDH1,UGP2,VPS54,PELI1,MIR4433B,MIR4433A,LGALSL,AFTPH,MIR4434,AFTPH,SERTAD2,LINC02245,SLC1A4,CEP68,RAB1A,ACTR2,SPRED2,MIR4778,MEIS1,MEIS1,MEIS1-AS2,ETAA1,C1D,WDR92,WDR92,PNO1,PNO1,PPP3R1,CNRIP1,PLEK,FBXO48,APLF,PROKR1,ARHGAP25,BMP10,GKN2,GKN1,ANTXR1,ANTXR1,MIR3126,GFPT1,NFU1,AAK1,AAK1,SNORA36C,ANXA4,GMCL1,SNRNP27,MXD1 | -0.308352 | 233.938 | 961 | 568.092 |
| 1 | 2 | 1.98E+08 | 204161363 | PGAP1,ANKRD44,SF3B1,COQ10B,HSPD1,HSPE1,HSPE1-MOB4,HSPE1-MOB4,MOB4,RFTN2,MARS2,BOLL,PLCL1,SATB2,FTCDNL1,C2orf69,TYW5,TYW5,MAIP1,MAIP1,SPATS2L,KCTD18,SGO2,AOX1,BZW1,CLK1,PPIL3,NIF3L1,ORC2,FAM126B,NDUFB3,CFLAR,CFLAR,CFLAR-AS1,CASP10,CASP8,ALS2CR12,TRAK2,STRADB,C2CD6,TMEM237,MPP4,ALS2,CDK15,FZD7,KIAA2012,KIAA2012,LOC729224,SUMO1,NOP58,NOP58,SNORD70,NOP58,SNORD70B,NOP58,SNORD11B,NOP58,SNORD11,BMPR2,FAM117B,ICA1L,WDR12,CARF,NBEAL1,CYP20A1 | -0.312285 | 247.322 | 889 | 522.588 |
| 1 | 18 | 18520842 | 30518068 | ROCK1,GREB1L,ESCO1,SNRPD1,ABHD3,ABHD3,MIR320C1,MIB1,MIB1,MIR133A1HG,MIR133A1,MIB1,MIR133A1HG,MIR1-2,GATA6,CTAGE1,RBBP8,MIR4741,RBBP8,CABLES1,TMEM241,RIOK3,RMC1,RMC1,NPC1,NPC1,ANKRD29,LAMA3,TTC39C,TTC39C-AS1,TTC39C,CABYR,OSBPL1A,OSBPL1A,MIR320C2,IMPACT,HRH4,ZNF521,SS18,PSMA8,TAF4B,KCTD1,AQP4,AQP4,AQP4-AS1,AQP4-AS1,CHST9,CHST9,CDH2,MIR302F,DSC3,DSC2,DSC2,DSCAS,DSCAS,DSC1,DSG1,DSG1,DSG1-AS1,DSG1-AS1,DSG4,DSG3,DSG2,DSG2,DSG2-AS1,TTR,B4GALT6,SLC25A52,TRAPPC8,RNF125,RNF138,MEP1B,GAREM1,WBP11P1,KLHL14,CCDC178 | -0.313487 | 244.801 | 927 | 557.472 |

| | | | | | | | | |
|---|---|---|---|---|---|---|---|---|
| 1 | 10 | 71993005 | 72972926 | PPA1,NPFFR1,LRRC20,EIF4EBP2,NODAL,PALD1,PRF1,ADAMTS14,TBATA,SGPL1,PCBD1,UNC5B | -0.314148 | 212.885 | 127 | 78.0069 |
| 1 | 1 | 42334805 | 43887060 | GUCA2B,GUCA2A,FOXJ3,RIMKLA,ZMYND12,CCDC30,PPCS,CCDC30,PPIH,YBX1,CLDN19,P3H1,C1orf50,TMEM269,SVBP,SVBP,ERMAP,ERMAP,ZNF691,SLC2A1,FAM183A,EBNA1BP2,EBNA1BP2,MIR6733,EBNA1BP2,CFAP57,CFAP57,TMEM125,C1orf210,TIE1,MPL,CDC20,ELOVL1,ELOVL1,MIR6734,MED8,SZT2 | -0.315305 | 235.908 | 309 | 190.365 |
| 1 | 19 | 50978627 | 54678163 | FAM71E1,FAM71E1,EMC10,EMC10,JOSD2,ASPDH,LRRC4B,SYT3,C19orf81,SHANK1,CLEC11A,GPR32,ACP4,C19orf48,C19orf48,SNORD88B,C19orf48,SNORD88A,C19orf48,SNORD88C,LINC01869,KLK1,KLK15,KLK15,LOC105372441,KLK3,KLK2,KLKP1,KLK4,KLK5,KLK6,KLK7,KLK8,KLK9,KLK10,KLK11,KLK12,KLK13,KLK14,CTU1,SIGLEC9,SIGLEC7,SIGLEC7,LOC101928517,LOC101928517,SIGLEC17P,CD33,SIGLECL1,IGLON5,VSIG10L,ETFB,CLDND2,NKG7,LIM2,C19orf84,SIGLEC10,SIGLEC10,LOC100129083,SIGLEC8,CEACAM18,SIGLEC12,SIGLEC6,ZNF175,LINC01530,SIGLEC5,SIGLEC14,SPACA6P-AS,SPACA6,MIR99B,MIRLET7E,SPACA6P-AS,SPACA6,MIR125A,SPACA6,HAS1,FPR1,FPR2,FPR3,ZNF577,ZNF649-AS1,ZNF649,ZNF613,ZNF350-AS1,ZNF350,ZNF615,ZNF614,ZNF432,ZNF841,ZNF616,ZNF836,PPP2R1A,ZNF766,ZNF766,MIR643,ZNF480,ZNF610,ZNF880,ZNF528-AS1,ZNF528,ZNF534,ZNF578,ZNF808,ZNF701,ZNF137P,ZNF83,ZNF611,ZNF600,ZNF28,ZNF468,ZNF320,ZNF888,ZNF321P,ZNF816-ZNF321P,ZNF816-ZNF321P,ZNF816,ERVV-1,ERVV-2,ZNF160,ZNF415,ZNF347,ZNF665,ZNF818P,ZNF677,VN1R2,VN1R4,FAM90A27P,BIRC8,ZNF845,ZNF525,ZNF765,ZNF765-ZNF761,ZNF765-ZNF761,ZNF761,ZNF813,ZNF331,LOC284379,DPRX,MIR512-2,MIR512-1,MIR1323,MIR498,MIR520E,MIR515-1,MIR515-2,MIR519E,MIR520F,MIR519C,MIR1283-1,MIR520A,MIR526B,MIR519B,MIR525,MIR523,MIR518F,MIR520B,MIR518B,MIR526A1,MIR520C,MIR518C,MIR524,MIR517A,MIR519D,MIR521-2,MIR520D,MIR517B,MIR520G,MIR516B2,MIR526A2,MIR518E,MIR518A1,MIR518D,MIR516B1,MIR518A2,MIR517C,MIR520H,MIR521- | -0.317023 | 253.477 | 1161 | 677.319 |
| 1 | 2 | 1.87E+08 | 197737351 | ZC3H15,ITGAV,FAM171B,ZSWIM2,CALCRL,TFPI,GULP1,DIRC1,COL3A1,COL3A1,MIR1245A,MIR1245B,COL5A2,COL5A2,MIR3129,WDR75,SLC40A1,ASDURF,ASNSD1,ASNSD1,ANKAR,ANKAR,OSGEPL1,OSGEPL1,ORMDL1,PMS1,MSTN,C2orf88,HIBCH,INPP1,MFSD6,NEMP2,NAB1,GLS,STAT1,LOC105373805,STAT4,STAT4,MYO1B,NABP1,CAVIN2,TMEFF2,SLC39A10,DNAH7,STK17B,HECW2,CCDC150,CCDC150,LOC100130452,GTF3C3,C2orf66,PGAP1 | -0.317183 | 234.753 | 802 | 480.315 |
| 1 | 17 | 7608927 | 7734619 | EFNB3,DNAH2 | -0.319256 | 242.615 | 88 | 56.6083 |

| | | | | | | | | |
|---|---|---|---|---|---|---|---|---|
| 1 | 3 | 1.22E+08 | 136002111 | IQCB1,EAF2,SLC15A2,ILDR1,CD86,CASR,CSTA,CCDC58,FAM162A,WDR5B,LOC102723582,KPNA1,KPNA1,PARP9,PARP9,DTX3L,DTX3L,PARP15,PARP14,HSPBAP1,DIRC2,LINC02035,SEMA5B,PDIA5,PDIA5,MIR7110,SEC22A,ADCY5,HACD2,MYLK-AS1,MYLK,MYLK,CCDC14,ROPN1,KALRN,KALRN,MIR5002,UMPS,UMPS,MIR544B,ITGB5,MUC13,HEG1,SLC12A8,SLC12A8,MIR5092,ZNF148,SNX4,OSBPL11,ALG1L,ROPN1B,SLC41A3,ALDH1L1,ALDH1L1-AS1,ALDH1L1,ALDH1L1,ALDH1L1-AS2,KLF15,CFAP100,ZXDC,UROC1,CHST13,CHST13,C3orf22,C3orf22,TXNRD3NB,TXNRD3NB,TXNRD3,TXNRD3,NUP210P1,CHCHD6,PLXNA1,C3orf56,LINC02016,TPRA1,TPRA1,MIR6825,MCM2,PODXL2,ABTB1,MGLL,KBTBD12,SEC61A1,SEC61A1,RUVBL1,RUVBL1,RUVBL1,EEFSEC,EEFSEC,DNAJB8,GATA2,LINC01565,RPN1,RAB7A,LOC653712,ACAD9,ACAD9,KIAA1257,KIAA1257,KIAA1257,EFCC1,EFCC1,GP9,RAB43,ISY1-RAB43,ISY1-RAB43,ISY1,CNBP,COPG1,COPG1,MIR6826,HMCES,H1FX,RPL32P3,RPL32P3,SNORA7B,EFCAB12,MBD4,IFT122,RHO,H1FOO,PLXND1,TMCC1,TRH,ALG1L2,LINC02014,COL6A5,COL6A6,PIK3R4,ATP2C1,ATP2C1,ASTE1,ASTE1,NEK11,NEK11,LOC339874,NUDT16,MRPL3,MRPL3,SNORA58,CPNE4,CPNE4,MIR5704,ACPP,DNAJC13,ACAD11,NPHP3-ACAD11,ACAD11,NPHP3-ACAD11,ACKR4,ACAD11,NPHP3-ACAD11,UBA5,NPHP3-ACAD11,UBA5,NPHP3-ACAD11,NPHP3,TMEM108,BFSP2,BFSP2,BFSP2-AS1,CDV3,TOPBP1,TF,SRPRB,RAB6B,C3orf36,SLCO2A1,RYK,AMOTL2,AMOTL2,MIR6827,MIR4788,ANAPC13,CEP63,KY,EPHB1,PPP2R3A,MSL2,PCCB | -0.320989 | 232.321 | 1983 | 1199.21 |
| 1 | 14 | 80671895 | 89343758 | DIO2,DIO2,DIO2-AS1,CEP128,TSHR,GTF2A1,GTF2A1,SNORA79,STON2,SEL1L,LINC02301,FLRT2,LINC01148,GALC,GPR65,KCNK10,SPATA7,PTPN21,ZC3H14,EML5,TTC8 | -0.326366 | 231.136 | 365 | 219.747 |
| 1 | 1 | 53537109 | 55541217 | PODN,SLC1A7,CPT2,C1orf123,MAGOH,LRP8,DMRTB1,GLIS1,NDC1,YIPF1,DIO1,HSPB11,LRRC42,LDLRAD1,TMEM59,TCEANC2,MIR4781,TCEANC2,CDCP2,CYB5RL,MRPL37,SSBP3,SSBP3,SSBP3-AS1,ACOT11,ACOT11,FAM151A,MROH7,MROH7-TTC4,MROH7-TTC4,MROH7-TTC4,TTC4,PARS2,TTC22,LEXM,DHCR24,TMEM61,BSND,PCSK9,USP24 | -0.331688 | 232.46 | 370 | 224.109 |
| 1 | 10 | 65928719 | 67828586 | CTNNA3 | -0.33257 | 130.859 | 21 | 11.8057 |
| 1 | 2 | 11598303 | 17997285 | E2F6,GREB1,MIR4429,GREB1,NTSR2,LPIN1,LPIN1,MIR548S,MIR4262,MIR3681HG,MIR3681,TRIB2,TRIB2,MIR3125,FAM84A,NBAS,DDX1,LINC01804,MYCNOS,MYCN,MYCN,FAM49A,RAD51AP2,VSNL1,SMC6,GEN1 | -0.333109 | 232.389 | 322 | 196.335 |

| | | | | | | | | |
|---|---|---|---|---|---|---|---|---|
| 1 | 8 | 82586087 | 94828755 | IMPA1,SLC10A5,ZFAND1,CHMP4C,SNX16,RALYL,LRRCC1,E2F5,E2F5,C8orf59,C8orf59,CA13,CA1,CA3,CA3,CA3-AS1,CA3-AS1,CA2,CA2,PSKH2,ATP6V0D2,SLC7A13,WWP1,RMDN1,CPNE3,CNGB3,CNBD1,DCAF4L2,MMP16,RIPK2,OSGIN2,NBN,DECR1,CALB1,TMEM64,NECAB1,NECAB1,C8orf88,C8orf88,PIP4P2,OTUD6B,LRRC69,LRRC69,MIR4661,LRRC69,SLC26A7,SLC26A7,RUNX1T1,TRIQK,C8orf87,LINC00535,FAM92A,RBM12B,RBM12B,RBM12B-AS1,TMEM67 | -0.33474 | 233.648 | 612 | 349.917 |
| 1 | 13 | 26934340 | 30524633 | CDK8,WASF3,GPR12,USP12,RPL21,RPL21P28,RPL21,RPL21P28,SNORD102,RPL21,RPL21P28,SNORA27,RASL11A,GTF3A,GTF3A,MTIF3,MTIF3,LNX2,POLR1D,GSX1,PDX1,CDX2,URAD,FLT3,PAN3-AS1,PAN3,PAN3,FLT1,POMP,SLC46A3,MTUS2,MTUS2,MTUS2-AS1,SLC7A1,UBL3,LINC00544 | -0.33768 | 226.769 | 297 | 176.133 |
| 1 | 17 | 53484995 | 57808923 | MMD,TMEM100,PCTP,ANKFN1,NOG,C17orf67,DGKE,TRIM25,TRIM25,MIR3614,COIL,SCPEP1,RNF126P1,AKAP1,MSI2,CCDC182,MRPS23,CUEDC1,VEZF1,SRSF1,DYNLL2,OR4D1,MSX2P1,OR4D2,EPX,MKS1,LPO,MPO,TSPOAP1,TSPOAP1,TSPOAP1-AS1,TSPOAP1-AS1,MIR142,TSPOAP1-AS1,TSPOAP1-AS1,MIR4736,TSPOAP1-AS1,SUPT4H1,RNF43,HSF5,MTMR4,SEPT4-AS1,SEPT4,SEPT4-AS1,C17orf47,TEX14,RAD51C,PPM1E,PPM1E,TRIM37,TRIM37,SKA2,SKA2,MIR454,SKA2,MIR301A,PRR11,SMG8,GDPD1,YPEL2,YPEL2,MIR4729,DHX40,CLTC,PTRH2,VMP1 | -0.343101 | 232.533 | 613 | 373.748 |
| 1 | 22 | 16050500 | 20508931 | DUXAP8,BMS1P22,CCT8L2,ANKRD62P1-PARP4P3,XKR3,GAB4,IL17RA,TMEM121B,LINC01664,HDHD5,HDHD5,HDHD5-AS1,ADA2,CECR2,SLC25A18,SLC25A18,LOC101929372,ATP6V1E1,BCL2L13,BID,BID,MIR3198-1,MICAL3,MICAL3,MIR648,PEX26,TUBA8,USP18,GGT3P,DGCR6,PRODH,DGCR2,DGCR2,DGCR11,ESS2,TSSK2,ESS2,GSC2,SLC25A1,CLTCL1,HIRA,MRPL40,C22orf39,UFD1,CDC45,CLDN5,SEPT5,SEPT5,SEPT5-GP1BB,SEPT5-GP1BB,GP1BB,TBX1,GNB1L,GNB1L,RTL10,TXNRD2,TXNRD2,COMT,COMT,COMT,MIR4761,ARVCF,TANGO2,MIR185,TANGO2,DGCR8,MIR3618,DGCR8,MIR1306,DGCR8,TRMT2A,TRMT2A,MIR6816,TRMT2A,RANBP1,RANBP1,RANBP1,SNORA77B,ZDHHC8,CCDC188,LINC00896,RTN4R,RTN4R,MIR1286,DGCR6L,LOC101927859,TMEM191B,LOC101927859,PI4KAP1 | -0.343204 | 227.337 | 689 | 404.093 |
| 1 | 10 | 88197228 | 88811098 | WAPL,OPN4,LDB3,BMPR1A,MMRN2,MMRN2,SNCG,SNCG,ADIRF,ADIRF,AGAP11,AGAP11 | -0.348846 | 246.881 | 105 | 64.5705 |
| 1 | 10 | 32096541 | 32740075 | ARHGAP12,KIF5B,EPC1 | -0.352945 | 253.93 | 65 | 40.7505 |
| 1 | 17 | 19437237 | 22220573 | SLC47A1,SLC47A1,SNORA59B,SNORA59A,ALDH3A2,SLC47A2,ALDH3A1,ULK2,AKAP10,SPECC1,CCDC144CP,LGALS9B,CDRT15L2,LOC100287072,CCDC144NL,CCDC144NL,CCDC144NL-AS1,USP22,DHRS7B,TMEM11,NATD1,MAP2K3,KCNJ12,KCNJ18,C17orf51,UBBP4,FAM27E5 | -0.356039 | 232.504 | 289 | 162.744 |
| 1 | X | 1.06E+08 | 106114056 | CXorf57,RNF128,TBC1D8B | -0.356363 | 260.965 | 53 | 31.601 |

| | | | | | | | | |
|---|---|---|---|---|---|---|---|---|
| 1 | 11 | 1.03E+08 | 111152234 | DYNC2H1,MIR4693,PDGFD,PDGFD,DDI1,CASP12,CASP4,CASP5,CASP1,CARD16,CARD17,CARD18,GRIA4,MSANTD4,KBTBD3,KBTBD3,AASDHPPT,AASDHPPT,GUCY1A2,CWF19L2,ALKBH8,ELMOD1,SLN,SLC35F2,RAB39A,CUL5,ACAT1,NPAT,ATM,ATM,C11orf65,C11orf65,KDELC2,EXPH5,DDX10,C11orf87,ZC3H12C,RDX,FDX1,ARHGAP20,C11orf53 | -0.358864 | 238.835 | 577 | 338.223 |
| 1 | 14 | 53251943 | 64911533 | FERMT2,DDHD1,MIR5580,BMP4,CDKN3,CNIH1,GMFB,CGRRF1,SAMD4A,GCH1,GCH1,MIR4308,WDHD1,SOCS4,MAPK1IP1L,LGALS3,DLGAP5,FBXO34,ATG14,TBPL2,KTN1-AS1,KTN1,PELI2,TMEM260,OTX2,EXOC5,AP5M1,NAA30,CCDC198,SLC35F4,C14orf37,ACTR10,PSMA3,PSMA3,PSMA3-AS1,PSMA3-AS1,ARID4A,TOMM20L,TOMM20L,TIMM9,TIMM9,TIMM9,KIAA0586,KIAA0586,DACT1,DAAM1,GPR135,L3HYPDH,JKAMP,JKAMP,CCDC175,CCDC175,RTN1,RTN1,MIR5586,LRRC9,PCNX4,DHRS7,PPM1A,C14orf39,SIX6,SIX1,SIX4,MNAT1,TRMT5,TRMT5,SLC38A6,SLC38A6,TMEM30B,PRKCH,LINC01303,FLJ22447,FLJ22447,HIF1A-AS1,HIF1A,HIF1A,HIF1A,LOC105370526,HIF1A,LOC105370526,HIF1A-AS2,SNAPC1,SYT16,LINC00643,KCNH5,RHOJ,GPHB5,PPP2R5E,WDR89,SGPP1,SYNE2,SYNE2,MIR548H1,ESR2,TEX21P,MTHFD1 | -0.359282 | 239.531 | 1154 | 690.527 |
| 1 | 18 | 66382152 | 72104007 | TMX3,CCDC102B,DOK6,CD226,RTTN,SOCS6,CBLN2,NETO1,NETO1,MIR548AV,FBXO15,TIMM21,CYB5A,C18orf63,FAM69C | -0.36073 | 204.19 | 202 | 120.067 |

| | | | | | | | | |
|---|---|---|---|---|---|---|---|---|
| 1 | 10 | 98369486 | 105727620 | PIK3AP1,MIR607,LCOR,SLIT1,SLIT1,ARHGAP19-SLIT1,ARHGAP19-SLIT1,ARHGAP19,FRAT1,FRAT2,RRP12,PGAM1,EXOSC1,ZDHHC16,MMS19,UBTD1,ANKRD2,HOGA1,HOGA1,C10orf62,MORN4,PI4K2A,AVPI1,MARVELD1,ZFYVE27,SFRP5,GOLGA7B,GOLGA7B,CRTAC1,CRTAC1,R3HCC1L,LOXL4,PYROXD2,PYROXD2,MIR1287,HPS1,HPS1,MIR4685,HPSE2,CNNM1,GOT1,NKX2-3,SLC25A28,ENTPD7,ENTPD7,COX15,COX15,COX15,CUTC,CUTC,ABCC2,DNMBP,DNMBP,DNMBP-AS1,CPN1,ERLIN1,CHUK,CWF19L1,CWF19L1,SNORA12,BLOC1S2,PKD2L1,SCD,WNT8B,SEC31B,NDUFB8,HIF1AN,PAX2,SLF2,SEMA4G,SEMA4G,MIR608,SEMA4G,MRPL43,MRPL43,TWNK,LZTS2,LZTS2,PDZD7,PDZD7,SFXN3,KAZALD1,TLX1NB,TLX1NB,TLX1,LBX1,LBX1-AS1,BTRC,POLL,POLL,DPCD,DPCD,DPCD,MIR3158-1,MIR3158-2,FBXW4,FGF8,NPM3,MGEA5,KCNIP2-AS1,KCNIP2,KCNIP2,C10orf76,HPS6,LDB1,PPRC1,NOLC1,ELOVL3,PITX3,GBF1,NFKB2,PSD,FBXL15,CUEDC2,MIR146B,RPARP-AS1,C10orf95,RPARP-AS1,MFSD13A,ACTR1A,SUFU,TRIM8,ARL3,SFXN2,WBP1L,CYP17A1,BORCS7,BORCS7-ASMT,BORCS7-ASMT,AS3MT,CNNM2,NT5C2,RPEL1,INA,PCGF6,TAF5,ATP5MD,ATP5MD,MIR1307,PDCD11,CALHM2,CALHM1,CALHM3,NEURL1-AS1,NEURL1,NEURL1,SH3PXD2A,STN1,SLK | -0.362205 | 224.286 | 1465 | 901.167 |
| 1 | 10 | 90562617 | 91465271 | LIPM,LIPM,ANKRD22,ANKRD22,STAMBPL1,ACTA2-AS1,ACTA2,ACTA2,ACTA2,FAS,FAS,CH25H,LIPA,IFIT2,IFIT3,IFIT1B,IFIT1,IFIT5,SLC16A12,SLC16A12,SLC16A12-AS1,PANK1,PANK1,MIR107,KIF20B | -0.363155 | 232.145 | 141 | 88.0836 |
| 1 | 3 | 1.83E+08 | 183862028 | DCUN1D1,MCCC1,LAMP3,MCF2L2,MCF2L2,B3GNT5,KLHL6,KLHL24,YEATS2,YEATS2,YEATS2-AS1,MAP6D1,PARL,ABCC5,ABCC5,ABCC5-AS1,HTR3D,HTR3C,HTR3E-AS1,HTR3E,HTR3E,EIF2B5 | -0.365331 | 230.208 | 251 | 147.342 |
| 1 | 10 | 73269775 | 74774128 | CDH23,CDH23-AS1,CDH23,CDH23,C10orf105,CDH23,VSIR,PSAP,CHST3,SPOCK2,ASCC1,ASCC1,ANAPC16,ANAPC16,DDIT4,DNAJB12,MICU1,MCU,MCU,MIR4676,OIT3,PLA2G12B,P4HA1 | -0.366904 | 243.74 | 245 | 146.51 |

| | | | | | | | | |
|---|---|---|---|---|---|---|---|---|
| 1 | 6 | 70071890 | 88300020 | ADGRB3,LMBRD1,COL19A1,COL9A1,FAM135A,SDHAF4,SMAP1,SMAP1,B3GAT2,B3GAT2,OGFRL1,MIR30C2,MIR30A,RIMS1,KCNQ5,KCNQ5,MIR4282,KHDC1L,KHDC1,DPPA5,KHDC3L,OOEP,DDX43,CGAS,MTO1,EEF1A1,EEF1A1,SNORD141A,SNORD141B,SLC17A5,LOC101928489,CD109,CD109,COL12A1,COX7A2,TMEM30A,FILIP1,FILIP1,LOC101928540,FILIP1,LOC101928540,MIR4463,SENP6,MYO6,IMPG1,HTR1B,IRAK1BP1,PHIP,HMGN3,HMGN3,HMGN3-AS1,LCA5,SH3BGRL2,LINC01621,ELOVL4,TTK,BCKDHB,FAM46A,IBTK,TPBG,UBE3D,DOPEY1,DOPEY1,PGM3,PGM3,RWDD2A,ME1,PRSS35,SNAP91,RIPPLY2,CYB5R4,MRAP2,CEP162,TBX18,NT5E,SNX14,SYNCRIP,SNHG5,SNORD50A,SNHG5,SNORD50B,HTR1E,CGA,ZNF292,GJB7,SMIM8,C6orf163,LINC01590,CFAP206,SLC35A1,RARS2,ORC3 | -0.368049 | 228.533 | 1287 | 757.922 |
| 1 | 2 | 21259954 | 24896409 | APOB,TDRD15,KLHL29,ATAD2B,UBXN2A,MFSD2B,WDCP,FKBP1B,SF3B6,FAM228B,TP53I3,FAM228B,PFN4,FAM228B,FAM228A,ITSN2,NCOA1 | -0.382579 | 218.506 | 237 | 146.509 |
| 1 | 7 | 61657308 | 65139352 | ZNF733P,LOC102724738,LOC100287704,LOC100287834,LOC100287834,MIR4283-1,MIR4283-2,ZNF727,ZNF735,ZNF679,ZNF736,ZNF680,ZNF107,ZNF138,ZNF273,ZNF117,ERV3-1-ZNF117,ERV3-1-ZNF117,ERV3-1,CCT6P3,SNORA22C,CCT6P3,INTS4P1,ZNF92,LOC441242,INTS4P2 | -0.382696 | 192.678 | 177 | 81.9529 |
| 1 | 3 | 1.95E+08 | 197961930 | PPP1R2,APOD,LOC105374297,SDHAP2,SDHAP2,MIR570HG,MIR570HG,MIR570,MUC20,MUC4,TNK2,TNK2-AS1,SDHAP1,TFRC,ZDHHC19,SLC51A,PCYT1A,TCTEX1D2,TCTEX1D2,TM4SF19-TCTEX1D2,TM4SF19-TCTEX1D2,TM4SF19-AS1,TM4SF19,TM4SF19-TCTEX1D2,TM4SF19,UBXN7,UBXN7,UBXN7-AS1,RNF168,SMCO1,WDR53,FBXO45,NRROS,CEP19,PIGX,PAK2,SENP5,NCBP2,NCBP2,NCBP2-AS1,NCBP2-AS2,PIGZ,MELTF,MELTF-AS1,MELTF,DLG1,DLG1,MIR4797,BDH1,RUBCN,MIR922,RUBCN,RUBCN,FYTTD1,FYTTD1,LRCH3,IQCG,IQCG,RPL35A,LMLN,LMLN,LMLN-AS1 | -0.385507 | 237.957 | 472 | 278.879 |
| 1 | 11 | 85447550 | 92085773 | SYTL2,CCDC83,PICALM,EED,EED,MIR6755,HIKESHI,CCDC81,ME3,PRSS23,PRSS23,FZD4,FZD4,TMEM135,RAB38,MIR3166,CTSC,GRM5-AS1,GRM5,GRM5,TYR,NOX4,FOLH1B,TRIM77,TRIM49,TRIM64B,TRIM64,TRIM49C,UBTFL1,NAALAD2,CHORDC1,DISC1FP1,MIR4490,DISC1FP1,MIR1261,FAT3 | -0.38765 | 225.103 | 367 | 204.047 |

| | | | | | | | | |
|---|---|---|---|---|---|---|---|---|
| 1 | 14 | 26918692 | 53101810 | NOVA1,MIR4307HG,MIR4307HG,MIR4307,FOXG1,LINC01551,PRKD1,G2E3-AS1,G2E3,G2E3,SCFD1,COCH,COCH,LOC100506071,STRN3,STRN3,MIR624,STRN3,AP4S1,AP4S1,HECTD1,HEATR5A,LOC101927124,LOC101927124,DTD2,DTD2,GPR33,NUBPL,LINC02313,ARHGAP5,AKAP6,NPAS3,EGLN3,SPTSSA,EAPP,SNX6,CFL2,BAZ1A,SRP54,FAM177A1,FAM177A1,LOC101927178,LOC101927178,PPP2R3C,PPP2R3C,KIAA0391,PSMA6,NFKBIA,INSM2,RALGAPA1,RALGAPA1P1,RALGAPA1,RALGAPA1P1,SNORA101B,BRMS1L,MBIP,SFTA3,SFTA3,NKX2-1,SFTA3,NKX2-1,NKX2-1-AS1,NKX2-1,NKX2-1-AS1,NKX2-8,PAX9,SLC25A21,SLC25A21,SLC25A21-AS1,MIPOL1,FOXA1,TTC6,SSTR1,CLEC14A,SEC23A,GEMIN2,TRAPPC6B,PNN,MIA2,FBXO33,LRFN5,FSCB,C14orf28,LOC101927418,KLHL28,TOGARAM1,PRPF39,PRPF39,SNORD127,FKBP3,FANCM,MIS18BP1,RPL10L,MDGA2,MIR548Y,RPS29,RPS29,LRR1,LRR1,RPL36AL,MGAT2,DNAAF2,POLE2,KLHDC1,KLHDC2,NEMF,ARF6,LINC01588,LINC01588,LINC01599,LINC01599,VCPKMT,SOS2,L2HGDH,L2HGDH,MIR4504,ATP5S,CDKL1,MAP4K5,ATL1,SAV1,NIN,NIN,LOC105370489,ABHD12B,PYGL,TRIM9,TMX1,FRMD6,GNG2,GNG2,LOC102723604,RTRAF,NID2,PTGDR,PTGER2,TXNDC16,GPR137C | -0.38869 | 229.237 | 1708 | 1005.55 |
| 1 | 12 | 1.23E+08 | 123110903 | ZCCHC8,RSRC2,KNTC1 | -0.391992 | 285.972 | 88 | 52.5926 |
| 1 | 2 | 1.8E+08 | 179572573 | MIR548N,TTN,TTN | -0.40038 | 273.552 | 96 | 59.1276 |
| 1 | 12 | 50384090 | 50410433 | RACGAP1 | -0.402372 | 232.639 | 19 | 10.3678 |
| 1 | 7 | 82792370 | 94252789 | SEMA3E,SEMA3A,SEMA3D,GRM3,KIAA1324L,LOC101927420,DMTF1,DMTF1,TMEM243,CROT,ABCB4,ABCB1,ABCB1,RUNDC3B,RUNDC3B,SLC25A40,DBF4,ADAM22,SRI,SRI,LOC102723885,STEAP4,ZNF804B,ZNF804B,TEX47,STEAP2-AS1,STEAP2-AS1,STEAP1,STEAP2,CFAP69,LOC101927446,GTPBP10,CLDN12,CDK14,FZD1,MTERF1,AKAP9,CYP51A1,CYP51A1-AS1,CYP51A1-AS1,LRRD1,KRIT1,ANKIB1,GATAD1,PEX1,RBM48,FAM133B,FAM133DP,CDK6,SAMD9,SAMD9L,HEPACAM2,VPS50,CALCR,CALCR,MIR653,CALCR,MIR489,MIR4652,TFPI2,GNGT1,GNG11,BET1,COL1A2,CASD1,SGCE | -0.411124 | 240.148 | 939 | 561.032 |
| 1 | 17 | 5042917 | 6908768 | USP6,ZNF594,LOC100130950,SCIMP,RABEP1,RABEP1,NUP88,NUP88,RPAIN,C1QBP,DHX33,DERL2,MIS12,LOC728392,NLRP1,WSCD1,AIPL1,PIMREG,PITPNM3,KIAA0753,TXNDC17,TXNDC17,MED31,MED31,C17orf100,MIR4520-1,MIR4520-2,ALOX15P1,SLC13A5,XAF1,FBXO39,TEKT1,ALOX12-AS1,ALOX12-AS1,ALOX12 | -0.41133 | 214.488 | 296 | 177.593 |
| 1 | 12 | 1.19E+08 | 118839696 | TAOK3,SUDS3 | -0.412056 | 227.035 | 27 | 15.0822 |
| 1 | 13 | 21148483 | 21949354 | IFT88,IL17D,EEF1AKMT1,XPO4,LATS2,SAP18,SKA3,SKA3,MRPL57,MRPL57,MIPEPP3,ZDHHC20 | -0.412449 | 220.809 | 98 | 59.3043 |

| | | | | | | | | |
|---|---|---|---|---|---|---|---|---|
| 1 | 3 | 1.14E+08 | 120833279 | ZBTB20,MIR4796,ZBTB20,GAP43,LSAMP,MIR4447,LOC105374060,IGSF11,C3orf30,UPK1B,B4GALT4,B4GALT4,B4GALT4-AS1,ARHGAP31,TMEM39A,POGLUT1,TIMMDC1,CD80,ADPRH,PLA1A,POPDC2,COX17,MAATS1,NR1I2,GSK3B,GPR156,LRRC58,FSTL1,MIR198,FSTL1,NDUFB4,HGD,RABL3,GTF2E1,STXBP5L,STXBP5L,MIR5682 | -0.412749 | 193.434 | 336 | 203.755 |
| 1 | 10 | 70928318 | 71914905 | VPS26A,SUPV3L1,LOC101928994,HKDC1,HKDC1,HK1,TACR2,TSPAN15,NEUROG3,FAM241B,COL13A1,H2AFY2,AIFM2,TYSND1,SAR1A | -0.412935 | 205.182 | 180 | 108.821 |
| 1 | 1 | 1.85E+08 | 185259257 | RNF2,TRMT1L,SWT1 | -0.415891 | 267.712 | 41 | 23.3926 |
| 1 | 13 | 22097333 | 26107588 | MICU2,FGF9,SGCG,SACS,TNFRSF19,MIPEP,MIPEP,PCOTH,PCOTH,C1QTNF9B,C1QTNF9B,SPATA13,MIR2276,SPATA13,C1QTNF9,PARP4,TPTE2P6,ATP12A,RNF17,CENPJ,TPTE2P1,PABPC3,AMER2,MTMR6,NUP58,ATP8A2 | -0.416515 | 232.754 | 395 | 237.462 |
| 1 | 3 | 1.85E+08 | 194990255 | VPS8,C3orf70,EHHADH-AS1,EHHADH,EHHADH,MIR5588,MAP3K13,TMEM41A,LIPH,SENP2,IGF2BP2,IGF2BP2,IGF2BP2-AS1,TRA2B,ETV5,DGKG,CRYGS,TBCCD1,DNAJB11,AHSG,FETUB,HRG,KNG1,EIF4A2,EIF4A2,SNORD2,EIF4A2,MIR1248,SNORA81,EIF4A2,SNORA63,EIF4A2,SNORA4,RFC4,ADIPOQ,ADIPOQ-AS1,ST6GAL1,RPL39L,LOC101929106,RTP1,MASP1,MASP1,LOC101929130,RTP4,SST,RTP2,RTP2,LOC100131635,LOC100131635,LOC100131635,BCL6,BCL6,LPP,FLJ42393,LPP,LPP,MIR28,TPRG1,TPRG1,TPRG1-AS2,TP63,TP63,MIR944,P3H2,CLDN1,CLDN16,TMEM207,IL1RAP,GMNC,OSTN,OSTN,OSTN-AS1,UTS2B,UTS2B,CCDC50,CCDC50,LINCR-0002,PYDC2,FGF12,FGF12,FGF12-AS1,MB21D2,HRASLS,ATP13A5,ATP13A5,ATP13A5-AS1,ATP13A4,ATP13A4,ATP13A4-AS1,OPA1,OPA1,OPA1-AS1,HES1,CPN2,LRRC15,GP5,ATP13A3,TMEM44-AS1,TMEM44,TMEM44,LSG1,FAM43A,XXYLT1,XXYLT1,MIR3137 | -0.417742 | 201.29 | 849 | 508.817 |
| 1 | 10 | 81521061 | 82191781 | BMS1P21,MBL1P,SFTPD,TMEM254-AS1,TMEM254,TMEM254,PLAC9,ANXA11,LOC100130698,MAT1A,DYDC1,DYDC1,DYDC2,DYDC2,FAM213A | -0.418366 | 230.399 | 91 | 51.515 |
| 1 | 11 | 82968195 | 85373560 | CCDC90B,DLG2,TMEM126B,TMEM126A,CREBZF | -0.421226 | 183.946 | 99 | 56.0198 |
| 1 | 12 | 37857194 | 44196330 | ALG10B,CPNE8,KIF21A,ABCD2,C12orf40,C12orf40,SLC2A13,SLC2A13,LRRK2,MUC19,CNTN1,PDZRN4,GXYLT1,YAF2,ZCRB1,PPHLN1,PRICKLE1,ADAMTS20,PUS7L,IRAK4,TWF1 | -0.422367 | 237.081 | 545 | 321.889 |

| | | | | | | | | |
|---|---|---|---|---|---|---|---|---|
| 1 | 1 | 94645278 | 109351516 | ARHGAP29,ABCD3,F3,SLC44A3-AS1,MIR378G,SLC44A3,CNN3,LOC729970,ALG14,ALG14,LOC101928098,TMEM56,TMEM56-RWDD3,TMEM56,TMEM56-RWDD3,LOC101928118,TMEM56-RWDD3,LOC101928118,TMEM56-RWDD3,RWDD3,RWDD3,PTBP2,DPYD,DPYD,DPYD-AS1,MIR137HG,MIR137HG,MIR2682,MIR137HG,MIR137,SNX7,PLPPR5,PLPPR5,LOC100129620,PLPPR4,PALMD,PALMD,MIR548AA1,MIR548AA1,FRRS1,FRRS1,AGL,SLC35A3,MFSD14A,SASS6,TRMT13,TRMT13,LRRC39,LRRC39,DBT,RTCA,RTCA,MIR553,CDC14A,GPR88,VCAM1,EXTL2,SLC30A7,DPH5,S1PR1,OLFM3,COL11A1,RNPC3,AMY2B,AMY2A,PRMT6,NTNG1,VAV3,VAV3,VAV3-AS1,SLC25A24,NBPF4,NBPF6,FAM102B,HENMT1,PRPF38B,FNDC7,STXBP3 | -0.424362 | 228.737 | 777 | 451.883 |
| 1 | 19 | 608122 | 652397 | HCN2,POLRMT,FGF22,RNF126 | -0.437292 | 188.155 | 47 | 24.5774 |
| 1 | 17 | 9153881 | 17067581 | STX8,CFAP52,USP43,DHRS7C,GSG1L2,GLP2R,RCVRN,GAS7,MYH13,MYHAS,MYH8,MYHAS,MYH4,MYHAS,MYH1,MYHAS,MYH2,MYH3,SCO1,ADPRM,TMEM220,TMEM220,TMEM220-AS1,PIRT,SHISA6,DNAH9,ZNF18,MAP2K4,MAP2K4,MIR744,MYOCD,MYOCD,LOC101928418,MYOCD,LOC100128006,LOC100128006,ARHGAP44,ARHGAP44,ARHGAP44,MIR1269B,ELAC2,HS3ST3A1,CDRT15P1,COX10,CDRT15,HS3ST3B1,PMP22,PMP22,MIR4731,TEKT3,CDRT4,TVP23C-CDRT4,TVP23C-CDRT4,TVP23C,CDRT1,TRIM16,ZNF286A,TBC1D26,CDRT15P2,MEIS3P1,ADORA2B,ZSWIM7,ZSWIM7,TTC19,TTC19,NCOR1,PIGL,PIGL,MIR1288,CENPV,UBB,TRPV2,LRRC75A-AS1,LRRC75A-AS1,SNORD49B,LRRC75A-AS1,SNORD49A,LRRC75A-AS1,SNORD65,LRRC75A-AS1,LRRC75A,LRRC75A,ZNF287,ZNF624,CCDC144A,KRT16P2,TBC1D27P,TNFRSF13B,LINC02090,MPRIP | -0.437764 | 225.209 | 1003 | 588.17 |
| 1 | 12 | 4611535 | 4665738 | C12orf4,RAD51AP1 | -0.438124 | 236.358 | 23 | 13.1681 |
| 1 | 10 | 75561474 | 76074567 | ZSWIM8,NDST2,CAMK2G,C10orf55,PLAU,VCL,AP3M1,ADK | -0.438246 | 197.863 | 104 | 64.0208 |
| 1 | 10 | 82558150 | 86131216 | NRG3,NRG3,NRG3-AS1,GHITM,C10orf99,CDHR1,LRIT2,LRIT1,RGR,CCSER2 | -0.439543 | 197.075 | 105 | 65.8428 |
| 1 | 19 | 19869774 | 24364012 | ZNF56,ZNF506,ZNF56,ZNF253,ZNF93,ZNF682,ZNF90,ZNF486,MIR1270,ZNF826P,MIR1270,ZNF737,ZNF626,ZNF66,ZNF85,ZNF430,ZNF714,ZNF431,ZNF708,ZNF738,ZNF493,ZNF429,LOC400682,ZNF100,ZNF100,LOC641367,ZNF43,ZNF208,ZNF257,ZNF676,ZNF729,ZNF98,GOLGA2P9,ZNF492,ZNF99,ZNF728,ZNF730,ZNF724,IPO5P1,ZNF91,ZNF675,ZNF681,ZNF726,ZNF254,HAVCR1P1 | -0.444401 | 230.174 | 456 | 206.759 |
| 1 | 8 | 15531891 | 17143853 | TUSC3,MSR1,FGF20,MICU3,ZDHHC2,CNOT7,VPS37A | -0.447648 | 220.175 | 97 | 53.6697 |

| | | | | | | | | |
|---|---|---|---|---|---|---|---|---|
| 1 | 17 | 30302522 | 36397655 | SUZ12,LRRC37B,LRRC37B,SH3GL1P1,LOC105371730,LOC105371730,RHOT1,RHOT1,RHBDL3,C17orf75,MIR632,ZNF207,ZNF207,PSMD11,CDK5R1,MYO1D,TMEM98,SPACA3,ASIC2,CCL2,CCL7,CCL11,CCL8,CCL13,CCL1,C17orf102,TMEM132E,CCT6B,ZNF830,LIG3,RFFL,RAD51L3-RFFL,RAD51L3-RFFL,RAD51D,RAD51L3-RFFL,FNDC8,NLE1,UNC45B,SLC35G3,SLFN5,SLFN11,SLFN12,SLFN13,SLFN12L,SLFN14,SLFN14,LOC107985033,LINC02001,SNORD7,PEX12,AP2B1,RASL10B,GAS2L2,MMP28,C17orf50,MMP28,TAF15,HEATR9,CCL5,RDM1,LYZL6,CCL16,CCL14,CCL15-CCL14,CCL15-CCL14,CCL15,CCL23,CCL18,CCL3,CCL4,CCL3L3,CCL3L1,CCL4L1,CCL4L2,ZNHIT3,ZNHIT3,MYO19,MYO19,PIGW,GGNBP2,DHRS11,MRM1,LHX1,AATF,AATF,MIR2909,ACACA,ACACA,SNORA90,ACACA,C17orf78,TADA2A,DUSP14,SYNRG,DDX52,HNF1B,YWHAEP7,LOC440434 | -0.447867 | 230.913 | 867 | 509.528 |
| 1 | 10 | 1.25E+08 | 127264837 | IKZF5,ACADSB,HMX3,HMX2,BUB3,GPR26,CPXM2,CHST15,OAT,NKX1-2,LHPP,FAM53B,FAM53B,FAM53B-AS1,EEF1AKMT2,ABRAXAS2,ZRANB1,CTBP2,CTBP2,MIR4296 | -0.448025 | 192.237 | 193 | 112.234 |
| 1 | 17 | 29226160 | 30205737 | TEFM,ADAP2,RNF135,LOC646030,MIR4733,NF1,NF1,OMG,NF1,EVI2B,NF1,EVI2A,RAB11FIP4,RAB11FIP4,MIR4724,MIR193A,MIR4725,MIR365B,COPRS,UTP6 | -0.453562 | 230.525 | 170 | 98.9495 |
| 1 | 3 | 1.36E+08 | 141205443 | PCCB,STAG1,SLC35G2,NCK1-AS1,NCK1,IL20RB,SOX14,CLDN18,DZIP1L,A4GNT,DBR1,ARMC8,ARMC8,NME9,NME9,MRAS,ESYT3,CEP70,FAIM,PIK3CB,FOXL2,FOXL2NB,PRR23A,PRR23B,PRR23C,MRPS22,COPB2,LOC100507291,RBP2,LOC100507291,RBP1,LOC100507291,NMNAT3,NMNAT3,CLSTN2,TRIM42,SLC25A36,SPSB4,PXYLP1,ZBTB38 | -0.457156 | 207.661 | 451 | 265.36 |
| 1 | 4 | 1.68E+08 | 169393964 | ANXA10,DDX60,DDX60L | -0.464918 | 218.791 | 106 | 61.2855 |
| 1 | 7 | 76871662 | 82532073 | CCDC146,GSAP,GSAP,LOC101927243,PTPN12,APTR,RSBN1L,RSBN1L,TMEM60,PHTF2,MAGI2,MAGI2,MAGI2-AS3,GNAI1,GNAT3,CD36,SEMA3C,HGF,CACNA2D1,CACNA2D1,LOC101927356,PCLO | -0.47013 | 209.598 | 335 | 195.376 |
| 1 | 16 | 11858838 | 11991904 | ZC3H7A,BCAR4,RSL1D1,GSPT1 | -0.472935 | 235.479 | 47 | 27.8168 |
| 1 | 9 | 99296129 | 99522759 | CDC14B,AAED1,ZNF510 | -0.482728 | 254.927 | 29 | 15.6891 |
| 1 | 10 | 1.18E+08 | 122809857 | C10orf82,HSPA12A,HSPA12A,ENO4,ENO4,SHTN1,VAX1,MIR3663HG,MIR3663,KCNK18,SLC18A2,PDZD8,EMX2OS,EMX2,EMX2,RAB11FIP2,FAM204A,PRLHR,CACUL1,NANOS1,EIF3A,EIF3A,SNORA19,FAM45A,FAM45BP,SFXN4,PRDX3,GRK5,GRK5,MIR4681,RGS10,TIAL1,BAG3,INPP5F,MCMBP,SEC23IP,MIR4682,PLPP4,LINC01561,WDR11 | -0.485255 | 221.415 | 400 | 242.93 |

| | | | | | | | | |
|---|---|---|---|---|---|---|---|---|
| 1 | 20 | 13765762 | 21141969 | NDUFAF5,SEL1L2,MACROD2,MACROD2,FLRT3,MACROD2,LOC613266,KIF16B,SNRPB2,OTOR,PCSK2,BFSP1,DSTN,RRBP1,BANF2,SNX5,SNX5,SNORD17,MGME1,OVOL2,PET117,PET117,KAT14,KAT14,ZNF133,LINC00851,DZANK1,POLR3F,POLR3F,MIR3192,RBBP9,SEC23B,SMIM26,DTD1,C20orf78,C20orf78,SCP2D1,SLC24A3,SLC24A3,LOC100130264,RIN2,NAA20,CRNKL1,CRNKL1,CFAP61,CFAP61,INSM1,RALGAPA2,KIZ | -0.485795 | 208.811 | 563 | 332.257 |
| 1 | 14 | 96755980 | 97347571 | ATG2B,GSKIP,AK7,PAPOLA,VRK1 | -0.490212 | 221.842 | 109 | 64.6789 |
| 1 | 20 | 55906878 | 55918510 | SPO11 | -0.490755 | 223.752 | 13 | 7.44615 |
| 1 | 18 | 10500 | 2796088 | ROCK1P1,USP14,THOC1,COLEC12,CETN1,CLUL1,CLUL1,TYMSOS,TYMSOS,TYMS,TYMS,TYMS,ENOSF1,ENOSF1,YES1,ADCYAP1,LINC00470,METTL4,NDC80,SMCHD1 | -0.494072 | 234.263 | 219 | 128.142 |
| 1 | X | 72783963 | 73045726 | CHIC1,TSIX,XIST | -0.49971 | 221.894 | 29 | 18.8923 |
| 1 | 21 | 40578007 | 40652227 | BRWD1 | -0.499924 | 260.01 | 28 | 17.1484 |
| 1 | 17 | 38573987 | 38976434 | TOP2A,IGFBP4,TNS4,CCR7,SMARCE1,KRT222,KRT24,KRT25,KRT26,KRT27,KRT28,KRT10,KRT10,TMEM99 | -0.504533 | 231.431 | 112 | 66.6825 |
| 1 | 3 | 1.12E+08 | 113866381 | CD200,BTLA,ATG3,ATG3,SLC35A5,SLC35A5,CCDC80,LOC100129297,CD200R1L,CD200R1L,CD200R1,GTPBP8,NEPRO,BOC,BOC,CFAP44,CFAP44,CFAP44,CFAP44-AS1,SPICE1,SIDT1,SIDT1,MIR4446,USF3,NAA50,ATP6V1A,GRAMD1C,ZDHHC23,CCDC191,QTRT2,DRD3 | -0.510543 | 226.876 | 343 | 210.584 |
| 1 | 16 | 1887762 | 1918190 | FAHD1,MEIOB,MEIOB | -0.51178 | 228.904 | 12 | 7.57558 |
| 1 | 13 | 35785824 | 41655018 | NBEA,NBEA,MAB21L1,DCLK1,SOHLH2,CCDC169-SOHLH2,CCDC169-SOHLH2,CCDC169,SPART,SPART,SPART-AS1,CCNA1,SERTM1,RFXAP,SMAD9,ALG5,EXOSC8,SUPT20H,CSNK1A1L,POSTN,TRPC4,UFM1,FREM2,STOML3,PROSER1,NHLRC3,LHFPL6,COG6,COG6,MIR4305,FOXO1,MIR320D1,MRPS31,SLC25A15,SLC25A15,TPTE2P5,SLC25A15,TPTE2P5,MIR621,TPTE2P5,TPTE2P5,SUGT1P3,ELF1,WBP4 | -0.515072 | 203.3 | 452 | 267.565 |
| 1 | 3 | 1.08E+08 | 111870774 | DZIP3,RETNLB,TRAT1,GUCA1C,MORC1,MORC1,MORC1-AS1,C3orf85,DPPA2,DPPA4,LINC01205,NECTIN3,CD96,CD96,ZBED2,PLCXD2,PLCXD2,PHLDB2,PHLDB2,ABHD10,TAGLN3,TMPRSS7,C3orf52,C3orf52,MIR567,GCSAM,SLC9C1 | -0.516958 | 201.149 | 261 | 156.875 |
| 1 | 20 | 6760256 | 13568005 | HAO1,TMX4,PLCB1,PLCB4,LAMP5-AS1,LAMP5,LAMP5,PAK5,SNAP25-AS1,ANKEF1,SNAP25,MKKS,SLX4IP,JAG1,JAG1,MIR6870,C20orf187,LINC00687,BTBD3,SPTLC3,ISM1,TASP1 | -0.519697 | 195.559 | 301 | 183.124 |
| 1 | 2 | 1.8E+08 | 187365066 | TTN,LOC101927055,TTN,CCDC141,SESTD1,ZNF385B,ZNF385B,MIR1258,CWC22,UBE2E3,LINC01934,MIR4437,ITGA4,ITGA4,CERKL,CERKL,NEUROD1,SSFA2,PPP1R1C,PDE1A,DNAJC10,FRZB,NCKAP1,DUSP19,NUP35,MIR548AE1,ZNF804A,LOC101927196,FSIP2,FSIP2,FSIP2,FSIP2-AS1,ZC3H15 | -0.521307 | 211.69 | 497 | 306.73 |
| 1 | 10 | 78317588 | 78976608 | KCNMA1,KCNMA1,KCNMA1-AS1 | -0.527373 | 168.2 | 49 | 28.2518 |

| | | | | | | | | |
|---|---|---|---|---|---|---|---|---|
| 1 | 4 | 1.04E+08 | 114680590 | CENPE,TACR3,CXXC4,CXXC4,CXXC4-AS1,TET2,TET2-AS1,PPA2,ARHGEF38,INTS12,GSTCD,GSTCD,LOC101929529,NPNT,LOC101929577,TBCK,TBCK,AIMP1,AIMP1,GIMD1,DKK2,PAPSS1,SGMS2,LOC101929595,LOC101929595,CYP2U1,CYP2U1,HADH,LEF1,LEF1,LEF1-AS1,RPL34-AS1,RPL34,OSTC,ETNPPL,COL25A1,SEC24B,MCUB,CASP6,PLA2G12A,CFI,GAR1,RRH,LRIT3,EGF,ELOVL6,ENPEP,PITX2,FAM241A,AP1AR,TIFA,ALPK1,NEUROG2,NEUROG2,LOC105377372,LOC105377372,ZGRF1,ZGRF1,LARP7,LARP7,LOC109864269,LARP7,LOC109864269,MIR367,MIR302D,LARP7,LOC109864269,MIR302A,MIR302C,LARP7,LOC109864269,MIR302C,MIR302B,ANK2,ANK2,MIR1243,CAMK2D | -0.533476 | 199.288 | 779 | 465.816 |
| 1 | 17 | 28088780 | 29161430 | SSH2,SSH2,EFCAB5,EFCAB5,NSRP1,MIR423,MIR3184,NSRP1,SLC6A4,BLMH,TMIGD1,CPD,GOSR1,TBC1D29,LOC107133515,SH3GL1P2,LOC107133515,SUZ12P1,SUZ12P1,CRLF3,CRLF3,ATAD5 | -0.534111 | 212.614 | 178 | 101.567 |
| 1 | 3 | 93519633 | 108270213 | PROS1,ARL13B,ARL13B,STX19,DHFR2,DHFR2,NSUN3,NSUN3,EPHA6,ARL6,CRYBG3,CRYBG3,RIOX2,RIOX2,GABRR3,OR5AC2,OR5H1,OR5H14,OR5H15,OR5H6,OR5H2,OR5K4,OR5K3,OR5K1,OR5K2,CLDND1,GPR15,CPOX,ST3GAL6,DCBLD2,MIR548G,COL8A1,MIR548G,HP09053,CMSS1,MIR548G,CMSS1,MIR548G,CMSS1,FILIP1L,MIR548G,CMSS1,FILIP1L,MIR3921,CMSS1,TMEM30CP,TBC1D23,NIT2,TOMM70,LNP1,TMEM45A,ADGRG7,TFG,ABI3BP,IMPG2,SENP7,FAM172BP,TRMT10C,PCNP,ZBTB11,ZBTB11,ZBTB11-AS1,RPL24,CEP97,NXPE3,NFKBIZ,ZPLD1,ALCAM,CBLB,CCDC54,BBX,LINC00636,CD47,IFT57,HHLA2,MYH15,CIP2A | -0.534629 | 198.424 | 887 | 528.458 |
| 1 | 4 | 84406139 | 104004189 | ABRAXAS1,GPAT3,LOC101928978,NKX6-1,CDS1,WDFY3,ARHGAP24,ARHGAP24,MIR4451,MAPK10,MAPK10,LOC101929064,PTPN13,SLC10A6,C4orf36,AFF1,KLHL8,HSD17B13,HSD17B11,NUDT9,SPARCL1,DSPP,DMP1,IBSP,MEPE,SPP1,PKD2,ABCG2,PPM1K,HERC6,HERC5,PYURF,PIGY,HERC3,HERC3,NAP1L5,FAM13A-AS1,FAM13A,FAM13A,TIGD2,GPRIN3,SNCA,MMRN1,CCSER1,GRID2,ATOH1,SMARCAD1,HPGDS,PDLIM5,BMPR1B,UNC5C,PDHA2,STPG2,RAP1GDS1,TSPAN5,EIF4E,METAP1,METAP1,MIR3684,ADH5,LOC100507053,ADH4,LOC100507053,ADH6,LOC100507053,ADH1A,ADH1B,ADH1C,ADH7,C4orf17,TRMT10A,MTTP,LOC285556,DAPP1,LAMTOR3,DNAJB14,H2AFZ,DDIT4L,DDIT4L,LOC101929353,EMCN,PPP3CA,PPP3CA,MIR1255A,FLJ20021,BANK1,SLC39A8,NFKB1,MANBA,LOC102723704,UBE2D3,UBE2D3,CISD2,CISD2,SLC9B1,SLC9B1,SLC9B2,BDH2 | -0.53751 | 198.95 | 1296 | 766.289 |
| 1 | 2 | 86335408 | 86406647 | PTCD3,PTCD3,SNORD94,IMMT | -0.542838 | 199.716 | 44 | 25.25 |

| | | | | | | | | |
|---|---|---|---|---|---|---|---|---|
| 1 | 4 | 17814525 | 47033763 | NCAPG,NCAPG,LCORL,LCORL,SLIT2,SLIT2,MIR218-1,PACRGL,PACRGL,KCNIP4,KCNIP4,ADGRA3,GBA3,PPARGC1A,MIR573,DHX15,SOD3,CCDC149,LGI2,SEPSECS,PI4K2B,ZCCHC4,ANAPC4,SLC34A2,SEL1L3,SMIM20,RBPJ,CCKAR,TBC1D19,STIM2,MIR4275,PCDH7,ARAP2,LOC439933,DTHD1,MIR1255B1,MIR4801,NWD2,C4orf19,C4orf19,RELL1,RELL1,PGM2,TBC1D1,TBC1D1,PTTG2,KLF3,TLR10,TLR1,TLR6,FAM114A1,MIR574,FAM114A1,TMEM156,KLHL5,WDR19,RFC1,KLB,RPL9,LIAS,UGDH,UGDH-AS1,SMIM14,SMIM14,UBE2K,PDS5A,LOC344967,N4BP2,RHOH,CHRNA9,RBM47,RBM47,MIR4802,NSUN7,APBB2,UCHL1,LIMCH1,PHOX2B,TMEM33,DCAF4L1,SLC30A9,BEND4,SHISA3,ATP8A1,GRXCR1,KCTD8,YIPF7,GUF1,GNPDA2,GABRG1,GABRA2,COX7B2,GABRA4,GABRB1 | -0.544874 | 191.888 | 1413 | 841.597 |
| 1 | 4 | 83033382 | 84377449 | HNRNPD,HNRNPDL,ENOPH1,TMEM150C,SCD5,SCD5,MIR575,SEC31A,THAP9-AS1,THAP9,THAP9,LIN54,COPS4,PLAC8,COQ2,HPSE,HELQ,MRPS18C | -0.548606 | 201.68 | 182 | 107.725 |
| 1 | X | 1.23E+08 | 123217423 | THOC2,XIAP,STAG2 | -0.553857 | 206.677 | 59 | 35.0371 |
| 1 | 9 | 1.24E+08 | 123877547 | C5,CNTRL | -0.556845 | 206.919 | 22 | 12.5834 |
| 1 | 3 | 57232204 | 57899030 | HESX1,APPL1,APPL1,ASB14,APPL1,ASB14,LOC105377102,ASB14,LOC105377102,ASB14,DNAH12,PDE12,PDE12,ARF4,PDE12,DENND6A,DENND6A,SLMAP | -0.559087 | 236.795 | 165 | 97.2116 |
| 1 | 5 | 1.62E+08 | 166108911 | GABRG2,CCNG1,NUDCD2,HMMR,HMMR,HMMR-AS1,MAT2B | -0.566328 | 123.637 | 72 | 44.9281 |
| 1 | 3 | 56592736 | 56707747 | CCDC66,CCDC66,FAM208A,FAM208A | -0.566347 | 254.865 | 57 | 34.9249 |
| 1 | 1 | 91870345 | 94009780 | HFM1,CDC7,TGFBR3,BRDT,EPHX4,BTBD8,KIAA1107,C1orf146,GLMN,RPAP2,GFI1,EVI5,RPL5,RPL5,FAM69A,RPL5,FAM69A,SNORD21,RPL5,FAM69A,SNORA66,FAM69A,MTF2,TMED5,TMED5,CCDC18,CCDC18,DR1,FNBP1L | -0.573852 | 201.835 | 288 | 166.655 |
| 1 | 1 | 2.25E+08 | 225496132 | DNAH14 | -0.574333 | 189.304 | 74 | 41.992 |
| 1 | 17 | 67310452 | 70118047 | ABCA5,MAP2K6,KCNJ16,KCNJ2,SOX9 | -0.575593 | 172.449 | 52 | 32.9837 |
| 1 | 1 | 47753210 | 53535854 | STIL,CMPK1,LINC01389,FOXE3,FOXD2,TRABD2B,SLC5A9,SPATA6,AGBL4,AGBL4,BEND5,ELAVL4,DMRTA2,FAF1,CDKN2C,MIR4421,C1orf185,RNF11,TTC39A,TTC39A,TTC39A-AS1,EPS15,OSBPL9,NRDC,NRDC,MIR761,RAB3B,TXNDC12,TXNDC12,KTI12,TXNDC12,TXNDC12-AS1,BTF3L4,ZFYVE9,CC2D1B,ORC1,PRPF38A,ZCCHC11,GPX7,SHISAL2A,COA7,ZYG11B,ZYG11A,ECHDC2,SCP2,SCP2,MIR1273F,SCP2,MIR1273G,PODN | -0.577159 | 192.808 | 597 | 343.798 |
| 1 | 15 | 49304850 | 51242381 | SECISBP2L,COPS2,GALK2,GALK2,MIR4716,GALK2,FAM227B,FAM227B,FAM227B,FGF7,DTWD1,ATP8B4,SLC27A2,HDC,GABPB1,GABPB1,GABPB1-IT1,GABPB1-AS1,MIR4712,USP8,USP8,USP50,USP50,TRPM7,SPPL2A,AP4E1 | -0.579816 | 215.555 | 290 | 163.287 |
| 1 | 1 | 55542858 | 62904991 | USP24,MIR4422HG,MIR4422,PLPP3,LOC101929935,PRKAA2,PRKAA2,FYB2,C8A,C8B,DAB1,OMA1,TACSTD2,MYSM1,JUN,LINC01135,FGGY,FGGY,MIR4711,HOOK1,CYP2J2,C1orf87,NFIA,TM2D1,PATJ,PATJ,MIR3116-1,MIR3116-2,L1TD1,KANK4 | -0.580285 | 179.65 | 457 | 268.261 |

| | | | | | | | | |
|---|---|---|---|---|---|---|---|---|
| 1 | 12 | 1.12E+08 | 111993775 | ATXN2 | -0.585107 | 212.7 | 19 | 11.2784 |
| 1 | 9 | 1.03E+08 | 102860445 | STX17,ERP44 | -0.590174 | 185.509 | 26 | 13.9779 |
| 1 | 12 | 48057223 | 48096663 | RPAP3 | -0.598067 | 269.21 | 16 | 9.14534 |
| 1 | 9 | 1.05E+08 | 106864442 | LINC00587,CYLC2,SMC2 | -0.602713 | 110.488 | 35 | 21.2108 |
| 1 | 17 | 62850631 | 67085687 | LRRC37A3,AMZ2P1,GNA13,RGS9,AXIN2,CEP112,APOH,PRKCA,PRKCA,MIR634,CACNG5,CACNG4,CACNG1,HELZ,PSMD12,PITPNC1,PITPNC1,MIR548AA2,MIR548D2,NOL11,NOL11,SNORA38B,BPTF,C17orf58,KPNA2,LINC00674,ARHGAP27P2,AMZ2,ARSG,SLC16A6,ARSG,ARSG,PRKAR1A,ARSG,PRKAR1A,WIPI1,ARSG,PRKAR1A,WIPI1,MIR635,PRKAR1A,WIPI1,PRKAR1A,PRKAR1A,FAM20A,FAM20A,ABCA8,ABCA9,ABCA9,ABCA9-AS1,ABCA6 | -0.606863 | 215.114 | 543 | 311.67 |
| 1 | 15 | 43109884 | 43348633 | TTBK2,UBR1 | -0.607797 | 211.116 | 46 | 27.6258 |
| 1 | 8 | 38975487 | 39311049 | ADAM32 | -0.614365 | 195.673 | 34 | 19.1852 |
| 1 | 3 | 1.54E+08 | 159975745 | DHX36,GPR149,MME,PLCH1,PLCH1,PLCH1-AS2,C3orf33,SLC33A1,GMPS,KCNAB1,SSR3,TIPARP,LEKR1,CCNL1,VEPH1,VEPH1,PTX3,PQLC2L,SHOX2,RSRC1,MLF1,GFM1,GFM1,LXN,RARRES1,MFSD1,IQCJ,IQCJ-SCHIP1,IQCJ-SCHIP1,SCHIP1,IL12A-AS1,IL12A,IL12A-AS1,IL12A-AS1,LINC01100,C3orf80 | -0.619814 | 182.314 | 434 | 251.975 |
| 1 | 2 | 1.14E+08 | 118578958 | SLC35F5,SLC35F5,MIR4782,LOC101060091,ACTR3,ACTR3,DPP10,DDX18 | -0.621636 | 139.953 | 100 | 58.4659 |
| 1 | 15 | 40894984 | 40949657 | KNL1 | -0.632076 | 177.39 | 40 | 25.9497 |
| 1 | 12 | 85286386 | 88589408 | TSPAN19,LRRIQ1,ALX1,RASSF9,NTS,MGAT4C,LINC02258,MKRN9P,C12orf50,C12orf29,C12orf29,CEP290,CEP290,TMTC3 | -0.635243 | 212.485 | 192 | 114.352 |
| 1 | 16 | 8994451 | 9015236 | USP7 | -0.635697 | 219.955 | 21 | 11.4146 |
| 1 | 2 | 2.39E+08 | 238940980 | RBM44,RAMP1,UBE2F,UBE2F-SCLY | -0.639769 | 170.384 | 41 | 23.3914 |
| 1 | 14 | 1.01E+08 | 101455631 | [illegible]2,MEG8,MEG8,SNORD113-4,MEG8,SNORD113-5,MEG8,SNORD113-6,MEG8,SNORD113-7,MEG8,SNORD113-9,MEG8,SNORD114-1,MEG8,SNORD114-2,MEG8,SNORD114-3,MEG8,SNORD114-4,MEG8,SNORD114-5,MEG8,SNORD114-6,MEG8,SNORD114-7,MEG8,SNORD114-8,MEG8,SNORD114-9,MEG8,SNORD114-10,MEG8,SNORD114-11,MEG8,SNORD114-12,MEG8,SNORD114-13,MEG8,SNORD114-14,MEG8,SNORD114-15,MEG8,SNORD114-16,MEG8,SNORD114-17,MEG8,SNORD114-18,MEG8,SNORD114-19,MEG8,SNORD114-20,MEG8,SNORD114-21,MEG8,SNORD114-22,MEG8,SNORD114-23,MEG8,SNORD114-24,MEG8,SNORD114-25,MEG8,SNORD114-26,MEG8,SNORD114-27,MEG8,SNORD114-28 | -0.641317 | 227.945 | 46 | 26.6164 |
| 1 | 10 | 1.06E+08 | 114154888 | SLK,COL17A1,COL17A1,MIR936,SFR1,CFAP43,CFAP43,MIR609,GSTO1,MIR4482,GSTO2,ITPRIP,CFAP58,SORCS3,SORCS1,XPNPEP1,ADD3-AS1,ADD3,MXI1,SMNDC1,DUSP5,SMC3,RBM20,PDCD4,PDCD4,MIR4680,BBIP1,SHOC2,RPL13AP6,SHOC2,ADRA2A,GPAM,TECTB,ACSL5 | -0.650923 | 180.388 | 497 | 298.56 |
| 1 | 10 | 1.18E+08 | 118084935 | GFRA1,CCDC172 | -0.665921 | 120.926 | 22 | 12.0099 |

| | | | | | | | | |
|---|---|---|---|---|---|---|---|---|
| 1 | 10 | 68688768 | 70884084 | CTNNA3,LRRTM3,CTNNA3,DNAJC12,SIRT1,HERC4,MYPN,ATOH7,PBLD,HNRNPH3,HNRNPH3,RUFY2,RUFY2,DNA2,SLC25A16,TET1,CCAR1,CCAR1,SNORD98,CCAR1,MIR1254-1,STOX1,DDX50,DDX21,KIF1BP,SRGN,VPS26A | -0.670903 | 201.82 | 312 | 184.482 |
| 1 | 3 | 1.6E+08 | 182683535 | KPNA4,ARL14,PPM1L,B3GALNT1,NMD3,SPTSSB,LINC02067,OTOL1,MIR1263,SI,SLITRK3,BCHE,ZBBX,SERPINI2,WDR49,PDCD10,SERPINI1,GOLIM4,EGFEM1P,MIR551B,EGFEM1P,MECOM,TERC,ACTRT3,MYNN,LRRC34,LRRIQ4,LRRC31,SAMD7,SEC62,GPR160,PHC3,PRKCI,SKIL,CLDN11,SLC7A14,LOC101928583,LOC101928583,RPL22L1,EIF5A2,SLC2A2,TNIK,TNIK,MIR569,PLD1,TMEM212,FNDC3B,GHSR,TNFSF10,NCEH1,ECT2,SPATA16,NLGN1,NAALADL2,NAALADL2,NAALADL2-AS3,NAALADL2,NAALADL2-AS2,NAALADL2,MIR4789,TBL1XR1,KCNMB2-AS1,KCNMB2,ZMAT3,PIK3CA,PIK3CA,KCNMB3,KCNMB3,ZNF639,MFN1,GNB4,ACTL6A,MRPL47,NDUFB5,USP13,PEX5L,PEX5L,PEX5L-AS2,TTC14,TTC14,CCDC39,CCDC39,LOC101928882,FXR1,DNAJC19,SOX2-OT,SOX2,ATP11B,DCUN1D1 | -0.671544 | 178.498 | 1115 | 661.553 |
| 1 | 21 | 19715808 | 27141363 | TMPRSS15,MIR548XHG,NCAM2,MIR155HG,MIR155,MRPL39,JAM2,ATP5PF,ATP5PF,GABPA,GABPA | -0.678259 | 137.631 | 139 | 79.0995 |
| 1 | 10 | 92503278 | 98363870 | HTR7,RPP30,ANKRD1,NUDT9P1,PCGF5,HECTD2-AS1,HECTD2,PPP1R3C,TNKS2,FGFBP3,BTAF1,CPEB3,MARCH5,IDE,KIF11,HHEX,EXOC6,CYP26C1,CYP26A1,MYOF,CEP55,FFAR4,RBP4,PDE6C,FRA10AC1,LGI1,SLC35G1,PIPSL,PLCE1,PLCE1,PLCE1-AS2,PLCE1,PLCE1-AS1,NOC3L,TBC1D12,HELLS,CYP2C18,CYP2C19,CYP2C9,CYP2C8,ACSM6,PDLIM1,SORBS1,ALDH18A1,TCTN3,ENTPD1,ENTPD1,ENTPD1-AS1,ENTPD1-AS1,CC2D2B,ENTPD1-AS1,CCNJ,ENTPD1-AS1,MIR3157,ZNF518A,ZNF518A,BLNK,BLNK,DNTT,OPALIN,TLL2,TM9SF3,PIK3AP1 | -0.693335 | 192.761 | 902 | 536.004 |
| 1 | 5 | 68487522 | 68706460 | CENPH,MRPS36,CDK7,CCDC125,AK6,AK6,TAF9,AK6,TAF9,RAD17,RAD17 | -0.697299 | 214.998 | 71 | 38.6371 |
| 1 | 14 | 91927672 | 92584053 | PPP4R3A,CATSPERB,TC2N,FBLN5,TRIP11,ATXN3,NDUFB1 | -0.703559 | 201.801 | 141 | 79.0913 |
| 1 | 6 | 1.2E+08 | 125266581 | MAN1A1,TBC1D32,GJA1,HSF2,SERINC1,PKIB,FABP7,SMPDL3A,CLVS2,TRDN,TRDN,TRDN-AS1,NKAIN2,RNF217-AS1 | -0.708643 | 167.567 | 236 | 132.932 |
| 1 | 10 | 1.32E+08 | 135524247 | MIR378C,TCERG1L,LINC01164,PPP2R2D,BNIP3,JAKMIP3,DPYSL4,STK32C,LRRC27,PWWP2B,C10orf91,INPP5A,NKX6-2,CFAP46,ADGRA1,KNDC1,UTF1,VENTX,MIR202HG,MIR202,ADAM8,TUBGCP2,TUBGCP2,ZNF511,ZNF511,CALY,PRAP1,FUOM,ECHS1,ECHS1,MIR3944,PAOX,MTG1,SPRN,SCART1,CYP2E1,SYCE1,SYCE1,SPRNP1,FRG2B | -0.713535 | 159.72 | 481 | 287.558 |

| | | | | | | | | |
|---|---|---|---|---|---|---|---|---|
| 1 | 3 | 1.41E+08 | 150420003 | RASA2,RNF7,GRK7,ATP1B3,TFDP2,GK5,XRN1,ATR,PLS1,TRPC1,PCOLCE2,PAQR9,U2SURP,CHST2,SLC9A9,C3orf58,PLOD2,PLSCR4,PLSCR2,PLSCR1,PLSCR5,ZIC4,ZIC1,AGTR1,CPB1,CPA3,GYG1,HLTF,HLTF,HLTF-AS1,HPS3,HPS3,CP,CP,TM4SF18,TM4SF1,TM4SF4,WWTR1,COMMD2,ANKUB1,RNF13,PFN2,TSC22D2,SERP1,EIF2A,SELENOT,ERICH6 | -0.739231 | 185.867 | 718 | 418.968 |
| 1 | 19 | 34904625 | 34959992 | PDCD2L,UBA2 | -0.742833 | 212.361 | 25 | 13.4432 |
| 1 | 18 | 35690905 | 40849864 | MIR924HG,MIR924,MIR924HG,MIR5583-2,MIR5583-1,PIK3C3,RIT2 | -0.743846 | 114.45 | 72 | 44.5148 |
| 1 | 10 | 76911350 | 76995700 | SAMD8,VDAC2,COMTD1 | -0.743995 | 212.718 | 27 | 14.9671 |
| 1 | 1 | 62907116 | 89435236 | USP1,DOCK7,DOCK7,ANGPTL3,ATG4C,FOXD3-AS1,FOXD3,ALG6,ITGB3BP,EFCAB7,EFCAB7,DLEU2L,PGM1,ROR1,UBE2U,CACHD1,CACHD1,MIR4794,RAVER2,JAK1,JAK1,MIR3671,JAK1,MIR101-1,AK4,DNAJC6,LEPROT,LEPR,LEPR,PDE4B,SGIP1,SGIP1,MIR3117,TCTEX1D1,INSL5,WDR78,MIER1,SLC35D1,C1orf141,IL23R,IL12RB2,SERBP1,GADD45A,GNG12,GNG12-AS1,DIRAS3,GNG12-AS1,WLS,GNG12-AS1,WLS,MIR1262,WLS,RPE65,DEPDC1,DEPDC1,DEPDC1-AS1,LRRC7,LRRC40,SRSF11,ANKRD13C,HHLA3,CTH,PTGER3,PTGER3,ZRANB2-AS1,ZRANB2-AS1,ZRANB2,ZRANB2,MIR186,ZRANB2,NEGR1,LRRIQ3,FPGT,FPGT-TNNI3K,FPGT-TNNI3K,FPGT-TNNI3K,TNNI3K,ERICH3,ERICH3,ERICH3-AS1,CRYZ,CRYZ,TYW3,TYW3,LHX8,SLC44A5,ACADM,RABGGTB,RABGGTB,SNORD45C,RABGGTB,SNORD45A,RABGGTB,SNORD45B,MSH4,ASB17,ST6GALNAC3,ST6GALNAC5,PIGK,AK5,ZZZ3,USP33,MIGA1,NEXN,FUBP1,DNAJB4,GIPC2,PTGFR,IFI44L,IFI44,ADGRL4,ADGRL2,TTLL7,PRKACB,SAMD13,DNASE2B,RPF1,GNG5,SPATA1,SPATA1,CTBS,CTBS,LINC01555,SSX2IP,LPAR3,MCOLN2,MCOLN3,WDR63,MIR4423,SYDE2,C1orf52,BCL10,BCL10,LOC646626,DDAH1,CYR61,ZNHIT6,COL24A1,ODF2L,CLCA2,CLCA1,CLCA4,CLCA4,LOC105378828,LOC105378828,CLCA3P,SH3GLB1,SELENOF,HS2ST1,LINC01140,LMO4,PKN2-AS1,PKN2,PKN2,GTF2B,KYAT3 | -0.744457 | 182.867 | 1835 | 1074.56 |
| 1 | 12 | 1.11E+08 | 110647080 | IFT81 | -0.748643 | 147.48 | 16 | 8.71815 |
| 1 | 3 | 44284366 | 44362006 | TOPAZ1 | -0.753169 | 191.454 | 26 | 16.1221 |
| 1 | 8 | 39335787 | 39694801 | ADAM3A,ADAM18,ADAM2 | -0.757433 | 192.583 | 52 | 27.4868 |
| 1 | 10 | 1.27E+08 | 131934107 | TEX36-AS1,TEX36,TEX36,EDRF1,EDRF1,EDRF1-AS1,MMP21,UROS,UROS,MIR4484,BCCIP,BCCIP,DHX32,DHX32,FANK1,ADAM12,C10orf90,LOC728158,C10orf90,DOCK1,DOCK1,FAM196A,NPS,FOXI2,CLRN3,PTPRE,PTPRE,AS-PTPRE,MKI67,MGMT,EBF3,EBF3,MIR4297,C10orf143,CTAGE7P | -0.767622 | 176.726 | 374 | 226.992 |
| 1 | 16 | 66792669 | 66884600 | TERB1,NAE1,CA7 | -0.77539 | 222.42 | 39 | 23.1487 |

| | | | | | | | | |
|---|---|---|---|---|---|---|---|---|
| 1 | 13 | 96684755 | 115109378 | UGGT2,HS6ST3,HS6ST3,MIR4501,OXGR1,MBNL2,RAP2A,IPO5,FARP1,RNF113B,FARP1,STK24,SLC15A1,DOCK9,UBAC2,UBAC2,GPR18,UBAC2,GPR183,UBAC2,FKSG29,UBAC2,MIR623,TM9SF2,CLYBL,CLYBL,MIR4306,CLYBL,LOC101927437,ZIC5,ZIC2,PCCA,PCCA,GGACT,GGACT,TMTC4,NALCN-AS1,NALCN,NALCN,ITGBL1,FGF14,FGF14,MIR2681,FGF14,MIR4705,TPP2,METTL21C,CCDC168,TEX30,KDELC1,BIVM,BIVM-ERCC5,BIVM-ERCC5,ERCC5,SLC10A2,DAOA-AS1,DAOA,EFNB2,ARGLU1,FAM155A,FAM155A,MIR1267,LIG4,ABHD13,TNFSF13B,MYO16,MYO16,MYO16-AS1,IRS2,COL4A1,COL4A2,COL4A2,COL4A2-AS2,COL4A2,COL4A2-AS1,RAB20,NAXD,CARS2,ING1,LINC00346,ANKRD10,LINC00431,ARHGEF7-AS2,ARHGEF7,ARHGEF7,TEX29,LINC02337,SOX1-OT,SOX1,SPACA7,TUBGCP3,ATP11AUN,ATP11A,MCF2L-AS1,MCF2L,F7,F10,F10,F10-AS1,PROZ,PCID2,PCID2,CUL4A,CUL4A,LAMP1,GRTP1,GRTP1,GRTP1-AS1,ADPRHL1,DCUN1D2,DCUN1D2,TMCO3,TMCO3,TFDP1,ATP4B,GRK1,LINC00552,TMEM255B,GAS6-AS1,GAS6,GAS6,LINC00452,RASA3,CDC16,CDC16,MIR548AR,MIR4502,UPF3A,CHAMP1 | -0.788214 | 167.342 | 1477 | 892.892 |
| 1 | 17 | 49076731 | 53481280 | SPAG9,NME1-NME2,NME1,NME1-NME2,NME2,MBTD1,UTP18,CA10,C17orf112,KIF2B,TOM1L1,TOM1L1,COX11,COX11,STXBP4,HLF,MMD | -0.79386 | 167.328 | 187 | 109.918 |
| 1 | 17 | 44249208 | 45664180 | KANSL1,KANSL1,KANSL1-AS1,ARL17B,ARL17B,LRRC37A,ARL17A,ARL17B,ARL17A,NSFP1,ARL17A,LRRC37A2,ARL17B,ARL17A,ARL17B,NSF,NSFP1,NSF,WNT3,WNT9B,GOSR2,MIR5089,RPRML,CDC27,MYL4,ITGB3,ITGB3,THCAT158,EFCAB13,MRPL45P2,NPEPPS | -0.794614 | 184.242 | 210 | 106.164 |
| 1 | 10 | 55997229 | 61411999 | PCDH15,PCDH15,LOC105378311,PCDH15,LOC105378311,MIR548F1,ZWINT,MIR3924,IPMK,CISD1,UBE2D1,TFAM,BICC1,PHYHIPL,PHYHIPL,FAM13C,FAM13C | -0.796282 | 137.141 | 152 | 86.7078 |
| 1 | 13 | 96213253 | 96508586 | CLDN10,DZIP1,DNAJC3,UGGT2 | -0.797515 | 169.028 | 57 | 32.8526 |
| 1 | 1 | 1.73E+08 | 173569368 | SLC9C2 | -0.799576 | 197.632 | 20 | 10.9269 |
| 1 | 10 | 1.24E+08 | 124753555 | ARMS2,HTRA1,DMBT1,C10orf120,CUZD1,FAM24B-CUZD1,FAM24B-CUZD1,FAM24B,LOC399815,FAM24A,C10orf88,PSTK,IKZF5 | -0.800483 | 177.319 | 115 | 67.1339 |
| 1 | 20 | 29420069 | 29890459 | FRG1BP,FRG1DP,FRG1BP,DEFB115 | -0.803187 | 797.507 | 14 | 6.21905 |
| 1 | 4 | 1.86E+08 | 185652200 | CASP3,PRIMPOL,PRIMPOL,CENPU,CENPU | -0.803853 | 192.946 | 40 | 21.7072 |
| 1 | 15 | 99816716 | 100243011 | LRRC28,MEF2A | -0.805514 | 157.347 | 28 | 15.1094 |
| 1 | 17 | 58767032 | 59155894 | BCAS3 | -0.806003 | 166.661 | 36 | 18.4037 |

| | | | | | | | | |
|---|---|---|---|---|---|---|---|---|
| 1 | 13 | 48881413 | 53036581 | RB1,RB1,LPAR6,RCBTB2,CYSLTR2,FNDC3A,MLNR,CDADC1,CAB39L,SETDB2,SETDB2-PHF11,SETDB2-PHF11,PHF11,RCBTB1,ARL11,EBPL,KPNA3,CTAGE10P,SPRYD7,DLEU2,MIR3613,DLEU2,TRIM13,DLEU2,TRIM13,KCNRG,DLEU2,KCNRG,DLEU2,MIR16-1,MIR15A,DLEU2,DLEU1,DLEU1,ST13P4,DLEU7,DLEU7,DLEU7-AS1,RNASEH2B-AS1,RNASEH2B,RNASEH2B,GUCY1B2,FAM124A,SERPINE3,SERPINE3,MIR5693,SERPINE3,INTS6,INTS6,MIR4703,WDFY2,DHRS12,TMEM272,CCDC70,ATP7B,ALG11,ALG11,UTP14C,UTP14C,NEK5,NEK3,MRPS31P5,THSD1,VPS36,CKAP2 | -0.806033 | 174.451 | 490 | 290.344 |
| 1 | 2 | 26435330 | 26532579 | HADHA,HADHB,ADGRF3 | -0.816175 | 175.2 | 34 | 19.3628 |
| 1 | 20 | 34292455 | 34308112 | RBM39 | -0.820203 | 149.541 | 10 | 4.54616 |
| 1 | 15 | 57523332 | 57966648 | TCF12,CGNL1,MYZAP,GCOM1 | -0.828356 | 162.567 | 59 | 36.077 |
| 1 | X | 1.29E+08 | 129484639 | ZNF280C,SLC25A14 | -0.831635 | 191.09 | 26 | 15.1297 |
| 1 | 5 | 1.59E+08 | 158712036 | RNF145,UBLCP1 | -0.833803 | 171.746 | 28 | 15.6824 |
| 1 | 4 | 1.86E+08 | 186384217 | C4orf47,C4orf47,CCDC110,CCDC110 | -0.834855 | 189.006 | 18 | 11.9963 |
| 1 | 4 | 68338360 | 68562445 | CENPC,STAP1,UBA6 | -0.840656 | 172.088 | 77 | 42.3258 |
| 1 | 5 | 1.7E+08 | 170626792 | RANBP17 | -0.848785 | 154.638 | 25 | 12.853 |
| 1 | X | 1.18E+08 | 117810768 | DOCK11 | -0.854255 | 167.335 | 58 | 29.7673 |
| 1 | 1 | 1.76E+08 | 176175142 | RFWD2 | -0.855949 | 131.391 | 32 | 16.3281 |
| 1 | 11 | 1.03E+08 | 103191972 | DCUN1D5,DYNC2H1 | -0.862729 | 180.78 | 91 | 54.9579 |
| 1 | 16 | 46457168 | 46594033 | ANKRD26P1 | -0.866055 | 186.939 | 23 | 12.4353 |
| 1 | 10 | 1.24E+08 | 124097696 | TACC2,BTBD16 | -0.8685 | 173.732 | 34 | 21.0845 |
| 1 | 2 | 1.7E+08 | 170802983 | FASTKD1,PPIG,CCDC173,PHOSPHO2-KLHL23,PHOSPHO2,PHOSPHO2-KLHL23,KLHL23,SSB,SSB,METTL5,METTL5,UBR3 | -0.869043 | 180.161 | 118 | 63.9108 |
| 1 | 2 | 32093517 | 32422889 | MEMO1,DPY30,SPAST,SLC30A6 | -0.869641 | 158.218 | 59 | 29.1539 |
| 1 | 1 | 35452708 | 35864124 | ZMYM6,ZMYM1,SFPQ,ZMYM4,ZMYM4,ZMYM4-AS1 | -0.876292 | 191.542 | 87 | 52.8631 |
| 1 | 4 | 1.46E+08 | 146095935 | ANAPC10,ABCE1,OTUD4 | -0.884663 | 209.021 | 63 | 31.2408 |
| 1 | 9 | 33533383 | 33567306 | ANKRD18B | -0.888012 | 366.856 | 13 | 7.06106 |
| 1 | 10 | 1.14E+08 | 117228911 | ACSL5,ZDHHC6,ZDHHC6,VTI1A,VTI1A,VTI1A,MIR4295,TCF7L2,HABP2,HABP2,NRAP,NRAP,CASP7,PLEKHS1,PLEKHS1,MIR4483,DCLRE1A,NHLRC2,ADRB1,CCDC186,CCDC186,MIR2110,TDRD1,VWA2,AFAP1L2,ABLIM1,ABLIM1,LOC101927692,FAM160B1,TRUB1,ATRNL1 | -0.902445 | 161.132 | 409 | 247.502 |
| 1 | 8 | 43152552 | 43398986 | POTEA | -0.903021 | 121.174 | 13 | 5.70443 |
| 1 | 5 | 43479912 | 43556148 | TMEM267,C5orf34,PAIP1 | -0.904881 | 147.808 | 26 | 14.4346 |
| 1 | 10 | 88811598 | 90537979 | GLUD1,FAM35A,MIR4678,MINPP1,PAPSS2,ATAD1,KLLN,PTEN,RNLS,LIPJ,LIPF,LIPK,LIPN | -0.905061 | 170.787 | 158 | 88.287 |
| 1 | 9 | 79814790 | 80022620 | VPS13A | -0.910839 | 178.491 | 75 | 43.3008 |

| | | | | | | | | |
|---|---|---|---|---|---|---|---|---|
| 1 | 13 | 53262521 | 84453105 | CNMD,MIR759,PCDH8,OLFM4,MIR1297,MIR5007,PCDH17,DIAPH3,DIAPH3,DIAPH3-AS1,TDRD3,MIR3169,PCDH20,MIR548X2,MIR4704,PCDH9,PCDH9,PCDH9-AS2,KLHL1,KLHL1,ATXN8OS,DACH1,MZT1,BORA,DIS3,PIBF1,KLF5,KLF12,LOC100288208,TBC1D4,COMMD6,UCHL3,UCHL3,LMO7-AS1,LMO7-AS1,LMO7,LMO7,LMO7DN,KCTD12,ACOD1,CLN5,FBXL3,MYCBP2,MYCBP2,MYCBP2-AS1,SCEL,SCEL,SCEL-AS1,SLAIN1,MIR3665,SLAIN1,EDNRB-AS1,EDNRB,EDNRB,RNF219-AS1,POU4F1,RNF219-AS1,RNF219,RNF219,RBM26,NDFIP2-AS1,NDFIP2,NDFIP2,SPRY2 | -0.914422 | 134.978 | 817 | 492.925 |
| 1 | 20 | 58425361 | 58497518 | SYCP2 | -0.918083 | 175.159 | 41 | 24.2543 |
| 1 | 2 | 97788579 | 98197035 | ANKRD36,ANKRD36B | -0.929717 | 259.691 | 41 | 17.2606 |
| 1 | 2 | 11312739 | 11597301 | PQLC3,ROCK2,E2F6 | -0.94357 | 193.036 | 48 | 27.8337 |
| 1 | 7 | 74105344 | 74167458 | GTF2I,LOC101926943,GTF2I | -0.957462 | 232.847 | 14 | 6.64235 |
| 1 | 2 | 73957005 | 74002249 | TPRKB,DUSP11 | -0.963146 | 140.648 | 13 | 7.97177 |
| 1 | 2 | 39223978 | 39605278 | SOS1,CDKL4,MAP4K3 | -0.965136 | 161.115 | 72 | 41.4734 |
| 1 | 2 | 96521007 | 96648124 | ANKRD36C | -0.968507 | 359.54 | 24 | 10.4243 |
| 1 | 2 | 71582792 | 71645853 | ZNF638 | -0.96863 | 187.343 | 26 | 15.2869 |
| 1 | 21 | 38459651 | 38564608 | TTC3,TTC3,TTC3-AS1 | -0.971059 | 193.274 | 63 | 32.3641 |
| 1 | 2 | 37315391 | 37449657 | GPATCH11,EIF2AK2,SULT6B1,CEBPZOS,CEBPZOS,CEBPZ,CEBPZ | -0.973117 | 198.192 | 50 | 28.0224 |
| 1 | 5 | 37000879 | 37046351 | NIPBL | -0.975676 | 198.133 | 23 | 14.5847 |
| 1 | 11 | 1.19E+08 | 118651963 | DDX6 | -0.978162 | 140.901 | 18 | 9.34914 |
| 1 | 15 | 62212720 | 62351931 | VPS13C | -0.981871 | 185.467 | 57 | 34.4179 |
| 1 | 17 | 37420389 | 37561699 | FBXL20,MED1 | -0.994535 | 143.31 | 20 | 11.4083 |
| 1 | 2 | 27891693 | 27917484 | SLC4A1AP | -0.998328 | 168.861 | 16 | 8.67441 |
| 1 | 9 | 72914959 | 72965395 | SMC5 | -0.999442 | 155.975 | 16 | 8.69628 |
| 1 | 9 | 26984295 | 27061103 | IFT74,IFT74,LRRC19 | -0.999518 | 164.124 | 21 | 13.2702 |
| 1 | 22 | 45748243 | 45804789 | SMC1B | -1.00764 | 203.861 | 23 | 14.1931 |
| 1 | 12 | 20968671 | 21716290 | SLCO1B3,SLCO1B7,SLCO1B1,SLCO1A2,SLCO1A2,IAPP,PYROXD1,PYROXD1,RECQL,RECQL,GOLT1B,SPX,GYS2 | -1.0087 | 162.807 | 144 | 76.7238 |
| 1 | 13 | 88636973 | 95673257 | MIR17HG,MIR17,MIR18A,MIR17HG,MIR19A,MIR20A,MIR17HG,MIR19B1,MIR92A1,GPC5,GPC5,MIR548AS,GPC6,GPC6,GPC6-AS2,DCT,TGDS,GPR180,SOX21 | -1.01729 | 99.979 | 117 | 70.6884 |
| 1 | 5 | 72147148 | 72354387 | TNPO1,TNPO1,MIR4804,FCHO2 | -1.03327 | 164.951 | 51 | 25.9336 |
| 1 | 2 | 1.21E+08 | 120844906 | PTPN4,EPB41L5 | -1.0381 | 162.842 | 42 | 22.7828 |
| 1 | 10 | 27294428 | 27431433 | ANKRD26,YME1L1 | -1.04576 | 175.771 | 59 | 33.0248 |
| 1 | 10 | 1.18E+08 | 118424504 | PNLIPRP3,PNLIP,PNLIPRP1,PNLIPRP2,C10orf82 | -1.05615 | 149.722 | 57 | 35.8647 |
| 1 | 2 | 75874150 | 75928448 | MRPL19,GCFC2 | -1.0698 | 166.303 | 21 | 12.4435 |
| 1 | 8 | 1.31E+08 | 130874646 | FAM49B | -1.09407 | 164.219 | 11 | 6.09457 |
| 1 | 3 | 1.95E+08 | 195256798 | XXYLT1,ACAP2,PPP1R2 | -1.10784 | 147.672 | 45 | 24.3843 |
| 1 | 9 | 19058941 | 19096854 | HAUS6,HAUS6,SCARNA8 | -1.11306 | 132.139 | 18 | 8.47488 |
| 1 | 17 | 38548251 | 38573113 | TOP2A | -1.12446 | 182.153 | 32 | 19.7772 |
| 1 | 10 | 1.24E+08 | 123792796 | ATE1,ATE1,ATE1-AS1,NSMCE4A,TACC2 | -1.12694 | 118.411 | 32 | 17.972 |
| 1 | 15 | 44615047 | 44816344 | CASC4,CTDSPL2 | -1.13613 | 124.002 | 31 | 15.4939 |
| 1 | 2 | 1.72E+08 | 172305341 | TLK1,METTL8,METTL8,DCAF17,DCAF17 | -1.14107 | 110.637 | 53 | 27.7789 |
| 1 | 15 | 79170435 | 79189324 | MORF4L1 | -1.1463 | 184.406 | 14 | 7.86229 |
| 1 | 1 | 2.47E+08 | 247091731 | AHCTF1 | -1.14907 | 118.808 | 32 | 14.8504 |
| 1 | 4 | 1.7E+08 | 170523809 | NEK1 | -1.16335 | 162.111 | 13 | 7.91516 |
| 1 | 2 | 2.14E+08 | 214239890 | SPAG16 | -1.17263 | 122.027 | 14 | 7.62777 |
| 1 | 18 | 30518568 | 31224276 | CCDC178,ASXL3 | -1.17878 | 98.3188 | 42 | 21.4036 |
| 1 | 2 | 55536143 | 55914912 | CCDC88A,CFAP36,PPP4R3B,PNPT1 | -1.18116 | 150.485 | 96 | 55.7968 |

| | | | | | | | | |
|---|---|---|---|---|---|---|---|---|
| 1 | 8 | 1.25E+08 | 124820702 | FAM91A1 | -1.18362 | 156.291 | 21 | 10.6374 |
| 1 | 11 | 1.12E+08 | 112042669 | IL18,TEX12 | -1.18694 | 124.056 | 10 | 5.42713 |
| 1 | 14 | 53110194 | 53251443 | ERO1A,PSMC6,STYX,GNPNAT1 | -1.21502 | 130.054 | 50 | 26.8483 |
| 1 | 18 | 65182367 | 66381272 | TMX3 | -1.23067 | 91.0608 | 22 | 13.5975 |
| 1 | 18 | 14763968 | 14851759 | ANKRD30B,ANKRD30B,MIR3156-2 | -1.24963 | 201.786 | 24 | 11.6319 |
| 1 | 8 | 95738471 | 95802201 | DPY19L4 | -1.25867 | 120.049 | 22 | 10.4193 |
| 1 | 3 | 5212065 | 5216226 | ARL8B | -1.27749 | 119.541 | 10 | 5.3846 |
| 1 | 12 | 76443191 | 76468034 | NAP1L1 | -1.28358 | 131.763 | 19 | 10.3181 |
| 1 | 1 | 1.51E+08 | 150692062 | HORMAD1 | -1.29988 | 128.318 | 17 | 8.68101 |
| 1 | 14 | 94706923 | 94733399 | PPP4R4 | -1.30036 | 169.906 | 15 | 9.24548 |
| 1 | 2 | 1.54E+08 | 153547641 | PRPF40A | -1.30569 | 143.849 | 21 | 12.9273 |
| 1 | 17 | 67087299 | 67309407 | ABCA6,ABCA6,MIR4524B,MIR4524A,ABCA10,ABCA5 | -1.31825 | 129.207 | 109 | 62.3343 |
| 1 | 11 | 94153276 | 94229910 | MRE11,MRE11,MIR548L,ANKRD49 | -1.32026 | 126.678 | 30 | 17.1285 |
| 1 | 1 | 91407353 | 91866661 | ZNF644,HFM1 | -1.32703 | 100.397 | 41 | 23.9484 |
| 1 | 12 | 1.01E+08 | 100729623 | SCYL2 | -1.32866 | 133.106 | 20 | 11.1436 |
| 1 | 4 | 1.04E+08 | 104118088 | BDH2,CENPE | -1.33323 | 132.529 | 58 | 35.3554 |
| 1 | 3 | 1.6E+08 | 160254708 | TRIM59-IFT80,IFT80,TRIM59-IFT80,SMC4,TRIM59-IFT80,SMC4,MIR15B,MIR16-2,TRIM59-IFT80,TRIM59,TRIM59-IFT80,KPNA4,KPNA4,SCARNA7 | -1.34712 | 119.633 | 87 | 47.3123 |
| 1 | 15 | 60724015 | 60768436 | ICE2 | -1.34825 | 150.673 | 22 | 11.6809 |
| 1 | 15 | 25584241 | 25616042 | SNHG14,UBE3A | -1.3517 | 158.857 | 12 | 6.74265 |
| 1 | 17 | 29161430 | 29221974 | ATAD5 | -1.35753 | 141.822 | 29 | 18.4293 |
| 1 | 2 | 1.72E+08 | 171649491 | ERICH2 | -1.37112 | 149.774 | 10 | 5.51103 |
| 1 | 2 | 1.09E+08 | 109102250 | GCC2 | -1.37454 | 119.144 | 17 | 10.4036 |
| 1 | 3 | 1.08E+08 | 108361485 | CIP2A,DZIP3 | -1.37699 | 138.54 | 34 | 20.8665 |
| 1 | 10 | 32740575 | 33197337 | CCDC7,ITGB1 | -1.39606 | 82.5452 | 58 | 30.2369 |
| 1 | 17 | 30205742 | 30300264 | UTP6,SUZ12 | -1.39958 | 129.048 | 24 | 11.7809 |
| 1 | 18 | 9104885 | 9134187 | NDUFV2,NDUFV2,NDUFV2-AS1 | -1.39997 | 123.218 | 10 | 5.01911 |
| 1 | 3 | 1.21E+08 | 120969692 | STXBP5L | -1.41161 | 98.3298 | 14 | 7.73719 |
| 1 | 3 | 1.12E+08 | 112052227 | SLC9C1,CD200 | -1.42258 | 101.391 | 31 | 16.8074 |
| 1 | 9 | 15468756 | 15506699 | PSIP1 | -1.43828 | 106.941 | 18 | 9.3687 |
| 1 | 13 | 21950728 | 22096833 | MIPEPP3,ZDHHC20,ZDHHC20,MICU2 | -1.44607 | 114.411 | 29 | 15.5255 |
| 1 | 8 | 1.26E+08 | 125535312 | TATDN1,TATDN1,MIR6844 | -1.4512 | 112.524 | 16 | 7.8584 |
| 1 | 18 | 48422261 | 48450647 | ME2 | -1.47616 | 125.801 | 13 | 7.6599 |
| 1 | 12 | 50822684 | 50848230 | LARP4 | -1.48187 | 102.586 | 16 | 8.58903 |
| 1 | 20 | 13568505 | 13763852 | TASP1,ESF1 | -1.48386 | 105.871 | 30 | 16.1727 |
| 1 | 18 | 9239420 | 9256628 | ANKRD12 | -1.49867 | 80.7896 | 11 | 6.79785 |
| 1 | 15 | 34434595 | 34455978 | KATNBL1 | -1.51771 | 161.402 | 11 | 5.41265 |
| 1 | 10 | 91468950 | 92502413 | KIF20B,HTR7 | -1.52005 | 85.8748 | 40 | 24.9537 |
| 1 | 16 | 10525192 | 10567925 | ATF7IP2 | -1.56532 | 102.795 | 14 | 7.33204 |
| 1 | 7 | 34982191 | 35058293 | DPY19L1 | -1.58611 | 99.2391 | 19 | 9.21686 |
| 1 | 1 | 1.15E+08 | 115537629 | SYCP1 | -1.59015 | 67.0969 | 31 | 16.1533 |
| 1 | 15 | 56665563 | 57522832 | TEX9,TEX9,MNS1,MNS1,ZNF280D,LOC145783,TCF12,TCF12 | -1.65264 | 99.3562 | 71 | 41.5589 |
| 1 | 6 | 88303955 | 88331801 | ORC3 | -1.65342 | 105.512 | 15 | 8.47012 |
| 1 | 10 | 71917440 | 71990228 | SAR1A,PPA1 | -1.69784 | 97.1391 | 21 | 10.2645 |
| 1 | 6 | 1.5E+08 | 149862732 | PPIL4 | -1.7111 | 100.198 | 13 | 6.87271 |
| 1 | 2 | 1.98E+08 | 197762011 | PGAP1 | -1.79167 | 88.8528 | 10 | 6.06867 |
| 1 | 13 | 96508866 | 96684255 | UGGT2 | -1.8443 | 76.9152 | 41 | 23.1292 |
| 1 | 13 | 53036582 | 53262021 | CKAP2,SUGT1 | -1.95088 | 100.993 | 23 | 12.698 |
| 1 | 10 | 1.32E+08 | 131977594 | GLRX3 | -2.0177 | 66.2532 | 14 | 6.66714 |
| 1 | 4 | 84377980 | 84403434 | MRPS18C,MRPS18C,ABRAXAS1,ABRAXAS1 | -2.02263 | 80.5984 | 19 | 9.87191 |
| 1 | 3 | 1.22E+08 | 121544959 | IQCB1 | -2.02353 | 60.7589 | 10 | 4.42103 |
| 1 | 10 | 70892605 | 70928313 | VPS26A | -2.09475 | 95.9577 | 14 | 7.45539 |
| 1 | 10 | 1.18E+08 | 118187635 | CCDC172,PNLIPRP3 | -2.25071 | 33.8385 | 11 | 5.77939 |
| 1 | Y | 2656174 | 10058894 | TGIF2LY,PCDH11Y,TBL1Y,PRKY | -3.7205 | 28.7449 | 90 | 48.1936 |

| | | | | | | | | |
|---|---|---|---|---|---|---|---|---|
| 1 | 22 | 24345594 | 24384315 | GSTTP1,LOC391322,GSTT1 | -5.60027 | 4.36747 | 13 | 5.62091 |
| 1 | Y | 13105053 | 59106039 | GYG2P1,UTY,VCY1B,VCY,VCY,VCY1B,NLGN4Y,TTTY14,CD24 | -6.21296 | 4.7257 | 151 | 84.6589 |
| 1 | 8 | 39311549 | 39335287 | ADAM3A | -21.8558 | 0.895811 | 10 | 3.71618 |
| 2 | 1 | 1.5E+08 | 149859508 | HIST2H2BF,HIST2H4A,HIST2H4B,HIST2H3C,HIST2H3A,HIST2H2AA3,HIST2H2AA4,HIST2H2BC,HIST2H2BE,HIST2H2AC,HIST2H2AB | 1.77203 | 1011.89 | 23 | 10.5838 |
| 2 | 3 | 4942887 | 5211565 | BHLHE40-AS1,BHLHE40,BHLHE40,ARL8B | 1.40213 | 790.45 | 14 | 8.49661 |
| 2 | 7 | 1.44E+08 | 144072748 | LOC101928605,OR2A1-AS1,OR2A1,OR2A42,LOC101928605,OR2A1-AS1,OR2A9P,OR2A20P,LOC101928605,OR2A1-AS1,OR2A7,LOC101928605,OR2A1-AS1,OR2A7,ARHGEF34P,LOC101928605,OR2A1-AS1,ARHGEF34P,OR2A1-AS1,OR2A20P,OR2A9P,OR2A1-AS1,OR2A1,OR2A42,ARHGEF5 | 1.24375 | 830.979 | 68 | 29.7297 |
| 2 | 19 | 36230126 | 36236941 | IGFLR1,IGFLR1,U2AF1L4,U2AF1L4,PSENEN | 1.12942 | 502.383 | 14 | 8.71254 |
| 2 | 1 | 1.62E+08 | 161719930 | FCRLB,DUSP12 | 1.06362 | 587.6 | 10 | 6.93099 |
| 2 | 8 | 1.42E+08 | 146303522 | DENND3,SLC45A4,SLC45A4,LOC105375787,LOC105375787,GPR20,PTP4A3,MROH5,MIR1302-7,MIR4472-1,TSNARE1,ADGRB1,ARC,JRK,PSCA,LY6K,LY6K,LOC100288181,THEM6,SLURP1,LYPD2,SLURP2,LYNX1-SLURP2,LYNX1-SLURP2,LYNX1,LY6D,GML,CYP11B1,CYP11B2,LY6E-DT,LY6E,C8orf31,LY6H,GPIHBP1,ZFP41,GLI4,ZNF696,TOP1MT,RHPN1,MAFA,ZC3H3,GSDMD,MROH6,NAPRT,EEF1D,TIGD5,PYCR3,TSTA3,ZNF623,ZNF707,CCDC166,MAPK15,FAM83H,FAM83H,MIR4664,FAM83H-AS1,SCRIB,SCRIB,MIR937,PUF60,NRBP2,NRBP2,MIR6845,EPPK1,PLEC,PLEC,MIR661,PARP10,GRINA,SPATC1,OPLAH,OPLAH,MIR6846,EXOSC4,EXOSC4,MIR6847,GPAA1,CYC1,SHARPIN,MAF1,WDR97,HGH1,MROH1,MIR7112,SCX,BOP1,BOP1,MIR7112,BOP1,SCX,HSF1,DGAT1,DGAT1,MIR6848,SCRT1,TMEM249,TMEM249,FBXL6,FBXL6,SLC52A2,ADCK5,CPSF1,CPSF1,MIR939,CPSF1,MIR6849,SLC39A4,VPS28,TONSL,TONSL,TONSL-AS1,MIR6893,TONSL,TONSL-AS1,CYHR1,KIFC2,FOXH1,PPP1R16A,GPT,MFSD3,RECQL4,LRRC14,LRRC14,LRRC24,C8orf82,ARHGAP39,ZNF251,ZNF34,RPL8,RPL8,MIR6850,ZNF517,ZNF517,LOC100130027,ZNF7,COMMD5,ZNF250,ZNF16,ZNF252P,ZNF252P,TMED10P1,ZNF252P-AS1,C8orf33 | 0.936071 | 445.503 | 1230 | 767.994 |
| 2 | 13 | 58206674 | 58299462 | PCDH17 | 0.861776 | 680.07 | 15 | 10.1468 |
| 2 | 15 | 44004527 | 44020871 | STRCP1 | 0.859838 | 278.514 | 20 | 10.5916 |
| 2 | 15 | 43904540 | 43910582 | STRC | 0.78183 | 275.796 | 19 | 10.7989 |
| 2 | 1 | 1.47E+08 | 147401012 | NBPF19,BCL9,NBPF19,ACP6,NBPF19,GJA5,NBPF19,GJA8,NBPF19,GPR89B | 0.769449 | 433.884 | 43 | 27.6475 |
| 2 | 11 | 72852856 | 73057364 | FCHSD2,P2RY2,P2RY6,LOC100287837,ARHGEF17,ARHGEF17 | 0.760279 | 425.805 | 26 | 17.677 |

| | | | | | | | | |
|---|---|---|---|---|---|---|---|---|
| 2 | 13 | 1.1E+08 | 115002416 | MYO16,MYO16,MYO16-AS1,IRS2,COL4A1,COL4A2,COL4A2,COL4A2-AS2,COL4A2,COL4A2-AS1,RAB20,NAXD,CARS2,ING1,LINC00346,ANKRD10,LINC00431,ARHGEF7-AS2,ARHGEF7,ARHGEF7,TEX29,LINC02337,SOX1-OT,SOX1,SPACA7,TUBGCP3,ATP11AUN,ATP11A,MCF2L-AS1,MCF2L,F7,F10,F10,F10-AS1,PROZ,PCID2,PCID2,CUL4A,CUL4A,LAMP1,GRTP1,GRTP1,GRTP1-AS1,ADPRHL1,DCUN1D2,DCUN1D2,TMCO3,TMCO3,TFDP1,ATP4B,GRK1,LINC00552,TMEM255B,GAS6-AS1,GAS6,GAS6,LINC00452,RASA3,CDC16 | 0.746837 | 430.517 | 628 | 375.772 |
| 2 | 8 | 21863125 | 22103204 | NPM2,FGF17,DMTN,FAM160B2,NUDT18,HR,REEP4,LGI3,SFTPC,BMP1,PHYHIP,MIR320A,POLR3D,POLR3D | 0.728937 | 446.789 | 143 | 91.0091 |
| 2 | 16 | 69345322 | 69377518 | VPS4A,PDF,COG8,COG8,COG8,NIP7,NIP7,TMED6 | 0.696934 | 409.319 | 27 | 17.5848 |
| 2 | 1 | 1.5E+08 | 150667365 | RPRD2,TARS2,TARS2,MIR6878,ECM1,ADAMTSL4,ADAMTSL4,MIR4257,ADAMTSL4-AS1,MCL1,ENSA,GOLPH3L | 0.686923 | 412.287 | 95 | 57.7382 |
| 2 | 10 | 1.24E+08 | 123971231 | NSMCE4A,TACC2 | 0.665635 | 328.29 | 49 | 30.0243 |
| 2 | 5 | 10500 | 4087339 | PLEKHG4B,LRRC14B,CCDC127,SDHA,PDCD6,PDCD6,AHRR,AHRR,EXOC3-AS1,EXOC3,PP7080,SLC9A3,SLC9A3,SLC9A3-AS1,SLC9A3,MIR4456,CEP72,TPPP,ZDHHC11B,ZDHHC11,BRD9,TRIP13,LOC100506688,NKD2,SLC12A7,SLC12A7,MIR4635,SLC6A19,SLC6A18,TERT,MIR4457,CLPTM1L,LINC01511,SLC6A3,LPCAT1,SDHAP3,LOC728613,MIR4277,MRPL36,NDUFS6,IRX4,IRX2,C5orf38,LINC01019,IRX1 | 0.656869 | 406.547 | 439 | 251.284 |
| 2 | 16 | 67180845 | 67322485 | C16orf70,B3GNT9,TRADD,FBXL8,FBXL8,HSF4,HSF4,NOL3,KIAA0895L,EXOC3L1,E2F4,ELMO3,ELMO3,MIR328,LRRC29,LRRC29,TMEM208,TMEM208,FHOD1,SLC9A5,PLEKHG4 | 0.644458 | 398.049 | 157 | 105.931 |
| 2 | 1 | 3774077 | 7796549 | DFFB,C1orf174,AJAP1,MIR4417,MIR4689,NPHP4,NPHP4,KCNAB2,CHD5,RPL22,RNF207,ICMT,HES3,GPR153,ACOT7,HES2,ESPN,ESPN,MIR4252,TNFRSF25,TNFRSF25,PLEKHG5,PLEKHG5,NOL9,TAS1R1,ZBTB48,KLHL21,PHF13,THAP3,THAP3,DNAJC11,DNAJC11,CAMTA1 | 0.624424 | 365.402 | 413 | 241.06 |
| 2 | 12 | 1.33E+08 | 133202938 | DDX51,NOC4L,GALNT9,GALNT9,LOC100130238,FBRSL1,LRCOL1,P2RX2,POLE | 0.615925 | 375.708 | 95 | 54.8648 |
| 2 | 16 | 31987810 | 34257343 | SLC6A10P | 0.609962 | 393.465 | 107 | 48.0297 |

| | | | | | | | | |
|---|---|---|---|---|---|---|---|---|
| 2 | 16 | 29142095 | 31191655 | SNX29P2,BOLA2-SMG1P6,LOC606724,BOLA2-SMG1P6,BOLA2,BOLA2B,BOLA2-SMG1P6,BOLA2B,BOLA2,SLX1B,SLX1A,SLX1B,SLX1A,SLX1A-SULT1A3,SLX1B-SULT1A4,SLX1A-SULT1A3,SLX1B-SULT1A4,SLX1A-SULT1A3,SLX1B-SULT1A4,SULT1A3,SULT1A4,LOC388242,LOC613038,SMG1P2,SMG1P2,MIR3680-2,MIR3680-1,SPN,QPRT,C16orf54,ZG16,KIF22,MAZ,PRRT2,PAGR1,MVP,CDIPT,CDIPT-AS1,SEZ6L2,ASPHD1,KCTD13,TMEM219,TAOK2,HIRIP3,INO80E,DOC2A,C16orf92,C16orf92,FAM57B,FAM57B,ALDOA,PPP4C,TBX6,YPEL3,LOC101928595,GDPD3,GDPD3,MAPK3,CORO1A,CORO1A,LOC606724,BOLA2,BOLA2B,BOLA2B,BOLA2,SLX1B,SLX1A,CD2BP2,TBC1D10B,MYLPF,SEPT1,SEPT1,ZNF48,ZNF48,ZNF771,DCTPP1,SEPHS2,ITGAL,ITGAL,MIR4518,ZNF768,ZNF747,ZNF764,ZNF688,ZNF785,ZNF689,PRR14,FBRS,SRCAP,SRCAP,SNORA30,PHKG2,PHKG2,CCDC189,CCDC189,RNF40,RNF40,ZNF629,BCL7C,BCL7C,MIR4519,BCL7C,MIR762HG,BCL7C,MIR762HG,MIR762,CTF1,FBXL19-AS1,FBXL19,FBXL19,ORAI3,SETD1A,HSD3B7,STX1B,STX4,ZNF668,ZNF646,PRSS53,VKORC1,BCKDK,KAT8,PRSS8,PRSS36,FUS | 0.605919 | 403.43 | 782 | 488.06 |
| 2 | 8 | 1.33E+08 | 133596033 | EFR3A,OC90,HHLA1,KCNQ3,LRRC6 | 0.599318 | 372.468 | 72 | 44.212 |
| 2 | 13 | 53262521 | 54049415 | CNMD,MIR759,PCDH8,OLFM4 | 0.589471 | 405.135 | 37 | 21.9251 |
| 2 | 1 | 1.55E+08 | 156918469 | ADAR,KCNN3,PMVK,PBXIP1,PYGO2,PYGO2,LOC101928120,SHC1,CKS1B,CKS1B,MIR4258,FLAD1,LENEP,ZBTB7B,ZBTB7B,DCST2,DCST2,DCST1,DCST1,DCST1-AS1,DCST1-AS1,ADAM15,DCST1-AS1,EFNA4,EFNA4,EFNA3,EFNA1,SLC50A1,DPM3,KRTCAP2,TRIM46,MUC1,MIR92B,THBS3,MTX1,MTX1,GBAP1,GBAP1,GBA,FAM189B,SCAMP3,CLK2,HCN3,PKLR,FDPS,FDPS,RUSC1-AS1,RUSC1-AS1,RUSC1,RUSC1,ASH1L,ASH1L,MIR555,ASH1L,POU5F1P4,ASH1L,ASH1L-AS1,ASH1L-AS1,MSTO1,MSTO2P,YY1AP1,DAP3,MSTO2P,MSTO2P,GON4L,GON4L,SYT11,RIT1,KHDC4,KHDC4,SNORA80E,KHDC4,SCARNA4,RXFP4,ARHGEF2,ARHGEF2,MIR6738,SSR2,UBQLN4,LAMTOR2,RAB25,MEX3A,LMNA,SEMA4A,SLC25A44,PMF1,PMF1-BGLAP,PMF1-BGLAP,BGLAP,PAQR6,SMG5,SMG5,TMEM79,GLMP,VHLL,CCT3,CCT3,TSACC,TSACC,RHBG,C1orf61,C1orf61,MIR9-1,MEF2D,IQGAP3,TTC24,NAXE,GPATCH4,HAPLN2,BCAN,NES,CRABP2,ISG20L2,RRNAD1,MRPL24,HDGF,PRCC,SH2D2A,SH2D2A,NTRK1,NTRK1,INSRR,NTRK1,PEAR1,LRRC71,ARHGEF11,MIR765,ARHGEF11 | 0.584864 | 395.605 | 1060 | 661.105 |
| 2 | 16 | 67424160 | 68071394 | TPPP3,ZDHHC1,HSD11B2,ATP6V0D1,AGRP,RIPOR1,CTCF,CARMIL2,CARMIL2,ACD,ACD,PARD6A,ENKD1,C16orf86,GFOD2,RANBP10,TSNAXIP1,CENPT,CENPT,THAP11,NUTF2,EDC4,NRN1L,PSKH1,CTRL,PSMB10,LCAT,LCAT,SLC12A4,SLC12A4,DPEP3,DPEP2,DDX28,DDX28,DUS2 | 0.582836 | 394.934 | 303 | 201.643 |

| | | | | | | | | |
|---|---|---|---|---|---|---|---|---|
| 2 | 2 | 96652782 | 97779654 | ANKRD36C,FAHD2CP,FAHD2CP,GPAT2,GPAT2,ADRA2B,ASTL,DUSP2,STARD7,STARD7,STARD7-AS1,STARD7-AS1,TMEM127,CIAO1,SNRNP200,ITPRIPL1,NCAPH,NEURL3,ARID5A,KANSL3,FER1L5,LMAN2L,CNNM4,CNNM4,MIR3127,CNNM3,ANKRD23,ANKRD39,SEMA4C,FAM178B,FAM178B,LOC101927053,FAHD2B,ANKRD36 | 0.577834 | 377.74 | 363 | 222.434 |
| 2 | 1 | 1.6E+08 | 160106160 | OR10J5,APCS,CRP,DUSP23,FCRL6,SLAMF8,SLAMF8,SNHG28,SNHG28,SNHG28,VSIG8,VSIG8,CFAP45,CFAP45,MIR4259,TAGLN2,IGSF9,SLAMF9,LINC01133,PIGM,KCNJ10,KCNJ9,IGSF8,ATP1A2 | 0.567198 | 379.651 | 149 | 93.8069 |
| 2 | 1 | 10500 | 3739899 | FAM87B,LINC00115,LINC01128,SAMD11,SAMD11,NOC2L,NOC2L,KLHL17,PLEKHN1,PERM1,HES4,ISG15,AGRN,RNF223,C1orf159,LINC01342,MIR200B,MIR200A,MIR429,TTLL10,TNFRSF18,TNFRSF4,SDF4,B3GALT6,C1QTNF12,UBE2J2,SCNN1D,ACAP3,ACAP3,MIR6726,PUSL1,PUSL1,INTS11,INTS11,INTS11,MIR6727,CPTP,TAS1R3,DVL1,DVL1,MIR6808,MXRA8,AURKAIP1,CCNL2,LOC148413,MRPL20,ANKRD65,TMEM88B,VWA1,ATAD3C,ATAD3B,ATAD3A,TMEM240,SSU72,FNDC10,LOC105378586,MIB2,MMP23B,MMP23B,MMP23A,CDK11B,CDK11B,SLC35E2B,CDK11B,MMP23A,CDK11B,CDK11A,SLC35E2,NADK,GNB1,CALML6,TMEM52,CFAP74,GABRD,PRKCZ,PRKCZ,PRKCZ-AS1,PRKCZ,FAAP20,FAAP20,SKI,MORN1,RER1,PEX10,PLCH2,PANK4,HES5,TNFRSF14-AS1,TNFRSF14,TNFRSF14,FAM213B,FAM213B,MMEL1,MMEL1,TTC34,ACTRT2,LINC00982,PRDM16,PRDM16,MIR4251,ARHGEF16,MEGF6,MEGF6,MIR551A,TPRG1L,WRAP73,TP73,TP73-AS1,CCDC27,SMIM1,LRRC47,CEP104 | 0.562111 | 374.333 | 1026 | 615.492 |
| 2 | 1 | 1.61E+08 | 161551393 | F11R,TSTD1,USF1,ARHGAP30,NECTIN4,KLHDC9,PFDN2,NIT1,NIT1,DEDD,DEDD,UFC1,USP21,PPOX,B4GALT3,ADAMTS4,NDUFS2,FCER1G,APOA2,TOMM40L,TOMM40L,MIR5187,TOMM40L,NR1I3,NR1I3,PCP4L1,MPZ,SDHC,CFAP126,FCGR2A,HSPA6,FCGR3A,FCGR2C | 0.559893 | 393.264 | 220 | 136.556 |
| 2 | 8 | 1.26E+08 | 130853848 | TATDN1,NDUFB9,NDUFB9,MTSS1,MIR4662B,MIR4662A,ZNF572,SQLE,WASHC5,NSMCE2,TRIB1,FAM84B,PRNCR1,CASC8,POU5F1B,MYC,PVT1,MIR1204,PVT1,TMEM75,PVT1,MIR1205,PVT1,MIR1206,PVT1,MIR1207,MIR1208,CCDC26,MIR3686,GSDMC | 0.549511 | 350.906 | 179 | 102.936 |
| 2 | 15 | 43813472 | 43821725 | MAP1A | 0.54858 | 333.86 | 31 | 22.0282 |
| 2 | 1 | 2.28E+08 | 229384918 | SNAP47,JMJD4,SNAP47,PRSS38,WNT9A,WNT9A,MIR5008,WNT3A,ARF1,MIR3620,ARF1,C1orf35,MRPL55,GUK1,GJC2,IBA57-AS1,IBA57,OBSCN-AS1,OBSCN-AS1,OBSCN,OBSCN,TRIM11,TRIM11,MIR6742,TRIM17,HIST3H3,HIST3H2A,HIST3H2BB,MIR4666A,RNF187,BTNL10,RHOU,DUSP5P1,RHOU | 0.547505 | 404.771 | 295 | 184.041 |
| 2 | 8 | 77616293 | 77912237 | ZFHX4,PEX2 | 0.544218 | 424.612 | 58 | 40.3423 |
| 2 | 8 | 1.42E+08 | 142199222 | PTK2,DENND3 | 0.53025 | 377.465 | 33 | 19.3563 |
| 2 | 2 | 1.28E+08 | 128477974 | IWS1,MYO7B,MYO7B,LOC105373609,LIMS2,LIMS2,GPR17,SFT2D3,WDR33 | 0.524505 | 404.771 | 96 | 60.5602 |
| 2 | 10 | 73976515 | 74127484 | ASCC1,ANAPC16,ANAPC16,DDIT4,DNAJB12 | 0.513376 | 313.42 | 23 | 13.2916 |

| | | | | | | | | |
|---|---|---|---|---|---|---|---|---|
| 2 | 8 | 22398153 | 22674588 | PPP3CC,SORBS3,PDLIM2,C8orf58,CCAR2,BIN3,EGR3,PEBP4 | 0.510632 | 320.883 | 94 | 58.3739 |
| 2 | 16 | 66861886 | 67037262 | NAE1,CA7,PDP2,CDH16,RRAD,FAM96B,CES2,CES3,CES4A | 0.500199 | 401.908 | 78 | 49.8309 |
| 2 | 17 | 47921309 | 48801396 | TAC4,TAC4,FLJ45513,DLX4,DLX3,ITGA3,PDK2,SAMD14,PPP1R9B,SGCA,SGCA,HILS1,COL1A1,TMEM92,XYLT2,MRPL27,MRPL27,EME1,EME1,LRRC59,ACSF2,ACSF2,CHAD,RSAD1,MYCBPAP,EPN3,EPN3,LOC105371824,SPATA20,CACNA1G-AS1,CACNA1G,CACNA1G,ABCC3,ANKRD40,LUC7L3 | 0.499143 | 392.204 | 361 | 228.401 |
| 2 | 8 | 1.34E+08 | 141745591 | PHF20L1,TG,TG,SLA,WISP1,NDRG1,ST3GAL1,ZFAT,ZFAT,ZFAT-AS1,MIR30B,MIR30D,LINC01591,KHDRBS3,FAM135B,COL22A1,KCNK9,TRAPPC9,CHRAC1,AGO2,PTK2 | 0.498178 | 328.302 | 435 | 247.793 |
| 2 | 19 | 12662090 | 13316871 | ZNF564,ZNF490,ZNF791,MAN2B1,WDR83,WDR83OS,WDR83,WDR83,DHPS,DHPS,GNG14,FBXW9,TNPO2,TNPO2,SNORD135,TNPO2,SNORD41,TRIR,ASNA1,BEST2,HOOK2,JUNB,PRDX2,RNASEH2A,RTBDN,MAST1,MAST1,MIR6794,DNASE2,KLF1,GCDH,GCDH,SYCE2,SYCE2,FARSA,FARSA,FARSA-AS1,CALR,CALR,MIR6515,RAD23A,GADD45GIP1,DAND5,NFIX,LYL1,TRMT1,NACC1,STX10,IER2 | 0.49247 | 381.314 | 313 | 198.034 |
| 2 | 22 | 46240097 | 51244066 | WNT7B,LOC730668,PRR34,PRR34,PRR34-AS1,MIRLET7BHG,MIR3619,MIRLET7BHG,MIRLET7BHG,MIRLET7A3,MIRLET7BHG,MIR4763,MIRLET7B,PPARA,CDPF1,PKDREJ,TTC38,GTSE1,TRMU,CELSR1,GRAMD4,CERK,TBC1D22A,TBC1D22A,TBC1D22A-AS1,LINC01644,LINC00898,MIR3201,FAM19A5,FAM19A5,LOC284933,MIR4535,C22orf34,C22orf34,MIR3667,BRD1,ZBED4,ALG12,CRELD2,PIM3,PIM3,MIR6821,IL17REL,TTLL8,MLC1,MOV10L1,PANX2,TRABD,SELENOO,SELENOO,TUBGCP6,TUBGCP6,HDAC10,MAPK12,MAPK11,PLXNB2,DENND6B,PPP6R2,SBF1,ADM2,MIOX,LMF2,NCAPH2,NCAPH2,SCO2,SCO2,TYMP,TYMP,ODF3B,KLHDC7B,SYCE3,CPT1B,CHKB-CPT1B,CHKB-CPT1B,CHKB,MAPK8IP2,ARSA,SHANK3,ACR,RPL23AP82,RABL2B | 0.484152 | 357.496 | 839 | 520.446 |

| | | | | | | | |
|---|---|---|---|---|---|---|---|
| 2 | 16 | 1918690 | 3340564 | HS3ST6,MSRB1,RPL3L,NDUFB10,RPS2,RPS2,SNORA10,RPS2,SNORA64,SNHG9,SNORA78,RNF151,TBL3,NOXO1,GFER,SYNGR3,ZNF598,NPW,SLC9A3R2,NTHL1,NTHL1,TSC2,TSC2,PKD1,PKD1,MIR1225,PKD1,LOC105371049,RAB26,SNHG19,SNORD60,TRAF7,TRAF7,CASKIN1,CASKIN1,MLST8,MLST8,BRICD5,BRICD5,PGP,E4F1,DNASE1L2,ECI1,RNPS1,LOC106660606,MIR3677,LOC106660606,MIR940,MIR4717,ABCA3,CCNF,CCNF,MIR6767,TEDC2,TEDC2,MIR6768,NTN3,TBC1D24,ATP6V0C,AMDHD2,AMDHD2,CEMP1,MIR3178,PDPK1,LOC652276,FLJ42627,ERVK13-1,KCTD5,PRSS27,SRRM2-AS1,SRRM2,SRRM2,ELOB,ELOB,PRSS33,PRSS41,PRSS21,ZG16B,PRSS30P,PRSS22,FLYWCH2,FLYWCH1,KREMEN2,PAQR4,PAQR4,PKMYT1,PKMYT1,CLDN9,CLDN6,TNFRSF12A,HCFC1R1,HCFC1R1,THOC6,THOC6,BICDL2,BICDL2,LOC100128770,LOC100128770,MMP25,MMP25,MMP25-AS1,IL32,ZSCAN10,ZNF205-AS1,ZNF205-AS1,ZNF205,ZNF205,ZNF213,CASP16P,OR1F1,OR1F2P,ZNF200,MEFV,ZNF263 | 0.479597 | 371.434 | 718 | 445.714 |
| 2 | 4 | 5981939 | 10529666 | JAKMIP1,WFS1,PPP2R2C,MAN2B2,MRFAP1,LOC93622,S100P,MRFAP1L1,BLOC1S4,KIAA0232,TBC1D14,LOC100129931,CCDC96,LOC100129931,TADA2B,TADA2B,GRPEL1,SORCS2,SORCS2,MIR4798,SORCS2,PSAPL1,SORCS2,MIR4274,AFAP1-AS1,AFAP1,AFAP1,AFAP1,LOC389199,ABLIM2,ABLIM2,MIR95,SH3TC1,HTRA3,ACOX3,TRMT44,GPR78,CPZ,HMX1,USP17L6P,DRD5,SLC2A9,WDR1,WDR1,MIR3138,ZNF518B,CLNK | 0.473714 | 335.662 | 464 | 277.575 |
| 2 | 11 | 67023663 | 67816730 | GRK2,ANKRD13D,SSH3,LOC100130987,POLD4,LOC100130987,CLCF1,RAD9A,PPP1CA,TBC1D10C,CARNS1,RPS6KB2,PTPRCAP,CORO1B,GPR152,GPR152,CABP4,CABP4,TMEM134,AIP,AIP,MIR6752,PITPNM1,CDK2AP2,CABP2,GSTP1,C11orf72,NDUFV1,NUDT8,TBX10,ACY3,ALDH3B2,FAM86C2P,UNC93B1,ALDH3B1,NDUFS8,NDUFS8,MIR7113,NDUFS8,MIR4691,TCIRG1,TCIRG1,MIR6753 | 0.47001 | 337.872 | 273 | 173.207 |
| 2 | 3 | 49028254 | 49226806 | P4HTM,WDR6,WDR6,DALRD3,DALRD3,DALRD3,MIR425,DALRD3,NDUFAF3,MIR191,NDUFAF3,IMPDH2,QRICH1,QARS,QARS,MIR6890,USP19,LAMB2,CCDC71,KLHDC8B,C3orf84 | 0.465322 | 349.435 | 162 | 112.25 |
| 2 | 22 | 39112728 | 40037243 | GTPBP1,SUN2,DNAL4,NPTXR,CBX6,APOBEC3A,APOBEC3A_B,APOBEC3A_B,APOBEC3A_B,APOBEC3B,APOBEC3A_B,APOBEC3B,APOBEC3B-AS1,APOBEC3C,APOBEC3D,APOBEC3F,APOBEC3G,APOBEC3H,CBX7,PDGFB,RPL3,RPL3,SNORD83B,RPL3,SNORD83A,RPL3,SNORD139,RPL3,SNORD43,SYNGR1,TAB1,TAB1,LOC100506472,MGAT3,MIEF1,ATF4,RPS19BP1,CACNA1I | 0.464995 | 343.632 | 228 | 127.228 |
| 2 | 1 | 43738490 | 44121476 | TMEM125,C1orf210,TIE1,MPL,CDC20,ELOVL1,ELOVL1,MIR6734,MED8,SZT2,SZT2,SZT2-AS1,SZT2,SZT2-AS1,MIR6735,SZT2,HYI,PTPRF,KDM4A | 0.463197 | 391.663 | 188 | 123.93 |
| 2 | 17 | 27844527 | 27993323 | TAOK1,ABHD15,TP53I13,GIT1,ANKRD13B,CORO6,SSH2 | 0.456004 | 343.77 | 80 | 53.8515 |

| | | | | | | | | |
|---|---|---|---|---|---|---|---|---|
| 2 | 7 | 74167958 | 76883192 | NCF1,GTF2IRD2,RCC1L,GTF2IRD2,GTF2IRD2B,NCF1C,GTF2IP4,GTF2IP1,GTF2IP1,PMS2P5,GTF2IP1,PMS2P5,CASTOR2,TRIM73,NSUN5P1,NSUN5P1,POM121C,POM121C,PMS2P3,HIP1,CCL26,CCL24,RHBDD2,POR,MIR4651,POR,SNORA14A,POR,TMEM120A,STYXL1,MDH2,SRRM3,HSPB1,YWHAG,SSC4D,SSC4D,ZP3,ZP3,DTX2,UPK3B,LOC100133091,POMZP3,DTX2P1-UPK3BP1-PMS2P11,FAM185BP,CCDC146,CCDC146,FGL2 | 0.455657 | 306.007 | 369 | 195.892 |
| 2 | 1 | 2.03E+08 | 203666783 | TMEM183A,TMEM183B,PPFIA4,MYOG,ADORA1,MYBPH,CHI3L1,CHIT1,BTG2,FMOD,PRELP,OPTC,ATP2B4 | 0.451441 | 345.017 | 116 | 70.9008 |
| 2 | 7 | 99516623 | 102390089 | TRIM4,GJC3,AZGP1,AZGP1P1,ZKSCAN1,ZSCAN21,ZSCAN21,ZNF3,ZNF3,COPS6,MCM7,MCM7,MIR25,MIR93,MCM7,MIR106B,MCM7,AP4M1,AP4M1,TAF6,TAF6,CNPY4,CNPY4,MBLAC1,LAMTOR4,C7orf43,C7orf43,MIR4658,GAL3ST4,GPC2,STAG3,STAG3,GATS,GATS,GATS,PVRIG,SPDYE3,STAG3L5P-PVRIG2P-PILRB,PVRIG2P,STAG3L5P-PVRIG2P-PILRB,MIR6840,STAG3L5P-PVRIG2P-PILRB,PILRB,PILRA,ZCWPW1,MEPCE,PPP1R35,C7orf61,TSC22D4,NYAP1,AGFG2,SAP25,LRCH4,ZASP,LRCH4,FBXO24,FBXO24,PCOLCE-AS1,PCOLCE-AS1,PCOLCE,PCOLCE,MOSPD3,TFR2,ACTL6B,GNB2,GIGYF1,POP7,EPO,ZAN,EPHB4,SLC12A9,TRIP6,TRIP6,MIR6875,SRRT,SRRT,UFSP1,UFSP1,ACHE,MUC3A,MUC12,MUC12,LOC102724094,MUC17,TRIM56,SERPINE1,AP1S1,AP1S1,MIR4653,VGF,NAT16,MOGAT3,PLOD3,ZNHIT1,CLDN15,FIS1,LOC101927746,IFT22,COL26A1,MYL10,CUX1,SH2B2,SH2B2,MIR4285,LOC100289561,LOC100630923,LOC100630923,LOC100630923,PRKRIP1,LOC100630923,PRKRIP1,MIR548O,ORAI2,ALKBH4,LRWD1,LRWD1,MIR5090,LRWD1,MIR4467,POLR2J,RASA4B,POLR2J3,RASA4,RASA4B,POLR2J2,UPK3BL1,POLR2J2,RASA4DP,FAM185A | 0.450605 | 385.746 | 902 | 542.793 |
| 2 | 1 | 44365198 | 45811281 | ST3GAL3,ARTN,IPO13,DPH2,ATP6V0B,B4GALT2,CCDC24,SLC6A9,KLF17,DMAP1,ERI3,RNF220,RNF220,MIR5584,TMEM53,C1orf228,KIF2C,KIF2C,SNORD160,RPS8,RPS8,SNORD55,RPS8,SNORD46,RPS8,SNORD38A,RPS8,SNORD38B,BEST4,PLK3,PLK3,TCTEX1D4,TCTEX1D4,BTBD19,PTCH2,EIF2B3,HECTD3,UROD,ZSWIM5,HPDL,MUTYH,MUTYH,TOE1,TOE1,TESK2 | 0.449978 | 356.533 | 336 | 212.38 |
| 2 | 7 | 44887455 | 45931680 | H2AFV,PURB,MIR4657,PURB,MYO1G,SNHG15,SNHG15,SNORA9,CCM2,NACAD,TBRG4,TBRG4,SNORA5A,TBRG4,SNORA5C,TBRG4,SNORA5B,RAMP3,ADCY1,SEPT7P2,IGFBP1 | 0.448753 | 331.879 | 129 | 75.5915 |
| 2 | 1 | 1.62E+08 | 162470887 | ATF6,OLFML2B,NOS1AP,NOS1AP,MIR4654,NOS1AP,MIR556,SPATA46,C1orf226,SH2D1B,UHMK1 | 0.447084 | 365.304 | 59 | 34.0887 |
| 2 | 2 | 74618315 | 74785959 | C2orf81,WDR54,RTKN,INO80B,INO80B-WBP1,INO80B-WBP1,WBP1,MOGS,MRPL53,CCDC142,CCDC142,TTC31,TTC31,LBX2,LBX2,LBX2-AS1,PCGF1,TLX2,DQX1,AUP1,AUP1,HTRA2,HTRA2,HTRA2,LOXL3,LOXL3,LOXL3,DOK1,DOK1,M1AP | 0.446432 | 358.516 | 154 | 102.415 |
| 2 | 7 | 1.44E+08 | 144150036 | ARHGEF5,NOBOX | 0.445205 | 322.801 | 17 | 10.4931 |

| | | | | | | | | |
|---|---|---|---|---|---|---|---|---|
| 2 | 16 | 60500 | 1884403 | POLR3K,SNRNP25,RHBDF1,MPG,MPG,NPRL3,NPRL3,HBZ,HBM,HBA2,HBA1,HBQ1,LUC7L,FAM234A,FAM234A,RGS11,RGS11,ARHGDIG,PDIA2,AXIN1,MRPL28,TMEM8A,LOC100134368,NME4,DECR2,RAB11FIP3,CAPN15,MIR5587,CAPN15,MIR3176,CAPN15,PRR35,NHLRC4,PIGQ,RAB40C,WFIKKN1,METTL26,LOC100287175,MCRIP2,LOC105371038,WDR90,WDR90,RHOT2,RHBDL1,STUB1,STUB1,JMJD8,JMJD8,WDR24,FBXL16,METRN,FAM173A,FAM173A,CCDC78,CCDC78,HAGHL,NARFL,MSLN,MIR662,RPUSD1,CHTF18,GNG13,PRR25,LMF1,LMF1,LMF1-AS1,SOX8,SSTR5-AS1,SSTR5,C1QTNF8,CACNA1H,CACNA1H,TPSG1,TPSG1,TPSB2,TPSAB1,TPSD1,UBE2I,BAIAP3,BAIAP3,TSR3,TSR3,GNPTG,UNKL,C16orf91,CCDC154,CLCN7,PTX4,TELO2,IFT140,IFT140,TMEM204,IFT140,LOC105371046,CRAMP1,JPT2,MAPK8IP3,MAPK8IP3,MIR3177,NME3,MRPS34,MRPS34,EME2,EME2,SPSB3,NUBP2,IGFALS,HAGH,FAHD1,FAHD1,MEIOB | 0.442979 | 362.119 | 951 | 603.771 |
| 2 | 10 | 1.05E+08 | 105648969 | PDCD11,CALHM2,CALHM1,CALHM3,NEURL1-AS1,NEURL1,NEURL1,SH3PXD2A,STN1 | 0.440283 | 331.205 | 72 | 44.0656 |
| 2 | 9 | 95274304 | 97190120 | CENPP,ECM2,CENPP,ECM2,MIR4670,CENPP,CENPP,IPPK,IPPK,IPPK,LOC100128361,BICD2,ANKRD19P,ZNF484,FGD3,FGD3,LOC101927954,SUSD3,CARD19,NINJ1,WNK2,WNK2,C9orf129,C9orf129,FAM120AOS,FAM120AOS,FAM120A,FAM120A,PHF2,PHF2,MIR548AU,MIR4291,BARX1,PTPDC1,MIRLET7A1,MIRLET7F1,MIRLET7DHG,MIRLET7D,ZNF169,NUTM2F,MFSD14B | 0.431111 | 345.325 | 286 | 165.845 |
| 2 | 3 | 50155700 | 50712039 | RBM5,SEMA3F-AS1,SEMA3F-AS1,SEMA3F,SEMA3F,GNAT1,SLC38A3,GNAI2,SEMA3B,SEMA3B,MIR6872,LSMEM2,IFRD2,HYAL3,HYAL3,NAT6,HYAL1,HYAL2,TUSC2,RASSF1,RASSF1,RASSF1-AS1,ZMYND10,NPRL2,NPRL2,CYB561D2,CYB561D2,CYB561D2,TMEM115,CYB561D2,CACNA2D2,CACNA2D2,C3orf18,HEMK1,CISH,MAPKAPK3 | 0.427001 | 329.245 | 221 | 141.515 |
| 2 | 2 | 1.77E+08 | 177135160 | LNPK,EVX2,HOXD13,HOXD12,HOXD11,HOXD10,HOXD9,HOXD8,MIR10B,HOXD4,HOXD3,HAGLR,HOXD1,MIR7704,HOXD1,MTX2 | 0.425659 | 356.889 | 58 | 35.9112 |
| 2 | 3 | 49413546 | 49999492 | TCTA,AMT,AMT,NICN1,NICN1,DAG1,BSN,APEH,MST1,RNF123,RNF123,AMIGO3,RNF123,GMPPB,GMPPB,IP6K1,CDHR4,FAM212A,UBA7,UBA7,MIR5193,TRAIP,CAMKV,MST1R,MON1A,RBM6 | 0.42218 | 346.499 | 282 | 188.068 |
| 2 | X | 47920160 | 49157257 | ZNF630-AS1,ZNF630,ZNF630,SSX6,SPACA5,SPACA5B,SSX5,SSX1,SSX9,SSX3,SSX4B,SSX4,SSX4,SSX4B,SLC38A5,FTSJ1,PORCN,EBP,TBC1D25,RBM3,WDR13,WAS,SUV39H1,GLOD5,GATA1,HDAC6,ERAS,PCSK1N,TIMM17B,PQBP1,SLC35A2,PIM2,OTUD5,KCND1,GRIPAP1,TFE3,CCDC120,PRAF2,WDR45,GPKOW,MAGIX,PLP2,PRICKLE3,SYP,SYP,SYP-AS1,CACNA1F,CCDC22,CCDC22,FOXP3,FOXP3,PPP1R3F | 0.418114 | 360.628 | 441 | 272.729 |

| | | | | | | | | |
|---|---|---|---|---|---|---|---|---|
| 2 | 19 | 9875568 | 11728516 | ZNF846,FBXL12,UBL5,PIN1,OLFM2,COL5A3,RDH8,MIR5589,C3P1,C19orf66,C19orf66,ANGPTL6,ANGPTL6,PPAN,PPAN-P2RY11,PPAN,PPAN-P2RY11,SNORD105,PPAN,PPAN-P2RY11,SNORD105B,PPAN,PPAN-P2RY11,P2RY11,PPAN-P2RY11,P2RY11,PPAN-P2RY11,P2RY11,EIF3G,EIF3G,DNMT1,S1PR2,S1PR2,MIR4322,MRPL4,ICAM1,ICAM4,ICAM5,ZGLP1,FDX1L,RAVER1,ICAM3,TYK2,CDC37,CDC37,MIR1181,PDE4A,KEAP1,S1PR5,ATG4D,ATG4D,MIR1238,ATG4D,KRI1,KRI1,CDKN2D,AP1M2,SLC44A2,ILF3,QTRT1,DNM2,DNM2,MIR638,DNM2,MIR4748,DNM2,MIR199A1,DNM2,MIR6793,TMED1,C19orf38,CARM1,YIPF2,YIPF2,TIMM29,TIMM29,SMARCA4,LDLR,LDLR,MIR6886,SPC24,KANK2,DOCK6,DOCK6,LOC105372273,DOCK6,ANGPTL8,TSPAN16,TSPAN16,RAB3D,RAB3D,TMEM205,CCDC159,PLPPR2,SWSAP1,EPOR,RGL3,CCDC151,CCDC151,PRKCSH,PRKCSH,ELAVL3,ZNF653,ECSIT,CNN1,ELOF1,ACP5,ZNF627 | 0.417924 | 357.422 | 804 | 502.841 |
| 2 | 16 | 3458407 | 5122156 | ZNF174,ZNF597,NAA60,NAA60,MIR6126,C16orf90,CLUAP1,NLRC3,SLX4,DNASE1,TRAP1,CREBBP,ADCY9,SRL,TFAP4,GLIS2,PAM16,CORO7-PAM16,CORO7-PAM16,CORO7,CORO7-PAM16,CORO7,VASN,DNAJA3,NMRAL1,HMOX2,CDIP1,C16orf96,UBALD1,MGRN1,MGRN1,MIR6769A,NUDT16L1,ANKS3,C16orf71,C16orf71,ZNF500,ZNF500,SEPT12,SMIM22,ROGDI,GLYR1,UBN1,PPL,SEC14L5,NAGPA,NAGPA,NAGPA-AS1,C16orf89,ALG1 | 0.416106 | 336.975 | 521 | 330.64 |
| 2 | 8 | 1.31E+08 | 132982882 | FAM49B,FAM49B,MIR5194,ASAP1,ADCY8,EFR3A | 0.41388 | 290.01 | 104 | 55.6795 |
| 2 | 7 | 1.49E+08 | 151039035 | EZH2,PDIA4,ZNF786,ZNF425,ZNF398,ZNF282,ZNF212,ZNF783,LOC155060,ZNF777,ZNF746,ZNF767P,KRBA1,ZNF467,SSPO,ZNF862,ATP6V0E2,ACTR3C,ACTR3C,LRRC61,LRRC61,ZBED6CL,LRRC61,RARRES2,REPIN1,ZNF775,GIMAP8,GIMAP7,GIMAP4,GIMAP6,GIMAP2,GIMAP1,GIMAP1-GIMAP5,GIMAP1-GIMAP5,GIMAP5,TMEM176B,TMEM176A,AOC1,KCNH2,NOS3,NOS3,ATG9B,ATG9B,ABCB8,ASIC3,CDK5,SLC4A2,FASTK,TMUB1,AGAP3,GBX1,ASB10,ABCF2,CHPF2,CHPF2,MIR671,SMARCD3,NUB1 | 0.411603 | 337.431 | 651 | 411.98 |
| 2 | 7 | 43847408 | 44836499 | MRPS24,URGCP-MRPS24,URGCP-MRPS24,URGCP,UBE2D4,UBE2D4,POLR2J4,POLR2J4,RASA4CP,LINC00957,DBNL,PGAM2,POLM,MIR6838,POLM,AEBP1,AEBP1,MIR4649,POLD2,MYL7,GCK,YKT6,CAMK2B,NUDCD3,NPC1L1,DDX56,TMED4,OGDH,ZMIZ2,PPIA | 0.406889 | 355.479 | 280 | 169.149 |
| 2 | 15 | 91347420 | 91503222 | BLM,FURIN,FES,MAN2A2,UNC45A,HDDC3,UNC45A,RCCD1 | 0.402402 | 339.902 | 91 | 60.0498 |
| 2 | 16 | 15501646 | 18793918 | MPV17L,C16orf45,MARF1,MARF1,MIR6506,NDE1,MIR484,NDE1,NDE1,MYH11,MYH11,FOPNL,ABCC1,ABCC6,NOMO3,PKD1P4-NPIPA8,XYLT1,XYLT1,LOC102723692,PKD1P5-LOC105376752,NOMO2,ABCC6P1 | 0.402249 | 321.69 | 257 | 147.664 |
| 2 | 1 | 1.8E+08 | 180283208 | CEP350,QSOX1,FLJ23867,LHX4,LHX4,LHX4-AS1,ACBD6 | 0.395225 | 264.499 | 37 | 20.3477 |
| 2 | 17 | 38573987 | 38784450 | TOP2A,IGFBP4,TNS4,CCR7 | 0.394015 | 312.83 | 30 | 18.783 |

| | | | | | | | | |
|---|---|---|---|---|---|---|---|---|
| 2 | 20 | 44451931 | 45000614 | TNNC2,SNX21,SNX21,ACOT8,ACOT8,ZSWIM3,ZSWIM1,SPATA25,NEURL2,NEURL2,CTSA,CTSA,PLTP,PCIF1,ZNF335,MMP9,MMP9,SLC12A5-AS1,SLC12A5-AS1,SLC12A5,SLC12A5,NCOA5,CD40,CDH22,SLC35C2,ELMO2 | 0.393809 | 344.643 | 208 | 136.592 |
| 2 | 15 | 78888944 | 79168994 | CHRNA3,CHRNB4,LOC646938,ADAMTS7,MORF4L1 | 0.389793 | 256.869 | 53 | 27.0393 |
| 2 | 1 | 2.47E+08 | 249240121 | ZNF669,C1orf229,ZNF124,MIR3916,VN1R5,ZNF496,NLRP3,OR2B11,OR2W5,GCSAML,GCSAML-AS1,GCSAML,OR2C3,GCSAML,OR2G2,OR2G3,OR13G1,OR6F1,OR14A2,OR14A2,OR14K1,OR1C1,OR14A16,OR11L1,TRIM58,OR2W3,OR2T8,OR2AJ1,OR2L13,OR2L8,OR2L13,OR2AK2,OR2L13,OR2L1P,OR2L13,OR2L13,OR2L5,OR2L13,OR2L2,OR2L13,OR2L3,OR2M1P,OR2M5,OR2M2,OR2M3,OR2M4,OR2T33,OR2T12,OR2M7,OR14C36,OR2T4,OR2T6,OR2T1,OR2T7,OR2T2,OR2T3,OR2T5,OR2G6,OR2T29,OR2T34,OR2T10,OR2T11,OR2T35,OR2T27,OR14I1,LYPD8,SH3BP5L,MIR3124,ZNF672,ZNF692,PGBD2 | 0.389198 | 414.499 | 330 | 187.692 |
| 2 | 9 | 88967539 | 95048136 | ZCCHC6,GAS1,C9orf170,DAPK1,CTSL,CTSL3P,CTSLP8,LOC392364,LOC497256,SPATA31E1,LOC497256,SPATA31C1,CDK20,SPATA31C2,SPIN1,NXNL2,MIR4289,C9orf47,C9orf47,S1PR3,S1PR3,SHC3,CKS2,CKS2,MIR3153,SECISBP2,SEMA4D,GADD45G,MIR4290HG,MIR4290,DIRAS2,SYK,AUH,NFIL3,MIR3910-1,MIR3910-2,ROR2,SPTLC1,LINC00475,PRSS47,IARS | 0.387356 | 354.892 | 373 | 219.634 |
| 2 | 11 | 68678895 | 70118490 | IGHMBP2,MRGPRD,MRGPRF,TPCN2,TPCN2,MIR3164,MYEOV,CCND1,ORAOV1,FGF19,FGF4,FGF3,ANO1,FADD,PPFIA1 | 0.387001 | 313.43 | 136 | 82.8756 |
| 2 | 16 | 31193579 | 31580552 | FUS,PYCARD,PYCARD,PYCARD-AS1,TRIM72,TRIM72,PYDC1,ITGAM,ITGAX,ITGAD,COX6A2,ZNF843,ARMC5,TGFB1I1,SLC5A2,SLC5A2,C16orf58,C16orf58,AHSP,FRG2KP,YBX3P1 | 0.384694 | 375.629 | 184 | 113.907 |
| 2 | 22 | 45809322 | 46085807 | SMC1B,RIBC2,FBLN1,ATXN10 | 0.379644 | 336.3 | 40 | 24.1118 |

| | | | | | | | | |
|---|---|---|---|---|---|---|---|---|
| 2 | 9 | 1.35E+08 | 141152931 | TTF1,CFAP77,BARHL1,DDX31,DDX31,GTF3C4,GTF3C4,AK8,SPACA9,TSC1,GFI1B,MIR548AW,GFI1B,GTF3C5,GTF3C5,MIR6877,CEL,CELP,RALGDS,GBGT1,OBP2B,ABO,SURF6,MED22,MED22,RPL7A,RPL7A,RPL7A,SNORD24,RPL7A,SNORD36B,RPL7A,SNORD36A,RPL7A,SNORD36C,SURF1,SURF1,SURF2,SURF2,SURF4,SURF4,STKLD1,STKLD1,REXO4,REXO4,ADAMTS13,ADAMTS13,CACFD1,SLC2A6,MYMK,ADAMTSL2,FAM163B,DBH,DBH,DBH-AS1,SARDH,VAV2,BRD3,WDR5,RXRA,MIR4669,RXRA,COL5A1,COL5A1,COL5A1-AS1,COL5A1,LOC101448202,LOC101448202,MIR3689A,MIR3689B,MIR3689D2,LOC101448202,MIR3689A,MIR3689E,MIR3689F,FCN2,FCN1,OLFM1,C9orf62,PPP1R26,C9orf116,MRPS2,MRPS2,LOC101928525,LCN1,OBP2A,PAEP,GLT6D1,LCN9,SOHLH1,KCNT1,CAMSAP1,UBAC1,NACC2,TMEM250,LHX3,QSOX2,DKFZP434A062,GPSM1,DNLZ,CARD9,SNAPC4,SDCCAG3,PMPCA,INPP5E,SEC16A,C9orf163,NOTCH1,NOTCH1,MIR4673,MIR4674,EGFL7,EGFL7,MIR126,AGPAT2,FAM69B,SNHG7,SNORA17B,SNHG7,SNORA17A,LCN10,LCN6,LCN6,LOC100128593,LCN8,LCN15,TMEM141,CCDC183,CCDC183,CCDC183-AS1,CCDC183-AS1,RABL6,RABL6,RABL6,MIR4292,AJM1,PHPT1,MAMDC4,EDF1,TRAF2,TRAF2,MIR4479,FBXW5,C8G,LCN12,PRR31,PTGDS,LCNL1,PAXX,CLIC3,ABCA2,ABCA2,C9orf139,C9orf139,FUT7,C9orf139,NPDC1,ENTPD2,SAPCD2,UAP1L1,MAN1B1-AS1,MAN1B1,DPP7,GRIN1,LRRC26,LRRC26,MIR3621,TMEM210,ANAPC2,SSNA1,TPRN,TMEM203,NDOR1,RNF208,CYSRT1,RNF224,SLC34A3,TUBB4B,TUBB4B,FAM166A,FAM166A,STPG3-AS1,STPG3,NELFB,TOR4A,NRARP,EXD3,NOXA1,NOXA1,ENTPD8,ENTPD8,NSMF,NSMF,MIR7114,PNPLA7,MRP | 0.379485 | 338.364 | 1715 | 1054.57 |
| 2 | 11 | 66583514 | 66888245 | C11orf80,C11orf80,RCE1,RCE1,PC,PC,LRFN4,PC,MIR3163,C11orf86,SYT12,SYT12,MIR6860,RHOD | 0.376057 | 354.228 | 65 | 41.8628 |
| 2 | 3 | 48447049 | 48788130 | PLXNB1,CCDC51,TMA7,ATRIP,ATRIP,TREX1,TREX1,SHISA5,PFKFB4,PFKFB4,MIR6823,UCN2,COL7A1,COL7A1,MIR711,UQCRC1,TMEM89,SLC26A6,SLC26A6,MIR6824,CELSR3,CELSR3,MIR4793,CELSR3-AS1,NCKIPSD,IP6K2 | 0.375702 | 335.977 | 299 | 198.71 |
| 2 | 2 | 1.29E+08 | 133618186 | UGGT1,HS6ST1,RAB6C-AS1,RAB6C,RAB6C,CCDC74B,SMPD4,SMPD4,MZT2B,MZT2B,TUBA3E,CCDC115,CCDC115,IMP4,IMP4,PTPN18,CFC1B,TISP43,LOC646743,LOC646743,TISP43,CFC1,CFC1B,GPR148,AMER3,ARHGEF4,FAM168B,PLEKHB2,RAB6D,NOC2LP2,TUBA3D,MZT2A,MZT2A,MIR4784,LOC150776,CCDC74A,C2orf27A,C2orf27B,ANKRD30BL,MIR663B,ZNF806,GPR39,GPR39,LYPD1,LYPD1,NCKAP5 | 0.375143 | 286.339 | 363 | 198.691 |
| 2 | 10 | 98740511 | 99187694 | LCOR,SLIT1,SLIT1,ARHGAP19-SLIT1,ARHGAP19-SLIT1,ARHGAP19,FRAT1,FRAT2,RRP12,PGAM1 | 0.37463 | 308.969 | 127 | 76.7341 |

| | | | | | | | | |
|---|---|---|---|---|---|---|---|---|
| 2 | 15 | 73925460 | 76136903 | NPTN,CD276,C15orf59,TBC1D21,LOXL1-AS1,LOXL1,LOXL1,STOML1,PML,LOC283731,ISLR2,ISLR,STRA6,CCDC33,CYP11A1,SEMA7A,SEMA7A,MIR6881,UBL7,ARID3B,CLK3,EDC3,CYP1A1,CYP1A2,CSK,MIR4513,CSK,LMAN1L,CPLX3,ULK3,SCAMP2,MPI,FAM219B,COX5A,RPP25,SCAMP5,PPCDC,C15orf39,LOC105376731,COMMD4,NEIL1,NEIL1,MIR631,MAN2C1,SIN3A,PTPN9,SNUPN,IMP3,SNX33,CSPG4,ODF3L1,DNM1P35,MIR4313,UBE2Q2 | 0.373961 | 303.519 | 551 | 330.416 |
| 2 | 19 | 35448833 | 36229501 | ZNF792,GRAMD1A,SCN1B,HPN,HPN,HPN-AS1,HPN-AS1,FXYD3,LGI4,FXYD1,FXYD7,FXYD5,FAM187B,LSR,USF2,HAMP,MAG,CD22,CD22,MIR5196,FFAR1,FFAR3,GPR42,LINC01531,FFAR2,KRTDAP,DMKN,SBSN,GAPDHS,GAPDHS,TMEM147-AS1,TMEM147-AS1,TMEM147,TMEM147,ATP4A,HAUS5,RBM42,ETV2,COX6B1,UPK1A,UPK1A,UPK1A-AS1,ZBTB32,KMT2B | 0.371605 | 355.507 | 334 | 215.316 |
| 2 | 8 | 1.25E+08 | 125499850 | FAM91A1,FER1L6,FER1L6,FER1L6-AS1,FER1L6,FER1L6-AS2,TMEM65,TRMT12,RNF139 | 0.37149 | 367.766 | 74 | 44.9646 |
| 2 | 1 | 2.26E+08 | 226420319 | EPHX1,TMEM63A,LEFTY1,PYCR2,PYCR2,MIR6741,LEFTY2,SDE2,H3F3A,H3F3AP4,ACBD3,ACBD3,ACBD3-AS1,MIXL1,LIN9 | 0.368424 | 304.291 | 89 | 51.5441 |
| 2 | 9 | 1.16E+08 | 117379688 | RGS3,ZNF618,AMBP,KIF12,COL27A1,COL27A1,MIR455,ORM1,ORM2,AKNA,WHRN,ATP6V1G1,TMEM268 | 0.36414 | 320.491 | 220 | 133.762 |

| | | | | | | | | |
|---|---|---|---|---|---|---|---|---|
| 2 | 11 | 63533321 | 66512434 | C11orf95,C11orf84,MARK2,RCOR2,NAA40,COX8A,OTUB1,MACROD1,MACROD1,FLRT1,STIP1,FERMT3,FERMT3,TRPT1,TRPT1,NUDT22,DNAJC4,VEGFB,FKBP2,PPP1R14B,PLCB3,BAD,BAD,GPR137,GPR137,KCNK4,KCNK4-TEX40,KCNK4-TEX40,CATSPERZ,ESRRA,TRMT112,PRDX5,CCDC88B,RPS6KA4,RPS6KA4,MIR1237,LOC100996455,SLC22A11,SLC22A12,NRXN2,RASGRP2,PYGM,SF1,MAP4K2,MEN1,CDC42BPG,EHD1,MIR194-2HG,MIR194-2HG,MIR192,MIR194-2,ATG2A,ATG2A,MIR6750,ATG2A,MIR6749,PPP2R5B,GPHA2,MAJIN,BATF2,ARL2,ARL2-SNX15,ARL2,ARL2-SNX15,MIR6879,ARL2-SNX15,SNX15,SAC3D1,NAALADL1,CDCA5,ZFPL1,ZFPL1,TMEM262,TMEM262,VPS51,TM7SF2,ZNHIT2,FAU,MRPL49,SYVN1,SYVN1,MIR6751,SPDYC,LOC728975,LOC728975,CAPN1,CAPN1,SLC22A20P,POLA2,CDC42EP2,DPF2,TIGD3,SLC25A45,FRMD8,NEAT1,MIR612,MALAT1,MALAT1,TALAM1,MALAT1,TALAM1,MASCRNA,SCYL1,SCYL1,LTBP3,LTBP3,SSSCA1-AS1,SSSCA1,FAM89B,EHBP1L1,KCNK7,MAP3K11,PCNX3,PCNX3,MIR4690,SIPA1,SIPA1,MIR4489,RELA,KAT5,KAT5,RNASEH2C,RNASEH2C,AP5B1,OVOL1,SNX32,CFL1,MUS81,MUS81,EFEMP2,EFEMP2,CTSW,FIBP,CCDC85B,FOSL1,C11orf68,DRAP1,TSGA10IP,SART1,EIF1AD,BANF1,CST6,CATSPER1,GAL3ST3,SF3B2,PACS1,KLC2,RAB1B,CNIH2,YIF1A,TMEM151A,CD248,RIN1,BRMS1,B4GAT1,LOC102724064,SLC29A2,SLC29A2,NPAS4,MRPL11,PELI3,PELI3,LOC101928069,DPP3,BBS1,BBS1,ZDHHC24,ZDHHC24,ACTN3,CTSF,CCDC87,CCS,RBM14,RBM14-RBM4,RBM14-RBM4,RBM4,RBM4,RBM4,RBM4B,RBM4B,SPTBN2,C11orf80 | 0.362598 | 324.447 | 1487 | 945.484 |
| 2 | 16 | 27078117 | 29141445 | C16orf82,KDM8,NSMCE1,FLJ21408,IL4R,IL21R,IL21R,IL21R-AS1,GTF3C1,KIAA0556,KIAA0556,LOC100128079,GSG1L,XPO6,SBK1,EIF3CL,EIF3C,EIF3C,CLN3,APOBR,IL27,NUPR1,SGF29,SULT1A2,SULT1A1,EIF3C,EIF3CL,ATXN2L,TUFM,TUFM,MIR4721,SH2B1,ATP2A1,ATP2A1,ATP2A1-AS1,ATP2A1,RABEP2,RABEP2,CD19,NFATC2IP,NFATC2IP,MIR4517,SPNS1,LAT,RRN3P2 | 0.360445 | 340.578 | 464 | 281.607 |
| 2 | 2 | 2.2E+08 | 220585821 | STK36,TTLL4,CYP27A1,PRKAG3,WNT6,WNT10A,CDK5R2,FEV,CRYBA2,MIR375,LOC100129175,CFAP65,CFAP65,IHH,IHH,MIR3131,NHEJ1,SLC23A3,CNPPD1,RETREG2,ZFAND2B,ABCB6,ATG9A,ANKZF1,GLB1L,STK16,STK16,TUBA4A,TUBA4A,TUBA4A,TUBA4B,TUBA4B,DNAJB2,PTPRN,PTPRN,MIR153-1,RESP18,DNPEP,DES,SPEG,LOC100996693,GMPPA,ASIC4,CHPF,TMEM198,TMEM198,MIR3132,OBSL1,INHA,STK11IP,SLC4A3 | 0.360118 | 324.106 | 531 | 336.971 |

| | | | | | | | | |
|---|---|---|---|---|---|---|---|---|
| 2 | 4 | 467813 | 2209982 | ABCA11P,ZNF721,PIGG,PDE6B,ATP5ME,MYL5,MYL5,SLC49A3,SLC49A3,PCGF3,LOC100129917,CPLX1,GAK,TMEM175,DGKQ,SLC26A1,SLC26A1,IDUA,IDUA,FGFRL1,RNF212,SPON2,SPON2,LOC100130872,CTBP1-AS,CTBP1,CTBP1,CTBP1-AS2,MAEA,UVSSA,CRIPAK,NKX1-1,FAM53A,SLBP,TMEM129,TACC3,FGFR3,LETM1,NSD2,NSD2,SCARNA22,NELFA,NELFA,MIR943,C4orf48,NAT8L,POLN | 0.34971 | 343.724 | 446 | 273.733 |
| 2 | 7 | 65139852 | 74103469 | LOC441242,INTS4P2,LOC441242,CCT6P1,SNORA22,LOC441242,VKORC1L1,GUSB,ASL,CRCP,TPST1,LINC00174,KCTD7,RABGEF1,TMEM248,SBDS,TYW1,TYW1,MIR4650-1,MIR4650-2,PMS2P4,STAG3L4,AUTS2,GALNT17,GALNT17,MIR3914-1,MIR3914-2,CALN1,TYW1B,TYW1B,MIR4650-1,MIR4650-2,SBDSP1,POM121,POM121,NSUN5P2,TRIM74,LOC541473,LOC100101148,GTF2IP4,GTF2IP1,NCF1B,NSUN5,TRIM50,FKBP6,FZD9,BAZ1B,BCL7B,TBL2,MLXIPL,VPS37D,DNAJC30,BUD23,STX1A,STX1A,MIR4284,ABHD11,CLDN3,CLDN4,METTL27,TMEM270,ELN,LIMK1,EIF4H,EIF4H,MIR590,LAT2,RFC2,CLIP2,GTF2IRD1,GTF2I,LOC101926943 | 0.34678 | 308.12 | 653 | 365.44 |
| 2 | 19 | 36237221 | 36830604 | PSENEN,LIN37,HSPB6,PROSER3,ARHGAP33,ARHGAP33,LINC01529,PRODH2,NPHS1,KIRREL2,APLP1,NFKBID,HCST,TYROBP,LRFN3,SDHAF1,SYNE4,ALKBH6,LOC101927572,CLIP3,LOC101927572,THAP8,THAP8,WDR62,OVOL3,OVOL3,POLR2I,POLR2I,TBCB,CAPNS1,COX7A1,ZNF565,ZNF146 | 0.344331 | 327.122 | 264 | 166.957 |
| 2 | 2 | 95770270 | 96504630 | MRPS5,ZNF514,ZNF2,PROM2,KCNIP3,FAHD2A,TRIM43B,TRIM43 | 0.344058 | 297.786 | 109 | 59.9857 |
| 2 | X | 46917784 | 47696116 | JADE3,RGN,NDUFB11,RBM10,UBA1,UBA1,INE1,CDK16,USP11,ZNF157,ZNF157,SNORA11C,ZNF41,LINC01560,ARAF,SYN1,SYN1,TIMP1,SYN1,MIR4769,CFP,ELK1,UXT,UXT,UXT-AS1,UXT-AS1 | 0.34402 | 347.247 | 201 | 128.041 |
| 2 | 2 | 26513405 | 26586560 | ADGRF3,ADGRF3,SELENOI | 0.343529 | 296.97 | 26 | 15.5187 |

| | | | | | | | | |
|---|---|---|---|---|---|---|---|---|
| 2 | 1 | 1.51E+08 | 154574704 | CTSS,CTSK,ARNT,SETDB1,CERS2,ANXA9,MINDY1,PRUNE1,BNIPL,C1orf56,CDC42SE1,MLLT11,GABPB2,SEMA6C,TNFAIP8L2,TNFAIP8L2-SCNM1,TNFAIP8L2-SCNM1,LYSMD1,TNFAIP8L2-SCNM1,SCNM1,TNFAIP8L2-SCNM1,SCNM1,TMOD4,TMOD4,VPS72,PIP5K1A,PSMD4,LOC100507670,LOC100507670,ZNF687,ZNF687,PI4KB,RFX5,SELENBP1,PSMB4,POGZ,CGN,TUFT1,TUFT1,MIR554,SNX27,CELF3,RIIAD1,MRPL9,MRPL9,OAZ3,OAZ3,TDRKH,LINGO4,RORC,C2CD4D,C2CD4D,LOC100132111,THEM5,THEM4,S100A10,S100A11,TCHHL1,TCHH,RPTN,HRNR,FLG,FLG,FLG-AS1,FLG-AS1,FLG2,CRNN,LCE5A,CRCT1,LCE3E,LCE3D,LCE3A,LCE2D,LCE2C,LCE2B,LCE2A,LCE4A,C1orf68,KPRP,LCE1F,LCE1E,LCE1C,LCE1B,LCE1A,LCE6A,SMCP,IVL,SPRR4,SPRR1A,SPRR3,SPRR1B,SPRR2C,SPRR2G,LELP1,PRR9,LOR,PGLYRP3,PGLYRP4,S100A9,S100A12,S100A8,S100A8,S100A7A,S100A7L2,S100A7,S100A6,S100A5,S100A4,LOC101928034,S100A3,S100A2,S100A16,S100A14,S100A13,S100A13,S100A1,CHTOP,SNAPIN,ILF2,NPR1,INTS3,SLC27A3,GATAD2B,DENND4B,CRTC2,SLC39A1,SLC39A1,MIR6737,CREB3L4,JTB,RAB13,RPS27,NUP210L,NUP210L,MIR5698,TPM3,MIR190B,C1orf189,C1orf43,C1orf43,UBAP2L,UBAP2L,HAX1,AQP10,ATP8B2,LOC101928101,IL6R,IL6R,SHE,TDRD10,UBE2Q1,UBE2Q1,UBE2Q1-AS1,CHRNB2,ADAR | 0.337804 | 356.497 | 1197 | 716.054 |

| | | | | | | | | |
|---|---|---|---|---|---|---|---|---|
| 2 | 19 | 44989246 | 50491732 | ZNF180,CEACAM20,IGSF23,PVR,PVR,MIR4531,CEACAM19,CEACAM16,BCL3,BCL3,MIR8085,CBLC,BCAM,NECTIN2,TOMM40,APOE,APOC1,APOC1P1,APOC4,APOC4-APOC2,APOC4-APOC2,APOC2,CLPTM1,RELB,CLASRP,ZNF296,GEMIN7,GEMIN7-AS1,GEMIN7,PPP1R37,NKPD1,TRAPPC6A,BLOC1S3,EXOC3L2,MARK4,CKM,KLC3,ERCC2,PPP1R13L,CD3EAP,CD3EAP,ERCC1,ERCC1,FOSB,RTN2,PPM1N,VASP,OPA3,GPR4,EML2,EML2,MIR330,EML2,EML2-AS1,GIPR,GIPR,MIR642A,MIR642B,SNRPD2,QPCTL,FBXO46,BHMG1,SIX5,SIX5,DM1-AS,DM1-AS,DMPK,DMPK,DMWD,RSPH6A,SYMPK,FOXA3,IRF2BP1,MYPOP,NANOS2,NOVA2,CCDC61,MIR769,PGLYRP1,IGFL4,IGFL3,IGFL2,IGFL1,HIF3A,PPP5C,CCDC8,PNMA8A,PPP5D1,PPP5D1,PNMA8B,CALM3,PTGIR,GNG8,DACT3,DACT3,DACT3-AS1,DACT3-AS1,PRKD2,PRKD2,PRKD2,MIR320E,STRN4,STRN4,FKRP,FKRP,SLC1A5,AP2S1,ARHGAP35,NPAS1,TMEM160,ZC3H4,SAE1,BBC3,BBC3,MIR3190,MIR3191,CCDC9,INAFM1,C5AR1,C5AR2,DHX34,MEIS3,SLC8A2,KPTN,NAPA-AS1,NAPA,NAPA,ZNF541,BICRA,EHD2,NOP53,NOP53,SNORD23,NOP53,NOP53-AS1,SELENOW,TPRX1,CRX,SULT2A1,BSPH1,ELSPBP1,CABP5,PLA2G4C,PLA2G4C,PLA2G4C-AS1,LIG1,ZSWIM9,CARD8,ZNF114,CCDC114,EMP3,TMEM143,SYNGR4,KDELR1,GRIN2D,GRWD1,KCNJ14,CYTH2,LMTK3,SULT2B1,FAM83E,FAM83E,SPACA4,RPL18,SPHK2,DBP,CA11,SEC1P,SEC1P,NTN5,SEC1P,FUT2,LOC105447645,MAMSTR,RASIP1,IZUMO1,FUT1,FGF21,BCAT2,HSD17B14,PLEKHA4,PPP1R15A,TULP2,NUCB1,NUCB1,NUCB1-AS1,DHDH,BAX,FTL,GYS1,RUVBL2,RUVBL2,MIR6798,R | 0.336243 | 336.138 | 2047 | 1260.66 |
| 2 | 1 | 47843057 | 48713255 | LINC01389,FOXE3,FOXD2,TRABD2B,SLC5A9 | 0.335768 | 291.934 | 41 | 23.9491 |
| 2 | 16 | 85144032 | 90294253 | FAM92B,MIR5093,GSE1,GINS2,C16orf74,C16orf74,MIR1910,EMC8,COX4I1,IRF8,LINC00917,FOXF1,MTHFSD,FLJ30679,FOXC2-AS1,FOXC2,FOXC2,FOXL1,C16orf95,FBXO31,MAP1LC3B,ZCCHC14,JPH3,KLHDC4,SLC7A5,CA5A,BANP,LOC400553,ZNF469,ZFPM1,ZFPM1,MIR5189,ZFPM1,LOC100128882,ZC3H18,IL17C,CYBA,MVD,SNAI3-AS1,SNAI3,RNF166,CTU2,PIEZO1,PIEZO1,MIR4722,PIEZO1,LOC100289580,PIEZO1,LOC339059,CDT1,APRT,GALNS,GALNS,TRAPPC2L,TRAPPC2L,PABPN1L,CBFA2T3,CBFA2T3,LOC101927793,CBFA2T3,LOC100129697,ACSF3,LINC02138,CDH15,SLC22A31,ZNF778,ANKRD11,ANKRD11,LOC100287036,SPG7,RPL13,RPL13,SNORD68,CPNE7,DPEP1,CHMP1A,CHMP1A,SPATA33,SPATA33,CDK10,SPATA2L,VPS9D1,VPS9D1,VPS9D1-AS1,VPS9D1,ZNF276,ZNF276,ZNF276,FANCA,FANCA,SPIRE2,TCF25,MC1R,TUBB3,DEF8,CENPBD1,AFG3L1P,DBNDD1,DBNDD1,GAS8,GAS8,GAS8,GAS8-AS1,GAS8,URAHP,PRDM7 | 0.334549 | 316.222 | 935 | 571.329 |

| | | | | | | | | |
|---|---|---|---|---|---|---|---|---|
| 2 | 10 | 71993005 | 73857207 | PPA1,NPFFR1,LRRC20,EIF4EBP2,NODAL,PALD1,PRF1,ADAMTS14,TBATA,SGPL1,PCBD1,UNC5B,SLC29A3,CDH23,CDH23,CDH23-AS1,CDH23,C10orf105,CDH23,VSIR,PSAP,CHST3,SPOCK2,ASCC1 | 0.330265 | 337.699 | 299 | 183.144 |
| 2 | 19 | 43857807 | 44339748 | CD177,TEX101,LYPD3,PHLDB3,ETHE1,ZNF575,XRCC1,PINLYP,IRGQ,ZNF576,ZNF428,ZNF428,SRRM5,CADM4,PLAUR,IRGC,SMG9,KCNN4,LYPD5,ZNF283 | 0.329681 | 329.117 | 164 | 101.624 |
| 2 | 1 | 2.01E+08 | 202300445 | CAMSAP2,GPR25,C1orf106,KIF21B,CACNA1S,TMEM9,IGFN1,PKP1,TNNT2,LAD1,TNNI1,PHLDA3,CSRP1,NAV1,NAV1,IPO9-AS1,NAV1,IPO9-AS1,MIR5191,NAV1,IPO9-AS1,MIR1231,IPO9-AS1,IPO9,IPO9,SHISA4,LMOD1,TIMM17A,RNPEP,RNPEP,ELF3-AS1,ELF3,GPR37L1,ARL8A,PTPN7,PTPRVP,LGR6 | 0.327066 | 310.719 | 423 | 258.021 |
| 2 | 6 | 13802483 | 17102567 | MCUR1,RNF182,CD83,JARID2,DTNBP1,MYLIP,MYLIP,MIR4639,GMPR,ATXN1,ATXN1,LOC101928433,STMND1 | 0.3268 | 286.123 | 127 | 74.3905 |
| 2 | 17 | 4269495 | 5043011 | UBE2G1,SPNS3,SPNS2,SPNS2,MYBBP1A,MYBBP1A,GGT6,SMTNL2,LINC01996,ALOX15,PELP1,PELP1,LOC101559451,ARRB2,MED11,CXCL16,ZMYND15,TM4SF5,VMO1,GLTPD2,PSMB6,PLD2,MINK1,CHRNE,CHRNE,C17orf107,GP1BA,SLC25A11,RNF167,PFN1,ENO3,SPAG7,CAMTA2,INCA1,KIF1C,KIF1C,LOC102724009,SLC52A1,ZFP3,ZNF232,USP6 | 0.324338 | 336.682 | 378 | 239.418 |
| 2 | 10 | 5855141 | 7796017 | GDI2,ANKRD16,FBXO18,IL15RA,IL2RA,RBM17,PFKFB3,PFKFB3,MIR3155A,MIR3155B,LOC399715,LOC399716,PRKCQ,SFMBT2,ITIH5,ITIH2,KIN | 0.311328 | 305.048 | 216 | 127.316 |
| 2 | 5 | 4087462 | 34869218 | ADAMTS16,ICE1,LINC02145,MED10,UBE2QL1,NSUN2,SRD5A1,PAPD7,MIR4278,LOC442132,ADCY2,C5orf49,FASTKD3,FASTKD3,MTRR,MTRR,MIR4458HG,MIR4458,SEMA5A,SEMA5A,MIR4636,SNHG18,SNORD123,TAS2R1,FAM173B,CCT5,CMBL,MARCH6,ROPN1L,LINC02213,ANKRD33B,DAP,CTNND2,DNAH5,TRIO,FAM105A,OTULIN,ANKH,ANKH,LOC100130744,ANKH,MIR4637,FBXL7,FBXL7,MIR887,MARCH11,ZNF622,RETREG1,RETREG1,LOC101929524,MYO10,BASP1,CDH18,GUSBP1,CDH12,PRDM9,C5orf17,CDH10,LINC02228,CDH9,CDH6,DROSHA,C5orf22,PDZD2,PDZD2,MIR4279,GOLPH3,MTMR12,ZFR,ZFR,MIR579,SUB1,NPR3,TARS,ADAMTS12,RXFP3,SLC45A2,AMACR,C1QTNF3-AMACR,C1QTNF3-AMACR,C1QTNF3,RAI14,TTC23L | 0.309976 | 303.478 | 1294 | 753.475 |
| 2 | 8 | 1.22E+08 | 124781097 | MTBP,SNTB1,HAS2,LINC01151,ZHX2,DERL1,TBC1D31,FAM83A,MIR4663,C8orf76,C8orf76,ZHX1-C8orf76,ZHX1-C8orf76,ZHX1,ATAD2,ATAD2,MIR548D1,WDYHV1,FBXO32,KLHL38,ANXA13,FAM91A1 | 0.308058 | 381.921 | 206 | 125.3 |

| | | | | | | | | |
|---|---|---|---|---|---|---|---|---|
| 2 | 19 | 38314683 | 40514526 | LOC644554,LOC100631378,WDR87,SIPA1L3,DPF1,PPP1R14A,SPINT2,YIF1B,C19orf33,YIF1B,KCNK6,CATSPERG,PSMD8,GGN,SPRED3,FAM98C,RASGRP4,RYR1,MAP4K1,MAP4K1,LOC105372397,EIF3K,ACTN4,CAPN12,LGALS7,LGALS7B,LGALS4,ECH1,HNRNPL,RINL,SIRT2,NFKBIB,CCER2,SARS2,SARS2,MRPS12,MRPS12,FBXO17,FBXO27,ACP7,PAK4,NCCRP1,SYCN,IFNL3,IFNL2,IFNL1,LRFN1,GMFG,SAMD4B,PAF1,MED29,ZFP36,MIR4530,PLEKHG2,RPS16,SUPT5H,TIMM50,DLL3,SELENOV,EID2B,EID2,LGALS13,LGALS16,LGALS17A,LGALS14,CLC,LEUTX,DYRK1B,FBL,FCGBP,PSMC4,ZNF546 | 0.307892 | 332.205 | 817 | 504.618 |
| 2 | 19 | 13317371 | 19779731 | CACNA1A,CCDC130,MRI1,C19orf53,ZSWIM4,LOC284454,MIR24-2,MIR27A,MIR23A,NANOS3,MIR181C,MIR181D,NANOS3,C19orf57,CC2D1A,PODNL1,PODNL1,DCAF15,DCAF15,RFX1,RLN3,IL27RA,PALM3,MISP3,MISP3,MIR1199,C19orf67,SAMD1,PRKACA,ASF1B,LOC100507373,ADGRL1,ADGRL1,ADGRE5,DDX39A,PKN1,PTGER1,GIPC1,DNAJB1,MIR639,TECR,TECR,NDUFB7,CLEC17A,ADGRE3,ZNF333,ADGRE2,OR7C1,OR7A5,OR7A10,OR7A17,OR7C2,SLC1A6,CCDC105,CASP14,OR1I1,SYDE1,ILVBL,NOTCH3,NOTCH3,MIR6795,EPHX3,BRD4,AKAP8,AKAP8L,WIZ,RASAL3,PGLYRP2,CYP4F22,CYP4F8,CYP4F3,CYP4F12,OR10H2,OR10H3,CYP4F24P,OR10H5,OR10H1,CYP4F2,CYP4F11,OR10H4,LINC00661,LINC00905,TPM4,RAB8A,HSH2D,CIB3,FAM32A,AP1M1,KLF2,EPS15L1,CALR3,C19orf44,C19orf44,CHERP,CHERP,SLC35E1,MED26,SMIM7,TMEM38A,NWD1,SIN3B,F2RL3,CPAMD8,HAUS8,MYO9B,USE1,OCEL1,NR2F6,USHBP1,BABAM1,ANKLE1,ABHD8,MRPL34,DDA1,ANO8,GTPBP3,PLVAP,BST2,MVB12A,TMEM221,NXNL1,SLC27A1,SLC27A1,LOC100507551,LOC100507551,PGLS,PGLS,FAM129C,COLGALT1,UNC13A,MAP1S,FCHO1,B3GNT3,INSL3,JAK3,RPL18A,RPL18A,SNORA68,SLC5A5,CCDC124,KCNN1,ARRDC2,IL12RB1,IL12RB1,MAST3,MAST3,PIK3R2,IFI30,MPV17L2,RAB3A,PDE4C,IQCN,JUND,MIR3188,LSM4,PGPEP1,GDF15,GDF15,MIR3189,LRRC25,SSBP4,ISYNA1,ELL,FKBP8,KXD1,UBA52,REX1BD,CRLF1,TMEM59L,KLHL26,CRTC1,COMP,UPF1,CERS1,GDF1,COPE,DDX49,HOMER3,SUGP2,SUGP2,ARMC6,ARMC6,SLC25A42,TMEM161A,MEF2B,BORCS8-MEF2B,BORCS8-MEF2B,BORCS8,RFXANK,RFXANK,NR2C2AP,NR2C2AP,NCAN,HAPLN4,TM6SF2,SUGP1,MAU2,GATAD2A,MIR640,GATAD2A,TSSK6,NDUFA13,YJEFN3,CILP2,PBX4,LPAR2 | 0.306447 | 328.415 | 2153 | 1323.02 |
| 2 | 1 | 2.26E+08 | 227216933 | LIN9,PARP1,STUM,ITPKB,PSEN2,COQ8A,CDC42BPA | 0.303895 | 330.999 | 105 | 61.8717 |

| | | | | | | | | |
|---|---|---|---|---|---|---|---|---|
| 2 | 19 | 40595840 | 43087550 | MAP3K10,TTC9B,CNTD2,AKT2,AKT2,MIR641,C19orf47,C19orf47,PLD3,PLD3,PLD3,MIR6796,HIPK4,PRX,SERTAD1,SERTAD3,BLVRB,SPTBN4,SHKBP1,LTBP4,NUMBL,COQ8B,ITPKC,C19orf54,SNRPA,MIA,MIA-RAB4B,MIA-RAB4B,RAB4B,RAB4B-EGLN2,RAB4B-EGLN2,EGLN2,CYP2T1P,CYP2A6,CYP2A7,CYP2G1P,CYP2B7P,CYP2B6,CYP2A13,CYP2F1,CYP2S1,AXL,HNRNPUL1,CCDC97,TGFB1,B9D2,TMEM91,EXOSC5,BCKDHA,B3GNT8,DMAC2,ERICH4,PCAT19,CEACAM21,CEACAM4,CEACAM7,CEACAM5,CEACAM6,CEACAM3,LYPD4,DMRTC2,RPS19,RPS19,MIR6797,CD79A,ARHGEF1,LOC100505585,RABAC1,ATP1A3,GRIK5,ZNF574,POU2F2,LOC100505622,MIR4323,DEDD2,ZNF526,GSK3A,ERF,CIC,PAFAH1B3,PAFAH1B3,PRR19,PRR19,TMEM145,MEGF8,MEGF8,MIR8077,CNFN,LOC101930071,LIPE-AS1,LIPE,LIPE-AS1,LIPE,LIPE-AS1,CXCL17,LIPE-AS1,LIPE-AS1,CEACAM1,LIPE-AS1,CEACAM8 | 0.302914 | 336.599 | 877 | 534.093 |
| 2 | 11 | 6173245 | 6704601 | OR52B2,OR52W1,C11orf42,FAM160A2,CNGA4,CCKBR,CAVIN3,SMPD1,APBB1,HPX,TRIM3,ARFIP2,ARFIP2,TIMM10B,TIMM10B,DNHD1,RRP8,ILK,ILK,TAF10,TAF10,TPP1,DCHS1,MRPL17 | 0.302478 | 322.386 | 275 | 185.908 |
| 2 | 7 | 1.42E+08 | 143884193 | PRSS58,PRSS1,PRSS3P2,EPHB6,TRPV6,TRPV5,LLCFC1,KEL,OR9A2,OR6V1,OR6W1P,PIP,TAS2R39,TAS2R40,LOC105375545,LOC105375545,GSTK1,LOC105375545,TMEM139,TMEM139,CASP2,CLCN1,FAM131B,ZYX,ZYX,MIR6892,EPHA1,EPHA1,EPHA1-AS1,EPHA1-AS1,TAS2R60,EPHA1-AS1,TAS2R41,TCAF2,LOC154761,TCAF1,OR2F2,OR2F1,OR6B1,OR2A5,OR2A25,OR2A12,OR2A2,OR2A14,ARHGEF35 | 0.301639 | 359.658 | 418 | 242.294 |
| 2 | 9 | 34343932 | 35956694 | MYORG,C9orf24,FAM219A,DNAI1,ENHO,CNTFR,CNTFR,CNTFR-AS1,RPP25L,DCTN3,ARID3C,SIGMAR1,GALT,IL11RA,IL11RA,CCL27,CCL27,LOC730098,CCL19,CCL21,FAM205A,FAM205BP,PHF24,DNAJB5,C9orf131,VCP,FANCG,PIGO,STOML2,FAM214B,UNC13B,RUSC2,RUSC2,FAM166B,FAM166B,TESK1,TESK1,MIR4667,CD72,SIT1,CCDC107,CCDC107,ARHGEF39,ARHGEF39,CA9,TPM2,TLN1,TLN1,MIR6852,CREB3,MIR6853,CREB3,GBA2,RGP1,RGP1,MSMP,MSMP,NPR2,NPR2,SPAG8,SPAG8,HINT2,FAM221B,TMEM8B,OR13J1,HRCT1,SPAAR | 0.29055 | 312.259 | 559 | 360.809 |

| | | | | | | | | |
|---|---|---|---|---|---|---|---|---|
| 2 | 11 | 60511170 | 62782458 | MS4A18,MS4A15,MS4A10,CCDC86,PTGDR2,ZP1,PRPF19,TMEM109,TMEM132A,SLC15A3,CD6,CD5,VPS37C,PGA3,PGA4,PGA5,VWCE,DDB1,TKFC,TKFC,CYB561A3,CYB561A3,TMEM138,TMEM216,CPSF7,SDHAF2,PPP1R32,MIR4488,LRRC10B,SYT7,RPLP0P2,DAGLA,MYRF,MYRF,DKFZP434K028,TMEM258,TMEM258,MIR611,FEN1,FADS1,FADS1,MIR1908,FADS1,FADS2,FADS2,FADS3,FADS3,MIR6746,RAB3IL1,BEST1,FTH1,INCENP,SCGB1D1,SCGB2A1,SCGB1D2,SCGB2A2,SCGB1D4,ASRGL1,SCGB1A1,AHNAK,EEF1G,EEF1G,MIR3654,EEF1G,MIR6747,TUT1,MTA2,EML3,ROM1,B3GAT3,GANAB,INTS5,C11orf98,LBHD1,LBHD1,CSKMT,SNORA57,LBHD1,CSKMT,LBHD1,LBHD1,UQCC3,UQCC3,UBXN1,LRRN4CL,BSCL2,HNRNPUL2-BSCL2,BSCL2,HNRNPUL2-BSCL2,GNG3,HNRNPUL2-BSCL2,HNRNPUL2,TTC9C,ZBTB3,POLR2G,TAF6L,TAF6L,TMEM179B,TMEM179B,TMEM179B,MIR6748,TMEM223,NXF1,MIR6514,NXF1,STX5,LOC105369332,WDR74,WDR74,SNHG1,SNORD22,SNHG1,SNORD31,SNHG1,SNORD30,SNHG1,SNORD29,SNHG1,SNORD28,SNHG1,SNORD27,SNORD26,SNHG1,SNORD25,SLC3A2,CHRM1,SLC22A6,SLC22A8 | 0.288848 | 328.157 | 834 | 521.764 |
| 2 | 10 | 43694491 | 51877836 | RASGEF1A,FXYD4,HNRNPF,ZNF487,ZNF239,ZNF485,ZNF32-AS3,ZNF32,ZNF32-AS1,ZNF32-AS3,ZNF32,ZNF32-AS2,HNRNPA3P1,LINC00619,CXCL12,TMEM72-AS1,TMEM72,RASSF4,RASSF4,DEPP1,C10orf25,C10orf25,ZNF22,ZNF22,RSU1P2,ANKRD30BP3,MIR3156-1,OR13A1,ALOX5,ALOX5,LOC102724323,MARCH8,ZFAND4,WASHC2C,PTPN20,FAM35BP,LOC102724593,LOC102724593,SYT15,SYT15,GPRIN2,NPY4R,NPY4R2,ANXA8,FAM35DP,ANTXRL,ANXA8L1,ZNF488,RBP3,GDF2,GDF10,FRMPD2,MAPK8,ARHGAP22,WDFY4,WDFY4,LRRC18,MIR4294,VSTM4,FAM170B-AS1,FAM170B,C10orf128,C10orf71,DRGX,ERCC6,ERCC6,PGBD3,CHAT,SLC18A3,CHAT,C10orf53,OGDHL,PARG,PARG,PARGP1,TIMM23B,TIMM23B,MSMB,TIMM23B,NCOA4,TIMM23B,TIMM23,WASHC2A | 0.288633 | 335.449 | 720 | 410.315 |
| 2 | 15 | 79189824 | 80365024 | CTSH,RASGRF1,RASGRF1,LOC100129540,ANKRD34C-AS1,MIR184,ANKRD34C,TMED3,KIAA1024,MTHFS,ST20-MTHFS,ST20-MTHFS,ST20,ST20-MTHFS,ST20,ST20-AS1,ST20,ST20-AS1,BCL2A1 | 0.286606 | 320.838 | 103 | 62.8308 |
| 2 | 3 | 46667305 | 47098982 | ALS2CL,TMIE,PRSS50,PRSS46P,PRSS45,PRSS42,MYL3,PTH1R,CCDC12,NBEAL2,NRADDP,SETD2 | 0.282882 | 310.135 | 160 | 105.155 |
| 2 | 3 | 3221275 | 4942387 | CRBN,LRRN1,SETMAR,SUMF1,ITPR1,BHLHE40-AS1 | 0.281948 | 317.403 | 124 | 75.1333 |
| 2 | 16 | 10574694 | 11792127 | ATF7IP2,EMP2,TEKT5,NUBP1,NUBP1,TVP23A,TVP23A,CIITA,DEXI,CLEC16A,SOCS1,TNP2,PRM3,PRM2,PRM1,RMI2,LITAF,SNN,TXNDC11 | 0.277521 | 300.916 | 162 | 95.2519 |
| 2 | 10 | 1.12E+08 | 112635218 | SMC3,RBM20 | 0.277164 | 328.476 | 31 | 18.3818 |
| 2 | 2 | 2.19E+08 | 219529651 | CNOT9,PLCD4,ZNF142,BCS1L,RNF25 | 0.274126 | 295.15 | 57 | 38.2888 |

| | | | | | | | | |
|---|---|---|---|---|---|---|---|---|
| 2 | 11 | 70229740 | 72694284 | CTTN,SHANK2,SHANK2,SHANK2-AS3,SHANK2,MIR3664,DHCR7,NADSYN1,KRTAP5-7,KRTAP5-8,KRTAP5-9,KRTAP5-10,KRTAP5-11,FAM86C1,ALG1L9P,LOC100133315,RNF121,IL18BP,NUMA1,NUMA1,LOC100128494,NUMA1,MIR3165,LRTOMT,LRTOMT,LAMTOR1,LRTOMT,ANAPC15,ANAPC15,FOLR3,FOLR1,FOLR2,INPPL1,PHOX2A,CLPB,LINC01537,PDE2A,PDE2A,MIR139,ARAP1,ARAP1,ARAP1-AS2,STARD10,STARD10,MIR4692,ATG16L2,FCHSD2,FCHSD2,MIR4459 | 0.2733 | 299.958 | 459 | 276.247 |
| 2 | 9 | 1.29E+08 | 135147188 | MVB12B,LMX1B,ZBTB43,ZBTB34,RALGPS1,RALGPS1,ANGPTL2,GARNL3,SLC2A8,ZNF79,RPL12,RPL12,SNORA65,LRSAM1,FAM129B,STXBP1,STXBP1,MIR3911,CFAP157,CFAP157,PTRH1,PTRH1,TTC16,TOR2A,SH2D3C,MIR3960,MIR2861,CDK9,CDK9,FPGS,ENG,ENG,LOC102723566,AK1,AK1,MIR4672,ST6GALNAC6,ST6GALNAC4,PIP5KL1,DPM2,FAM102A,NAIF1,SLC25A25,PTGES2,PTGES2-AS1,LCN2,C9orf16,CIZ1,CIZ1,DNM1,DNM1,DNM1,MIR199B,MIR3154,GOLGA2,GOLGA2,SWI5,SWI5,TRUB2,COQ4,SLC27A4,SLC27A4,MIR1268A,MIR1268A,URM1,MIR1268A,MIR219A2,MIR219B,MIR1268A,CERCAM,MIR1268A,ODF2,MIR1268A,GLE1,MIR1268A,SPTAN1,MIR1268A,WDR34,SET,PKN3,ZDHHC12,LOC100506100,ZER1,ZER1,TBC1D13,ENDOG,ENDOG,SPOUT1,SPOUT1,KYAT1,LRRC8A,PHYHD1,DOLK,DOLK,NUP188,NUP188,SH3GLB2,MIGA2,DOLPP1,CRAT,PTPA,IER5L,C9orf106,NTMT1,C9orf50,NTMT1,NTMT1,ASB6,ASB6,PRRX2,PTGES,TOR1B,TOR1A,C9orf78,USP20,USP20,MIR6855,FNBP1,GPR107,LOC401554,NCS1,HMCN2,ASS1,FUBP3,PRDM12,EXOSC2,ABL1,QRFP,FIBCD1,LAMC3,AIF1L,NUP214,FAM78A,PLPP7,PRRC2B,PRRC2B,SNORD62A,SNORD62B,POMT1,UCK1,RAPGEF1,MED27,NTNG2,SETX | 0.272736 | 320.989 | 1477 | 905.07 |
| 2 | 1 | 1.76E+08 | 178646342 | RFWD2,PAPPA2,ASTN1,ASTN1,MIR488,BRINP2,SEC16B,CRYZL2P-SEC16B,RASAL2-AS1,RASAL2,TEX35,C1orf220 | 0.271883 | 306.996 | 180 | 109.034 |
| 2 | 1 | 2.08E+08 | 210001573 | CD46,MIR29B2CHG,MIR29C,MIR29B2CHG,MIR29B2,MIR29B2CHG,MIR29B2CHG,LOC148696,CD34,PLXNA2,MIR205HG,MIR205HG,MIR205,CAMK1G,LAMB3,LAMB3,MIR4260,LOC101930114,G0S2,LOC101930114,HSD11B1,TRAF3IP3,TRAF3IP3,C1orf74,C1orf74,IRF6,DIEXF | 0.27044 | 279.693 | 156 | 94.2888 |
| 2 | 14 | 23979187 | 24912092 | THTPA,ZFHX2,THTPA,AP1G2,AP1G2,LOC102724814,JPH4,DHRS2,DHRS4-AS1,DHRS4-AS1,DHRS4,DHRS4,DHRS4L2,DHRS4L1,CARMIL3,CPNE6,NRL,NRL,PCK2,DCAF11,FITM1,PSME1,EMC9,PSME2,MIR7703,PSME2,RNF31,IRF9,REC8,REC8,IPO4,IPO4,TM9SF1,TSSK4,CHMP4A,MDP1,NEDD8-MDP1,NEDD8-MDP1,NEDD8,GMPR2,TINF2,TGM1,RABGGTA,DHRS1,NOP9,NOP9,CIDEB,CIDEB,LTB4R2,CIDEB,LTB4R2,LTB4R,LTB4R2,LTB4R,LTB4R,ADCY4,RIPK3,NFATC4,NYNRIN,CBLN3,CBLN3,KHNYN,KHNYN,KHNYN,SDR39U1,SDR39U1 | 0.269046 | 311.261 | 514 | 333.279 |

| | | | | | | | | |
|---|---|---|---|---|---|---|---|---|
| 2 | 11 | 60500 | 2966214 | SCGB1C2,SCGB1C1,ODF3,BET1L,RIC8A,RIC8A,MIR6743,SIRT3,PSMD13,NLRP6,PGGHG,IFITM5,IFITM2,IFITM1,IFITM3,B4GALNT4,PKP3,SIGIRR,ANO9,PTDSS2,RNH1,HRAS,LRRC56,LMNTD2,LMNTD2,LOC692247,RASSF7,MIR210HG,MIR210,PHRF1,IRF7,CDHR5,SCT,DRD4,DEAF1,DEAF1,TMEM80,TMEM80,EPS8L2,TALDO1,GATD1,LOC171391,CEND1,SLC25A22,PIDD1,RPLP2,RPLP2,SNORA52,PNPLA2,CRACR2B,CD151,POLR2L,TSPAN4,CHID1,AP2A2,MUC6,MUC2,MUC5AC,MUC5B,MUC5B,MIR6744,TOLLIP,BRSK2,MOB2,DUSP8,KRTAP5-AS1,KRTAP5-1,KRTAP5-AS1,KRTAP5-2,KRTAP5-AS1,KRTAP5-3,KRTAP5-5,KRTAP5-6,IFITM10,CTSD,SYT8,TNNI2,LSP1,LSP1,MIR4298,LSP1,MIR7847,TNNT3,MRPL23,MRPL23-AS1,HOTS,H19,H19,H19,MIR675,IGF2,INS-IGF2,IGF2,INS-IGF2,MIR483,IGF2,INS-IGF2,IGF2-AS,INS-IGF2,INS-IGF2,INS,TH,MIR4686,ASCL2,C11orf21,C11orf21,TSPAN32,TSPAN32,CD81-AS1,CD81-AS1,CD81,CD81,TSSC4,TRPM5,KCNQ1,KCNQ1,KCNQ1OT1,KCNQ1,KCNQ1-AS1,CDKN1C,SLC22A18AS,SLC22A18AS,SLC22A18,SLC22A18,PHLDA2 | 0.262875 | 337.207 | 951 | 589.177 |
| 2 | 9 | 33290480 | 33528853 | NFX1,AQP7,AQP3,NOL6,ANKRD18B | 0.262326 | 299.554 | 74 | 45.7886 |
| 2 | X | 1.49E+08 | 149760468 | IDS,LINC00893,CXorf40A,CXorf40A,HSFX3,HSFX4,MAGEA9,MAGEA9B,HSFX2,HSFX1,TMEM185A,MAGEA11,HSFX1,HSFX2,MAGEA9B,MAGEA9,MAGEA8-AS1,MAGEA8,CXorf40A,HSFX4,CXorf40A,CXorf40B,LINC00894,MIR2114,MAMLD1 | 0.262287 | 322.409 | 105 | 59.8395 |
| 2 | 9 | 1.01E+08 | 102591237 | NANS,TRIM14,CORO2A,TBC1D2,TBC1D2,MIR6854,GABBR2,ANKS6,GALNT12,COL15A1,TGFBR1,ALG2,SEC61B,NR4A3 | 0.260057 | 292.65 | 197 | 115.931 |
| 2 | 20 | 21335327 | 26256532 | XRN2,NKX2-4,NKX2-2,PAX1,FOXA2,SSTR4,THBD,CD93,NXT1,GZF1,NAPB,CSTL1,CST11,CST8,CST9L,CST9,CST3,CST4,CST1,CST2,CST5,GGTLC1,SYNDIG1,CST7,APMAP,ACSS1,VSX1,LOC101926889,ENTPD6,ENTPD6,PYGB,PYGB,ABHD12,ABHD12,GINS1,NINL,NANP,ZNF337-AS1,ZNF337,ZNF337,FAM182B,LOC101926935,FAM182A,NCOR1P1,MIR663AHG,MIR663A | 0.25452 | 330.798 | 339 | 197.05 |
| 2 | 15 | 40512766 | 40894484 | BUB1B,BUB1B-PAK6,BUB1B-PAK6,PAK6,BUB1B-PAK6,PAK6,C15orf56,ANKRD63,PLCB2,CCDC9B,PHGR1,DISP2,KNSTRN,IVD,BAHD1,CHST14,CCDC32,MRPL42P5,CCDC32,RPUSD2 | 0.253649 | 318.577 | 165 | 106.188 |
| 2 | 17 | 61497304 | 61628178 | TANC2,CYB561,ACE,KCNH6,DCAF7 | 0.25237 | 353.521 | 67 | 42.2179 |
| 2 | 20 | 240537 | 468297 | C20orf96,ZCCHC3,SOX12,NRSN2,TRIB3,RBCK1,TBC1D20,CSNK2A1 | 0.250445 | 292.178 | 64 | 38.6179 |
| 2 | 2 | 26624832 | 27604623 | DRC1,OTOF,C2orf70,CIB4,KCNK3,SLC35F6,CENPA,DPYSL5,MAPRE3,TMEM214,AGBL5,OST4,EMILIN1,KHK,KHK,CGREF1,CGREF1,ABHD1,PREB,PRR30,TCF23,SLC5A6,SLC5A6,ATRAID,ATRAID,CAD,SLC30A3,DNAJC5G,TRIM54,UCN,MPV17,GTF3C2,GTF3C2,GTF3C2-AS1,EIF2B4,SNX17,SNX17,ZNF513,ZNF513,PPM1G | 0.249884 | 309.024 | 398 | 254.43 |

| | | | | | | | | |
|---|---|---|---|---|---|---|---|---|
| 2 | 22 | 41252371 | 45751029 | ST13,XPNPEP3,XPNPEP3,DNAJB7,RBX1,MIR1281,EP300,EP300,L3MBTL2,L3MBTL2,LOC100506544,L3MBTL2,CHADL,CHADL,RANGAP1,ZC3H7B,TEF,TOB2,PHF5A,ACO2,ACO2,POLR3H,POLR3H,CSDC2,PMM1,DESI1,XRCC6,SNU13,C22orf46,MEI1,CCDC134,SREBF2,SREBF2,MIR33A,SHISA8,MIR378I,TNFRSF13C,CENPM,LINC00634,SEPT3,WBP2NL,NAGA,FAM109B,SMDT1,NDUFA6,LOC101929829,CYP2D6,CYP2D7,TCF20,NFAM1,SERHL,SERHL,RRP7A,RRP7A,SERHL2,SERHL2,RRP7BP,RRP7BP,POLDIP3,CYB5R3,CYB5R3,ATP5MGL,A4GALT,ARFGAP3,PACSIN2,LOC100506679,TTLL1,TTLL1,BIK,MCAT,TSPO,TTLL12,FLJ30901,SCUBE1,MPPED1,EFCAB6-AS1,EFCAB6,EFCAB6,SULT4A1,PNPLA5,PNPLA3,SAMM50,PARVB,PARVG,SHISAL1,RTL6,LINC00207,PRR5,PRR5,PRR5-ARHGAP8,PRR5-ARHGAP8,ARHGAP8,PHF21B,NUP50,NUP50,LOC105373064,KIAA0930,KIAA0930,MIR1249,UPK3A,FAM118A,SMC1B | 0.247156 | 295.937 | 889 | 524.035 |
| 2 | 3 | 51696556 | 52570962 | RAD54L2,TEX264,GRM2,IQCF6,IQCF3,IQCF2,IQCF5-AS1,IQCF5,IQCF1,RRP9,PARP3,GPR62,PCBP4,ABHD14B,ABHD14A,ABHD14A-ACY1,ABHD14A-ACY1,ACY1,RPL29,DUSP7,LINC00696,POC1A,ALAS1,TLR9,TWF2,PPM1M,WDR82,WDR82,MIRLET7G,GLYCTK,GLYCTK,MIR135A1,DNAH1,BAP1,PHF7,SEMA3G,TNNC1,NISCH,STAB1,STAB1,NT5DC2,NT5DC2,SMIM4 | 0.246148 | 316.851 | 436 | 284.452 |
| 2 | 1 | 2.04E+08 | 207262906 | LINC00303,SOX13,ETNK2,ETNK2,ERLNC1,REN,KISS1,GOLT1A,PLEKHA6,PPP1R15B,PIK3C2B,MDM4,LRRN2,NFASC,CNTN2,TMEM81,RBBP5,DSTYK,TMCC2,NUAK2,KLHDC8A,LEMD1-AS1,LEMD1,LEMD1,BLACAT1,MIR135B,CDK18,MFSD4A,ELK4,SLC45A3,NUCKS1,RAB29,SLC41A1,PM20D1,LOC284581,SLC26A9,AVPR1B,RHEX,CTSE,SRGAP2,SRGAP2,SRGAP2D,IKBKE,IKBKE,MIR6769B,RASSF5,EIF2D,DYRK3,MAPKAPK2,IL10,IL19,IL20,IL24,FCMR,PIGR,FCAMR,C1orf116,YOD1,PFKFB2,C4BPB | 0.245301 | 301.234 | 649 | 398.898 |
| 2 | X | 1.52E+08 | 153906576 | MAGEA2,MAGEA2B,CSAG3,MAGEA3,CETN2,NSDHL,ZNF185,PNMA5,PNMA3,MAGEA1,ZNF275,ZFP92,TREX2,HAUS7,BGN,ATP2B3,CCNQ,DUSP9,PNCK,SLC6A8,BCAP31,ABCD1,PLXNB3,SRPK3,IDH3G,IDH3G,SSR4,SSR4,PDZD4,L1CAM,L1CAM,LCA10,LCA10,AVPR2,ARHGAP4,NAA10,RENBP,HCFC1,TMEM187,MIR3202-1,MIR3202-2,TMEM187,IRAK1,MECP2,OPN1LW,OPN1MW2,OPN1MW,OPN1MW3,TKTL1,FLNA,EMD,RPL10,RPL10,SNORA70,DNASE1L1,DNASE1L1,TAZ,TAZ,ATP6AP1,GDI1,FAM50A,FAM50A,MIR6858,PLXNA3,LAGE3,UBL4A,SLC10A3,FAM3A,G6PD,G6PD,IKBKG,IKBKG,CTAG1B,CTAG1A,CTAG2,GAB3 | 0.243079 | 317.438 | 692 | 432.852 |

| | | | | | | | | |
|---|---|---|---|---|---|---|---|---|
| 2 | 22 | 20609931 | 38945392 | ZNF74,SCARF2,KLHL22,MED15,POM121L4P,TMEM191A,PI4KA,PI4KA,SERPIND1,SNAP29,CRKL,LINC01637,AIFM3,LZTR1,THAP7,THAP7,THAP7-AS1,THAP7-AS1,TUBA3FP,P2RX6,SLC7A4,MIR649,LRRC74B,GGT2,HIC2,TMEM191C,PI4KAP2,UBE2L3,YDJC,CCDC116,SDF2L1,MIR301B,MIR130B,PPIL2,YPEL1,MAPK1,PPM1F,PPM1F,LOC100286925,TOP3B,PRAMENP,VPREB1,BMS1P20,ZNF280B,ZNF280A,PRAME,PRAME,LL22NC03-63E9.3,LL22NC03-63E9.3,GGTLC2,MIR5571,IGLL5,RSPH14,RSPH14,GNAZ,RAB36,BCR,IGLL1,DRICH1,GUSBP11,GUSBP11,RGL4,ZNF70,VPREB3,C22orf15,C22orf15,CHCHD10,CHCHD10,MMP11,SMARCB1,SMARCB1,DERL3,DERL3,SLC2A11,MIF-AS1,MIF,MIF-AS1,GSTT2B,GSTT2,DDTL,DDTL,DDT,DDT,GSTT2,GSTT2B,GSTTP1,LOC391322,GSTT1,GSTTP2,CABIN1,SUSD2,GGT5,SPECC1L,SPECC1L-ADORA2A,SPECC1L-ADORA2A,ADORA2A,ADORA2A-AS1,UPB1,GUCD1,GUCD1,SNRPD3,SNRPD3,GGT1,GGT1,LRRC75B,BCRP3,PIWIL3,SGSM1,TMEM211,KIAA1671,CRYBB3,CRYBB2,IGLL3P,LRP5L,CRYBB2P1,GRK3,MYO18B,SEZ6L,ASPHD2,ASPHD2,HPS4,HPS4,SRRD,SRRD,TFIP11,TFIP11,TPST2,TPST2,MIR548J,CRYBB1,CRYBA4,LOC284898,MN1,PITPNB,TTC28-AS1,MIR3199-1,MIR3199-2,TTC28-AS1,TTC28,TTC28,CHEK2,HSCB,CCDC117,XBP1,ZNRF3,C22orf31,KREMEN1,EMID1,RHBDD3,EWSR1,GAS2L1,RASL10A,AP1B1,AP1B1,MIR3653,SNORD125,RFPL1S,RFPL1S,RFPL1,NEFH,THOC5,NIPSNAP1,NF2,CABP7,CABP7,ZMAT5,ZMAT5,UQCR10,ASCC2,MTMR3,MTMR3,HORMAD2-AS1,HORMAD2,LIF,LIF,LOC91370,OSM,CASTOR1,TBC1D10A,SF3A1,SF3A1,CCDC157,CCDC157,CCDC157,KIAA | 0.241085 | 307.717 | 3185 | 1887.02 |
| 2 | 11 | 1.19E+08 | 120099868 | CXCR5,BCL9L,BCL9L,MIR4492,UPK2,FOXR1,CCDC84,RPS25,TRAPPC4,MIR3656,TRAPPC4,SLC37A4,HYOU1,VPS11,HMBS,H2AFX,DPAGT1,C2CD2L,HINFP,ABCG4,NLRX1,PDZD3,CCDC153,CBL,MCAM,MCAM,MIR6756,RNF26,C1QTNF5,MFRP,MFRP,C1QTNF5,USP2,USP2-AS1,USP2-AS1,THY1,NECTIN1,TRIM29,OAF | 0.239293 | 301.253 | 350 | 223.648 |
| 2 | 10 | 1.32E+08 | 135524247 | MIR378C,TCERG1L,LINC01164,PPP2R2D,BNIP3,JAKMIP3,DPYSL4,STK32C,LRRC27,PWWP2B,C10orf91,INPP5A,NKX6-2,CFAP46,ADGRA1,KNDC1,UTF1,VENTX,MIR202HG,MIR202,ADAM8,TUBGCP2,TUBGCP2,ZNF511,ZNF511,CALY,PRAP1,FUOM,ECHS1,ECHS1,MIR3944,PAOX,MTG1,SPRN,SCART1,CYP2E1,SYCE1,SYCE1,SPRNP1,FRG2B | 0.23834 | 280.456 | 481 | 284.406 |
| 2 | 10 | 75239772 | 75802327 | PPP3CB,PPP3CB-AS1,PPP3CB-AS1,USP54,USP54,MYOZ1,SYNPO2L,GLUD1P3,SEC24C,FUT11,CHCHD1,ZSWIM8,ZSWIM8,ZSWIM8-AS1,NDST2,CAMK2G,C10orf55,PLAU,VCL | 0.23315 | 299.106 | 188 | 118.53 |

| | | | | | | | | |
|---|---|---|---|---|---|---|---|---|
| 2 | 19 | 60500 | 9346560 | OR4F17,PLPP2,MIER2,THEG,C2CD4C,SHC2,ODF3L2,MADCAM1,TPGS1,CDC34,GZMM,BSG,HCN2,POLRMT,FGF22,RNF126,FSTL3,PRSS57,PALM,MISP,PTBP1,PTBP1,MIR4745,PLPPR3,PLPPR3,MIR3187,AZU1,PRTN3,ELANE,CFD,MED16,R3HDM4,KISS1R,ARID3A,WDR18,GRIN3B,GRIN3B,TMEM259,TMEM259,CNN2,ABCA7,ARHGAP45,POLR2E,GPX4,SBNO2,STK11,CBARP,ATP5F1D,MIDN,CIRBP-AS1,CIRBP-AS1,CIRBP,CIRBP,C19orf24,EFNA2,MUM1,NDUFS7,GAMT,DAZAP1,RPS15,APC2,C19orf25,PCSK4,REEP6,ADAMTSL5,PLK5,MEX3D,MBD3,UQCR11,TCF3,ONECUT3,ATP8B3,REXO1,REXO1,MIR1909,REXO1,LOC100288123,KLF16,ABHD17A,ADAT3,SCAMP4,SCAMP4,CSNK1G2,CSNK1G2-AS1,CSNK1G2,BTBD2,MKNK2,MOB3A,IZUMO4,AP3D1,DOT1L,PLEKHJ1,MIR1227,PLEKHJ1,MIR6789,PLEKHJ1,SF3A2,AMH,AMH,MIR4321,JSRP1,OAZ1,PEAK3,LINGO3,LSM7,SPPL2B,TMPRSS9,TMPRSS9,TIMM13,TIMM13,LMNB2,LMNB2,MIR7108,GADD45B,GNG7,DIRAS1,SLC39A3,SGTA,THOP1,ZNF554,ZNF555,ZNF556,ZNF57,ZNF77,TLE6,TLE2,AES,GNA11,GNA15,GNA15,LOC100996351,S1PR4,NCLN,CELF5,NFIC,SMIM24,DOHH,FZR1,C19orf71,MFSD12,HMG20B,GIPC3,TBXA2R,CACTIN-AS1,CACTIN,CACTIN,PIP5K1C,TJP3,TJP3,MIR1268A,MIR1268A,APBA3,MIR1268A,MRPL54,MIR1268A,RAX2,MIR1268A,MATK,MIR1268A,ZFR2,MIR1268A,ATCAY,MIR1268A,NMRK2,MIR1268A,DAPK3,MIR1268A,DAPK3,MIR637,MIR1268A,EEF2,MIR1268A,EEF2,SNORD37,PIAS4,ZBTB7A,MAP2K2,CREB3L3,SIRT6,ANKRD24,EBI3,CCDC94,SHD,TMIGD2,FSD1,STAP2,MPND,SH3GL1,CHAF1A,UBXN6,UBXN6,MIR4746,HDGFL2,HDGFL2,PLIN4,PLIN4,PLIN5,LRG1,SEMA6B,TNFAIP8L1,MYDGF,DPP9,DPP9,DPP9-AS1,MIR7-3HG,MIR7-3HG,MIR7- | 0.232108 | 306.279 | 3235 | 1987.33 |
| 2 | 7 | 1.27E+08 | 128922597 | ZNF800,GCC1,ARF5,FSCN3,PAX4,SND1,SND1,SND1-IT1,SND1,LRRC4,SND1,MIR593,MIR129-1,LEP,RBM28,PRRT4,IMPDH1,HILPDA,METTL2B,LINC01000,FAM71F2,FAM71F1,CALU,CALU,OPN1SW,OPN1SW,CCDC136,FLNC,FLNC,FLNC-AS1,ATP6V1F,LOC100130705,KCP,IRF5,TNPO3,LOC407835,TSPAN33,SMO,AHCYL2 | 0.23086 | 301.281 | 398 | 242.295 |
| 2 | 4 | 2242419 | 4422709 | POLN,HAUS3,MXD4,MIR4800,MXD4,ZFYVE28,CFAP99,RNF4,FAM193A,TNIP2,SH3BP2,ADD1,MFSD10,NOP14-AS1,NOP14,NOP14,GRK4,HTT,MSANTD1,RGS12,HGFAC,DOK7,LRPAP1,LINC00955,ADRA2C,OTOP1,TMEM128,LYAR,ZBTB49,NSG1,STX18 | 0.228897 | 326.772 | 410 | 246.749 |

| | | | | | | | | |
|---|---|---|---|---|---|---|---|---|
| 2 | 3 | 9004922 | 15252429 | RAD18,SRGAP3,THUMPD3,SETD5,LHFPL4,MTMR14,CPNE9,BRPF1,OGG1,OGG1,CAMK1,CAMK1,TADA3,TADA3,ARPC4,ARPC4-TTLL3,ARPC4,ARPC4-TTLL3,ARPC4-TTLL3,TTLL3,RPUSD3,CIDEC,JAGN1,IL17RE,IL17RC,CRELD1,PRRT3,PRRT3,PRRT3-AS1,EMC3,EMC3,EMC3-AS1,FANCD2,FANCD2,FANCD2OS,FANCD2OS,BRK1,VHL,IRAK2,TATDN2,GHRLOS,LINC00852,GHRL,GHRLOS,GHRL,SEC13,ATP2B2,ATP2B2,MIR378B,ATP2B2,MIR885,SLC6A11,SLC6A1,SLC6A1-AS1,SLC6A1,HRH1,ATG7,ATG7,VGLL4,VGLL4,TAMM41,SYN2,SYN2,TIMP4,PPARG,TSEN2,MKRN2OS,MKRN2,RAF1,TMEM40,CAND2,RPL32,RPL32,SNORA7A,IQSEC1,NUP210,HDAC11,FBLN2,WNT7A,TPRXL,CHCHD4,TMEM43,XPC,LSM3,SLC6A6,GRIP2,CCDC174,C3orf20,FGD5,NR2C2,NR2C2,MRPS25,MRPS25,RBSN,COL6A4P1,CAPN7 | 0.223472 | 303.699 | 1045 | 633.571 |
| 2 | 17 | 25268559 | 27794236 | MIR4522,WSB1,TBC1D3P5,KSR1,LGALS9,NOS2,LYRM9,NLK,PPY2P,KRT18P55,TMEM97,TMEM97,IFT20,IFT20,TNFAIP1,POLDIP2,TMEM199,TMEM199,MIR4723,SEBOX,VTN,SARM1,SARM1,SLC46A1,SLC46A1,SLC13A2,FOXN1,UNC119,PIGS,ALDOC,SPAG5,SPAG5-AS1,SGK494,SPAG5-AS1,KIAA0100,KIAA0100,SDF2,SUPT6H,PROCA1,RAB34,RPL23A,RPL23A,SNORD42B,RPL23A,SNORD4A,RPL23A,SNORD42A,RPL23A,SNORD4B,TLCD1,NEK8,TRAF4,FAM222B,ERAL1,MIR451A,MIR451B,MIR144,MIR4732,FLOT2,DHRS13,PHF12,PHF12,LOC101927018,SEZ6,PIPOX,MYO18A,TIAF1,MYO18A,CRYBA1,NUFIP2,MIR4523,TAOK1 | 0.2206 | 297.383 | 603 | 374.866 |
| 2 | 19 | 34718741 | 34919538 | KIAA0355,GPI,PDCD2L,UBA2 | 0.219684 | 329.675 | 52 | 33.2283 |
| 2 | 8 | 80523340 | 82195816 | STMN2,HEY1,MRPS28,TPD52,MIR5708,ZBTB10,ZNF704,PAG1,FABP5 | 0.216625 | 312.959 | 95 | 52.4627 |
| 2 | 16 | 9017048 | 10525187 | USP7,C16orf72,GRIN2A,ATF7IP2 | 0.21462 | 248.233 | 55 | 32.4746 |
| 2 | 10 | 60500 | 5803494 | TUBB8,ZMYND11,DIP2C,DIP2C,MIR5699,DIP2C,PRR26,LARP4B,GTPBP4,IDI2,IDI2,IDI2-AS1,IDI2-AS1,IDI2-AS1,IDI1,IDI1,WDR37,ADARB2,ADARB2,ADARB2-AS1,PFKP,PITRM1,PITRM1,PITRM1-AS1,KLF6,AKR1E2,AKR1C1,AKR1C2,AKR1C3,AKR1C8P,AKR1C4,UCN3,TUBAL3,NET1,CALML5,CALML3-AS1,CALML3-AS1,CALML3,ASB13,FAM208B | 0.213249 | 315.542 | 443 | 257.54 |

| | | | | | | | | |
|---|---|---|---|---|---|---|---|---|
| 2 | 21 | 44282339 | 47856044 | WDR4,NDUFV3,ERVH48-1,MIR5692B,PKNOX1,CBS,CBSL,CBSL,CBS,U2AF1,U2AF1L5,CRYAA,CRYAA2,SIK1B,SIK1,HSF2BP,HSF2BP,H2BFS,RRP1B,PDXK,CSTB,RRP1,AGPAT3,TRAPPC10,PWP2,C21orf33,ICOSLG,DNMT3L,DNMT3L,LOC105372833,AIRE,PFKL,C21orf2,TRPM2,TRPM2,TRPM2-AS,LRRC3,TSPEAR,TSPEAR,TSPEAR-AS1,TSPEAR,TSPEAR-AS2,TSPEAR,KRTAP10-1,TSPEAR,KRTAP10-2,TSPEAR,KRTAP10-3,TSPEAR,KRTAP10-4,TSPEAR,KRTAP10-5,TSPEAR,KRTAP10-6,TSPEAR,KRTAP10-7,TSPEAR,KRTAP10-8,TSPEAR,KRTAP10-9,TSPEAR,KRTAP10-10,TSPEAR,KRTAP10-11,TSPEAR,KRTAP12-4,TSPEAR,KRTAP12-3,TSPEAR,KRTAP12-2,TSPEAR,KRTAP12-1,TSPEAR,KRTAP10-12,UBE2G2,SUMO3,PTTG1IP,ITGB2,LINC01547,FAM207A,ADARB1,POFUT2,COL18A1,COL18A1,COL18A1-AS1,COL18A1,MIR6815,SLC19A1,PCBP3,COL6A1,COL6A2,FTCD,FTCD,FTCD-AS1,SPATC1L,LSS,MCM3AP-AS1,MCM3AP-AS1,MCM3AP,MCM3AP,YBEY,C21orf58,PCNT | 0.210988 | 308.81 | 813 | 484.363 |
| 2 | 7 | 10500 | 6821140 | FAM20C,PDGFA,PRKAR1B,PRKAR1B,LOC101926963,PRKAR1B,DNAAF5,DNAAF5,SUN1,GET4,ADAP1,COX19,CYP2W1,C7orf50,C7orf50,MIR339,C7orf50,GPR146,C7orf50,GPER1,ZFAND2A,UNCX,MICALL2,INTS1,MAFK,LOC100128653,MAFK,TMEM184A,PSMG3,PSMG3-AS1,ELFN1,MAD1L1,MAD1L1,MIR4655,MRM2,MRM2,NUDT1,NUDT1,SNX8,SNX8,MIR6836,EIF3B,CHST12,GRIFIN,LFNG,LFNG,MIR4648,BRAT1,IQCE,TTYH3,AMZ1,AMZ1,GNA12,GNA12,CARD11,LOC100129603,SDK1,FOXK1,AP5Z1,AP5Z1,MIR4656,RADIL,RADIL,PAPOLB,MMD2,RNF216P1,RBAK,RBAK-RBAKDN,RBAK-RBAKDN,RBAKDN,ZNF890P,WIPI2,SLC29A4,TNRC18,LOC100129484,FBXL18,FBXL18,MIR589,ACTB,FSCN1,RNF216,RNF216,MIR6874,ZNF815P,OCM,CCZ1,RSPH10B2,RSPH10B,PMS2,AIMP2,AIMP2,EIF2AK1,EIF2AK1,EIF2AK1,ANKRD61,USP42,CYTH3,FAM220A,RAC1,DAGLB,KDELR2,GRID2IP,ZDHHC4,C7orf26,ZNF853,ZNF12,PMS2CL,RSPH10B,RSPH10B2 | 0.210137 | 297.746 | 1150 | 670.269 |
| 2 | 11 | 73057864 | 73388409 | ARHGEF17,RELT,FAM168A,PLEKHB1 | 0.209349 | 278.557 | 55 | 33.4075 |
| 2 | 2 | 85046397 | 86080254 | DNAH6,TRABD2A,TMSB10,KCMF1,TCF7L1,TGOLN2,RETSAT,ELMOD3,CAPG,SH2D6,MAT2A,GGCX,VAMP8,VAMP5,RNF181,TMEM150A,USP39,C2orf68,USP39,SFTPB,GNLY,ATOH8,ST3GAL5 | 0.209183 | 282.266 | 195 | 117.917 |

| | | | | | | | | |
|---|---|---|---|---|---|---|---|---|
| 2 | 6 | 25819710 | 44311291 | SLC17A1,SLC17A3,SLC17A2,TRIM38,HIST1H1A,HIST1H3A,HIST1H4A,HIST1H4B,HIST1H3B,HIST1H2AB,HIST1H2BB,HIST1H3C,HIST1H1C,LOC108783645,HFE,HFE,HIST1H4C,HIST1H1T,HIST1H2BC,HIST1H2AC,HIST1H1E,HIST1H2BD,HIST1H2BE,HIST1H4D,HIST1H3D,HIST1H3D,HIST1H2AD,HIST1H2BF,HIST1H4E,HIST1H2BG,HIST1H2AE,HIST1H3E,HIST1H1D,HIST1H4F,HIST1H4G,HIST1H3F,HIST1H2BH,HIST1H3G,HIST1H2BI,HIST1H4H,BTN3A2,BTN2A2,BTN3A1,BTN2A3P,BTN3A3,BTN2A1,BTN2A1,LOC285819,BTN1A1,HMGN4,ABT1,ZNF322,HIST1H2BJ,HIST1H2AG,HIST1H2BK,HIST1H4I,HIST1H2BK,HIST1H2AH,HIST1H2AH,MIR3143,PRSS16,POM121L2,VN1R10P,ZNF204P,ZNF391,ZNF184,HIST1H2BL,HIST1H2AI,HIST1H3H,HIST1H2AJ,HIST1H2BM,HIST1H4J,HIST1H4K,HIST1H2AK,HIST1H2BN,HIST1H2AL,HIST1H1B,HIST1H3I,HIST1H4L,HIST1H3J,HIST1H2AM,HIST1H2BO,OR2B2,OR2B6,ZNF165,ZSCAN12P1,ZSCAN16-AS1,ZSCAN16-AS1,ZSCAN16,ZKSCAN8,ZNF192P1,ZSCAN9,ZKSCAN4,NKAPL,ZSCAN26,PGBD1,ZSCAN31,ZSCAN31,ZKSCAN3,ZKSCAN3,ZSCAN12,ZSCAN23,GPX6,GPX5,ZBED9,TRIM27,LINC01556,ZNF311,LOC100129636,OR2W1,LOC100129636,OR2B3,OR2J1,OR2J3,OR2J2,LOC101929006,OR14J1,OR5V1,OR12D3,OR12D2,OR12D1,OR11A1,OR10C1,OR2H1,MAS1L,UBD,SNORD32B,OR2H2,GABBR1,MOG,ZFP57,HLA-F,HLA-F,HLA-F-AS1,HLA-F-AS1,HCG4,HCG4,HLA-V,HLA-V,HLA-G,HLA-H,HLA-A,HCG9,ZNRD1ASP,HLA-J,ZNRD1ASP,ZNRD1,PPP1R11,RNF39,TRIM31,TRIM31,TRIM31-AS1,TRIM40,TRIM10,TRIM15,TRIM26,HCG17,HLA-L,HCG17,HLA-L,MIR6891,TRIM39,TRIM39-RPP21,TRIM39-RPP21,RPP21,HLA-E,GNL1,GNL1,PRR3,PRR3,ABCF1,ABCF1,MIR877,PPP1R | 0.2084 | 308.833 | 4580 | 2781.06 |
| 2 | 10 | 76748760 | 76971480 | KAT6B,DUPD1,DUSP13,SAMD8,VDAC2 | 0.207867 | 340.137 | 45 | 26.8674 |
| 2 | 2 | 74005381 | 74361995 | DUSP11,C2orf78,STAMBP,ACTG2,DGUOK,DGUOK,DGUOK-AS1,TET3 | 0.205529 | 313.504 | 83 | 51.3196 |
| 2 | 10 | 1.03E+08 | 104848914 | SEMA4G,SEMA4G,MIR608,SEMA4G,MRPL43,MRPL43,TWNK,LZTS2,LZTS2,PDZD7,PDZD7,SFXN3,KAZALD1,TLX1NB,TLX1NB,TLX1,LBX1,LBX1-AS1,BTRC,POLL,POLL,DPCD,DPCD,DPCD,MIR3158-1,MIR3158-2,FBXW4,FGF8,NPM3,MGEA5,KCNIP2-AS1,KCNIP2,KCNIP2,C10orf76,HPS6,LDB1,PPRC1,NOLC1,ELOVL3,PITX3,GBF1,NFKB2,PSD,FBXL15,CUEDC2,MIR146B,RPARP-AS1,C10orf95,RPARP-AS1,MFSD13A,ACTR1A,SUFU,TRIM8,ARL3,SFXN2,WBP1L,CYP17A1,BORCS7,BORCS7-ASMT,BORCS7-ASMT,AS3MT,CNNM2 | 0.201207 | 304.363 | 540 | 333.985 |
| 2 | 3 | 1.84E+08 | 184542551 | DVL3,AP2M1,ABCF3,VWA5B2,VWA5B2,MIR1224,ALG3,EEF1AKMT4,EEF1AKMT4-ECE2,EEF1AKMT4-ECE2,CAMK2N2,EEF1AKMT4-ECE2,ECE2,PSMD2,EIF4G1,EIF4G1,SNORD66,FAM131A,CLCN2,POLR2H,THPO,CHRD,LINC02054,EPHB3,MAGEF1,VPS8 | 0.201063 | 292.064 | 262 | 170.398 |
| 2 | 17 | 60685476 | 61020988 | TLK2,MRC2,MARCH10,MARCH10,LOC105371855 | 0.200774 | 306.441 | 56 | 34.8867 |

| | | | | | | | | |
|---|---|---|---|---|---|---|---|---|
| 2 | 1 | 1.6E+08 | 160969307 | ATP1A2,ATP1A4,CASQ1,LOC729867,PEA15,PEA15,DCAF8,PEX19,COPA,COPA,NCSTN,NCSTN,NHLH1,VANGL2,SLAMF6,CD84,SLAMF1,CD48,SLAMF7,LY9,CD244,ITLN1,LOC101928372,ITLN2,ITLN2,F11R | 0.200519 | 320.336 | 240 | 147.431 |
| 2 | 4 | 4474001 | 5977736 | STX18,MSX1,CYTL1,STK32B,LINC01587,EVC2,EVC,CRMP1,MIR378D1 | 0.194896 | 277.142 | 128 | 73.6073 |
| 2 | 12 | 1.24E+08 | 132599200 | DNAH10,CCDC92,ZNF664-RFLNA,ZNF664,ZNF664-RFLNA,RFLNA,NCOR2,NCOR2,MIR6880,SCARB1,UBC,MIR5188,DHX37,BRI3BP,AACS,TMEM132B,LOC283435,LINC02347,LINC02347,LINC00944,LINC00943,TMEM132C,TMEM132C,MIR3612,SLC15A4,GLT1D1,TMEM132D,TMEM132D,TMEM132D-AS2,LINC02418,FZD10,PIWIL1,RIMBP2,STX2,RAN,ADGRD1,ADGRD1,ADGRD1-AS1,LINC01257,SFSWAP,MMP17,ULK1,PUS1,EP400,EP400,SNORA49,EP400P1 | 0.194672 | 272.1 | 591 | 348.574 |
| 2 | 1 | 1.63E+08 | 167886939 | NUF2,PBX1,LMX1A,RXRG,LOC400794,LRRC52,MGST3,ALDH9A1,TMCO1,UCK2,UCK2,MIR3658,FAM78B,FAM78B,MIR921,FMO9P,POGK,TADA1,ILDR2,MAEL,GPA33,DUSP27,POU2F1,CD247,CREG1,RCSD1,MPZL1,ADCY10 | 0.184349 | 287.368 | 298 | 174.939 |
| 2 | 20 | 59431439 | 62959313 | CDH4,CDH4,LOC100128310,MIR1257,TAF4,LSM14B,PSMA7,SS18L1,MTG2,HRH3,OSBPL2,ADRM1,LAMA5,LAMA5,MIR4758,LAMA5,LAMA5-AS1,RPS21,CABLES2,RBBP8NL,GATA5,MIR1-1HG-AS1,MIR1-1HG,MIR1-1HG,MIR1-1,MIR1-1HG,MIR133A2,SLCO4A1,SLCO4A1,SLCO4A1-AS1,NTSR1,MRGBP,OGFR-AS1,OGFR,OGFR,COL9A3,TCFL5,TCFL5,DPH3P1,DIDO1,GID8,SLC17A9,BHLHE23,LINC01749,MIR124-3,YTHDF1,BIRC7,BIRC7,MIR3196,NKAIN4,NKAIN4,FLJ16779,ARFGAP1,ARFGAP1,MIR4326,COL20A1,CHRNA4,CHRNA4,LOC100130587,KCNQ2,EEF1A2,PPDPF,PTK6,SRMS,FNDC11,HELZ2,GMEB2,STMN3,RTEL1,RTEL1-TNFRSF6B,RTEL1-TNFRSF6B,TNFRSF6B,ARFRP1,ZGPAT,ZGPAT,LIME1,LIME1,SLC2A4RG,ZBTB46,ABHD16B,TPD52L2,DNAJC5,UCKL1,UCKL1,MIR1914,UCKL1,MIR647,UCKL1,UCKL1-AS1,ZNF512B,SAMD10,PRPF6,C20orf204,SOX18,TCEA2,RGS19,RGS19,MIR6813,OPRL1,LKAAEAR1,OPRL1,NPBWR2,MYT1,PCMTD2 | 0.182136 | 298.427 | 861 | 526.588 |
| 2 | 11 | 56258039 | 58275590 | OR5M8,OR5M11,OR5M10,OR5M1,OR5AP2,OR5AR1,OR9G9,OR9G1,OR9G4,OR5AK2,OR5AK4P,LRRC55,APLNR,TNKS1BP1,SSRP1,P2RX3,PRG3,PRG2,SLC43A3,RTN4RL2,SLC43A1,TIMM10,SMTNL1,UBE2L6,SERPING1,MIR130A,YPEL4,CLP1,ZDHHC5,MED19,TMX2,TMX2-CTNND1,TMX2-CTNND1,SELENOH,TMX2-CTNND1,BTBD18,TMX2-CTNND1,CTNND1,OR9Q1,OR6Q1,OR9Q1,OR9Q1,OR9I1,OR9Q2,OR1S2,OR1S1,OR10Q1,OR10W1,OR5B17,OR5B3,OR5B2,OR5B12,OR5B21 | 0.181663 | 291.858 | 349 | 215.475 |

| | | | | | | | | |
|---|---|---|---|---|---|---|---|---|
| 2 | 2 | 2.42E+08 | 243188873 | FARP2,FARP2,MIR3133,STK25,BOK,THAP4,ATG4B,DTYMK,ING5,D2HGDH,GAL3ST2,NEU4,PDCD1,RTP5,LINC01237,LOC285095,LINC01237,LOC285097,LINC01881 | 0.180448 | 297.841 | 156 | 94.0406 |
| 2 | 7 | 26903888 | 27805667 | SKAP2,HOXA1,HOXA2,HOXA3,HOXA4,HOXA-AS3,HOXA5,HOXA-AS3,HOXA6,HOXA-AS3,HOXA-AS3,HOXA7,HOXA7,HOXA9,HOXA10-HOXA9,HOXA10-HOXA9,HOXA10-AS,HOXA10-HOXA9,HOXA10-AS,MIR196B,HOXA10-HOXA9,HOXA10-AS,HOXA10,HOXA10-HOXA9,HOXA10,HOXA11,HOXA13,EVX1-AS,EVX1,HIBADH,HIBADH,TSL,TAX1BP1 | 0.18038 | 296.645 | 94 | 56.6589 |
| 2 | 10 | 28909162 | 32096041 | BAMBI,C10orf126,LYZL1,SVIL-AS1,SVIL,SVIL,SVIL,MIR604,SVIL,MIR938,JCAD,MTPAP,GOLGA2P6,MAP3K8,LYZL2,SVILP1,ZNF438,ZEB1-AS1,ZEB1,ZEB1 | 0.179987 | 314.172 | 181 | 108.585 |
| 2 | 22 | 40038803 | 41215348 | CACNA1I,ENTHD1,GRAP2,FAM83F,TNRC6B,ADSL,SGSM3,MKL1,MCHR1,SLC25A17,SLC25A17,MIR4766 | 0.176805 | 293.237 | 201 | 120.584 |
| 2 | 16 | 53859813 | 58753095 | FTO,LINC02169,IRX3,IRX5,IRX6,MMP2,LPCAT2,LPCAT2,CAPNS2,SLC6A2,CES1P1,CES1,CES5A,GNAO1,GNAO1,DKFZP434H168,GNAO1,MIR3935,AMFR,NUDT21,OGFOD1,BBS2,MT4,MT3,MT2A,MT1E,MT1M,MT1A,MT1B,MT1F,MT1G,MT1H,MT1IP,MT1X,NUP93,MIR138-2,SLC12A3,SLC12A3,MIR6863,HERPUD1,CETP,NLRC5,CPNE2,FAM192A,RSPRY1,ARL2BP,PLLP,CCL22,CX3CL1,CCL17,CIAPIN1,CIAPIN1,COQ9,COQ9,POLR2C,DOK4,CCDC102A,ADGRG5,ADGRG1,ADGRG3,DRC7,KATNB1,KIFC3,KIFC3,MIR6772,LOC388282,CNGB1,TEPP,ZNF319,USB1,MMP15,CFAP20,CSNK2A2,CCDC113,CCDC113,PRSS54,PRSS54,GINS3,NDRG4,SETD6,CNOT1,CNOT1,SNORA46,CNOT1,SNORA50A,SLC38A7,GOT2 | 0.174433 | 314.889 | 887 | 535.603 |
| 2 | 11 | 17373388 | 17867634 | NCR3LG1,KCNJ11,ABCC8,USH1C,OTOG,MYOD1,KCNC1,SERGEF | 0.172026 | 305.384 | 169 | 107.47 |
| 2 | 5 | 1.49E+08 | 150076452 | CSNK1A1,ARHGEF37,PPARGC1B,PPARGC1B,MIR378A,PDE6A,SLC26A2,TIGD6,TIGD6,HMGXB3,HMGXB3,CSF1R,PDGFRB,CDX1,SLC6A7,CAMK2A,ARSI,TCOF1,CD74,RPS14,NDST1,SYNPO,MYOZ3,RBM22 | 0.169892 | 289.995 | 303 | 192.461 |
| 2 | 2 | 2.16E+08 | 219320420 | FN1,MREG,PECR,TMEM169,XRCC5,MARCH4,SMARCAL1,RPL37A,IGFBP2,IGFBP5,TNP1,LINC01921,DIRC3,TNS1,TNS1,MIR6809,LOC105373878,RUFY4,CXCR2P1,RUFY4,CXCR2,CXCR1,ARPC2,GPBAR1,AAMP,PNKD,PNKD,TMBIM1,PNKD,TMBIM1,MIR6513,PNKD,CATIP-AS2,PNKD,CATIP-AS2,MIR6810,CATIP-AS2,CATIP,CATIP,CATIP,CATIP-AS1,SLC11A1,CTDSP1,CTDSP1,MIR26B,VIL1,USP37 | 0.169176 | 272.002 | 383 | 229.441 |

| | | | | | | | | |
|---|---|---|---|---|---|---|---|---|
| 2 | 17 | 5389372 | 10222514 | DERL2,MIS12,LOC728392,NLRP1,WSCD1,AIPL1,PIMREG,PITPNM3,KIAA0753,TXNDC17,TXNDC17,MED31,MED31,C17orf100,MIR4520-1,MIR4520-2,ALOX15P1,SLC13A5,XAF1,FBXO39,TEKT1,ALOX12-AS1,ALOX12-AS1,ALOX12,RNASEK,RNASEK-C17orf49,RNASEK-C17orf49,C17orf49,RNASEK-C17orf49,C17orf49,MIR497HG,MIR497HG,MIR195,MIR497HG,MIR497,BCL6B,SLC16A13,SLC16A11,CLEC10A,ASGR2,ASGR1,DLG4,DLG4,ACADVL,ACADVL,ACADVL,MIR324,DVL2,PHF23,GABARAP,CTDNEP1,ELP5,CLDN7,SLC2A4,YBX2,EIF5A,GPS2,NEURL4,ACAP1,KCTD11,TMEM95,TNK1,PLSCR3,TMEM256-PLSCR3,TMEM256-PLSCR3,TMEM256,NLGN2,SPEM1,SPEM2,TMEM102,FGF11,CHRNB1,ZBTB4,ZBTB4,SLC35G6,POLR2A,TNFSF12,TNFSF12-TNFSF13,TNFSF12-TNFSF13,TNFSF13,SENP3,SENP3-EIF4A1,SENP3-EIF4A1,EIF4A1,SENP3-EIF4A1,EIF4A1,SNORA48,SENP3-EIF4A1,EIF4A1,SNORD10,SENP3-EIF4A1,EIF4A1,SNORA67,CD68,LOC100996842,MPDU1,MPDU1,SOX15,FXR2,FXR2,SHBG,SHBG,SAT2,SHBG,ATP1B2,TP53,WRAP53,EFNB3,DNAH2,KDM6B,TMEM88,NAA38,NAA38,CYB5D1,CHD3,CHD3,SCARNA21,LINC02581,KCNAB3,TRAPPC1,CNTROB,GUCY2D,ALOX15B,ALOX12B,MIR4314,ALOXE3,HES7,PER1,VAMP2,TMEM107,MIR4521,BORCS6,AURKB,CTC1,PFAS,SLC25A35,RANGRF,SLC25A35,ARHGEF15,ODF4,LOC100128288,KRBA2,RPL26,RNF222,NDEL1,MYH10,CCDC42,SPDYE4,MFSD6L,PIK3R6,PIK3R5,NTN1,STX8,CFAP52,USP43,DHRS7C,GSG1L2,GLP2R,RCVRN,GAS7,MYH13 | 0.168791 | 296.274 | 1388 | 868.825 |

| | | | | | | | | |
|---|---|---|---|---|---|---|---|---|
| 2 | 17 | 70118901 | 81194710 | SOX9,SLC39A11,SSTR2,COG1,COG1,FAM104A,FAM104A,FAM104A,C17orf80,C17orf80,C17orf80,CPSF4L,CPSF4L,CDC42EP4,SDK2,LOC100134391,LINC00469,LINC00469,RPL38,TTYH2,DNAI2,KIF19,BTBD17,GPR142,GPRC5C,CD300A,CD300LB,CD300C,CD300LD,CD300LD,C17orf77,C17orf77,CD300E,RAB37,RAB37,CD300LF,SLC9A3R1,MIR3615,SLC9A3R1,NAT9,TMEM104,GRIN2C,FDXR,FADS6,USH1G,OTOP2,OTOP3,HID1,HID1,HID1-AS1,CDR2L,MRPL58,KCTD2,KCTD2,ATP5PD,SLC16A5,ARMC7,NT5C,JPT1,SUMO2,NUP85,GGA3,GGA3,MRPS7,MRPS7,MIF4GD,LOC100287042,LOC100287042,SLC25A19,SLC25A19,GRB2,MIR3678,TMEM94,TMEM94,MIR6785,CASKIN2,TSEN54,LLGL2,MYO15B,RECQL5,RECQL5,SMIM5,RECQL5,SMIM6,SAP30BP,ITGB4,GALK1,H3F3B,MIR4738,UNK,UNC13D,WBP2,TRIM47,TRIM65,MRPL38,FBF1,ACOX1,TEN1,TEN1-CDK3,TEN1-CDK3,CDK3,EVPL,SRP68,GALR2,ZACN,ZACN,EXOC7,EXOC7,EXOC7,MIR6868,FOXJ1,RNF157-AS1,RNF157,RNF157,UBALD2,QRICH2,PRPSAP1,SPHK1,UBE2O,AANAT,RHBDF2,CYGB,PRCD,PRCD,SNHG16,SNORD1C,SNHG16,SNHG16,SNORD1B,SNHG16,SNORD1A,ST6GALNAC2,ST6GALNAC1,MXRA7,JMJD6,METTL23,SRSF2,SRSF2,MIR636,SRSF2,MFSD11,MFSD11,LINC00868,MGAT5B,SNHG20,SEC14L1,SCARNA16,SNHG20,SEC14L1,SCARNA16,MIR6516,SEC14L1,LOC105371907,SEPT9,SEPT9,SEPT9,MIR4316,LINC01973,TNRC6C,TNRC6C,TNRC6C-AS1,TNRC6C-AS1,TMC6,TMC6,TMC8,TMC8,C17orf99,SYNGR2,TK1,AFMID,BIRC5,TMEM235,LINC01993,SOCS3,PGS1,PGS1,DNAH17,DNAH17,DNAH17,DNAH17-AS1,CYTH1,USP36,TIMP2,TIMP2,CEP295NL,LGALS3BP,CANT1,C1QTNF1-AS1,C1QTNF1,ENGASE,RBFOX3,MIR4739,LINC02078,E | 0.167557 | 292.553 | 2812 | 1748.61 |
| 2 | 16 | 68071894 | 69344822 | DUS2,NFATC3,ESRP2,ESRP2,MIR6773,PLA2G15,SLC7A6,SLC7A6,SLC7A6OS,SLC7A6OS,SLC7A6OS,PRMT7,PRMT7,SMPD3,ZFP90,CDH3,CDH1,TANGO6,HAS3,HAS3,CHTF8,CHTF8,CHTF8,UTP4,UTP4,SNTB2 | 0.166187 | 308.473 | 251 | 152.414 |
| 2 | 17 | 45678906 | 47919082 | NPEPPS,KPNB1,TBKBP1,TBX21,OSBPL7,MRPL10,LRRC46,SCRN2,SP6,SP2,SP2,SP2-AS1,PNPO,PRR15L,CDK5RAP3,COPZ2,COPZ2,MIR152,NFE2L1,CBX1,SNX11,SKAP1,SKAP1,MIR1203,HOXB1,HOXB2,HOXB2,HOXB-AS1,HOXB-AS1,HOXB3,HOXB3,HOXB3,HOXB4,HOXB3,MIR10A,HOXB-AS3,HOXB5,HOXB-AS3,HOXB6,HOXB7,HOXB8,HOXB9,MIR196A1,PRAC1,PRAC2,MIR3185,HOXB13,TTLL6,CALCOCO2,LOC105371814,ATP5MC1,LOC105371814,UBE2Z,UBE2Z,SNF8,GIP,IGF2BP1,B4GALNT2,GNGT2,ABI3,PHOSPHO1,FLJ40194,ZNF652,PHB,NGFR,NGFR,LOC100288866,NXPH3,SPOP,SLC35B1,FAM117A,KAT7,TAC4 | 0.164315 | 278.963 | 482 | 286.783 |

| | | | | | | | | |
|---|---|---|---|---|---|---|---|---|
| 2 | 2 | 2.32E+08 | 234402267 | ARMC9,ARMC9,MIR4777,B3GNT7,NCL,NCL,SNORA75,NCL,SNORD20,NCL,SNORD82,LINC00471,NMUR1,TEX44,PTMA,PDE6D,COPS7B,MIR1471,NPPC,DIS3L2,ALPP,ALPPL2,ALPI,ECEL1,PRSS56,CHRND,CHRNG,TIGD1,TIGD1,MIR5001,EIF4E2,EIF4E2,EFHD1,GIGYF2,GIGYF2,KCNJ13,SNORC,NGEF,NGEF,LOC101928881,LOC101928881,NEU2,INPP5D,ATG16L1,ATG16L1,SCARNA5,ATG16L1,SCARNA6,SAG,DGKD,USP40 | 0.16404 | 286.723 | 451 | 267.867 |
| 2 | 22 | 16050500 | 20508931 | DUXAP8,BMS1P22,CCT8L2,ANKRD62P1-PARP4P3,XKR3,GAB4,IL17RA,TMEM121B,LINC01664,HDHD5,HDHD5,HDHD5-AS1,ADA2,CECR2,SLC25A18,SLC25A18,LOC101929372,ATP6V1E1,BCL2L13,BID,BID,MIR3198-1,MICAL3,MICAL3,MIR648,PEX26,TUBA8,USP18,GGT3P,DGCR6,PRODH,DGCR2,DGCR2,DGCR11,ESS2,TSSK2,ESS2,GSC2,SLC25A1,CLTCL1,HIRA,MRPL40,C22orf39,UFD1,CDC45,CLDN5,SEPT5,SEPT5,SEPT5-GP1BB,SEPT5-GP1BB,GP1BB,TBX1,GNB1L,GNB1L,RTL10,TXNRD2,TXNRD2,COMT,COMT,COMT,MIR4761,ARVCF,TANGO2,MIR185,TANGO2,DGCR8,MIR3618,DGCR8,MIR1306,DGCR8,TRMT2A,TRMT2A,MIR6816,TRMT2A,RANBP1,RANBP1,RANBP1,SNORA77B,ZDHHC8,CCDC188,LINC00896,RTN4R,RTN4R,MIR1286,DGCR6L,LOC101927859,TMEM191B,LOC101927859,PI4KAP1 | 0.159145 | 286.072 | 689 | 400.603 |
| 2 | X | 9000484 | 10437961 | FAM9B,TBL1X,GPR143,SHROOM2,CLDN34,WWC3,CLCN4,MID1 | 0.152046 | 310.468 | 124 | 73.5813 |
| 2 | 12 | 1.23E+08 | 123636581 | HCAR2,HCAR3,HCAR1,DENR,CCDC62,HIP1R,VPS37B,ABCB9,OGFOD2,ARL6IP4,PITPNM2,PITPNM2,MIR4304 | 0.148711 | 289.099 | 144 | 89.2229 |
| 2 | 17 | 39135144 | 41199711 | KRT40,KRTAP3-3,KRTAP3-2,KRTAP3-1,KRTAP1-5,KRTAP1-4,KRTAP1-3,KRTAP1-1,KRTAP2-3,KRTAP2-4,KRTAP4-7,KRTAP4-8,KRTAP4-8,KRTAP4-9,KRTAP4-8,KRTAP4-11,KRTAP4-6,KRTAP4-5,KRTAP4-4,KRTAP4-3,KRTAP4-2,KRTAP4-1,KRTAP9-1,KRTAP9-3,KRTAP9-9,KRTAP9-9,KRTAP9-4,KRTAP9-9,KRTAP9-7,KRTAP29-1,KRTAP16-1,KRTAP17-1,KRT33A,KRT33B,KRT34,KRT31,KRT37,KRT38,KRT32,KRT35,KRT36,KRT13,KRT15,KRT19,KRT9,KRT14,KRT16,KRT17,EIF1,GAST,HAP1,JUP,P3H4,FKBP10,NT5C3B,NT5C3B,KLHL10,KLHL10,KLHL11,ACLY,TTC25,CNP,CNP,DNAJC7,DNAJC7,NKIRAS2,ZNF385C,DHX58,KAT2A,HSPB9,RAB5C,KCNH4,HCRT,GHDC,STAT5B,STAT5A,STAT3,CAVIN1,ATP6V0A1,ATP6V0A1,MIR548AT,ATP6V0A1,MIR5010,NAGLU,LOC108783654,HSD17B1,COASY,MLX,MLX,PSMC3IP,PSMC3IP,RETREG3,TUBG1,TUBG2,PLEKHH3,CCR10,CNTNAP1,EZH1,EZH1,MIR6780A,RAMP2-AS1,RAMP2-AS1,RAMP2,RAMP2,VPS25,WNK4,COA3,CNTD1,CNTD1,BECN1,BECN1,PSME3,AOC2,AOC3,AOC4P,LINC00671,G6PC,AARSD1,PTGES3L-AARSD1,PTGES3L-AARSD1,PTGES3L,RUNDC1,RPL27,IFI35,VAT1,RND2,BRCA1 | 0.148465 | 301.315 | 880 | 528.735 |

| | | | | | | | | |
|---|---|---|---|---|---|---|---|---|
| 2 | 2 | 72968392 | 73956505 | EXOC6B,SPR,EMX1,SFXN5,RAB11FIP5,NOTO,SMYD5,PRADC1,CCT7,FBXO41,EGR4,ALMS1,NAT8,ALMS1P1,NAT8B | 0.147194 | 288.555 | 207 | 124.164 |
| 2 | 17 | 41343382 | 45199997 | NBR1,TMEM106A,LINC00910,ARL4D,MIR2117HG,MIR2117,DHX8,DHX8,ETV4,ETV4,MEOX1,SOST,DUSP3,C17orf105,MPP3,CD300LG,MPP2,FAM215A,LINC01976,PPY,PYY,NAGS,TMEM101,LSM12,G6PC3,HDAC5,HDAC5,LOC105371789,C17orf53,ASB16,ASB16,ASB16-AS1,TMUB2,ATXN7L3,UBTF,UBTF,MIR6782,SLC4A1,RUNDC3A,SLC25A39,GRN,FAM171A2,ITGA2B,GPATCH8,FZD2,MEIOC,CCDC43,DBF4B,ADAM11,GJC1,HIGD1B,HIGD1B,EFTUD2,EFTUD2,CCDC103,FAM187A,GFAP,KIF18B,KIF18B,MIR6783,C1QL1,DCAKD,NMT1,PLCD3,ACBD4,HEXIM1,HEXIM2,HEXIM2,LOC105371795,FMNL1,MAP3K14-AS1,SPATA32,MAP3K14-AS1,MAP3K14,MAP3K14,ARHGAP27,PLEKHM1,PLEKHM1,MIR4315-1,MIR4315-2,LRRC37A4P,MAPK8IP1P2,LINC02210,LINC02210-CRHR1,LINC02210-CRHR1,CRHR1,MAPT-AS1,SPPL2C,MAPT,MAPT,STH,KANSL1,KANSL1,KANSL1-AS1,ARL17B,ARL17B,LRRC37A,ARL17A,ARL17B,ARL17A,NSFP1,ARL17A,LRRC37A2,ARL17B,ARL17A,ARL17B,NSF,NSFP1,NSF,WNT3,WNT9B,GOSR2,MIR5089,RPRML,CDC27 | 0.146151 | 285.416 | 886 | 535.926 |
| 2 | 2 | 71662493 | 72719630 | DYSF,CYP26B1,EXOC6B | 0.141924 | 290.364 | 91 | 54.511 |
| 2 | 1 | 1.1E+08 | 110091506 | TAF13,TMEM167B,SCARNA2,C1orf194,KIAA1324,SARS,CELSR2,PSRC1,MYBPHL,SORT1,PSMA5,SYPL2,ATXN7L2,CYB561D1,AMIGO1,GPR61,GNAI3 | 0.139571 | 311.314 | 192 | 123.294 |
| 2 | 6 | 617705 | 4021762 | EXOC2,EXOC2,HUS1B,LINC01622,FOXQ1,FOXF2,FOXF2,MIR6720,FOXC1,GMDS,LINC01600,MYLK4,MYLK4,WRNIP1,WRNIP1,SERPINB1,MIR4645,LOC101927730,SERPINB9,SERPINB6,NQO2,RIPK1,BPHL,TUBB2A,TUBB2B,LOC100422781,PSMG4,SLC22A23,SLC22A23,LOC643327,PXDC1,FAM50B,PRPF4B | 0.138761 | 243.577 | 217 | 125.826 |
| 2 | 20 | 488972 | 1209245 | CSNK2A1,TCF15,SRXN1,SCRT2,SLC52A3,FAM110A,ANGPT4,RSPO4,PSMF1,TMEM74B,C20orf202,RAD21L1 | 0.13614 | 249.012 | 75 | 43.4253 |
| 2 | 7 | 97599696 | 99143812 | OCM2,LMTK2,BHLHA15,TECPR1,BRI3,BRI3,BAIAP2L1,BAIAP2L1,NPTX2,TMEM130,TRRAP,TRRAP,MIR3609,SCARNA28,TRRAP,SCARNA28,LOC101927550,SMURF1,SMURF1,KPNA7,MYH16,ARPC1A,ARPC1B,PDAP1,BUD31,BUD31,PTCD1,ATP5MF-PTCD1,PTCD1,ATP5MF-PTCD1,ATP5MF-PTCD1,CPSF4,ATP5MF-PTCD1,ATP5MF,ZNF789,ZNF789,ZNF394,ZNF394,ZKSCAN5 | 0.135081 | 325.951 | 336 | 204.703 |
| 2 | 11 | 46894686 | 47639839 | LRP4-AS1,LRP4,LRP4,C11orf49,ARFGAP2,PACSIN3,PACSIN3,MIR6745,DDB2,ACP2,ACP2,NR1H3,NR1H3,MADD,MYBPC3,SPI1,SLC39A13,PSMC3,RAPSN,CELF1,PTPMT1,PTPMT1,KBTBD4,KBTBD4,KBTBD4,NDUFS3,NDUFS3,FAM180B,C1QTNF4 | 0.132994 | 296.482 | 258 | 164.558 |
| 2 | 15 | 43821725 | 43904259 | MAP1A,PPIP5K1,CKMT1B,STRC | 0.132655 | 325.251 | 71 | 40.4593 |

| | | | | | | | | |
|---|---|---|---|---|---|---|---|---|
| 2 | 15 | 98512370 | 99795741 | ARRDC4,FAM169B,IRAIN,IGF1R,IGF1R,IGF1R,MIR4714,PGPEP1L,SYNM,TTC23 | 0.132398 | 260.748 | 93 | 56.0851 |
| 2 | 3 | 1.7E+08 | 172471794 | SKIL,CLDN11,SLC7A14,LOC101928583,LOC101928583,RPL22L1,EIF5A2,SLC2A2,TNIK,TNIK,MIR569,PLD1,TMEM212,FNDC3B,GHSR,TNFSF10,NCEH1 | 0.132169 | 295.198 | 215 | 125.884 |
| 2 | 20 | 34326771 | 35526364 | RBM39,PHF20,SCAND1,CNBD2,NORAD,EPB41L1,EPB41L1,LOC100130373,AAR2,DLGAP4,DLGAP4,DLGAP4-AS1,DLGAP4-AS1,MYL9,TGIF2,TGIF2-C20orf24,TGIF2-C20orf24,C20orf24,SLA2,NDRG3,DSN1,SOGA1,TLDC2,TLDC2,SAMHD1,SAMHD1 | 0.130265 | 284.763 | 222 | 131.433 |
| 2 | 1 | 1.5E+08 | 150432796 | BOLA1,SV2A,SF3B4,MTMR11,OTUD7B,VPS45,PLEKHO1,ANP32E,CA14,APH1A,C1orf54,CIART,MRPS21,PRPF3,RPRD2 | 0.129293 | 286.824 | 172 | 102.76 |
| 2 | 11 | 43923824 | 46766171 | ALKBH3,ALKBH3-AS1,C11orf96,ACCSL,ACCS,EXT2,ALX4,CD82,TSPAN18,TP53I11,PRDM11,SYT13,CHST1,DKFZp779M0652,SLC35C1,CRY2,MAPK8IP1,C11orf94,PEX16,LARGE2,PHF21A,CREB3L1,DGKZ,DGKZ,MIR4688,MDK,CHRM4,AMBRA1,AMBRA1,MIR3160-1,MIR3160-2,HARBI1,ATG13,ARHGAP1,ZNF408,F2,CKAP5 | 0.127146 | 269.333 | 405 | 249.046 |
| 2 | 15 | 1E+08 | 101013271 | MEF2A,LYSMD4,DNM1P46,LOC400464,ADAMTS17,SPATA41,CERS3-AS1,CERS3,CERS3 | 0.125428 | 239.536 | 80 | 42.1556 |
| 2 | 17 | 61895009 | 62461689 | DDX42,FTSJ3,FTSJ3,PSMC5,PSMC5,SMARCD2,CSH2,GH2,CSH1,CSHL1,GH1,CD79B,SCN4A,PRR29-AS1,PRR29,PRR29,PRR29,ICAM2,ICAM2,ERN1,SNHG25,SNORD104,SNORA50C,TEX2,PECAM1,MILR1 | 0.124233 | 300.058 | 185 | 112.352 |

| | | | | | | | | |
|---|---|---|---|---|---|---|---|---|
| 2 | 5 | 1.39E+08 | 141353227 | PAIP2,PAIP2,SLC23A1,SLC23A1,MZB1,PROB1,SPATA24,DNAJC18,ECSCR,TMEM173,UBE2D2,CXXC5,PSD2,NRG2,PURA,IGIP,CYSTM1,PFDN1,HBEGF,SLC4A9,ANKHD1,ANKHD1-EIF4EBP3,ANKHD1-EIF4EBP3,ANKHD1-EIF4EBP3,EIF4EBP3,SRA1,APBB3,APBB3,MIR6831,SLC35A4,CD14,TMCO6,NDUFA2,IK,MIR3655,IK,WDR55,DND1,HARS,HARS,HARS2,HARS2,ZMAT2,PCDHA1,PCDHA1,PCDHA2,PCDHA1,PCDHA2,PCDHA3,PCDHA1,PCDHA2,PCDHA3,PCDHA4,PCDHA1,PCDHA2,PCDHA3,PCDHA4,PCDHA5,PCDHA1,PCDHA2,PCDHA3,PCDHA4,PCDHA5,PCDHA6,PCDHA1,PCDHA2,PCDHA3,PCDHA4,PCDHA5,PCDHA6,PCDHA7,PCDHA1,PCDHA2,PCDHA3,PCDHA4,PCDHA5,PCDHA6,PCDHA7,PCDHA8,PCDHA1,PCDHA2,PCDHA3,PCDHA4,PCDHA5,PCDHA6,PCDHA7,PCDHA8,PCDHA9,PCDHA1,PCDHA2,PCDHA3,PCDHA4,PCDHA5,PCDHA6,PCDHA7,PCDHA8,PCDHA9,PCDHA10,PCDHA1,PCDHA2,PCDHA3,PCDHA4,PCDHA5,PCDHA6,PCDHA7,PCDHA8,PCDHA9,PCDHA10,PCDHA11,PCDHA1,PCDHA2,PCDHA3,PCDHA4,PCDHA5,PCDHA6,PCDHA7,PCDHA8,PCDHA9,PCDHA10,PCDHA11,PCDHA12,PCDHA1,PCDHA2,PCDHA3,PCDHA4,PCDHA5,PCDHA6,PCDHA7,PCDHA8,PCDHA9,PCDHA10,PCDHA11,PCDHA12,PCDHA13,PCDHA1,PCDHA2,PCDHA3,PCDHA4,PCDHA5,PCDHA6,PCDHA7,PCDHA8,PCDHA9,PCDHA10,PCDHA11,PCDHA12,PCDHA13,PCDHAC1,PCDHA1,PCDHA2,PCDHA3,PCDHA4,PCDHA5,PCDHA6,PCDHA7,PCDHA8,PCDHA9,PCDHA10,PCDHA11,PCDHA12,PCDHA13,PCDHAC1,PCDHAC2,LOC101926905,PCDHB1,PCDHB2,PCDHB3,PCDHB4,PCDHB5,PCDHB6,PCDHB17P,PCDHB7,PCDHB8,PCDHB16,PCDHB9,PCDHB10,PCDHB11,PCDHB12,PCDHB13,PCDHB14,PCDHB18P,PCDHB19P,PCDHB15,SLC25A2,TAF7,PCDHGA1,PCDHGA1,PCDHGA2,PCDHGA1,PCDHGA2,PCDHGA3,PCDHGA1,PCDHGA2,PCDHGA3,PCD | 0.123332 | 314.291 | 981 | 626.834 |
| 2 | 17 | 3397566 | 3923161 | SPATA22,ASPA,TRPV3,TRPV1,SHPK,CTNS,TAX1BP3,P2RX5-TAX1BP3,P2RX5-TAX1BP3,EMC6,P2RX5-TAX1BP3,P2RX5-TAX1BP3,P2RX5,ITGAE,ITGAE,HASPIN,NCBP3,CAMKK1,P2RX1,ATP2A3,ZZEF1 | 0.122646 | 290.038 | 209 | 131.297 |
| 2 | 13 | 1.15E+08 | 115109378 | CDC16,CDC16,MIR548AR,MIR4502,UPF3A,CHAMP1 | 0.121339 | 388.901 | 39 | 23.5814 |

| | | | | | | | | |
|---|---|---|---|---|---|---|---|---|
| 2 | 8 | 10500 | 13425439 | ZNF596,FAM87A,FBXO25,TDRP,ERICH1,DLGAP2,DLGAP2,DLGAP2-AS1,CLN8,MIR3674,MIR596,ARHGEF10,KBTBD11,MYOM2,CSMD1,MCPH1,MCPH1,ANGPT2,MCPH1,MCPH1-AS1,AGPAT5,AGPAT5,MIR4659A,MIR4659B,XKR5,XKR5,GS1-24F4.2,DEFB1,DEFA6,DEFA4,DEFA5,DEFB4B,DEFB103B,DEFB103A,SPAG11B,DEFB104A,DEFB104B,DEFB106B,DEFB106A,DEFB105B,DEFB105A,DEFB107A,DEFB107B,PRR23D1,PRR23D2,PRR23D2,PRR23D1,SPAG11B,SPAG11A,SPAG11A,DEFB4A,PRAG1,CLDN23,MFHAS1,ERI1,ERI1,MIR4660,PPP1R3B,TNKS,TNKS,MIR597,MIR124-1,MSRA,PRSS55,RP1L1,MIR4286,C8orf74,SOX7,SOX7,LOC102723313,LOC102723313,PINX1,PINX1,PINX1,MIR1322,XKR6,XKR6,MIR598,XKR6,LOC101929269,MTMR9,SLC35G5,TDH,LOC100129129,FAM167A-AS1,FAM167A,FAM167A,BLK,GATA4,C8orf49,NEIL2,FDFT1,CTSB,DEFB136,DEFB135,DEFB134,LOC729732,MIR5692A1,MIR5692A2,LONRF1,LONRF1,MIR3926-1,MIR3926-2,KIAA1456,DLC1,DLC1,C8orf48 | 0.119659 | 276.852 | 873 | 503.029 |
| 2 | 10 | 70948966 | 71912432 | SUPV3L1,LOC101928994,HKDC1,HKDC1,HK1,TACR2,TSPAN15,NEUROG3,FAM241B,COL13A1,H2AFY2,AIFM2,TYSND1,SAR1A | 0.119256 | 262.968 | 172 | 103.122 |
| 2 | 15 | 42966526 | 43109384 | STARD9,CDAN1,TTBK2 | 0.114553 | 274.458 | 112 | 74.9486 |
| 2 | 8 | 39695533 | 42202626 | ADAM2,IDO1,IDO2,TCIM,ZMAT4,SFRP1,SFRP1,MIR548AO,GOLGA7,GINS4,GINS4,LOC102723729,GPAT4,NKX6-3,ANK1,ANK1,MIR486-1,MIR486-2,KAT6A,AP3M2,PLAT,IKBKB,POLB | 0.11301 | 269.823 | 255 | 153.95 |
| 2 | 17 | 6003 | 3343642 | DOC2B,RPH3AL,RPH3AL,LOC100506388,LOC105371430,C17orf97,RFLNB,VPS53,FAM57A,GEMIN4,GLOD4,GLOD4,MRM3,MRM3,NXN,TIMM22,ABR,ABR,MIR3183,BHLHA9,TUSC5,YWHAE,CRK,MYO1C,INPP5K,PITPNA,SLC43A2,SCARF1,RILP,PRPF8,TLCD2,MIR22HG,MIR22HG,MIR22,WDR81,SERPINF2,SERPINF1,SMYD4,RPA1,RTN4RL1,DPH1,DPH1,OVCA2,HIC1,SMG6,SRR,SRR,TSR1,TSR1,TSR1,SNORD91B,TSR1,SNORD91A,SGSM2,MNT,LOC284009,METTL16,PAFAH1B1,CLUH,CLUH,MIR6776,CCDC92B,MIR1253,RAP1GAP2,RAP1GAP2,LOC101927911,OR1D5,OR1D2,OR1G1,OR1A2,OR1A1,OR1D4,OR3A2,OR3A1,OR3A4P,OR1E1,OR3A3,OR1E2,SPATA22 | 0.112072 | 276.039 | 685 | 412.981 |
| 2 | 15 | 77240775 | 78730775 | RCN2,PSTPIP1,TSPAN3,PEAK1,HMG20A,LINGO1,LINGO1,LINGO1-AS1,LOC645752,TBC1D2B,SH2D7,CIB2,IDH3A,ACSBG1,DNAJA4,WDR61,CRABP1,IREB2 | 0.111849 | 273.761 | 206 | 121.071 |

| | | | | | | | | |
|---|---|---|---|---|---|---|---|---|
| 2 | 1 | 35881001 | 43738486 | ZMYM4,KIAA0319L,NCDN,TFAP2E,PSMB2,C1orf216,CLSPN,AGO4,AGO1,AGO3,TEKT2,ADPRHL2,COL8A2,TRAPPC3,MAP7D1,THRAP3,SH3D21,EVA1B,STK40,LSM10,OSCP1,MRPS15,CSF3R,GRIK3,MIR4255,ZC3H12A,ZC3H12A,MIR6732,MEAF6,MEAF6,MIR5581,SNIP1,DNALI1,GNL2,RSPO1,C1orf109,CDCA8,EPHA10,MANEAL,YRDC,YRDC,C1orf122,C1orf122,MTF1,INPP5B,SF3A3,FHL3,UTP11,POU3F1,MIR3659,LINC01343,RRAGC,MYCBP,GJA9-MYCBP,MYCBP,GJA9-MYCBP,LOC105378663,GJA9-MYCBP,LOC105378663,GJA9-MYCBP,LOC105378663,GJA9,GJA9-MYCBP,GJA9,RHBDL2,AKIRIN1,NDUFS5,MACF1,MACF1,KIAA0754,BMP8A,BMP8A,OXCT2P1,BMP8A,PPIEL,PABPC4,PABPC4,LOC101929516,PABPC4,LOC101929516,SNORA55,HEYL,NT5C1A,HPCAL4,PPIE,PPIE,BMP8B,BMP8B,BMP8B,OXCT2,TRIT1,MYCL,MFSD2A,CAP1,PPT1,RLF,TMCO2,ZMPSTE24,COL9A2,SMAP2,ZFP69B,ZFP69,EXO5,ZNF684,RIMS3,NFYC-AS1,NFYC,NFYC,NFYC,MIR30E,NFYC,MIR30C1,KCNQ4,CITED4,CTPS1,SLFNL1-AS1,SLFNL1,SLFNL1-AS1,SCMH1,SCMH1,FOXO6,EDN2,HIVEP3,GUCA2B,GUCA2A,FOXJ3,RIMKLA,ZMYND12,CCDC30,PPCS,CCDC30,PPIH,YBX1,CLDN19,P3H1,C1orf50,TMEM269,SVBP,SVBP,ERMAP,ERMAP,ZNF691,SLC2A1,FAM183A,EBNA1BP2,EBNA1BP2,MIR6733,EBNA1BP2,CFAP57,CFAP57,TMEM125 | 0.111251 | 281.416 | 1400 | 853.652 |
| 2 | 12 | 1.11E+08 | 111923713 | TCTN1,TCTN1,HVCN1,HVCN1,PPP1CC,CCDC63,MYL2,LINC01405,CUX2,FAM109A,SH2B3,ATXN2 | 0.109783 | 239.375 | 126 | 75.0072 |
| 2 | 4 | 1.24E+08 | 126412947 | SPATA5,SPRY1,ANKRD50,FAT4 | 0.10631 | 319.753 | 114 | 74.8031 |
| 2 | 20 | 29420069 | 34288865 | FRG1BP,FRG1DP,FRG1BP,DEFB115,DEFB116,DEFB118,DEFB119,DEFB121,DEFB123,DEFB124,REM1,HM13,HM13,HM13-AS1,ID1,MIR3193,COX4I2,BCL2L1,BCL2L1,ABALON,TPX2,MYLK2,FOXS1,DUSP15,DUSP15,TTLL9,TTLL9,PDRG1,XKR7,CCM2L,HCK,TM9SF4,TSPY26P,PLAGL2,POFUT1,POFUT1,MIR1825,KIF3B,ASXL1,NOL4L,NOL4L,LOC101929698,NOL4L-DT,C20orf203,COMMD7,DNMT3B,MAPRE1,EFCAB8,SUN5,BPIFB2,BPIFB6,BPIFB3,BPIFB4,BPIFA2,BPIFA3,BPIFA1,BPIFB1,CDK5RAP1,SNTA1,CBFA2T2,NECAB3,NECAB3,C20orf144,NECAB3,ACTL10,E2F1,PXMP4,ZNF341,ZNF341,ZNF341-AS1,CHMP4B,RALY,MIR4755,RALY,EIF2S2,ASIP,AHCY,ITCH,ITCH,MIR644A,DYNLRB1,MAP1LC3A,PIGU,TP53INP2,NCOA6,GGT7,ACSS2,GSS,MYH7B,MYH7B,MIR499A,MIR499B,TRPC4AP,EDEM2,MMP24-AS1-EDEM2,MMP24-AS1-EDEM2,MMP24-AS1-EDEM2,PROCR,MMP24-AS1-EDEM2,MMP24,EIF6,FAM83C,UQCC1,GDF5OS,GDF5OS,GDF5,GDF5,GDF5,MIR1289-1,CEP250,C20orf173,ERGIC3,FER1L4,SPAG4,CPNE1,CPNE1,RBM12,NFS1,NFS1,ROMO1,ROMO1 | 0.106039 | 284.092 | 1094 | 661.882 |

| | | | | | | | | |
|---|---|---|---|---|---|---|---|---|
| 2 | 12 | 1.33E+08 | 133841395 | POLE,PXMP2,PGAM5,ANKLE2,GOLGA3,CHFR,ZNF605,ZNF26,ZNF84,ZNF140,ZNF891,ZNF10,ZNF268,ANHX | 0.103126 | 269.826 | 219 | 131.463 |
| 2 | 2 | 1.79E+08 | 179481718 | MIR548N,TTN-AS1,TTN | 0.101907 | 322.301 | 253 | 182.516 |
| 2 | 1 | 45811564 | 47746843 | TESK2,CCDC163,MMACHC,PRDX1,AKR1A1,NASP,CCDC17,GPBP1L1,TMEM69,TMEM69,IPP,IPP,MAST2,PIK3R3,LOC110117498-PIK3R3,LOC110117498-PIK3R3,TSPAN1,LOC110117498,TSPAN1,POMGNT1,POMGNT1,LURAP1,RAD54L,LRRC41,UQCRH,NSUN4,FAAH,DMBX1,MKNK1-AS1,KNCN,MKNK1-AS1,MKNK1,MKNK1,MOB3C,ATPAF1,TEX38,EFCAB14-AS1,EFCAB14,EFCAB14,CYP4B1,CYP4Z2P,CYP4A11,CYP4X1,CYP4Z1,CYP4A22,PDZK1IP1,TAL1,STIL | 0.100653 | 296.764 | 450 | 268.274 |
| 2 | 1 | 1.57E+08 | 159504300 | ARHGEF11,ETV3L,ETV3,FCRL5,FCRL4,FCRL3,FCRL2,FCRL1,CD5L,KIRREL1,CD1D,CD1A,CD1C,CD1B,CD1E,OR10T2,OR10K2,OR10K1,OR10R2,OR6Y1,OR6P1,OR10X1,OR10Z1,SPTA1,OR6K2,OR6K3,OR6K6,OR6N1,OR6N2,MNDA,PYHIN1,IFI16,AIM2,CADM3,CADM3,CADM3-AS1,ACKR1,FCER1A,OR10J3,OR10J4,OR10J1 | 0.100444 | 300.521 | 423 | 251.418 |
| 2 | 2 | 24916050 | 26432874 | NCOA1,PTRHD1,PTRHD1,CENPO,CENPO,CENPO,ADCY3,ADCY3,DNAJC27,EFR3B,POMC,DNMT3A,DNMT3A,MIR1301,DTNB,ASXL2,KIF3C,RAB10,GAREM2,HADHA | 0.0991371 | 274.425 | 247 | 145.593 |
| 2 | 3 | 1.25E+08 | 126328168 | SNX4,OSBPL11,ALG1L,ROPN1B,SLC41A3,ALDH1L1,ALDH1L1-AS1,ALDH1L1,ALDH1L1,ALDH1L1-AS2,KLF15,CFAP100,ZXDC,UROC1,CHST13,CHST13,C3orf22,C3orf22,TXNRD3NB,TXNRD3NB,TXNRD3,TXNRD3 | 0.0989365 | 275.029 | 160 | 97.1466 |
| 2 | 14 | 95598847 | 96756205 | DICER1,DICER1,MIR3173,CLMN,SYNE3,SNHG10,SCARNA13,GLRX5,TCL6,TCL1B,TCL1A,C14orf132,BDKRB2,BDKRB1,ATG2B | 0.0977827 | 258.335 | 104 | 63.6664 |
| 2 | 17 | 16734782 | 19681064 | KRT16P2,TBC1D27P,TNFRSF13B,LINC02090,MPRIP,PLD6,FLCN,COPS3,NT5M,MED9,RASD1,PEMT,RAI1,SMCR5,RAI1,RAI1,SREBF1,SREBF1,SREBF1,MIR6777,SREBF1,MIR33B,TOM1L2,DRC3,ATPAF2,GID4,DRG2,MYO15A,ALKBH5,LLGL1,FLII,MIEF2,TOP3A,SMCR8,SHMT1,EVPLL,LINC02076,LGALS9C,CCDC144B,TBC1D28,ZNF286B,ZNF286B,FOXO3B,TRIM16L,FBXW10,TVP23B,PRPSAP2,SLC5A10,SLC5A10,FAM83G,GRAP,LOC388436,LOC79999,GRAPL,GRAPL,LOC388436,LOC79999,EPN2,EPN2,B9D1,B9D1,B9D1,MIR1180,MAPK7,MFAP4,RNF112,SLC47A1,SLC47A1,SNORA59B,SNORA59A,ALDH3A2,SLC47A2,ALDH3A1,ULK2 | 0.0963233 | 290.424 | 769 | 463.884 |
| 2 | 1 | 52992422 | 55549079 | GPX7,SHISAL2A,COA7,ZYG11B,ZYG11A,ECHDC2,SCP2,SCP2,MIR1273F,SCP2,MIR1273G,PODN,SLC1A7,CPT2,C1orf123,MAGOH,LRP8,DMRTB1,GLIS1,NDC1,YIPF1,DIO1,HSPB11,LRRC42,LDLRAD1,TMEM59,TCEANC2,MIR4781,TCEANC2,CDCP2,CYB5RL,MRPL37,SSBP3,SSBP3,SSBP3-AS1,ACOT11,ACOT11,FAM151A,MROH7,MROH7-TTC4,MROH7-TTC4,MROH7-TTC4,TTC4,PARS2,TTC22,LEXM,DHCR24,TMEM61,BSND,PCSK9,USP24 | 0.0957609 | 297.334 | 470 | 281.189 |

| | | | | | | | | |
|---|---|---|---|---|---|---|---|---|
| 2 | 17 | 36384844 | 38547940 | LOC440434,MRPL45,GPR179,SOCS7,ARHGAP23,SRCIN1,EPOP,MIR4734,MLLT6,MLLT6,MIR4726,CISD3,CISD3,PCGF2,PCGF2,LOC100287808,PSMB3,PIP4K2B,CWC25,MIR4727,C17orf98,RPL23,RPL23,SNORA21,LASP1,LASP1,MIR6779,FBXO47,LOC100131347,LOC100131347,PLXDC1,PLXDC1,ARL5C,CACNB1,RPL19,STAC2,FBXL20,MED1,CDK12,NEUROD2,PPP1R1B,STARD3,TCAP,PNMT,PGAP3,ERBB2,ERBB2,MIR4728,MIEN1,GRB7,IKZF3,ZPBP2,GSDMB,ORMDL3,LRRC3C,GSDMA,PSMD3,CSF3,MED24,MED24,MIR6884,MED24,SNORD124,THRA,THRA,NR1D1,NR1D1,MSL1,CASC3,CASC3,MIR6866,RAPGEFL1,RAPGEFL1,MIR6867,WIPF2,CDC6,RARA,RARA,RARA-AS1,GJD3,TOP2A | 0.0922874 | 283.392 | 698 | 420.427 |
| 2 | 1 | 1.43E+08 | 147083824 | NBPF20,NBPF19,NBPF8,NBPF9,NBPF20,NBPF19,NBPF9,LOC100996724,PDE4DIP,NBPF20,NBPF19,NBPF9,PDE4DIP,NBPF20,NBPF19,NBPF9,SEC22B,NBPF20,NBPF19,NBPF9,NBPF20,NBPF19,NBPF9,NOTCH2NL,NBPF20,NBPF19,NBPF10,HFE2,NBPF20,NBPF19,NBPF10,TXNIP,NBPF20,NBPF19,NBPF10,POLR3GL,NBPF20,NBPF19,NBPF10,ANKRD34A,NBPF20,NBPF19,NBPF10,LIX1L,NBPF20,NBPF19,NBPF10,LIX1L,LIX1L-AS1,NBPF20,NBPF19,NBPF10,LIX1L-AS1,RBM8A,NBPF20,NBPF19,NBPF10,RBM8A,NBPF20,NBPF19,NBPF10,RBM8A,GNRHR2,NBPF20,NBPF19,NBPF10,GNRHR2,NBPF20,NBPF19,NBPF10,PEX11B,NBPF20,NBPF19,NBPF10,ITGA10,NBPF20,NBPF19,NBPF10,ANKRD35,NBPF20,NBPF19,NBPF10,PIAS3,NBPF20,NBPF19,NBPF10,PIAS3,MIR6736,NBPF20,NBPF19,NBPF10,PIAS3,NUDT17,NBPF20,NBPF19,NBPF10,NUDT17,NBPF20,NBPF19,NBPF10,POLR3C,NBPF20,NBPF19,NBPF10,RNF115,NBPF20,NBPF19,NBPF10,CD160,NBPF20,NBPF19,NBPF10,PDZK1,NBPF20,NBPF19,NBPF10,GPR89A,NBPF20,NBPF19,NBPF10,NBPF25P,NBPF20,NBPF19,NBPF10,NBPF25P,PDE4DIPP1,NBPF19,LOC728989,NBPF19,PRKAB2,NBPF19,CHD1L,PDIA3P1,NBPF19,CHD1L,FMO5,NBPF19,CHD1L,NBPF19,BCL9 | 0.0880178 | 346.166 | 357 | 205.498 |
| 2 | 2 | 2.39E+08 | 242182021 | UBE2F,UBE2F-SCLY,UBE2F-SCLY,SCLY,ESPNL,KLHL30,ERFE,ILKAP,LOC151174,LOC643387,HES6,PER2,TRAF3IP1,ASB1,TWIST2,HDAC4,HDAC4,MIR4440,HDAC4,MIR4269,HDAC4,MIR2467,LOC101928111,LOC150935,NDUFA10,NDUFA10,MIR4786,OR6B2,OR6B3,COPS9,OTOS,GPC1,GPC1,PP14571,GPC1,PP14571,MIR149,ANKMY1,ANKMY1,DUSP28,DUSP28,RNPEPL1,CAPN10,GPR35,AQP12B,LOC285191,AQP12A,KIF1A,AGXT,C2orf54,CROCC2,CROCC2,LOC200772,SNED1,SNED1,MTERF4,MTERF4,PASK,PASK,PPP1R7,PPP1R7,ANO7,HDLBP | 0.08683 | 258.254 | 578 | 352.947 |

| | | | | | | | | |
|---|---|---|---|---|---|---|---|---|
| 2 | 15 | 84789809 | 91290019 | EFL1P1,LOC440300,GOLGA2P7,ZSCAN2,SCAND2P,WDR73,NMB,SEC11A,ZNF592,ALPK3,SLC28A1,PDE8A,AKAP13,KLHL25,KLHL25,MIR1276,MIR548AP,AGBL1,AGBL1,LOC102724452,NTRK3,NTRK3,NTRK3-AS1,MRPL46,MRPS11,DET1,MIR1179,MIR7-2,MIR3529,AEN,ISG20,ACAN,HAPLN3,MFGE8,ABHD2,RLBP1,FANCI,FANCI,POLG,POLG,POLG,MIR6766,MIR9-3HG,MIR9-3,RHCG,TICRR,KIF7,PLIN1,PEX11A,WDR93,MESP1,MESP2,ANPEP,AP3S2,C15orf38-AP3S2,AP3S2,C15orf38-AP3S2,MIR5094,AP3S2,C15orf38-AP3S2,MIR5009,C15orf38-AP3S2,ARPIN,ZNF710,MIR3174,ZNF710,ZNF710,ZNF710-AS1,IDH2,IDH2,IDH2-DT,SEMA4B,CIB1,CIB1,GDPGP1,CIB1,TTLL13P,CIB1,NGRN,NGRN,GABARAPL3,ZNF774,IQGAP1,CRTC3,CRTC3,CRTC3-AS1 | 0.0857615 | 281.304 | 778 | 479.916 |
| 2 | 3 | 5220234 | 8923174 | ARL8B,EDEM1,MIR4790,GRM7-AS3,GRM7,LMCD1-AS1,LMCD1,SSUH2,CAV3,OXTR,RAD18 | 0.0810951 | 245.008 | 125 | 70.9643 |
| 2 | 3 | 1.26E+08 | 130116906 | TXNRD3,NUP210P1,CHCHD6,PLXNA1,C3orf56,LINC02016,TPRA1,TPRA1,MIR6825,MCM2,PODXL2,ABTB1,MGLL,KBTBD12,SEC61A1,SEC61A1,RUVBL1,RUVBL1,RUVBL1,EEFSEC,EEFSEC,DNAJB8,GATA2,LINC01565,RPN1,RAB7A,LOC653712,ACAD9,ACAD9,KIAA1257,KIAA1257,KIAA1257,EFCC1,EFCC1,GP9,RAB43,ISY1-RAB43,ISY1-RAB43,ISY1,CNBP,COPG1,COPG1,MIR6826,HMCES,H1FX,RPL32P3,RPL32P3,SNORA7B,EFCAB12,MBD4,IFT122,RHO,H1FOO,PLXND1,TMCC1,TRH,ALG1L2,LINC02014,COL6A5 | 0.0727554 | 270.238 | 522 | 313.215 |
| 2 | 3 | 37408124 | 38257071 | C3orf35,ITGA9,ITGA9,ITGA9-AS1,CTDSPL,CTDSPL,MIR26A1,VILL,PLCD1,DLEC1,ACAA1,MYD88,OXSR1 | 0.070492 | 272.179 | 168 | 100.732 |
| 2 | 3 | 44903310 | 45744409 | MIR564,TMEM42,TMEM42,TGM4,ZDHHC3,EXOSC7,CLEC3B,CDCP1,TMEM158,LARS2,LARS2,LARS2-AS1,LIMD1,SACM1L | 0.0671113 | 233.315 | 117 | 69.9753 |
| 2 | 3 | 56716629 | 57231704 | FAM208A,ARHGEF3,ARHGEF3,ARHGEF3-AS1,ARHGEF3,SPATA12,IL17RD | 0.0669195 | 231.977 | 60 | 34.4211 |
| 2 | 1 | 94009615 | 94334727 | FNBP1L,BCAR3,BCAR3,LOC100129046,BCAR3,MIR760 | 0.0668993 | 227.98 | 28 | 16.8611 |

| | | | | | | | | |
|---|---|---|---|---|---|---|---|---|
| 2 | 1 | 7796891 | 35450977 | CAMTA1,VAMP3,PER3,UTS2,TNFRSF9,PARK7,ERRFI1,SLC45A1,RERE,ENO1,ENO1,MIR6728,CA6,SLC2A7,SLC2A5,GPR157,MIR34AHG,MIR34A,MIR34AHG,LINC01759,H6PD,SPSB1,SLC25A33,TMEM201,PIK3CD,PIK3CD-AS1,PIK3CD,CLSTN1,CTNNBIP1,LZIC,NMNAT1,MIR5697,NMNAT1,RBP7,UBE4B,KIF1B,PGD,CENPS,CENPS-CORT,CENPS-CORT,CORT,DFFA,PEX14,CASZ1,C1orf127,TARDBP,MASP2,SRM,EXOSC10,EXOSC10,LOC105376736,MTOR,MTOR,MTOR-AS1,MTOR,ANGPTL7,UBIAD1,DISP3,FBXO2,FBXO2,FBXO44,FBXO44,FBXO6,MAD2L2,DRAXIN,AGTRAP,C1orf167,C1orf167,LOC102724659,C1orf167,MTHFR,MTHFR,CLCN6,NPPA-AS1,NPPA,NPPB,KIAA2013,PLOD1,MFN2,MIIP,MIIP,MIR6729,TNFRSF8,MIR7846,TNFRSF1B,TNFRSF1B,TNFRSF1B,MIR4632,VPS13D,VPS13D,SNORA59B,SNORA59A,DHRS3,AADACL4,AADACL3,C1orf158,PRAMEF12,PRAMEF1,PRAMEF11,HNRNPCL4,HNRNPCL3,HNRNPCL1,PRAMEF2,PRAMEF18,HNRNPCL2,PRAMEF18,PRAMEF22,PRAMEF18,PRAMEF22,PRAMEF19,PRAMEF18,PRAMEF20,PRAMEF18,PRAMEF19,PRAMEF20,LRRC38,PDPN,PRDM2,KAZN,KAZN,TMEM51-AS1,TMEM51,C1orf195,TMEM51,FHAD1,FHAD1,LOC101927417,EFHD2,CTRC,CELA2A,CELA2B,CELA2B,CASP9,CASP9,DNAJC16,AGMAT,DDI2,DDI2,RSC1A1,RSC1A1,PLEKHM2,SLC25A34,SLC25A34-AS1,TMEM82,FBLIM1,UQCRHL,FLJ37453,SPEN,SPEN,MIR5096,SPEN,ZBTB17,SRARP,HSPB7,CLCNKA,CLCNKB,FAM131C,EPHA2,ARHGEF19,RSG1,FBXO42,SZRD1,SPATA21,NECAP2,CROCCP3,LOC440570,CROCCP2,MST1P2,FAM231AP,FAM231A,FAM231C,ESPNP,FAM231C,FAM231AP,FAM231A,MST1L,LOC102724562,MST1L,CR | 0.0654878 | 273.739 | 4955 | 2972.07 |
| 2 | 2 | 74415395 | 74617815 | MTHFD2,SLC4A5,DCTN1,DCTN1,DCTN1-AS1 | 0.0628895 | 259.327 | 73 | 44.665 |
| 2 | 5 | 1.5E+08 | 151166359 | ZNF300P1,GPX3,TNIP1,ANXA6,CCDC69,GM2A,SLC36A3,SLC36A2,SLC36A1,FAT2,SPARC,ATOX1,G3BP1 | 0.0626755 | 256.28 | 189 | 122.215 |
| 2 | 1 | 1.1E+08 | 111217432 | GNAT2,AMPD2,GSTM4,GSTM2,GSTM5,GSTM3,EPS8L3,CSF1,AHCYL1,STRIP1,ALX3,UBL4B,SLC6A17,KCNC4,RBM15,SLC16A4,LAMTOR5,LAMTOR5,LAMTOR5-AS1,PROK1,KCNA10,KCNA2,KCNA3 | 0.062475 | 292.831 | 228 | 139.937 |
| 2 | 2 | 29087851 | 31805970 | TRMT61B,WDR43,WDR43,SNORD92,WDR43,SNORD53,TOGARAM2,C2orf71,CLIP4,ALK,YPEL5,LBH,LCLAT1,CAPN13,GALNT14,CAPN14,EHD3,XDH,SRD5A2 | 0.0562476 | 268.008 | 290 | 176.838 |

| | | | | | | | | |
|---|---|---|---|---|---|---|---|---|
| 2 | 19 | 54006847 | 59118483 | ZNF331,LOC284379,DPRX,MIR512-2,MIR512-1,MIR1323,MIR498,MIR520E,MIR515-1,MIR515-2,MIR519E,MIR520F,MIR519C,MIR1283-1,MIR520A,MIR526B,MIR519B,MIR525,MIR523,MIR518F,MIR520B,MIR518B,MIR526A1,MIR520C,MIR518C,MIR524,MIR517A,MIR519D,MIR521-2,MIR520D,MIR517B,MIR520G,MIR516B2,MIR526A2,MIR518E,MIR518A1,MIR518D,MIR516B1,MIR518A2,MIR517C,MIR520H,MIR521-1,MIR522,MIR519A1,MIR527,MIR516A1,MIR1283-2,MIR516A2,MIR371A,MIR371B,MIR372,MIR373,NLRP12,MYADM,PRKCG,CACNG7,CACNG8,CACNG8,MIR935,CACNG6,VSTM1,TARM1,OSCAR,NDUFA3,TFPT,PRPF31,CNOT3,LENG1,TMC4,MBOAT7,TSEN34,RPS9,LILRB3,LILRB3,LILRA6,LILRB5,LILRB2,MIR4752,LILRA3,LILRA5,LILRA4,LAIR1,TTYH1,LENG8,LENG8,LENG9,LENG9,CDC42EP5,LAIR2,KIR3DX1,LILRA2,LILRA1,LILRB1,LILRB4,KIR3DL3,KIR2DL3,LOC101928804,KIR2DL1,KIR2DL1,KIR2DL4,KIR3DL1,KIR2DS4,KIR3DL2,FCAR,NCR1,NLRP7,NLRP2,GP6,RDH13,EPS8L1,PPP1R12C,TNNT1,TNNI3,DNAAF3,SYT5,PTPRH,TMEM86B,PPP6R1,MIR6804,PPP6R1,PPP6R1,MIR6802,PPP6R1,MIR6803,HSPBP1,BRSK1,TMEM150B,KMT5C,COX6B2,FAM71E2,IL11,TMEM190,TMEM238,RPL28,RPL28,MIR6805,UBE2S,SHISA7,ISOC2,ZNF628,NAT14,SSC5D,SBK2,SBK3,ZNF579,FIZ1,ZNF524,ZNF865,ZNF784,ZNF580,ZNF581,CCDC106,U2AF2,EPN1,NLRP9,RFPL4A,RFPL4AL1,NLRP11,NLRP4,NLRP13,NLRP8,NLRP5,ZNF787,ZNF444,GALP,ZSCAN5B,ZSCAN5C,ZSCAN5A,ZNF542P,ZNF582,ZNF583,ZNF667,ZNF667-AS1,ZNF471,ZFP28,ZNF470,ZNF71,SMIM17,ZNF835,ZIM2-AS1,ZIM2,ZIM2,PEG3,PEG3-AS1,ZIM2,PEG3,USP29,ZIM3,DUXA,ZNF264,AURKC,ZNF805,ZNF460,ZNF543,ZNF304,TRAPPC2B,ZNF547,ZNF | 0.0554345 | 289.341 | 1839 | 1106.27 |
| 2 | 5 | 1.71E+08 | 180374817 | FBXW11,STK10,EFCAB9,UBTD2,SH3PXD2B,NEURL1B,NEURL1B,MIR5003,DUSP1,ERGIC1,LOC100268168,RPL26L1,ATP6V0E1,ATP6V0E1,SNORA74B,CREBRF,BNIP1,NKX2-5,STC2,BOD1,CPEB4,C5orf47,NSG2,MSX2,MIR4634,DRD1,SFXN1,HRH2,CPLX2,THOC3,LOC100996385,FAM153B,SIMC1,KIAA1191,ARL10,ARL10,MIR1271,NOP16,NOP16,HIGD2A,HIGD2A,CLTB,FAF2,RNF44,CDHR2,GPRIN1,SNCB,SNCB,MIR4281,EIF4E1B,TSPAN17,UNC5A,HK3,UIMC1,ZNF346,FGFR4,NSD1,RAB24,PRELID1,PRELID1,MXD3,MXD3,LMAN2,RGS14,SLC34A1,PFN3,F12,GRK6,GRK6,PRR7-AS1,PRR7,DBN1,PDLIM7,DOK3,DDX41,FAM193B,TMED9,B4GALT7,LOC202181,FAM153A,LOC728554,PROP1,FAM153C,N4BP3,RMND5B,NHP2,GMCL2,GMCL1P1,HNRNPAB,HNRNPAB,PHYKPL,PHYKPL,COL23A1,CLK4,ZNF354A,ZNF354B,ZFP2,ZNF454,GRM6,ZNF879,ZNF354C,ADAMTS2,RUFY1,RUFY1,LOC101928445,HNRNPH1,C5orf60,CBY3,CANX,MAML1,LTC4S,MGAT4B,MIR1229,MGAT4B,MGAT4B,SQSTM1,SQSTM1,SQSTM1,MRNIP,MRNIP,LOC100996419,TBC1D9B,RNF130,RNF130,MIR340,RASGEF1C,MAPK9,GFPT2,CNOT6,SCGB3A1,FLT4,OR2Y1,MGAT1,ZFP62,BTNL8 | 0.0530181 | 274.051 | 1340 | 796.608 |

| | | | | | | | | |
|---|---|---|---|---|---|---|---|---|
| 2 | 2 | 74785964 | 75874362 | M1AP,SEMA4F,HK2,POLE4,TACR1,TACR1,MIR5000,EVA1A,MRPL19 | 0.0529903 | 252.958 | 85 | 48.8722 |
| 2 | 5 | 1.8E+08 | 180904760 | BTNL3,BTNL9,OR2V1,OR2V2,TRIM7,MIR4638,TRIM41,RACK1,RACK1,SNORD96A,RACK1,SNORD95,CTC-338M12.4,CTC-338M12.4,TRIM52,TRIM52 | 0.0525627 | 270.762 | 79 | 47.262 |
| 2 | 16 | 64981519 | 66793202 | CDH11,LINC00922,CDH5,BEAN1,TK2,CKLF,CKLF-CMTM1,CKLF-CMTM1,CMTM1,CMTM2,CMTM3,CMTM4,DYNC1LI2,TERB1 | 0.0475919 | 271.919 | 120 | 70.3953 |
| 2 | 1 | 1.81E+08 | 182811094 | XPR1,KIAA1614,KIAA1614-AS1,STX6,MR1,IER5,CACNA1E,ZNF648,GLUL,TEDDM1,RGSL1,RNASEL,RGS16,RGS8,NPL | 0.0465896 | 270.019 | 233 | 139.839 |
| 2 | 12 | 1.13E+08 | 118650185 | RPH3A,OAS1,OAS3,OAS2,DTX1,RASAL1,CFAP73,CFAP73,DDX54,CFAP73,DDX54,MIR7106,DDX54,RITA1,IQCD,TPCN1,TPCN1,MIR6762,SLC8B1,PLBD2,SDS,SDSL,LHX5,RBM19,TBX5,TBX3,MED13L,MED13L,MIR620,LINC00173,MAP1LC3B2,C12orf49,RNFT2,HRK,FBXW8,FBXW8,LOC100506551,TESC,FBXO21,NOS1,KSR2,RFC5,WSB2,VSIG10,PEBP1,TAOK3 | 0.0447057 | 253.185 | 558 | 332.318 |
| 2 | 20 | 17207989 | 20610166 | PCSK2,BFSP1,DSTN,RRBP1,BANF2,SNX5,SNX5,SNORD17,MGME1,OVOL2,PET117,PET117,KAT14,KAT14,ZNF133,LINC00851,DZANK1,POLR3F,POLR3F,MIR3192,RBBP9,SEC23B,SMIM26,DTD1,C20orf78,C20orf78,SCP2D1,SLC24A3,SLC24A3,LOC100130264,RIN2,NAA20,CRNKL1,CRNKL1,CFAP61,CFAP61,INSM1,RALGAPA2 | 0.0434538 | 289.28 | 394 | 230.475 |
| 2 | X | 49157757 | 58505576 | GAGE10,GAGE1,PAGE1,PAGE4,USP27X-AS1,USP27X,CLCN5,CLCN5,MIR532,CLCN5,MIR188,CLCN5,MIR500A,CLCN5,MIR362,CLCN5,MIR501,CLCN5,MIR500B,CLCN5,MIR660,CLCN5,MIR502,AKAP4,CCNB3,DGKK,SHROOM4,BMP15,NUDT10,CXorf67,NUDT11,GSPT2,MAGED1,MAGED4B,MAGED4,MAGED4B,MAGED4,SNORA11D,SNORA11E,XAGE2,SSX8,SSX7,SSX2B,SSX2,SSX2,SSX2B,SPANXN5,XAGE5,XAGE3,FAM156B,FAM156A,GPR173,TSPYL2,KDM5C,KDM5C,MIR6895,KDM5C,MIR6894,IQSEC2,SMC1A,SMC1A,MIR6857,RIBC1,HSD17B10,HUWE1,HUWE1,MIR98,HUWE1,MIRLET7F2,PHF8,FAM120C,WNK3,TSR2,TSR2,FGD1,FGD1,GNL3L,ITIH6,MAGED2,MAGED2,SNORA11,TRO,PFKFB1,APEX2,ALAS2,PAGE2B,PAGE2,FAM104B,MTRNR2L10,PAGE5,PAGE3,MIR4536-1,MIR4536-2,MAGEH1,USP51,FOXR2,RRAGB,KLF8,UBQLN2,SPIN3,SPIN2B,SPIN2A,FAAH2,ZXDB,ZXDA | 0.0417913 | 279.001 | 923 | 551.517 |
| 2 | 15 | 40951555 | 42480131 | KNL1,RAD51,RMDN3,GCHFR,DNAJC17,DNAJC17,C15orf62,DNAJC17,ZFYVE19,ZFYVE19,PPP1R14D,SPINT1-AS1,SPINT1,SPINT1,RHOV,VPS18,DLL4,CHAC1,INO80,EXD1,CHP1,OIP5,NUSAP1,NDUFAF1,RTF1,ITPKA,LTK,RPAP1,TYRO3,MGA,MGA,MIR626,MAPKBP1,JMJD7,JMJD7-PLA2G4B,JMJD7-PLA2G4B,PLA2G4B,SPTBN5,SPTBN5,MIR4310,SPTBN5,LOC105370792,LOC105370792,EHD4,PLA2G4E-AS1,PLA2G4E,PLA2G4E,PLA2G4D,PLA2G4F,VPS39 | 0.0410104 | 262.28 | 598 | 375.933 |

| | | | | | | | | |
|---|---|---|---|---|---|---|---|---|
| 2 | 19 | 33324097 | 34699417 | SLC7A9,CEP89,FAAP24,RHPN2,GPATCH1,WDR88,LRP3,LRP3,SLC7A10,SLC7A10,CEBPA,CEBPG,PEPD,CHST8,KCTD15,LSM14A | 0.0385787 | 280.056 | 189 | 109.218 |
| 2 | 10 | 7850245 | 18874371 | TAF3,GATA3-AS1,GATA3,LOC101928272,CELF2,CELF2,CELF2-AS1,USP6NL,ECHDC3,PROSER2,PROSER2-AS1,UPF2,DHTKD1,SEC61A2,SEC61A2,NUDT5,NUDT5,CDC123,CAMK1D,CAMK1D,MIR4480,CAMK1D,MIR4481,CAMK1D,MIR548Q,CCDC3,OPTN,MCM10,UCMA,PHYH,SEPHS1,BEND7,PRPF18,FRMD4A,MIR4293,FAM107B,CDNF,HSPA14,SUV39H2,SUV39H2,DCLRE1C,DCLRE1C,MEIG1,DCLRE1CP1,ACBD7-DCLRE1CP1,ACBD7-DCLRE1CP1,OLAH,ACBD7-DCLRE1CP1,ACBD7,C10orf111,RPP38,NMT2,NMT2,PPIAP30,FAM171A1,ITGA8,MINDY3,PTER,C1QL3,RSU1,CUBN,TRDMT1,VIM-AS1,VIM,VIM,ST8SIA6,ST8SIA6,ST8SIA6-AS1,HACD1,STAM,TMEM236,MRC1,MRC1,MIR511,MRC1,TMEM236,SLC39A12,SLC39A12,SLC39A12-AS1,CACNB2,NSUN6 | 0.0381034 | 275.242 | 895 | 508.291 |
| 2 | 17 | 20305919 | 22220573 | LGALS9B,CDRT15L2,LOC100287072,CCDC144NL,CCDC144NL,CCDC144NL-AS1,USP22,DHRS7B,TMEM11,NATD1,MAP2K3,KCNJ12,KCNJ18,C17orf51,UBBP4,FAM27E5 | 0.0379049 | 299.627 | 123 | 63.4612 |
| 2 | 1 | 2.23E+08 | 225139863 | DISP1,TLR5,SUSD4,CCDC185,CAPN8,CAPN2,TP53BP2,LOC100287497,FBXO28,DEGS1,LOC101927164,NVL,NVL,NVL,MIR320B2,CNIH4,WDR26,WDR26,MIR4742,CNIH3,DNAH14 | 0.0369876 | 265.259 | 234 | 138.949 |
| 2 | 14 | 23043984 | 23978687 | DAD1,ABHD4,OR6J1,OXA1L,SLC7A7,MRPL52,MMP14,LRP10,REM2,RBM23,PRMT5-AS1,PRMT5,PRMT5,LOC101926933,HAUS4,HAUS4,HAUS4,MIR4707,AJUBA,C14orf93,PSMB5,PSMB11,CDH24,ACIN1,C14orf119,LMLN2,CEBPE,SLC7A8,RNF212B,HOMEZ,PPP1R3E,BCL2L2,BCL2L2-PABPN1,BCL2L2-PABPN1,BCL2L2-PABPN1,PABPN1,SLC22A17,EFS,IL25,CMTM5,MYH6,MYH6,MIR208A,MYH7,MYH7,MHRT,MYH7,MIR208B,NGDN | 0.0355894 | 270.453 | 395 | 242.46 |
| 2 | 19 | 50492918 | 52327814 | VRK3,ZNF473,LOC400710,IZUMO2,MYH14,KCNC3,NAPSB,NAPSA,NR1H2,POLD1,SPIB,MYBPC2,FAM71E1,FAM71E1,EMC10,EMC10,JOSD2,ASPDH,LRRC4B,SYT3,C19orf81,SHANK1,CLEC11A,GPR32,ACP4,C19orf48,C19orf48,SNORD88B,C19orf48,SNORD88A,C19orf48,SNORD88C,LINC01869,KLK1,KLK15,KLK15,LOC105372441,KLK3,KLK2,KLKP1,KLK4,KLK5,KLK6,KLK7,KLK8,KLK9,KLK10,KLK11,KLK12,KLK13,KLK14,CTU1,SIGLEC9,SIGLEC7,SIGLEC7,LOC101928517,LOC101928517,SIGLEC17P,CD33,SIGLECL1,IGLON5,VSIG10L,ETFB,CLDND2,NKG7,LIM2,C19orf84,SIGLEC10,SIGLEC10,LOC100129083,SIGLEC8,CEACAM18,SIGLEC12,SIGLEC6,ZNF175,LINC01530,SIGLEC5,SIGLEC14,SPACA6P-AS,SPACA6,MIR99B,MIRLET7E,SPACA6P-AS,SPACA6,MIR125A,SPACA6,HAS1,FPR1,FPR2,FPR3 | 0.0354576 | 265.184 | 633 | 379.591 |

| | | | | | | | | |
|---|---|---|---|---|---|---|---|---|
| 2 | 5 | 1.48E+08 | 148875949 | ADRB2,SH3TC2,ABLIM3,AFAP1L1,GRPEL2,GRPEL2,GRPEL2-AS1,PCYOX1L,IL17B,CARMN,MIR143,CARMN,MIR145 | 0.035055 | 232.776 | 115 | 69.9184 |
| 2 | 14 | 19057400 | 21624149 | DUXAP10,BMS1P22,OR4Q3,OR4M1,OR4N2,OR4K2,OR4K5,OR4K1,OR4K15,OR4Q2,OR4K14,OR4K13,OR4L1,OR4K17,OR4N5,OR11G2,OR11H6,OR11H7,OR11H4,TTC5,CCNB1IP1,CCNB1IP1,SNORD126,PARP2,TEP1,KLHL33,OSGEP,APEX1,PIP4P1,PNP,RNASE10,RNASE9,RNASE11,LOC254028,RNASE12,LOC254028,OR6S1,ANG,RNASE4,RNASE4,EDDM3A,EDDM3B,RNASE6,RNASE1,RNASE3,ECRP,RNASE2,METTL17,METTL17,LOC101929718,SLC39A2,NDRG2,NDRG2,MIR6717,NDRG2,TPPP2,NDRG2,RNASE13,NDRG2,RNASE7,NDRG2,RNASE8,NDRG2,ARHGEF40,ARHGEF40,ARHGEF40,ZNF219,ZNF219,ZNF219,TMEM253,OR5AU1 | 0.033538 | 284.351 | 398 | 236.875 |
| 2 | 19 | 28263182 | 32845886 | UQCRFS1,VSTM2B,POP4,PLEKHF1,C19orf12,CCNE1,URI1,ZNF536,TSHZ3,THEG5,ZNF507 | 0.0322331 | 255.48 | 139 | 84.6884 |
| 2 | 14 | 97348071 | 101377664 | LINC00618,LINC02291,LINC02312,LINC01550,C14orf177,BCL11B,SETD3,CCNK,CCDC85C,HHIPL1,CYP46A1,EML1,EVL,EVL,MIR151B,MIR342,DEGS2,YY1,YY1,MIR6764,SLC25A29,MIR345,SLC25A47,WARS,WDR25,BEGAIN,LINC00523,DLK1,MIR2392,MEG3,MEG3,MIR770,MIR493,MIR337,MIR665,RTL1,RTL1,MIR431,RTL1,MIR433,RTL1,MIR127,RTL1,MIR432,RTL1,MIR136,MEG8,MEG8,MIR370 | 0.0320269 | 248.816 | 299 | 177.421 |
| 2 | 14 | 94173132 | 94697678 | UNC79,PRIMA1,FAM181A-AS1,FAM181A,FAM181A,ASB2,ASB2,MIR4506,CCDC197,OTUB2,DDX24,IFI27L1,IFI27,IFI27L2,PPP4R4 | 0.0305941 | 250.395 | 90 | 53.1577 |
| 2 | 20 | 1234923 | 5528506 | RAD21L1,SNPH,SDCBP2,FKBP1A-SDCBP2,FKBP1A-SDCBP2,SDCBP2-AS1,FKBP1A-SDCBP2,SDCBP2-AS1,FKBP1A,FKBP1A-SDCBP2,FKBP1A,MIR6869,NSFL1C,SIRPB2,SIRPD,SIRPB1,SIRPG,SIRPG,SIRPG-AS1,SIRPA,PDYN-AS1,PDYN,STK35,TGM3,TGM6,SNRPB,SNRPB,SNORD119,ZNF343,TMC2,NOP56,NOP56,MIR1292,NOP56,SNORD110,NOP56,SNORA51,NOP56,SNORD86,NOP56,SNORD56,NOP56,SNORD57,NOP56,IDH3B,IDH3B,EBF4,CPXM1,C20orf141,TMEM239,PCED1A,PCED1A,VPS16,VPS16,VPS16,PTPRA,PTPRA,GNRH2,MRPS26,OXT,AVP,UBOX5-AS1,UBOX5,UBOX5-AS1,UBOX5,FASTKD5,LZTS3,DDRGK1,ITPA,SLC4A11,C20orf194,ATRN,GFRA4,ADAM33,SIGLEC1,HSPA12B,C20orf27,SPEF1,CENPB,CDC25B,AP5S1,MAVS,PANK2,PANK2,MIR103A2,MIR103B2,RNF24,SMOX,ADRA1D,PRNP,PRND,PRNT,RASSF2,SLC23A2,TMEM230,PCNA,PCNA,PCNA-AS1,CDS2,PROKR2,LOC643406,LINC00654,GPCPD1 | 0.0287485 | 272.057 | 728 | 442.305 |

| | | | | | | | | |
|---|---|---|---|---|---|---|---|---|
| 2 | 12 | 48105406 | 50821753 | ENDOU,RAPGEF3,SLC48A1,HDAC7,VDR,TMEM106C,COL2A1,SENP1,SENP1,PFKM,PFKM,ASB8,CCDC184,OR10AD1,H1FNT,ZNF641,ANP32D,C12orf54,OR8S1,LALBA,KANSL2,KANSL2,SNORA2C,MIR1291,KANSL2,SNORA2A,KANSL2,SNORA2B,CCNT1,TEX49,ADCY6,ADCY6,MIR4701,CACNB3,DDX23,RND1,CCDC65,FKBP11,ARF3,WNT10B,WNT1,DDN,DDN,DDN-AS1,DDN-AS1,PRKAG1,KMT2D,RHEBL1,DHH,LMBR1L,TUBA1B,TUBA1A,TUBA1C,LOC101927267,PRPH,LOC101927267,TROAP,TROAP,C1QL4,DNAJC22,SPATS2,KCNH3,MCRS1,FAM186B,PRPF40B,PRPF40B,FMNL3,FMNL3,TMBIM6,NCKAP5L,BCDIN3D-AS1,BCDIN3D,BCDIN3D,FAIM2,LINC02396,AQP2,AQP2,LOC101927318,LOC101927318,AQP5,AQP5,AQP6,RACGAP1,ASIC1,SMARCD1,GPD1,COX14,CERS5,LIMA1,FAM186A,LARP4 | 0.0287316 | 258.297 | 933 | 580.757 |
| 2 | 13 | 1.03E+08 | 109707485 | BIVM,BIVM-ERCC5,BIVM-ERCC5,ERCC5,SLC10A2,DAOA-AS1,DAOA,EFNB2,ARGLU1,FAM155A,FAM155A,MIR1267,LIG4,ABHD13,TNFSF13B,MYO16 | 0.0269586 | 243.354 | 168 | 97.8184 |
| 2 | 11 | 89955781 | 92881256 | CHORDC1,DISC1FP1,MIR4490,DISC1FP1,MIR1261,FAT3,MTNR1B | 0.0268435 | 280.119 | 99 | 62.1288 |
| 2 | 8 | 18661993 | 21862625 | PSD3,SH2D4A,CSGALNACT1,INTS10,LPL,SLC18A1,ATP6V1B2,LZTS1,GFRA2,DOK2,XPO7 | 0.0244927 | 246.2 | 197 | 118.593 |
| 2 | 10 | 1.02E+08 | 102684769 | BLOC1S2,PKD2L1,SCD,WNT8B,SEC31B,NDUFB8,HIF1AN,PAX2,SLF2 | 0.0201651 | 272.08 | 118 | 70.6274 |
| 2 | 6 | 4077584 | 7326511 | FAM217A,C6orf201,C6orf201,ECI2,ECI2,CDYL,RPP40,RPP40,LYRM4-AS1,LYRM4-AS1,LYRM4,PPP1R3G,LYRM4-AS1,LYRM4,LYRM4-AS1,LYRM4,MIR3691,LYRM4,FARS2,NRN1,F13A1,F13A1,MIR7853,MIR5683,LY86-AS1,LY86,LY86,RREB1,SSR1 | 0.0183852 | 235.937 | 177 | 100.969 |
| 2 | 1 | 2.04E+08 | 203769358 | ATP2B4,ATP2B4,SNORA77,LAX1,ZC3H11A,ZBED6 | 0.0172509 | 274.364 | 46 | 29.9723 |
| 2 | 7 | 30102925 | 31569448 | PLEKHA8,MTURN,LOC105375218,ZNRF2,ZNRF2,MIR550A1,MIR550B1,ZNRF2,NOD1,GGCT,GARS,CRHR2,INMT,INMT-MINDY4,INMT-MINDY4,INMT-MINDY4,MINDY4,AQP1,GHRHR,ADCYAP1R1,NEUROD6,CCDC129 | 0.0169778 | 258.634 | 172 | 101.742 |
| 2 | 14 | 92628696 | 93676288 | SLC24A4,RIN3,LGMN,GOLGA5,CHGA,ITPK1,MOAP1,TMEM251,GON7,UBR7 | 0.0169342 | 247.137 | 135 | 77.933 |

| | | | | | | | | |
|---|---|---|---|---|---|---|---|---|
| 2 | 20 | 35697073 | 44445484 | RBL1,MROH8,MROH8,RPN2,RPN2,GHRH,MANBAL,SRC,BLCAP,BLCAP,NNAT,CTNNBL1,VSTM2L,TTI1,RPRD1B,TGM2,KIAA1755,KIAA1755,LOC149684,BPI,LBP,SNHG17,SNORA71B,SNHG17,SNORA71A,SNHG17,SNORA71C,SNHG17,SNORA71D,SNHG11,SNORA71E,SNHG11,SNORA60,RALGAPB,RALGAPB,MIR548O2,ADIG,ARHGAP40,SLC32A1,ACTR5,PPP1R16B,FAM83D,DHX35,MAFB,LOC100128988,TOP1,TOP1,PLCG1-AS1,PLCG1-AS1,PLCG1,PLCG1,ZHX3,LPIN3,EMILIN3,CHD6,PTPRT,SRSF6,L3MBTL1,SGK2,IFT52,MYBL2,GTSF1L,TOX2,JPH2,OSER1,GDAP1L1,FITM2,R3HDML,HNF4A,HNF4A,HNF4A-AS1,HNF4A,MIR3646,LINC01620,TTPAL,SERINC3,PKIG,ADA,KCNK15-AS1,WISP2,KCNK15-AS1,KCNK15,KCNK15,RIMS4,YWHAB,PABPC1L,PABPC1L,TOMM34,TOMM34,STK4,KCNS1,WFDC5,WFDC12,PI3,SEMG1,SEMG2,SLPI,MATN4,MATN4,RBPJL,RBPJL,SDC4,SYS1,SYS1-DBNDD2,SYS1,SYS1-DBNDD2,TP53TG5,SYS1-DBNDD2,TP53TG5,SYS1-DBNDD2,DBNDD2,PIGT,PIGT,MIR6812,WFDC2,SPINT3,WFDC6,WFDC6,EPPIN-WFDC6,EPPIN-WFDC6,EPPIN,WFDC8,WFDC9,WFDC9,WFDC10A,WFDC11,WFDC10B,WFDC10B,WFDC13,WFDC10B,WFDC13,MIR3617,WFDC13,SPINT4,WFDC3,WFDC3,DNTTIP1,DNTTIP1,UBE2C | 0.0163954 | 261.643 | 1043 | 635.467 |
| 2 | 11 | 33363062 | 36615728 | HIPK3,KIAA1549L,C11orf91,CD59,FBXO3,LMO2,CAPRIN1,NAT10,ABTB2,CAT,ELF5,EHF,APIP,APIP,PDHX,PDHX,PDHX,MIR1343,CD44,SLC1A2,PAMR1,FJX1,TRIM44,LDLRAD3,MIR3973,LDLRAD3,COMMD9,PRR5L,TRAF6,RAG1,RAG2 | 0.0158473 | 280.27 | 344 | 210.894 |
| 2 | 15 | 43910781 | 44004517 | STRC,CATSPER2,PPIP5K1P1-CATSPER2,PPIP5K1P1-CATSPER2,CKMT1A,STRCP1 | 0.012125 | 290.819 | 88 | 48.3061 |
| 2 | 3 | 1.38E+08 | 141230423 | CEP70,FAIM,PIK3CB,FOXL2,FOXL2NB,PRR23A,PRR23B,PRR23C,MRPS22,COPB2,LOC100507291,RBP2,LOC100507291,RBP1,LOC100507291,NMNAT3,NMNAT3,CLSTN2,TRIM42,SLC25A36,SPSB4,PXYLP1,ZBTB38,RASA2 | 0.0119783 | 266.114 | 212 | 122.581 |
| 2 | 20 | 47732238 | 55905066 | STAU1,DDX27,ZNFX1,ZFAS1,SNORD12C,ZFAS1,SNORD12B,ZFAS1,SNORD12,KCNB1,PTGIS,B4GALT5,SLC9A8,SPATA2,RNF114,SNAI1,UBE2V1,TMEM189-UBE2V1,TMEM189-UBE2V1,TMEM189,CEBPB-AS1,CEBPB,SMIM25,PTPN1,MIR645,RIPOR3,RIPOR3,MIR1302-5,PARD6B,BCAS4,ADNP,ADNP-AS1,DPM1,DPM1,MOCS3,KCNG1,NFATC2,NFATC2,MIR3194,ATP9A,SALL4,ZFP64,TSHZ2,LOC101927770,ZNF217,ZNF217,BCAS1,BCAS1,MIR4756,CYP24A1,PFDN4,DOK5,CBLN4,MC3R,FAM210B,AURKA,CSTF1,CASS4,RTFDC1,RTFDC1,GCNT7,GCNT7,FAM209A,FAM209B,TFAP2C,BMP7,MIR4325,SPO11 | 0.0117094 | 274.522 | 602 | 362.214 |

| | | | | | | | | |
|---|---|---|---|---|---|---|---|---|
| 2 | 10 | 99190335 | 101916067 | PGAM1,EXOSC1,ZDHHC16,MMS19,UBTD1,ANKRD2,HOGA1,HOGA1,C10orf62,MORN4,PI4K2A,AVPI1,MARVELD1,ZFYVE27,SFRP5,GOLGA7B,GOLGA7B,CRTAC1,CRTAC1,R3HCC1L,LOXL4,PYROXD2,PYROXD2,MIR1287,HPS1,HPS1,MIR4685,HPSE2,CNNM1,GOT1,NKX2-3,SLC25A28,ENTPD7,ENTPD7,COX15,COX15,COX15,CUTC,CUTC,ABCC2,DNMBP,DNMBP,DNMBP-AS1,CPN1,ERLIN1 | 0.0112073 | 266.455 | 405 | 247.695 |
| 2 | 12 | 1.19E+08 | 122962562 | SUDS3,SRRM4,HSPB8,LINC00934,CCDC60,TMEM233,PRKAB1,CIT,CIT,MIR1178,BICDL1,RAB35,GCN1,GCN1,MIR4498,RPLP0,PXN-AS1,PXN,PXN,SIRT4,PLA2G1B,MSI1,COX6A1,TRIAP1,TRIAP1,GATC,GATC,GATC,SRSF9,SRSF9,DYNLL1,COQ5,RNF10,POP5,CABP1,MLEC,UNC119B,UNC119B,MIR4700,ACADS,SPPL3,HNF1A,HNF1A,C12orf43,C12orf43,OASL,P2RX7,P2RX4,CAMKK2,ANAPC5,RNF34,KDM2B,KDM2B,MIR7107,ORAI1,MORN3,TMEM120B,TMEM120B,RHOF,RHOF,LINC01089,SETD1B,HPD,PSMD9,WDR66,BCL7A,MLXIP,LRRC43,IL31,LRRC43,B3GNT4,B3GNT4,DIABLO,DIABLO,VPS33A,CLIP1,ZCCHC8 | 0.0108974 | 270.383 | 755 | 459.038 |
| 2 | 9 | 10500 | 2719107 | CBWD1,C9orf66,C9orf66,DOCK8,DOCK8,KANK1,DMRT1,DMRT3,DMRT2,SMARCA2,VLDLR-AS1,VLDLR,VLDLR,KCNV2 | 0.0106029 | 267.279 | 203 | 123.1 |
| 2 | 2 | 2.38E+08 | 238722435 | COL6A3,MLPH,MLPH,MIR6811,PRLH,RAB17,LRRFIP1,RBM44 | 0.0105065 | 255.372 | 143 | 87.9739 |
| 2 | 17 | 48801896 | 49062439 | LUC7L3,LINC00483,WFIKKN2,TOB1,SPAG9 | 0.00775103 | 259.214 | 44 | 27.2862 |
| 2 | 16 | 69381678 | 75794782 | TMED6,TERF2,CYB5B,MIR1538,NFAT5,NQO1,NOB1,WWP2,WWP2,MIR140,CLEC18A,PDXDC2P-NPIPB14P,PDXDC2P-NPIPB14P,MIR1972-1,MIR1972-2,PDPR,PDPR,LOC400541,CLEC18C,EXOSC6,AARS,DDX19B,DDX19B,LOC100506083,DDX19A,ST3GAL2,FUK,COG4,SF3B3,SF3B3,SNORD111B,SF3B3,SNORD111,IL34,MTSS1L,VAC14,VAC14,VAC14-AS1,HYDIN,CMTR2,CALB2,ZNF23,ZNF19,CHST4,TAT-AS1,TAT,TAT,MARVELD3,PHLPP2,PHLPP2,SNORA70D,AP1G1,AP1G1,SNORD71,ATXN1L,ZNF821,IST1,IST1,PKD1L3,PKD1L3,DHODH,HP,HPR,TXNL4B,DHX38,PMFBP1,ZFHX3,HCCAT5,C16orf47,PSMD7,LOC283922,NPIPB15,CLEC18B,GLG1,RFWD3,MLKL,FA2H,WDR59,ZNRF1,LDHD,ZFP1,CTRB2,CTRB1,BCAR1,CFDP1,TMEM170A,CHST6,CHST5,TMEM231,GABARAPL2,ADAT1,KARS,TERF2IP,DUXB | 0.00747457 | 274.616 | 1178 | 701.304 |
| 2 | 1 | 1.83E+08 | 184021114 | SHCBP1L,LAMC1,LAMC1,LAMC1-AS1,LAMC2,NMNAT2,SMG7,NCF2,ARPC5,RGL1,APOBEC4,RGL1,COLGALT2,TSEN15 | 0.00523311 | 284.242 | 180 | 110.053 |
| 2 | 2 | 27917984 | 29001423 | LINC01460,MRPL33,RBKS,RBKS,BABAM2,BRE-AS1,BABAM2,BABAM2,MIR4263,BABAM2,LOC100505716,FLJ31356,FOSL2,FOSL2,PLB1,PPP1CB | 0.00439791 | 269.122 | 131 | 77.1676 |
| 2 | 15 | 45335484 | 45723338 | SORD,DUOX2,DUOXA2,DUOXA2,DUOXA1,DUOXA1,DUOX1,SHF,LOC101928414,SLC28A2,GATM,SPATA5L1,C15orf48 | 0.00399899 | 285.643 | 155 | 93.5414 |
| 2 | 3 | 1.7E+08 | 169977258 | LRRC34,LRRIQ4,LRRC31,SAMD7,SEC62,GPR160,PHC3,PRKCI | 0.00101387 | 324.701 | 75 | 45.8009 |

| | | | | | | | | |
|---|---|---|---|---|---|---|---|---|
| 2 | 18 | 2796391 | 9104348 | SMCHD1,EMILIN2,LPIN2,LPIN2,LOC727896,MYOM1,MYL12A,MYL12A,LOC104968399,MYL12B,TGIF1,DLGAP1,DLGAP1,DLGAP1-AS1,DLGAP1,DLGAP1-AS2,DLGAP1,DLGAP1-AS3,AKAIN1,LINC00667,ZBTB14,EPB41L3,MIR3976,TMEM200C,L3MBTL4,L3MBTL4,MIR4317,LINC01387,LOC101927168,ARHGAP28,LINC00668,LAMA1,LAMA1,LOC101927188,LRRC30,PTPRM,RAB12,GACAT2,MTCL1,NDUFV2 | 0.00075721 | 276.674 | 472 | 273.537 |
| 2 | 1 | 1.16E+08 | 117911145 | SYCP1,TSHB,TSPAN2,NGF,VANGL1,CASQ2,NHLH2,LOC101928977,SLC22A15,SLC22A15,MAB21L3,ATP1A1,ATP1A1,ATP1A1-AS1,ATP1A1-AS1,CD58,IGSF3,MIR320B1,C1orf137,CD2,PTGFRN,CD101,CD101,LOC101929099,TTF2,TTF2,MIR942,TRIM45,VTCN1,MAN1A2 | -0.00033381 | 258.454 | 243 | 145.363 |
| 2 | 17 | 54587791 | 56634455 | NOG,C17orf67,DGKE,TRIM25,TRIM25,MIR3614,COIL,SCPEP1,RNF126P1,AKAP1,MSI2,CCDC182,MRPS23,CUEDC1,VEZF1,SRSF1,DYNLL2,OR4D1,MSX2P1,OR4D2,EPX,MKS1,LPO,MPO,TSPOAP1,TSPOAP1,TSPOAP1-AS1,TSPOAP1-AS1,MIR142,TSPOAP1-AS1,TSPOAP1-AS1,MIR4736,TSPOAP1-AS1,SUPT4H1,RNF43,HSF5,MTMR4,SEPT4-AS1,SEPT4,SEPT4-AS1,C17orf47,TEX14 | -0.0016332 | 266.858 | 350 | 214.822 |
| 2 | 14 | 1.01E+08 | 107283157 | MEG8,SNORD114-30,MEG8,SNORD114-31,MIR379,MIR411,MIR299,MIR380,MIR1197,MIR323A,MIR758,MIR494,MIR1193,MIR543,MIR495,MIR376C,MIR376A2,MIR654,MIR376B,MIR300,MIR1185-1,MIR1185-2,MIR381HG,MIR381,MIR381HG,MIR487B,MIR381HG,MIR539,MIR381HG,MIR889,MIR381HG,MIR544A,MIR381HG,MIR655,MIR487A,MIR382,MIR134,MIR668,MIR485,MIR323B,MIR154,MIR496,MIR377,MIR541,MIR409,MIR412,MIR369,MIR410,MIR656,DIO3OS,MIR1247,DIO3,LINC00239,PPP2R5C,DYNC1H1,HSP90AA1,HSP90AA1,WDR20,WDR20,MOK,ZNF839,CINP,TECPR2,ANKRD9,MIR4309,RCOR1,TRAF3,AMN,CDC42BPB,EXOC3L4,LINC00677,TNFAIP2,EIF5,EIF5,SNORA28,MARK3,CKB,TRMT61A,BAG5,APOPT1,KLC1,KLC1,XRCC3,XRCC3,ZFYVE21,PPP1R13B,LINC00637,ATP5MPL,TDRD9,TDRD9,RD3L,ASPG,MIR203A,MIR203B,KIF26A,C14orf144,C14orf180,TMEM179,MIR4710,INF2,ADSSL1,SIVA1,AKT1,ZBTB42,CEP170B,PLD4,AHNAK2,CLBA1,CDCA4,GPR132,JAG2,JAG2,MIR6765,NUDT14,BRF1,BRF1,BTBD6,BRF1,PACS2,PACS2,TEX22,MTA1,CRIP2,CRIP1,TEDC1,TMEM121,MIR4537,FAM30A,ADAM6,LINC00221,MIR5195 | -0.00211514 | 277.483 | 1216 | 709.038 |
| 2 | 20 | 45002026 | 47675165 | ELMO2,ZNF334,OCSTAMP,SLC13A3,TP53RK,SLC2A10,EYA2,EYA2,MIR3616,ZMYND8,ZMYND8,LOC100131496,NCOA3,SULF2,PREX1,ARFGEF2,CSE1L | -0.00459923 | 252.53 | 285 | 173.131 |
| 2 | 21 | 42080360 | 44279963 | DSCAM,MIR3197,BACE2,BACE2,PLAC4,FAM3B,MX2,MX1,TMPRSS2,LINC00479,RIPK4,RIPK4,MIR6814,PRDM15,C2CD2,ZBTB21,ZNF295-AS1,UMODL1,UMODL1,UMODL1-AS1,ABCG1,TFF3,TFF2,TFF1,TMPRSS3,UBASH3A,RSPH1,SLC37A1,PDE9A,WDR4 | -0.00491868 | 265.945 | 340 | 204.588 |

| | | | | | | | | |
|---|---|---|---|---|---|---|---|---|
| 2 | 11 | 94229984 | 94905859 | ANKRD49,C11orf97,FUT4,FUT4,LOC105369438,LOC105369438,PIWIL4,LOC105369438,AMOTL1,CWC15,KDM4D,KDM4E,SRSF8,ENDOD1 | -0.00695838 | 291.675 | 101 | 59.9309 |
| 2 | X | 1.29E+08 | 129338387 | SMARCA1,OCRL,APLN,XPNPEP2,SASH3,ZDHHC9,UTP14A,BCORL1,ELF4,AIFM1,RAB33A | -0.00727677 | 274.615 | 169 | 103.356 |
| 2 | 10 | 32344735 | 32740889 | KIF5B,EPC1,CCDC7 | -0.00763334 | 249.101 | 24 | 14.4808 |
| 2 | 10 | 1.06E+08 | 105882050 | SLK,COL17A1,COL17A1,MIR936,SFR1 | -0.00815968 | 270.108 | 64 | 39.0922 |
| 2 | 16 | 5122909 | 8993698 | ALG1,RBFOX1,TMEM114,METTL22,ABAT,TMEM186,TMEM186,PMM2,PMM2,LOC100130283,CARHSP1,CARHSP1,USP7 | -0.00875212 | 228.654 | 125 | 73.001 |
| 2 | 1 | 2.29E+08 | 234510217 | RAB4A,RAB4A,SPHAR,CCSAP,ACTA1,NUP133,ABCB10,TAF5L,URB2,GALNT2,PGBD5,COG2,AGT,CAPN9,C1orf198,TTC13,ARV1,FAM89A,MIR1182,FAM89A,TRIM67,C1orf131,GNPAT,EXOC8,SPRTN,EGLN1,TSNAX,TSNAX-DISC1,TSNAX-DISC1,TSNAX-DISC1,DISC1,TSNAX-DISC1,DISC1,DISC2,SIPA1L2,MAP10,NTPCR,PCNX2,MAP3K21,KCNK1,KCNK1,MIR4427,SLC35F3,SLC35F3,MIR4671,LOC101927765,COA6,COA6 | -0.00925764 | 281.041 | 533 | 316.091 |
| 2 | 10 | 76990533 | 89265281 | VDAC2,COMTD1,ZNF503,ZNF503,ZNF503-AS2,ZNF503-AS2,LRMDA,LRMDA,MIR606,KCNMA1,KCNMA1,KCNMA1-AS1,DLG5,DLG5-AS1,POLR3A,RPS24,ZMIZ1-AS1,ZMIZ1,PPIF,ZCCHC24,LOC729815,EIF5AL1,SFTPA2,SFTPA1,BMS1P21,MBL1P,SFTPD,TMEM254-AS1,TMEM254,TMEM254,PLAC9,ANXA11,LOC100130698,MAT1A,DYDC1,DYDC1,DYDC2,DYDC2,FAM213A,TSPAN14,SH2D4B,NRG3,NRG3,NRG3-AS1,GHITM,C10orf99,CDHR1,LRIT2,LRIT1,RGR,CCSER2,GRID1-AS1,GRID1,GRID1,GRID1,MIR346,WAPL,OPN4,LDB3,BMPR1A,MMRN2,MMRN2,SNCG,SNCG,ADIRF,ADIRF,AGAP11,AGAP11,GLUD1,FAM35A,MIR4678,MINPP1 | -0.0106263 | 259.361 | 715 | 415.061 |
| 2 | 15 | 1.01E+08 | 102520892 | LINS1,ASB7,ALDH1A3,ALDH1A3,LOC101927751,LRRK1,CHSY1,SELENOS,SNRPA1,PCSK6,PCSK6,LOC100507472,TM2D3,TARSL2,LOC100128108,OR4F6,OR4F15,OR4F13P,OR4F4 | -0.0153865 | 279.299 | 206 | 119.819 |
| 2 | 16 | 67322658 | 67424155 | PLEKHG4,PLEKHG4,KCTD19,KCTD19,LRRC36,TPPP3 | -0.0154464 | 281.332 | 43 | 26.5773 |
| 2 | 5 | 1.31E+08 | 131876977 | FNIP1,ACSL6,IL3,CSF2,P4HA2,P4HA2,MIR6830,PDLIM4,SLC22A4,SLC22A4,MIR3936HG,MIR3936HG,MIR3936,SLC22A5,C5orf56,IRF1 | -0.0163786 | 270.162 | 107 | 65.8086 |

| | | | | | | | | |
|---|---|---|---|---|---|---|---|---|
| 2 | 12 | 50854882 | 59307196 | LARP4,DIP2B,ATF1,TMPRSS12,METTL7A,HIGD1C,SLC11A2,LETMD1,LETMD1,CSRNP2,CSRNP2,TFCP2,POU6F1,DAZAP2,DAZAP2,SMAGP,SMAGP,BIN2,CELA1,GALNT6,SLC4A8,SCN8A,FIGNL2,ANKRD33,ACVRL1,ACVR1B,GRASP-AS1,GRASP,GRASP,NR4A1,ATG101,OR7E47P,KRT80,C12orf80,KRT7,KRT7,KRT7-AS,KRT87P,KRT86,KRT81,KRT86,KRT83,KRT85,KRT84,KRT82,KRT75,KRT6B,KRT6C,KRT6A,KRT5,KRT71,KRT74,KRT72,KRT73,KRT73,KRT73-AS1,KRT2,KRT1,KRT77,KRT76,KRT3,KRT4,KRT79,KRT78,KRT8,KRT8,KRT18,KRT18,EIF4B,LOC283335,TNS2,TNS2,TNS2,MIR6757,SPRYD3,IGFBP6,SOAT2,CSAD,CSAD,ZNF740,ZNF740,ITGB7,RARG,MFSD5,ESPL1,PFDN5,C12orf10,AAAS,SP7,SP1,AMHR2,PRR13,PCBP2,PCBP2,PCBP2-OT1,PCBP2,MAP3K12,MAP3K12,TARBP2,NPFF,ATF7,LOC100652999,ATF7,ATP5MC2,CALCOCO1,HOXC13-AS,HOXC13,HOXC13,HOXC12,HOTAIR,HOXC11,HOXC11,HOXC-AS3,HOXC10,HOXC10,MIR196A2,HOXC9,HOXC8,HOXC6,HOXC5,HOXC4,HOXC5,HOXC4,HOXC5,HOXC4,MIR615,HOXC4,FLJ12825,LOC100240734,LINC02381,SMUG1,CBX5,MIR3198-2,CBX5,HNRNPA1,HNRNPA1P10,NFE2,COPZ1,COPZ1,MIR148B,LOC102724050,GPR84,LOC102724050,ZNF385A,LOC102724050,ITGA5,LOC102724050,GTSF1,GTSF1,NCKAP1L,PDE1B,PPP1R1A,GLYCAM1,LACRT,DCD,MUCL1,TESPA1,NEUROD4,OR9K2,OR10A7,OR6C74,OR6C6,OR6C1,OR6C3,OR6C75,OR6C65,OR6C76,OR6C2,OR6C70,OR6C68,OR6C4,OR2AP1,OR10P1,METTL7B,ITGA7,BLOC1S1,BLOC1S1-RDH5,BLOC1S1-RDH5,RDH5,CD63,GDF11,SARNP,ORMDL2,DNAJC14,T | -0.0167126 | 265.557 | 2606 | 1597.22 |
| 2 | 14 | 94741693 | 95570427 | PPP4R4,SERPINA10,SERPINA6,SERPINA2,SERPINA1,SERPINA11,SERPINA9,SERPINA12,SERPINA4,SERPINA5,SERPINA3,SERPINA13P,GSC,DICER1 | -0.0195623 | 235.55 | 98 | 63.763 |
| 2 | 22 | 38978850 | 39112723 | FAM227A,CBY1,TOMM22,JOSD1,GTPBP1 | -0.0202469 | 242.25 | 37 | 22.1025 |
| 2 | 15 | 91503497 | 93483006 | RCCD1,PRC1,PRC1-AS1,PRC1,VPS33B,SV2B,SLCO3A1,ST8SIA2,C15orf32,FAM174B,ASB9P1,CHD2,CHD2,MIR3175 | -0.0244002 | 259.524 | 125 | 75.1202 |
| 2 | 2 | 70131926 | 71577454 | MXD1,ASPRV1,PCBP1-AS1,PCBP1,C2orf42,TIA1,PCYOX1,SNRPG,FAM136A,TGFA,ADD2,FIGLA,CLEC4F,CD207,VAX2,ATP6V1B1,ATP6V1B1,ATP6V1B1-AS1,ANKRD53,TEX261,NAGK,MCEE,MPHOSPH10,PAIP2B,ZNF638 | -0.0262525 | 262.953 | 218 | 129.867 |

| | | | | | | | | |
|---|---|---|---|---|---|---|---|---|
| 2 | 2 | 2.34E+08 | 237995945 | UGT1A8,UGT1A8,UGT1A10,UGT1A8,UGT1A10,UGT1A9,UGT1A8,UGT1A10,UGT1A9,UGT1A7,UGT1A8,UGT1A10,UGT1A9,UGT1A7,UGT1A6,UGT1A8,UGT1A10,UGT1A9,UGT1A7,UGT1A6,UGT1A5,UGT1A8,UGT1A10,UGT1A9,UGT1A7,UGT1A6,UGT1A5,UGT1A4,UGT1A8,UGT1A10,UGT1A9,UGT1A7,UGT1A6,UGT1A5,UGT1A4,UGT1A3,UGT1A8,UGT1A10,UGT1A9,UGT1A7,UGT1A6,UGT1A5,UGT1A4,UGT1A3,DNAJB3,UGT1A8,UGT1A10,UGT1A9,UGT1A7,UGT1A6,UGT1A5,UGT1A4,UGT1A3,LOC100286922,UGT1A8,UGT1A10,UGT1A9,UGT1A7,UGT1A6,UGT1A5,UGT1A4,UGT1A3,UGT1A1,MROH2A,HJURP,MSL3P1,TRPM8,SPP2,ARL4C,SH3BP4,AGAP1,GBX2,ASB18,IQCA1,ACKR3,COPS8 | -0.0291074 | 267.428 | 296 | 168.822 |
| 2 | 1 | 2.02E+08 | 202990009 | UBE2T,PPP1R12B,SYT2,KDM5B,MGAT4EP,RABIF,KLHL12,ADIPOR1,CYB5R1,LOC100506747,TMEM183A,TMEM183B | -0.0294839 | 253.101 | 147 | 84.3025 |
| 2 | 15 | 80429761 | 83424097 | ZFAND6,FAH,ARNT2,ARNT2,LOC101929586,ARNT2,MIR5572,ABHD17C,CEMIP,MIR549A,CEMIP,CEMIP,MESD,MESD,MIR4514,TLNRD1,CFAP161,IL16,STARD5,TMC3-AS1,TMC3,MEX3B,LINC01583,EFL1,SAXO2,ADAMTS7P1,GOLGA6L10,UBE2Q2P2,GOLGA6L10,GOLGA6L17P,GOLGA6L9,RPS17,GOLGA6L10,GOLGA6L17P,GOLGA6L9,GOLGA6L10,GOLGA6L17P,GOLGA6L9,ADAMTS7P1,GOLGA6L17P,GOLGA6L9,UBE2Q2P2,RPS17,CPEB1,CPEB1,CPEB1-AS1,CPEB1-AS1,AP3B2,AP3B2,ACTG1P17 | -0.0297492 | 260.052 | 328 | 191.46 |
| 2 | 16 | 11992404 | 15494790 | GSPT1,NPIPB2,TNFRSF17,SNX29,CPPED1,CPPED1,MIR4718,SHISA9,ERCC4,MKL2,MIR193BHG,MIR193B,MIR193BHG,MIR365A,PARN,BFAR,PLA2G10,ABCC6P2,NOMO1,PDXDC1,PDXDC1,NTAN1,PDXDC1,RRN3,MPV17L | -0.0310115 | 263.867 | 264 | 144.41 |
| 2 | X | 67519492 | 69516923 | OPHN1,YIPF6,STARD8,EFNB1,PJA1,LINC00269,FAM155B,EDA,EDA,MIR676,AWAT2,OTUD6A,IGBP1,DGAT2L6,AWAT1,P2RY4,ARR3,RAB41,PDZD11,KIF4A | -0.0312763 | 255.703 | 162 | 97.5357 |
| 2 | X | 60500 | 8667186 | PLCXD1,GTPBP6,PPP2R3B,SHOX,CRLF2,CSF2RA,IL3RA,SLC25A6,ASMTL-AS1,ASMTL,ASMTL,P2RY8,AKAP17A,AKAP17A,ASMT,ASMT,DHRSX,DHRSX,ZBED1,CD99,XG,XG,XGY2,GYG2,ARSD,ARSD-AS1,ARSD,ARSE,ARSH,ARSF,LINC01546,MXRA5,PRKX,LOC389906,FAM239B,NLGN4X,MIR4770,PUDP,PUDP,STS,MIR4767,PUDP,STS,STS,PNPLA4,MIR651,VCX2,ANOS1 | -0.0321118 | 209.323 | 456 | 231.713 |
| 2 | 1 | 1.19E+08 | 121484934 | SPAG17,TBX15,WARS2,WARS2,LOC101929147,HAO2,HSD3B2,HSD3B1,ZNF697,PHGDH,HMGCS2,REG4,NBPF7,ADAM30,NOTCH2,FCGR1B,SRGAP2-AS1,SRGAP2D,SRGAP2B,SRGAP2C,EMBP1 | -0.034011 | 282.954 | 220 | 127.3 |
| 2 | 7 | 39125547 | 43846908 | POU6F2,YAE1D1,RALA,CDK13,MPLKIP,SUGCT,INHBA,INHBA,INHBA-AS1,GLI3,C7orf25,PSMA2,MRPL32,HECW1,HECW1,HECW1-IT1,MIR3943,HECW1,LUARIS,STK17A,STK17A,COA1,COA1,BLVRA | -0.0356936 | 246.48 | 250 | 143.244 |

| | | | | | | | | |
|---|---|---|---|---|---|---|---|---|
| 2 | 16 | 18794418 | 27077617 | RPS15A,ARL6IP1,SMG1,TMC7,LOC102723385,COQ7,COQ7,ITPRIPL2,SYT17,CLEC19A,TMC5,GDE1,CCP110,C16orf62,KNOP1,IQCK,GPRC5B,GPR139,GP2,UMOD,PDILT,ACSM5,ACSM2A,ACSM2B,ACSM1,THUMPD1,ACSM3,ACSM3,ERI2,ERI2,ERI2,REXO5,REXO5,DCUN1D3,LYRM1,DNAH3,DNAH3,TMEM159,TMEM159,ZP2,ANKS4B,CRYM,CRYM-AS1,SNX29P1,MIR3680-2,MIR3680-1,SLC7A5P2,LOC101927814,METTL9,METTL9,METTL9,IGSF6,OTOA,RRN3P1,UQCRC2,PDZD9,C16orf52,VWA3A,EEF2K,POLR3E,CDR2,RRN3P3,LOC653786,HS3ST2,USP31,SCNN1G,SCNN1B,COG7,GGA2,EARS2,UBFD1,NDUFAB1,PALB2,DCTN5,PLK1,ERN2,CHP2,PRKCB,CACNG3,RBBP6,TNRC6A,LINC01567,TNRC6A,SLC5A11,ARHGAP17,LOC554206,LCMT1,AQP8,ZKSCAN2,HS3ST4,HS3ST4,MIR548W | -0.0386806 | 274.533 | 1228 | 739.273 |
| 2 | 18 | 76934141 | 78016748 | ATP9B,NFATC1,LOC284241,CTDP1,KCNG2,PQLC1,HSBP1L1,TXNL4A,RBFA,ADNP2,PARD6G-AS1,PARD6G-AS1,PARD6G,PARD6G | -0.0387483 | 260.96 | 151 | 85.9872 |
| 2 | 3 | 47168960 | 48446107 | SETD2,KIF9-AS1,KIF9,KIF9,KLHL18,PTPN23,SCAP,ELP6,CSPG5,SMARCC1,DHX30,DHX30,MIR1226,MAP4,CDC25A,MIR4443,CAMP,ZNF589,NME6,SPINK8,SPINK8,MIR2115,FBXW12,PLXNB1 | -0.0397654 | 257.495 | 284 | 177.098 |
| 2 | 16 | 81061785 | 85144030 | CENPN,ATMIN,C16orf46,GCSH,PKD1L2,BCO1,GAN,MIR4720,CMIP,PLCG2,SDR42E1,HSD17B2,MPHOSPH6,CDH13,CDH13,MIR3182,HSBP1,MLYCD,OSGIN1,NECAB2,SLC38A8,MBTPS1,HSDL1,DNAAF1,DNAAF1,TAF1C,TAF1C,ADAD2,ADAD2,LOC654780,KCNG4,WFDC1,ATP2C2,ATP2C2,ATP2C2-AS1,TLDC1,COTL1,KLHL36,USP10,CRISPLD2,ZDHHC7,KIAA0513,FAM92B | -0.0400341 | 255.211 | 544 | 321.631 |
| 2 | 8 | 32611897 | 38965410 | NRG1,FUT10,MAK16,MAK16,TTI2,TTI2,RNF122,DUSP26,UNC5D,UNC5D,LOC101929550,KCNU1,LINC01605,ZNF703,LOC102723701,ERLIN2,ERLIN2,LOC728024,PLPBP,ADGRA2,BRF2,RAB11FIP1,GOT1L1,ADRB3,EIF4EBP1,ASH2L,STAR,LSM1,BAG4,DDHD2,PLPP5,NSD3,LETM2,FGFR1,C8orf86,RNF5P1,TACC1,PLEKHA2,HTRA4,TM2D2,ADAM9,ADAM32 | -0.0465009 | 259.211 | 509 | 302.058 |
| 2 | 2 | 27605001 | 27898601 | PPM1G,NRBP1,KRTCAP3,KRTCAP3,IFT172,IFT172,FNDC4,GCKR,C2orf16,ZNF512,CCDC121,CCDC121,GPN1,GPN1,SUPT7L,SUPT7L,SLC4A1AP,SLC4A1AP | -0.0472853 | 264.551 | 194 | 124.673 |
| 2 | 17 | 45266402 | 45405068 | CDC27,MYL4,ITGB3,ITGB3,THCAT158 | -0.0519161 | 247.492 | 30 | 18.3528 |
| 2 | 12 | 1.07E+08 | 110564710 | CRY1,BTBD11,PWP1,PRDM4,PRDM4,LOC101929162,ASCL4,WSCD2,CMKLR1,FICD,SART3,ISCU,TMEM119,SELPLG,MIR4496,CORO1C,SSH1,SSH1,MIR619,DAO,SVOP,USP30,USP30-AS1,USP30,ALKBH2,UNG,ACACB,FOXN4,MYO1H,KCTD10,UBE3B,MMAB,MVK,FAM222A,FAM222A-AS1,TRPV4,TRPV4,MIR4497,GLTP,TCHP,GIT2,ANKRD13A,C12orf76 | -0.054112 | 249.534 | 521 | 318.236 |

| | | | | | | | | |
|---|---|---|---|---|---|---|---|---|
| 2 | 11 | 1.26E+08 | 134946016 | CDON,RPUSD4,FAM118B,SRPRA,FOXRED1,TIRAP,DCPS,DCPS,GSEC,ST3GAL4,KIRREL3,KIRREL3,LOC101929427,KIRREL3-AS3,LOC101929473,ETS1,ETS1,LOC101929517,LOC101929538,FLI1,FLI1,SENCR,FLI1,KCNJ1,KCNJ5,C11orf45,KCNJ5,TP53AIP1,ARHGAP32,BARX2,TMEM45B,NFRKB,PRDM10,LINC00167,APLP2,ST14,ZBTB44,LOC646383,ADAMTS8,ADAMTS15,C11orf44,LINC02551,SNX19,NTM,OPCML,SPATA19,MIR4697HG,MIR4697,IGSF9B,JAM3,NCAPD3,VPS26B,THYN1,ACAD8,GLB1L3,GLB1L2,B3GAT1,LOC100507548 | -0.0541717 | 243.683 | 649 | 396.505 |
| 2 | 20 | 55919010 | 58422336 | RAE1,LOC100291105,RBM38,RBM38,CTCFL,PCK1,ZBP1,PMEPA1,PMEPA1,NKILA,MIR4532,C20orf85,ANKRD60,PPP4R1L,RAB22A,VAPB,APCDD1L,LINC01711,STX16,STX16-NPEPL1,STX16-NPEPL1,NPEPL1,MIR296,MIR298,GNAS-AS1,GNAS,GNAS,NELFCD,CTSZ,TUBB1,ATP5F1E,SLMO2-ATP5E,SLMO2-ATP5E,PRELID3B,ZNF831,EDN3,PHACTR3 | -0.0554643 | 256.37 | 310 | 181.193 |
| 2 | 10 | 1.17E+08 | 118099717 | ATRNL1,GFRA1,CCDC172 | -0.0593669 | 179.023 | 29 | 15.5545 |
| 2 | 8 | 26227652 | 27593190 | PPP2R2A,BNIP3L,PNMA2,DPYSL2,ADRA1A,STMN4,TRIM35,PTK2B,CHRNA2,EPHX2,CLU,CLU,MIR6843,SCARA3,MIR3622B,MIR3622A | -0.0611181 | 251.596 | 170 | 104.129 |
| 2 | X | 1.51E+08 | 151919056 | PASD1,PRRG3,FATE1,CNGA2,MAGEA4,GABRE,GABRE,MIR224,GABRE,MIR452,MAGEA5,MAGEA10-MAGEA5,MAGEA10-MAGEA5,MAGEA10,GABRA3,GABRA3,MIR105-1,GABRA3,MIR767,GABRA3,MIR105-2,GABRQ,MAGEA6,CSAG3,MAGEA2,MAGEA2B,CSAG4,MAGEA12,CSAG1 | -0.0619981 | 265.832 | 115 | 65.4622 |
| 2 | 8 | 22675088 | 23701781 | PEBP4,RHOBTB2,TNFRSF10B,TNFRSF10B,LOC286059,LOC254896,TNFRSF10C,TNFRSF10C,TNFRSF10D,TNFRSF10A,CHMP7,R3HCC1,LOXL2,LOXL2,LOC100507156,ENTPD4,SLC25A37,NKX3-1,NKX2-6 | -0.0624428 | 269.056 | 144 | 86.4332 |
| 2 | 18 | 72022373 | 76870591 | FAM69C,CNDP2,CNDP1,LINC00909,ZNF407,ZADH2,TSHZ1,SMIM21,ZNF516,C18orf65,ZNF236,MBP,GALR1,SALL3,ATP9B | -0.0630007 | 242.71 | 236 | 142.95 |
| 2 | 1 | 1.62E+08 | 161692912 | FCGR2C,HSPA7,FCGR3B,FCGR2B,FCRLA,FCRLB | -0.0659916 | 243.273 | 49 | 24.6814 |
| 2 | 2 | 15769687 | 17691591 | DDX1,LINC01804,MYCNOS,MYCN,MYCN,FAM49A | -0.0661534 | 193.034 | 39 | 22.4785 |
| 2 | 11 | 1.11E+08 | 112013780 | ARHGAP20,C11orf53,COLCA1,COLCA2,MIR4491,POU2AF1,BTG4,LOC728196,MIR34B,LOC728196,MIR34C,C11orf88,LAYN,SIK2,SIK2,PPP2R1B,PPP2R1B,ALG9,FDXACB1,C11orf1,CRYAB,CRYAB,HSPB2,HSPB2-C11orf52,HSPB2,HSPB2-C11orf52,HSPB2-C11orf52,HSPB2-C11orf52,C11orf52,DIXDC1,DLAT,DLAT,PIH1D2,PIH1D2,NKAPD1,TIMM8B,TIMM8B,SDHD,SDHD | -0.0677924 | 268.07 | 220 | 130.515 |
| 2 | 1 | 44125975 | 44365024 | KDM4A,KDM4A,KDM4A-AS1,ST3GAL3 | -0.0712952 | 266.516 | 42 | 24.2647 |

| | | | | | | | | |
|---|---|---|---|---|---|---|---|---|
| 2 | 8 | 96070687 | 121457931 | NDUFAF6,MIR3150BHG,MIR3150B,MIR3150A,NDUFAF6,PLEKHF2,C8orf37,C8orf37,C8orf37-AS1,GDF6,UQCRB,MTERF3,PTDSS1,LOC102724804,SDC2,CPQ,CPQ,LOC101927066,LOC101927066,TSPYL5,MTDH,LAPTM4B,MATN2,RPL30,RPL30,SNORA72,ERICH5,RIDA,POP1,NIPAL2,KCNS2,STK3,OSR2,VPS13B,VPS13B,MIR599,MIR875,COX6C,RGS22,RGS22,MIR1273A,FBXO43,POLR2K,SPAG1,RNF19A,ANKRD46,SNX31,PABPC1,YWHAZ,ZNF706,NACAP1,GRHL2,NCALD,MIR5680,RRM2B,UBR5,ODF1,KLF10,GASAL1,AZIN1,AZIN1-AS1,ATP6V1C1,LINC01181,BAALC-AS2,BAALC-AS2,BAALC,BAALC,MIR3151,BAALC,FZD6,CTHRC1,SLC25A32,SLC25A32,DCAF13,DCAF13,LOC105375690,RIMS2,RIMS2,DCSTAMP,DPYS,LRP12,ZFPM2,ZFPM2,ZFPM2-AS1,OXR1,ABRA,ANGPT1,RSPO2,EIF3E,EMC2,TMEM74,TRHR,NUDCD1,ENY2,PKHD1L1,EBAG9,SYBU,SYBU,LOC100132813,KCNV1,CSMD3,CSMD3,MIR2053,TRPS1,EIF3H,UTP23,RAD21,RAD21,RAD21-AS1,MIR3610,RAD21-AS1,AARD,SLC30A8,MED30,EXT1,SAMD12,SAMD12,SAMD12-AS1,TNFRSF11B,COLEC10,LOC101927513,COLEC10,MAL2,MAL2,MAL2-AS1,NOV,ENPP2,TAF2,DSCC1,DEPTOR,COL14A1,MRPL13,MTBP | -0.0717071 | 284.292 | 1581 | 904.19 |
| 2 | 1 | 1.47E+08 | 149783314 | NBPF19,GPR89B,NBPF19,PDE4DIPP1,NBPF19,MIR5087,NBPF25P,FAM231D,LOC388692,LOC100996741,FCGR1CP,HIST2H3PS2,LOC101929798,HIST2H2BF,FCGR1A,HIST2H2BF | -0.0722449 | 192.616 | 40 | 16.5714 |
| 2 | 4 | 52771226 | 56316420 | DCUN1D4,LRRC66,SGCB,SPATA18,USP46,DANCR,MIR4449,DANCR,SNORA26,ERVMER34-1,RASL11B,SCFD2,FIP1L1,LNX1,LNX1,LNX1-AS1,RPL21P44,CHIC2,GSX2,PDGFRA,KIT,KDR,SRD5A3,SRD5A3,SRD5A3-AS1,TMEM165,CLOCK | -0.0738399 | 250.645 | 267 | 161.851 |
| 2 | 18 | 14851633 | 15381134 | ANKRD30B | -0.074106 | 156.285 | 14 | 6.85847 |
| 2 | 1 | 2.47E+08 | 247265220 | AHCTF1,ZNF695,ZNF670-ZNF695,ZNF670-ZNF695,ZNF670,ZNF669 | -0.0752492 | 282.171 | 31 | 16.9517 |
| 2 | 8 | 22104630 | 22397042 | POLR3D,PIWIL2,SLC39A14,PPP3CC | -0.075962 | 237.813 | 71 | 41.8401 |
| 2 | 2 | 10500 | 8937054 | FAM110C,SH3YL1,ACP1,ALKAL2,TMEM18,LOC101060391,SNTG2,SNTG2,TPO,PXDN,MYT1L,EIPR1,TRAPPC12,ADI1,RNASEH1,RPS7,COLEC11,ALLC,DCDC2C,SOX11,CMPK2,RSAD2,RNF144A,RNF144A,LOC101929452,LINC00299,ID2,KIDINS220 | -0.0763312 | 235.512 | 413 | 237.277 |

| | | | | | | | | |
|---|---|---|---|---|---|---|---|---|
| 2 | 11 | 1.12E+08 | 118622147 | BCO2,PTS,PLET1,PLET1,LOC100132686,LOC101928847,NCAM1,NCAM1,NCAM1,NCAM1-AS1,TTC12,ANKK1,DRD2,DRD2,MIR4301,TMPRSS5,ZW10,CLDN25,USP28,HTR3B,HTR3A,ZBTB16,NNMT,C11orf71,RBM7,REXO2,NXPE1,NXPE4,NXPE2,CADM1,BUD13,ZPR1,APOA5,APOA4,APOC3,APOA1,APOA1,APOA1-AS,APOA1-AS,SIK3,SIK3,PAFAH1B2,SIDT2,SIDT2,LOC100652768,TAGLN,TAGLN,PCSK7,PCSK7,RNF214,BACE1,BACE1,BACE1-AS,CEP164,DSCAML1,FXYD2,FXYD6-FXYD2,FXYD6-FXYD2,FXYD6-FXYD2,FXYD6,TMPRSS13,IL10RA,SMIM35,SMIM35,TMPRSS4,TMPRSS4,SCN4B,SCN2B,JAML,MPZL3,MPZL2,CD3E,CD3D,CD3G,UBE4A,UBE4A,LOC100131626,LOC100131626,ATP5MG,KMT2A,KMT2A,LOC101929089,LOC101929089,TTC36,TTC36,TMEM25,TMEM25,TMEM25,IFT46,IFT46,ARCN1,PHLDB1,PHLDB1,MIR6716,TREH | -0.0823009 | 244.429 | 920 | 561.242 |
| 2 | 3 | 39135376 | 44283963 | WDR48,GORASP1,GORASP1,TTC21A,TTC21A,TTC21A,MIR6822,CSRNP1,XIRP1,CX3CR1,CCR8,SLC25A38,RPSA,RPSA,SNORA6,RPSA,SNORA62,MOBP,MYRIP,MYRIP,EIF1B-AS1,EIF1B,ENTPD3,ENTPD3,ENTPD3-AS1,RPL14,ZNF619,ZNF620,ZNF621,CTNNB1,ULK4,TRAK1,CCK,LYZL4,VIPR1,VIPR1,VIPR1-AS1,SEC22C,SEC22C,SS18L2,SEC22C,NKTR,NKTR,LOC101928323,ZBTB47,KLHL40,HHATL,CCDC13,CCDC13,CCDC13-AS1,HIGD1A,ACKR2,CYP8B1,ZNF662,KRBOX1,FAM198A,POMGNT2,SNRK,SNRK,SNRK-AS1,ANO10,ANO10,ABHD5,ABHD5,MIR138-1,TOPAZ1 | -0.0852202 | 233.961 | 527 | 322.506 |
| 2 | 9 | 1.24E+08 | 128728260 | GSN,GSN-AS1,GSN,STOM,GGTA1P,DAB2IP,TTLL11,MIR4478,NDUFA8,MORN5,LHX6,RBM18,MRRF,PTGS1,OR1J1,OR1J2,OR1J4,OR1N1,OR1N2,OR1L8,OR1Q1,OR1B1,OR1L1,OR1L3,OR1L4,OR1L6,OR5C1,OR1K1,PDCL,RC3H2,RC3H2,SNORD90,ZBTB6,ZBTB26,RABGAP1,RABGAP1,GPR21,MIR600HG,MIR600HG,MIR600,STRBP,CRB2,DENND1A,DENND1A,MIR601,LOC100505588,LHX2,NEK6,PSMB7,LOC100129034,PSMB7,NR5A1,NR6A1,NR6A1,MIR181A2HG,MIR181A2,NR6A1,MIR181A2HG,MIR181B2,OLFML2A,WDR38,RPL35,ARPC5L,GOLGA1,SCAI,PPP6C,RABEPK,HSPA5,GAPVD1,MAPKAP1,PBX3 | -0.0866384 | 250.878 | 675 | 399.33 |

| | | | | | | | | |
|---|---|---|---|---|---|---|---|---|
| 2 | 11 | 1.2E+08 | 125441928 | ARHGEF12,GRIK4,GRIK4,LOC105369532,GRIK4,LOC101929227,GRIK4,LOC101929208,TBCEL,TECTA,SC5D,SORL1,MIR100HG,MIR125B1,MIR100HG,BLID,MIR100HG,MIRLET7A2,MIR100HG,MIR100,MIR100HG,UBASH3B,CRTAM,JHY,BSX,HSPA8,CLMP,MIR4493,LOC100128242,GRAMD1B,SCN3B,ZNF202,OR6X1,OR6M1,TMEM225,OR8D4,OR4D5,OR6T1,OR10S1,OR10G4,OR10G9,OR10G8,OR10G7,VWA5A,OR10D3,OR8G2P,OR8G1,OR8G1,OR8G5,OR8D1,OR8D2,OR8B2,OR8B3,OR8B4,OR8B8,OR8B12,OR8A1,PANX3,TBRG1,SIAE,SIAE,SPA17,SPA17,NRGN,VSIG2,ESAM,ESAM,LOC101929340,MSANTD2,ROBO3,ROBO4,HEPN1,HEPACAM,HEPACAM,CCDC15,SLC37A2,TMEM218,PKNOX2,FEZ1,FEZ1,LOC403312,LOC403312 | -0.0868711 | 249.388 | 647 | 389.075 |
| 2 | 1 | 1.74E+08 | 176012434 | SLC9C2,ANKRD45,ANKRD45,TEX50,KLHL20,CENPL,DARS2,GAS5,SNORD81,GAS5,SNORD47,GAS5,SNORD80,GAS5,SNORD79,GAS5,SNORD78,GAS5,SNORD44,GAS5,SNORA103,SNORD77,GAS5,SNORD76,GAS5,SNORD75,GAS5,SNORD74,ZBTB37,SERPINC1,RC3H1,RABGAP1L,RABGAP1L,GPR52,CACYBP,MRPS14,TNN,KIAA0040,TNR,RFWD2,RFWD2,SCARNA3 | -0.087029 | 281.551 | 244 | 142.608 |
| 2 | 3 | 38292859 | 39104714 | OXSR1,SLC22A13,SLC22A14,XYLB,ACVR2B-AS1,ACVR2B,ACVR2B,EXOG,SCN5A,SCN10A,SCN11A,SCN11A,WDR48,WDR48 | -0.0885741 | 259.747 | 196 | 119.187 |
| 2 | 9 | 97190620 | 100777659 | MFSD14B,PCAT7,FBP2,FBP2,FBP1,C9orf3,C9orf3,MIR2278,C9orf3,LOC101928119,C9orf3,MIR23B,MIR27B,C9orf3,MIR3074,MIR24-1,FANCC,PTCH1,PTCH1,LOC100507346,ERCC6L2,LINC00092,LOC158434,HSD17B3,HSD17B3,HSD17B3-AS1,SLC35D2,ZNF367,HABP4,CDC14B,AAED1,ZNF510,ZNF782,NUTM2G,MFSD14C,CTSV,ANKRD18CP,LOC100499484,LOC100499484-C9ORF174,LOC100499484-C9ORF174,LOC100499484-C9ORF174,CCDC180,LOC100499484-C9ORF174,CCDC180,MIR1302-8,TDRD7,TMOD1,TMOD1,TSTD2,TSTD2,NCBP1,XPA,FOXE1,TRMO,HEMGN,ANP32B | -0.0896223 | 256.6 | 486 | 282.249 |
| 2 | 18 | 47918477 | 48421761 | SKA1,MAPK4,MRO | -0.0986062 | 259.249 | 22 | 13.5117 |
| 2 | 3 | 45785030 | 46666805 | SACM1L,SLC6A20,LZTFL1,LZTFL1,CCR9,FYCO1,FYCO1,CXCR6,XCR1,CCR1,CCR3,CCR2,LOC102724297,CCR5,CCRL2,LTF,RTP3,LRRC2,TDGF1,FAM240A | -0.0997594 | 266.37 | 152 | 95.7422 |
| 2 | 16 | 67037389 | 67180283 | CES4A,CBFB,C16orf70 | -0.0999534 | 235.493 | 33 | 19.5517 |
| 2 | 9 | 1.17E+08 | 123744211 | TMEM268,TEX48,TNFSF15,TNFSF8,TNC,DEC1,PAPPA,PAPPA,PAPPA-AS1,ASTN2,ASTN2,TRIM32,ASTN2,SNORA70C,TLR4,BRINP1,MIR147A,CDK5RAP2,MEGF9,FBXW2,PSMD5,CUTALP,PHF19,TRAF1,C5-OT1,C5,C5 | -0.102234 | 228.662 | 351 | 208.933 |

| | | | | | | | | |
|---|---|---|---|---|---|---|---|---|
| 2 | 5 | 1.34E+08 | 137225338 | DDX46,C5orf24,TXNDC15,PCBD2,CATSPER3,PITX1,PITX1,C5orf66,C5orf66,C5orf66-AS2,C5orf66,C5orf66,H2AFY,H2AFY,DCANP1,TIFAB,NEUROG1,CXCL14,SLC25A48,LINC01959,SLC25A48,MIR5692C1,SLC25A48,IL9,FBXL21,LECT2,TGFBI,VTRNA2-1,SMAD5-AS1,SMAD5,SMAD5,SMIM32,TRPC7,TRPC7,TRPC7-AS2,SPOCK1,KLHL3,KLHL3,MIR874,HNRNPA0,NPY6R,MYOT,PKD2L2 | -0.108359 | 230.671 | 237 | 138.656 |
| 2 | 10 | 35427339 | 37431236 | CREM,CCNY,GJD4,FZD8,FZD8,MIR4683,ANKRD30A | -0.108605 | 239.585 | 75 | 42.4902 |
| 2 | 17 | 31203740 | 33255226 | MYO1D,TMEM98,SPACA3,ASIC2,CCL2,CCL7,CCL11,CCL8,CCL13,CCL1,C17orf102,TMEM132E,CCT6B | -0.110319 | 228.859 | 90 | 51.9606 |
| 2 | 10 | 98346485 | 98740011 | TM9SF3,PIK3AP1,MIR607,LCOR | -0.110736 | 211.579 | 34 | 20.095 |
| 2 | 11 | 73388909 | 79442211 | RAB6A,MRPL48,COA4,PAAF1,DNAJB13,UCP2,UCP3,C2CD3,PPME1,P4HA3,P4HA3,P4HA3-AS1,PGM2L1,MIR548AL,KCNE3,LIPT2,LIPT2,LOC100287896,LOC100287896,POLD3,CHRDL2,CHRDL2,MIR4696,RNF169,XRRA1,SPCS2,NEU3,OR2AT4,SLCO2B1,TPBGL,ARRB1,ARRB1,MIR326,RPS3,RPS3,SNORD15A,RPS3,SNORD15B,KLHL35,GDPD5,SERPINH1,LOC105369391,MAP6,MAP6,MOGAT2,DGAT2,UVRAG,WNT11,THAP12,GVQW3,EMSY,LRRC32,GUCY2EP,TSKU,ACER3,B3GNT6,CAPN5,CAPN5,OMP,MYO7A,GDPD4,PAK1,AQP11,CLNS1A,RSF1,AAMDC,AAMDC,INTS4,INTS4,KCTD14,NDUFC2-KCTD14,NDUFC2-KCTD14,THRSP,NDUFC2-KCTD14,NDUFC2,ALG8,KCTD21-AS1,KCTD21,KCTD21,USP35,GAB2,NARS2,TENM4,TENM4,MIR708,TENM4,MIR5579 | -0.110778 | 240.721 | 889 | 519.952 |
| 2 | 2 | 11598303 | 15601492 | E2F6,GREB1,MIR4429,GREB1,NTSR2,LPIN1,LPIN1,MIR548S,MIR4262,MIR3681HG,MIR3681,TRIB2,TRIB2,MIR3125,FAM84A,NBAS | -0.110839 | 246.421 | 170 | 101.028 |
| 2 | 2 | 54176905 | 55402384 | PSME4,ACYP2,ACYP2,TSPYL6,C2orf73,SPTBN1,SPTBN1,RPL23AP32,EML6,RTN4 | -0.111969 | 246.199 | 157 | 94.5166 |
| 2 | 1 | 1.86E+08 | 186282060 | HMCN1,PRG4,PRG4,TPR | -0.11243 | 275.024 | 128 | 81.0617 |
| 2 | 3 | 1.23E+08 | 124952521 | SEMA5B,PDIA5,PDIA5,MIR7110,SEC22A,ADCY5,HACD2,MYLK-AS1,MYLK,MYLK,CCDC14,ROPN1,KALRN,KALRN,MIR5002,UMPS,UMPS,MIR544B,ITGB5,MUC13,HEG1,SLC12A8,SLC12A8,MIR5092,ZNF148 | -0.113182 | 237.728 | 388 | 226.132 |
| 2 | 9 | 33568663 | 34343432 | ANKRD18B,ANXA2P2,PTENP1,PRSS3,UBE2R2,UBAP2,UBAP2,SNORD121B,UBAP2,SNORD121A,DCAF12,UBAP1,KIF24,NUDT2 | -0.113856 | 264.404 | 137 | 78.0385 |
| 2 | 9 | 35957194 | 38571215 | OR2S2,RECK,GLIPR2,CCIN,CLTA,GNE,RNF38,MELK,MIR4475,PAX5,PAX5,MIR4540,PAX5,MIR4476,ZCCHC7,GRHPR,ZBTB5,POLR1E,FBXO10,TOMM5,FRMPD1,TRMT10B,EXOSC3,DCAF10,SLC25A51,SHB,ALDH1B1,IGFBPL1 | -0.114182 | 246.852 | 306 | 181.034 |
| 2 | X | 46387625 | 46917784 | ZNF674,CHST7,SLC9A7,RP2,LINC01545,JADE3 | -0.116065 | 244.49 | 63 | 35.177 |
| 2 | 1 | 94374575 | 94640305 | GCLM,ABCA4,ARHGAP29 | -0.116075 | 249.593 | 68 | 42.134 |
| 2 | 8 | 1.34E+08 | 133854897 | LRRC6,TMEM71,PHF20L1 | -0.117066 | 266.196 | 49 | 27.7724 |
| 2 | 11 | 93494679 | 94152776 | C11orf54,MED17,VSTM5,HEPHL1,PANX1,IZUMO1R,GPR83 | -0.117875 | 232.055 | 65 | 39.9176 |

| | | | | | | | | |
|---|---|---|---|---|---|---|---|---|
| 2 | 17 | 33285589 | 35310450 | CCT6B,ZNF830,LIG3,RFFL,RAD51L3-RFFL,RAD51L3-RFFL,RAD51D,RAD51L3-RFFL,FNDC8,NLE1,UNC45B,SLC35G3,SLFN5,SLFN11,SLFN12,SLFN13,SLFN12L,SLFN14,SLFN14,LOC107985033,LINC02001,SNORD7,PEX12,AP2B1,RASL10B,GAS2L2,MMP28,C17orf50,MMP28,TAF15,HEATR9,CCL5,RDM1,LYZL6,CCL16,CCL14,CCL15-CCL14,CCL15-CCL14,CCL15,CCL23,CCL18,CCL3,CCL4,CCL3L3,CCL3L1,CCL4L1,CCL4L2,ZNHIT3,ZNHIT3,MYO19,MYO19,PIGW,GGNBP2,DHRS11,MRM1,LHX1,AATF | -0.119609 | 267.684 | 412 | 246.441 |
| 2 | 15 | 20044632 | 35149299 | CHEK2P2,HERC2P3,NBEAP1,POTEB,POTEB2,POTEB3,MIR5701-1,MIR5701-2,MIR5701-3,FAM30C,LOC101927079,LINC02203,LOC101927079,LINC02203,OR4M2,LOC101927079,OR4N4,OR4N3P,IGHV1OR15-1,LOC102724760,IGHV1OR15-3,LOC642131,REREP3,TUBGCP5,CYFIP1,NIPA2,NIPA1,WHAMMP3,GOLGA8IP,HERC2P2,GOLGA6L2,MIR4508,MKRN3,MAGEL2,NDN,NPAP1,SNRPN,SNHG14,SNURF,SNHG14,SNHG14,SNORD107,PWARSN,SNHG14,PWAR5,SNORD64,SNHG14,PWAR5,SNORD108,SNHG14,SNORD109B,SNORD109A,SNHG14,SNORD116-1,SNHG14,SNORD116-2,SNHG14,SNORD116-3,SNORD116-9,SNHG14,SNORD116-4,SNHG14,SNORD116-5,SNORD116-7,SNHG14,SNORD116-2,SNORD116-6,SNHG14,SNORD116-8,SNHG14,SNORD116-10,SNHG14,SNORD116-11,SNHG14,SNORD116-12,SNHG14,SNORD116-13,SNHG14,SNORD116-14,SNHG14,SNORD116-19,SNORD116-17,SNHG14,SNORD116-22,SNHG14,SNORD116-23,SNHG14,SNORD116-24,SNHG14,SNORD116-25,SNHG14,SNORD116-26,SNHG14,SNORD116-27,SNHG14,SNORD116-28,SNHG14,SNORD116-29,SNHG14,SNORD115-1,SNHG14,SNORD115-5,SNORD115-9,SNORD115-10,SNORD115-12,SNHG14,SNORD115-6,SNHG14,SNORD115-7,SNHG14,SNORD115-8,SNHG14,SNORD115-9,SNORD115-10,SNORD115-12,SNORD115-5,SNHG14,SNORD115-10,SNHG14,SNORD115-13,SNHG14,SNORD115-14,SNHG14,SNORD115-16,SNHG14,SNORD115-17,SNORD115-18,SNORD115-19,SNHG14,SNORD115-20,SNORD115-15,SNORD115-21,SNHG14,SNORD115-15,SNORD115- | -0.11985 | 274.696 | 1297 | 716.807 |
| 2 | X | 1.39E+08 | 146993762 | ATP11C,ATP11C,MIR505,CXorf66,LOC728660,SOX3,LINC00632,CDR1,MIR320D2,SPANXB1,LDOC1,SPANXA2-OT1,LOC645188,SPANXD,SPANXC,MAGEC3,MAGEC1,MAGEC2,SPANXN4,SPANXN3,SLITRK4,SPANXN2,UBE2NL,SPANXN1,SLITRK2,MIR890,MIR888,MIR892A,MIR892B,MIR891B,MIR891A,CXorf51A,CXorf51B,MIR513C,MIR513B,MIR513A1,MIR513A2,MIR506,MIR506,MIR507,MIR508,MIR514B,MIR509-1,MIR509-2,MIR509-3,MIR509-3,MIR510,FMR1 | -0.119947 | 221.36 | 201 | 109.105 |

| | | | | | | | | |
|---|---|---|---|---|---|---|---|---|
| 2 | 16 | 47698720 | 53243726 | PHKB,ABCC12,ABCC11,LONP2,LONP2,MIR5095,LONP2,MIR5095,SIAH1,MIR5095,SIAH1,MIR5095,N4BP1,CBLN1,C16orf78,ZNF423,CNEP1R1,HEATR3,PAPD5,ADCY7,ADCY7,MIR6771,BRD7,NKD1,SNX20,SNX20,LOC101927272,NOD2,CYLD,MIR3181,CYLD,LINC02168,SALL1,C16orf97,LINC00919,TOX3,LOC105371267,CHD9 | -0.120173 | 262.101 | 400 | 238.847 |
| 2 | 1 | 2.45E+08 | 247027474 | CATSPERE,DESI2,COX20,HNRNPU,LOC101928068,EFCAB2,EFCAB2,KIF26B,SMYD3,TFB2M,CNST,SCCPDH,AHCTF1 | -0.120482 | 277.387 | 195 | 113.575 |
| 2 | 7 | 45932501 | 48027023 | IGFBP1,IGFBP3,LOC730338,TNS3,C7orf65,PKD1L1,PKD1L1,C7orf69,HUS1,SUN3 | -0.122654 | 218.4 | 154 | 94.2024 |
| 2 | 21 | 47856805 | 48119395 | PCNT,DIP2A,S100B,PRMT2 | -0.125173 | 275.432 | 75 | 44.6033 |
| 2 | 11 | 67817054 | 68676176 | TCIRG1,CHKA,KMT5B,C11orf24,LRP5,PPP6R3,GAL,TESMIN,CPT1A,MRPL21,MRPL21,IGHMBP2,IGHMBP2 | -0.127664 | 274.26 | 166 | 100.244 |
| 2 | 4 | 1.29E+08 | 129864349 | LARP1B,PGRMC2,JADE1,SCLT1 | -0.128201 | 213.146 | 51 | 28.1584 |
| 2 | 16 | 3348976 | 3458404 | TIGD7,ZNF75A,OR2C1,ZSCAN32,ZNF174 | -0.130799 | 237.081 | 45 | 27.8305 |
| 2 | 4 | 1.85E+08 | 185552353 | TRAPPC11,STOX2,ENPP6,IRF2,CASP3 | -0.132209 | 239.099 | 56 | 34.5928 |
| 2 | 3 | 49227306 | 49413046 | C3orf84,CCDC36,C3orf62,C3orf62,MIR4271,USP4,GPX1,RHOA | -0.133777 | 244.007 | 62 | 36.7652 |
| 2 | 1 | 1.12E+08 | 113082898 | DENND2D,CHI3L2,CHIAP2,CHIA,PIFO,OVGP1,WDR77,WDR77,ATP5PB,ATP5PB,C1orf162,TMIGD3,TMIGD3,ADORA3,RAP1A,FAM212B,FAM212B,DDX20,DDX20,KCND3,LINC01750,CTTNBP2NL,MIR4256,WNT2B,ST7L | -0.135212 | 240.88 | 174 | 107.013 |
| 2 | 6 | 1.22E+08 | 122733995 | TBC1D32,GJA1,HSF2 | -0.136114 | 270.051 | 23 | 12.6545 |
| 2 | 3 | 1.32E+08 | 138193174 | NPHP3-ACAD11,NPHP3,TMEM108,BFSP2,BFSP2,BFSP2-AS1,CDV3,TOPBP1,TF,SRPRB,RAB6B,C3orf36,SLCO2A1,RYK,AMOTL2,AMOTL2,MIR6827,MIR4788,ANAPC13,CEP63,KY,EPHB1,PPP2R3A,MSL2,PCCB,STAG1,SLC35G2,NCK1-AS1,NCK1,IL20RB,SOX14,CLDN18,DZIP1L,A4GNT,DBR1,ARMC8,ARMC8,NME9,NME9,MRAS,ESYT3 | -0.138668 | 251.083 | 503 | 294.837 |
| 2 | 6 | 1.37E+08 | 149826905 | BCLAF1,MAP7,MAP3K5,MAP3K5,LOC101928461,PEX7,SLC35D3,IL20RA,IL22RA2,IFNGR1,OLIG3,TNFAIP3,PERP,ARFGEF3,ARFGEF3,PBOV1,HEBP2,NHSL1,NHSL1,MIR3145,FLJ46906,GVQW2,GVQW2,CCDC28A,CCDC28A,ECT2L,REPS1,ABRACL,HECA,TXLNB,CITED2,MIR3668,MIR4465,NMBR,NMBR,GJE1,VTA1,ADGRG6,HIVEP2,AIG1,ADAT2,PEX3,FUCA2,PHACTR2,LTV1,ZC2HC1B,PLAGL1,SF3B5,STX11,UTRN,EPM2A,EPM2A,LOC100507557,LOC100507557,LOC100507557,FBXO30,LOC100507557,SHPRH,SHPRH,GRM1,RAB32,ADGB,ADGB,KATNBL1P6,STXBP5-AS1,STXBP5,STXBP5,SAMD5,SASH1,UST,UST,UST-AS1,TAB2,TAB2,SUMO4,ZC3H12D,PPIL4 | -0.144659 | 255.383 | 951 | 560.667 |

| | | | | | | | | |
|---|---|---|---|---|---|---|---|---|
| 2 | 7 | 1.29E+08 | 141921080 | AHCYL2,STRIP2,SMKR1,NRF1,MIR182,MIR96,MIR183,UBE2H,ZC3HC1,KLHDC10,TMEM209,SSMEM1,CPA2,CPA4,CPA5,CPA1,CEP41,MEST,MEST,MIR335,COPG2,COPG2,TSGA13,TSGA13,KLF14,MIR29A,MIR29B1,LINC-PINT,MKLN1,PODXL,PLXNA4,FLJ40288,CHCHD3,EXOC4,EXOC4,LOC101928861,LRGUK,SLC35B4,AKR1B1,AKR1B10,AKR1B15,BPGM,CALD1,AGBL3,AGBL3,C7orf49,C7orf49,C7orf49,TMEM140,WDR91,WDR91,MIR6509,STRA8,CNOT4,NUP205,STMP1,SLC13A4,FAM180A,LUZP6,MTPN,CHRM2,LOC349160,MIR490,CHRM2,LOC349160,PTN,DGKI,CREB3L2,CREB3L2,LOC100130880,AKR1D1,MIR4468,TRIM24,SVOPL,ATP6V0A4,ATP6V0A4,TMEM213,TMEM213,KIAA1549,ZC3HAV1L,ZC3HAV1,TTC26,UBN2,LUC7L2,FMC1,C7orf55-LUC7L2,LUC7L2,C7orf55-LUC7L2,LUC7L2,C7orf55-LUC7L2,LOC100129148,KLRG2,CLEC2L,HIPK2,TBXAS1,PARP12,KDM7A,SLC37A3,RAB19,MKRN1,DENND2A,ADCK2,NDUFB2-AS1,NDUFB2-AS1,NDUFB2,NDUFB2,BRAF,MRPS33,TMEM178B,AGK,KIAA1147,WEE2-AS1,WEE2,SSBP1,TAS2R3,TAS2R4,TAS2R5,PRSS37,OR9A4,CLEC5A,TAS2R38,MGAM,MGAM2 | -0.145346 | 247.018 | 1492 | 864.764 |
| 2 | 17 | 61628678 | 61782356 | DCAF7,TACO1,MAP3K3,LIMD2,STRADA | -0.145404 | 248.215 | 41 | 24.1622 |
| 2 | 10 | 95452311 | 96087718 | FRA10AC1,LGI1,SLC35G1,PIPSL,PLCE1,PLCE1,PLCE1-AS2,PLCE1,PLCE1-AS1 | -0.148736 | 271.225 | 87 | 52.863 |
| 2 | 14 | 89343593 | 91927962 | TTC8,FOXN3,FOXN3,FOXN3-AS1,EFCAB11,TDP1,KCNK13,PSMC1,NRDE2,CALM1,TTC7B,TTC7B,LOC101928909,TTC7B,LOC105370622,RPS6KA5,RPS6KA5,DGLUCY,DGLUCY,SNORA11B,DGLUCY,GPR68,CCDC88C,PPP4R3A | -0.151847 | 211.649 | 267 | 155.534 |

| | | | | | | | | |
|---|---|---|---|---|---|---|---|---|
| 2 | 6 | 1.5E+08 | 171054567 | PPIL4,GINM1,KATNA1,LATS1,LATS1,LOC645967,NUP43,PCMT1,LRP11,LRP11,RAET1E-AS1,RAET1E-AS1,RAET1E,RAET1E-AS1,RAET1G,RAET1G,ULBP2,ULBP1,RAET1K,RAET1L,ULBP3,PPP1R14C,IYD,PLEKHG1,MTHFD1L,AKAP12,ZBTB2,RMND1,ARMT1,CCDC170,ESR1,ESR1,SYNE1,SYNE1,SYNE1,MIR3163,SYNE1,SYNE1-AS1,MYCT1,VIP,FBXO5,MTRF1L,RGS17,OPRM1,OPRM1,IPCEF1,IPCEF1,CNKSR3,SCAF8,MIR1273C,TIAM2,TIAM2,TFB1M,TFB1M,TFB1M,CLDN20,NOX3,MIR1202,ARID1B,ARID1B,MIR4466,TMEM242,ZDHHC14,ZDHHC14,MIR3692,SNX9,SYNJ2,SERAC1,GTF2H5,TULP4,TMEM181,DYNLT1,SYTL3,SYTL3,MIR3918,EZR,EZR,EZR-AS1,OSTCP1,C6orf99,RSPH3,TAGAP,LOC101929122,FNDC1,FNDC1,SOD2,SOD2,WTAP,SOD2,SOD2-OT1,SOD2,SOD2-OT1,ACAT2,ACAT2,ACAT2,TCP1,TCP1,TCP1,SNORA20,TCP1,SNORA29,MRPL18,PNLDC1,MAS1,IGF2R,SLC22A1,SLC22A2,SLC22A3,LPAL2,LPA,PLG,MAP3K4,AGPAT4,AGPAT4,AGPAT4-IT1,PRKN,PRKN,PACRG,PACRG,PACRG,PACRG-AS3,PACRG,PACRG-AS1,QKI,C6orf118,PDE10A,LINC00473,LINC00602,LINC00602,T,PRR18,SFT2D1,MPC1,RPS6KA2,RPS6KA2,MIR1913,RNASET2,MIR3939,FGFR1OP,CCR6,GPR31,TCP10L2,UNC93A,TTLL2,TCP10,LINC02538,LINC01558,AFDN-DT,AFDN,HGC6.3,KIF25,FRMD1,DACT2,SMOC2,LOC101929523,THBS2,THBS2,WDR27,WDR27,C6orf120,C6orf120,C6orf120,PHF10,PHF10,TCTE3,TCTE3,ERMARD,ERMARD,LINC00242,LINC00574,DLL1,FAM120B,FAM120B,MIR4644,PSMB1,TBP,PDCD2 | -0.158451 | 244.093 | 1950 | 1144.45 |
| 2 | 10 | 1.05E+08 | 105200277 | ATP5MD,MIR1307,PDCD11 | -0.159539 | 247.128 | 33 | 21.5633 |
| 2 | 16 | 77225084 | 81010118 | MON1B,MON1B,SYCE1L,SYCE1L,ADAMTS18,NUDT7,VAT1L,CLEC3A,WWOX,MAF,LOC102724084,DYNLRB2,CDYL2,CMC2 | -0.162607 | 210.203 | 147 | 85.8388 |
| 2 | 10 | 1.24E+08 | 131934107 | TACC2,BTBD16,PLEKHA1,PLEKHA1,MIR3941,ARMS2,HTRA1,DMBT1,C10orf120,CUZD1,FAM24B-CUZD1,FAM24B-CUZD1,FAM24B,LOC399815,FAM24A,C10orf88,PSTK,IKZF5,ACADSB,HMX3,HMX2,BUB3,GPR26,CPXM2,CHST15,OAT,NKX1-2,LHPP,FAM53B,FAM53B,FAM53B-AS1,EEF1AKMT2,ABRAXAS2,ZRANB1,CTBP2,CTBP2,MIR4296,TEX36-AS1,TEX36,TEX36,EDRF1,EDRF1,EDRF1-AS1,MMP21,UROS,UROS,MIR4484,BCCIP,BCCIP,DHX32,DHX32,FANK1,ADAM12,C10orf90,LOC728158,C10orf90,DOCK1,DOCK1,FAM196A,NPS,FOXI2,CLRN3,PTPRE,PTPRE,AS-PTPRE,MKI67,MGMT,EBF3,EBF3,MIR4297,C10orf143,CTAGE7P | -0.163311 | 244.929 | 731 | 429.97 |
| 2 | 21 | 27141424 | 28338583 | GABPA,APP,CYYR1-AS1,CYYR1,CYYR1,ADAMTS1,ADAMTS5,ADAMTS5,MIR4759 | -0.165587 | 246.777 | 79 | 46.2462 |

| | | | | | | | | |
|---|---|---|---|---|---|---|---|---|
| 2 | X | 69945788 | 72783463 | TEX11,SLC7A3,SNX12,FOXO4,CXorf65,IL2RG,MED12,NLGN3,BCYRN1,GJB1,BCYRN1,ZMYM3,BCYRN1,NONO,BCYRN1,ITGB1BP2,BCYRN1,BCYRN1,TAF1,BCYRN1,OGT,BCYRN1,GCNA,BCYRN1,CXCR3,BCYRN1,CXorf49B,CXorf49,CXorf49B,CXorf49,NHSL2,NHSL2,RTL5,FLJ44635,PIN4,PIN4,ERCC6L,RPS4X,CITED1,HDAC8,PHKA1,PHKA1,PHKA1-AS1,FAM236B,FAM236A,DMRTC1,DMRTC1B,FAM226B,FAM226A,PABPC1L2B,PABPC1L2A,NAP1L6,NAP1L2,CDX4,CHIC1 | -0.165995 | 244.737 | 482 | 284.254 |
| 2 | 2 | 86406652 | 92267022 | IMMT,IMMT,MIR4779,MRPL35,REEP1,KDM3A,CHMP3,RNF103-CHMP3,RNF103-CHMP3,RNF103,RNF103-CHMP3,RMND5A,RMND5A,CD8A,CD8B,ANAPC1P1,LOC285074,MIR4771-1,MIR4771-2,MIR4435-1,MIR4435-2,RGPD1,KRCC1,SMYD1,SMYD1,MIR4780,FABP1,THNSL2,FOXI3,TEX37,LOC101928371,EIF2AK3,EIF2AK3,RPIA,ANKRD36BP2,MIR4436A | -0.166493 | 253.227 | 411 | 211.858 |
| 2 | 3 | 52574400 | 56591389 | SMIM4,PBRM1,GNL3,GNL3,SNORD136,SNORD19,GNL3,SNORD19B,GNL3,SNORD69,GLT8D1,GLT8D1,SPCS1,SPCS1,NEK4,ITIH1,ITIH3,ITIH4,ITIH4,ITIH4-AS1,MUSTN1,TMEM110-MUSTN1,TMEM110-MUSTN1,TMEM110-MUSTN1,STIMATE,SFMBT1,RFT1,PRKCD,TKT,DCP1A,CACNA1D,CHDH,IL17RB,ACTR8,SELENOK,CACNA2D3,CACNA2D3,CACNA2D3-AS1,CACNA2D3,LRTM1,WNT5A,ERC2,ERC2,MIR3938,CCDC66 | -0.170525 | 237.005 | 479 | 289.072 |
| 2 | 6 | 50681698 | 53743946 | TFAP2D,TFAP2B,PKHD1,MIR206,LINCMD1,MIR133B,IL17A,IL17F,MCM3,PAQR8,EFHC1,TRAM2,TMEM14A,GSTA2,GSTA1,GSTA5,GSTA3,GSTA4,ICK,FBXO9,GCM1,ELOVL5,ELOVL5,MIR5685,GCLC,KLHL31,LRRC1 | -0.171204 | 239.971 | 333 | 195.754 |
| 2 | 7 | 1.51E+08 | 159128163 | NUB1,WDR86,WDR86,WDR86-AS1,WDR86-AS1,CRYGN,CRYGN,MIR3907,RHEB,PRKAG2,PRKAG2,LOC644090,GALNTL5,GALNT11,KMT2C,XRCC2,ACTR3B,DPP6,PAXIP1-AS2,PAXIP1-AS2,PAXIP1,PAXIP1,HTR5A-AS1,HTR5A-AS1,HTR5A,HTR5A,INSIG1,BLACE,LOC100286906,EN2,CNPY1,LOC100506302,RBM33,SHH,LOC389602,C7orf13,RNF32,RNF32,LMBR1,LMBR1,NOM1,MNX1,MNX1,MNX1-AS2,UBE3C,DNAJB6,LOC101927914,PTPRN2,PTPRN2,MIR153-2,PTPRN2,MIR595,LINC01022,MIR5707,NCAPG2,ESYT2,WDR60,VIPR2 | -0.171635 | 232.524 | 710 | 409.237 |
| 2 | 7 | 27843345 | 30089051 | TAX1BP1,JAZF1,JAZF1,JAZF1-AS1,JAZF1-AS1,CREB5,TRIL,LOC100506497,CPVL,CPVL,CPVL,CHN2,CHN2,PRR15,LOC646762,LOC646762,MIR550A3,WIPF3,SCRN1,FKBP14,PLEKHA8 | -0.173771 | 222.937 | 174 | 95.1043 |
| 2 | 3 | 44449680 | 44794231 | ZNF445,ZNF852,ZKSCAN7,ZNF660,ZNF660-ZNF197,ZNF660-ZNF197,ZNF197,ZNF35,ZNF502,ZNF501 | -0.174243 | 199.3 | 82 | 50.1559 |
| 2 | X | 1.24E+08 | 128580577 | TENM1,TEX13C,DCAF12L2,DCAF12L1,PRR32,ACTRT1 | -0.180828 | 229.521 | 111 | 67.2422 |

| | | | | | | | | |
|---|---|---|---|---|---|---|---|---|
| 2 | 13 | 39261431 | 41891171 | FREM2,STOML3,PROSER1,NHLRC3,LHFPL6,COG6,COG6,MIR4305,FOXO1,MIR320D1,MRPS31,SLC25A15,SLC25A15,TPTE2P5,SLC25A15,TPTE2P5,MIR621,TPTE2P5,TPTE2P5,SUGT1P3,ELF1,WBP4,MIR3168,KBTBD6,LOC101929140,KBTBD7,LOC101929140,MTRF1,MTRF1,MTRF1,NAA16,NAA16 | -0.185058 | 251.176 | 229 | 134.68 |
| 2 | X | 62042080 | 65259900 | SPIN4,ARHGEF9,MIR1468,AMER1,ASB12,MTMR8,ZC4H2,ZC3H12B,LAS1L,FRMD8P1,MSN,MIR223,VSIG4 | -0.187449 | 198.245 | 152 | 90.985 |
| 2 | 10 | 42379857 | 43694487 | CCNYL2,ZNF33B,BMS1,MIR5100,RET,CSGALNACT2,RASGEF1A | -0.189299 | 243.226 | 109 | 58.6557 |
| 2 | 2 | 95326671 | 95767550 | LOC442028,TEKT4,MAL,MRPS5 | -0.190278 | 312.951 | 25 | 13.8335 |
| 2 | X | 69606471 | 69825369 | KIF4A,GDPD2,DLG3,DLG3,DLG3-AS1,TEX11 | -0.190894 | 227.95 | 68 | 39.7411 |
| 2 | 9 | 1.15E+08 | 116260494 | UGCG,UGCG,MIR4668,SUSD1,PTBP3,HSDL2,HSDL2,C9orf147,C9orf147,C9orf147,KIAA1958,KIAA1958,INIP,SNX30,SLC46A2,ZNF883,ZFP37,FAM225B,FAM225A,SLC31A2,FKBP15,SLC31A1,CDC26,PRPF4,RNF183,WDR31,BSPRY,HDHD3,ALAD,POLE3,C9orf43,RGS3 | -0.191968 | 224.843 | 291 | 169.338 |
| 2 | 14 | 64561806 | 80669684 | SYNE2,MIR548H1,SYNE2,ESR2,TEX21P,MTHFD1,MTHFD1,ZBTB25,ZBTB25,AKAP5,ZBTB25,ZBTB1,LOC102723809,HSPA2,PPP1R36,PLEKHG3,SPTB,SPTB,MIR7855,CHURC1,CHURC1-FNTB,CHURC1-FNTB,GPX2,CHURC1-FNTB,CHURC1-FNTB,RAB15,CHURC1-FNTB,FNTB,CHURC1-FNTB,FNTB,MAX,CHURC1-FNTB,FNTB,MAX,MIR4706,MAX,MAX,LOC100506321,MIR4708,FUT8,FUT8-AS1,FUT8,MIR625,FUT8,CCDC196,GPHN,FAM71D,MPP5,ATP6V1D,EIF2S1,PLEK2,MIR5694,TMEM229B,PLEKHH1,PIGH,ARG2,ARG2,VTI1B,VTI1B,RDH11,RDH12,ZFYVE26,RAD51B,ZFP36L1,ACTN1,DCAF5,EXD2,GALNT16,ERH,ERH,SLC39A9,SLC39A9,PLEKHD1,CCDC177,SUSD6,LOC100289511,SRSF5,SRSF5,SLC10A1,SMOC1,SLC8A3,LOC646548,ADAM21P1,COX16,SYNJ2BP-COX16,SYNJ2BP-COX16,SYNJ2BP,ADAM21,ADAM20P1,ADAM20,MED6,TTC9,MAP3K9,PCNX1,SIPA1L1,RGS6,DPF3,DCAF4,ZFYVE1,RBM25,PSEN1,PAPLN,PAPLN,LOC101928123,NUMB,HEATR4,HEATR4,RIOX1,HEATR4,ACOT1,ACOT2,ACOT4,ACOT6,DNAL1,PNMA1,ELMSAN1,ELMSAN1,MIR4505,PTGR2,ZNF410,FAM161B,FAM161B,COQ6,COQ6,COQ6,ENTPD5,ENTPD5,BBOF1,BBOF1,ALDH6A1,ALDH6A1,LIN52,VSX2,ABCD4,VRTN,SYNDIG1L,NPC2,MIR4709,NPC2,ISCA2,LTBP2,AREL1,AREL1,FCF1,FCF1,YLPM1,PROX2,DLST,RPS6KL1,PGF,EIF2B2,MLH3,ACYP1,ZC2HC1C,NEK9,TMED10,FOS,JDP2,BATF,LOC102724153,FLVCR2,FLVCR2,ERG28,TTLL5,TGFB3,IFT43,GPATCH2L,ESRRB,VASH1,ANGEL1,LRRC74A,IRF2BPL,CIPC,ZDHHC22,TMEM63C,NGB,MIR1260A,NGB,POMT2,GSTZ1,TMED8,SAMD15,NOXRED1,VIPAS39,AHSA1,ISM2,SPTLC2,ALKBH1,ALKBH1,SLIRP,SLIRP,SLIRP,SNW1,SNW1,SNW1,C14orf178,C14orf178,ADCK1,NRXN3,DIO2 | -0.192266 | 234.163 | 2120 | 1273.64 |
| 2 | 10 | 33197413 | 35300956 | ITGB1,NRP1,PARD3,CUL2 | -0.195444 | 209.722 | 101 | 57.8076 |
| 2 | 15 | 43476390 | 43813295 | TMEM62,CCNDBP1,EPB42,TGM5,TGM7,LCMT2,LCMT2,ADAL,ADAL,ZSCAN29,TUBGCP4,TUBGCP4,TP53BP1,TP53BP1,MAP1A | -0.196803 | 264.745 | 155 | 98.2337 |

| | | | | | | | | |
|---|---|---|---|---|---|---|---|---|
| 2 | 5 | 1.32E+08 | 133487527 | KIF3A,CCNI2,CCNI2,SEPT8,SEPT8,SOWAHA,SHROOM1,GDF9,GDF9,UQCRQ,UQCRQ,LEAP2,AFF4,ZCCHC10,HSPA4,FSTL4,FSTL4,MIR1289-2,C5orf15,VDAC1,TCF7 | -0.196852 | 222.142 | 177 | 105.542 |
| 2 | 5 | 1.37E+08 | 138644041 | FAM13B,LOC100130172,WNT8A,NME5,BRD8,KIF20A,CDC23,GFRA3,CDC25C,CDC25C,FAM53C,FAM53C,KDM3B,REEP2,EGR1,ETF1,HSPA9,HSPA9,SNORD63,CTNNA1,CTNNA1,LRRTM2,SIL1,SNHG4,MATR3,SNORA74D,SNHG4,MATR3,SNORA74A,MATR3 | -0.19908 | 233.667 | 258 | 156.095 |
| 2 | X | 1.29E+08 | 133379001 | SLC25A14,GPR119,RBMX2,FAM45BP,ENOX2,ARHGAP36,IGSF1,OR13H1,STK26,FRMD7,RAP2C,RAP2C-AS1,MBNL3,MBNL3,HS6ST2,HS6ST2,HS6ST2-AS1,USP26,TFDP3,GPC4,GPC3,MIR363,MIR92A2,MIR19B2,MIR20B,MIR18B,MIR106A,CCDC160 | -0.199999 | 218.486 | 218 | 130.598 |
| 2 | 4 | 1.86E+08 | 191025802 | CCDC110,CCDC110,LOC105377590,LOC105377590,PDLIM3,SORBS2,TLR3,FAM149A,CYP4V2,KLKB1,F11,F11,F11-AS1,F11-AS1,MTNR1A,FAT1,ZFP42,TRIML2,TRIML1,FRG1,FRG2 | -0.203563 | 242.871 | 291 | 177.703 |
| 2 | 13 | 96684755 | 103390791 | UGGT2,HS6ST3,HS6ST3,MIR4501,OXGR1,MBNL2,RAP2A,IPO5,FARP1,RNF113B,FARP1,STK24,SLC15A1,DOCK9,UBAC2,UBAC2,GPR18,UBAC2,GPR183,UBAC2,FKSG29,UBAC2,MIR623,TM9SF2,CLYBL,CLYBL,MIR4306,CLYBL,LOC101927437,ZIC5,ZIC2,PCCA,PCCA,GGACT,GGACT,TMTC4,NALCN-AS1,NALCN,NALCN,ITGBL1,FGF14,FGF14,MIR2681,FGF14,MIR4705,TPP2,METTL21C,CCDC168 | -0.204338 | 250.828 | 566 | 331.524 |
| 2 | 11 | 2966714 | 6173242 | NAP1L4,NAP1L4,SNORA54,CARS,CARS,CARS-AS1,OSBPL5,MRGPRG,MRGPRG,MRGPRG-AS1,MRGPRG-AS1,MRGPRE,ZNF195,ART5,ART1,CHRNA10,NUP98,PGAP2,RHOG,STIM1,MIR4687,STIM1,RRM1,OR52B4,TRIM21,OR52K2,OR52K1,OR52M1,C11orf40,OR52I2,OR52I1,TRIM68,OR51D1,OR51E1,OR51E2,OR51F1,OR52R1,OR51F2,OR51S1,OR51H1,OR51T1,OR51A7,OR51G2,OR51G1,OR51A4,OR51A2,MMP26,OR51L1,OR52J3,OR52E2,OR52A5,OR52A1,OR52Z1,OR51V1,HBB,HBD,HBBP1,HBG1,HBG2,HBE1,OR51B4,OR51B2,OR51B5,OR51B5,OR51B6,OR51B5,OR51M1,OR51B5,OR51J1,OR51B5,OR51Q1,OR51B5,OR51I1,OR51B5,OR51I2,OR51B5,OR52D1,UBQLN3,UBQLNL,OR52H1,OR52B6,TRIM6,TRIM6-TRIM34,TRIM6-TRIM34,TRIM34,TRIM5,TRIM22,OR56B1,OR52N4,OR52N5,OR52N1,OR52N2,OR52E6,OR52E8,OR52E4,OR52E5,OR56A3,OR56A5,OR52L1,OR56A4,OR56A1,OR56B4 | -0.205544 | 247.795 | 644 | 390.909 |
| 2 | 3 | 1.83E+08 | 183862767 | DCUN1D1,MCCC1,LAMP3,MCF2L2,MCF2L2,B3GNT5,KLHL6,KLHL24,YEATS2,YEATS2,YEATS2-AS1,MAP6D1,PARL,ABCC5,ABCC5,ABCC5-AS1,HTR3D,HTR3C,HTR3E-AS1,HTR3E,HTR3E,EIF2B5 | -0.208625 | 231.211 | 253 | 146.984 |
| 2 | 10 | 96367745 | 97681220 | CYP2C18,CYP2C19,CYP2C9,CYP2C8,ACSM6,PDLIM1,SORBS1,ALDH18A1,TCTN3,ENTPD1,ENTPD1,ENTPD1-AS1 | -0.212315 | 223.484 | 184 | 105.543 |

| | | | | | | | | |
|---|---|---|---|---|---|---|---|---|
| 2 | 17 | 12016569 | 16454627 | MAP2K4,MYOCD,MYOCD,LOC101928418,MYOCD,LOC100128006,LOC100128006,ARHGAP44,ARHGAP44,ARHGAP44,MIR1269B,ELAC2,HS3ST3A1,CDRT15P1,COX10,CDRT15,HS3ST3B1,PMP22,PMP22,MIR4731,TEKT3,CDRT4,TVP23C-CDRT4,TVP23C-CDRT4,TVP23C,CDRT1,TRIM16,ZNF286A,TBC1D26,CDRT15P2,MEIS3P1,ADORA2B,ZSWIM7,ZSWIM7,TTC19,TTC19,NCOR1,PIGL,PIGL,MIR1288,CENPV,UBB,TRPV2,LRRC75A-AS1,LRRC75A-AS1,SNORD49B,LRRC75A-AS1,SNORD49A,LRRC75A-AS1,SNORD65,LRRC75A-AS1,LRRC75A,LRRC75A | -0.213007 | 240.28 | 380 | 218.927 |
| 2 | 15 | 62352431 | 65954339 | VPS13C,C2CD4A,C2CD4B,GOLGA2P11,MGC15885,TLN2,TLN2,MIR190A,TPM1,TPM1,TPM1-AS,LACTB,RPS27L,RAB8B,APH1B,CA12,USP3,USP3,USP3-AS1,USP3-AS1,FBXL22,FBXL22,HERC1,MIR422A,DAPK2,DAPK2,LOC101928988,FAM96A,SNX1,SNX22,SNX22,PPIB,PPIB,CSNK1G1,PCLAF,TRIP4,ZNF609,OAZ2,RBPMS2,RBPMS2,MIR1272,PIF1,PLEKHO2,ANKDD1A,SPG21,MTFMT,SLC51B,RASL12,KBTBD13,UBAP1L,PDCD7,CLPX,CILP,PARP16,IGDCC3,IGDCC4,DPP8,HACD3,INTS14,SLC24A1,SLC24A1,DENND4A,DENND4A | -0.218403 | 232.194 | 705 | 421.82 |
| 2 | 7 | 50180830 | 57938684 | SPATA48,IKZF1,FIGNL1,DDC,DDC,DDC-AS1,GRB10,COBL,POM121L12,VSTM2A,VSTM2A,VSTM2A-OT1,SEC61G,EGFR,EGFR,EGFR-AS1,LANCL2,VOPP1,FKBP9P1,SEPT14,ZNF713,MRPS17,NIPSNAP2,PSPH,CCT6A,CCT6A,SNORA15,SUMF2,PHKG1,CHCHD2,NUPR2,LOC401357,MIR4283-1,MIR4283-2,ZNF479,GUSBP10,MIR3147,ZNF716 | -0.220339 | 216.248 | 366 | 201.488 |
| 2 | 2 | 17963724 | 21361327 | MSGN1,KCNS3,RDH14,NT5C1B-RDH14,NT5C1B-RDH14,NT5C1B,MIR4757,OSR1,TTC32,WDR35,LOC101928222,MATN3,MATN3,LAPTM4A,SDC1,PUM2,RHOB,HS1BP3,GDF7,LDAH,APOB,TDRD15 | -0.220503 | 238.997 | 253 | 154.418 |
| 2 | 13 | 95248187 | 96508586 | TGDS,GPR180,SOX21,ABCC4,CLDN10,DZIP1,DNAJC3,UGGT2 | -0.22082 | 223.799 | 124 | 70.9065 |

| | | | | | | | | |
|---|---|---|---|---|---|---|---|---|
| 2 | 2 | 97783869 | 128262516 | ANKRD36,ANKRD36B,COX5B,ACTR1B,ZAP70,TMEM131,VWA3B,CNGA3,INPP4A,COA5,UNC50,MGAT4A,KIAA1211L,TSGA10,TSGA10,C2orf15,TSGA10,LIPT1,LIPT1,MITD1,MRPL30,LYG2,LYG1,TXNDC9,EIF5B,REV1,AFF3,LONRF2,CHST10,NMS,PDCL3,NPAS2,NPAS2,LOC101927142,RPL31,RPL31,TBC1D8,TBC1D8,CNOT11,SNORD89,RNF149,MIR5696,CREG2,RFX8,MAP4K4,IL1R2,IL1R1,IL1RL2,IL1RL1,IL18R1,IL18RAP,IL18RAP,MIR4772,SLC9A4,SLC9A2,MFSD9,TMEM182,LOC100287010,POU3F3,LINC01159,LOC102724691,MRPS9,MRPS9,MRPS9,UTAT33,GPR45,TGFBRAP1,C2orf49,FHL2,NCK2,C2orf40,UXS1,PLGLA,CD8B2,ST6GAL2,SLC5A7,SULT1C3,SULT1C2,SULT1C4,GCC2,GCC2,GCC2-AS1,LIMS1,RANBP2,CCDC138,EDAR,SH3RF3-AS1,SH3RF3-AS1,SH3RF3,SH3RF3,MIR4265,SH3RF3,MIR4266,SH3RF3,SEPT10,SOWAHC,RGPD5,RGPD6,LIMS3,LIMS4,LIMS3-LOC440895,MIR4267,MALL,MALL,MIR4436B1,MIR4436B2,NPHP1,MIR4436B1,MIR4436B2,LIMS3-LOC440895,LIMS3,LIMS4,RGPD6,RGPD5,BUB1,ACOXL,BCL2L11,MIR4435-2HG,MIR4435-2HG,MIR4435-1,MIR4435-2,ANAPC1,ANAPC1,MIR4771-1,MIR4771-2,MERTK,TMEM87B,FBLN7,ZC3H8,ZC3H6,RGPD8,RGPD5,TTL,LOC105373562,POLR1B,POLR1B,CHCHD5,SLC20A1,NT5DC4,NT5DC4,CKAP2L,CKAP2L,IL1A,IL1B,IL37,IL36G,IL36A,IL36B,IL36RN,IL1F10,IL1RN,PSD4,PAX8,PAX8,PAX8-AS1,CBWD2,RABL2A,SLC35F5,SLC35F5,MIR4782,LOC101060091,ACTR3,ACTR3,DPP10,DDX18,CCDC93,INSIG2,EN1,MARCO,C1QL2,STEAP3,STEAP3,STEAP3-AS1,C2orf76,DBI,TMEM37,SCTR,SCTR,LOC107105282,CFAP221,TMEM177,PTPN4,EPB41L5,TMEM185B,RALB | -0.223879 | 250.803 | 2237 | 1295.43 |
| 2 | 2 | 39607466 | 48032195 | MAP4K3,TMEM178A,THUMPD2,SLC8A1-AS1,SLC8A1,SLC8A1,C2orf91,PKDCC,LOC102723824,EML4,EML4,COX7A2L,KCNG3,MTA3,OXER1,HAAO,ZFP36L2,LINC01126,THADA,PLEKHH2,PLEKHH2,C1GALT1C1L,DYNC2LI1,DYNC2LI1,ABCG5,ABCG5,ABCG8,LRPPRC,PPM1B,SLC3A1,SLC3A1,PREPL,PREPL,CAMKMT,SIX3,SIX2,SRBD1,PRKCE,EPAS1,TMEM247,ATP6V1E2,RHOQ,RHOQ,LOC100506142,PIGF,CRIPT,SOCS5,LINC01118,LINC01119,MCFD2,MCFD2,TTC7A,TTC7A,STPG4,CALM2,LOC101927043,EPCAM,EPCAM,MIR559,MSH2,KCNK12,MSH6 | -0.228465 | 245.769 | 676 | 398.904 |
| 2 | 10 | 1.13E+08 | 115891108 | SHOC2,ADRA2A,GPAM,TECTB,ACSL5,ZDHHC6,ZDHHC6,VTI1A,VTI1A,VTI1A,MIR4295,TCF7L2,HABP2,HABP2,NRAP,NRAP,CASP7,PLEKHS1,PLEKHS1,MIR4483,DCLRE1A,NHLRC2,ADRB1,CCDC186 | -0.231238 | 235.351 | 270 | 161.68 |
| 2 | 4 | 1.3E+08 | 141295437 | PCDH10,PABPC4L,PCDH18,SLC7A11-AS1,SLC7A11,SLC7A11,NOCT,ELF2,MGARP,NDUFC1,NDUFC1,NAA15,NAA15,RAB33B,SETD7,MGST2,MGST2,MAML3,MAML3,MAML3,LOC101927516,SCOC,SCOC-AS1,SCOC | -0.233024 | 214.712 | 241 | 138.9 |

| | | | | | | | | |
|---|---|---|---|---|---|---|---|---|
| 2 | 1 | 1.68E+08 | 172520754 | MPC2,MPC2,DCAF6,DCAF6,DCAF6,MIR1255B2,GPR161,TIPRL,SFT2D2,ANKRD36BP1,TBX19,MIR557,XCL2,XCL1,DPT,ATP1B1,ATP1B1,NME7,NME7,BLZF1,BLZF1,CCDC181,CCDC181,SLC19A2,F5,SELP,SELL,SELE,METTL18,C1orf112,SCYL3,KIFAP3,METTL11B,METTL11B,MIR3119-1,MIR3119-2,LOC101928650,GORAB,GORAB,PRRX1,MROH9,FMO3,FMO3,MIR1295A,MIR1295B,FMO6P,FMO2,FMO1,FMO4,PRRC2C,MYOC,VAMP4,METTL13,DNM3,DNM3,DNM3OS,MIR214,MIR3120,DNM3,DNM3OS,MIR214,DNM3,DNM3OS,MIR199A2,C1orf105,C1orf105,PIGC,SUCO | -0.234192 | 257.74 | 578 | 340.032 |
| 2 | 11 | 6705101 | 9187567 | GVINP1,OR2AG2,OR2AG1,OR6A2,OR10A5,OR10A2,OR10A4,OR2D2,OR2D3,ZNF215,ZNF214,NLRP14,RBMXL2,MIR302E,SYT9,OLFML1,PPFIBP2,CYB5R2,OVCH2,OR5P2,OR5P3,OR5E1P,OR10A6,OR10A3,NLRP10,EIF3F,TUB,TUB,RIC3,RIC3,LMO1,STK33,TRIM66,RPL27A,RPL27A,SNORA3A,RPL27A,SNORA3B,ST5,ST5,LOC102724784,AKIP1,C11orf16,ASCL3,TMEM9B,NRIP3,SCUBE2,SCUBE2,MIR5691,DENND5A | -0.234586 | 235.042 | 444 | 270.554 |
| 2 | 3 | 1.67E+08 | 169500416 | WDR49,PDCD10,SERPINI1,GOLIM4,EGFEM1P,MIR551B,EGFEM1P,MECOM,TERC,ACTRT3,MYNN | -0.238186 | 235.249 | 121 | 72.832 |
| 2 | 9 | 1.03E+08 | 104500268 | ERP44,INVS,INVS,TEX10,TEX10,MSANTD3,MSANTD3-TMEFF1,MSANTD3-TMEFF1,TMEFF1,CAVIN4,PLPPR1,BAAT,MRPL50,ZNF189,ALDOB,TMEM246-AS1,TMEM246,RNF20,GRIN3A,GRIN3A,PPP3R2 | -0.242329 | 225.951 | 169 | 101.117 |
| 2 | 3 | 50712539 | 51696551 | MIR4787,DOCK3,DOCK3,MANF,RBM15B,DCAF1,RAD54L2 | -0.243657 | 229.346 | 159 | 93.9333 |
| 2 | 13 | 48956089 | 53036581 | RB1,LPAR6,RB1,RCBTB2,CYSLTR2,FNDC3A,MLNR,CDADC1,CAB39L,SETDB2,SETDB2-PHF11,SETDB2-PHF11,PHF11,RCBTB1,ARL11,EBPL,KPNA3,CTAGE10P,SPRYD7,DLEU2,MIR3613,DLEU2,TRIM13,DLEU2,TRIM13,KCNRG,DLEU2,KCNRG,DLEU2,MIR16-1,MIR15A,DLEU2,DLEU1,DLEU1,ST13P4,DLEU7,DLEU7,DLEU7-AS1,RNASEH2B-AS1,RNASEH2B,RNASEH2B,GUCY1B2,FAM124A,SERPINE3,SERPINE3,MIR5693,SERPINE3,INTS6,INTS6,MIR4703,WDFY2,DHRS12,TMEM272,CCDC70,ATP7B,ALG11,ALG11,UTP14C,UTP14C,NEK5,NEK3,MRPS31P5,THSD1,VPS36,CKAP2 | -0.244123 | 261.441 | 473 | 277.349 |
| 2 | 18 | 9256633 | 14105631 | ANKRD12,TWSG1,RALBP1,PPP4R1,RAB31,TXNDC2,VAPA,APCDD1,NAPG,PIEZO2,PIEZO2,MIR6788,SLC35G4,GNAL,GNAL,CHMP1B,GNAL,MPPE1,MPPE1,IMPA2,ANKRD62,CIDEA,TUBB6,TUBB6,AFG3L2,AFG3L2,PRELID3A,SPIRE1,PSMG2,CEP76,PSMG2,PTPN2,SEH1L,CEP192,LDLRAD4,LDLRAD4,MIR5190,LDLRAD4,MIR4526,FAM210A,RNMT,MC5R,MC2R,ZNF519 | -0.245348 | 255.407 | 490 | 282.555 |

| | | | | | | | | |
|---|---|---|---|---|---|---|---|---|
| 2 | 13 | 41949544 | 48878244 | NAA16,RGCC,VWA8,MIR5006,VWA8,DGKH,AKAP11,TNFSF11,FAM216B,EPSTI1,DNAJC15,ENOX1,CCDC122,LACC1,LINC00390,SMIM2-AS1,SMIM2,SMIM2,SERP2,TSC22D1,TSC22D1,TSC22D1-AS1,NUFIP1,GPALPP1,GTF2F2,GTF2F2,KCTD4,TPT1,SNORA31,TPT1,SLC25A30,COG3,ERICH6B,SPERT,SIAH3,ZC3H13,CPB2-AS1,CPB2,CPB2,LCP1,LRRC63,RUBCNL,LRCH1,ESD,HTR2A,SUCLA2,NUDT15,MED4,MED4-AS1,MED4,ITM2B,RB1 | -0.249111 | 240.97 | 561 | 322.907 |
| 2 | 2 | 2.2E+08 | 219562707 | RNF25,RNF25,STK36,STK36 | -0.250599 | 233.602 | 34 | 22.3838 |
| 2 | 1 | 1.13E+08 | 115398253 | CAPZA1,MOV10,RHOC,PPM1J,FAM19A3,SLC16A1,LRIG2,MAGI3,PHTF1,RSBN1,AP4B1-AS1,PTPN22,AP4B1-AS1,BCL2L15,AP4B1-AS1,AP4B1,AP4B1,AP4B1,DCLRE1B,DCLRE1B,HIPK1-AS1,HIPK1,HIPK1,OLFML3,SYT6,TRIM33,BCAS2,DENND2C,AMPD1,NRAS,CSDE1,SIKE1,SYCP1 | -0.252045 | 246.539 | 344 | 212.718 |
| 2 | 11 | 66888745 | 67023163 | KDM2A | -0.252419 | 234.686 | 32 | 19.5129 |
| 2 | 18 | 42281305 | 47814020 | SETBP1,SETBP1,MIR4319,SLC14A2,SLC14A1,SIGLEC15,EPG5,PSTPIP2,ATP5F1A,HAUS1,C18orf25,RNF165,LOXHD1,ST8SIA5,PIAS2,PIAS2,KATNAL2,KATNAL2,KATNAL2,ELOA2,HDHD2,IER3IP1,SKOR2,MIR4527HG,MIR4527,SMAD2,ZBTB7C,CTIF,CTIF,MIR4743,SMAD7,LOC100129878,DYM,DYM,MIR4744,DYM,C18orf32,RPL17-C18orf32,RPL17-C18orf32,MIR1539,RPL17-C18orf32,RPL17,RPL17-C18orf32,RPL17,SNORD58C,RPL17-C18orf32,RPL17,SNORD58A,RPL17-C18orf32,RPL17,SNORD58B,LIPG,ACAA2,SCARNA17,SNHG22,SNHG22,MYO5B,MYO5B,MYO5B,MIR4320,CFAP53,MBD1,CXXC1 | -0.253099 | 234.741 | 588 | 342.191 |
| 2 | 9 | 65468179 | 72915143 | LINC01410,PTGER4P2-CDK2AP2P2,LOC403323,LINC00537,MIR4477A,MIR4477B,FRG1JP,MIR1299,PGM5P2,CBWD6,CBWD5,PGM5,TMEM252,PIP5K1B,FAM122A,PIP5K1B,PIP5K1B,LOC101927069,PRKACG,FXN,TJP2,FAM189A2,APBA1,PTAR1,C9orf135,MAMDC2,MAMDC2,MAMDC2-AS1,MAMDC2,SMC5-AS1,SMC5 | -0.254312 | 224.829 | 228 | 124.237 |
| 2 | 15 | 44021371 | 44614547 | CATSPER2P1,PDIA3,ELL3,ELL3,SERF2,SERF2,SERF2-C15ORF63,SERF2,SERF2-C15ORF63,MIR1282,SERF2,SERF2-C15ORF63,SERINC4,SERF2-C15ORF63,SERINC4,SERF2-C15ORF63,HYPK,MFAP1,WDR76,FRMD5,CASC4 | -0.259377 | 242.382 | 109 | 64.4295 |

| | | | | | | | | |
|---|---|---|---|---|---|---|---|---|
| 2 | 4 | 1.46E+08 | 156269059 | OTUD4,SMAD1,SMAD1-AS1,SMAD1,MMAA,C4orf51,ZNF827,LSM6,REELD1,SLC10A7,POU4F2,TTC29,EDNRA,TMEM184C,PRMT9,ARHGAP10,ARHGAP10,MIR4799,NR3C2,DCLK2,LRBA,LRBA,LOC729558,LRBA,MAB21L2,RPS3A,RPS3A,SNORD73B,RPS3A,SNORD73A,SH3D19,PRSS48,FAM160A1,GATB,FBXW7,FBXW7,FBXW7-AS1,FBXW7,MIR3140,MIR4453HG,MIR4453,TMEM154,TIGD4,ARFIP1,FHDC1,TRIM2,TRIM2,ANXA2P1,MND1,TMEM131L,TLR2,RNF175,SFRP2,DCHS2,PLRG1,FGB,FGA,FGG,LRAT,RBM46,NPY2R,MAP9 | -0.264448 | 227.653 | 737 | 436.131 |
| 2 | 3 | 15253612 | 17299410 | CAPN7,SH3BP5-AS1,SH3BP5,SH3BP5,METTL6,EAF1,COLQ,COLQ,MIR4270,HACL1,HACL1,BTD,BTD,ANKRD28,ANKRD28,MIR3134,MIR563,GALNT15,DPH3,OXNAD1,OXNAD1,RFTN1,RFTN1,DAZL,PLCL2,PLCL2,MIR3714,TBC1D5 | -0.267897 | 222.568 | 251 | 145.064 |
| 2 | 6 | 7414459 | 13801692 | RIOK1,DSP,SNRNP48,BMP6,BLOC1S5-TXNDC5,TXNDC5,BLOC1S5-TXNDC5,PIP5K1P1,BLOC1S5-TXNDC5,BLOC1S5,EEF1E1-BLOC1S5,EEF1E1-BLOC1S5,EEF1E1,SLC35B3,TFAP2A,TFAP2A,TFAP2A-AS2,TFAP2A,TFAP2A-AS1,LINC00518,MIR5689HG,MIR5689,GCNT2,C6orf52,PAK1IP1,TMEM14C,TMEM14B,MAK,GCM2,SYCP2L,SYCP2L,LOC101928191,ELOVL2,ELOVL2,ELOVL2-AS1,SMIM13,SMIM13,ERVFRD-1,NEDD9,TMEM170B,ADTRP,HIVEP1,EDN1,PHACTR1,PHACTR1,TBC1D7-LOC100130357,PHACTR1,TBC1D7-LOC100130357,LOC100130357,TBC1D7-LOC100130357,LOC100130357,TBC1D7-LOC100130357,TBC1D7,GFOD1,SIRT5,NOL7,NOL7,RANBP9,RANBP9,MCUR1 | -0.272194 | 233.862 | 517 | 307.079 |
| 2 | 21 | 38567913 | 40574470 | TTC3,DSCR9,DSCR3,DYRK1A,KCNJ6,DSCR4,DSCR8,DSCR10,KCNJ15,ERG,ETS2,LOC400867,PSMG1,BRWD1 | -0.272595 | 216.481 | 128 | 76.4718 |
| 2 | X | 96330837 | 105855993 | DIAPH2,PCDH19,TNMD,TSPAN6,SRPX2,SYTL4,CSTF2,NOX1,XKRX,ARL13A,TRMT2B,TMEM35A,CENPI,DRP2,TAF7L,TIMM8A,BTK,RPL36A,RPL36A-HNRNPH2,RPL36A-HNRNPH2,GLA,RPL36A-HNRNPH2,HNRNPH2,ARMCX4,ARMCX1,ARMCX6,ARMCX3,ARMCX2,NXF5,ZMAT1,TCEAL2,TCEAL6,BEX5,TCP11X2,NXF2B,NXF2,TMSB15A,ARMCX5,ARMCX5-GPRASP2,ARMCX5-GPRASP2,GPRASP1,ARMCX5-GPRASP2,GPRASP2,ARMCX5-GPRASP2,BHLHB9,ARMCX5-GPRASP2,LINC00630,LINC00630,LINC00630,RAB40AL,BEX1,NXF3,BEX4,TCEAL8,TCEAL5,BEX2,TCEAL7,TCEAL9,BEX3,RAB40A,TCEAL4,TCEAL3,TCEAL1,MORF4L2,GLRA4,GLRA4,TMEM31,RAB9B,PLP1,RAB9B,TMSB15B,H2BFXP,LOC100101478,H2BFWT,H2BFM,SLC25A53,SLC25A53,ZCCHC18,FAM199X,ESX1,IL1RAPL2,IL1RAPL2,TEX13A,NRK,SERPINA7,MUM1L1,CXorf57 | -0.274985 | 230.381 | 720 | 424.689 |

| | | | | | | | | |
|---|---|---|---|---|---|---|---|---|
| 2 | 20 | 5585656 | 13698190 | C20orf196,CHGB,TRMT6,MCM8,MCM8,MCM8-AS1,CRLS1,LRRN4,FERMT1,BMP2,HAO1,TMX4,PLCB1,PLCB4,LAMP5-AS1,LAMP5,LAMP5,PAK5,SNAP25-AS1,ANKEF1,SNAP25,MKKS,SLX4IP,JAG1,JAG1,MIR6870,C20orf187,LINC00687,BTBD3,SPTLC3,ISM1,TASP1,ESF1 | -0.275625 | 227.771 | 416 | 245.979 |
| 2 | 14 | 21624649 | 23034147 | HNRNPC,RPGRIP1,SUPT16H,CHD8,CHD8,SNORD9,CHD8,SNORD8,RAB2B,TOX4,TOX4,METTL3,METTL3,SALL2,OR10G3,OR10G2,OR4E2,LOC105370401,LINC02332,DAD1 | -0.277611 | 213.484 | 446 | 265.232 |
| 2 | 9 | 38615678 | 47317179 | ANKRD18A,FAM201A,CNTNAP3,ZNF658B,ZNF658,CNTNAP3B,CNTNAP3P2 | -0.278301 | 105.492 | 82 | 38.6372 |
| 2 | 1 | 3739992 | 3769037 | CEP104 | -0.279858 | 266.267 | 21 | 12.3052 |
| 2 | 17 | 3924470 | 4268995 | ZZEF1,CYB5D2,ANKFY1,UBE2G1 | -0.283517 | 216.555 | 96 | 58.7992 |
| 2 | 1 | 1.62E+08 | 163295325 | UHMK1,UAP1,DDR2,HSD17B7,CCDC190,RGS4,RGS5,RGS5,LOC101928404 | -0.283747 | 259.066 | 78 | 45.6003 |
| 2 | 3 | 48788630 | 49028055 | PRKAR2A,SLC25A20,ARIH2OS,ARIH2OS,ARIH2,ARIH2,P4HTM | -0.284724 | 225.212 | 56 | 31.9094 |
| 2 | 2 | 37450339 | 39222525 | CEBPZ,CEBPZ,NDUFAF7,NDUFAF7,NDUFAF7,PRKD3,PRKD3,QPCT,CDC42EP3,RMDN2,RMDN2,RMDN2-AS1,CYP1B1,ATL2,LOC101929596,HNRNPLL,GALM,SRSF7,GEMIN6,DHX57,MORN2,ARHGEF33,ARHGEF33,LOC375196,SOS1 | -0.2851 | 249.095 | 220 | 133.28 |
| 2 | 3 | 1.93E+08 | 197961930 | HES1,CPN2,LRRC15,GP5,ATP13A3,TMEM44-AS1,TMEM44,TMEM44,LSG1,FAM43A,XXYLT1,XXYLT1,MIR3137,ACAP2,PPP1R2,APOD,LOC105374297,SDHAP2,SDHAP2,MIR570HG,MIR570HG,MIR570,MUC20,MUC4,TNK2,TNK2-AS1,SDHAP1,TFRC,ZDHHC19,SLC51A,PCYT1A,TCTEX1D2,TCTEX1D2,TM4SF19-TCTEX1D2,TM4SF19-TCTEX1D2,TM4SF19-AS1,TM4SF19,TM4SF19-TCTEX1D2,TM4SF19,UBXN7,UBXN7,UBXN7-AS1,RNF168,SMCO1,WDR53,FBXO45,NRROS,CEP19,PIGX,PAK2,SENP5,NCBP2,NCBP2,NCBP2-AS1,NCBP2-AS2,PIGZ,MELTF,MELTF-AS1,MELTF,DLG1,DLG1,MIR4797,BDH1,RUBCN,MIR922,RUBCN,RUBCN,FYTTD1,FYTTD1,LRCH3,IQCG,IQCG,RPL35A,LMLN,LMLN,LMLN-AS1 | -0.287445 | 242.464 | 643 | 375.669 |
| 2 | 10 | 94716040 | 95421901 | EXOC6,CYP26C1,CYP26A1,MYOF,CEP55,FFAR4,RBP4,PDE6C | -0.28766 | 243.165 | 135 | 82.94 |
| 2 | 5 | 34869718 | 45907753 | TTC23L,RAD1,BRIX1,DNAJC21,AGXT2,PRLR,SPEF2,IL7R,CAPSL,UGT3A1,UGT3A2,LMBRD2,LMBRD2,MIR580,SKP2,NADK2,RANBP3L,SLC1A3,NIPBL,C5orf42,NUP155,WDR70,GDNF,EGFLAM,LIFR,LIFR,LIFR-AS1,MIR3650,OSMR-AS1,OSMR,RICTOR,FYB1,C9,DAB2,PTGER4,TTC33,PRKAA1,RPL37,SNORD72,RPL37,CARD6,C7,MROH2B,C6,PLCXD3,OXCT1,OXCT1,OXCT1-AS1,C5orf51,FBXO4,GHR,CCDC152,CCDC152,SELENOP,SELENOP,LOC648987,ANXA2R,LOC648987,LOC100132356,ZNF131,NIM1K,HMGCS1,CCL28,TMEM267,C5orf34,PAIP1,NNT,FGF10,MRPS30,HCN1 | -0.295153 | 253.175 | 1014 | 598.95 |
| 2 | 17 | 61432120 | 61495826 | TANC2 | -0.295961 | 217.468 | 17 | 11.0804 |

| | | | | | | | | |
|---|---|---|---|---|---|---|---|---|
| 2 | 21 | 30445738 | 34045787 | CCT8,MAP3K7CL,BACH1,GRIK1,GRIK1,GRIK1-AS2,CLDN17,CLDN8,KRTAP24-1,KRTAP25-1,KRTAP26-1,KRTAP27-1,KRTAP23-1,KRTAP13-2,MIR4327,KRTAP13-1,KRTAP13-3,KRTAP13-4,KRTAP15-1,KRTAP19-1,KRTAP19-2,KRTAP19-3,KRTAP19-4,KRTAP19-5,KRTAP19-6,KRTAP19-7,KRTAP22-2,KRTAP6-3,KRTAP6-2,KRTAP22-1,KRTAP6-1,KRTAP20-1,KRTAP20-4,KRTAP20-2,KRTAP20-3,KRTAP21-3,KRTAP21-2,KRTAP21-1,KRTAP8-1,KRTAP7-1,KRTAP11-1,KRTAP19-8,TIAM1,LOC150051,SOD1,SCAF4,HUNK,MIS18A,MRAP,MRAP,URB1,URB1,URB1,SNORA80A,URB1-AS1,EVA1C,C21orf59-TCP10L,TCP10L,C21orf59-TCP10L,C21orf59-TCP10L,C21orf59,SYNJ1 | -0.297906 | 231.352 | 361 | 211.852 |
| 2 | 19 | 34960722 | 35448333 | WTIP,ZNF807,SCGB1B2P,SCGB2B2,ZNF302,ZNF181,ZNF599,ZNF30 | -0.299214 | 222.569 | 70 | 39.9469 |
| 2 | 15 | 66179983 | 73924960 | RAB11A,MEGF11,MEGF11,MIR4311,DIS3L,TIPIN,TIPIN,SCARNA14,MAP2K1,MAP2K1,SNAPC5,SNAPC5,RPL4,RPL4,SNORD18C,RPL4,SNORD18B,RPL4,SNORD16,RPL4,SNORD18A,ZWILCH,ZWILCH,LCTL,LCTL,LINC01169,SMAD6,LINC02206,LOC102723493,SMAD3,AAGAB,AAGAB,IQCH,IQCH,IQCH,IQCH-AS1,IQCH-AS1,C15orf61,C15orf61,MAP2K5,SKOR1,PIAS1,CALML4,CLN6,FEM1B,ITGA11,CORO2B,ANP32A,ANP32A,MIR4312,MIR548H4,SPESP1,NOX5,MIR548H4,NOX5,GLCE,PAQR5,PAQR5,LOC145694,LOC145694,KIF23,KIF23,RPLP1,DRAIC,TLE3,TLE3,MIR629,UACA,LARP6,LARP6,LRRC49,LRRC49,LRRC49,THAP10,CT62,THSD4,NR2E3,MYO9A,SENP8,GRAMD2A,PKM,PARP6,CELF6,HEXA,HEXA-AS1,TMEM202,TMEM202,TMEM202-AS1,TMEM202-AS1,ARIH1,MIR630,HIGD2B,BBS4,ADPGK,ADPGK,ADPGK-AS1,ADPGK-AS1,NEO1,HCN4,REC114,NPTN | -0.299218 | 236.142 | 946 | 554.328 |
| 2 | 3 | 1.49E+08 | 159975745 | TM4SF1,TM4SF4,WWTR1,COMMD2,ANKUB1,RNF13,PFN2,TSC22D2,SERP1,EIF2A,SELENOT,ERICH6,ERICH6,ERICH6-AS1,SIAH2,MINDY4B,CLRN1,CLRN1,CLRN1-AS1,MED12L,MED12L,GPR171,MED12L,P2RY14,MED12L,GPR87,MED12L,P2RY13,MED12L,P2RY12,IGSF10,MIR5186,MIR548H2,AADACL2,MIR548H2,AADACL2,AADACL2-AS1,MIR548H2,AADACL2-AS1,AADAC,AADACL2-AS1,AADAC,AADACL2-AS1,SUCNR1,MBNL1-AS1,MBNL1,MBNL1,MBNL1,TMEM14EP,P2RY1,RAP2B,LINC02006,C3orf79,ARHGEF26,DHX36,GPR149,MME,PLCH1,PLCH1,PLCH1-AS2,C3orf33,SLC33A1,GMPS,KCNAB1,SSR3,TIPARP,LEKR1,CCNL1,VEPH1,VEPH1,PTX3,PQLC2L,SHOX2,RSRC1,MLF1,GFM1,GFM1,LXN,RARRES1,MFSD1,IQCJ,IQCJ-SCHIP1,IQCJ-SCHIP1,SCHIP1,IL12A-AS1,IL12A,IL12A-AS1,IL12A-AS1,LINC01100,C3orf80 | -0.301091 | 254.18 | 816 | 471.759 |
| 2 | 14 | 24912592 | 26918192 | LOC101927045,CMA1,CTSG,GZMH,GZMB,STXBP6,NOVA1 | -0.302411 | 180.614 | 58 | 32.3085 |

| | | | | | | | | |
|---|---|---|---|---|---|---|---|---|
| 2 | 3 | 57902627 | 69071797 | SLMAP,FLNB,FLNB,FLNB-AS1,DNASE1L3,ABHD6,HTD2,RPP14,PXK,PDHB,KCTD6,ACOX2,FAM107A,FAM107A,FAM3D-AS1,FAM3D-AS1,FAM3D,FAM3D,C3orf67,C3orf67,C3orf67-AS1,FHIT,PTPRG,PTPRG,PTPRG-AS1,C3orf14,FEZF2,CADPS,SYNPR,SYNPR,SYNPR-AS1,SNTN,C3orf49,C3orf49,THOC7,THOC7,ATXN7,PSMD6-AS2,PSMD6,PSMD6,PRICKLE2-AS1,PRICKLE2,PRICKLE2,PRICKLE2,PRICKLE2-AS3,ADAMTS9,ADAMTS9,ADAMTS9-AS1,ADAMTS9,ADAMTS9-AS2,MAGI1,SLC25A26,LRIG1,KBTBD8,MIR4272,SUCLG2,FAM19A1,FAM19A4,EOGT | -0.305324 | 201.885 | 662 | 386.352 |
| 2 | 12 | 1.12E+08 | 113279868 | ATXN2,BRAP,ACAD10,ALDH2,ALDH2,MIR6761,MAPKAPK5-AS1,MAPKAPK5,MAPKAPK5,ADAM1A,TMEM116,ERP29,NAA25,NAA25,MIR3657,TRAFD1,HECTD4,HECTD4,MIR6861,RPL6,PTPN11,RPH3A,MIR1302-1,RPH3A | -0.309097 | 231.493 | 277 | 166.837 |
| 2 | 10 | 1.16E+08 | 116853814 | CCDC186,CCDC186,MIR2110,TDRD1,VWA2,AFAP1L2,ABLIM1,ABLIM1,LOC101927692,FAM160B1,TRUB1,ATRNL1 | -0.312849 | 226.247 | 145 | 88.478 |
| 2 | X | 73045726 | 76777870 | TSIX,XIST,XIST,FTX,FTX,MIR421,MIR374B,MIR374C,FTX,MIR545,MIR374A,ZCCHC13,SLC16A2,RLIM,NEXMIF,ABCB7,UPRT,ZDHHC15,TTC3P1,MAGEE2,PBDC1,MAGEE1,MIR325HG,MIR384,MIR325HG,MIR325,FGF16,ATRX | -0.313727 | 209.332 | 223 | 135.915 |
| 2 | 2 | 1.28E+08 | 128938680 | WDR33,POLR2D,AMMECR1L,SAP130,UGGT1 | -0.317768 | 224.795 | 100 | 60.5828 |
| 2 | X | 1.18E+08 | 119660056 | IL13RA1,ZCCHC12,LONRF3,KIAA1210,PGRMC1,SLC25A43,SLC25A5-AS1,SLC25A5,SLC25A5,CXorf56,UBE2A,NKRF,SEPT6,SEPT6,MIR766,SOWAHD,RPL39,RPL39,SNORA69,UPF3B,RNF113A,NDUFA1,AKAP14,NKAP,RHOXF1-AS1,RHOXF2B,RHOXF2,RHOXF1-AS1,RHOXF1,RHOXF2,RHOXF2B,ZBTB33,TMEM255A,ATP1B4,LAMP2 | -0.31984 | 229.924 | 253 | 149.321 |
| 2 | 4 | 1.77E+08 | 184562095 | SPATA4,ASB5,SPCS3,VEGFC,NEIL3,AGA,LINC01098,MIR1305,TENM3,DCTD,WWC2-AS2,WWC2,WWC2,WWC2-AS1,WWC2,CLDN22,CLDN24,CDKN2AIP,ING2 | -0.32125 | 189.76 | 221 | 129.451 |
| 2 | 3 | 49999992 | 50154746 | RBM6,RBM5,RBM5,RBM5-AS1,RBM5,SEMA3F-AS1 | -0.323657 | 195.204 | 56 | 33.8315 |
| 2 | 2 | 32429661 | 37316893 | SLC30A6,NLRC4,YIPF4,BIRC6,BIRC6,MIR558,TTC27,TTC27,MIR4765,LTBP1,MIR4430,RASGRP3,RASGRP3,LOC105374454,FAM98A,LINC01317,MYADML,CRIM1,FEZ2,VIT,STRN,HEATR5B,GPATCH11 | -0.324144 | 236.923 | 401 | 234.642 |
| 2 | 7 | 94257456 | 97599196 | SGCE,PEG10,PPP1R9A,PON1,PON3,PON2,ASB4,PDK4,DYNC1I1,SLC25A13,SLC25A13,MIR591,SEM1,DLX6-AS1,DLX6,DLX5,SDHAF3,TAC1,ASNS,CZ1P-ASNS,CZ1P-ASNS,MIR5692C2,CZ1P-ASNS,CCZ1P-OR7E38P | -0.325671 | 239.37 | 216 | 124.643 |
| 2 | 9 | 80030815 | 86354720 | VPS13A,GNA14,GNAQ,CEP78,PSAT1,TLE4,TLE1,SPATA31D5P,SPATA31D4,SPATA31D3,SPATA31D1,RASEF,FRMD3,IDNK,UBQLN1,LOC105376114,GKAP1 | -0.325975 | 239.222 | 291 | 157.843 |
| 2 | 10 | 70706177 | 70884084 | DDX50,DDX21,KIF1BP,SRGN,VPS26A | -0.328634 | 243.315 | 36 | 21.1788 |

| | | | | | | | | |
|---|---|---|---|---|---|---|---|---|
| 2 | 17 | 64066645 | 65211391 | CEP112,APOH,PRKCA,PRKCA,MIR634,CACNG5,CACNG4,CACNG1,HELZ | -0.331429 | 236.628 | 107 | 62.564 |
| 2 | 3 | 1.85E+08 | 193201176 | VPS8,C3orf70,EHHADH-AS1,EHHADH,EHHADH,MIR5588,MAP3K13,TMEM41A,LIPH,SENP2,IGF2BP2,IGF2BP2,IGF2BP2-AS1,TRA2B,ETV5,DGKG,CRYGS,TBCCD1,DNAJB11,AHSG,FETUB,HRG,KNG1,EIF4A2,EIF4A2,SNORD2,EIF4A2,MIR1248,SNORA81,EIF4A2,SNORA63,EIF4A2,SNORA4,RFC4,ADIPOQ,ADIPOQ-AS1,ST6GAL1,RPL39L,LOC101929106,RTP1,MASP1,MASP1,LOC101929130,RTP4,SST,RTP2,RTP2,LOC100131635,LOC100131635,LOC100131635,BCL6,BCL6,LPP,FLJ42393,LPP,LPP,MIR28,TPRG1,TPRG1,TPRG1-AS2,TP63,TP63,MIR944,P3H2,CLDN1,CLDN16,TMEM207,IL1RAP,GMNC,OSTN,OSTN,OSTN-AS1,UTS2B,UTS2B,CCDC50,CCDC50,LINCR-0002,PYDC2,FGF12,FGF12,FGF12-AS1,MB21D2,HRASLS,ATP13A5,ATP13A5,ATP13A5-AS1,ATP13A4 | -0.333056 | 200.993 | 626 | 370.072 |
| 2 | 19 | 44341259 | 44988746 | ZNF283,ZNF404,ZNF45,ZNF221,ZNF155,ZNF155,LOC101928063,LOC101928063,ZNF230,ZNF222,ZNF223,ZNF284,ZNF224,ZNF224,LOC100379224,ZNF225,ZNF234,ZNF226,ZNF227,ZNF233,ZNF235,ZNF112,ZNF285,ZNF229,ZNF180 | -0.333709 | 238.319 | 236 | 141.572 |
| 2 | 11 | 70118495 | 70229240 | PPFIA1,PPFIA1,MIR548K | -0.336911 | 235.444 | 44 | 26.1032 |
| 2 | 8 | 27691217 | 30601907 | SCARA5,SCARA5,MIR4287,NUGGC,ELP3,PNOC,ZNF395,FBXO16,FZD3,FZD3,MIR4288,EXTL3,INTS9,HMBOX1,KIF13B,DUSP4,MIR3148,SARAF,LEPROTL1,LEPROTL1,MBOAT4,MBOAT4,DCTN6,RBPMS-AS1,RBPMS,RBPMS,GTF2E2,GTF2E2,SMIM18,GSR,UBXN8 | -0.338865 | 209.531 | 314 | 182.249 |
| 2 | 21 | 34117839 | 38459151 | PAXBP1,C21orf62-AS1,C21orf62-AS1,C21orf62,C21orf62,OLIG2,OLIG1,LINC01548,IFNAR2,IL10RB-AS1,IL10RB,IFNAR1,IFNGR2,IFNGR2,TMEM50B,TMEM50B,DNAJC28,GART,SON,SON,MIR6501,DONSON,CRYZL1,ITSN1,ATP5PO,SLC5A3,MRPS6,MRPS6,KCNE2,SMIM11A,SMIM11B,C21orf140,KCNE1,KCNE1B,RCAN1,CLIC6,RUNX1,RUNX1,RUNX1-IT1,MIR802,SETD4,LOC100133286,CBR1,CBR3-AS1,CBR3,DOPEY2,MORC3,CHAF1B,CLDN14,SIM2,HLCS,RIPPLY3,PIGP | -0.342481 | 228.458 | 520 | 308.099 |
| 2 | 19 | 19779736 | 19904847 | ZNF101,ZNF14,LINC00663 | -0.34428 | 168.586 | 25 | 13.3359 |
| 2 | 9 | 1.07E+08 | 114449155 | SMC2,OR13F1,OR13C4,OR13C3,OR13C8,OR13C5,OR13C2,OR13C9,OR13D1,NIPSNAP3A,NIPSNAP3B,ABCA1,SLC44A1,FSD1L,FKTN,TAL2,TMEM38B,ZNF462,ZNF462,LOC340512,RAD23B,KLF4,ACTL7B,ACTL7A,ELP1,FAM206A,CTNNAL1,TMEM245,TMEM245,MIR32,FRRS1L,EPB41L4B,PTPN3,MIR3927,PALM2,PALM2,PALM2-AKAP2,PALM2-AKAP2,PALM2-AKAP2,AKAP2,C9orf152,TXN,TXNDC8,SVEP1,MUSK,LPAR1,OR2K2,KIAA0368,ZNF483,ZNF483,PTGR1,PTGR1,LRRC37A5P,DNAJC25,DNAJC25-GNG10,DNAJC25-GNG10,GNG10,C9orf84 | -0.344498 | 223.882 | 728 | 433.137 |

| | | | | | | | | |
|---|---|---|---|---|---|---|---|---|
| 2 | 6 | 1.23E+08 | 123539924 | HSF2,SERINC1,PKIB,FABP7,SMPDL3A,CLVS2,TRDN | -0.346426 | 258.916 | 45 | 26.0214 |
| 2 | 5 | 70862474 | 72144239 | MCCC2,CARTPT,MAP1B,MAP1B,MIR4803,MRPS27,PTCD2,ZNF366,TNPO1 | -0.346663 | 210.76 | 107 | 66.4342 |
| 2 | 6 | 1.29E+08 | 136551866 | PTPRK,LAMA2,ARHGAP18,TMEM244,L3MBTL3,SAMD3,TMEM200A,SMLR1,EPB41L2,AKAP7,ARG1,ARG1,MED23,MED23,ENPP3,ENPP3,OR2A4,MIR548H5,ENPP1,CTGF,MIR548AJ1,MOXD1,STX7,TAAR9,TAAR8,TAAR6,TAAR5,TAAR3P,TAAR2,TAAR1,VNN1,VNN3,VNN2,SLC18B1,RPS12,RPS12,SNORD101,RPS12,SNORD100,RPS12,SNORA33,EYA4,EYA4,TARID,TCF21,TBPL1,SLC2A12,SGK1,ALDH8A1,HBS1L,HBS1L,MIR3662,MYB,MIR548A2,AHI1,LINC00271,PDE7B | -0.34827 | 244.286 | 654 | 386.277 |
| 2 | 11 | 62782958 | 63532661 | SLC22A24,SLC22A25,SLC22A10,SLC22A9,HRASLS5,LGALS12,RARRES3,HRASLS2,PLA2G16,ATL3,RTN3,C11orf95 | -0.348458 | 210.293 | 149 | 86.5322 |
| 2 | 11 | 1.02E+08 | 102933229 | TMEM123,LOC102723838,MMP7,MMP20,MMP27,MMP8,MMP10,WTAPP1,MMP1,WTAPP1,MMP3,MMP3,MMP12,MMP13,DCUN1D5 | -0.351584 | 204.244 | 122 | 72.8772 |
| 2 | 19 | 43092848 | 43857307 | LIPE-AS1,CEACAM8,PSG3,PSG8,PSG10P,PSG1,PSG6,PSG7,PSG11,PSG2,PSG5,PSG4,PSG9 | -0.356015 | 272.936 | 60 | 27.017 |
| 2 | 2 | 2.21E+08 | 232161033 | MIR4268,EPHA4,PAX3,PAX3,CCDC140,CCDC140,SGPP2,FARSB,MOGAT1,ACSL3,KCNE4,SCG2,AP1S3,WDFY1,MRPL44,SERPINE2,FAM124B,CUL3,DOCK10,DOCK10,MIR4439,NYAP2,MIR5702,IRS1,RHBDD1,COL4A4,COL4A3,COL4A3,LOC654841,MFF,TM4SF20,MIR5703,AGFG1,AGFG1,C2orf83,SLC19A3,CCL20,DAW1,SPHKAP,PID1,DNER,TRIP12,TRIP12,FBXO36,FBXO36,SLC16A14,SP110,SP140,SP140L,SP100,CAB39,ITM2C,GPR55,SPATA3,C2orf72,PSMD1,PSMD1,HTR2B,ARMC9 | -0.356327 | 209.489 | 897 | 521.625 |
| 2 | 8 | 31014903 | 32611894 | WRN,NRG1 | -0.356664 | 178.907 | 35 | 20.6375 |
| 2 | 1 | 2.1E+08 | 223168437 | DIEXF,SYT14,SERTAD4-AS1,SERTAD4-AS1,SERTAD4,SERTAD4,HHAT,KCNH1,RCOR3,TRAF5,LINC00467,RD3,SLC30A1,NEK2,LPGAT1,INTS7,DTL,DTL,MIR3122,PPP2R5A,PPP2R5A,SNORA16B,TMEM206,NENF,ATF3,FAM71A,BATF3,NSL1,TATDN3,SPATA45,FLVCR1,VASH2,ANGEL2,RPS6KC1,PROX1,SMYD2,PTPN14,CENPF,KCNK2,KCTD3,USH2A,USH2A,LOC102723833,ESRRG,GPATCH2,SPATA17,SPATA17,SPATA17-AS1,RRP15,TGFB2,TGFB2,MIR548F3,MIR548F3,C1orf143,LYPLAL1,LOC102723886,ZC3H11B,RNU5F-1,SLC30A10,RNU5F-1,EPRS,RNU5F-1,BPNT1,RNU5F-1,IARS2,RNU5F-1,IARS2,MIR215,RNU5F-1,IARS2,MIR194-1,IARS2,RAB3GAP2,RAB3GAP2,MIR664A,SNORA36B,MARK1,C1orf115,MARC2,MARC1,HLX-AS1,HLX,HLX,C1orf140,DUSP10,HHIPL2,TAF1A,MIA3,AIDA,BROX,FAM177B,DISP1 | -0.357306 | 234.085 | 1025 | 603.845 |

| | | | | | | | | |
|---|---|---|---|---|---|---|---|---|
| 2 | 11 | 58276090 | 60511165 | LPXN,ZFP91,ZFP91-CNTF,ZFP91-CNTF,CNTF,GLYAT,GLYATL2,GLYATL1,GLYATL1,LOC283194,FAM111B,FAM111A,DTX4,MPEG1,OR5AN1,OR5A2,OR5A1,OR4D6,OR4D10,OR4D11,OR4D9,OSBP,OSBP,MIR3162,PATL1,OR10V1,OR10V2P,STX3,MRPL16,GIF,TCN1,OOSP1,OOSP2,MS4A3,MS4A2,MS4A6A,MS4A4E,MS4A4A,MS4A6E,MS4A7,MS4A14,MS4A5,MS4A1,MS4A12,MS4A13,MS4A8,MS4A18 | -0.357315 | 229.167 | 364 | 212.843 |
| 2 | 12 | 60500 | 34431528 | IQSEC3,IQSEC3,LOC574538,SLC6A12,SLC6A12,LOC101929384,SLC6A13,KDM5A,CCDC77,B4GALNT3,NINJ2,NINJ2,LOC100049716,WNK1,RAD52,ERC1,FBXL14,WNT5B,MIR3649,ADIPOR2,CACNA2D4,CACNA2D4,LRTM2,LINC00940,DCP1B,CACNA1C,CACNA1C,CACNA1C-AS2,CACNA1C,CACNA1C-AS1,ITFG2-AS1,ITFG2-AS1,FKBP4,ITFG2-AS1,ITFG2,ITFG2,ITFG2,NRIP2,ITFG2,FOXM1,FOXM1,RHNO1,TULP3,TEAD4,TSPAN9,LOC100128253,PRMT8,PRMT8,THCAT155,CRACR2A,PARP11,CCND2-AS1,CCND2,CCND2,TIGAR,FGF23,FGF6,C12orf4,RAD51AP1,DYRK4,AKAP3,AKAP3,NDUFA9,NDUFA9,GALNT8,KCNA6,KCNA1,KCNA5,NTF3,ANO2,VWF,CD9,PLEKHG6,TNFRSF1A,SCNN1A,SCNN1A,LTBR,LTBR,CD27-AS1,CD27-AS1,CD27,TAPBPL,TAPBPL,VAMP1,VAMP1,MRPL51,NCAPD2,NCAPD2,SCARNA10,GAPDH,IFFO1,NOP2,CHD4,CHD4,SCARNA11,LPAR5,ACRBP,ING4,ZNF384,PIANP,COPS7A,MLF2,PTMS,LAG3,CD4,GPR162,P3H3,GNB3,GNB3,CDCA3,CDCA3,USP5,TPI1,SPSB2,LRRC23,ENO2,ATN1,C12orf57,PTPN6,MIR200CHG,MIR200C,MIR200CHG,MIR141,PHB2,PHB2,SCARNA12,EMG1,EMG1,LPCAT3,LPCAT3,C1S,C1R,C1RL,C1RL,C1RL-AS1,C1RL-AS1,RBP5,CLSTN3,PEX5,ACSM4,CD163L1,CD163,APOBEC1,GDF3,DPPA3,CLEC4C,NANOGNB,NANOG,SLC2A14,SLC2A3,FOXJ2,C3AR1,NECAP1,CLEC4A,CLEC4A,POU5F1P3,FAM90A1,LINC00937,CLEC6A,CLEC4D,CLEC4E,AICDA,MFAP5,RIMKLB,A2ML1,PHC1,PHC1,M6PR,M6PR,KLRG1,LINC00612,A2M-AS1,A2M,A2M,PZP,LOC642846,LINC02367,LOC728715,LOC728715,DDX12P,KLRB1,CLEC2D,CLECL1,CD69,KLRF1,CLEC2B,KLRF2,CLEC2A,CLEC12A-AS1,CLEC12A,CLEC12A,CLEC1B,CLEC12B,CLEC12B,LOC | -0.359521 | 229.527 | 4152 | 2416.2 |
| 2 | 10 | 90537682 | 91469836 | LIPN,LIPM,LIPM,ANKRD22,ANKRD22,STAMBPL1,ACTA2-AS1,ACTA2,ACTA2,ACTA2,FAS,FAS,CH25H,LIPA,IFIT2,IFIT3,IFIT1B,IFIT1,IFIT5,SLC16A12,SLC16A12,SLC16A12-AS1,PANK1,PANK1,MIR107,KIF20B | -0.362341 | 222.791 | 144 | 89.1695 |
| 2 | X | 47705608 | 47920001 | ZNF81,ZNF182,SPACA5,SPACA5B,ZNF630-AS1,ZNF630-AS1,ZNF630 | -0.364839 | 185.217 | 43 | 24.3174 |
| 2 | 14 | 50319306 | 50886884 | NEMF,ARF6,LINC01588,LINC01588,LINC01599,LINC01599,VCPKMT,SOS2,L2HGDH,L2HGDH,MIR4504,ATP5S,CDKL1,MAP4K5 | -0.365735 | 241.958 | 116 | 67.2746 |
| 2 | 10 | 1.06E+08 | 112327690 | CFAP43,CFAP43,MIR609,GSTO1,MIR4482,GSTO2,ITPRIP,CFAP58,SORCS3,SORCS1,XPNPEP1,ADD3-AS1,ADD3,MXI1,SMNDC1,DUSP5,SMC3 | -0.377409 | 194.493 | 263 | 154.31 |

| | | | | | | | | |
|---|---|---|---|---|---|---|---|---|
| 2 | 3 | 1.73E+08 | 178916750 | ECT2,SPATA16,NLGN1,NAALADL2,NAALADL2,NAALADL2-AS3,NAALADL2,NAALADL2-AS2,NAALADL2,MIR4789,TBL1XR1,KCNMB2-AS1,KCNMB2,ZMAT3,PIK3CA | -0.377817 | 162.541 | 143 | 79.2525 |
| 2 | 10 | 18874871 | 27284161 | NSUN6,ARL5B,MALRD1,PLXDC2,MIR4675,NEBL,NEBL,C10orf113,CASC10,CASC10,MIR1915,SKIDA1,MLLT10,DNAJC1,EBLN1,LOC100130992,COMMD3,COMMD3-BMI1,COMMD3-BMI1,BMI1,SPAG6,PIP4K2A,ARMC3,MSRB2,PTF1A,C10orf67,OTUD1,KIAA1217,KIAA1217,MIR603,ARHGAP21,PRTFDC1,ENKUR,ENKUR,THNSL1,GPR158-AS1,GPR158,GPR158,MYO3A,GAD2,APBB1IP,PDSS1,ABI1 | -0.379933 | 224.031 | 627 | 356.907 |
| 2 | 6 | 17111022 | 25819461 | STMND1,RBM24,CAP2,CAP2,LOC101928491,FAM8A1,NUP153,NUP153,LOC105374952,KIF13A,NHLRC1,TPMT,KDM1B,DEK,RNF144B,MIR548A1HG,MIR548A1,ID4,MBOAT1,E2F3,CDKAL1,SOX4,CASC15,NBAT1,PRL,HDGFL1,NRSN1,DCDC2,DCDC2,KAAG1,MRS2,GPLD1,ALDH5A1,KIAA0319,TDP2,ACOT13,C6orf62,GMNN,C6orf229,RIPOR2,CMAHP,CARMIL1,SCGN,HIST1H2AA,HIST1H2BA,SLC17A4,SLC17A1 | -0.383316 | 207.547 | 579 | 339.433 |
| 2 | 2 | 1.98E+08 | 201397138 | PGAP1,ANKRD44,SF3B1,COQ10B,HSPD1,HSPE1,HSPE1-MOB4,HSPE1-MOB4,MOB4,RFTN2,MARS2,BOLL,PLCL1,SATB2,FTCDNL1,C2orf69,TYW5,TYW5,MAIP1,MAIP1,SPATS2L,KCTD18 | -0.383336 | 228.999 | 287 | 161.638 |
| 2 | 2 | 2.04E+08 | 214149545 | CYP20A1,ABI2,RAPH1,CD28,CTLA4,ICOS,PARD3B,NRP2,INO80D,NDUFS1,EEF1B2,EEF1B2,SNORD51,EEF1B2,SNORA41,GPR1,ZDBF2,ADAM23,FAM237A,DYTN,MDH1B,FASTKD2,FASTKD2,MIR3130-1,MIR3130-2,CPO,KLF7,KLF7,MIR2355,LINC01802,MIR1302-4,CREB1,CREB1,METTL21A,METTL21A,CCNYL1,CCNYL1,MIR4775,FZD5,PLEKHM3,LOC100507443,CRYGD,LOC100507443,CRYGC,LOC100507443,CRYGB,CRYGA,C2orf80,IDH1,PIKFYVE,PTH2R,LOC101927960,MAP2,UNC80,RPE,KANSL1L,KANSL1L,LOC101928020,ACADL,MYL1,LANCL1-AS1,LANCL1,LANCL1,CPS1,ERBB4,ERBB4,MIR548F2,MIR4776-1,MIR4776-2,IKZF2,LOC100130451,SPAG16 | -0.383568 | 205.613 | 836 | 494.371 |
| 2 | 1 | 48713755 | 52195725 | SPATA6,AGBL4,AGBL4,BEND5,ELAVL4,DMRTA2,FAF1,CDKN2C,MIR4421,C1orf185,RNF11,TTC39A,TTC39A,TTC39A-AS1,EPS15,OSBPL9 | -0.385027 | 200.829 | 216 | 115.854 |
| 2 | 1 | 2.07E+08 | 207963676 | C4BPB,C4BPA,CD55,CR2,CR1,CR1L,CD46 | -0.386844 | 266.065 | 131 | 71.1646 |
| 2 | 10 | 1.18E+08 | 123727387 | PNLIPRP3,PNLIP,PNLIPRP1,PNLIPRP2,C10orf82,HSPA12A,HSPA12A,ENO4,ENO4,SHTN1,VAX1,MIR3663HG,MIR3663,KCNK18,SLC18A2,PDZD8,EMX2OS,EMX2,EMX2,RAB11FIP2,FAM204A,PRLHR,CACUL1,NANOS1,EIF3A,EIF3A,SNORA19,FAM45A,FAM45BP,SFXN4,PRDX3,GRK5,GRK5,MIR4681,RGS10,TIAL1,BAG3,INPP5F,MCMBP,SEC23IP,MIR4682,PLPP4,LINC01561,WDR11,FGFR2,ATE1,ATE1,ATE1-AS1,NSMCE4A | -0.387134 | 229.671 | 521 | 311.209 |

| | | | | | | | | |
|---|---|---|---|---|---|---|---|---|
| 2 | 9 | 72967111 | 79814290 | SMC5,KLF9,TRPM3,TRPM3,MIR204,TMEM2,ABHD17B,C9orf85,C9orf57,GDA,ZFAND5,TMC1,ALDH1A1,ANXA1,RORB-AS1,RORB,RORB,TRPM6,C9orf40,C9orf41-AS1,CARNMT1,CARNMT1,NMRK1,OSTF1,PCSK5,RFK,GCNT1,PRUNE2,FOXB2,VPS13A-AS1,VPS13A | -0.388361 | 215.188 | 468 | 275.34 |
| 2 | 5 | 75009292 | 76326798 | SV2C,IQGAP2,IQGAP2,F2RL2,F2R,F2RL1,S100Z,CRHBP,AGGF1 | -0.388553 | 205.963 | 107 | 64.3887 |
| 2 | 13 | 88636973 | 95227094 | MIR17HG,MIR17,MIR18A,MIR17HG,MIR19A,MIR20A,MIR17HG,MIR19B1,MIR92A1,GPC5,GPC5,MIR548AS,GPC6,GPC6,GPC6-AS2,DCT,TGDS | -0.389747 | 139.4 | 88 | 50.3844 |
| 2 | 11 | 55035775 | 56258039 | TRIM48,OR4A16,OR4A15,OR4C15,OR4C16,OR4C11,OR4P4,OR4S2,OR4C6,OR5D13,OR5D14,OR5L1,OR5D18,OR5L2,OR5D16,TRIM51,OR5W2,OR5I1,OR10AG1,OR7E5P,OR5F1,OR5AS1,OR8I2,OR8H2,OR8H3,OR8J3,OR8K5,OR5J2,OR5T2,OR5T3,OR5T1,OR8H1,OR8K3,OR8K1,OR8J1,OR8U8,OR8U1,OR5R1,OR5M9,OR5M3,OR5M8 | -0.391481 | 272.48 | 190 | 105.953 |
| 2 | 17 | 10223413 | 11924355 | MYH13,MYHAS,MYH8,MYHAS,MYH4,MYHAS,MYH1,MYHAS,MYH2,MYH3,SCO1,ADPRM,TMEM220,TMEM220,TMEM220-AS1,PIRT,SHISA6,DNAH9,ZNF18,MAP2K4 | -0.393633 | 228.13 | 382 | 227.295 |
| 2 | 1 | 2.35E+08 | 244572350 | COA6,TARBP1,IRF2BP2,TOMM20,TOMM20,SNORA14B,RBM34,ARID4B,ARID4B,MIR4753,GGPS1,TBCE,TBCE,B3GALNT2,B3GALNT2,MIR5096,GNG4,MIR5096,LYST,LYST,LYST,MIR1537,NID1,GPR137B,ERO1B,EDARADD,LGALS8,LGALS8-AS1,LGALS8,LGALS8,HEATR1,HEATR1,ACTN2,MTR,RYR2,RYR2,MIR4428,LOC100130331,ZP4,CHRM3,FMN2,MIR1273E,MIR1273E,GREM2,RGS7,RGS7,MIR3123,FH,KMO,KMO,OPN3,OPN3,OPN3,CHML,WDR64,EXO1,MAP1LC3C,PLD5,CEP170,SDCCAG8,SDCCAG8,MIR4677,SDCCAG8,AKT3,AKT3,ZBTB18,C1orf100 | -0.394221 | 230.81 | 892 | 524.865 |
| 2 | 5 | 1.51E+08 | 171384745 | G3BP1,GLRA1,NMUR2,GRIA1,FAM114A2,MFAP3,GALNT10,GALNT10,MIR1294,GALNT10,SAP30L-AS1,SAP30L,HAND1,MIR3141,MIR1303,LARP1,FAXDC2,FAXDC2,MIR378H,CNOT8,GEMIN5,MRPL22,KIF4B,SGCD,PPP1R2P3,TIMD4,HAVCR1,HAVCR2,MED7,FAM71B,ITK,CYFIP2,CYFIP2,FNDC9,CYFIP2,LOC102724404,NIPAL4,ADAM19,SOX30,C5orf52,THG1L,LSM11,CLINT1,EBF1,RNF145,UBLCP1,IL12B,ADRA1B,TTC1,PWWP2A,FABP6,CCNJL,C1QTNF2,ZBED8,SLU7,PTTG1,MIR3142HG,MIR3142HG,MIR146A,ATP10B,GABRB2,GABRA6,GABRA1,GABRG2,CCNG1,NUDCD2,HMMR,HMMR,HMMR-AS1,MAT2B,TENM2,WWC1,RARS,FBLL1,PANK3,PANK3,MIR103A1,MIR103B1,SLIT3,SLIT3,LOC101927969,SLIT3,MIR218-2,SLIT3,MIR585,SPDL1,DOCK2,DOCK2,FAM196B,DOCK2,MIR378E,FOXI1,C5orf58,C5orf58,LCP2,LCP2,KCNIP1,KCNIP1,KCNMB1,GABRP,RANBP17,TLX3,MIR3912,NPM1,FGF18,SMIM23,FBXW11 | -0.395243 | 197.479 | 1234 | 722.93 |
| 2 | 6 | 60500 | 610180 | LOC285766,DUSP22,IRF4,EXOC2 | -0.396242 | 272.372 | 62 | 32.127 |
| 2 | 1 | 1.62E+08 | 161927711 | DUSP12,ATF6 | -0.402353 | 219.898 | 24 | 13.0303 |

| | | | | | | | | |
|---|---|---|---|---|---|---|---|---|
| 2 | 18 | 31224776 | 35539848 | ASXL3,NOL4,DTNA,MAPRE2,ZNF397,ZNF397,ZSCAN30,ZSCAN30,ZNF271P,ZNF24,ZNF396,INO80C,MIR3975,GALNT1,MIR187,MIR3929,C18orf21,RPRD1A,SLC39A6,ELP2,MOCOS,FHOD3,TPGS2,TPGS2,KIAA1328,KIAA1328,CELF4,CELF4,LOC105372068,MIR4318 | -0.404827 | 215.828 | 361 | 211.36 |
| 2 | X | 1.06E+08 | 114238029 | TBC1D8B,RIPPLY1,CLDN2,CLDN2,MORC4,RBM41,NUP62CL,PIH1D3,FRMPD3-AS1,FRMPD3,FRMPD3,PRPS1,TSC22D3,NCBP2L,MID2,MID2,LOC101928335,TEX13B,VSIG1,PSMD10,ATG4A,COL4A6,COL4A5,IRS4,GUCY2F,NXT2,KCNE5,ACSL4,TMEM164,TMEM164,MIR652,TMEM164,MIR3978,AMMECR1,AMMECR1,SNORD96B,RTL9,TDGF1P3,CHRDL1,PAK3,CAPN6,DCX,ALG13,TRPC5,TRPC5,TRPC5OS,RTL4,LHFPL1,AMOT,AMOT,MIR4329,HTR2C,SNORA35,HTR2C,MIR764,HTR2C,MIR1912,HTR2C,MIR1264,HTR2C,MIR1298,HTR2C,HTR2C,MIR1911,HTR2C,MIR448 | -0.406158 | 190.733 | 617 | 356.598 |
| 2 | 21 | 40652061 | 42079860 | BRWD1,BRWD1-AS2,HMGN1,WRB,WRB-SH3BGR,WRB-SH3BGR,WRB,LCA5L,WRB-SH3BGR,SH3BGR,B3GALT5-AS1,B3GALT5,IGSF5,PCP4,DSCAM,DSCAM,MIR4760 | -0.407223 | 197.034 | 128 | 72.1709 |
| 2 | 2 | 1.71E+08 | 171853213 | UBR3,MYO3B,LOC101926913,LINC01124,LOC101926913,SP5,LOC101926913,ERICH2,ERICH2,GAD1,GORASP2,TLK1 | -0.40911 | 224.365 | 152 | 87.6234 |
| 2 | 17 | 62462533 | 64001288 | MILR1,POLG2,DDX5,DDX5,MIR3064,DDX5,MIR5047,DDX5,CEP95,CEP95,SMURF2,ARHGAP27P1-BPTFP1-KPNA2P3,ARHGAP27P1-BPTFP1-KPNA2P3,ARHGAP27P1,ARHGAP27P1-BPTFP1-KPNA2P3,ARHGAP27P1,MIR6080,PLEKHM1P1,PLEKHM1P1,MIR4315-1,MIR4315-2,LRRC37A3,AMZ2P1,GNA13,RGS9,AXIN2,CEP112 | -0.411349 | 235.533 | 199 | 113.328 |
| 2 | 3 | 69126910 | 90311186 | UBA3,ARL6IP5,LMOD3,FRMD4B,MITF,FOXP1,FOXP1,MIR1284,EIF4E3,EIF4E3,GPR27,GPR27,PROK2,RYBP,SHQ1,GXYLT2,PPP4R2,PPP4R2,EBLN2,PDZRN3,PDZRN3,LOC101927296,PDZRN3,PDZRN3-AS1,CNTN3,MIR1324,FRG2C,FRG2EP,ZNF717,ZNF717,MIR4273,ROBO2,ROBO1,ROBO1,MIR3923,GBE1,CADM2,CADM2,MIR5688,CADM2,CADM2-AS2,VGLL3,MIR4795,CHMP2B,POU1F1,HTR1F,CGGBP1,CGGBP1,ZNF654,C3orf38,EPHA3 | -0.412402 | 189.118 | 595 | 331.51 |
| 2 | 7 | 35058793 | 39125543 | DPY19L1,DPY19L2P1,TBX20,LOC401324,HERPUD2,SEPT7,EEPD1,KIAA0895,KIAA0895,ANLN,ANLN,AOAH,ELMO1,ELMO1,MIR1200,ELMO1,ELMO1-AS1,GPR141,NME8,SFRP4,EPDR1,STARD3NL,TARP,TRG-AS1,AMPH,FAM183BP,VPS41,POU6F2,POU6F2-AS2,POU6F2 | -0.415723 | 215.621 | 366 | 205.585 |
| 2 | 20 | 58498018 | 59430939 | FAM217B,PPP1R3D,FAM217B,CDH26,C20orf197,MIR646HG,MIR646,MIR4533,MIR548AG2 | -0.417909 | 215.297 | 52 | 29.1021 |
| 2 | 18 | 9134687 | 9235760 | ANKRD12 | -0.4208 | 159.586 | 11 | 5.82006 |
| 2 | 4 | 1.86E+08 | 186357376 | CENPU,ACSL1,MIR3945HG,MIR3945,MIR4455,HELT,LINC02436,SLC25A4,CFAP97,SNX25,LRP2BP,ANKRD37,ANKRD37,UFSP2,UFSP2,C4orf47 | -0.42481 | 200.516 | 110 | 65.3287 |

| | | | | | | | | |
|---|---|---|---|---|---|---|---|---|
| 2 | 4 | 71114775 | 84378159 | CSN3,CABS1,SMR3A,SMR3B,OPRPN,MUC7,AMTN,AMBN,ENAM,JCHAIN,UTP3,RUFY3,GRSF1,MOB1B,DCK,SLC4A4,GC,NPFFR2,ADAMTS3,COX18,ANKRD17,ALB,AFP,AFM,RASSF6,CXCL8,CXCL6,PF4V1,CXCL1,PF4,PPBP,CXCL5,CXCL3,CXCL2,MTHFD2L,EPGN,EREG,AREG,BTC,PARM1,RCHY1,THAP6,ODAPH,CDKL2,G3BP2,USO1,PPEF2,NAAA,SDAD1,SDAD1,LOC101928809,LOC101928809,CXCL9,CXCL9,ART3,CXCL10,ART3,CXCL11,ART3,NUP54,SCARB2,FAM47E,FAM47E,FAM47E-STBD1,FAM47E-STBD1,FAM47E-STBD1,STBD1,CCDC158,SHROOM3,SHROOM3,MIR4450,SOWAHB,SEPT11,CCNI,CCNG2,CXCL13,CNOT6L,MRPL1,FRAS1,ANXA3,BMP2K,PAQR3,NAA11,GK2,ANTXR2,PRDM8,FGF5,C4orf22,BMP3,PRKG2,PRKG2,LOC101928942,RASGEF1B,HNRNPD,HNRNPDL,ENOPH1,TMEM150C,SCD5,SCD5,MIR575,SEC31A,THAP9-AS1,THAP9,THAP9,LIN54,COPS4,PLAC8,COQ2,HPSE,HELQ,MRPS18C | -0.427894 | 226.152 | 1319 | 764.905 |
| 2 | 2 | 8938288 | 11312163 | KIDINS220,MBOAT2,ASAP2,ITGB1BP1,ITGB1BP1,CPSF3,CPSF3,IAH1,ADAM17,YWHAQ,TAF1B,GRHL1,KLF11,CYS1,RRM2,C2orf48,C2orf48,MIR4261,HPCAL1,ODC1,ODC1,SNORA80B,LOC101929715,NOL10,ATP6V1C2,ATP6V1C2,PDIA6,PDIA6,KCNF1,FLJ33534,C2orf50,PQLC3 | -0.431742 | 229.446 | 318 | 187.333 |
| 2 | 12 | 1.06E+08 | 107486000 | APPL2,C12orf75,NUAK1,CKAP4,TCP11L2,POLR3B,POLR3B,LOC100287944,LOC100287944,RFX4,LOC100287944,RFX4,LOC100505978,LOC100287944,RIC8B,RIC8B,TMEM263,MTERF2,CRY1 | -0.432777 | 187.333 | 181 | 103.216 |
| 2 | 7 | 1.44E+08 | 148534157 | TPK1,CNTNAP2,CNTNAP2,MIR548F4,CNTNAP2,MIR548T,C7orf33,CUL1,EZH2 | -0.433011 | 177.127 | 157 | 86.3329 |
| 2 | 15 | 83424597 | 84784933 | SNHG21,FSD2,SCARNA15,FSD2,WHAMM,HOMER2,FAM103A1,C15orf40,C15orf40,BTBD1,BTBD1,MIR4515,TM6SF1,HDGFL3,BNC1,SH3GL3,ADAMTSL3,EFL1P1 | -0.440392 | 217.967 | 190 | 105.47 |
| 2 | 2 | 2.02E+08 | 203644367 | C2CD6,TMEM237,MPP4,ALS2,CDK15,FZD7,KIAA2012,KIAA2012,LOC729224,SUMO1,NOP58,NOP58,SNORD70,NOP58,SNORD70B,NOP58,SNORD11B,NOP58,SNORD11,BMPR2,FAM117B,ICA1L | -0.440774 | 222.131 | 206 | 121.171 |
| 2 | 1 | 52226398 | 52991922 | OSBPL9,NRDC,NRDC,MIR761,RAB3B,TXNDC12,TXNDC12,KTI12,TXNDC12,TXNDC12-AS1,BTF3L4,ZFYVE9,CC2D1B,ORC1,PRPF38A,ZCCHC11 | -0.440974 | 224.64 | 216 | 126.89 |
| 2 | 11 | 46771748 | 46894684 | CKAP5,CKAP5,MIR5582,CKAP5,SNORD67,LRP4-AS1,LRP4 | -0.443674 | 209.507 | 51 | 34.1583 |
| 2 | 9 | 15509991 | 19058795 | PSIP1,CCDC171,C9orf92,BNC2,CNTLN,SH3GL2,ADAMTSL1,ADAMTSL1,MIR3152,SAXO1,RRAGA,HAUS6 | -0.452081 | 198.537 | 228 | 128.222 |
| 2 | 11 | 1.25E+08 | 125885148 | EI24,STT3A-AS1,STT3A-AS1,STT3A,CHEK1,CHEK1,ACRV1,ACRV1,PATE1,PATE2,PATE3,PATE4,HYLS1,PUS3,DDX25,CDON | -0.456639 | 204.472 | 121 | 75.0761 |

| | | | | | | | | |
|---|---|---|---|---|---|---|---|---|
| 2 | 4 | 1.04E+08 | 113524863 | CENPE,TACR3,CXXC4,CXXC4,CXXC4-AS1,TET2,TET2-AS1,PPA2,ARHGEF38,INTS12,GSTCD,GSTCD,LOC101929529,NPNT,LOC101929577,TBCK,TBCK,AIMP1,AIMP1,GIMD1,DKK2,PAPSS1,SGMS2,LOC101929595,LOC101929595,CYP2U1,CYP2U1,HADH,LEF1,LEF1,LEF1-AS1,RPL34-AS1,RPL34,OSTC,ETNPPL,COL25A1,SEC24B,MCUB,CASP6,PLA2G12A,CFI,GAR1,RRH,LRIT3,EGF,ELOVL6,ENPEP,PITX2,FAM241A,AP1AR,TIFA,ALPK1,NEUROG2,NEUROG2,LOC105377372,LOC105377372,ZGRF1,ZGRF1 | -0.459394 | 206.849 | 617 | 358.761 |
| 2 | 9 | 27062612 | 33280799 | IFT74,TEK,EQTN,MOB3B,MOB3B,IFNK,C9orf72,LINGO2,LINGO2,MIR876,LINGO2,MIR873,ACO1,DDX58,TOPORS,TOPORS,SMIM27,SMIM27,SMIM27,NDUFB6,NDUFB6,TAF1L,TMEM215,APTX,DNAJA1,SMU1,B4GALT1,B4GALT1,B4GALT1-AS1,SPINK4,BAG1,CHMP5 | -0.460312 | 225.63 | 273 | 160.504 |
| 2 | 12 | 62986376 | 64803971 | MON2,LINC01465,MIRLET7I,PPM1H,AVPR1A,DPY19L2,TMEM5,TMEM5,TMEM5-AS1,SRGAP1,C12orf66,C12orf56,XPOT | -0.462188 | 209.129 | 131 | 71.1839 |
| 2 | 15 | 38766397 | 40510846 | FAM98B,RASGRP1,C15orf53,C15orf54,THBS1,FSIP1,GPR176,EIF2AK4,SRP14,BMF,BUB1B,BUB1B,BUB1B-PAK6 | -0.46312 | 206.643 | 172 | 105.583 |
| 2 | 6 | 61944508 | 76330662 | KHDRBS2,LGSN,PTP4A1,PHF3,EYS,EYS,LOC441155,ADGRB3,LMBRD1,COL19A1,COL9A1,FAM135A,SDHAF4,SMAP1,SMAP1,B3GAT2,B3GAT2,OGFRL1,MIR30C2,MIR30A,RIMS1,KCNQ5,KCNQ5,MIR4282,KHDC1L,KHDC1,DPPA5,KHDC3L,OOEP,DDX43,CGAS,MTO1,EEF1A1,EEF1A1,SNORD141A,SNORD141B,SLC17A5,LOC101928489,CD109,CD109,COL12A1,COX7A2,TMEM30A,FILIP1,FILIP1,LOC101928540,FILIP1,LOC101928540,MIR4463,SENP6 | -0.463129 | 209.408 | 779 | 444.012 |
| 2 | 19 | 11728586 | 12659779 | ZNF627,ZNF833P,ZNF823,ZNF441,ZNF491,ZNF440,ZNF439,ZNF69,ZNF700,ZNF763,ZNF433-AS1,ZNF433,ZNF433-AS1,ZNF878,ZNF878,ZNF844,ZNF788,ZNF20,ZNF625-ZNF20,ZNF625-ZNF20,ZNF625-ZNF20,ZNF625,ZNF136,ZNF44,ZNF563,ZNF442,ZNF799,ZNF443,ZNF709,ZNF564 | -0.465134 | 245.044 | 275 | 151.132 |
| 2 | 1 | 1.79E+08 | 180068126 | MIR4424,RALGPS2,RALGPS2,ANGPTL1,FAM20B,TOR3A,ABL2,SOAT1,AXDND1,AXDND1,NPHS2,NPHS2,TDRD5,FAM163A,TOR1AIP2,TOR1AIP1,CEP350 | -0.466003 | 230.416 | 250 | 146.19 |

| | | | | | | | | |
|---|---|---|---|---|---|---|---|---|
| 2 | X | 10442671 | 46382620 | MID1,HCCS,ARHGAP6,ARHGAP6,AMELX,ARHGAP6,MIR548AX,MSL3,FRMPD4,PRPS2,TLR7,TLR8-AS1,TLR8,TMSB4X,FAM9C,GS1-600G8.3,ATXN3L,EGFL6,TCEANC,RAB9A,TRAPPC2,OFD1,GPM6B,GEMIN8,UBE2E4P,GLRA2,FANCB,MOSPD2,ASB9,ASB11,PIGA,VEGFD,PIR-FIGF,PIR-FIGF,PIR,BMX,ACE2,TMEM27,CA5BP1,CA5B,ZRSR2,AP1S2,GRPR,MAGEB17,CTPS2,CTPS2,MIR548AM,CTPS2,S100G,SYAP1,TXLNG,RBBP7,REPS2,NHS,NHS,MIR4768,SCML1,RAI2,BEND2,SCML2,CDKL5,CDKL5,RS1,RS1,PPEF1,PHKA2-AS1,PHKA2,PHKA2,ADGRG2,PDHA1,PDHA1,MAP3K15,MAP3K15,SH3KBP1,BCLAF3,MAP7D2,MAP7D2,MIR23C,EIF1AX,RPS6KA3,CNKSR2,KLHL34,SMPX,MBTPS2,MBTPS2,YY2,SMS,PHEX,PHEX,PHEX-AS1,PHEX,PTCHD1-AS,PTCHD1-AS,ZNF645,PTCHD1-AS,DDX53,PTCHD1,PRDX4,ACOT9,SAT1,APOO,CXorf58,KLHL15,EIF2S3,ZFX,SUPT20HL2,SUPT20HL1,PDK3,PCYT1B,POLA1,POLA1,SCARNA23,ARX,MAGEB18,MAGEB6,MAGEB5,VENTXP1,PPP4R3C,DCAF8L2,MAGEB10,DCAF8L1,IL1RAPL1,IL1RAPL1,MIR4666B,MAGEB2,MAGEB3,MAGEB4,MAGEB1,NR0B1,CXorf21,GK,TAB3,FTHL17,DMD,DMD,MIR3915,DMD,MIR548F5,FAM47A,TMEM47,FAM47B,MAGEB16,CFAP47,CFAP47,LOC101928627,FAM47C,PRRG1,LANCL3,XK,CYBB,DYNLT3,HYPM,SYTL5,MIR548AJ2,SYTL5,SRPX,RPGR,OTC,TSPAN7,MID1IP1,MIR3937,BCOR,ATP6AP2,MPC1L,CXorf38,MED14,MED14,MED14OS,MED14OS,USP9X,DDX3X,NYX,CASK,CASK,GPR34,CASK,GPR82,PPP1R2P9,MAOA,MAOB,NDP,NDP-AS1,EFHC2,FUNDC1,DUSP21,KDM6A,CXorf36,MIR221,MIR222,KRBOX4,ZNF674 | -0.466141 | 211.628 | 2316 | 1332.22 |
| 2 | 15 | 93485012 | 98511377 | CHD2,RGMA,LINC02207,LINC01579,MCTP2,NR2F2-AS1,NR2F2-AS1,NR2F2,NR2F2,NR2F2,MIR1469,SPATA8,LINC02254,LINC00923,ARRDC4 | -0.467538 | 188.972 | 147 | 84.8713 |
| 2 | 14 | 50953417 | 52898527 | MAP4K5,ATL1,SAV1,NIN,NIN,LOC105370489,ABHD12B,PYGL,TRIM9,TMX1,FRMD6,GNG2,GNG2,LOC102723604,RTRAF,NID2,PTGDR,PTGER2 | -0.468957 | 217.573 | 237 | 139.758 |
| 2 | 9 | 19102432 | 26982397 | HAUS6,PLIN2,DENND4C,RPS6,ACER2,SLC24A2,MLLT3,MLLT3,MIR4473,MLLT3,MIR4474,FOCAD,FOCAD,MIR491,HACD4,IFNB1,IFNW1,IFNA21,IFNA7,IFNA16,IFNA14,IFNA5,KLHL9,IFNA6,IFNA13,IFNA2,IFNA8,IFNA1,MIR31HG,IFNE,MIR31HG,MIR31,MTAP,CDKN2A-AS1,CDKN2A,CDKN2B-AS1,CDKN2B,CDKN2B-AS1,DMRTA1,ELAVL2,IZUMO3,TUSC1,LOC100506422,CAAP1,PLAA,PLAA,IFT74,IFT74 | -0.472078 | 217.891 | 361 | 204.526 |

| | | | | | | | | |
|---|---|---|---|---|---|---|---|---|
| 2 | 9 | 2719607 | 15468751 | KCNV2,PUM3,RFX3,GLIS3,GLIS3,GLIS3-AS1,SLC1A1,SPATA6L,SPATA6L,PLPP6,CDC37L1,AK3,RCL1,RCL1,MIR101-2,JAK2,INSL6,INSL4,RLN2,RLN1,PLGRKT,CD274,PDCD1LG2,RIC1,ERMP1,MLANA,KIAA2026,KIAA2026,MIR4665,RANBP6,IL33,TPD52L3,UHRF2,GLDC,KDM4C,DMAC1,PTPRD,TYRP1,TYRP1,LURAP1L-AS1,LURAP1L-AS1,LURAP1L,LURAP1L,MPDZ,LINC01235,LINC00583,NFIB,ZDHHC21,CER1,FREM1,LOC389705,TTC39B,SNAPC3,PSIP1 | -0.474664 | 204.321 | 742 | 424.624 |
| 2 | X | 1.34E+08 | 138668668 | MIR542,MIR503HG,MIR503,MIR503HG,MIR424,PLAC1,FAM122B,FAM122C,MOSPD1,SMIM10,RTL8B,RTL8C,RTL8A,SMIM10L2B-AS1,CT55,ZNF75D,ZNF449,INTS6L,CT45A10,SAGE1,MMGT1,SLC9A6,FHL1,MAP7D3,ADGRG4,BRS3,HTATSF1,VGLL1,VGLL1,MIR934,CD40LG,ARHGEF6,RBMX,RBMX,SNORD61,GPR101,ZIC3,FGF13,FGF13,MIR504,F9,MCF2 | -0.479717 | 220.628 | 388 | 224.053 |
| 2 | 19 | 9346684 | 9874002 | OR7E24,ZNF699,ZNF559,ZNF559-ZNF177,ZNF559-ZNF177,ZNF559-ZNF177,ZNF177,ZNF266,ZNF560,ZNF426,ZNF121,ZNF561,ZNF561-AS1,ZNF562,ZNF812P,ZNF846 | -0.482093 | 219.815 | 132 | 76.0772 |
| 2 | X | 1.54E+08 | 155260060 | GAB3,DKC1,DKC1,SNORA36A,MIR664B,DKC1,SNORA56,MPP1,SMIM9,F8,FUNDC2,CMC4,CMC4,MTCP1,BRCC3,VBP1,RAB39B,CLIC2,TMLHE-AS1,LOC101927830,TMLHE-AS1,LOC101927830,TMLHE,TMLHE,SPRY3,VAMP7,IL9R | -0.483509 | 183.558 | 188 | 103.824 |
| 2 | 4 | 1.56E+08 | 169013213 | MAP9,GUCY1A1,GUCY1B1,ASIC5,TDO2,CTSO,PDGFC,GLRB,GRIA2,FAM198B,FAM198B,FAM198B-AS1,TMEM144,RXFP1,C4orf46,ETFDH,PPID,FNIP2,FNIP2,C4orf45,C4orf45,RAPGEF2,FSTL5,FSTL5,LOC101928052,NAF1,NPY1R,NPY5R,TKTL2,TMA16,MARCH1,MARCH1,ANP32C,TRIM61,FAM218A,TRIM61,TRIM60,TMEM192,KLHL2,KLHL2,GK3P,MSMO1,CPE,CPE,MIR578,TLL1,SPOCK3 | -0.484086 | 192.58 | 561 | 323.217 |
| 2 | 3 | 60500 | 3217060 | CHL1,CHL1,CHL1-AS1,CNTN6,CNTN4,CNTN4,CNTN4-AS1,IL5RA,TRNT1,CRBN | -0.485167 | 186.915 | 150 | 88.3447 |
| 2 | 2 | 62066483 | 70057359 | FAM161A,CCT4,COMMD1,B3GNT2,MIR5192,B3GNT2,TMEM17,EHBP1,EHBP1,LOC100132215,OTX1,WDPCP,MDH1,UGP2,VPS54,PELI1,MIR4433B,MIR4433A,LGALSL,AFTPH,MIR4434,AFTPH,SERTAD2,LINC02245,SLC1A4,CEP68,RAB1A,ACTR2,SPRED2,MIR4778,MEIS1,MEIS1,MEIS1-AS2,ETAA1,C1D,WDR92,WDR92,PNO1,PNO1,PPP3R1,CNRIP1,PLEK,FBXO48,APLF,PROKR1,ARHGAP25,BMP10,GKN2,GKN1,ANTXR1,ANTXR1,MIR3126,GFPT1,NFU1,AAK1,AAK1,SNORA36C,ANXA4,GMCL1 | -0.485547 | 209.049 | 604 | 349.785 |
| 2 | 17 | 5044649 | 5384707 | USP6,ZNF594,LOC100130950,SCIMP,RABEP1,RABEP1,NUP88,NUP88,RPAIN,C1QBP,DHX33,DERL2 | -0.486973 | 204.953 | 112 | 66.4008 |

| | | | | | | | | |
|---|---|---|---|---|---|---|---|---|
| 2 | 1 | 55549439 | 62904991 | USP24,MIR4422HG,MIR4422,PLPP3,LOC101929935,PRKAA2,PRKAA2,FYB2,C8A,C8B,DAB1,OMA1,TACSTD2,MYSM1,JUN,LINC01135,FGGY,FGGY,MIR4711,HOOK1,CYP2J2,C1orf87,NFIA,TM2D1,PATJ,PATJ,MIR3116-1,MIR3116-2,L1TD1,KANK4 | -0.491472 | 199.24 | 451 | 258.547 |
| 2 | 4 | 57366803 | 68337860 | SRP72,ARL9,THEGL,HOPX,SPINK2,REST,NOA1,POLR2B,IGFBP7,IGFBP7,IGFBP7-AS1,MIR548AG1,ADGRL3,TECRL,EPHA5,MIR1269A | -0.494426 | 167.97 | 249 | 144.363 |
| 2 | 12 | 1.24E+08 | 124377928 | MPHOSPH9,C12orf65,CDK2AP1,SBNO1,KMT5A,RILPL2,SNRNP35,RILPL1,MIR3908,TMED2,DDX55,EIF2B1,GTF2H3,TCTN2,ATP6V0A2,DNAH10 | -0.496349 | 221.812 | 238 | 146.001 |
| 2 | 10 | 51885769 | 70666515 | WASHC2A,ASAH2,SGMS1,SGMS1-AS1,ASAH2B,A1CF,PRKG1,PRKG1,MIR605,PRKG1,CSTF2T,DKK1,MBL2,PCDH15,PCDH15,LOC105378311,PCDH15,LOC105378311,MIR548F1,ZWINT,MIR3924,IPMK,CISD1,UBE2D1,TFAM,BICC1,PHYHIPL,PHYHIPL,FAM13C,FAM13C,SLC16A9,CCDC6,LINC01553,ANK3,CDK1,RHOBTB1,TMEM26,TMEM26,TMEM26-AS1,CABCOCO1,ARID5B,RTKN2,ZNF365,ADO,EGR2,NRBF2,JMJD1C,JMJD1C,MIR1296,JMJD1C,JMJD1C-AS1,JMJD1C,REEP3,REEP3,CTNNA3,CTNNA3,LOC101928961,LRRTM3,CTNNA3,LRRTM3,DNAJC12,SIRT1,HERC4,MYPN,ATOH7,PBLD,HNRNPH3,HNRNPH3,RUFY2,RUFY2,DNA2,SLC25A16,TET1,CCAR1,CCAR1,SNORD98,CCAR1,MIR1254-1,STOX1,DDX50 | -0.496466 | 215.146 | 958 | 558.927 |
| 2 | 7 | 82538165 | 87514515 | PCLO,SEMA3E,SEMA3A,SEMA3D,GRM3,KIAA1324L,LOC101927420,DMTF1,DMTF1,TMEM243,CROT,ABCB4,ABCB1,ABCB1,RUNDC3B,RUNDC3B,SLC25A40,DBF4 | -0.499157 | 225.346 | 345 | 203.561 |
| 2 | 2 | 75929334 | 85043248 | GCFC2,LRRTM4,REG3G,REG1B,REG1A,REG3A,CTNNA2,CTNNA2,MIR4264,CTNNA2,LRRTM1,FUNDC2P2,SUCLG1,DNAH6 | -0.501926 | 177.819 | 238 | 140.871 |
| 2 | 4 | 84406139 | 104032182 | ABRAXAS1,GPAT3,LOC101928978,NKX6-1,CDS1,WDFY3,ARHGAP24,ARHGAP24,MIR4451,MAPK10,MAPK10,LOC101929064,PTPN13,SLC10A6,C4orf36,AFF1,KLHL8,HSD17B13,HSD17B11,NUDT9,SPARCL1,DSPP,DMP1,IBSP,MEPE,SPP1,PKD2,ABCG2,PPM1K,HERC6,HERC5,PYURF,PIGY,HERC3,HERC3,NAP1L5,FAM13A-AS1,FAM13A,FAM13A,TIGD2,GPRIN3,SNCA,MMRN1,CCSER1,GRID2,ATOH1,SMARCAD1,HPGDS,PDLIM5,BMPR1B,UNC5C,PDHA2,STPG2,RAP1GDS1,TSPAN5,EIF4E,METAP1,METAP1,MIR3684,ADH5,LOC100507053,ADH4,LOC100507053,ADH6,LOC100507053,ADH1A,ADH1B,ADH1C,ADH7,C4orf17,TRMT10A,MTTP,LOC285556,DAPP1,LAMTOR3,DNAJB14,H2AFZ,DDIT4L,DDIT4L,LOC101929353,EMCN,PPP3CA,PPP3CA,MIR1255A,FLJ20021,BANK1,SLC39A8,NFKB1,MANBA,LOC102723704,UBE2D3,UBE2D3,CISD2,CISD2,SLC9B1,SLC9B1,SLC9B2,BDH2,CENPE | -0.502839 | 208.616 | 1309 | 758.29 |
| 2 | 1 | 2.26E+08 | 225976978 | DNAH14,LBR,ENAH,SRP9 | -0.506363 | 226.548 | 75 | 42.6368 |

| | | | | | | | | |
|---|---|---|---|---|---|---|---|---|
| 2 | 10 | 74127984 | 75239272 | MICU1,MCU,MCU,MIR4676,OIT3,PLA2G12B,P4HA1,NUDT13,ECD,FAM149B1,DNAJC9,DNAJC9,DNAJC9-AS1,DNAJC9-AS1,DNAJC9-AS1,MRPS16,DNAJC9-AS1,CFAP70,CFAP70,ANXA7,MSS51,PPP3CB | -0.506818 | 206.122 | 210 | 120.02 |
| 2 | 4 | 1.41E+08 | 145916079 | UCP1,TBC1D9,RNF150,ZNF330,IL15,INPP4B,USP38,GAB1,GAB1,MIR3139,SMARCA5,SMARCA5-AS1,SMARCA5,FREM3,GYPE,GYPB,GYPA,HHIP | -0.507012 | 201.926 | 292 | 165.404 |
| 2 | 5 | 68709931 | 70820157 | RAD17,MARVELD2,OCLN,GTF2H2C_2,GTF2H2C,SERF1A,SERF1B,SMN1,SMN2,GUSBP3,GTF2H2B,GUSBP3,NAIP,GTF2H2,LOC647859,BDP1 | -0.509177 | 191.593 | 140 | 68.5777 |
| 2 | 13 | 25442693 | 32893507 | RNF17,CENPJ,TPTE2P1,PABPC3,AMER2,MTMR6,NUP58,ATP8A2,SHISA2,RNF6,CDK8,WASF3,GPR12,USP12,RPL21,RPL21P28,RPL21,RPL21P28,SNORD102,RPL21,RPL21P28,SNORA27,RASL11A,GTF3A,GTF3A,MTIF3,MTIF3,LNX2,POLR1D,GSX1,PDX1,CDX2,URAD,FLT3,PAN3-AS1,PAN3,PAN3,FLT1,POMP,SLC46A3,MTUS2,MTUS2,MTUS2-AS1,SLC7A1,UBL3,LINC00544,KATNAL1,HMGB1,USPL1,ALOX5AP,TEX26-AS1,MEDAG,TEX26,HSPH1,B3GLCT,RXFP2,FRY,ZAR1L,BRCA2 | -0.510916 | 208.642 | 694 | 408.891 |
| 2 | 17 | 65740394 | 66873898 | BPTF,C17orf58,KPNA2,LINC00674,ARHGAP27P2,AMZ2,ARSG,SLC16A6,ARSG,ARSG,PRKAR1A,ARSG,PRKAR1A,WIPI1,ARSG,PRKAR1A,WIPI1,MIR635,PRKAR1A,WIPI1,PRKAR1A,PRKAR1A,FAM20A,FAM20A,ABCA8 | -0.512784 | 220.793 | 183 | 104.012 |
| 2 | 5 | 72359596 | 73989621 | FCHO2,TMEM171,TMEM174,FOXD1,BTF3,ANKRA2,UTP15,ARHGEF28,ENC1,HEXB | -0.513172 | 189.257 | 117 | 69.0354 |
| 2 | 2 | 2.14E+08 | 216226889 | SPAG16,SPAG16,MIR4438,VWC2L,BARD1,LOC101928103,ABCA12,ABCA12,ATIC,FN1 | -0.521685 | 185.881 | 137 | 79.9071 |
| 2 | 10 | 37509237 | 39076015 | ANKRD30A,ZNF248,ZNF25,ZNF33A,ZNF37A,HSD17B7P2,SEPT7P9,LINC00999 | -0.525063 | 194.475 | 86 | 43.7904 |
| 2 | 2 | 1.52E+08 | 153512978 | NEB,ARL5A,CACNB4,STAM2,FMNL2,PRPF40A | -0.525805 | 220.976 | 254 | 152.803 |
| 2 | 11 | 9467174 | 17111443 | ZNF143,WEE1,SWAP70,SBF2-AS1,SBF2,SBF2,SBF2,LOC101928008,ADM,AMPD3,RNF141,MRVI1-AS1,LYVE1,MRVI1-AS1,MRVI1,MRVI1,CTR9,EIF4G2,EIF4G2,SNORD97,ZBED5,GALNT18,GALNT18,CSNK2A3,MIR4299,USP47,DKK3,MICAL2,MICALCL,PARVA,TEAD1,RASSF10,ARNTL,BTBD10,PTH,FAR1,SPON1,RRAS2,COPB1,PSMA1,PDE3B,CYP2R1,CALCA,CALCB,INSC,SOX6,C11orf58,PLEKHA7,OR7E14P,RPS13,PIK3C2A | -0.526831 | 193.328 | 684 | 396.083 |
| 2 | 2 | 1.89E+08 | 190553735 | GULP1,DIRC1,COL3A1,COL3A1,MIR1245A,MIR1245B,COL5A2,COL5A2,MIR3129,WDR75,SLC40A1,ASDURF,ASNSD1,ASNSD1,ANKAR | -0.526918 | 176.597 | 167 | 96.9948 |
| 2 | 10 | 75802827 | 76744983 | VCL,AP3M1,ADK,ADK,LOC102723439,KAT6B | -0.530163 | 177.822 | 75 | 44.2335 |
| 2 | 19 | 36831104 | 38314183 | ZFP14,ZFP82,ZNF566,ZNF566,LOC728752,LOC728752,ZNF260,ZNF529,ZNF529,ZNF529-AS1,ZNF382,ZNF461,ZNF567,ZNF850,ZNF790-AS1,ZNF790,ZNF345,ZNF345,ZNF829,ZNF829,ZNF568,ZNF420,ZNF585A,ZNF585B,ZNF383,HKR1,ZNF527,ZNF569,ZNF570,ZNF793,ZNF571-AS1,ZNF571-AS1,ZNF540,ZNF571-AS1,ZNF540,ZNF571,ZNF540,ZFP30,ZNF781,ZNF607,ZNF573 | -0.533695 | 191.881 | 345 | 196.312 |

| | | | | | | | | |
|---|---|---|---|---|---|---|---|---|
| 2 | 7 | 99144312 | 99514405 | FAM200A,ZNF655,TMEM225B,ZSCAN25,ZSCAN25,CYP3A5,CYP3A5,CYP3A7-CYP3A51P,CYP3A7-CYP3A51P,CYP3A7,CYP3A4,CYP3A43,OR2AE1,TRIM4 | -0.534139 | 232.618 | 121 | 70.7914 |
| 2 | 10 | 27382306 | 28908662 | ANKRD26,YME1L1,MASTL,MASTL,ACBD5,ACBD5,LRRC37A6P,ARMC4P1,PTCHD3,RAB18,MKX,ARMC4,MPP7,WAC | -0.537222 | 233.332 | 178 | 100.433 |
| 2 | X | 1.2E+08 | 122757159 | CUL4B,MCTS1,C1GALT1C1,CT47B1,GLUD2,MIR3672,GRIA3,THOC2 | -0.538862 | 151.321 | 97 | 53.4682 |
| 2 | 2 | 1.78E+08 | 179395177 | AGPS,TTC30B,TTC30A,PDE11A,PDE11A,LOC105373764,RBM45,OSBPL6,OSBPL6,MIR548N,MIR548N,LOC101927027,PRKRA,MIR548N,PRKRA,MIR548N,PJVK,MIR548N,FKBP7,MIR548N,PLEKHA3,MIR548N,TTN-AS1,TTN | -0.540939 | 233.19 | 140 | 79.9286 |
| 2 | 14 | 93676979 | 94173127 | UBR7,BTBD7,UNC79,COX8C,UNC79 | -0.541065 | 209.564 | 103 | 60.9903 |
| 2 | 17 | 38784950 | 39134635 | SMARCE1,KRT222,KRT24,KRT25,KRT26,KRT27,KRT28,KRT10,KRT10,TMEM99,TMEM99,KRT12,KRT20,KRT23,KRT39,KRT40 | -0.541743 | 230.343 | 132 | 77.9115 |
| 2 | 9 | 86407814 | 88961419 | GKAP1,KIF27,C9orf64,HNRNPK,HNRNPK,MIR7-1,RMI1,SLC28A3,NTRK2,AGTPBP1,LOC389765,NAA35,GOLM1,C9orf153,ISCA1,ZCCHC6 | -0.544955 | 224.694 | 248 | 144.326 |
| 2 | 20 | 13756487 | 17207489 | ESF1,NDUFAF5,SEL1L2,MACROD2,MACROD2,FLRT3,MACROD2,LOC613266,KIF16B,SNRPB2,OTOR | -0.546725 | 209.825 | 154 | 88.4153 |
| 2 | 11 | 1.2E+08 | 120338072 | POU2F3,TMEM136,ARHGEF12 | -0.5519 | 182.474 | 52 | 32.0321 |
| 2 | X | 86772829 | 96140129 | KLHL4,CPXCR1,TGIF2LX,PABPC5,PCDH11X,NAP1L3,FAM133A,MIR548M,DIAPH2,DIAPH2,RPA4 | -0.554901 | 165.356 | 144 | 79.1991 |
| 2 | 3 | 1.12E+08 | 122606224 | CD200,BTLA,ATG3,ATG3,SLC35A5,SLC35A5,CCDC80,LOC100129297,CD200R1L,CD200R1L,CD200R1,GTPBP8,NEPRO,BOC,BOC,CFAP44,CFAP44,CFAP44,CFAP44-AS1,SPICE1,SIDT1,SIDT1,MIR4446,USF3,NAA50,ATP6V1A,GRAMD1C,ZDHHC23,CCDC191,QTRT2,DRD3,ZNF80,TIGIT,ZBTB20,MIR568,ZBTB20,ZBTB20,ZBTB20-AS1,ZBTB20,MIR4796,GAP43,LSAMP,MIR4447,LOC105374060,IGSF11,C3orf30,UPK1B,B4GALT4,B4GALT4,B4GALT4-AS1,ARHGAP31,TMEM39A,POGLUT1,TIMMDC1,CD80,ADPRH,PLA1A,POPDC2,COX17,MAATS1,NR1I2,GSK3B,GPR156,LRRC58,FSTL1,MIR198,FSTL1,NDUFB4,HGD,RABL3,GTF2E1,STXBP5L,STXBP5L,MIR5682,POLQ,ARGFX,FBXO40,HCLS1,GOLGB1,IQCB1,EAF2,SLC15A2,ILDR1,CD86,CASR,CSTA,CCDC58,FAM162A,WDR5B,LOC102723582,KPNA1,KPNA1,PARP9,PARP9,DTX3L,DTX3L,PARP15,PARP14,HSPBAP1,DIRC2,LINC02035 | -0.55562 | 203.267 | 1098 | 664.896 |
| 2 | 2 | 2.42E+08 | 242376006 | HDLBP,SEPT2,FARP2 | -0.557304 | 209.906 | 50 | 29.0425 |
| 2 | 7 | 1.22E+08 | 127026264 | PTPRZ1,AASS,FEZF1,FEZF1,FEZF1-AS1,CADPS2,CADPS2,RNF133,CADPS2,RNF148,TAS2R16,SLC13A1,IQUB,NDUFA5,ASB15,LOC102724555,ASB15,LMOD2,WASL,HYAL4,SPAM1,TMEM229A,GPR37,C7orf77,POT1,GRM8,GRM8,MIR592,ZNF800 | -0.557525 | 213.525 | 328 | 198.444 |

| | | | | | | | | |
|---|---|---|---|---|---|---|---|---|
| 2 | 6 | 88332301 | 109468182 | ORC3,AKIRIN2,SPACA1,CNR1,RNGTT,PNRC1,SRSF12,PM20D2,GABRR1,GABRR2,UBE2J1,RRAGD,ANKRD6,LYRM2,LYRM2,LOC101929057,LOC101929057,MDN1,MDN1,CASP8AP2,GJA10,BACH2,MIR4464,MAP3K7,MIR4643,EPHA7,MANEA,FUT9,UFL1,FHL5,GPR63,NDUFAF4,KLHL32,KLHL32,MIR548H3,MIR548H3,MMS22L,MIR2113,POU3F2,FBXL4,MIR548AI,FAXC,COQ3,PNISR,USP45,TSTD3,CCNC,PRDM13,MCHR2,SIM1,ASCC3,GRIK2,HACE1,LIN28B-AS1,LIN28B,BVES,BVES-AS1,BVES-AS1,POPDC3,PREP,PRDM1,ATG5,LOC105377924,CRYBG1,RTN4IP1,RTN4IP1,QRSL1,QRSL1,LINC02532,MIR587,C6orf203,BEND3,PDSS2,SOBP,SCML4,SEC63,OSTM1,NR2E1,SNX3,AFG1L,FOXO3,ARMC2,ARMC2,ARMC2-AS1,SESN1,CEP57L1 | -0.559667 | 202.73 | 1145 | 668.286 |
| 2 | 7 | 76883692 | 76990274 | CCDC146,GSAP | -0.559899 | 185.07 | 38 | 23.2525 |
| 2 | 2 | 1.34E+08 | 152267147 | NCKAP5,NCKAP5,LOC101928185,MIR3679,MGAT5,TMEM163,ACMSD,ACMSD,CCNT2-AS1,CCNT2,MAP3K19,RAB3GAP1,ZRANB3,R3HDM1,R3HDM1,MIR128-1,UBXN4,LCT,LCT,LOC100507600,MCM6,DARS,DARS,DARS-AS1,CXCR4,THSD7B,HNMT,SPOPL,NXPH2,LRP1B,KYNU,ARHGAP15,LOC101928386,GTDC1,GTDC1,ZEB2,PABPC1P2,ACVR2A,ORC4,MBD5,EPC2,KIF5C,LYPD6B,LYPD6,MMADHC,RND3,RBM43,NMI,LOC101929319,TNFAIP6,LOC101929319,TNFAIP6,MIR4773-1,MIR4773-2,RIF1 | -0.561203 | 192.557 | 843 | 491.316 |
| 2 | 17 | 35310451 | 36375187 | AATF,AATF,MIR2909,ACACA,ACACA,SNORA90,ACACA,C17orf78,TADA2A,DUSP14,SYNRG,DDX52,HNF1B,YWHAEP7,LOC440434 | -0.56153 | 217.526 | 195 | 111.696 |
| 2 | 5 | 1.41E+08 | 148016690 | RNF14,GNPDA1,NDFIP1,SPRY4,FGF1,ARHGAP26,NR3C1,MIR5197,HMHB1,YIPF5,KCTD16,PRELID2,GRXCR2,SH3RF2,PLAC8L1,LARS,RBM27,POU4F3,TCERG1,GPR151,PPP2R2B,STK32A,DPYSL3,JAKMIP2-AS1,JAKMIP2-AS1,JAKMIP2,JAKMIP2,SPINK1,SCGB3A2,C5orf46,SPINK5,SPINK14,SPINK6,LOC102546294,SPINK13,LOC102546294,SPINK7,LOC102546294,LOC102546294,SPINK9,FBXO38,HTR4 | -0.562866 | 188.484 | 491 | 287.011 |
| 2 | 2 | 1.79E+08 | 180809309 | MIR548N,TTN-AS1,TTN,MIR548N,TTN,TTN,TTN,LOC101927055,CCDC141,SESTD1,ZNF385B,ZNF385B,MIR1258 | -0.56314 | 229.359 | 369 | 243.37 |
| 2 | 17 | 61784004 | 61894406 | STRADA,CCDC47,DDX42 | -0.563538 | 212.36 | 38 | 23.9559 |
| 2 | 5 | 1.5E+08 | 150322315 | RBM22,DCTN4,SMIM3,IRGM,IRGM,ZNF300,ZNF300,ZNF300P1 | -0.565325 | 209.905 | 48 | 27.7409 |

| | | | | | | | | |
|---|---|---|---|---|---|---|---|---|
| 2 | 11 | 17868134 | 32615561 | SERGEF,TPH1,SAAL1,MRGPRX3,MRGPRX4,LOC494141,SAA4,SAA2-SAA4,SAA2-SAA4,SAA2,SAA1,HPS5,GTF2H1,LDHA,LDHC,LDHAL6A,TSG101,UEVLD,SPTY2D1OS,SPTY2D1,SPTY2D1,TMEM86A,TMEM86A,IGSF22,IGSF22,PTPN5,MRGPRX1,MRGPRX2,ZDHHC13,CSRP3,E2F8,NAV2,NAV2,MIR4486,NAV2,LOC100126784,NAV2,MIR4694,NAV2,NAV2-AS2,DBX1,HTATIP2,PRMT3,SLC6A5,NELL1,ANO5,SLC17A6,FANCF,GAS2,SVIP,CCDC179,LUZP2,ANO3,ANO3,MUC15,SLC5A12,FIBIN,BBOX1,BBOX1-AS1,CCDC34,LGR4,LGR4,LOC105376671,LIN7C,LIN7C,BDNF-AS,BDNF-AS,BDNF,BDNF,KIF18A,KIF18A,MIR610,METTL15,KCNA4,FSHB,ARL14EP,MPPED2,DCDC1,DNAJC24,DNAJC24,IMMP1L,IMMP1L,ELP4,PAX6,PAX6,PAX6-AS1,RCN1,WT1,WT1-AS,EIF3M | -0.569748 | 202.47 | 925 | 531.48 |
| 2 | 13 | 58299962 | 88484001 | DIAPH3,DIAPH3,DIAPH3-AS1,TDRD3,MIR3169,PCDH20,MIR548X2,MIR4704,PCDH9,PCDH9,PCDH9-AS2,KLHL1,KLHL1,ATXN8OS,DACH1,MZT1,BORA,DIS3,PIBF1,KLF5,KLF12,LOC100288208,TBC1D4,COMMD6,UCHL3,UCHL3,LMO7-AS1,LMO7-AS1,LMO7,LMO7,LMO7DN,KCTD12,ACOD1,CLN5,FBXL3,MYCBP2,MYCBP2,MYCBP2-AS1,SCEL,SCEL,SCEL-AS1,SLAIN1,MIR3665,SLAIN1,EDNRB-AS1,EDNRB,EDNRB,RNF219-AS1,POU4F1,RNF219-AS1,RNF219,RNF219,RBM26,NDFIP2-AS1,NDFIP2,NDFIP2,SPRY2,SLITRK1,SLITRK6,MIR4500HG,MIR4500,SLITRK5 | -0.573003 | 178.948 | 793 | 461.922 |
| 2 | 15 | 57025696 | 60720911 | ZNF280D,LOC145783,TCF12,TCF12,CGNL1,MYZAP,GCOM1,GCOM1,GCOM1,POLR2M,ALDH1A2,ALDH1A2,LOC283665,AQP9,LIPC,LIPC,LIPC-AS1,ADAM10,ADAM10,HSP90AB4P,MINDY2,SLTM,RNF111,CCNB2,MYO1E,MYO1E,MIR2116,MYO1E,LDHAL6B,FAM81A,GCNT3,GTF2A2,BNIP2,FOXB1,ANXA2,ICE2 | -0.573271 | 194.549 | 363 | 212.001 |
| 2 | 11 | 47640339 | 51566742 | MTCH2,AGBL2,FNBP4,NUP160,PTPRJ,PTPRJ,MIR3161,OR4B1,OR4X2,OR4X1,OR4S1,OR4C3,OR4C45,OR4C5,OR4A47,TRIM49B,TRIM64C,FOLH1,LOC440040,OR4C13,OR4C12,LOC441601,OR4A5,OR4C46 | -0.573392 | 226.425 | 300 | 166.821 |
| 2 | 6 | 44320439 | 50681198 | SPATS1,CDC5L,CDC5L,MIR4642,SUPT3H,SUPT3H,MIR586,SUPT3H,RUNX2,RUNX2,CLIC5,ENPP4,ENPP5,RCAN2,RCAN2,LOC101926915,CYP39A1,SLC25A27,SLC25A27,LOC101926934,LOC101926934,TDRD6,TDRD6,PLA2G7,ANKRD66,MEP1A,ADGRF5,ADGRF5,LOC101926962,ADGRF1,TNFRSF21,CD2AP,ADGRF2,ADGRF4,OPN5,PTCHD4,MUT,CENPQ,GLYATL3,C6orf141,RHAG,CRISP2,CRISP3,PGK2,CRISP1,DEFB133,DEFB114,DEFB113,DEFB110,DEFB112 | -0.573499 | 198.111 | 453 | 268.707 |
| 2 | X | 65260400 | 67518992 | HEPH,EDA2R,AR,OPHN1 | -0.577398 | 155.246 | 101 | 58.0073 |

| | | | | | | | | |
|---|---|---|---|---|---|---|---|---|
| 2 | 8 | 47458041 | 77615793 | LINC00293,LOC100287846,SPIDR,CEBPD,PRKDC,MCM4,UBE2V2,EFCAB1,SNAI2,PPDPFL,SNTG1,PXDNL,PCMTD1,ST18,ALKAL1,RB1CC1,NPBWR1,OPRK1,ATP6V1H,RGS20,TCEA1,LYPLA1,MRPL15,SOX17,RP1,XKR4,TMEM68,TGS1,LYN,RPS20,RPS20,SNORD54,MOS,PLAG1,CHCHD7,SDR16C5,SDR16C6P,PENK,PENK,LOC101929415,IMPAD1,FAM110B,UBXN2B,CYP7A1,SDCBP,NSMAF,TOX,CA8,RAB2A,CHD7,LOC100130298,CLVS1,ASPH,MIR4470,NKAIN3,NKAIN3,UG0898H09,GGH,TTPA,YTHDF3,MIR124-2HG,MIR124-2HG,MIR124-2,LOC401463,BHLHE22,CYP7B1,LINC01299,ARMC1,MTFR1,PDE7A,DNAJC5B,TRIM55,CRH,LINC00967,RRS1,ADHFE1,C8orf46,MYBL1,VCPIP1,C8orf44,C8orf44-SGK3,C8orf44-SGK3,SGK3,PTTG3P,C8orf44-SGK3,SGK3,MCMDC2,SNHG6,SNORD87,TCF24,PPP1R42,COPS5,CSPP1,ARFGEF1,LOC102724708,CPA6,CPA6,PREX2,C8orf34-AS1,C8orf34-AS1,C8orf34,C8orf34,SULF1,SLCO5A1,PRDM14,NCOA2,TRAM1,LACTB2-AS1,LACTB2,LACTB2,XKR9,EYA1,MSC,MSC,MSC-AS1,MSC-AS1,MSC-AS1,TRPA1,TRPA1,LOC392232,KCNB2,TERF1,SBSPON,C8orf89,RPL7,RDH10,RDH10,RDH10-AS1,STAU2-AS1,STAU2-AS1,STAU2,STAU2,UBE2W,ELOC,TMEM70,LY96,JPH1,GDAP1,MIR5681A,MIR5681B,MIR2052HG,MIR2052HG,MIR2052,PI15,CRISPLD1,HNF4G | -0.581063 | 198.819 | 1797 | 1032.89 |
| 2 | 3 | 1.6E+08 | 167006755 | KPNA4,ARL14,PPM1L,B3GALNT1,NMD3,SPTSSB,LINC02067,OTOL1,MIR1263,SI,SLITRK3,BCHE,ZBBX | -0.58821 | 183.641 | 181 | 103.924 |
| 2 | 7 | 1.02E+08 | 120990080 | FAM185A,FAM185A,FBXL13,FBXL13,FBXL13,LRRC17,ARMC10,NAPEPLD,DPY19L2P2,PMPCB,PMPCB,DNAJC2,DNAJC2,PSMC2,PSMC2,SLC26A5,SLC26A5,LOC101927870,RELN,RELN,ORC5,LHFPL3,LHFPL3,LHFPL3-AS2,KMT2E,SRPK2,PUS7,RINT1,RINT1,EFCAB10,EFCAB10,ATXN7L1,CDHR3,SYPL1,NAMPT,CCDC71L,PIK3CG,PRKAR2B,HBP1,COG5,COG5,GPR22,COG5,DUS4L,DUS4L,BCAP29,SLC26A4-AS1,SLC26A4,SLC26A4,LOC101927974,CBLL1,CBLL1,SLC26A3,DLD,LAMB1,LAMB4,NRCAM,PNPLA8,THAP5,DNAJB9,C7orf66,EIF3IP1,IMMP2L,IMMP2L,LRRN3,DOCK4,DOCK4,DOCK4-AS1,ZNF277,IFRD1,LSMEM1,TMEM168,BMT2,GPR85,SMIM30,PPP1R3A,FOXP2,FOXP2,MIR3666,MDFIC,TFEC,TES,CAV2,CAV1,MET,CAPZA2,ST7-AS1,ST7,ST7,ST7-OT4,ST7,ST7,ST7-AS2,ST7,ST7-OT3,WNT2,ASZ1,CFTR,CFTR,CFTR-AS1,CTTNBP2,LSM8,ANKRD7,KCND2,TSPAN12,ING3,CPED1,WNT16 | -0.59048 | 216.601 | 1362 | 801.279 |
| 2 | 10 | 97786985 | 98311170 | ENTPD1-AS1,CC2D2B,ENTPD1-AS1,CCNJ,ENTPD1-AS1,MIR3157,ZNF518A,ZNF518A,BLNK,BLNK,DNTT,OPALIN,TLL2,TM9SF3 | -0.59615 | 208.332 | 113 | 67.3747 |

| | | | | | | | | |
|---|---|---|---|---|---|---|---|---|
| 2 | 18 | 18651139 | 30518068 | ROCK1,GREB1L,ESCO1,SNRPD1,ABHD3,ABHD3,MIR320C1,MIB1,MIB1,MIR133A1HG,MIR133A1,MIB1,MIR133A1HG,MIR1-2,GATA6,CTAGE1,RBBP8,MIR4741,RBBP8,CABLES1,TMEM241,RIOK3,RMC1,RMC1,NPC1,NPC1,ANKRD29,LAMA3,TTC39C,TTC39C-AS1,TTC39C,CABYR,OSBPL1A,OSBPL1A,MIR320C2,IMPACT,HRH4,ZNF521,SS18,PSMA8,TAF4B,KCTD1,AQP4,AQP4,AQP4-AS1,AQP4-AS1,CHST9,CHST9,CDH2,MIR302F,DSC3,DSC2,DSC2,DSCAS,DSCAS,DSC1,DSG1,DSG1,DSG1-AS1,DSG1-AS1,DSG4,DSG3,DSG2,DSG2,DSG2-AS1,TTR,B4GALT6,SLC25A52,TRAPPC8,RNF125,RNF138,MEP1B,GAREM1,WBP11P1,KLHL14,CCDC178 | -0.597881 | 201.207 | 891 | 526.92 |
| 2 | 4 | 1.14E+08 | 123857383 | ANK2,ANK2,MIR1243,CAMK2D,ARSJ,UGT8,UGT8,MIR577,NDST4,TRAM1L1,NDST3,SNHG8,SNORA24,PRSS12,CEP170P1,METTL14,SEC24D,SYNPO2,MYOZ2,LOC101929762,USP53,C4orf3,FABP2,LOC645513,PDE5A,PDE5A,MAD2L1,PRDM5,NDNF,TNIP3,QRFPR,ANXA5,TMEM155,PP12613,EXOSC9,EXOSC9,CCNA2,CCNA2,BBS7,TRPC3,KIAA1109,ADAD1,IL2,IL21,IL21,IL21-AS1,BBS12,FGF2,FGF2,NUDT6,NUDT6,SPATA5 | -0.598981 | 203.343 | 724 | 434.035 |
| 2 | 5 | 49555074 | 54551778 | EMB,PARP8,ISL1,LINC02118,PELO,ITGA1,ITGA1,ITGA2,MOCS2,FST,NDUFS4,ARL15,ARL15,MIR581,ARL15,MIR4459,HSPB3,SNX18,ESM1,GZMK,GZMA,CDC20B,CDC20B,GPX8,CDC20B,MIR449A,MIR449B,CDC20B,MIR449C,MCIDAS,CCNO | -0.599703 | 189.693 | 258 | 150.485 |
| 2 | 2 | 21366629 | 24914559 | KLHL29,ATAD2B,UBXN2A,MFSD2B,WDCP,FKBP1B,SF3B6,FAM228B,TP53I3,FAM228B,PFN4,FAM228B,FAM228A,ITSN2,NCOA1 | -0.601395 | 192.481 | 209 | 124.582 |
| 2 | 17 | 19683791 | 20305419 | ULK2,AKAP10,SPECC1,CCDC144CP | -0.607768 | 191.972 | 93 | 51.8986 |
| 2 | 2 | 86088300 | 86406647 | ST3GAL5,POLR1A,PTCD3,PTCD3,SNORD94,IMMT | -0.607801 | 195.685 | 89 | 53.1562 |
| 2 | 2 | 1.72E+08 | 176860449 | DCAF17,CYBRD1,DYNC1I2,SLC25A12,HAT1,METAP1D,DLX1,DLX2,ITGA6,PDK1,RAPGEF4-AS1,RAPGEF4,RAPGEF4,MAP3K20,MAP3K20,MAP3K20-AS1,CDCA7,SP3,OLA1,SP9,CIR1,CIR1,SCRN3,SCRN3,GPR155,WIPF1,CHRNA1,CHN1,ATF2,ATF2,MIR933,ATP5MC3,LNPK | -0.608355 | 191.104 | 403 | 230.236 |
| 2 | 11 | 32782264 | 33362724 | PRRG4,QSER1,DEPDC7,TCP11L1,CSTF3,HIPK3 | -0.611925 | 213.646 | 92 | 56.6456 |
| 2 | 8 | 42206513 | 43398986 | POLB,DKK4,VDAC3,SLC20A2,SMIM19,CHRNB3,CHRNA6,THAP1,RNF170,RNF170,MIR4469,HOOK3,FNTA,POMK,HGSNAT,POTEA | -0.613758 | 201.761 | 175 | 99.9073 |
| 2 | 13 | 22097333 | 25352565 | MICU2,FGF9,SGCG,SACS,TNFRSF19,MIPEP,MIPEP,PCOTH,PCOTH,C1QTNF9B,C1QTNF9B,SPATA13,MIR2276,SPATA13,C1QTNF9,PARP4,TPTE2P6,ATP12A,RNF17 | -0.613998 | 193.243 | 264 | 154.534 |
| 2 | 7 | 44838798 | 44883036 | PPIA,H2AFV | -0.621162 | 235.845 | 15 | 7.45114 |

| | | | | | | | | |
|---|---|---|---|---|---|---|---|---|
| 2 | 19 | 52327814 | 54006347 | FPR3,ZNF577,ZNF649-AS1,ZNF649,ZNF613,ZNF350-AS1,ZNF350,ZNF615,ZNF614,ZNF432,ZNF841,ZNF616,ZNF836,PPP2R1A,ZNF766,ZNF766,MIR643,ZNF480,ZNF610,ZNF880,ZNF528-AS1,ZNF528,ZNF534,ZNF578,ZNF808,ZNF701,ZNF137P,ZNF83,ZNF611,ZNF600,ZNF28,ZNF468,ZNF320,ZNF888,ZNF321P,ZNF816-ZNF321P,ZNF816-ZNF321P,ZNF816,ERVV-1,ERVV-2,ZNF160,ZNF415,ZNF347,ZNF665,ZNF818P,ZNF677,VN1R2,VN1R4,FAM90A27P,BIRC8,ZNF845,ZNF525,ZNF765,ZNF765-ZNF761,ZNF765-ZNF761,ZNF761,ZNF813 | -0.622101 | 209.491 | 484 | 270.194 |
| 2 | 6 | 1.09E+08 | 121433831 | CCDC162P,C6orf183,CCDC162P,CD164,PPIL6,PPIL6,SMPD2,SMPD2,MICAL1,MICAL1,ZBTB24,ZBTB24,AK9,FIG4,GPR6,WASF1,CDC40,METTL24,DDO,SLC22A16,CDK19,CDK19,AMD1,AMD1,GTF3C6,RPF2,GSTM2P1,SLC16A10,MFSD4B,REV3L,REV3L,TRAF3IP2-AS1,TRAF3IP2-AS1,TRAF3IP2-AS1,TRAF3IP2,FYN,WISP3,TUBE1,FAM229B,LAMA4,LAMA4,LOC101927640,RFPL4B,MARCKS,HDAC2,HDAC2,HDAC2-AS2,HDAC2-AS2,HS3ST5,FRK,NT5DC1,NT5DC1,COL10A1,TSPYL4,DSE,DSE,TSPYL1,DSE,LOC100287467,CALHM6,TRAPPC3L,TRAPPC3L,CALHM5,TRAPPC3L,CALHM4,CALHM4,RWDD1,RSPH4A,ZUFSP,KPNA5,FAM162B,GPRC6A,RFX6,VGLL2,ROS1,DCBLD1,DCBLD1,GOPC,GOPC,NUS1,SLC35F1,CEP85L,CEP85L,PLN,MCM9,MCM9,ASF1A,FAM184A,FAM184A,MIR548B,MAN1A1,TBC1D32 | -0.623648 | 200.275 | 972 | 558.764 |
| 2 | 18 | 14105691 | 14763934 | ZNF519,ANKRD20A5P,POTEC,ANKRD30B | -0.63558 | 201.105 | 30 | 13.1132 |
| 2 | X | 1.14E+08 | 117511795 | LRCH2,RBMXL3,LRCH2,LUZP4,PLS3,AGTR2,SLC6A14,CT83,KLHL13,WDR44 | -0.635764 | 202.943 | 105 | 60.3937 |
| 2 | 15 | 49936316 | 50730839 | ATP8B4,SLC27A2,HDC,GABPB1,GABPB1,GABPB1-IT1,GABPB1-AS1,MIR4712 | -0.637213 | 208.708 | 90 | 51.6236 |
| 2 | 17 | 67310452 | 70118047 | ABCA5,MAP2K6,KCNJ16,KCNJ2,SOX9 | -0.640119 | 164.439 | 52 | 31.3013 |
| 2 | 5 | 76357559 | 86626118 | AGGF1,ZBED3,ZBED3,SNORA47,PDE8B,WDR41,OTP,TBCA,AP3B1,SCAMP1,LHFPL2,ARSB,DMGDH,BHMT2,BHMT,JMY,HOMER1,PAPD4,CMYA5,MTX3,THBS4,THBS4,CTD-2201I18.1,SERINC5,LOC644936,SPZ1,ZFYVE16,FAM151B,ANKRD34B,DHFR,DHFR,MTRNR2L2,DHFR,MSH3,MSH3,RASGRF2-AS1,RASGRF2,RASGRF2,RASGRF2,RNU5E-1,RNU5D-1,RNU5E-1,RNU5D-1,CKMT2,CKMT2-AS1,RNU5E-1,RNU5D-1,ZCCHC9,RNU5E-1,RNU5D-1,ACOT12,RNU5E-1,RNU5D-1,SSBP2,SSBP2,ATG10,RPS23,ATP6AP1L,MIR3977,TMEM167A,TMEM167A,SCARNA18,TMEM167A,XRCC4,XRCC4,VCAN,VCAN,VCAN-AS1,HAPLN1,EDIL3,NBPF22P,COX7C,COX7C,SNORD138,MIR4280,LOC101929380,LINC01949,RASA1 | -0.644044 | 177.321 | 710 | 412.083 |
| 2 | 16 | 34267204 | 35193080 | - | -0.645924 | 178.885 | 34 | 16.4804 |

| | | | | | | | | |
|---|---|---|---|---|---|---|---|---|
| 2 | 7 | 6825551 | 26894542 | RSPH10B,RSPH10B2,CCZ1B,MIR3683,LOC100131257,C1GALT1,COL28A1,MIOS,RPA3,RPA3,UMAD1,UMAD1,GLCCI1,ICA1,NXPH1,NDUFA4,PHF14,THSD7A,TMEM106B,VWDE,SCIN,ARL4A,ETV1,DGKB,AGMO,MEOX2,ISPD,ISPD,ISPD-AS1,SOSTDC1,LRRC72,ANKMY2,BZW2,TSPAN13,AGR2,AGR3,AHR,SNX13,PRPS1L1,HDAC9,MIR1302-6,HDAC9,TWIST1,FERD3L,TWISTNB,TWISTNB,MIR3146,TMEM196,MACC1,ITGB8,ABCB5,SP8,SP4,SP4,MIR1183,DNAH11,DNAH11,CDCA7L,CDCA7L,RAPGEF5,STEAP1B,LOC541472,IL6,IL6,TOMM7,SNHG26,SNORD93,FAM126A,KLHL7,NUPL2,GPNMB,MALSU1,IGF2BP3,RPS2P32,TRA2A,CLK2P1,CCDC126,FAM221A,STK31,NPY,MPP6,GSDME,OSBPL3,CYCS,C7orf31,NPVF,MIR148A,NFE2L3,HNRNPA2B1,CBX3,SNX10,SNX10,LOC105375304,LOC441204,KIAA0087,C7orf71,SKAP2 | -0.648122 | 187.297 | 1225 | 697.204 |
| 2 | 15 | 42481239 | 42966266 | VPS39,VPS39,MIR627,TMEM87A,GANC,CAPN3,ZNF106,SNAP23,LRRC57,HAUS2,STARD9 | -0.651948 | 190.193 | 173 | 106.104 |
| 2 | 13 | 32930568 | 39260931 | BRCA2,N4BP2L1,N4BP2L2,PDS5B,KL,STARD13,RFC3,NBEA,NBEA,MAB21L1,DCLK1,SOHLH2,CCDC169-SOHLH2,CCDC169-SOHLH2,CCDC169,SPART,SPART,SPART-AS1,CCNA1,SERTM1,RFXAP,SMAD9,ALG5,EXOSC8,SUPT20H,CSNK1A1L,POSTN,TRPC4,UFM1 | -0.654979 | 192.628 | 459 | 268.093 |
| 2 | 17 | 29226160 | 31203240 | TEFM,ADAP2,RNF135,LOC646030,MIR4733,NF1,NF1,OMG,NF1,EVI2B,NF1,EVI2A,RAB11FIP4,RAB11FIP4,MIR4724,MIR193A,MIR4725,MIR365B,COPRS,UTP6,SUZ12,LRRC37B,LRRC37B,SH3GL1P1,LOC105371730,LOC105371730,RHOT1,RHOT1,RHBDL3,C17orf75,MIR632,ZNF207,ZNF207,PSMD11,CDK5R1,MYO1D | -0.656516 | 200.468 | 351 | 196.881 |
| 2 | 20 | 68259 | 240037 | DEFB125,DEFB126,DEFB127,DEFB128,DEFB129,DEFB132 | -0.660266 | 191.197 | 19 | 11.2868 |
| 2 | 7 | 77267891 | 81593648 | PTPN12,APTR,RSBN1L,RSBN1L,TMEM60,PHTF2,MAGI2,MAGI2,MAGI2-AS3,GNAI1,GNAT3,CD36,SEMA3C,HGF,CACNA2D1 | -0.661325 | 173.409 | 188 | 108.275 |
| 2 | 7 | 48320920 | 50179918 | ABCA13,CDC14C,VWC2,ZPBP,SPATA48 | -0.662903 | 182.517 | 116 | 64.1502 |
| 2 | 15 | 76146675 | 77239955 | UBE2Q2,FBXO22,FBXO22,FBXO22-AS1,NRG4,TMEM266,TMEM266,LOC101929439,ETFA,ISL2,SCAPER,SCAPER,MIR3713,RCN2 | -0.663187 | 189.805 | 136 | 72.934 |
| 2 | 7 | 31569452 | 34981501 | CCDC129,PPP1R17,PDE1C,LSM5,AVL9,AVL9,DPY19L1P1,DPY19L1P1,MIR550A2,MIR550B2,DPY19L1P2,KBTBD2,RP9P,FKBP9,NT5C3A,RP9,BBS9,BMPER,NPSR1-AS1,NPSR1-AS1,NPSR1,NPSR1,NPSR1-AS1,NPSR1,DPY19L1 | -0.663497 | 199.682 | 258 | 143.039 |
| 2 | 17 | 27993823 | 29161430 | SSH2,SSH2,EFCAB5,EFCAB5,NSRP1,MIR423,MIR3184,NSRP1,SLC6A4,BLMH,TMIGD1,CPD,GOSR1,TBC1D29,LOC107133515,SH3GL1P2,LOC107133515,SUZ12P1,SUZ12P1,CRLF3,CRLF3,ATAD5 | -0.664862 | 190.679 | 189 | 106.645 |
| 2 | 7 | 61657308 | 65139352 | ZNF733P,LOC102724738,LOC100287704,LOC100287834,LOC100287834,MIR4283-1,MIR4283-2,ZNF727,ZNF735,ZNF679,ZNF736,ZNF680,ZNF107,ZNF138,ZNF273,ZNF117,ERV3-1-ZNF117,ERV3-1-ZNF117,ERV3-1,CCT6P3,SNORA22C,CCT6P3,INTS4P1,ZNF92,LOC441242,INTS4P2 | -0.665551 | 164.242 | 177 | 79.8197 |

| | | | | | | | | |
|---|---|---|---|---|---|---|---|---|
| 2 | 2 | 2.01E+08 | 202319063 | AOX1,BZW1,CLK1,PPIL3,NIF3L1,ORC2,FAM126B,NDUFB3,CFLAR,CFLAR,CFLAR-AS1,CASP10,CASP8,ALS2CR12,TRAK2 | -0.66563 | 187.725 | 201 | 121.507 |
| 2 | 4 | 10529670 | 26585947 | CLNK,HS3ST1,RAB28,LINC01097,NKX3-2,LINC01096,BOD1L1,MIR5091,CPEB2-AS1,CPEB2,CPEB2,LOC101929095,LOC101929095,C1QTNF7,C1QTNF7,CC2D2A,FBXL5,FAM200B,BST1,CD38,FGFBP1,FGFBP2,PROM1,TAPT1,TAPT1-AS1,LDB2,QDPR,CLRN2,LAP3,MED28,FAM184B,DCAF16,NCAPG,NCAPG,LCORL,LCORL,SLIT2,SLIT2,MIR218-1,PACRGL,PACRGL,KCNIP4,KCNIP4,ADGRA3,GBA3,PPARGC1A,MIR573,DHX15,SOD3,CCDC149,LGI2,SEPSECS,PI4K2B,ZCCHC4,ANAPC4,SLC34A2,SEL1L3,SMIM20,RBPJ,CCKAR,TBC1D19 | -0.666626 | 174.479 | 849 | 498.549 |
| 2 | 11 | 79442211 | 89896862 | MIR4300HG,MIR4300,FAM181B,PRCP,DDIAS,RAB30,RAB30,SNORA70E,PCF11,PCF11,LOC100506282,ANKRD42,CCDC90B,DLG2,TMEM126B,TMEM126A,CREBZF,CCDC89,SYTL2,CCDC83,PICALM,EED,EED,MIR6755,HIKESHI,CCDC81,ME3,PRSS23,PRSS23,FZD4,FZD4,TMEM135,RAB38,MIR3166,CTSC,GRM5-AS1,GRM5,GRM5,TYR,NOX4,FOLH1B,TRIM77,TRIM49,TRIM64B,TRIM64,TRIM49C,UBTFL1,NAALAD2 | -0.669582 | 190.141 | 578 | 329.105 |
| 2 | 8 | 77912737 | 80522840 | PKIA,ZC2HC1A,IL7 | -0.675969 | 134.603 | 39 | 21.7697 |
| 2 | 9 | 1.35E+08 | 135266209 | SETX,TTF1 | -0.677922 | 196.842 | 45 | 28.7478 |
| 2 | 8 | 82196190 | 96070187 | FABP5,PMP2,FABP9,FABP4,FABP12,IMPA1,SLC10A5,ZFAND1,CHMP4C,SNX16,RALYL,LRRCC1,E2F5,E2F5,C8orf59,C8orf59,CA13,CA1,CA3,CA3,CA3-AS1,CA3-AS1,CA2,CA2,PSKH2,ATP6V0D2,SLC7A13,WWP1,RMDN1,CPNE3,CNGB3,CNBD1,DCAF4L2,MMP16,RIPK2,OSGIN2,NBN,DECR1,CALB1,TMEM64,NECAB1,NECAB1,C8orf88,C8orf88,PIP4P2,OTUD6B,LRRC69,LRRC69,MIR4661,LRRC69,SLC26A7,SLC26A7,RUNX1T1,TRIQK,C8orf87,LINC00535,FAM92A,RBM12B,RBM12B,RBM12B-AS1,TMEM67,MIR378D2,PDP1,CDH17,GEM,RAD54B,RAD54B,FSBP,VIRMA,LOC100288748,ESRP1,DPY19L4,INTS8,INTS8,CCNE2,CCNE2,NDUFAF6,TP53INP1,NDUFAF6 | -0.68018 | 202.66 | 835 | 473.581 |
| 2 | 10 | 1.05E+08 | 105152287 | NT5C2,RPEL1,INA,PCGF6,TAF5,ATP5MD | -0.680607 | 189.425 | 67 | 37.3818 |
| 2 | 17 | 56635079 | 60683686 | TEX14,RAD51C,PPM1E,PPM1E,TRIM37,TRIM37,SKA2,SKA2,MIR454,SKA2,MIR301A,PRR11,SMG8,GDPD1,YPEL2,YPEL2,MIR4729,DHX40,CLTC,PTRH2,VMP1,VMP1,MIR21,TUBD1,RPS6KB1,RNFT1,RNFT1,TBC1D3P1-DHX40P1,TBC1D3P1-DHX40P1,RNFT1-DT,TBC1D3P1-DHX40P1,MIR4737,HEATR6,HEATR6,LOC653653,CA4,USP32,USP32,SCARNA20,C17orf64,APPBP2,PPM1D,BCAS3,TBX2,C17orf82,TBX4,NACA2,BRIP1,INTS2,MED13,TBC1D3P2,EFCAB3,METTL2A,TLK2 | -0.681261 | 205.608 | 664 | 377.254 |

| | | | | | | | | |
|---|---|---|---|---|---|---|---|---|
| 2 | 3 | 93519633 | 108247504 | PROS1,ARL13B,ARL13B,STX19,DHFR2,DHFR2,NSUN3,NSUN3,EPHA6,ARL6,CRYBG3,CRYBG3,RIOX2,RIOX2,GABRR3,OR5AC2,OR5H1,OR5H14,OR5H15,OR5H6,OR5H2,OR5K4,OR5K3,OR5K1,OR5K2,CLDND1,GPR15,CPOX,ST3GAL6,DCBLD2,MIR548G,COL8A1,MIR548G,HP09053,CMSS1,MIR548G,CMSS1,MIR548G,CMSS1,FILIP1L,MIR548G,CMSS1,FILIP1L,MIR3921,CMSS1,TMEM30CP,TBC1D23,NIT2,TOMM70,LNP1,TMEM45A,ADGRG7,TFG,ABI3BP,IMPG2,SENP7,FAM172BP,TRMT10C,PCNP,ZBTB11,ZBTB11,ZBTB11-AS1,RPL24,CEP97,NXPE3,NFKBIZ,ZPLD1,ALCAM,CBLB,CCDC54,BBX,LINC00636,CD47,IFT57,HHLA2,MYH15 | -0.687582 | 181.113 | 884 | 516.337 |
| 2 | 12 | 64895039 | 71950516 | TBK1,RASSF3,RASSF3,MIR548C,MIR548Z,GNS,TBC1D30,WIF1,LEMD3,MSRB3,LOC100507065,RPSAP52,RPSAP52,HMGA2,HMGA2,HMGA2,LOC100129940,LLPH,TMBIM4,IRAK3,HELB,GRIP1,CAND1,DYRK2,IFNG,IL26,IL22,MDM1,RAP1B,SNORA70G,RAP1B,NUP107,SLC35E3,MDM2,CPM,CPSF6,CPSF6,MIR1279,LYZ,YEATS4,FRS2,CCT2,LRRC10,BEST3,RAB3IP,MYRFL,CNOT2,KCNMB4,PTPRB,PTPRR,TSPAN8,LGR5 | -0.689417 | 181.94 | 606 | 354.926 |
| 2 | 3 | 17299910 | 37407624 | TBC1D5,LOC339862,SATB1,KCNH8,KCNH8,MIR4791,EFHB,RAB5A,RAB5A,PP2D1,PP2D1,KAT2B,KAT2B,MIR3135A,SGO1,SGO1,SGO1-AS1,ZNF385D,ZNF385D,ZNF385D-AS1,UBE2E2,UBE2E2,MIR548AC,UBE2E1,NKIRAS1,RPL15,NR1D2,THRB,THRB,LOC101927854,MIR4792,RARB,TOP2B,MIR4442,NGLY1,OXSM,LINC00692,LRRC3B,NEK10,SLC4A7,EOMES,CMC1,AZI2,ZCWPW2,RBMS3,TGFBR2,GADL1,MIR466,STT3B,OSBPL10,ZNF860,GPD1L,CMTM8,CMTM7,CMTM6,DYNC1LI1,CNOT10,TRIM71,CCR4,GLB1,GLB1,TMPPE,CRTAP,SUSD5,FBXL2,FBXL2,UBP1,UBP1,CLASP2,PDCD6IP,ARPP21,ARPP21,MIR128-2,STAC,DCLK3,TRANK1,EPM2AIP1,MLH1,LRRFIP2,LOC152048,GOLGA4,GOLGA4 | -0.692047 | 177.359 | 1105 | 644.001 |
| 2 | 15 | 78732130 | 78885616 | IREB2,HYKK,PSMA4,CHRNA5,CHRNA5,CHRNA3 | -0.692057 | 182.037 | 46 | 28.5747 |
| 2 | 2 | 1.91E+08 | 197711887 | HIBCH,INPP1,MFSD6,NEMP2,NAB1,GLS,STAT1,LOC105373805,STAT4,STAT4,MYO1B,NABP1,CAVIN2,TMEFF2,SLC39A10,DNAH7,STK17B,HECW2,CCDC150,CCDC150,LOC100130452,GTF3C3,C2orf66,PGAP1 | -0.694193 | 186.041 | 429 | 256.021 |
| 2 | X | 1.5E+08 | 150844499 | MTM1,MTMR1,CD99L2,HMGB3,MIR4330,GPR50-AS1,GPR50,GPR50,VMA21,PASD1 | -0.694517 | 185.572 | 94 | 55.3468 |
| 2 | 3 | 1.08E+08 | 111870774 | DZIP3,RETNLB,TRAT1,GUCA1C,MORC1,MORC1,MORC1-AS1,C3orf85,DPPA2,DPPA4,LINC01205,NECTIN3,CD96,CD96,ZBED2,PLCXD2,PLCXD2,PHLDB2,PHLDB2,ABHD10,TAGLN3,TMPRSS7,C3orf52,C3orf52,MIR567,GCSAM,SLC9C1 | -0.695088 | 174.845 | 252 | 148.831 |
| 2 | 22 | 46088812 | 46239597 | ATXN10,ATXN10,MIR4762 | -0.699809 | 180.128 | 23 | 12.2863 |

| | | | | | | | | |
|---|---|---|---|---|---|---|---|---|
| 2 | 5 | 54718653 | 61642392 | MTREX,MTREX,PLPP1,PLPP1,PLPP1,MIR5687,PLPP1,RNF138P1,SLC38A9,DDX4,IL31RA,IL6ST,FLJ31104,ANKRD55,LINC01948,C5orf67,MAP3K1,SETD9,SETD9,MIER3,MIER3,GPBP1,ACTBL2,PLK2,GAPT,MIR548AE2,LINC02108,RAB3C,PDE4D,DEPDC1B,ELOVL7,ERCC8,NDUFAF2,SMIM15,ZSWIM6,C5orf64,C5orf64,LOC101928651,KIF2A | -0.700466 | 165.141 | 409 | 229.587 |
| 2 | 18 | 48452141 | 66344471 | ME2,ELAC1,SMAD4,MEX3C,LINC01630,DCC,DCC,MIR4528,MBD2,MBD2,SNORA37,POLI,STARD6,C18orf54,DYNAP,RAB27B,CCDC68,TCF4,TCF4,TCF4-AS1,TCF4,TCF4-AS1,MIR4529,LOC642484,LOC642484,LINC01905,TXNL1,WDR7,BOD1L2,ST8SIA3,ONECUT2,FECH,NARS,LOC100505549,LOC100505549,ATP8B1,ATP8B1,NEDD4L,MIR122,MIR3591,ALPK2,LOC101927322,MALT1,MALT1,ZNF532,OACYLP,SEC11C,GRP,RAX,CPLX4,LMAN1,CCBE1,PMAIP1,MC4R,CDH20,RNF152,PIGN,KIAA1468,TNFRSF11A,ZCCHC2,PHLPP1,BCL2,KDSR,VPS4B,SERPINB5,SERPINB12,SERPINB13,SERPINB4,SERPINB3,SERPINB11,SERPINB7,SERPINB2,SERPINB10,HMSD,SERPINB8,LINC00305,CDH7,CDH19,MIR5011,DSEL,TMX3 | -0.701651 | 186.345 | 949 | 555.864 |
| 2 | 6 | 1.24E+08 | 128812341 | TRDN,TRDN-AS1,TRDN,NKAIN2,RNF217-AS1,RNF217-AS1,RNF217,RNF217,TPD52L1,HDDC2,HEY2,NCOA7,NCOA7-AS1,NCOA7,HINT3,TRMT11,CENPW,RSPO3,RNF146,ECHDC1,KIAA0408,SOGA3,SOGA3,C6orf58,THEMIS,PTPRK,PTPRK,LOC101928140 | -0.703772 | 206.585 | 308 | 178.058 |
| 2 | 4 | 1.26E+08 | 128811372 | MIR2054,INTU,SLC25A31,HSPA4L,PLK4 | -0.704578 | 178.487 | 84 | 48.8754 |
| 2 | 8 | 13425939 | 18659944 | SGCZ,SGCZ,MIR383,TUSC3,MSR1,FGF20,MICU3,ZDHHC2,CNOT7,VPS37A,MTMR7,SLC7A2,PDGFRL,MTUS1,MTUS1,MIR548V,FGL1,PCM1,ASAH1,NAT1,NAT2,PSD3 | -0.707337 | 188.179 | 309 | 177.926 |
| 2 | 4 | 26744120 | 49632814 | TBC1D19,STIM2,MIR4275,PCDH7,ARAP2,LOC439933,DTHD1,MIR1255B1,MIR4801,NWD2,C4orf19,C4orf19,RELL1,RELL1,PGM2,TBC1D1,TBC1D1,PTTG2,KLF3,TLR10,TLR1,TLR6,FAM114A1,MIR574,FAM114A1,TMEM156,KLHL5,WDR19,RFC1,KLB,RPL9,LIAS,UGDH,UGDH-AS1,SMIM14,SMIM14,UBE2K,PDS5A,LOC344967,N4BP2,RHOH,CHRNA9,RBM47,RBM47,MIR4802,NSUN7,APBB2,UCHL1,LIMCH1,PHOX2B,TMEM33,DCAF4L1,SLC30A9,BEND4,SHISA3,ATP8A1,GRXCR1,KCTD8,YIPF7,GUF1,GNPDA2,GABRG1,GABRA2,COX7B2,GABRA4,GABRB1,COMMD8,ATP10D,CORIN,CORIN,LOC101927179,LOC101927179,NFXL1,NFXL1,NFXL1,LOC101927157,LOC101927157,CNGA1,NIPAL1,TXK,TEC,SLAIN2,SLC10A4,ZAR1,FRYL,OCIAD1,OCIAD1,OCIAD1-AS1,OCIAD2,CWH43 | -0.707917 | 184.61 | 1246 | 728.332 |

| | | | | | | | | |
|---|---|---|---|---|---|---|---|---|
| 2 | 2 | 1.54E+08 | 170336166 | PRPF40A,ARL6IP6,RPRM,GALNT13,GALNT13,LOC100144595,KCNJ3,NR4A2,GPD2,GALNT5,ERMN,CYTIP,ACVR1C,ACVR1,UPP2,CCDC148-AS1,CCDC148,CCDC148,PKP4,PKP4,PKP4-AS1,DAPL1,TANC1,TANC1,MIR6888,WDSUB1,BAZ2B,MARCH7,CD302,LY75-CD302,LY75-CD302,LY75,PLA2R1,ITGB6,RBMS1,TANK,TANK,LOC101929512,PSMD14,TBR1,AHCTF1P1,SLC4A10,DPP4,LOC101929532,GCG,LOC101929532,FAP,FAP,IFIH1,GCA,KCNH7,FIGN,GRB14,COBLL1,COBLL1,SNORA70F,COBLL1,LOC101929633,SLC38A11,SCN3A,SCN2A,CSRNP3,GALNT3,TTC21B,TTC21B,TTC21B-AS1,LOC102724058,SCN1A,SCN1A,LOC101929680,SCN9A,SCN9A,SCN7A,XIRP2,XIRP2,XIRP2-AS1,LOC105616981,B3GALT1,STK39,CERS6,CERS6,MIR4774,NOSTRIN,SPC25,G6PC2,ABCB11,DHRS9,LRP2,BBS5 | -0.708335 | 197.946 | 1317 | 783.083 |
| 2 | 6 | 53747682 | 58745455 | LRRC1,LOC101927189,MLIP,TINAG,FAM83B,HCRTR2,GFRAL,HMGCLL1,BMP5,COL21A1,DST,DST,LOC101930010,BEND6,KIAA1586,ZNF451,ZNF451,LOC101927211,BAG2,RAB23,PRIM2,PRIM2,MIR548U,LINC00680-GUSBP4,LINC00680 | -0.709737 | 203.093 | 451 | 270.671 |
| 2 | 19 | 32847521 | 33321720 | ZNF507,DPY19L3,PDCD5,ANKRD27,RGS9BP,NUDT19,TDRD12,SLC7A9 | -0.714101 | 200.822 | 114 | 64.9787 |
| 2 | 1 | 1.18E+08 | 118609527 | MAN1A2,FAM46C,GDAP2,WDR3,WDR3,SPAG17,SPAG17 | -0.718776 | 175.371 | 106 | 65.0569 |
| 2 | 10 | 93260241 | 94652600 | HECTD2-AS1,HECTD2,PPP1R3C,TNKS2,FGFBP3,BTAF1,CPEB3,MARCH5,IDE,KIF11,HHEX,EXOC6 | -0.729162 | 165.478 | 184 | 105.259 |
| 2 | 2 | 2.39E+08 | 238940980 | RAMP1,UBE2F,UBE2F-SCLY | -0.729518 | 114.97 | 24 | 11.7207 |
| 2 | 6 | 76640702 | 88300020 | IMPG1,HTR1B,IRAK1BP1,PHIP,HMGN3,HMGN3,HMGN3-AS1,LCA5,SH3BGRL2,LINC01621,ELOVL4,TTK,BCKDHB,FAM46A,IBTK,TPBG,UBE3D,DOPEY1,DOPEY1,PGM3,PGM3,RWDD2A,ME1,PRSS35,SNAP91,RIPPLY2,CYB5R4,MRAP2,CEP162,TBX18,NT5E,SNX14,SYNCRIP,SNHG5,SNORD50A,SNHG5,SNORD50B,HTR1E,CGA,ZNF292,GJB7,SMIM8,C6orf163,LINC01590,CFAP206,SLC35A1,RARS2,ORC3 | -0.732028 | 187.227 | 686 | 399.017 |
| 2 | 13 | 19020500 | 21949354 | TUBA3C,LOC101928697,TPTE2,MPHOSPH8,PSPC1,ZMYM5,ZMYM2,GJA3,GJB2,GJB6,CRYL1,CRYL1,MIR4499,IFT88,IL17D,EEF1AKMT1,XPO4,LATS2,SAP18,SKA3,SKA3,MRPL57,MRPL57,MIPEPP3,ZDHHC20 | -0.740472 | 183.981 | 268 | 147.85 |
| 2 | 15 | 45724204 | 49833377 | C15orf48,C15orf48,MIR147B,SLC30A4,SLC30A4,HMGN2P46,HMGN2P46,BLOC1S6,SQOR,SEMA6D,SLC24A5,SLC24A5,MYEF2,MYEF2,CTXN2,SLC12A1,DUT,FBN1,CEP152,SHC4,SHC4,EID1,SECISBP2L,COPS2,GALK2,GALK2,MIR4716,GALK2,FAM227B,FAM227B,FAM227B,FGF7 | -0.741221 | 191.12 | 355 | 212.492 |

| | | | | | | | | |
|---|---|---|---|---|---|---|---|---|
| 2 | 12 | 1.01E+08 | 105501674 | SCYL2,SLC17A8,NR1H4,GAS2L3,ANO4,SLC5A8,UTP20,ARL1,SPIC,MYBPC1,CHPT1,CHPT1,SYCP3,SYCP3,GNPTAB,DRAM1,WASHC3,NUP37,PARPBP,PARPBP,PMCH,IGF1,PAH,PAH,ASCL1,ASCL1,LOC101929058,C12orf42,STAB2,NT5DC3,TTC41P,HSP90B1,MIR3652,HSP90B1,C12orf73,TDG,GLT8D2,HCFC2,NFYB,TXNRD1,TXNRD1,EID3,CHST11,CHST11,MIR3922,SLC41A2,C12orf45,ALDH1L2,WASHC4 | -0.746164 | 186.49 | 683 | 404.062 |
| 2 | 2 | 71649970 | 71661993 | ZNF638 | -0.748681 | 176.035 | 13 | 9.08487 |
| 2 | 17 | 41201118 | 41342760 | BRCA1,NBR2,NBR1 | -0.751379 | 198.053 | 49 | 28.6395 |
| 2 | 5 | 1.15E+08 | 131080407 | CCDC112,FEM1C,TICAM2,TMED7-TICAM2,TMED7-TICAM2,LOC101927100,TMED7,TMED7-TICAM2,TMED7,CDO1,ATG12,ATG12,AP3S1,AP3S1,LVRN,ARL14EPL,COMMD10,SEMA6A,SEMA6A,SEMA6A-AS1,LINC02147,LINC02208,LINC02215,DTWD2,DMXL1,DMXL1,MIR5706,TNFAIP8,HSD17B4,FAM170A,PRR16,FTMT,SRFBP1,LOX,ZNF474,LOC100505841,SNCAIP,SNCAIP,MGC32805,LINC02201,SNX2,SNX24,PPIC,LOC105379152,PRDM6,PRDM6,CEP120,CSNK1G3,ZNF608,GRAMD2B,ALDH7A1,PHAX,TEX43,LMNB1,MARCH3,C5orf63,MEGF10,PRRC1,CTXN3,SLC12A2,FBN2,SLC27A6,ISOC1,ISOC1,MIR4633,MIR4460,ADAMTS19-AS1,ADAMTS19,ADAMTS19,KIAA1024L,CHSY3,HINT1,LYRM7,CDC42SE2,RAPGEF6,FNIP1 | -0.759839 | 177.638 | 910 | 532.205 |
| 2 | 10 | 73861973 | 73973100 | ASCC1 | -0.76448 | 162.826 | 17 | 9.54501 |
| 2 | 5 | 1.03E+08 | 114547711 | PPIP5K2,C5orf30,NUDT12,EFNA5,FBXL17,FER,PJA2,MAN2A1,LINC01848,TMEM232,TMEM232,MIR548F3,SLC25A46,TSLP,WDR36,CAMK4,STARD4,STARD4-AS1,STARD4-AS1,NREP,NREP,NREP,NREP-AS1,EPB41L4A,EPB41L4A,EPB41L4A-AS1,EPB41L4A,EPB41L4A-AS1,SNORA13,EPB41L4A,LOC101927023,EPB41L4A-AS2,APC,SRP19,SRP19,REEP5,REEP5,DCP2,MCC,MCC,TSSK1B,YTHDC2,KCNN2,KCNN2,LOC101927078,TRIM36 | -0.765829 | 161.556 | 519 | 296.995 |
| 2 | 2 | 48032717 | 54176405 | MSH6,FBXO11,FOXN2,PPP1R21,STON1-GTF2A1L,STON1-GTF2A1L,STON1,STON1-GTF2A1L,GTF2A1L,STON1-GTF2A1L,LHCGR,FSHR,NRXN1,MIR4431,ASB3,GPR75-ASB3,ASB3,GPR75-ASB3,CHAC2,GPR75-ASB3,ERLEC1,GPR75-ASB3,MIR3682,GPR75-ASB3,GPR75,PSME4 | -0.771432 | 167.681 | 312 | 181.609 |
| 2 | 15 | 44816844 | 45332704 | EIF3J-AS1,EIF3J,SPG11,PATL2,B2M,TRIM69,SORD2P,TERB2,SORD | -0.773585 | 180.594 | 112 | 64.4298 |
| 2 | 12 | 1.11E+08 | 111074318 | IFT81,ATP2A2,ANAPC7,ARPC3,GPN3,GPN3,FAM216A,FAM216A,VPS29,RAD9B,PPTC7,TCTN1 | -0.775256 | 210.054 | 124 | 73.7543 |
| 2 | 5 | 61898215 | 68485610 | IPO11,IPO11-LRRC70,HTR1A,RNF180,RGS7BP,SHISAL2B,SREK1IP1,CWC27,ADAMTS6,CENPK,PPWD1,TRIM23,TRAPPC13,SGTB,NLN,ERBIN,SREK1,MAST4,CD180,LINC02219,PIK3R1,SLC30A5,CCNB1,CENPH | -0.779443 | 175.922 | 408 | 236.017 |

| | | | | | | | | |
|---|---|---|---|---|---|---|---|---|
| 2 | 8 | 23702281 | 26223943 | STC1,ADAM28,ADAM28,LOC101929294,LOC101929294,ADAMDEC1,LOC101929294,ADAM7,LOC101929294,ADAM7,LOC101929315,LOC101929294,NEFM,NEFL,NEFL,MIR6841,DOCK5,DOCK5,MIR6876,GNRH1,KCTD9,CDCA2,EBF2,PPP2R2A | -0.780083 | 174.819 | 238 | 142.209 |
| 2 | 8 | 1.42E+08 | 141900129 | PTK2 | -0.780389 | 180.576 | 30 | 14.846 |
| 2 | 17 | 49063056 | 54587291 | SPAG9,NME1-NME2,NME1,NME1-NME2,NME2,MBTD1,UTP18,CA10,C17orf112,KIF2B,TOM1L1,TOM1L1,COX11,COX11,STXBP4,HLF,MMD,TMEM100,PCTP,ANKFN1 | -0.781186 | 173.602 | 244 | 139.908 |
| 2 | 1 | 86852573 | 91726281 | ODF2L,CLCA2,CLCA1,CLCA4,CLCA4,LOC105378828,LOC105378828,CLCA3P,SH3GLB1,SELENOF,HS2ST1,LINC01140,LMO4,PKN2-AS1,PKN2,PKN2,GTF2B,KYAT3,KYAT3,RBMXL1,GBP3,GBP1,GBP2,GBP7,GBP4,GBP5,GBP6,GBP1P1,LRRC8B,LRRC8C,LRRC8D,ZNF326,BARHL2,ZNF644 | -0.800338 | 186.707 | 370 | 216.985 |
| 2 | 10 | 1.02E+08 | 102040815 | ERLIN1,CHUK,CWF19L1,CWF19L1,SNORA12,BLOC1S2 | -0.801456 | 182.864 | 50 | 29.6192 |
| 2 | X | 1.47E+08 | 148582684 | FMR1,FMR1NB,AFF2,IDS | -0.80286 | 156.606 | 84 | 49.1261 |
| 2 | 12 | 97346425 | 100685521 | RMST,MIR1251,MIR135A2,MIR4495,MIR4303,SLC9A7P1,LINC02453,TMPO-AS1,TMPO,TMPO,SLC25A3,SLC25A3,SNORA53,IKBIP,APAF1,ANKS1B,ANKS1B,FAM71C,UHRF1BP1L,GOLGA2P5,ACTR6,ACTR6,DEPDC4,DEPDC4,SCYL2 | -0.809469 | 162.914 | 217 | 126.574 |
| 2 | 15 | 51250719 | 56657956 | AP4E1,MIR4713HG,TNFAIP8L3,MIR4713HG,CYP19A1,MIR4713HG,CYP19A1,MIR4713,GLDN,DMXL2,SCG3,LYSMD2,TMOD2,TMOD3,LEO1,MAPK6,BCL2L10,GNB5,GNB5,CERNA1,CERNA1,MYO5C,MYO5C,MYO5C,MIR1266,MYO5A,ARPP19,FAM214A,ONECUT1,WDR72,UNC13C,RSL24D1,RAB27A,PIGB,PIGB,CCPG1,DNAAF4-CCPG1,CCPG1,DNAAF4-CCPG1,CCPG1,DNAAF4-CCPG1,MIR628,DNAAF4-CCPG1,C15orf65,DNAAF4-CCPG1,C15orf65,DNAAF4,DNAAF4-CCPG1,DNAAF4,PYGO1,PRTG,NEDD4,RFX7,TEX9 | -0.813486 | 176.611 | 591 | 348.021 |
| 2 | 1 | 1.73E+08 | 173494116 | SUCO,FASLG,TNFSF18,TNFSF4,PRDX6,SLC9C2 | -0.819765 | 186.822 | 67 | 39.8556 |
| 2 | 12 | 88589908 | 96894734 | KITLG,DUSP6,POC1B,POC1B,GALNT4,POC1B-GALNT4,POC1B,POC1B-GALNT4,POC1B,POC1B-GALNT4,POC1B-AS1,ATP2B1,LINC00615,CCER1,EPYC,KERA,LUM,DCN,LINC01619,BTG1,CLLU1OS,CLLU1OS,CLLU1,C12orf74,PLEKHG7,EEA1,LOC643339,LOC643339,LINC02412,NUDT4,NUDT4P2,NUDT4B,UBE2N,MRPL42,SOCS2-AS1,SOCS2,SOCS2,CRADD,PLXNC1,CEP83,MIR5700,TMCC3,MIR492,MIR492,KRT19P2,NDUFA12,NR2C1,FGD6,VEZT,MIR331,MIR3685,METAP2,USP44,PGAM1P5,NTN4,NTN4,LOC105369921,LINC02410,SNRPF,CCDC38,AMDHD1,HAL,LTA4H,ELK3,CDK17,CFAP54 | -0.82164 | 170.285 | 591 | 341.63 |
| 2 | 14 | 80671895 | 89341485 | DIO2,DIO2,DIO2-AS1,CEP128,TSHR,GTF2A1,GTF2A1,SNORA79,STON2,SEL1L,LINC02301,FLRT2,LINC01148,GALC,GPR65,KCNK10,SPATA7,PTPN21,ZC3H14,EML5,TTC8 | -0.834414 | 169.29 | 364 | 213.38 |

| | | | | | | | |
|---|---|---|---|---|---|---|---|
| 2 | 3 | 1.41E+08 | 149087662 | RASA2,RNF7,GRK7,ATP1B3,TFDP2,GK5,XRN1,ATR,PLS1,TRPC1,PCOLCE2,PAQR9,U2SURP,CHST2,SLC9A9,C3orf58,PLOD2,PLSCR4,PLSCR2,PLSCR1,PLSCR5,ZIC4,ZIC1,AGTR1,CPB1,CPA3,GYG1,HLTF,HLTF,HLTF-AS1,HPS3,HPS3,CP,CP,TM4SF18,TM4SF1 | -0.835921 | 191.244 | 580 | 335.617 |
| 2 | 5 | 87524908 | 98194064 | TMEM161B,LINC00461,MIR9-2,LINC00461,MEF2C-AS2,MEF2C,MEF2C,MEF2C-AS1,MIR3660,CETN3,LOC731157,MBLAC2,POLR3G,LYSMD3,ADGRV1,ARRDC3,ARRDC3,ARRDC3-AS1,NR2F1,FAM172A,MIR2277,FAM172A,FAM172A,POU5F2,KIAA0825,SLF1,MCTP1,FAM81B,TTC37,ARSK,GPR150,RFESD,SPATA9,SPATA9,SPATA9,RHOBTB3,RHOBTB3,GLRX,LINC01554,ELL2,LOC101929710,MIR583,LOC101929710,PCSK1,LOC101929710,CAST,CAST,ERAP1,ERAP1,ERAP2,LNPEP,LIX1,RIOK2,RGMB,RGMB-AS1,RGMB,CHD1 | -0.836227 | 169.641 | 667 | 390.385 |
| 2 | 18 | 66382152 | 72021873 | TMX3,CCDC102B,DOK6,CD226,RTTN,SOCS6,CBLN2,NETO1,NETO1,MIR548AV,FBXO15,TIMM21,CYB5A,C18orf63 | -0.837212 | 151.784 | 200 | 115.135 |
| 2 | 14 | 60952291 | 64560254 | SIX6,SIX1,SIX4,MNAT1,TRMT5,TRMT5,SLC38A6,SLC38A6,TMEM30B,PRKCH,LINC01303,FLJ22447,FLJ22447,HIF1A-AS1,HIF1A,HIF1A,HIF1A,LOC105370526,HIF1A,LOC105370526,HIF1A-AS2,SNAPC1,SYT16,LINC00643,KCNH5,RHOJ,GPHB5,PPP2R5E,WDR89,SGPP1,SYNE2 | -0.838575 | 181.031 | 306 | 179.69 |
| 2 | 11 | 1.03E+08 | 110581736 | DYNC2H1,MIR4693,PDGFD,PDGFD,DDI1,CASP12,CASP4,CASP5,CASP1,CARD16,CARD17,CARD18,GRIA4,MSANTD4,KBTBD3,KBTBD3,AASDHPPT,AASDHPPT,GUCY1A2,CWF19L2,ALKBH8,ELMOD1,SLN,SLC35F2,RAB39A,CUL5,ACAT1,NPAT,ATM,ATM,C11orf65,C11orf65,KDELC2,EXPH5,DDX10,C11orf87,ZC3H12C,RDX,FDX1,ARHGAP20 | -0.840604 | 180.854 | 568 | 326.756 |
| 2 | 16 | 58756147 | 64981019 | GOT2,LOC729159,CDH8 | -0.841473 | 113.253 | 75 | 42.5089 |
| 2 | 16 | 75795282 | 77224584 | CNTNAP4,LINC02125,MIR4719 | -0.84299 | 140.355 | 44 | 24.2977 |
| 2 | 16 | 46457168 | 47697720 | ANKRD26P1,SHCBP1,VPS35,ORC6,MYLK3,C16orf87,GPT2,DNAJA2,NETO2,ITFG1-AS1,ITFG1,ITFG1,PHKB | -0.850281 | 203.344 | 197 | 110.469 |
| 2 | 20 | 20615995 | 21330101 | RALGAPA2,KIZ,KIZ,KIZ-AS1,XRN2 | -0.853116 | 183.009 | 52 | 30.1888 |
| 2 | 11 | 36616228 | 43923324 | C11orf74,LRRC4C,LOC100507205,API5,TTC17,MIR670,MIR129-2,HSD17B12,ALKBH3,ALKBH3,SEC14L1P1 | -0.856885 | 133.94 | 162 | 89.5692 |
| 2 | 20 | 35526822 | 35696573 | SAMHD1,RBL1 | -0.872828 | 175.6 | 34 | 21.4557 |
| 2 | 3 | 1.3E+08 | 132438694 | COL6A5,COL6A6,PIK3R4,ATP2C1,ATP2C1,ASTE1,ASTE1,NEK11,NEK11,LOC339874,NUDT16,MRPL3,MRPL3,SNORA58,CPNE4,CPNE4,MIR5704,ACPP,DNAJC13,ACAD11,NPHP3-ACAD11,ACAD11,NPHP3-ACAD11,ACKR4,ACAD11,NPHP3-ACAD11,UBA5,NPHP3-ACAD11,UBA5,NPHP3-ACAD11,NPHP3 | -0.874573 | 173.229 | 385 | 230.566 |
| 2 | 9 | 95048771 | 95272410 | IARS,IARS,MIR3651,SNORA84,NOL8,CENPP,CENPP,OGN,CENPP,OMD,CENPP,ASPN,CENPP,ECM2 | -0.879329 | 217.964 | 73 | 42.611 |
| 2 | 4 | 13322 | 466544 | ZNF595,ZNF718,ZNF718,ZNF732,ZNF141,ZNF141,MIR571,ABCA11P,ABCA11P,ZNF721 | -0.881768 | 227.42 | 70 | 36.434 |
| 2 | 1 | 1.11E+08 | 111730958 | CD53,LRIF1,DRAM2,CEPT1,DENND2D | -0.887778 | 164.368 | 45 | 26.288 |
| 2 | 13 | 54049415 | 58206174 | MIR1297,MIR5007 | -0.916399 | 6.63023 | 31 | 16.9273 |

| | | | | | | | | |
|---|---|---|---|---|---|---|---|---|
| 2 | 10 | 92503278 | 93220335 | HTR7,RPP30,ANKRD1,NUDT9P1,PCGF5,HECTD2-AS1,HECTD2 | -0.917634 | 186.097 | 53 | 31.6398 |
| 2 | 16 | 11794310 | 11991904 | TXNDC11,ZC3H7A,BCAR4,RSL1D1,GSPT1 | -0.918164 | 178.444 | 67 | 38.7374 |
| 2 | 4 | 68547794 | 70800093 | UBA6,UBA6-AS1,GNRHR,TMPRSS11D,TMPRSS11A,TMPRSS11F,LOC550113,TMPRSS11F,LOC550113,SYT14P1,TMPRSS11F,FTLP10,TMPRSS11BNL,TMPRSS11BNL,TMPRSS11B,YTHDC1,TMPRSS11E,UGT2B17,UGT2B15,UGT2B10,UGT2A3,UGT2B7,LOC105377267,UGT2B11,UGT2B28,UGT2B4,UGT2A2,UGT2A1,UGT2A1,SULT1B1,SULT1E1,CSN1S1 | -0.919681 | 194.126 | 226 | 116.657 |
| 2 | 5 | 1.33E+08 | 134133006 | SKP1,PPP2CA,PPP2CA,MIR3661,CDKL3,UBE2B,CDKN2AIPNL,JADE2,SAR1B,SEC24A,CAMLG,DDX46 | -0.920661 | 194.66 | 127 | 73.2538 |
| 2 | 15 | 60770053 | 62238089 | ICE2,RORA-AS1,RORA,RORA,VPS13C | -0.920992 | 157.875 | 81 | 46.8611 |
| 2 | 4 | 1.69E+08 | 177114721 | ANXA10,DDX60,DDX60L,PALLD,CBR4,SH3RF1,NEK1,CLCN3,HPF1,MFAP3L,AADAT,GALNTL6,LOC101930370,GALNT7,GALNT7,HMGB2,SAP30,SCRG1,HAND2,HAND2,HAND2-AS1,HAND2-AS1,FBXO8,CEP44,MIR4276,HPGD,GLRA3,ADAM29,GPM6A,GPM6A,LOC101928590,WDR17,SPATA4 | -0.926908 | 152.614 | 466 | 267.171 |
| 2 | 2 | 2.19E+08 | 219458358 | USP37,CNOT9 | -0.932475 | 203.251 | 35 | 20.2279 |
| 2 | 12 | 37857194 | 48104695 | ALG10B,CPNE8,KIF21A,ABCD2,C12orf40,C12orf40,SLC2A13,SLC2A13,LRRK2,MUC19,CNTN1,PDZRN4,GXYLT1,YAF2,ZCRB1,PPHLN1,PRICKLE1,ADAMTS20,PUS7L,IRAK4,TWF1,TMEM117,NELL2,DBX2,PLEKHA8P1,ANO6,LINC00938,ARID2,SCAF11,SLC38A1,SLC38A2,SLC38A4,AMIGO2,PCED1B,MIR4698,PCED1B,MIR4494,RPAP3,ENDOU | -0.932716 | 173.564 | 815 | 476.152 |
| 2 | 1 | 1.04E+08 | 109617735 | AMY2B,AMY2A,PRMT6,NTNG1,VAV3,VAV3,VAV3-AS1,SLC25A24,NBPF4,NBPF6,FAM102B,HENMT1,PRPF38B,FNDC7,STXBP3,AKNAD1,GPSM2,GPSM2,CLCC1,CLCC1,WDR47,TAF13 | -0.936065 | 169.896 | 260 | 150.305 |
| 2 | 18 | 10500 | 2796088 | ROCK1P1,USP14,THOC1,COLEC12,CETN1,CLUL1,CLUL1,TYMSOS,TYMSOS,TYMS,TYMS,TYMS,ENOSF1,ENOSF1,YES1,ADCYAP1,LINC00470,METTL4,NDC80,SMCHD1 | -0.949003 | 194.952 | 219 | 126.374 |
| 2 | 3 | 47103664 | 47168188 | SETD2 | -0.950447 | 185.015 | 34 | 22.4026 |
| 2 | 10 | 1.06E+08 | 105765834 | STN1,SLK | -0.952773 | 137.691 | 25 | 15.0584 |
| 2 | 16 | 53244226 | 53859313 | CHD9,LOC643802,RBL2,AKTIP,RPGRIP1L,FTO | -0.954707 | 180.286 | 122 | 72.7712 |
| 2 | 17 | 61021488 | 61428838 | MIR633,TANC2 | -0.956709 | 145.91 | 23 | 11.4841 |
| 2 | 11 | 94906359 | 102272457 | SESN3,SESN3,LOC100129203,FAM76B,CEP57,MTMR2,MAML2,MAML2,MIR1260B,CCDC82,JRKL,CNTN5,LOC100128386,ARHGAP42,ARHGAP42,PGR,PGR,PGR-AS1,TRPC6,TRPC6,MIR3920,ANGPTL5,ANGPTL5,CEP126,CEP126,C11orf70,YAP1,BIRC3,BIRC2,TMEM123 | -0.964292 | 161.231 | 329 | 188.901 |
| 2 | 1 | 1.84E+08 | 185892793 | TSEN15,C1orf21,EDEM3,FAM129A,RNF2,TRMT1L,SWT1,IVNS1ABP,HMCN1 | -0.966593 | 185.219 | 149 | 86.6129 |

| | | | | | | | | |
|---|---|---|---|---|---|---|---|---|
| 2 | 7 | 87533600 | 94252789 | DBF4,ADAM22,SRI,SRI,LOC102723885,STEAP4,ZNF804B,ZNF804B,TEX47,STEAP2-AS1,STEAP2-AS1,STEAP1,STEAP2,CFAP69,LOC101927446,GTPBP10,CLDN12,CDK14,FZD1,MTERF1,AKAP9,CYP51A1,CYP51A1-AS1,CYP51A1-AS1,LRRD1,KRIT1,ANKIB1,GATAD1,PEX1,RBM48,FAM133B,FAM133DP,CDK6,SAMD9,SAMD9L,HEPACAM2,VPS50,CALCR,CALCR,MIR653,CALCR,MIR489,MIR4652,TFPI2,GNGT1,GNG11,BET1,COL1A2,CASD1,SGCE | -0.981797 | 176.145 | 636 | 381.389 |
| 2 | 1 | 91870345 | 92697072 | HFM1,CDC7,TGFBR3,BRDT,EPHX4,BTBD8,KIAA1107,C1orf146 | -0.990145 | 158.613 | 110 | 63.5142 |
| 2 | 1 | 2.27E+08 | 227843566 | CDC42BPA,ZNF678 | -0.994609 | 164.746 | 51 | 28.0277 |
| 2 | 14 | 95571452 | 95598036 | DICER1 | -1.02121 | 180.823 | 18 | 11.7707 |
| 2 | 1 | 1.86E+08 | 200817711 | PRG4,TPR,TPR,ODR4,ODR4,OCLM,LOC102724919,PDC,PTGS2,PLA2G4A,BRINP3,RGS18,RGS21,RGS1,RGS13,RGS2,UCHL5,UCHL5,TROVE2,TROVE2,GLRX2,CDC73,CDC73,MIR1278,CDC73,B3GALT2,KCNT2,KCNT2,MIR4735,CFH,CFHR3,CFHR1,CFHR4,CFHR2,CFHR5,F13B,ASPM,ZBTB41,CRB1,DENND1B,C1orf53,LHX9,NEK7,ATP6V1G3,PTPRC,MIR181A1HG,MIR181B1,MIR181A1,NR5A2,LINC00862,ZNF281,KIF14,DDX59,CAMSAP2 | -1.03004 | 170.082 | 727 | 418.97 |
| 2 | 2 | 55920195 | 62065848 | PNPT1,EFEMP1,MIR217HG,MIR217,MIR217HG,MIR216A,MIR217HG,MIR216B,LOC100129434,CCDC85A,CCDC85A,VRK2,VRK2,FANCL,FANCL,MIR4432HG,MIR4432,BCL11A,PAPOLG,REL,PUS10,PUS10,PEX13,PEX13,KIAA1841,C2orf74,AHSA2P,AHSA2P,USP34,USP34,USP34,SNORA70B,XPO1,FAM161A | -1.04058 | 152.793 | 329 | 193.102 |
| 2 | 9 | 1.24E+08 | 123955794 | C5,CNTRL,RAB14 | -1.04351 | 160.714 | 82 | 50.1853 |
| 2 | X | 1.39E+08 | 138901668 | MCF2,ATP11C | -1.04459 | 133.69 | 41 | 23.4844 |
| 2 | 1 | 2.04E+08 | 203839001 | ZC3H11A,ZBED6,ZC3H11A,SNRPE | -1.04924 | 197.459 | 29 | 15.4277 |
| 2 | 15 | 91290519 | 91346925 | BLM | -1.05046 | 156.536 | 23 | 14.6756 |
| 2 | 1 | 94643153 | 104068838 | ARHGAP29,ABCD3,F3,SLC44A3-AS1,MIR378G,SLC44A3,CNN3,LOC729970,ALG14,ALG14,LOC101928098,TMEM56,TMEM56-RWDD3,TMEM56,TMEM56-RWDD3,LOC101928118,TMEM56-RWDD3,LOC101928118,TMEM56-RWDD3,RWDD3,RWDD3,PTBP2,DPYD,DPYD,DPYD-AS1,MIR137HG,MIR137HG,MIR2682,MIR137HG,MIR137,SNX7,PLPPR5,PLPPR5,LOC100129620,PLPPR4,PALMD,PALMD,MIR548AA1,MIR548AA1,FRRS1,FRRS1,AGL,SLC35A3,MFSD14A,SASS6,TRMT13,TRMT13,LRRC39,LRRC39,DBT,RTCA,RTCA,MIR553,CDC14A,GPR88,VCAM1,EXTL2,SLC30A7,DPH5,S1PR1,OLFM3,COL11A1,RNPC3 | -1.06051 | 154.084 | 568 | 325.888 |
| 2 | 13 | 1.03E+08 | 103486933 | CCDC168,TEX30,KDELC1,BIVM,BIVM-ERCC5 | -1.06064 | 185.248 | 76 | 50.0227 |
| 2 | 1 | 35452708 | 35879754 | ZMYM6,ZMYM1,SFPQ,ZMYM4,ZMYM4,ZMYM4-AS1 | -1.06089 | 180.665 | 93 | 56.078 |
| 2 | 15 | 35152234 | 38765848 | AQR,ZNF770,DPH6,MIR3942,DPH6,MIR4510,C15orf41,C15orf41,CSNK1A1P1,CSNK1A1P1,LOC145845,MEIS2,TMCO5A,SPRED1,FAM98B | -1.0716 | 148.091 | 163 | 90.954 |
| 2 | 2 | 1.77E+08 | 178284488 | MTX2,MIR1246,HNRNPA3,NFE2L2,NFE2L2,MIR3128,AGPS | -1.07665 | 133.103 | 43 | 23.8621 |
| 2 | 5 | 1.32E+08 | 132070184 | IL5,RAD50,RAD50,TH2LCRR,IL13,IL4,IL4,LOC105379176,KIF3A | -1.08466 | 161.164 | 61 | 36.598 |

| | | | | | | | | |
|---|---|---|---|---|---|---|---|---|
| 2 | 4 | 4425136 | 4473501 | STX18 | -1.08593 | 161.693 | 17 | 8.55645 |
| 2 | 14 | 26918692 | 50318601 | NOVA1,MIR4307HG,MIR4307HG,MIR4307,FOXG1,LINC01551,PRKD1,G2E3-AS1,G2E3,G2E3,SCFD1,COCH,COCH,LOC100506071,STRN3,STRN3,MIR624,STRN3,AP4S1,AP4S1,HECTD1,HEATR5A,LOC101927124,LOC101927124,DTD2,DTD2,GPR33,NUBPL,LINC02313,ARHGAP5,AKAP6,NPAS3,EGLN3,SPTSSA,EAPP,SNX6,CFL2,BAZ1A,SRP54,FAM177A1,FAM177A1,LOC101927178,LOC101927178,PPP2R3C,PPP2R3C,KIAA0391,PSMA6,NFKBIA,INSM2,RALGAPA1,RALGAPA1P1,RALGAPA1,RALGAPA1P1,SNORA101B,BRMS1L,MBIP,SFTA3,SFTA3,NKX2-1,SFTA3,NKX2-1,NKX2-1-AS1,NKX2-1,NKX2-1-AS1,NKX2-8,PAX9,SLC25A21,SLC25A21,SLC25A21-AS1,MIPOL1,FOXA1,TTC6,SSTR1,CLEC14A,SEC23A,GEMIN2,TRAPPC6B,PNN,MIA2,FBXO33,LRFN5,FSCB,C14orf28,LOC101927418,KLHL28,TOGARAM1,PRPF39,PRPF39,SNORD127,FKBP3,FANCM,MIS18BP1,RPL10L,MDGA2,MIR548Y,RPS29,RPS29,LRR1,LRR1,RPL36AL,MGAT2,DNAAF2,POLE2,KLHDC1,KLHDC2,NEMF | -1.08853 | 160.244 | 1290 | 743.972 |
| 2 | 2 | 74362739 | 74414895 | BOLA3,MOB1A | -1.08904 | 134.072 | 13 | 6.98479 |
| 2 | 1 | 62907116 | 86808869 | USP1,DOCK7,DOCK7,ANGPTL3,ATG4C,FOXD3-AS1,FOXD3,ALG6,ITGB3BP,EFCAB7,EFCAB7,DLEU2L,PGM1,ROR1,UBE2U,CACHD1,CACHD1,MIR4794,RAVER2,JAK1,JAK1,MIR3671,JAK1,MIR101-1,AK4,DNAJC6,LEPROT,LEPR,LEPR,PDE4B,SGIP1,SGIP1,MIR3117,TCTEX1D1,INSL5,WDR78,MIER1,SLC35D1,C1orf141,IL23R,IL12RB2,SERBP1,GADD45A,GNG12,GNG12-AS1,DIRAS3,GNG12-AS1,WLS,GNG12-AS1,WLS,MIR1262,WLS,RPE65,DEPDC1,DEPDC1,DEPDC1-AS1,LRRC7,LRRC40,SRSF11,ANKRD13C,HHLA3,CTH,PTGER3,PTGER3,ZRANB2-AS1,ZRANB2-AS1,ZRANB2,ZRANB2,MIR186,ZRANB2,NEGR1,LRRIQ3,FPGT,FPGT-TNNI3K,FPGT-TNNI3K,FPGT-TNNI3K,TNNI3K,ERICH3,ERICH3,ERICH3-AS1,CRYZ,CRYZ,TYW3,TYW3,LHX8,SLC44A5,ACADM,RABGGTB,RABGGTB,SNORD45C,RABGGTB,SNORD45A,RABGGTB,SNORD45B,MSH4,ASB17,ST6GALNAC3,ST6GALNAC5,PIGK,AK5,ZZZ3,USP33,MIGA1,NEXN,FUBP1,DNAJB4,GIPC2,PTGFR,IFI44L,IFI44,ADGRL4,ADGRL2,TTLL7,PRKACB,SAMD13,DNASE2B,RPF1,GNG5,SPATA1,SPATA1,CTBS,CTBS,LINC01555,SSX2IP,LPAR3,MCOLN2,MCOLN3,WDR63,MIR4423,SYDE2,C1orf52,BCL10,BCL10,LOC646626,DDAH1,CYR61,ZNHIT6,COL24A1 | -1.11002 | 154.349 | 1648 | 945.716 |
| 2 | 1 | 47748006 | 47842557 | STIL,CMPK1 | -1.12032 | 123.898 | 21 | 11.2922 |
| 2 | 3 | 44794731 | 44894332 | KIAA1143,KIF15 | -1.12494 | 166.507 | 45 | 26.7265 |
| 2 | 19 | 40519591 | 40595340 | ZNF546,ZNF780B,ZNF780A | -1.12617 | 178.348 | 40 | 23.8502 |
| 2 | 4 | 52660617 | 52765641 | DCUN1D4 | -1.139 | 129.171 | 17 | 8.07539 |
| 2 | 1 | 1.8E+08 | 180771993 | ACBD6,XPR1 | -1.14044 | 125.546 | 18 | 9.31955 |

| | | | | | | | |
|---|---|---|---|---|---|---|---|
| 2 | 19 | 19905347 | 24364012 | ZNF56,ZNF506,ZNF56,ZNF253,ZNF93,ZNF682,ZNF90,ZNF486,MIR1270,ZNF826P,MIR1270,ZNF737,ZNF626,ZNF66,ZNF85,ZNF430,ZNF714,ZNF431,ZNF708,ZNF738,ZNF493,ZNF429,LOC400682,ZNF100,ZNF100,LOC641367,ZNF43,ZNF208,ZNF257,ZNF676,ZNF729,ZNF98,GOLGA2P9,ZNF492,ZNF99,ZNF728,ZNF730,ZNF724,IPO5P1,ZNF91,ZNF675,ZNF681,ZNF726,ZNF254,HAVCR1P1 | -1.1436 | 153.472 | 455 | 202.649 |
| 2 | 11 | 92881756 | 93493068 | SLC36A4,DEUP1,SMCO4,CEP295,CEP295,SCARNA9,CEP295,TAF1D,TAF1D,SNORA25,TAF1D,SNORA32,TAF1D,SNORD6,TAF1D,SNORA1,TAF1D,SNORA8,TAF1D,SNORD5,TAF1D,SNORA18,TAF1D,TAF1D,SNORA40,C11orf54 | -1.14476 | 159.485 | 102 | 61.6347 |
| 2 | 2 | 26587060 | 26624332 | SELENOI | -1.14566 | 120.784 | 10 | 6.04176 |
| 2 | 16 | 8994451 | 9015236 | USP7 | -1.14726 | 154.261 | 21 | 11.4146 |
| 2 | X | 76778701 | 86772329 | ATRX,MAGT1,COX7B,ATP7A,ATP7A,PGAM4,PGK1,TAF9B,CYSLTR1,RTL3,LPAR4,MIR4328,P2RY10,GPR174,ITM2A,TBX22,FAM46D,BRWD3,HMGN5,SH3BGRL,POU3F4,CYLC1,RPS6KA6,MIR548I4,HDX,UBE2DNL,APOOL,APOOL,SATL1,SATL1,ZNF711,POF1B,MIR1321,CHM,CHM,MIR361,DACH2 | -1.15139 | 138.7 | 461 | 261.764 |
| 2 | 3 | 1.26E+08 | 126373724 | TXNRD3 | -1.15177 | 187.048 | 16 | 9.42587 |
| 2 | 15 | 1.01E+08 | 101120608 | CERS3,LINS1 | -1.15448 | 161.388 | 18 | 11.2365 |
| 2 | 2 | 26435330 | 26512905 | HADHA,HADHB | -1.15891 | 152.694 | 31 | 17.6939 |
| 2 | 15 | 43109884 | 43473536 | TTBK2,UBR1,TMEM62 | -1.17405 | 149.717 | 72 | 42.0906 |
| 2 | 10 | 7797985 | 7849745 | KIN,ATP5F1C | -1.1745 | 268.902 | 26 | 15.8334 |
| 2 | 2 | 2.04E+08 | 204161363 | ICA1L,WDR12,CARF,NBEAL1,CYP20A1 | -1.17885 | 162.807 | 126 | 71.7067 |
| 2 | 4 | 56319173 | 57361620 | CLOCK,PDCL2,NMU,EXOC1L,EXOC1,CEP135,KIAA1211,AASDH,PPAT,PAICS,SRP72 | -1.18338 | 174.799 | 169 | 98.3113 |
| 2 | 14 | 52899027 | 60900146 | TXNDC16,GPR137C,ERO1A,PSMC6,STYX,GNPNAT1,FERMT2,DDHD1,MIR5580,BMP4,CDKN3,CNIH1,GMFB,CGRRF1,SAMD4A,GCH1,GCH1,MIR4308,WDHD1,SOCS4,MAPK1IP1L,LGALS3,DLGAP5,FBXO34,ATG14,TBPL2,KTN1-AS1,KTN1,PELI2,TMEM260,OTX2,EXOC5,AP5M1,NAA30,CCDC198,SLC35F4,C14orf37,ACTR10,PSMA3,PSMA3,PSMA3-AS1,PSMA3-AS1,ARID4A,TOMM20L,TOMM20L,TIMM9,TIMM9,TIMM9,KIAA0586,KIAA0586,DACT1,DAAM1,GPR135,L3HYPDH,JKAMP,JKAMP,CCDC175,CCDC175,RTN1,RTN1,MIR5586,LRRC9,PCNX4,DHRS7,PPM1A | -1.195 | 156.087 | 807 | 471.665 |
| 2 | 17 | 16455127 | 16734282 | ZNF287,ZNF624,CCDC144A | -1.20111 | 142.648 | 45 | 23.6819 |
| 2 | 12 | 71953332 | 85407733 | LGR5,ZFC3H1,THAP2,TMEM19,RAB21,TBC1D15,TPH2,TRHDE-AS1,TRHDE-AS1,TRHDE,TRHDE,ATXN7L3B,KCNC2,CAPS2,CAPS2,GLIPR1L1,GLIPR1L2,GLIPR1,GLIPR1,KRR1,KRR1,PHLDA1,NAP1L1,BBS10,OSBPL8,ZDHHC17,CSRP2,E2F7,NAV3,SYT1,SYT1,MIR1252,PAWR,PPP1R12A,OTOGL,PTPRQ,MYF6,MYF5,LIN7A,LIN7A,MIR617,LIN7A,MIR618,ACSS3,ACSS3,MIR4699,PPFIA2,PPFIA2,PPFIA2-AS1,CCDC59,CCDC59,METTL25,METTL25,TMTC2,SLC6A15 | -1.20875 | 146.689 | 808 | 469.76 |

| | | | | | | | | |
|---|---|---|---|---|---|---|---|---|
| 2 | 21 | 9411693 | 27141363 | MIR3648-1,MIR3648-2,TEKT4P2,TPTE,BAGE2,BAGE4,BAGE3,BAGE5,BAGE2,BAGE4,BAGE3,BAGE5,BAGE,ANKRD20A11P,LIPI,RBM11,ABCC13,HSPA13,SAMSN1,SAMSN1,SAMSN1-AS1,SAMSN1-AS1,SAMSN1-AS1,LOC388813,LOC388813,NRIP1,USP25,MIR99AHG,MIR99AHG,MIR99A,MIR99AHG,MIRLET7C,MIR99AHG,MIR125B2,LINC01549,CXADR,BTG3,C21orf91,CHODL,TMPRSS15,MIR548XHG,NCAM2,MIR155HG,MIR155,MRPL39,JAM2,ATP5PF,ATP5PF,GABPA,GABPA | -1.20967 | 144.886 | 428 | 222.547 |
| 2 | 12 | 1.19E+08 | 118839904 | TAOK3,SUDS3 | -1.20996 | 134.97 | 28 | 15.4398 |
| 2 | 15 | 99796241 | 100243011 | LRRC28,LRRC28,HSP90B2P,MEF2A | -1.21883 | 148.518 | 34 | 18.1618 |
| 2 | 7 | 48028361 | 48319514 | SUN3,C7orf57,UPP1,ABCA13 | -1.23195 | 145.86 | 77 | 49.645 |
| 2 | 11 | 66515814 | 66581469 | C11orf80 | -1.23296 | 150.967 | 10 | 5.81184 |
| 2 | 14 | 96756380 | 97347571 | ATG2B,GSKIP,AK7,PAPOLA,VRK1 | -1.25611 | 140.043 | 108 | 63.5182 |
| 2 | 10 | 32096541 | 32337541 | ARHGAP12,KIF5B | -1.26011 | 149.772 | 42 | 26.6466 |
| 2 | 16 | 31580677 | 31987310 | YBX3P1,ZNF720,ZNF267 | -1.26771 | 141.082 | 47 | 24.5098 |
| 2 | 18 | 35539848 | 42280805 | MIR924HG,MIR924,MIR924HG,MIR5583-2,MIR5583-1,PIK3C3,RIT2,SYT4 | -1.26871 | 89.3662 | 90 | 52.2623 |
| 2 | 9 | 1.03E+08 | 102822552 | NR4A3,STX17,ERP44 | -1.2792 | 123.582 | 34 | 18.2558 |
| 2 | 1 | 1.1E+08 | 110146751 | GNAI3,MIR197,GNAT2 | -1.28649 | 118.101 | 11 | 6.64885 |
| 2 | 1 | 94335227 | 94370161 | DNTTIP2,GCLM | -1.29043 | 211.937 | 19 | 11.6542 |
| 2 | 1 | 1.83E+08 | 182920629 | DHX9,SHCBP1L | -1.29693 | 157.577 | 43 | 24.6991 |
| 2 | 5 | 73992491 | 75008792 | HEXB,HEXB,GFM2,GFM2,GFM2,NSA2,NSA2,FAM169A,GCNT4,ANKRD31,HMGCR,COL4A3BP,COL4A3BP,POLK,POLK,ANKDD1B,POC5 | -1.2987 | 146.306 | 192 | 114.74 |
| 2 | 3 | 1.79E+08 | 182683535 | PIK3CA,PIK3CA,KCNMB3,KCNMB3,ZNF639,MFN1,GNB4,ACTL6A,MRPL47,NDUFB5,USP13,PEX5L,PEX5L,PEX5L-AS2,TTC14,TTC14,CCDC39,CCDC39,LOC101928882,FXR1,DNAJC19,SOX2-OT,SOX2,ATP11B,DCUN1D1 | -1.30882 | 146.632 | 295 | 169.654 |
| 2 | 3 | 57232204 | 57899030 | HESX1,APPL1,APPL1,ASB14,APPL1,ASB14,LOC105377102,ASB14,LOC105377102,ASB14,DNAH12,PDE12,PDE12,ARF4,PDE12,DENND6A,DENND6A,SLMAP | -1.31868 | 164.012 | 165 | 96.5063 |
| 2 | 21 | 28338584 | 30444433 | ADAMTS5,N6AMT1,LTN1,RWDD2B,USP16,CCT8 | -1.32214 | 136.923 | 100 | 57.8806 |
| 2 | 10 | 89267899 | 90534953 | MINPP1,PAPSS2,ATAD1,KLLN,PTEN,RNLS,LIPJ,LIPF,LIPK,LIPN | -1.34289 | 150.408 | 123 | 69.3248 |
| 2 | 10 | 5804490 | 5853917 | FAM208B,GDI2 | -1.34612 | 141.196 | 14 | 7.65148 |
| 2 | 2 | 31806470 | 32422889 | MEMO1,DPY30,SPAST,SLC30A6 | -1.35602 | 122.221 | 61 | 29.6364 |
| 2 | X | 69519023 | 69606466 | KIF4A | -1.35888 | 120.627 | 19 | 10.2597 |
| 2 | 12 | 59307696 | 62982760 | LRIG3,SLC16A7,FAM19A2,USP15,MIR6125,USP15,MON2 | -1.36471 | 112.492 | 128 | 73.2953 |
| 2 | 2 | 1.81E+08 | 189434918 | CWC22,UBE2E3,LINC01934,MIR4437,ITGA4,ITGA4,CERKL,CERKL,NEUROD1,SSFA2,PPP1R1C,PDE1A,DNAJC10,FRZB,NCKAP1,DUSP19,NUP35,MIR548AE1,ZNF804A,LOC101927196,FSIP2,FSIP2,FSIP2,FSIP2-AS1,ZC3H15,ITGAV,FAM171B,ZSWIM2,CALCRL,TFPI,GULP1 | -1.37335 | 128.925 | 506 | 305.597 |
| 2 | 2 | 72722561 | 72960267 | EXOC6B | -1.3893 | 111.622 | 17 | 9.44432 |
| 2 | 7 | 74105344 | 74167458 | GTF2I,LOC101926943,GTF2I | -1.39004 | 167.093 | 14 | 6.59744 |
| 2 | 8 | 1.33E+08 | 133014085 | EFR3A | -1.39781 | 137.39 | 10 | 6.20894 |
| 2 | 12 | 85450461 | 88442149 | LRRIQ1,ALX1,RASSF9,NTS,MGAT4C,LINC02258,MKRN9P,C12orf50,C12orf29 | -1.39849 | 125.765 | 103 | 59.0123 |

| | | | | | | | | |
|---|---|---|---|---|---|---|---|---|
| 2 | 1 | 92773371 | 94009613 | RPAP2,GFI1,EVI5,RPL5,RPL5,FAM69A,RPL5,FAM69A,SNORD21,RPL5,FAM69A,SNORA66,FAM69A,MTF2,TMED5,TMED5,CCDC18,CCDC18,DR1,FNBP1L | -1.40293 | 129.724 | 145 | 81.4948 |
| 2 | 10 | 1.13E+08 | 112769157 | PDCD4,PDCD4,MIR4680,BBIP1,SHOC2,RPL13AP6,SHOC2 | -1.42586 | 110.107 | 35 | 18.7181 |
| 2 | 20 | 47679727 | 47731579 | CSE1L,STAU1 | -1.43486 | 152.534 | 26 | 17.4761 |
| 2 | 3 | 1.25E+08 | 125216250 | ZNF148,SNX4 | -1.43662 | 119.145 | 20 | 12.0095 |
| 2 | 9 | 1.05E+08 | 106898306 | LINC00587,CYLC2,SMC2 | -1.44326 | 82.4572 | 51 | 30.0622 |
| 2 | 15 | 65956635 | 66172171 | DENND4A,RAB11A | -1.4475 | 139.928 | 46 | 27.3094 |
| 2 | 20 | 34292455 | 34324545 | RBM39 | -1.4536 | 108.323 | 19 | 10.0697 |
| 2 | 3 | 45744909 | 45781186 | SACM1L | -1.46344 | 141.304 | 15 | 9.63547 |
| 2 | 14 | 91928424 | 92628196 | PPP4R3A,CATSPERB,TC2N,FBLN5,TRIP11,ATXN3,NDUFB1,CPSF2 | -1.46951 | 137.543 | 155 | 86.7555 |
| 2 | 11 | 9191339 | 9466674 | DENND5A,TMEM41B,IPO7,IPO7,SNORA23 | -1.47144 | 123.124 | 57 | 31.2067 |
| 2 | 2 | 15601808 | 15768953 | NBAS,DDX1 | -1.485 | 139.035 | 46 | 27.4062 |
| 2 | 2 | 29001694 | 29084164 | PPP1CB,SPDYA,SPDYA,TRMT61B,TRMT61B | -1.49025 | 115.74 | 18 | 10.4416 |
| 2 | 8 | 1.31E+08 | 130874646 | FAM49B | -1.49076 | 128.144 | 11 | 6.09457 |
| 2 | 6 | 7327011 | 7413250 | CAGE1,RIOK1 | -1.50303 | 140.773 | 35 | 21.2871 |
| 2 | 5 | 98194643 | 102515895 | CHD1,FAM174A,ST8SIA4,ST8SIA4,MIR548P,SLCO4C1,SLCO6A1,PAM,GIN1,PPIP5K2 | -1.52371 | 111.419 | 175 | 97.3606 |
| 2 | 22 | 41222638 | 41246974 | ST13 | -1.53362 | 132.252 | 10 | 4.55266 |
| 2 | 10 | 96087720 | 96361470 | PLCE1,NOC3L,TBC1D12,HELLS | -1.53472 | 145.435 | 65 | 37.8881 |
| 2 | 16 | 1887762 | 1918190 | FAHD1,MEIOB,MEIOB | -1.54319 | 144.334 | 12 | 7.57558 |
| 2 | 11 | 72694784 | 72851187 | FCHSD2 | -1.54374 | 84.5174 | 13 | 7.00136 |
| 2 | 2 | 2.34E+08 | 234474205 | USP40 | -1.54518 | 133.345 | 25 | 15.7642 |
| 2 | 1 | 2.26E+08 | 226488992 | LIN9 | -1.54521 | 96.3891 | 15 | 8.13164 |
| 2 | X | 1.06E+08 | 106114056 | CXorf57,RNF128,TBC1D8B | -1.54922 | 121.198 | 53 | 31.2266 |
| 2 | 3 | 38257571 | 38291603 | OXSR1 | -1.55434 | 129.388 | 12 | 7.2704 |
| 2 | 17 | 45405568 | 45677111 | EFCAB13,MRPL45P2,NPEPPS | -1.56353 | 167.906 | 63 | 32.9755 |
| 2 | 14 | 1.01E+08 | 101456552 | [illegible] 2,MEG8,MEG8,SNORD113-4,MEG8,SNORD113-5,MEG8,SNORD113-6,MEG8,SNORD113-7,MEG8,SNORD113-9,MEG8,SNORD114-1,MEG8,SNORD114-2,MEG8,SNORD114-3,MEG8,SNORD114-4,MEG8,SNORD114-5,MEG8,SNORD114-6,MEG8,SNORD114-7,MEG8,SNORD114-8,MEG8,SNORD114-9,MEG8,SNORD114-10,MEG8,SNORD114-11,MEG8,SNORD114-12,MEG8,SNORD114-13,MEG8,SNORD114-14,MEG8,SNORD114-15,MEG8,SNORD114-16,MEG8,SNORD114-17,MEG8,SNORD114-18,MEG8,SNORD114-19,MEG8,SNORD114-20,MEG8,SNORD114-21,MEG8,SNORD114-22,MEG8,SNORD114-23,MEG8,SNORD114-24,MEG8,SNORD114-25,MEG8,SNORD114-26,MEG8,SNORD114-27,MEG8,SNORD114-28,MEG8,SNORD114-29 | -1.56539 | 115.992 | 47 | 27.134 |
| 2 | 17 | 27802675 | 27838048 | TAOK1 | -1.57307 | 139.93 | 12 | 7.42368 |
| 2 | 11 | 1.19E+08 | 118656861 | DDX6 | -1.57812 | 95.8857 | 19 | 9.79617 |
| 2 | 12 | 1.23E+08 | 123110903 | ZCCHC8,RSRC2,KNTC1 | -1.58285 | 149.264 | 86 | 51.45 |
| 2 | 3 | 44284366 | 44449180 | TOPAZ1,TCAIM | -1.5838 | 103.312 | 44 | 26.8486 |
| 2 | 2 | 39223978 | 39606966 | SOS1,CDKL4,MAP4K3 | -1.58774 | 113.802 | 73 | 41.6681 |
| 2 | 3 | 1.85E+08 | 184711902 | VPS8 | -1.60143 | 128.567 | 53 | 29.7345 |
| 2 | 3 | 1.93E+08 | 193410043 | ATP13A4,ATP13A4,ATP13A4-AS1,OPA1,OPA1,OPA1-AS1 | -1.60229 | 123.372 | 43 | 24.5072 |
| 2 | 3 | 56592736 | 56707747 | CCDC66,CCDC66,FAM208A,FAM208A | -1.60776 | 142.658 | 57 | 34.781 |
| 2 | 8 | 30608848 | 31012299 | UBXN8,PPP2CB,TEX15,PURG,WRN | -1.61237 | 126.496 | 105 | 64.9521 |
| 2 | 1 | 2.45E+08 | 244781031 | ADSS,CATSPERE | -1.61574 | 117.679 | 48 | 26.9206 |

| | | | | | | | | |
|---|---|---|---|---|---|---|---|---|
| 2 | X | 1.23E+08 | 123505333 | THOC2,XIAP,STAG2,SH2D1A | -1.61819 | 116.818 | 84 | 49.1291 |
| 2 | 6 | 4031761 | 4074911 | PRPF4B,FAM217A | -1.61851 | 121.154 | 32 | 18.8589 |
| 2 | 15 | 40894984 | 40949657 | KNL1 | -1.6282 | 86.7504 | 40 | 25.9016 |
| 2 | X | 1.29E+08 | 128652508 | SMARCA1 | -1.643 | 123.727 | 24 | 14.3599 |
| 2 | 3 | 1.7E+08 | 170016983 | PRKCI | -1.65623 | 78.1277 | 19 | 9.98204 |
| 2 | 2 | 1.91E+08 | 191175850 | ANKAR,ANKAR,OSGEPL1,OSGEPL1,ORMDL1,PMS1,MSTN,C2orf88,HIBCH | -1.66705 | 117.929 | 86 | 50.5709 |
| 2 | 7 | 81594857 | 82532073 | CACNA2D1,CACNA2D1,LOC101927356,PCLO | -1.67243 | 118.977 | 71 | 38.3872 |
| 2 | 8 | 27593690 | 27690717 | CCDC25,ESCO2,PBK | -1.67358 | 126.608 | 34 | 19.2872 |
| 2 | X | 1.18E+08 | 117901035 | WDR44,WDR44,MIR1277,DOCK11,IL13RA1 | -1.67545 | 99.8053 | 109 | 58.679 |
| 2 | 6 | 76331162 | 76633501 | SENP6,MYO6,IMPG1 | -1.67593 | 121.541 | 63 | 38.5419 |
| 2 | X | 1.29E+08 | 129493024 | ZNF280C,SLC25A14 | -1.67749 | 101.891 | 29 | 16.4411 |
| 2 | 12 | 1.12E+08 | 111993775 | ATXN2 | -1.68057 | 115.669 | 17 | 9.93786 |
| 2 | 19 | 34921399 | 34960222 | UBA2 | -1.68668 | 111.272 | 22 | 11.6508 |
| 2 | 1 | 1.13E+08 | 113212154 | ST7L,CAPZA1 | -1.69486 | 114.587 | 32 | 17.9254 |
| 2 | 3 | 8931995 | 9000767 | RAD18 | -1.69567 | 107.755 | 12 | 7.18339 |
| 2 | 17 | 65211891 | 65739894 | HELZ,PSMD12,PITPNC1,PITPNC1,MIR548AA2,MIR548D2,NOL11,NOL11,SNORA38B | -1.70173 | 133.092 | 62 | 31.4232 |
| 2 | X | 8667686 | 9000268 | ANOS1,FAM9A,FAM9B | -1.71185 | 179.154 | 20 | 9.33008 |
| 2 | 10 | 35302527 | 35426839 | CUL2,CUL2,MIR3611,CREM | -1.71365 | 120.181 | 21 | 13.2247 |
| 2 | 15 | 80365524 | 80423718 | ZFAND6 | -1.71552 | 87.3652 | 10 | 5.5004 |
| 2 | 11 | 17112888 | 17372888 | PIK3C2A,NUCB2 | -1.72488 | 105.466 | 46 | 28.7903 |
| 2 | 22 | 45754554 | 45804789 | SMC1B | -1.72605 | 134.915 | 19 | 11.8001 |
| 2 | 10 | 1.17E+08 | 117486201 | ATRNL1 | -1.73001 | 81.3012 | 43 | 21.4864 |
| 2 | 10 | 1.12E+08 | 112361967 | SMC3 | -1.73397 | 94.0037 | 25 | 14.7849 |
| 2 | 2 | 1.52E+08 | 152331530 | RIF1 | -1.73747 | 112.336 | 43 | 27.9841 |
| 2 | 4 | 1.29E+08 | 129043425 | PLK4,MFSD8,ABHD18,LARP1B | -1.74895 | 116.79 | 55 | 32.0349 |
| 2 | 4 | 1.46E+08 | 146095810 | ANAPC10,ABCE1,OTUD4 | -1.74955 | 127.42 | 63 | 31.0415 |
| 2 | 3 | 69072297 | 69124773 | TMF1,TMF1,MIR3136,UBA3 | -1.75057 | 140.475 | 39 | 23.4492 |
| 2 | 2 | 96521007 | 96648124 | ANKRD36C | -1.76728 | 216.129 | 24 | 10.2996 |
| 2 | 19 | 34699917 | 34718241 | LSM14A | -1.77002 | 114.116 | 10 | 5.24679 |
| 2 | 17 | 66877263 | 67309407 | ABCA8,ABCA9,ABCA9,ABCA9-AS1,ABCA6,ABCA6,MIR4524B,MIR4524A,ABCA10,ABCA5 | -1.7705 | 114.264 | 197 | 115.097 |
| 2 | 8 | 1.25E+08 | 124824801 | FAM91A1 | -1.77515 | 106.696 | 27 | 13.8997 |
| 2 | 4 | 1.3E+08 | 130032982 | SCLT1,C4orf33 | -1.77541 | 95.6848 | 28 | 15.1914 |
| 2 | 2 | 37317701 | 37449657 | GPATCH11,EIF2AK2,SULT6B1,CEBPZOS,CEBPZOS,CEBPZ,CEBPZ | -1.79468 | 128.842 | 47 | 26.6952 |
| 2 | 12 | 96897661 | 97345925 | CFAP54,NEDD1 | -1.80039 | 103.664 | 91 | 53.6584 |
| 2 | 10 | 1.03E+08 | 102721790 | SLF2 | -1.80061 | 107.233 | 15 | 8.60977 |
| 2 | 20 | 469392 | 485964 | CSNK2A1 | -1.80108 | 137.219 | 11 | 5.87349 |
| 2 | 17 | 38548251 | 38573113 | TOP2A | -1.80211 | 123.425 | 32 | 19.7772 |
| 2 | 11 | 1.03E+08 | 103191972 | DCUN1D5,DYNC2H1 | -1.80472 | 106.574 | 91 | 54.7737 |
| 2 | X | 1.33E+08 | 133674670 | CCDC160,PHF6,HPRT1,MIR450B,MIR450A1,MIR450A1,MIR450A2 | -1.80693 | 76.9862 | 32 | 17.6356 |
| 2 | 21 | 40578007 | 40650808 | BRWD1 | -1.82507 | 115.662 | 27 | 16.5604 |
| 2 | 2 | 55402884 | 55914912 | CLHC1,RPS27A,RPS27A,MIR4426,MTIF2,CCDC88A,CFAP36,PPP4R3B,PNPT1 | -1.8268 | 115.28 | 141 | 82.6577 |
| 2 | 2 | 1.72E+08 | 172333477 | TLK1,METTL8,METTL8,DCAF17,DCAF17 | -1.82684 | 79.7575 | 61 | 32.405 |
| 2 | 15 | 50731339 | 51242381 | USP8,USP8,USP50,USP50,TRPM7,SPPL2A,AP4E1 | -1.83077 | 107.531 | 118 | 64.8931 |
| 2 | 2 | 1.7E+08 | 170802983 | BBS5,KLHL41,FASTKD1,PPIG,CCDC173,PHOSPHO2-KLHL23,PHOSPHO2,PHOSPHO2-KLHL23,KLHL23,KLHL23,SSB,SSB,METTL5,METTL5,UBR3 | -1.83173 | 116.768 | 137 | 75.9107 |
| 2 | 3 | 1.6E+08 | 160254708 | TRIM59-IFT80,IFT80,TRIM59-IFT80,SMC4,TRIM59-IFT80,SMC4,MIR15B,MIR16-2,TRIM59-IFT80,TRIM59,TRIM59-IFT80,KPNA4,KPNA4,SCARNA7 | -1.83849 | 104.496 | 87 | 46.9983 |
| 2 | 3 | 39107246 | 39133242 | WDR48 | -1.84775 | 116.995 | 15 | 9.47807 |

| | | | | | | | | |
|---|---|---|---|---|---|---|---|---|
| 2 | 2 | 17692091 | 17963224 | RAD51AP2,VSNL1,SMC6,GEN1 | -1.84787 | 106.009 | 66 | 40.8553 |
| 2 | 8 | 38975487 | 39694801 | ADAM32,ADAM3A,ADAM18,ADAM2 | -1.85748 | 98.5304 | 96 | 49.6004 |
| 2 | 3 | 1.38E+08 | 138291828 | ESYT3,CEP70 | -1.86212 | 71.9669 | 21 | 11.985 |
| 2 | 9 | 38571952 | 38611371 | ANKRD18A | -1.87311 | 164.394 | 15 | 8.01132 |
| 2 | 6 | 1.37E+08 | 136597335 | MTFR2,BCLAF1 | -1.8738 | 143.437 | 28 | 15.2341 |
| 2 | 5 | 1.37E+08 | 137347595 | PKD2L2,PKD2L2,FAM13B,FAM13B | -1.88834 | 84.3757 | 36 | 21.0142 |
| 2 | 18 | 18520842 | 18650639 | ROCK1 | -1.89384 | 130.203 | 36 | 21.1256 |
| 2 | 17 | 3346433 | 3392721 | SPATA22,SPATA22,ASPA | -1.90777 | 121.331 | 12 | 6.44542 |
| 2 | 2 | 11312739 | 11597301 | PQLC3,ROCK2,E2F6 | -1.91851 | 118.035 | 48 | 27.6351 |
| 2 | 2 | 73957005 | 74002249 | TPRKB,DUSP11 | -1.93296 | 80.5004 | 13 | 7.9168 |
| 2 | 10 | 71913511 | 71990228 | SAR1A,PPA1 | -1.93822 | 81.7365 | 23 | 11.3213 |
| 2 | 5 | 61642892 | 61897715 | KIF2A,DIMT1,IPO11,IPO11,LRRC70,IPO11-LRRC70,IPO11,IPO11-LRRC70 | -1.93982 | 116.586 | 76 | 42.9169 |
| 2 | 5 | 86626618 | 87524408 | RASA1,CCNH,TMEM161B | -1.95715 | 104.91 | 56 | 31.9157 |
| 2 | X | 69826762 | 69945288 | TEX11 | -1.97145 | 79.8585 | 16 | 8.67929 |
| 2 | 12 | 1.06E+08 | 105582297 | WASHC4,APPL2 | -1.98088 | 110.312 | 36 | 21.2999 |
| 2 | 17 | 33255934 | 33281629 | CCT6B | -1.98204 | 97.2386 | 10 | 5.85191 |
| 2 | 22 | 38945892 | 38978542 | DMC1,FAM227A | -1.99375 | 88.8583 | 11 | 6.01604 |
| 2 | 1 | 2.25E+08 | 225496132 | DNAH14 | -1.99558 | 80.0035 | 73 | 41.1683 |
| 2 | 10 | 97681720 | 97784741 | ENTPD1-AS1,CC2D2B | -1.99601 | 83.4968 | 23 | 14.2196 |
| 2 | 4 | 1.85E+08 | 184606329 | RWDD4,TRAPPC11 | -2.03809 | 114.303 | 20 | 11.4951 |
| 2 | 15 | 79170435 | 79189324 | MORF4L1 | -2.03858 | 120.875 | 14 | 7.86229 |
| 2 | 2 | 27900113 | 27917484 | SLC4A1AP | -2.04481 | 92.7109 | 13 | 6.68611 |
| 2 | 4 | 2209986 | 2242419 | POLN,POLN,HAUS3 | -2.05008 | 85.7788 | 11 | 7.15853 |
| 2 | 3 | 1.72E+08 | 172520547 | ECT2 | -2.05253 | 111.219 | 17 | 10.5389 |
| 2 | 5 | 54552278 | 54711981 | DHX29,MTREX | -2.05597 | 110.178 | 60 | 33.9058 |
| 2 | 3 | 1.67E+08 | 167240349 | ZBBX,SERPINI2,WDR49 | -2.06044 | 76.626 | 33 | 18.8509 |
| 2 | 3 | 5212065 | 5216226 | ARL8B | -2.06367 | 88.3054 | 10 | 5.3846 |
| 2 | 4 | 1.86E+08 | 185652200 | CASP3,PRIMPOL,PRIMPOL,CENPU,CENPU | -2.08538 | 92.4605 | 38 | 21.0399 |
| 2 | 10 | 1.32E+08 | 131977594 | GLRX3 | -2.08688 | 65.0243 | 14 | 6.63297 |
| 2 | 13 | 41892928 | 41948981 | NAA16 | -2.08785 | 103.278 | 19 | 10.9037 |
| 2 | X | 1.14E+08 | 114422944 | IL13RA2,LRCH2 | -2.10101 | 83.3734 | 26 | 15.6592 |
| 2 | 5 | 68487522 | 68706460 | CENPH,MRPS36,CDK7,CCDC125,AK6,AK6,TAF9,AK6,TAF9,RAD17,RAD17 | -2.10193 | 113.79 | 71 | 38.56 |
| 2 | 7 | 1.21E+08 | 121644851 | FAM3C,PTPRZ1 | -2.10262 | 87.4617 | 40 | 20.9631 |
| 2 | 7 | 30089927 | 30102425 | PLEKHA8 | -2.1226 | 100.491 | 10 | 5.39846 |
| 2 | 16 | 81012218 | 81060236 | CMC2,CENPN | -2.12558 | 81.0129 | 18 | 10.0743 |
| 2 | 1 | 1.73E+08 | 173569368 | SLC9C2 | -2.13517 | 87.8344 | 20 | 10.8374 |
| 2 | 1 | 2.47E+08 | 247081765 | AHCTF1 | -2.13627 | 54.0457 | 31 | 14.3344 |
| 2 | 13 | 32899142 | 32929465 | BRCA2 | -2.13916 | 82.0016 | 33 | 22.7954 |
| 2 | 12 | 64808565 | 64891882 | XPOT,TBK1 | -2.14357 | 89.7747 | 49 | 28.1788 |
| 2 | 15 | 44615047 | 44816344 | CASC4,CTDSPL2 | -2.1473 | 70.8367 | 31 | 15.2663 |
| 2 | 14 | 50889782 | 50952917 | MAP4K5 | -2.14835 | 93.7189 | 28 | 15.6222 |
| 2 | 5 | 1.39E+08 | 138665023 | MATR3 | -2.15123 | 93.2668 | 17 | 9.35661 |
| 2 | 4 | 1.14E+08 | 113825743 | ZGRF1,LARP7,LARP7,LOC109864269,LARP7,LOC109864269,MIR367,MIR302D,LARP7,LOC109864269,MIR302A,MIR302C,LARP7,LOC109864269,MIR302C,MIR302B,ANK2 | -2.15484 | 73.662 | 39 | 22.2191 |
| 2 | 1 | 1.63E+08 | 163318882 | NUF2 | -2.16012 | 70.2826 | 12 | 7.02151 |
| 2 | 21 | 34048578 | 34117295 | SYNJ1,SYNJ1,PAXBP1-AS1,PAXBP1-AS1,PAXBP1,PAXBP1 | -2.16273 | 108.738 | 23 | 13.2797 |
| 2 | 4 | 68338360 | 68547398 | CENPC,STAP1,UBA6 | -2.16393 | 86.518 | 74 | 40.4738 |
| 2 | 4 | 70800353 | 71113635 | CSN1S1,CSN2,STATH,HTN3,HTN1,CSN1S2AP,CSN1S2BP,PRR27,ODAM,FDCSP,CSN3 | -2.1711 | 97.6703 | 71 | 38.8263 |
| 2 | X | 1.2E+08 | 119691947 | CUL4B | -2.17249 | 84.0804 | 20 | 11.8156 |
| 2 | X | 72783963 | 73045726 | CHIC1,TSIX,XIST | -2.17606 | 73.8481 | 29 | 18.7781 |
| 2 | 10 | 76971879 | 76989532 | VDAC2 | -2.17721 | 90.5212 | 11 | 5.38811 |
| 2 | 5 | 1.49E+08 | 148904785 | CSNK1A1 | -2.17953 | 84.2147 | 10 | 4.73623 |
| 2 | 16 | 66801260 | 66861580 | TERB1,NAE1 | -2.18494 | 102.458 | 31 | 18.7481 |

| | | | | | | | | |
|---|---|---|---|---|---|---|---|---|
| 2 | 10 | 1.16E+08 | 115923122 | CCDC186 | -2.19721 | 95.6059 | 12 | 7.19134 |
| 2 | 7 | 27807396 | 27842845 | TAX1BP1 | -2.19923 | 65.2196 | 15 | 8.3577 |
| 2 | 11 | 1.12E+08 | 112042669 | IL18,TEX12 | -2.20639 | 64.0335 | 10 | 5.38535 |
| 2 | 17 | 29161430 | 29221974 | ATAD5 | -2.20971 | 85.6527 | 29 | 18.4293 |
| 2 | 18 | 30518568 | 31224276 | CCDC178,ASXL3 | -2.21169 | 62.1343 | 42 | 20.6092 |
| 2 | 3 | 1.08E+08 | 108388713 | MYH15,CIP2A,DZIP3 | -2.21624 | 99.2331 | 46 | 27.7144 |
| 2 | 13 | 53036582 | 53262021 | CKAP2,SUGT1 | -2.23047 | 115.158 | 23 | 12.6465 |
| 2 | 18 | 9104885 | 9134187 | NDUFV2,NDUFV2,NDUFV2-AS1 | -2.23883 | 70.9294 | 10 | 4.97136 |
| 2 | 2 | 2.02E+08 | 202469425 | STRADB,C2CD6 | -2.2503 | 81.0602 | 47 | 27.0315 |
| 2 | 3 | 1.7E+08 | 169526533 | MYNN,LRRC34 | -2.26119 | 74.3501 | 16 | 9.52605 |
| 2 | 2 | 2.39E+08 | 238743055 | RBM44 | -2.26632 | 99.9638 | 16 | 10.8037 |
| 2 | X | 96140629 | 96330337 | DIAPH2 | -2.2671 | 49.4711 | 17 | 9.58898 |
| 2 | 8 | 1.26E+08 | 125535312 | TATDN1,TATDN1,MIR6844 | -2.26883 | 73.0784 | 16 | 7.8584 |
| 2 | X | 1.39E+08 | 138708467 | MCF2 | -2.2712 | 83.0797 | 17 | 9.9771 |
| 2 | 11 | 94153276 | 94229910 | MRE11,MRE11,MIR548L,ANKRD49 | -2.2714 | 74.8154 | 30 | 17.1285 |
| 2 | 17 | 11924855 | 12013819 | MAP2K4,MAP2K4,MIR744 | -2.27783 | 67.127 | 16 | 7.5958 |
| 2 | 7 | 76991872 | 77266439 | GSAP,GSAP,LOC101927243,PTPN12 | -2.27965 | 87.6491 | 37 | 20.8581 |
| 2 | 2 | 2.38E+08 | 238006302 | COPS8 | -2.28381 | 61.7184 | 10 | 5.53409 |
| 2 | 4 | 1.41E+08 | 141471498 | SCOC,CLGN,MGAT4D,ELMOD2 | -2.29612 | 92.229 | 41 | 22.5385 |
| 2 | 8 | 1.21E+08 | 121514887 | MTBP | -2.30018 | 59.8911 | 15 | 8.97238 |
| 2 | 13 | 25353782 | 25440410 | RNF17 | -2.31619 | 75.5669 | 29 | 17.8561 |
| 2 | 2 | 2.01E+08 | 201448228 | SGO2 | -2.32135 | 63.9077 | 22 | 13.6812 |
| 2 | 10 | 27294428 | 27381506 | ANKRD26 | -2.33984 | 86.191 | 35 | 19.7036 |
| 2 | 2 | 21361327 | 21366129 | TDRD15 | -2.34956 | 58.5931 | 20 | 14.1754 |
| 2 | 6 | 1.21E+08 | 121625954 | TBC1D32 | -2.35574 | 66.2465 | 31 | 16.4602 |
| 2 | 1 | 1.76E+08 | 176153875 | RFWD2 | -2.36617 | 44.4042 | 24 | 12.1098 |
| 2 | 9 | 1.14E+08 | 114553803 | C9orf84 | -2.37151 | 80.9964 | 32 | 19.3765 |
| 2 | 13 | 96508866 | 96684255 | UGGT2 | -2.37721 | 60.6541 | 41 | 22.9296 |
| 2 | 13 | 95228531 | 95246247 | TGDS | -2.38056 | 79.286 | 12 | 6.54408 |
| 2 | 11 | 89902006 | 89951426 | NAALAD2,CHORDC1 | -2.3907 | 69.2603 | 25 | 13.6103 |
| 2 | 15 | 56665563 | 57017227 | TEX9,TEX9,MNS1,MNS1,ZNF280D | -2.40013 | 75.122 | 52 | 30.5885 |
| 2 | 18 | 76873231 | 76933841 | ATP9B | -2.40267 | 53.1418 | 10 | 4.35223 |
| 2 | 10 | 70892605 | 70947513 | VPS26A,SUPV3L1 | -2.40517 | 117.466 | 20 | 11.0485 |
| 2 | 5 | 70828085 | 70861974 | BDP1 | -2.41815 | 84.2171 | 21 | 11.2627 |
| 2 | 20 | 1210532 | 1229463 | RAD21L1 | -2.43212 | 67.4975 | 11 | 6.83565 |
| 2 | 9 | 33533383 | 33567306 | ANKRD18B | -2.45398 | 134.039 | 13 | 7.06106 |
| 2 | 2 | 71582792 | 71645853 | ZNF638 | -2.46816 | 103.953 | 26 | 15.2869 |
| 2 | 9 | 79814790 | 80022620 | VPS13A | -2.4818 | 74.3616 | 75 | 43.2024 |
| 2 | 15 | 62238501 | 62351931 | VPS13C | -2.49528 | 78.7167 | 44 | 26.2953 |
| 2 | 2 | 70064772 | 70131426 | GMCL1,SNRNP27 | -2.49748 | 70.0612 | 26 | 13.4908 |
| 2 | 21 | 38459651 | 38564608 | TTC3,TTC3,TTC3-AS1 | -2.49997 | 81.5581 | 63 | 32.3171 |
| 2 | 13 | 21950728 | 22096833 | MIPEPP3,ZDHHC20,ZDHHC20,MICU2 | -2.50176 | 77.3272 | 29 | 15.4326 |
| 2 | 20 | 55906878 | 55918510 | SPO11 | -2.51972 | 59.5213 | 13 | 7.44615 |
| 2 | 2 | 2.14E+08 | 214239890 | SPAG16 | -2.52174 | 40.9843 | 14 | 7.48951 |
| 2 | 9 | 72920176 | 72965395 | SMC5 | -2.53796 | 65.613 | 15 | 8.05016 |
| 2 | 17 | 45201218 | 45259087 | CDC27 | -2.55436 | 50.7535 | 22 | 10.0929 |
| 2 | 6 | 1.23E+08 | 122752699 | HSF2 | -2.56603 | 71.2462 | 13 | 6.62372 |
| 2 | 11 | 32616390 | 32781764 | EIF3M,EIF3M,CCDC73,CCDC73 | -2.57892 | 58.0262 | 34 | 18.9442 |
| 2 | 2 | 75879224 | 75928448 | MRPL19,GCFC2 | -2.59474 | 67.9354 | 20 | 11.8082 |
| 2 | 4 | 1.86E+08 | 186384217 | C4orf47,C4orf47,CCDC110,CCDC110 | -2.59603 | 64.7299 | 18 | 11.9963 |
| 2 | 2 | 1.78E+08 | 178378703 | AGPS | -2.59649 | 38.9474 | 20 | 10.0492 |
| 2 | 14 | 94699924 | 94733399 | PPP4R4 | -2.5997 | 82.6305 | 18 | 11.1203 |
| 2 | 5 | 1.15E+08 | 114620667 | PGGT1B,CCDC112 | -2.60038 | 79.8467 | 21 | 11.6784 |
| 2 | 6 | 1.5E+08 | 149862732 | PPIL4 | -2.60057 | 53.0304 | 13 | 6.87271 |
| 2 | 4 | 1.04E+08 | 104118088 | CENPE | -2.62352 | 63.5123 | 45 | 27.8926 |
| 2 | 9 | 26984295 | 27061103 | IFT74,IFT74,LRRC19 | -2.63746 | 64.2453 | 21 | 13.2702 |
| 2 | 9 | 15468756 | 15506699 | PSIP1 | -2.65033 | 42.5711 | 18 | 9.32661 |
| 2 | 17 | 64001788 | 64066145 | CEP112 | -2.65881 | 68.4919 | 11 | 6.33251 |
| 2 | 10 | 32742230 | 33197337 | CCDC7,ITGB1 | -2.66125 | 36.1434 | 57 | 28.7869 |

| | | | | | | | | |
|---|---|---|---|---|---|---|---|---|
| 2 | 5 | 72147148 | 72354387 | TNPO1,TNPO1,MIR4804,FCHO2 | -2.66546 | 76.3753 | 51 | 25.7563 |
| 2 | 2 | 1.54E+08 | 153547641 | PRPF40A | -2.67863 | 59.5567 | 22 | 13.5639 |
| 2 | 18 | 47814520 | 47917706 | SKA1 | -2.6835 | 46.0231 | 7 | 3.91911 |
| 2 | 12 | 88443035 | 88589408 | C12orf29,CEP290,CEP290,TMTC3 | -2.69577 | 77.3903 | 71 | 42.1398 |
| 2 | 10 | 91470706 | 92502413 | KIF20B,HTR7 | -2.70109 | 44.7369 | 38 | 23.1473 |
| 2 | 3 | 1.12E+08 | 112005664 | SLC9C1 | -2.71235 | 53.833 | 29 | 15.6546 |
| 2 | 1 | 92706354 | 92772871 | C1orf146,GLMN,RPAP2 | -2.72397 | 54.2942 | 32 | 18.9156 |
| 2 | 20 | 5538588 | 5585031 | GPCPD1 | -2.73356 | 67.1538 | 26 | 14.2367 |
| 2 | 10 | 70666591 | 70703093 | DDX50 | -2.7492 | 47.5435 | 15 | 7.84465 |
| 2 | 5 | 76330224 | 76357554 | AGGF1 | -2.75919 | 66.7374 | 14 | 7.56331 |
| 2 | 6 | 1.24E+08 | 123760270 | TRDN | -2.77022 | 41.8044 | 34 | 17.7663 |
| 2 | 1 | 91726781 | 91866661 | HFM1 | -2.79808 | 48.8643 | 37 | 21.4927 |
| 2 | 10 | 98312678 | 98336603 | TM9SF3 | -2.79959 | 44.1809 | 6 | 3.53565 |
| 2 | 18 | 14763968 | 14851626 | ANKRD30B,ANKRD30B,MIR3156-2 | -2.79963 | 67.4935 | 23 | 11.1736 |
| 2 | 1 | 52205754 | 52226393 | OSBPL9 | -2.80067 | 59.155 | 10 | 6.06093 |
| 2 | 13 | 48881413 | 48955589 | RB1 | -2.80665 | 52.539 | 17 | 9.2899 |
| 2 | 4 | 26586637 | 26741657 | TBC1D19 | -2.81015 | 34.8833 | 20 | 10.0287 |
| 2 | 10 | 1.06E+08 | 105900790 | SFR1,CFAP43 | -2.82018 | 40.9171 | 11 | 6.75451 |
| 2 | 15 | 60724015 | 60768436 | ICE2 | -2.83604 | 69.5237 | 22 | 11.6809 |
| 2 | 20 | 13699450 | 13755981 | ESF1 | -2.85812 | 28.9635 | 15 | 7.43725 |
| 2 | 18 | 48422261 | 48450647 | ME2 | -2.88752 | 56.2157 | 13 | 7.61553 |
| 2 | 9 | 19058941 | 19096854 | HAUS6,HAUS6,SCARNA8 | -2.90238 | 56.1995 | 18 | 8.47488 |
| 2 | 10 | 37433795 | 37508737 | ANKRD30A | -2.91299 | 67.4438 | 16 | 7.38206 |
| 2 | 12 | 50822684 | 50848230 | LARP4 | -2.9399 | 35.0529 | 16 | 8.54288 |
| 2 | 15 | 49833877 | 49935816 | FAM227B,DTWD1 | -2.94157 | 55.285 | 23 | 12.0839 |
| 2 | 18 | 66346660 | 66381272 | TMX3 | -2.96158 | 42.299 | 13 | 7.97308 |
| 2 | 18 | 9239420 | 9256628 | ANKRD12 | -2.96354 | 25.2103 | 11 | 6.76231 |
| 2 | 10 | 94653100 | 94715540 | EXOC6 | -2.98509 | 43.8614 | 14 | 8.6486 |
| 2 | 4 | 1.56E+08 | 156283313 | MAP9,MAP9,LOC102724776 | -2.99901 | 45.178 | 10 | 5.54363 |
| 2 | 10 | 95422275 | 95451861 | PDE6C,FRA10AC1 | -3.03688 | 47.4589 | 16 | 8.46324 |
| 2 | 10 | 93220979 | 93258911 | HECTD2-AS1,HECTD2 | -3.07451 | 60.6985 | 16 | 9.30882 |
| 2 | 7 | 34982191 | 35058293 | DPY19L1 | -3.08846 | 30.9671 | 19 | 9.11403 |
| 2 | 9 | 86356862 | 86403657 | GKAP1 | -3.10274 | 23.6107 | 10 | 5.60005 |
| 2 | 12 | 1.01E+08 | 100729623 | SCYL2 | -3.11187 | 37.49 | 19 | 10.5054 |
| 2 | 6 | 88303955 | 88331801 | ORC3 | -3.14795 | 35.3356 | 15 | 8.47012 |
| 2 | 1 | 1.51E+08 | 150692062 | HORMAD1 | -3.18631 | 38.3486 | 17 | 8.68101 |
| 2 | 1 | 1.04E+08 | 104115913 | RNPC3,AMY2B | -3.189 | 37.8935 | 18 | 8.9871 |
| 2 | 16 | 10525192 | 10567925 | ATF7IP2 | -3.22362 | 31.0961 | 14 | 7.26097 |
| 2 | 10 | 1.18E+08 | 118187635 | CCDC172,PNLIPRP3 | -3.27033 | 14.1289 | 10 | 5.26039 |
| 2 | 4 | 84379417 | 84403434 | MRPS18C,MRPS18C,ABRAXAS1,ABRAXAS1 | -3.30169 | 33.8631 | 18 | 9.56343 |
| 2 | 1 | 86814439 | 86851297 | ODF2L | -3.34735 | 38.4041 | 17 | 9.93007 |
| 2 | 1 | 1.15E+08 | 115537456 | SYCP1 | -3.35177 | 18.9245 | 30 | 15.3669 |
| 2 | 6 | 1.09E+08 | 109484105 | CEP57L1 | -3.4159 | 51.0742 | 12 | 5.50951 |
| 2 | 20 | 58425361 | 58497518 | SYCP2 | -3.4345 | 32.8491 | 41 | 24.2281 |
| 2 | 2 | 1.98E+08 | 197762011 | PGAP1 | -3.45228 | 27.7239 | 15 | 8.97116 |
| 2 | 12 | 85408233 | 85450461 | TSPAN19,LRRIQ1 | -3.70542 | 37.2864 | 17 | 10.6555 |
| 2 | 17 | 5386004 | 5388625 | DERL2 | -3.90554 | 17.1443 | 3 | 1.83055 |
| 2 | 14 | 60903574 | 60951791 | C14orf39 | -3.91671 | 16.0471 | 16 | 8.3936 |
| 2 | Y | 2656174 | 10058894 | TGIF2LY,PCDH11Y,TBL1Y,PRKY | -3.95832 | 23.2764 | 90 | 44.1849 |
| 2 | 7 | 87516041 | 87530279 | DBF4 | -4.36463 | 15.439 | 12 | 5.73831 |
| 2 | Y | 13105053 | 59106039 | GYG2P1,UTY,VCY1B,VCY,VCY,VCY1B,NLGN4Y,TTTY14,CD24 | -5.41118 | 8.11333 | 151 | 75.6933 |
| 2 | 5 | 1.8E+08 | 180429824 | BTNL8,BTNL3 | -8.66984 | 10.4715 | 12 | 5.92183 |
| 3 | 1 | 1.5E+08 | 149871142 | HIST2H4A,HIST2H4B,HIST2H3C,HIST2H3A,HIST2H2AA3,HIST2H2AA4,HIST2H2BC,HIST2H2BE,HIST2H2AC,HIST2H2AB | 1.63904 | 791.229 | 23 | 10.5463 |

| | | | | | | | | |
|---|---|---|---|---|---|---|---|---|
| 3 | 8 | 1.26E+08 | 129311089 | TATDN1,NDUFB9,NDUFB9,MTSS1,MIR4662B,MIR4662A,ZNF572,SQLE,WASHC5,NSMCE2,TRIB1,FAM84B,PRNCR1,CASC8,POU5F1B,MYC,PVT1,MIR1204,PVT1,TMEM75,PVT1,MIR1205,PVT1,MIR1206,PVT1,MIR1207,MIR1208 | 1.61394 | 588.856 | 152 | 90.7384 |
| 3 | 8 | 1.22E+08 | 124787860 | SNTB1,HAS2,LINC01151,ZHX2,DERL1,TBC1D31,FAM83A,MIR4663,C8orf76,C8orf76,ZHX1-C8orf76,ZHX1-C8orf76,ZHX1,ATAD2,ATAD2,MIR548D1,WDYHV1,FBXO32,KLHL38,ANXA13,FAM91A1 | 1.52457 | 706.693 | 202 | 125.33 |
| 3 | 6 | 31783639 | 31797454 | HSPA1A,HSPA1B | 1.46809 | 642.107 | 16 | 10.8831 |
| 3 | 8 | 1.21E+08 | 121457931 | DSCC1,DEPTOR,COL14A1,MRPL13,MTBP | 1.43393 | 597.188 | 90 | 52.1509 |
| 3 | 8 | 1.25E+08 | 125499850 | FAM91A1,FER1L6,FER1L6,FER1L6-AS1,FER1L6,FER1L6-AS2,TMEM65,TRMT12,RNF139 | 1.36828 | 628.152 | 87 | 52.2173 |
| 3 | 4 | 1.89E+08 | 191025802 | FRG1,FRG2 | 1.36331 | 472.166 | 46 | 22.7294 |
| 3 | 8 | 1.16E+08 | 120759194 | TRPS1,EIF3H,UTP23,RAD21,RAD21,RAD21-AS1,MIR3610,RAD21-AS1,AARD,SLC30A8,MED30,EXT1,SAMD12,SAMD12,SAMD12-AS1,TNFRSF11B,COLEC10,LOC101927513,COLEC10,MAL2,MAL2,MAL2-AS1,NOV,ENPP2,TAF2 | 1.35801 | 597.826 | 203 | 123.045 |
| 3 | 17 | 64066645 | 65211391 | CEP112,APOH,PRKCA,PRKCA,MIR634,CACNG5,CACNG4,CACNG1,HELZ | 1.35201 | 617.796 | 107 | 63.9287 |
| 3 | 17 | 65353493 | 65717713 | PSMD12,PITPNC1,PITPNC1,MIR548AA2,MIR548D2,NOL11 | 1.35182 | 529.604 | 24 | 12.7176 |
| 3 | 22 | 36662612 | 37961889 | MYH9,TXN2,FOXRED2,EIF3D,CACNG2,LOC105373021,IFT27,IFT27,PVALB,NCF4-AS1,NCF4,NCF4,CSF2RB,TEX33,TST,TST,MPST,MPST,KCTD17,TMPRSS6,IL2RB,C1QTNF6,SSTR3,RAC2,CYTH4,ELFN2,MFNG,CARD10 | 1.31348 | 540.556 | 285 | 179.935 |
| 3 | 17 | 60679494 | 64001288 | TLK2,MRC2,MARCH10,MARCH10,LOC105371855,MIR633,TANC2,CYB561,ACE,KCNH6,DCAF7,TACO1,MAP3K3,LIMD2,STRADA,CCDC47,DDX42,FTSJ3,FTSJ3,PSMC5,PSMC5,SMARCD2,CSH2,GH2,CSH1,CSHL1,GH1,CD79B,SCN4A,PRR29-AS1,PRR29,PRR29,PRR29,ICAM2,ICAM2,ERN1,SNHG25,SNORD104,SNORA50C,TEX2,PECAM1,MILR1,POLG2,DDX5,DDX5,MIR3064,DDX5,MIR5047,DDX5,CEP95,CEP95,SMURF2,ARHGAP27P1-BPTFP1-KPNA2P3,ARHGAP27P1-BPTFP1-KPNA2P3,ARHGAP27P1,ARHGAP27P1-BPTFP1-KPNA2P3,ARHGAP27P1,MIR6080,PLEKHM1P1,PLEKHM1P1,MIR4315-1,MIR4315-2,LRRC37A3,AMZ2P1,GNA13,RGS9,AXIN2,CEP112 | 1.22561 | 577.093 | 628 | 378.518 |

| | | | | | | | | |
|---|---|---|---|---|---|---|---|---|
| 3 | 5 | 10500 | 20957651 | PLEKHG4B,LRRC14B,CCDC127,SDHA,PDCD6,PDCD6,AHRR,AHRR,EXOC3-AS1,EXOC3,PP7080,SLC9A3,SLC9A3,SLC9A3-AS1,SLC9A3,MIR4456,CEP72,TPPP,ZDHHC11B,ZDHHC11,BRD9,TRIP13,LOC100506688,NKD2,SLC12A7,SLC12A7,MIR4635,SLC6A19,SLC6A18,TERT,MIR4457,CLPTM1L,LINC01511,SLC6A3,LPCAT1,SDHAP3,LOC728613,MIR4277,MRPL36,NDUFS6,IRX4,IRX2,C5orf38,LINC01019,IRX1,ADAMTS16,ICE1,LINC02145,MED10,UBE2QL1,NSUN2,SRD5A1,PAPD7,MIR4278,LOC442132,ADCY2,C5orf49,FASTKD3,FASTKD3,MTRR,MTRR,MIR4458HG,MIR4458,SEMA5A,SEMA5A,MIR4636,SNHG18,SNORD123,TAS2R1,FAM173B,CCT5,CMBL,MARCH6,ROPN1L,LINC02213,ANKRD33B,DAP,CTNND2,DNAH5,TRIO,FAM105A,OTULIN,ANKH,ANKH,LOC100130744,ANKH,MIR4637,FBXL7,FBXL7,MIR887,MARCH11,ZNF622,RETREG1,RETREG1,LOC101929524,MYO10,BASP1,CDH18 | 1.0979 | 439.404 | 1236 | 731.9 |
| 3 | 17 | 67310452 | 74301097 | ABCA5,MAP2K6,KCNJ16,KCNJ2,SOX9,SLC39A11,SSTR2,COG1,COG1,FAM104A,FAM104A,FAM104A,C17orf80,C17orf80,C17orf80,CPSF4L,CPSF4L,CDC42EP4,SDK2,LOC100134391,LINC00469,LINC00469,RPL38,TTYH2,DNAI2,KIF19,BTBD17,GPR142,GPRC5C,CD300A,CD300LB,CD300C,CD300LD,CD300LD,C17orf77,C17orf77,CD300E,RAB37,RAB37,CD300LF,SLC9A3R1,MIR3615,SLC9A3R1,NAT9,TMEM104,GRIN2C,FDXR,FADS6,USH1G,OTOP2,OTOP3,HID1,HID1,HID1-AS1,CDR2L,MRPL58,KCTD2,KCTD2,ATP5PD,SLC16A5,ARMC7,NT5C,JPT1,SUMO2,NUP85,GGA3,GGA3,MRPS7,MRPS7,MIF4GD,LOC100287042,LOC100287042,SLC25A19,SLC25A19,GRB2,MIR3678,TMEM94,TMEM94,MIR6785,CASKIN2,TSEN54,LLGL2,MYO15B,RECQL5,RECQL5,SMIM5,RECQL5,SMIM6,SAP30BP,ITGB4,GALK1,H3F3B,MIR4738,UNK,UNC13D,WBP2,TRIM47,TRIM65,MRPL38,FBF1,ACOX1,TEN1,TEN1-CDK3,TEN1-CDK3,CDK3,EVPL,SRP68,GALR2,ZACN,ZACN,EXOC7,EXOC7,EXOC7,MIR6868,FOXJ1,RNF157-AS1,RNF157,RNF157,UBALD2,QRICH2 | 1.07802 | 477.166 | 1118 | 710.056 |
| 3 | 14 | 69707691 | 69908261 | EXD2,GALNT16,ERH,ERH,SLC39A9,SLC39A9 | 1.02321 | 401.148 | 27 | 16.0819 |
| 3 | 12 | 6976735 | 7080297 | TPI1,SPSB2,LRRC23,ENO2,ATN1,C12orf57,PTPN6,MIR200CHG,MIR200C,MIR200CHG,MIR141,PHB2,PHB2,SCARNA12,EMG1 | 1.02257 | 467.288 | 84 | 53.0781 |
| 3 | 17 | 65740394 | 67083611 | BPTF,C17orf58,KPNA2,LINC00674,ARHGAP27P2,AMZ2,ARSG,SLC16A6,ARSG,ARSG,PRKAR1A,ARSG,PRKAR1A,WIPI1,ARSG,PRKAR1A,WIPI1,MIR635,PRKAR1A,WIPI1,PRKAR1A,PRKAR1A,FAM20A,FAM20A,ABCA8,ABCA9,ABCA9,ABCA9-AS1,ABCA6 | 1.01808 | 541.075 | 269 | 157.019 |
| 3 | 14 | 49431910 | 50089388 | RPS29,RPS29,LRR1,LRR1,RPL36AL,MGAT2 | 1.00113 | 474.072 | 31 | 17.7566 |

| | | | | | | | | |
|---|---|---|---|---|---|---|---|---|
| 3 | 1 | 43888067 | 47748180 | SZT2,SZT2,SZT2-AS1,SZT2,SZT2-AS1,MIR6735,SZT2,HYI,PTPRF,KDM4A,KDM4A,KDM4A-AS1,ST3GAL3,ARTN,IPO13,DPH2,ATP6V0B,B4GALT2,CCDC24,SLC6A9,KLF17,DMAP1,ERI3,RNF220,RNF220,MIR5584,TMEM53,C1orf228,KIF2C,KIF2C,SNORD160,RPS8,RPS8,SNORD55,RPS8,SNORD46,RPS8,SNORD38A,RPS8,SNORD38B,BEST4,PLK3,PLK3,TCTEX1D4,TCTEX1D4,BTBD19,PTCH2,EIF2B3,HECTD3,UROD,ZSWIM5,HPDL,MUTYH,MUTYH,TOE1,TOE1,TESK2,CCDC163,MMACHC,PRDX1,AKR1A1,NASP,CCDC17,GPBP1L1,TMEM69,TMEM69,IPP,IPP,MAST2,PIK3R3,LOC110117498-PIK3R3,LOC110117498-PIK3R3,TSPAN1,LOC110117498,TSPAN1,POMGNT1,POMGNT1,LURAP1,RAD54L,LRRC41,UQCRH,NSUN4,FAAH,DMBX1,MKNK1-AS1,KNCN,MKNK1-AS1,MKNK1,MKNK1,MOB3C,ATPAF1,TEX38,EFCAB14-AS1,EFCAB14,EFCAB14,CYP4B1,CYP4Z2P,CYP4A11,CYP4X1,CYP4Z1,CYP4A22,PDZK1IP1,TAL1,STIL | 1.00067 | 473.435 | 935 | 581.784 |
| 3 | 16 | 2495391 | 2820900 | CCNF,MIR6767,CCNF,TEDC2,TEDC2,MIR6768,NTN3,TBC1D24,ATP6V0C,AMDHD2,AMDHD2,CEMP1,MIR3178,PDPK1,LOC652276,FLJ42627,ERVK13-1,KCTD5,PRSS27,SRRM2-AS1,SRRM2 | 0.984588 | 481.167 | 168 | 102.859 |
| 3 | 7 | 1.02E+08 | 102343262 | RASA4B,POLR2J3,RASA4,RASA4B,POLR2J2,UPK3BL1,POLR2J2 | 0.952605 | 357.503 | 38 | 13.4317 |
| 3 | 16 | 684539 | 779253 | METTL26,LOC100287175,MCRIP2,LOC105371038,WDR90,WDR90,RHOT2,RHBDL1,STUB1,STUB1,JMJD8,JMJD8,WDR24,FBXL16,METRN,FAM173A,FAM173A,CCDC78,CCDC78,HAGHL | 0.951667 | 476.405 | 143 | 96.0989 |

| | | | | | | | | |
|---|---|---|---|---|---|---|---|---|
| 3 | 22 | 21192891 | 36662112 | PI4KA,SNAP29,CRKL,LINC01637,AIFM3,LZTR1,THAP7,THAP7,THAP7-AS1,THAP7-AS1,TUBA3FP,P2RX6,SLC7A4,MIR649,LRRC74B,GGT2,HIC2,TMEM191C,PI4KAP2,UBE2L3,YDJC,CCDC116,SDF2L1,MIR301B,MIR130B,PPIL2,YPEL1,MAPK1,PPM1F,PPM1F,LOC100286925,TOP3B,PRAMENP,VPREB1,BMS1P20,ZNF280B,ZNF280A,PRAME,PRAME,LL22NC03-63E9.3,LL22NC03-63E9.3,GGTLC2,MIR5571,IGLL5,RSPH14,RSPH14,GNAZ,RAB36,BCR,IGLL1,DRICH1,GUSBP11,GUSBP11,RGL4,ZNF70,VPREB3,C22orf15,C22orf15,CHCHD10,CHCHD10,MMP11,SMARCB1,SMARCB1,DERL3,DERL3,SLC2A11,MIF-AS1,MIF,MIF-AS1,GSTT2B,GSTT2,DDTL,DDTL,DDT,DDT,GSTT2,GSTT2B,GSTTP1,LOC391322,GSTT1,GSTTP2,CABIN1,SUSD2,GGT5,SPECC1L,SPECC1L-ADORA2A,SPECC1L-ADORA2A,ADORA2A,ADORA2A-AS1,UPB1,GUCD1,GUCD1,SNRPD3,SNRPD3,GGT1,GGT1,LRRC75B,BCRP3,PIWIL3,SGSM1,TMEM211,KIAA1671,CRYBB3,CRYBB2,IGLL3P,LRP5L,CRYBB2P1,GRK3,MYO18B,SEZ6L,ASPHD2,ASPHD2,HPS4,HPS4,SRRD,SRRD,TFIP11,TFIP11,TPST2,TPST2,MIR548J,CRYBB1,CRYBA4,LOC284898,MN1,PITPNB,TTC28-AS1,MIR3199-1,MIR3199-2,TTC28-AS1,TTC28,TTC28,CHEK2,HSCB,CCDC117,XBP1,ZNRF3,C22orf31,KREMEN1,EMID1,RHBDD3,EWSR1,GAS2L1,RASL10A,AP1B1,AP1B1,MIR3653,SNORD125,RFPL1S,RFPL1S,RFPL1,NEFH,THOC5,NIPSNAP1,NF2,CABP7,CABP7,ZMAT5,ZMAT5,UQCR10,ASCC2,MTMR3,MTMR3,HORMAD2-AS1,HORMAD2,LIF,LIF,LOC91370,OSM,CASTOR1,TBC1D10A,SF3A1,SF3A1,CCDC157,CCDC157,CCDC157,KIAA1656,RNF215,SEC14L2,MTFP1,LOC105372990,SEC14L | 0.946075 | 427.209 | 2412 | 1434.49 |
| 3 | 6 | 1.37E+08 | 149826905 | MAP7,MAP3K5,MAP3K5,LOC101928461,PEX7,SLC35D3,IL20RA,IL22RA2,IFNGR1,OLIG3,TNFAIP3,PERP,ARFGEF3,ARFGEF3,PBOV1,HEBP2,NHSL1,NHSL1,MIR3145,FLJ46906,GVQW2,GVQW2,CCDC28A,CCDC28A,ECT2L,REPS1,ABRACL,HECA,TXLNB,CITED2,MIR3668,MIR4465,NMBR,NMBR,GJE1,VTA1,ADGRG6,HIVEP2,AIG1,ADAT2,PEX3,FUCA2,PHACTR2,LTV1,ZC2HC1B,PLAGL1,SF3B5,STX11,UTRN,EPM2A,EPM2A,LOC100507557,LOC100507557,LOC100507557,FBXO30,LOC100507557,SHPRH,SHPRH,GRM1,RAB32,ADGB,ADGB,KATNBL1P6,STXBP5-AS1,STXBP5,STXBP5,SAMD5,SASH1,UST,UST,UST-AS1,TAB2,TAB2,SUMO4,ZC3H12D,PPIL4 | 0.924459 | 435.218 | 938 | 566.953 |

| | | | | | | | | |
|---|---|---|---|---|---|---|---|---|
| 3 | 19 | 52671231 | 59118483 | ZNF836,PPP2R1A,ZNF766,ZNF766,MIR643,ZNF480,ZNF610,ZNF880,ZNF528-AS1,ZNF528,ZNF534,ZNF578,ZNF808,ZNF701,ZNF137P,ZNF83,ZNF611,ZNF600,ZNF28,ZNF468,ZNF320,ZNF888,ZNF321P,ZNF816-ZNF321P,ZNF816-ZNF321P,ZNF816,ERVV-1,ERVV-2,ZNF160,ZNF415,ZNF347,ZNF665,ZNF818P,ZNF677,VN1R2,VN1R4,FAM90A27P,BIRC8,ZNF845,ZNF525,ZNF765,ZNF765-ZNF761,ZNF765-ZNF761,ZNF761,ZNF813,ZNF331,LOC284379,DPRX,MIR512-2,MIR512-1,MIR1323,MIR498,MIR520E,MIR515-1,MIR515-2,MIR519E,MIR520F,MIR519C,MIR1283-1,MIR520A,MIR526B,MIR519B,MIR525,MIR523,MIR518F,MIR520B,MIR518B,MIR526A1,MIR520C,MIR518C,MIR524,MIR517A,MIR519D,MIR521-2,MIR520D,MIR517B,MIR520G,MIR516B2,MIR526A2,MIR518E,MIR518A1,MIR518D,MIR516B1,MIR518A2,MIR517C,MIR520H,MIR521-1,MIR522,MIR519A1,MIR527,MIR516A1,MIR1283-2,MIR516A2,MIR371A,MIR371B,MIR372,MIR373,NLRP12,MYADM,PRKCG,CACNG7,CACNG8,CACNG8,MIR935,CACNG6,VSTM1,TARM1,OSCAR,NDUFA3,TFPT,PRPF31,CNOT3,LENG1,TMC4,MBOAT7,TSEN34,RPS9,LILRB3,LILRB3,LILRA6,LILRB5,LILRB2,MIR4752,LILRA3,LILRA5,LILRA4,LAIR1,TTYH1,LENG8,LENG8,LENG9,LENG9,CDC42EP5,LAIR2,KIR3DX1,LILRA2,LILRA1,LILRB1,LILRB4,KIR3DL3,KIR2DL3,LOC101928804,KIR2DL1,KIR2DL1,KIR2DL4,KIR3DL1,KIR2DS4,KIR3DL2,FCAR,NCR1,NLRP7,NLRP2,GP6,RDH13,EPS8L1,PPP1R12C,TNNT1,TNNI3,DNAAF3,SYT5,PTPRH,TMEM86B,PPP6R1,MIR6804,PPP6R1,PPP6R1,MIR6802,PPP6R1,MIR6803,HSPBP1,BRSK1,TMEM150B,KMT5C,COX6B2,FAM71E2,IL11,TMEM190,TMEM238,RPL28,RPL28,MIR6805,UBE2S,SHISA7,ISOC2,Z | 0.906824 | 459.144 | 2205 | 1312.54 |
| 3 | 1 | 1.55E+08 | 155296977 | ADAR,KCNN3,PMVK,PBXIP1,PYGO2,PYGO2,LOC101928120,SHC1,CKS1B,CKS1B,MIR4258,FLAD1,LENEP,ZBTB7B,ZBTB7B,DCST2,DCST2,DCST1,DCST1,DCST1-AS1,DCST1-AS1,ADAM15,DCST1-AS1,EFNA4,EFNA4,EFNA3,EFNA1,SLC50A1,DPM3,KRTCAP2,TRIM46,MUC1,MIR92B,THBS3,MTX1,MTX1,GBAP1,GBAP1,GBA,FAM189B,SCAMP3,CLK2,HCN3,PKLR,FDPS,FDPS,RUSC1-AS1,RUSC1-AS1,RUSC1,RUSC1 | 0.8862 | 409.73 | 341 | 218.317 |
| 3 | 3 | 1.22E+08 | 124952521 | IQCB1,EAF2,SLC15A2,ILDR1,CD86,CASR,CSTA,CCDC58,FAM162A,WDR5B,LOC102723582,KPNA1,KPNA1,PARP9,PARP9,DTX3L,DTX3L,PARP15,PARP14,HSPBAP1,DIRC2,LINC02035,SEMA5B,PDIA5,PDIA5,MIR7110,SEC22A,ADCY5,HACD2,MYLK-AS1,MYLK,MYLK,CCDC14,ROPN1,KALRN,KALRN,MIR5002,UMPS,UMPS,MIR544B,ITGB5,MUC13,HEG1,SLC12A8,SLC12A8,MIR5092,ZNF148 | 0.873282 | 418.42 | 597 | 360.818 |
| 3 | 1 | 1.6E+08 | 160065017 | CFAP45,MIR4259,TAGLN2,IGSF9,SLAMF9,LINC01133,PIGM,KCNJ10,KCNJ9,IGSF8 | 0.853864 | 390.135 | 61 | 39.9334 |
| 3 | 3 | 1.21E+08 | 121509008 | STXBP5L,POLQ,ARGFX,FBXO40,HCLS1,GOLGB1,IQCB1 | 0.847094 | 470.35 | 154 | 98.0786 |

| | | | | | | | | |
|---|---|---|---|---|---|---|---|---|
| 3 | 1 | 53537109 | 55541217 | PODN,SLC1A7,CPT2,C1orf123,MAGOH,LRP8,DMRTB1,GLIS1,NDC1,YIPF1,DIO1,HSPB11,LRRC42,LDLRAD1,TMEM59,TCEANC2,MIR4781,TCEANC2,CDCP2,CYB5RL,MRPL37,SSBP3,SSBP3,SSBP3-AS1,ACOT11,ACOT11,FAM151A,MROH7,MROH7-TTC4,MROH7-TTC4,MROH7-TTC4,TTC4,PARS2,TTC22,LEXM,DHCR24,TMEM61,BSND,PCSK9,USP24 | 0.846995 | 420.7 | 370 | 224.49 |
| 3 | 16 | 31447604 | 31473707 | ZNF843,ARMC5 | 0.842392 | 477.932 | 10 | 6.54416 |
| 3 | 6 | 1.5E+08 | 171054567 | PPIL4,GINM1,KATNA1,LATS1,LATS1,LOC645967,NUP43,PCMT1,LRP11,LRP11,RAET1E-AS1,RAET1E-AS1,RAET1E,RAET1E-AS1,RAET1G,RAET1G,ULBP2,ULBP1,RAET1K,RAET1L,ULBP3,PPP1R14C,IYD,PLEKHG1,MTHFD1L,AKAP12,ZBTB2,RMND1,ARMT1,CCDC170,ESR1,ESR1,SYNE1,SYNE1,SYNE1,MIR3163,SYNE1,SYNE1-AS1,MYCT1,VIP,FBXO5,MTRF1L,RGS17,OPRM1,OPRM1,IPCEF1,IPCEF1,CNKSR3,SCAF8,MIR1273C,TIAM2,TIAM2,TFB1M,TFB1M,TFB1M,CLDN20,NOX3,MIR1202,ARID1B,ARID1B,MIR4466,TMEM242,ZDHHC14,ZDHHC14,MIR3692,SNX9,SYNJ2,SERAC1,GTF2H5,TULP4,TMEM181,DYNLT1,SYTL3,SYTL3,MIR3918,EZR,EZR,EZR-AS1,OSTCP1,C6orf99,RSPH3,TAGAP,LOC101929122,FNDC1,FNDC1,SOD2,SOD2,WTAP,SOD2,SOD2-OT1,SOD2,SOD2-OT1,ACAT2,ACAT2,ACAT2,TCP1,TCP1,TCP1,SNORA20,TCP1,SNORA29,MRPL18,PNLDC1,MAS1,IGF2R,SLC22A1,SLC22A2,SLC22A3,LPAL2,LPA,PLG,MAP3K4,AGPAT4,AGPAT4,AGPAT4-IT1,PRKN,PRKN,PACRG,PACRG,PACRG,PACRG-AS3,PACRG,PACRG-AS1,QKI,C6orf118,PDE10A,LINC00473,LINC00602,LINC00602,T,PRR18,SFT2D1,MPC1,RPS6KA2,RPS6KA2,MIR1913,RNASET2,MIR3939,FGFR1OP,CCR6,GPR31,TCP10L2,UNC93A,TTLL2,TCP10,LINC02538,LINC01558,AFDN-DT,AFDN,HGC6.3,KIF25,FRMD1,DACT2,SMOC2,LOC101929523,THBS2,THBS2,WDR27,WDR27,C6orf120,C6orf120,C6orf120,PHF10,PHF10,TCTE3,TCTE3,ERMARD,ERMARD,LINC00242,LINC00574,DLL1,FAM120B,FAM120B,MIR4644,PSMB1,TBP,PDCD2 | 0.836503 | 405.258 | 1950 | 1165.98 |
| 3 | 1 | 1.52E+08 | 152958056 | TCHHL1,TCHH,RPTN,HRNR,FLG,FLG,FLG-AS1,FLG-AS1,FLG2,CRNN,LCE5A,CRCT1,LCE3E,LCE3D,LCE3A,LCE2D,LCE2C,LCE2B,LCE2A,LCE4A,C1orf68,KPRP,LCE1F,LCE1E,LCE1C,LCE1B,LCE1A,LCE6A,SMCP,IVL,SPRR4,SPRR1A | 0.814184 | 688.954 | 189 | 104.346 |
| 3 | 22 | 39639810 | 40030066 | PDGFB,RPL3,RPL3,SNORD83B,RPL3,SNORD83A,RPL3,SNORD139,RPL3,SNORD43,SYNGR1,TAB1,TAB1,LOC100506472,MGAT3,MIEF1,ATF4,RPS19BP1,CACNA1I | 0.800906 | 387.461 | 85 | 47.5455 |
| 3 | 3 | 1.2E+08 | 120833279 | RABL3,GTF2E1,STXBP5L,STXBP5L,MIR5682 | 0.783293 | 383.542 | 21 | 12.4354 |

| | | | | | | | | |
|---|---|---|---|---|---|---|---|---|
| 3 | 7 | 1.44E+08 | 144098649 | LOC101928605,OR2A1-AS1,OR2A1,OR2A42,LOC101928605,OR2A1-AS1,OR2A9P,OR2A20P,LOC101928605,OR2A1-AS1,OR2A7,LOC101928605,OR2A1-AS1,OR2A7,ARHGEF34P,LOC101928605,OR2A1-AS1,ARHGEF34P,OR2A1-AS1,OR2A20P,OR2A9P,OR2A1-AS1,OR2A1,OR2A42,ARHGEF5,NOBOX | 0.754765 | 505.992 | 80 | 37.6469 |
| 3 | 6 | 31935512 | 32011932 | SKIV2L,DXO,DXO,STK19,STK19,C4A,C4B_2,LOC110384692,C4B,CYP21A2,CYP21A1P,CYP21A2,CYP21A1P,TNXA,TNXB,TNXA,TNXB,C4B_2,C4B,CYP21A2,CYP21A2,TNXB,TNXB | 0.739591 | 251.414 | 156 | 82.3865 |
| 3 | 16 | 2821263 | 3253680 | SRRM2,ELOB,ELOB,PRSS33,PRSS41,PRSS21,ZG16B,PRSS30P,PRSS22,FLYWCH2,FLYWCH1,KREMEN2,PAQR4,PAQR4,PKMYT1,PKMYT1,CLDN9,CLDN6,TNFRSF12A,HCFC1R1,HCFC1R1,THOC6,THOC6,BICDL2,BICDL2,LOC100128770,LOC100128770,MMP25,MMP25,MMP25-AS1,IL32,ZSCAN10,ZNF205-AS1,ZNF205-AS1,ZNF205,ZNF205,ZNF213,CASP16P | 0.731337 | 372.362 | 181 | 111.718 |
| 3 | 8 | 1.1E+08 | 116425755 | TMEM74,TRHR,NUDCD1,ENY2,PKHD1L1,EBAG9,SYBU,SYBU,LOC100132813,KCNV1,CSMD3,CSMD3,MIR2053 | 0.70697 | 398.073 | 287 | 175.63 |
| 3 | 16 | 60500 | 684066 | POLR3K,SNRNP25,RHBDF1,MPG,MPG,NPRL3,NPRL3,HBZ,HBM,HBA2,HBA1,HBQ1,LUC7L,FAM234A,FAM234A,RGS11,RGS11,ARHGDIG,PDIA2,AXIN1,MRPL28,TMEM8A,LOC100134368,NME4,DECR2,RAB11FIP3,CAPN15,MIR5587,CAPN15,MIR3176,CAPN15,PRR35,NHLRC4,PIGQ,RAB40C,WFIKKN1 | 0.683654 | 366.32 | 276 | 173.852 |
| 3 | 16 | 1918690 | 2493814 | HS3ST6,MSRB1,RPL3L,NDUFB10,RPS2,RPS2,SNORA10,RPS2,SNORA64,SNHG9,SNORA78,RNF151,TBL3,NOXO1,GFER,SYNGR3,ZNF598,NPW,SLC9A3R2,NTHL1,NTHL1,TSC2,TSC2,PKD1,PKD1,MIR1225,PKD1,LOC105371049,RAB26,SNHG19,SNORD60,TRAF7,TRAF7,CASKIN1,CASKIN1,MLST8,MLST8,BRICD5,BRICD5,PGP,E4F1,DNASE1L2,ECI1,RNPS1,LOC106660606,MIR3677,LOC106660606,MIR940,MIR4717,ABCA3,CCNF | 0.677774 | 377.518 | 329 | 207.353 |

| | | | | | | | | |
|---|---|---|---|---|---|---|---|---|
| 3 | 17 | 76128414 | 81194710 | TMC6,TMC8,TMC8,C17orf99,SYNGR2,TK1,AFMID,BIRC5,TMEM235,LINC01993,SOCS3,PGS1,PGS1,DNAH17,DNAH17,DNAH17,DNAH17-AS1,CYTH1,USP36,TIMP2,TIMP2,CEP295NL,LGALS3BP,CANT1,C1QTNF1-AS1,C1QTNF1,ENGASE,RBFOX3,MIR4739,LINC02078,ENPP7,CBX2,CBX8,CBX4,TBC1D16,CCDC40,GAA,EIF4A3,CARD14,SGSH,SLC26A11,RNF213,RNF213,LOC100294362,LOC100294362,ENDOV,ENDOV,ENDOV,MIR4730,NPTX1,RPTOR,CHMP6,BAIAP2-AS1,BAIAP2,AATK,AATK,MIR657,AATK,MIR3065,MIR338,AATK,MIR1250,AATK,PVALEF,PVALEF,CEP131,TEPSIN,LOC105371925,TEPSIN,NDUFAF8,SLC38A10,LINC00482,TMEM105,BAHCC1,BAHCC1,MIR4740,BAHCC1,MIR3186,ACTG1,FSCN2,FAAP100,NPLOC4,TSPAN10,PDE6G,OXLD1,CCDC137,ARL16,HGS,HGS,MIR6786,MRPL12,SLC25A10,GCGR,MCRIP1,PPP1R27,P4HB,ARHGDIA,ALYREF,ALYREF,ANAPC11,ANAPC11,PCYT2,NPB,PCYT2,SIRT7,MAFG,PYCR1,MYADML2,NOTUM,ASPSCR1,CENPX,LRRC45,RAC3,DCXR,RFNG,GPS1,DUS1L,FASN,CCDC57,SLC16A3,CSNK1D,CD7,SECTM1,TEX19,UTS2R,OGFOD3,OGFOD3,HEXDC,HEXDC,HEXDC,C17orf62,C17orf62,NARF,FOXK2,WDR45B,RAB40B,RAB40B,MIR4525,FN3KRP,FN3K,TBCD,TBCD,ZNF750,TBCD,B3GNTL1,B3GNTL1,METRNL | 0.675023 | 365.64 | 1432 | 892.9 |
| 3 | 1 | 1.55E+08 | 156900414 | RUSC1,ASH1L,ASH1L,MIR555,ASH1L,POU5F1P4,ASH1L,ASH1L-AS1,ASH1L-AS1,MSTO1,MSTO2P,YY1AP1,DAP3,MSTO2P,MSTO2P,GON4L,GON4L,SYT11,RIT1,KHDC4,KHDC4,SNORA80E,KHDC4,SCARNA4,RXFP4,ARHGEF2,ARHGEF2,MIR6738,SSR2,UBQLN4,LAMTOR2,RAB25,MEX3A,LMNA,SEMA4A,SLC25A44,PMF1,PMF1-BGLAP,PMF1-BGLAP,BGLAP,PAQR6,SMG5,SMG5,TMEM79,GLMP,VHLL,CCT3,CCT3,TSACC,TSACC,RHBG,C1orf61,C1orf61,MIR9-1,MEF2D,IQGAP3,TTC24,NAXE,GPATCH4,HAPLN2,BCAN,NES,CRABP2,ISG20L2,RRNAD1,MRPL24,HDGF,PRCC,SH2D2A,SH2D2A,NTRK1,NTRK1,INSRR,NTRK1,PEAR1,LRRC71 | 0.669163 | 377.263 | 695 | 430.605 |
| 3 | 17 | 59115213 | 60678248 | BCAS3,TBX2,C17orf82,TBX4,NACA2,BRIP1,INTS2,MED13,TBC1D3P2,EFCAB3,METTL2A,TLK2 | 0.666596 | 453.192 | 221 | 126.433 |
| 3 | 11 | 90602245 | 92881256 | DISC1FP1,MIR1261,FAT3,MTNR1B | 0.662644 | 371.206 | 92 | 61.1172 |
| 3 | 3 | 98220155 | 99568630 | CLDND1,GPR15,CPOX,ST3GAL6,DCBLD2,MIR548G,COL8A1,MIR548G,HP09053,CMSS1,MIR548G,CMSS1,MIR548G,CMSS1,FILIP1L | 0.660878 | 359.207 | 90 | 54.8987 |
| 3 | 16 | 4415386 | 5516709 | CORO7-PAM16,CORO7,CORO7-PAM16,CORO7,VASN,DNAJA3,NMRAL1,HMOX2,CDIP1,C16orf96,UBALD1,MGRN1,MGRN1,MIR6769A,NUDT16L1,ANKS3,C16orf71,C16orf71,ZNF500,ZNF500,SEPT12,SMIM22,ROGDI,GLYR1,UBN1,PPL,SEC14L5,NAGPA,NAGPA,NAGPA-AS1,C16orf89,ALG1 | 0.655566 | 349.301 | 283 | 179.767 |

| | | | | | | | | |
|---|---|---|---|---|---|---|---|---|
| 3 | 1 | 1.61E+08 | 162470887 | F11R,TSTD1,USF1,ARHGAP30,NECTIN4,KLHDC9,PFDN2,NIT1,NIT1,DEDD,DEDD,UFC1,USP21,PPOX,B4GALT3,ADAMTS4,NDUFS2,FCER1G,APOA2,TOMM40L,TOMM40L,MIR5187,TOMM40L,NR1I3,NR1I3,PCP4L1,MPZ,SDHC,CFAP126,FCGR2A,HSPA6,FCGR3A,FCGR2C,HSPA7,FCGR3B,FCGR2B,FCRLA,FCRLB,DUSP12,ATF6,OLFML2B,NOS1AP,NOS1AP,MIR4654,NOS1AP,MIR556,SPATA46,C1orf226,SH2D1B,UHMK1 | 0.655141 | 369.696 | 360 | 215.83 |
| 3 | 5 | 1.71E+08 | 180904760 | FGF18,SMIM23,FBXW11,STK10,EFCAB9,UBTD2,SH3PXD2B,NEURL1B,NEURL1B,MIR5003,DUSP1,ERGIC1,LOC100268168,RPL26L1,ATP6V0E1,ATP6V0E1,SNORA74B,CREBRF,BNIP1,NKX2-5,STC2,BOD1,CPEB4,C5orf47,NSG2,MSX2,MIR4634,DRD1,SFXN1,HRH2,CPLX2,THOC3,LOC100996385,FAM153B,SIMC1,KIAA1191,ARL10,ARL10,MIR1271,NOP16,NOP16,HIGD2A,HIGD2A,CLTB,FAF2,RNF44,CDHR2,GPRIN1,SNCB,SNCB,MIR4281,EIF4E1B,TSPAN17,UNC5A,HK3,UIMC1,ZNF346,FGFR4,NSD1,RAB24,PRELID1,PRELID1,MXD3,MXD3,LMAN2,RGS14,SLC34A1,PFN3,F12,GRK6,GRK6,PRR7-AS1,PRR7,DBN1,PDLIM7,DOK3,DDX41,FAM193B,TMED9,B4GALT7,LOC202181,FAM153A,LOC728554,PROP1,FAM153C,N4BP3,RMND5B,NHP2,GMCL2,GMCL1P1,HNRNPAB,HNRNPAB,PHYKPL,PHYKPL,COL23A1,CLK4,ZNF354A,ZNF354B,ZFP2,ZNF454,GRM6,ZNF879,ZNF354C,ADAMTS2,RUFY1,RUFY1,LOC101928445,HNRNPH1,C5orf60,CBY3,CANX,MAML1,LTC4S,MGAT4B,MIR1229,MGAT4B,MGAT4B,SQSTM1,SQSTM1,SQSTM1,MRNIP,MRNIP,LOC100996419,TBC1D9B,RNF130,RNF130,MIR340,RASGEF1C,MAPK9,GFPT2,CNOT6,SCGB3A1,FLT4,OR2Y1,MGAT1,ZFP62,BTNL8,BTNL3,BTNL9,OR2V1,OR2V2,TRIM7,MIR4638,TRIM41,RACK1,RACK1,SNORD96A,RACK1,SNORD95,CTC-338M12.4,CTC-338M12.4,TRIM52,TRIM52 | 0.652474 | 348.016 | 1467 | 882.113 |
| 3 | 16 | 779255 | 1884403 | HAGHL,NARFL,MSLN,MIR662,RPUSD1,CHTF18,GNG13,PRR25,LMF1,LMF1,LMF1-AS1,SOX8,SSTR5-AS1,SSTR5,C1QTNF8,CACNA1H,CACNA1H,TPSG1,TPSG1,TPSB2,TPSAB1,TPSD1,UBE2I,BAIAP3,BAIAP3,TSR3,TSR3,GNPTG,UNKL,C16orf91,CCDC154,CLCN7,PTX4,TELO2,IFT140,IFT140,TMEM204,IFT140,LOC105371046,CRAMP1,JPT2,MAPK8IP3,MAPK8IP3,MIR3177,NME3,MRPS34,MRPS34,EME2,EME2,SPSB3,NUBP2,IGFALS,HAGH,FAHD1,FAHD1,MEIOB | 0.644421 | 366.83 | 532 | 335.933 |
| 3 | 11 | 43964882 | 47493294 | C11orf96,ACCSL,ACCS,EXT2,ALX4,CD82,TSPAN18,TP53I11,PRDM11,SYT13,CHST1,DKFZp779M0652,SLC35C1,CRY2,MAPK8IP1,C11orf94,PEX16,LARGE2,PHF21A,CREB3L1,DGKZ,DGKZ,MIR4688,MDK,CHRM4,AMBRA1,AMBRA1,MIR3160-1,MIR3160-2,HARBI1,ATG13,ARHGAP1,ZNF408,F2,CKAP5,CKAP5,MIR5582,CKAP5,SNORD67,LRP4-AS1,LRP4,LRP4,C11orf49,ARFGAP2,PACSIN3,PACSIN3,MIR6745,DDB2,ACP2,ACP2,NR1H3,NR1H3,MADD,MYBPC3,SPI1,SLC39A13,PSMC3,RAPSN | 0.641357 | 346.64 | 662 | 418.521 |

| | | | | | | | | |
|---|---|---|---|---|---|---|---|---|
| 3 | 3 | 1.59E+08 | 159975745 | IQCJ,IQCJ-SCHIP1,IQCJ-SCHIP1,SCHIP1,IL12A-AS1,IL12A,IL12A-AS1,IL12A-AS1,LINC01100,C3orf80 | 0.641295 | 268.743 | 39 | 23.5343 |
| 3 | 14 | 94741693 | 96756205 | PPP4R4,SERPINA10,SERPINA6,SERPINA2,SERPINA1,SERPINA11,SERPINA9,SERPINA12,SERPINA4,SERPINA5,SERPINA3,SERPINA13P,GSC,DICER1,DICER1,MIR3173,CLMN,SYNE3,SNHG10,SCARNA13,GLRX5,TCL6,TCL1B,TCL1A,C14orf132,BDKRB2,BDKRB1,ATG2B | 0.626312 | 328.988 | 220 | 141.358 |
| 3 | 3 | 1.25E+08 | 127410532 | ZNF148,SNX4,OSBPL11,ALG1L,ROPN1B,SLC41A3,ALDH1L1,ALDH1L1-AS1,ALDH1L1,ALDH1L1,ALDH1L1-AS2,KLF15,CFAP100,ZXDC,UROC1,CHST13,CHST13,C3orf22,C3orf22,TXNRD3NB,TXNRD3NB,TXNRD3,TXNRD3,NUP210P1,CHCHD6,PLXNA1,C3orf56,LINC02016,TPRA1,TPRA1,MIR6825,MCM2,PODXL2,ABTB1 | 0.623665 | 365.24 | 327 | 198.538 |
| 3 | 19 | 51608404 | 52663952 | SIGLEC9,SIGLEC7,SIGLEC7,LOC101928517,LOC101928517,SIGLEC17P,CD33,SIGLECL1,IGLON5,VSIG10L,ETFB,CLDND2,NKG7,LIM2,C19orf84,SIGLEC10,SIGLEC10,LOC100129083,SIGLEC8,CEACAM18,SIGLEC12,SIGLEC6,ZNF175,LINC01530,SIGLEC5,SIGLEC14,SPACA6P-AS,SPACA6,MIR99B,MIRLET7E,SPACA6P-AS,SPACA6,MIR125A,SPACA6,HAS1,FPR1,FPR2,FPR3,ZNF577,ZNF649-AS1,ZNF649,ZNF613,ZNF350-AS1,ZNF350,ZNF615,ZNF614,ZNF432,ZNF841,ZNF616,ZNF836 | 0.619035 | 364.657 | 349 | 209.118 |
| 3 | 12 | 7083475 | 7168637 | EMG1,EMG1,LPCAT3,LPCAT3 | 0.608031 | 288.456 | 21 | 13.0179 |
| 3 | 20 | 24646659 | 25398859 | CST7,APMAP,ACSS1,VSX1,LOC101926889,ENTPD6,ENTPD6,PYGB,PYGB,ABHD12,ABHD12,GINS1 | 0.596289 | 317.241 | 111 | 67.5568 |
| 3 | 1 | 1.46E+08 | 149783905 | NBPF20,NBPF19,NBPF10,NBPF25P,NBPF19,LOC728989,NBPF19,PRKAB2,NBPF19,CHD1L,PDIA3P1,NBPF19,CHD1L,FMO5,NBPF19,CHD1L,NBPF19,BCL9,NBPF19,ACP6,NBPF19,GJA5,NBPF19,GJA8,NBPF19,GPR89B,NBPF19,PDE4DIPP1,NBPF19,MIR5087,NBPF25P,FAM231D,LOC388692,LOC100996741,FCGR1CP,HIST2H3PS2,LOC101929798,HIST2H2BF,FCGR1A,HIST2H2BF | 0.572 | 315.517 | 155 | 86.475 |

| | | | | | | | | |
|---|---|---|---|---|---|---|---|---|
| 3 | 11 | 67435013 | 79442211 | FAM86C2P,UNC93B1,ALDH3B1,NDUFS8,NDUFS8,MIR7113,NDUFS8,MIR4691,TCIRG1,TCIRG1,MIR6753,CHKA,KMT5B,C11orf24,LRP5,PPP6R3,GAL,TESMIN,CPT1A,MRPL21,MRPL21,IGHMBP2,IGHMBP2,MRGPRD,MRGPRF,TPCN2,TPCN2,MIR3164,MYEOV,CCND1,ORAOV1,FGF19,FGF4,FGF3,ANO1,FADD,PPFIA1,PPFIA1,MIR548K,CTTN,SHANK2,SHANK2,SHANK2-AS3,SHANK2,MIR3664,DHCR7,NADSYN1,KRTAP5-7,KRTAP5-8,KRTAP5-9,KRTAP5-10,KRTAP5-11,FAM86C1,ALG1L9P,LOC100133315,RNF121,IL18BP,NUMA1,NUMA1,LOC100128494,NUMA1,MIR3165,LRTOMT,LRTOMT,LAMTOR1,LRTOMT,ANAPC15,ANAPC15,FOLR3,FOLR1,FOLR2,INPPL1,PHOX2A,CLPB,LINC01537,PDE2A,PDE2A,MIR139,ARAP1,ARAP1,ARAP1-AS2,STARD10,STARD10,MIR4692,ATG16L2,FCHSD2,FCHSD2,MIR4459,P2RY2,P2RY6,LOC100287837,ARHGEF17,ARHGEF17,RELT,FAM168A,PLEKHB1,RAB6A,MRPL48,COA4,PAAF1,DNAJB13,UCP2,UCP3,C2CD3,PPME1,P4HA3,P4HA3,P4HA3-AS1,PGM2L1,MIR548AL,KCNE3,LIPT2,LIPT2,LOC100287896,LOC100287896,POLD3,CHRDL2,CHRDL2,MIR4696,RNF169,XRRA1,SPCS2,NEU3,OR2AT4,SLCO2B1,TPBGL,ARRB1,ARRB1,MIR326,RPS3,RPS3,SNORD15A,RPS3,SNORD15B,KLHL35,GDPD5,SERPINH1,LOC105369391,MAP6,MAP6,MOGAT2,DGAT2,UVRAG,WNT11,THAP12,GVQW3,EMSY,LRRC32,GUCY2EP,TSKU,ACER3,B3GNT6,CAPN5,CAPN5,OMP,MYO7A,GDPD4,PAK1,AQP11,CLNS1A,RSF1,AAMDC,AAMDC,INTS4,INTS4,KCTD14,NDUFC2-KCTD14,NDUFC2-KCTD14,THRSP,NDUFC2-KCTD14,NDUFC2,ALG8,KCTD21-AS1,KCTD21,KCTD21,USP35,GAB2,NARS2,TENM4,TENM4,MIR708,TENM4,MIR5579 | 0.571256 | 322.333 | 1830 | 1104.7 |
| 3 | 3 | 49020247 | 49201648 | ARIH2,P4HTM,WDR6,WDR6,DALRD3,DALRD3,DALRD3,MIR425,DALRD3,NDUFAF3,MIR191,NDUFAF3,IMPDH2,QRICH1,QARS,QARS,MIR6890,USP19,LAMB2,CCDC71 | 0.570411 | 336.11 | 155 | 108.189 |
| 3 | 1 | 33585599 | 35450977 | AZIN2,TRIM62,ZNF362,A3GALT2,PHC2,PHC2,MIR3605,PHC2,LOC101929464,ZSCAN20,CSMD2,CSMD2,HMGB4,C1orf94,MIR552,GJB5,GJB4,GJB3,GJA4,SMIM12,DLGAP3,LOC653160,TMEM35B | 0.569837 | 333.043 | 228 | 141.227 |
| 3 | 1 | 1.5E+08 | 150667365 | BOLA1,SV2A,SF3B4,MTMR11,OTUD7B,VPS45,PLEKHO1,ANP32E,CA14,APH1A,C1orf54,CIART,MRPS21,PRPF3,RPRD2,TARS2,TARS2,MIR6878,ECM1,ADAMTSL4,ADAMTSL4,MIR4257,ADAMTSL4-AS1,MCL1,ENSA,GOLPH3L | 0.567318 | 332.335 | 266 | 161.297 |

| | | | | | | | | |
|---|---|---|---|---|---|---|---|---|
| 3 | 14 | 97348071 | 107283157 | LINC00618,LINC02291,LINC02312,LINC01550,C14orf177,BCL11B,SETD3,CCNK,CCDC85C,HHIPL1,CYP46A1,EML1,EVL,EVL,MIR151B,MIR342,DEGS2,YY1,YY1,MIR6764,SLC25A29,MIR345,SLC25A47,WARS,WDR25,BEGAIN,LINC00523,DLK1,MIR2392,MEG3,MEG3,MIR770,MIR493,MIR337,MIR665,RTL1,RTL1,MIR431,RTL1,MIR433,RTL1,MIR127,RTL1,MIR432,RTL1,MIR136,MEG8,MEG8,MIR370,MEG8,SNORD113-1,MEG8,SNORD113-2,MEG8,SNORD113-4,MEG8,SNORD113-5,MEG8,SNORD113-6,MEG8,SNORD113-7,MEG8,SNORD113-9,MEG8,SNORD114-1,MEG8,SNORD114-2,MEG8,SNORD114-3,MEG8,SNORD114-4,MEG8,SNORD114-5,MEG8,SNORD114-6,MEG8,SNORD114-7,MEG8,SNORD114-8,MEG8,SNORD114-9,MEG8,SNORD114-10,MEG8,SNORD114-11,MEG8,SNORD114-12,MEG8,SNORD114-13,MEG8,SNORD114-14,MEG8,SNORD114-15,MEG8,SNORD114-16,MEG8,SNORD114-17,MEG8,SNORD114-18,MEG8,SNORD114-19,MEG8,SNORD114-20,MEG8,SNORD114-21,MEG8,SNORD114-22,MEG8,SNORD114-23,MEG8,SNORD114-24,MEG8,SNORD114-25,MEG8,SNORD114-26,MEG8,SNORD114-27,MEG8,SNORD114-28,MEG8,SNORD114-29,MEG8,SNORD114-30,MEG8,SNORD114-31,MIR379,MIR411,MIR299,MIR380,MIR1197,MIR323A,MIR758,MIR494,MIR1193,MIR543,MIR495,MIR376C,MIR376A2,MIR654,MIR376B,MIR300,MIR1185-1,MIR1185-2,MIR381HG,MIR381,MIR381HG,MIR487B,MIR381HG,MIR539,MIR381HG,MIR889,MIR381HG,MIR544A,MIR381HG,MIR655,MIR487A,MIR382,MIR134,MIR668,MI | 0.56655 | 344.794 | 1562 | 925.052 |

| | | | | | | | | |
|---|---|---|---|---|---|---|---|---|
| 3 | 1 | 35881001 | 43887895 | ZMYM4,KIAA0319L,NCDN,TFAP2E,PSMB2,C1orf216,CLSPN,AGO4,AGO1,AGO3,TEKT2,ADPRHL2,COL8A2,TRAPPC3,MAP7D1,THRAP3,SH3D21,EVA1B,STK40,LSM10,OSCP1,MRPS15,CSF3R,GRIK3,MIR4255,ZC3H12A,ZC3H12A,MIR6732,MEAF6,MEAF6,MIR5581,SNIP1,DNALI1,GNL2,RSPO1,C1orf109,CDCA8,EPHA10,MANEAL,YRDC,YRDC,C1orf122,C1orf122,MTF1,INPP5B,SF3A3,FHL3,UTP11,POU3F1,MIR3659,LINC01343,RRAGC,MYCBP,GJA9-MYCBP,MYCBP,GJA9-MYCBP,LOC105378663,GJA9-MYCBP,LOC105378663,GJA9-MYCBP,LOC105378663,GJA9,GJA9-MYCBP,GJA9,RHBDL2,AKIRIN1,NDUFS5,MACF1,MACF1,KIAA0754,BMP8A,BMP8A,OXCT2P1,BMP8A,PPIEL,PABPC4,PABPC4,LOC101929516,PABPC4,LOC101929516,SNORA55,HEYL,NT5C1A,HPCAL4,PPIE,PPIE,BMP8B,BMP8B,BMP8B,OXCT2,TRIT1,MYCL,MFSD2A,CAP1,PPT1,RLF,TMCO2,ZMPSTE24,COL9A2,SMAP2,ZFP69B,ZFP69,EXO5,ZNF684,RIMS3,NFYC-AS1,NFYC,NFYC,NFYC,MIR30E,NFYC,MIR30C1,KCNQ4,CITED4,CTPS1,SLFNL1-AS1,SLFNL1,SLFNL1-AS1,SCMH1,SCMH1,FOXO6,EDN2,HIVEP3,GUCA2B,GUCA2A,FOXJ3,RIMKLA,ZMYND12,CCDC30,PPCS,CCDC30,PPIH,YBX1,CLDN19,P3H1,C1orf50,TMEM269,SVBP,SVBP,ERMAP,ERMAP,ZNF691,SLC2A1,FAM183A,EBNA1BP2,EBNA1BP2,MIR6733,EBNA1BP2,CFAP57,CFAP57,TMEM125,C1orf210,TIE1,MPL,CDC20,ELOVL1,ELOVL1,MIR6734,MED8,SZT2 | 0.545047 | 325.133 | 1482 | 915.241 |
| 3 | 1 | 1.51E+08 | 152057648 | CTSS,CTSK,ARNT,SETDB1,CERS2,ANXA9,MINDY1,PRUNE1,BNIPL,C1orf56,CDC42SE1,MLLT11,GABPB2,SEMA6C,TNFAIP8L2,TNFAIP8L2-SCNM1,TNFAIP8L2-SCNM1,LYSMD1,TNFAIP8L2-SCNM1,SCNM1,TNFAIP8L2-SCNM1,SCNM1,TMOD4,TMOD4,VPS72,PIP5K1A,PSMD4,LOC100507670,LOC100507670,ZNF687,ZNF687,PI4KB,RFX5,SELENBP1,PSMB4,POGZ,CGN,TUFT1,TUFT1,MIR554,SNX27,CELF3,RIIAD1,MRPL9,MRPL9,OAZ3,OAZ3,TDRKH,LINGO4,RORC,C2CD4D,C2CD4D,LOC100132111,THEM5,THEM4,S100A10,S100A11,TCHHL1 | 0.538942 | 317.746 | 497 | 305.823 |
| 3 | 12 | 6948409 | 6975316 | P3H3,GNB3,GNB3,CDCA3,CDCA3,USP5 | 0.536872 | 338.478 | 37 | 23.2371 |
| 3 | 1 | 47753210 | 53535854 | STIL,CMPK1,LINC01389,FOXE3,FOXD2,TRABD2B,SLC5A9,SPATA6,AGBL4,AGBL4,BEND5,ELAVL4,DMRTA2,FAF1,CDKN2C,MIR4421,C1orf185,RNF11,TTC39A,TTC39A,TTC39A-AS1,EPS15,OSBPL9,NRDC,NRDC,MIR761,RAB3B,TXNDC12,TXNDC12,KTI12,TXNDC12,TXNDC12-AS1,BTF3L4,ZFYVE9,CC2D1B,ORC1,PRPF38A,ZCCHC11,GPX7,SHISAL2A,COA7,ZYG11B,ZYG11A,ECHDC2,SCP2,SCP2,MIR1273F,SCP2,MIR1273G,PODN | 0.533161 | 343.167 | 597 | 344.978 |

| | | | | | | | | |
|---|---|---|---|---|---|---|---|---|
| 3 | 1 | 2.01E+08 | 203769358 | CAMSAP2,GPR25,C1orf106,KIF21B,CACNA1S,TMEM9,IGFN1,PKP1,TNNT2,LAD1,TNNI1,PHLDA3,CSRP1,NAV1,NAV1,IPO9-AS1,NAV1,IPO9-AS1,MIR5191,NAV1,IPO9-AS1,MIR1231,IPO9-AS1,IPO9,IPO9,SHISA4,LMOD1,TIMM17A,RNPEP,RNPEP,ELF3-AS1,ELF3,GPR37L1,ARL8A,PTPN7,PTPRVP,LGR6,UBE2T,PPP1R12B,SYT2,KDM5B,MGAT4EP,RABIF,KLHL12,ADIPOR1,CYB5R1,LOC100506747,TMEM183A,TMEM183B,PPFIA4,MYOG,ADORA1,MYBPH,CHI3L1,CHIT1,BTG2,FMOD,PRELP,OPTC,ATP2B4,ATP2B4,SNORA77,LAX1,ZC3H11A,ZBED6 | 0.519475 | 319.637 | 732 | 446.996 |
| 3 | 1 | 1.53E+08 | 154574704 | SPRR3,SPRR1B,SPRR2C,SPRR2G,LELP1,PRR9,LOR,PGLYRP3,PGLYRP4,S100A9,S100A12,S100A8,S100A8,S100A7A,S100A7L2,S100A7,S100A6,S100A5,S100A4,LOC101928034,S100A3,S100A2,S100A16,S100A14,S100A13,S100A13,S100A1,CHTOP,SNAPIN,ILF2,NPR1,INTS3,SLC27A3,GATAD2B,DENND4B,CRTC2,SLC39A1,SLC39A1,MIR6737,CREB3L4,JTB,RAB13,RPS27,NUP210L,NUP210L,MIR5698,TPM3,MIR190B,C1orf189,C1orf43,C1orf43,UBAP2L,UBAP2L,HAX1,AQP10,ATP8B2,LOC101928101,IL6R,IL6R,SHE,TDRD10,UBE2Q1,UBE2Q1,UBE2Q1-AS1,CHRNB2,ADAR | 0.517875 | 322.864 | 511 | 310.874 |
| 3 | 1 | 2.47E+08 | 249240121 | ZNF669,C1orf229,ZNF124,MIR3916,VN1R5,ZNF496,NLRP3,OR2B11,OR2W5,GCSAML,GCSAML-AS1,GCSAML,OR2C3,GCSAML,OR2G2,OR2G3,OR13G1,OR6F1,OR14A2,OR14A2,OR14K1,OR1C1,OR14A16,OR11L1,TRIM58,OR2W3,OR2T8,OR2AJ1,OR2L13,OR2L8,OR2L13,OR2AK2,OR2L13,OR2L1P,OR2L13,OR2L13,OR2L5,OR2L13,OR2L2,OR2L13,OR2L3,OR2M1P,OR2M5,OR2M2,OR2M3,OR2M4,OR2T33,OR2T12,OR2M7,OR14C36,OR2T4,OR2T6,OR2T1,OR2T7,OR2T2,OR2T3,OR2T5,OR2G6,OR2T29,OR2T34,OR2T10,OR2T11,OR2T35,OR2T27,OR14I1,LYPD8,SH3BP5L,MIR3124,ZNF672,ZNF692,PGBD2 | 0.511587 | 405.784 | 330 | 190.877 |
| 3 | 11 | 32862301 | 36615728 | PRRG4,QSER1,DEPDC7,TCP11L1,CSTF3,HIPK3,KIAA1549L,C11orf91,CD59,FBXO3,LMO2,CAPRIN1,NAT10,ABTB2,CAT,ELF5,EHF,APIP,APIP,PDHX,PDHX,PDHX,MIR1343,CD44,SLC1A2,PAMR1,FJX1,TRIM44,LDLRAD3,MIR3973,LDLRAD3,COMMD9,PRR5L,TRAF6,RAG1,RAG2 | 0.507498 | 340.445 | 431 | 268.504 |
| 3 | 14 | 89343593 | 91925161 | TTC8,FOXN3,FOXN3,FOXN3-AS1,EFCAB11,TDP1,KCNK13,PSMC1,NRDE2,CALM1,TTC7B,TTC7B,LOC101928909,TTC7B,LOC105370622,RPS6KA5,RPS6KA5,DGLUCY,DGLUCY,SNORA11B,DGLUCY,GPR68,CCDC88C,PPP4R3A | 0.506285 | 278.231 | 265 | 157.449 |
| 3 | 3 | 50155700 | 50677215 | RBM5,SEMA3F-AS1,SEMA3F-AS1,SEMA3F,SEMA3F,GNAT1,SLC38A3,GNAI2,SEMA3B,SEMA3B,MIR6872,LSMEM2,IFRD2,HYAL3,HYAL3,NAT6,HYAL1,HYAL2,TUSC2,RASSF1,RASSF1,RASSF1-AS1,ZMYND10,NPRL2,NPRL2,CYB561D2,CYB561D2,CYB561D2,TMEM115,CYB561D2,CACNA2D2,CACNA2D2,C3orf18,HEMK1,CISH,MAPKAPK3 | 0.502712 | 305.346 | 210 | 135.61 |
| 3 | 21 | 38567913 | 39470868 | TTC3,DSCR9,DSCR3,DYRK1A,KCNJ6,DSCR4 | 0.501009 | 307.661 | 50 | 30.9949 |

| | | | | | | | | |
|---|---|---|---|---|---|---|---|---|
| 3 | 14 | 92628696 | 94704007 | SLC24A4,RIN3,LGMN,GOLGA5,CHGA,ITPK1,MOAP1,TMEM251,GON7,UBR7,BTBD7,UNC79,COX8C,UNC79,PRIMA1,FAM181A-AS1,FAM181A,FAM181A,ASB2,ASB2,MIR4506,CCDC197,OTUB2,DDX24,IFI27L1,IFI27,IFI27L2,PPP4R4 | 0.49542 | 312.301 | 331 | 196.706 |
| 3 | 1 | 2.04E+08 | 210001573 | LINC00303,SOX13,ETNK2,ETNK2,ERLNC1,REN,KISS1,GOLT1A,PLEKHA6,PPP1R15B,PIK3C2B,MDM4,LRRN2,NFASC,CNTN2,TMEM81,RBBP5,DSTYK,TMCC2,NUAK2,KLHDC8A,LEMD1-AS1,LEMD1,LEMD1,BLACAT1,MIR135B,CDK18,MFSD4A,ELK4,SLC45A3,NUCKS1,RAB29,SLC41A1,PM20D1,LOC284581,SLC26A9,AVPR1B,RHEX,CTSE,SRGAP2,SRGAP2,SRGAP2D,IKBKE,IKBKE,MIR6769B,RASSF5,EIF2D,DYRK3,MAPKAPK2,IL10,IL19,IL20,IL24,FCMR,PIGR,FCAMR,C1orf116,YOD1,PFKFB2,C4BPB,C4BPA,CD55,CR2,CR1,CR1L,CD46,MIR29B2CHG,MIR29C,MIR29B2CHG,MIR29B2,MIR29B2CHG,MIR29B2CHG,LOC148696,CD34,PLXNA2,MIR205HG,MIR205HG,MIR205,CAMK1G,LAMB3,LAMB3,MIR4260,LOC101930114,G0S2,LOC101930114,HSD11B1,TRAF3IP3,TRAF3IP3,C1orf74,C1orf74,IRF6,DIEXF | 0.493582 | 320.958 | 936 | 570.729 |
| 3 | 11 | 65123258 | 65715309 | TIGD3,SLC25A45,FRMD8,NEAT1,MIR612,MALAT1,MALAT1,TALAM1,MALAT1,TALAM1,MASCRNA,SCYL1,SCYL1,LTBP3,LTBP3,SSSCA1-AS1,SSSCA1,FAM89B,EHBP1L1,KCNK7,MAP3K11,PCNX3,PCNX3,MIR4690,SIPA1,SIPA1,MIR4489,RELA,KAT5,KAT5,RNASEH2C,RNASEH2C,AP5B1,OVOL1,SNX32,CFL1,MUS81,MUS81,EFEMP2,EFEMP2,CTSW,FIBP,CCDC85B,FOSL1,C11orf68,DRAP1,TSGA10IP | 0.490817 | 310.777 | 353 | 226.176 |
| 3 | 1 | 1.85E+08 | 186306323 | SWT1,IVNS1ABP,HMCN1,PRG4,PRG4,TPR,TPR | 0.489883 | 365.405 | 181 | 113.939 |
| 3 | 1 | 2.26E+08 | 227247152 | LIN9,PARP1,STUM,ITPKB,PSEN2,COQ8A,CDC42BPA | 0.4878 | 331.524 | 114 | 68.4203 |
| 3 | 3 | 49568307 | 49999492 | DAG1,BSN,APEH,MST1,RNF123,RNF123,AMIGO3,RNF123,GMPPB,GMPPB,IP6K1,CDHR4,FAM212A,UBA7,UBA7,MIR5193,TRAIP,CAMKV,MST1R,MON1A,RBM6 | 0.487059 | 321.459 | 253 | 171.949 |
| 3 | 8 | 1.21E+08 | 120862796 | TAF2,DSCC1 | 0.486462 | 401.693 | 33 | 19.2116 |
| 3 | 1 | 91870345 | 92427772 | HFM1,CDC7,TGFBR3 | 0.479359 | 347.836 | 49 | 28.6007 |
| 3 | 1 | 2.26E+08 | 226420319 | ENAH,SRP9,EPHX1,TMEM63A,LEFTY1,PYCR2,PYCR2,MIR6741,LEFTY2,SDE2,H3F3A,H3F3AP4,ACBD3,ACBD3,ACBD3-AS1,MIXL1,LIN9 | 0.47255 | 289.574 | 112 | 64.0608 |
| 3 | 1 | 55542858 | 63024923 | USP24,MIR4422HG,MIR4422,PLPP3,LOC101929935,PRKAA2,PRKAA2,FYB2,C8A,C8B,DAB1,OMA1,TACSTD2,MYSM1,JUN,LINC01135,FGGY,FGGY,MIR4711,HOOK1,CYP2J2,C1orf87,NFIA,TM2D1,PATJ,PATJ,MIR3116-1,MIR3116-2,L1TD1,KANK4,USP1,DOCK7 | 0.466588 | 320.483 | 501 | 296.793 |

| | | | | | | | | |
|---|---|---|---|---|---|---|---|---|
| 3 | 2 | 2.27E+08 | 238455337 | MIR5702,IRS1,RHBDD1,COL4A4,COL4A3,COL4A3,LOC654841,MFF,TM4SF20,MIR5703,AGFG1,AGFG1,C2orf83,SLC19A3,CCL20,DAW1,SPHKAP,PID1,DNER,TRIP12,TRIP12,FBXO36,FBXO36,SLC16A14,SP110,SP140,SP140L,SP100,CAB39,ITM2C,GPR55,SPATA3,C2orf72,PSMD1,PSMD1,HTR2B,ARMC9,ARMC9,MIR4777,B3GNT7,NCL,NCL,SNORA75,NCL,SNORD20,NCL,SNORD82,LINC00471,NMUR1,TEX44,PTMA,PDE6D,COPS7B,MIR1471,NPPC,DIS3L2,ALPP,ALPPL2,ALPI,ECEL1,PRSS56,CHRND,CHRNG,TIGD1,TIGD1,MIR5001,EIF4E2,EIF4E2,EFHD1,GIGYF2,GIGYF2,KCNJ13,SNORC,NGEF,NGEF,LOC101928881,LOC101928881,NEU2,INPP5D,ATG16L1,ATG16L1,SCARNA5,ATG16L1,SCARNA6,SAG,DGKD,USP40,UGT1A8,UGT1A8,UGT1A10,UGT1A8,UGT1A10,UGT1A9,UGT1A8,UGT1A10,UGT1A9,UGT1A7,UGT1A8,UGT1A10,UGT1A9,UGT1A7,UGT1A6,UGT1A8,UGT1A10,UGT1A9,UGT1A7,UGT1A6,UGT1A5,UGT1A8,UGT1A10,UGT1A9,UGT1A7,UGT1A6,UGT1A5,UGT1A4,UGT1A8,UGT1A10,UGT1A9,UGT1A7,UGT1A6,UGT1A5,UGT1A4,UGT1A3,UGT1A8,UGT1A10,UGT1A9,UGT1A7,UGT1A6,UGT1A5,UGT1A4,UGT1A3,DNAJB3,UGT1A8,UGT1A10,UGT1A9,UGT1A7,UGT1A6,UGT1A5,UGT1A4,UGT1A3,LOC100286922,UGT1A8,UGT1A10,UGT1A9,UGT1A7,UGT1A6,UGT1A5,UGT1A4,UGT1A3,UGT1A1,MROH2A,HJURP,MSL3P1,TRPM8,SPP2,ARL4C,SH3BP4,AGAP1,GBX2,ASB18,IQCA1,ACKR3,COPS8,COL6A3,MLPH,MLPH,MIR6811 | 0.462894 | 316.224 | 1454 | 866.456 |
| 3 | 1 | 1.6E+08 | 160970123 | IGSF8,ATP1A2,ATP1A4,CASQ1,LOC729867,PEA15,PEA15,DCAF8,PEX19,COPA,COPA,NCSTN,NCSTN,NHLH1,VANGL2,SLAMF6,CD84,SLAMF1,CD48,SLAMF7,LY9,CD244,ITLN1,LOC101928372,ITLN2,ITLN2,F11R | 0.457698 | 336.665 | 267 | 165.142 |
| 3 | 1 | 1.57E+08 | 159863074 | LRRC71,ARHGEF11,MIR765,ARHGEF11,ETV3L,ETV3,FCRL5,FCRL4,FCRL3,FCRL2,FCRL1,CD5L,KIRREL1,CD1D,CD1A,CD1C,CD1B,CD1E,OR10T2,OR10K2,OR10K1,OR10R2,OR6Y1,OR6P1,OR10X1,OR10Z1,SPTA1,OR6K2,OR6K3,OR6K6,OR6N1,OR6N2,MNDA,PYHIN1,IFI16,AIM2,CADM3,CADM3,CADM3-AS1,ACKR1,FCER1A,OR10J3,OR10J4,OR10J1,OR10J5,APCS,CRP,DUSP23,FCRL6,SLAMF8,SLAMF8,SNHG28,SNHG28,SNHG28,VSIG8,VSIG8,CFAP45 | 0.448577 | 331.171 | 510 | 308.649 |
| 3 | 2 | 1.79E+08 | 179500083 | MIR548N,TTN-AS1,TTN,MIR548N,TTN | 0.441887 | 362.887 | 285 | 204.971 |
| 3 | 1 | 1.16E+08 | 116948388 | SYCP1,TSHB,TSPAN2,NGF,VANGL1,CASQ2,NHLH2,LOC101928977,SLC22A15,SLC22A15,MAB21L3,ATP1A1,ATP1A1,ATP1A1-AS1,ATP1A1-AS1 | 0.43769 | 320.194 | 102 | 62.2001 |

| | | | | | | | | |
|---|---|---|---|---|---|---|---|---|
| 3 | 1 | 2.28E+08 | 234510217 | CDC42BPA,ZNF678,SNAP47,JMJD4,SNAP47,PRSS38,WNT9A,WNT9A,MIR5008,WNT3A,ARF1,MIR3620,ARF1,C1orf35,MRPL55,GUK1,GJC2,IBA57-AS1,IBA57,OBSCN-AS1,OBSCN-AS1,OBSCN,OBSCN,TRIM11,TRIM11,MIR6742,TRIM17,HIST3H3,HIST3H2A,HIST3H2BB,MIR4666A,RNF187,BTNL10,RHOU,DUSP5P1,RHOU,RAB4A,RAB4A,SPHAR,CCSAP,ACTA1,NUP133,ABCB10,TAF5L,URB2,GALNT2,PGBD5,COG2,AGT,CAPN9,C1orf198,TTC13,ARV1,FAM89A,MIR1182,FAM89A,TRIM67,C1orf131,GNPAT,EXOC8,SPRTN,EGLN1,TSNAX,TSNAX-DISC1,TSNAX-DISC1,TSNAX-DISC1,DISC1,TSNAX-DISC1,DISC1,DISC2,SIPA1L2,MAP10,NTPCR,PCNX2,MAP3K21,KCNK1,KCNK1,MIR4427,SLC35F3,SLC35F3,MIR4671,LOC101927765,COA6,COA6 | 0.436696 | 322.001 | 842 | 514.908 |
| 3 | 1 | 1.43E+08 | 145474870 | NBPF20,NBPF19,NBPF8,NBPF9,NBPF20,NBPF19,NBPF9,LOC100996724,PDE4DIP,NBPF20,NBPF19,NBPF9,PDE4DIP,NBPF20,NBPF19,NBPF9,SEC22B,NBPF20,NBPF19,NBPF9,NBPF20,NBPF19,NBPF9,NOTCH2NL,NBPF20,NBPF19,NBPF10,HFE2,NBPF20,NBPF19,NBPF10,TXNIP,NBPF20,NBPF19,NBPF10,POLR3GL,NBPF20,NBPF19,NBPF10,ANKRD34A | 0.432411 | 506.467 | 138 | 76.2426 |
| 3 | 19 | 12768995 | 13316871 | MAN2B1,WDR83,WDR83OS,WDR83,WDR83,DHPS,DHPS,GNG14,FBXW9,TNPO2,TNPO2,SNORD135,TNPO2,SNORD41,TRIR,ASNA1,BEST2,HOOK2,JUNB,PRDX2,RNASEH2A,RTBDN,MAST1,MAST1,MIR6794,DNASE2,KLF1,GCDH,GCDH,SYCE2,SYCE2,FARSA,FARSA,FARSA-AS1,CALR,CALR,MIR6515,RAD23A,GADD45GIP1,DAND5,NFIX,LYL1,TRMT1,NACC1,STX10,IER2 | 0.43004 | 313.769 | 276 | 177.763 |
| 3 | 1 | 64038710 | 70554884 | PGM1,ROR1,UBE2U,CACHD1,CACHD1,MIR4794,RAVER2,JAK1,JAK1,MIR3671,JAK1,MIR101-1,AK4,DNAJC6,LEPROT,LEPR,LEPR,PDE4B,SGIP1,SGIP1,MIR3117,TCTEX1D1,INSL5,WDR78,MIER1,SLC35D1,C1orf141,IL23R,IL12RB2,SERBP1,GADD45A,GNG12,GNG12-AS1,DIRAS3,GNG12-AS1,WLS,GNG12-AS1,WLS,MIR1262,WLS,RPE65,DEPDC1,DEPDC1,DEPDC1-AS1,LRRC7 | 0.425215 | 349.373 | 518 | 303.262 |
| 3 | 6 | 1.34E+08 | 136681223 | TCF21,TBPL1,SLC2A12,SGK1,ALDH8A1,HBS1L,HBS1L,MIR3662,MYB,MIR548A2,AHI1,LINC00271,PDE7B,MTFR2,BCLAF1,MAP7 | 0.405655 | 352.237 | 220 | 129.917 |
| 3 | 3 | 1.49E+08 | 154006772 | HLTF,HLTF,HLTF-AS1,HPS3,HPS3,CP,CP,TM4SF18,TM4SF1,TM4SF4,WWTR1,COMMD2,ANKUB1,RNF13,PFN2,TSC22D2,SERP1,EIF2A,SELENOT,ERICH6,ERICH6,ERICH6-AS1,SIAH2,MINDY4B,CLRN1,CLRN1,CLRN1-AS1,MED12L,MED12L,GPR171,MED12L,P2RY14,MED12L,GPR87,MED12L,P2RY13,MED12L,P2RY12,IGSF10,MIR5186,MIR548H2,AADACL2,MIR548H2,AADACL2,AADACL2-AS1,MIR548H2,AADACL2-AS1,AADAC,AADACL2-AS1,AADAC,AADACL2-AS1,SUCNR1,MBNL1-AS1,MBNL1,MBNL1,MBNL1,TMEM14EP,P2RY1,RAP2B,LINC02006,C3orf79,ARHGEF26,DHX36 | 0.402861 | 341.964 | 440 | 265.678 |

| | | | | | | | | |
|---|---|---|---|---|---|---|---|---|
| 3 | 3 | 48448362 | 48788858 | PLXNB1,CCDC51,TMA7,ATRIP,ATRIP,TREX1,TREX1,SHISA5,PFKFB4,PFKFB4,MIR6823,UCN2,COL7A1,COL7A1,MIR711,UQCRC1,TMEM89,SLC26A6,SLC26A6,MIR6824,CELSR3,CELSR3,MIR4793,CELSR3-AS1,NCKIPSD,IP6K2,PRKAR2A | 0.398022 | 300.51 | 298 | 198.512 |
| 3 | 11 | 93148134 | 94152776 | DEUP1,SMCO4,CEP295,CEP295,SCARNA9,CEP295,TAF1D,TAF1D,SNORA25,TAF1D,SNORA32,TAF1D,SNORD6,TAF1D,SNORA1,TAF1D,SNORA8,TAF1D,SNORD5,TAF1D,SNORA18,TAF1D,TAF1D,SNORA40,C11orf54,MED17,VSTM5,HEPHL1,PANX1,IZUMO1R,GPR83 | 0.3966 | 314.577 | 137 | 85.7187 |
| 3 | 2 | 1.8E+08 | 179612963 | TTN | 0.394622 | 356.565 | 87 | 63.8833 |
| 3 | 16 | 28332751 | 31196476 | EIF3CL,EIF3C,EIF3C,CLN3,APOBR,IL27,NUPR1,SGF29,SULT1A2,SULT1A1,EIF3C,EIF3CL,ATXN2L,TUFM,TUFM,MIR4721,SH2B1,ATP2A1,ATP2A1,ATP2A1-AS1,ATP2A1,RABEP2,RABEP2,CD19,NFATC2IP,NFATC2IP,MIR4517,SPNS1,LAT,RRN3P2,SNX29P2,BOLA2-SMG1P6,LOC606724,BOLA2-SMG1P6,BOLA2,BOLA2B,BOLA2-SMG1P6,BOLA2B,BOLA2,SLX1B,SLX1A,SLX1B,SLX1A,SLX1A-SULT1A3,SLX1B-SULT1A4,SLX1A-SULT1A3,SLX1B-SULT1A4,SLX1A-SULT1A3,SLX1B-SULT1A4,SULT1A3,SULT1A4,LOC388242,LOC613038,SMG1P2,SMG1P2,MIR3680-2,MIR3680-1,SPN,QPRT,C16orf54,ZG16,KIF22,MAZ,PRRT2,PAGR1,MVP,CDIPT,CDIPT-AS1,SEZ6L2,ASPHD1,KCTD13,TMEM219,TAOK2,HIRIP3,INO80E,DOC2A,C16orf92,C16orf92,FAM57B,FAM57B,ALDOA,PPP4C,TBX6,YPEL3,LOC101928595,GDPD3,GDPD3,MAPK3,CORO1A,CORO1A,LOC606724,BOLA2,BOLA2B,BOLA2B,BOLA2,SLX1B,SLX1A,CD2BP2,TBC1D10B,MYLPF,SEPT1,SEPT1,ZNF48,ZNF48,ZNF771,DCTPP1,SEPHS2,ITGAL,ITGAL,MIR4518,ZNF768,ZNF747,ZNF764,ZNF688,ZNF785,ZNF689,PRR14,FBRS,SRCAP,SRCAP,SNORA30,PHKG2,PHKG2,CCDC189,CCDC189,RNF40,RNF40,ZNF629,BCL7C,BCL7C,MIR4519,BCL7C,MIR762HG,BCL7C,MIR762HG,MIR762,CTF1,FBXL19-AS1,FBXL19,FBXL19,ORAI3,SETD1A,HSD3B7,STX1B,STX4,ZNF668,ZNF646,PRSS53,VKORC1,BCKDK,KAT8,PRSS8,PRSS36,FUS | 0.393241 | 305.155 | 1047 | 651.526 |
| 3 | 3 | 1.34E+08 | 141230423 | EPHB1,PPP2R3A,MSL2,PCCB,STAG1,SLC35G2,NCK1-AS1,NCK1,IL20RB,SOX14,CLDN18,DZIP1L,A4GNT,DBR1,ARMC8,ARMC8,NME9,NME9,MRAS,ESYT3,CEP70,FAIM,PIK3CB,FOXL2,FOXL2NB,PRR23A,PRR23B,PRR23C,MRPS22,COPB2,LOC100507291,RBP2,LOC100507291,RBP1,LOC100507291,NMNAT3,NMNAT3,CLSTN2,TRIM42,SLC25A36,SPSB4,PXYLP1,ZBTB38,RASA2 | 0.390392 | 298.392 | 532 | 315.769 |

| | | | | | | | | |
|---|---|---|---|---|---|---|---|---|
| 3 | 1 | 1.12E+08 | 115399922 | RAP1A,FAM212B,FAM212B,DDX20,DDX20,KCND3,LINC01750,CTTNBP2NL,MIR4256,WNT2B,ST7L,CAPZA1,MOV10,RHOC,PPM1J,FAM19A3,SLC16A1,LRIG2,MAGI3,PHTF1,RSBN1,AP4B1-AS1,PTPN22,AP4B1-AS1,BCL2L15,AP4B1-AS1,AP4B1,AP4B1,AP4B1,DCLRE1B,DCLRE1B,HIPK1-AS1,HIPK1,HIPK1,OLFML3,SYT6,TRIM33,BCAS2,DENND2C,AMPD1,NRAS,CSDE1,SIKE1,SYCP1 | 0.386904 | 323.499 | 446 | 277.144 |
| 3 | 6 | 31797570 | 31935174 | HSPA1B,C6orf48,C6orf48,SNORD48,C6orf48,SNORD52,NEU1,SLC44A4,EHMT2,EHMT2,C2,C2,ZBTB12,C2,C2,C2-AS1,CFB,NELFE,NELFE,MIR1236,SKIV2L | 0.384667 | 288.381 | 121 | 80.3073 |
| 3 | 19 | 51306251 | 51607904 | LINC01869,KLK1,KLK15,KLK15,LOC105372441,KLK3,KLK2,KLKP1,KLK4,KLK5,KLK6,KLK7,KLK8,KLK9,KLK10,KLK11,KLK12,KLK13,KLK14,CTU1 | 0.383416 | 273.37 | 109 | 65.4665 |
| 3 | 1 | 94012376 | 94643709 | FNBP1L,BCAR3,BCAR3,LOC100129046,BCAR3,MIR760,DNTTIP2,GCLM,ABCA4,ARHGAP29 | 0.380821 | 319.07 | 116 | 72.0399 |
| 3 | 7 | 1.49E+08 | 151038352 | EZH2,PDIA4,ZNF786,ZNF425,ZNF398,ZNF282,ZNF212,ZNF783,LOC155060,ZNF777,ZNF746,ZNF767P,KRBA1,ZNF467,SSPO,ZNF862,ATP6V0E2,ACTR3C,ACTR3C,LRRC61,LRRC61,ZBED6CL,LRRC61,RARRES2,REPIN1,ZNF775,GIMAP8,GIMAP7,GIMAP4,GIMAP6,GIMAP2,GIMAP1,GIMAP1-GIMAP5,GIMAP1-GIMAP5,GIMAP5,TMEM176B,TMEM176A,AOC1,KCNH2,NOS3,NOS3,ATG9B,ATG9B,ABCB8,ASIC3,CDK5,SLC4A2,FASTK,TMUB1,AGAP3,GBX1,ASB10,ABCF2,CHPF2,CHPF2,MIR671,SMARCD3 | 0.372744 | 288.867 | 665 | 423.764 |
| 3 | 6 | 30632551 | 31783593 | DHX16,PPP1R18,NRM,MDC1,MDC1,MDC1-AS1,TUBB,FLOT1,IER3,LINC00243,DDR1,DDR1,MIR4640,GTF2H4,VARS2,SFTA2,DPCR1,DPCR1,HCG21,MUC21,MUC22,C6orf15,PSORS1C1,PSORS1C1,CDSN,PSORS1C1,PSORS1C2,CCHCR1,TCF19,POU5F1,HCG27,HLA-C,HLA-B,HLA-B,MIR6891,MICA,HCP5,MICB,MCCD1,DDX39B,ATP6V1G2-DDX39B,DDX39B,ATP6V1G2-DDX39B,SNORD117,DDX39B,ATP6V1G2-DDX39B,SNORD84,DDX39B,ATP6V1G2-DDX39B,DDX39B-AS1,ATP6V1G2-DDX39B,ATP6V1G2,NFKBIL1,LOC100287329,LTA,LTA,TNF,LTB,LST1,NCR3,AIF1,PRRC2A,PRRC2A,SNORA38,BAG6,APOM,C6orf47,GPANK1,CSNK2B,LY6G5B,LY6G5C,ABHD16A,ABHD16A,MIR4646,LY6G6F,LY6G6F-LY6G6D,LY6G6F-LY6G6D,LY6G6E,LY6G6F-LY6G6D,LY6G6D,LY6G6C,MPIG6B,DDAH2,CLIC1,MSH5,MSH5-SAPCD1,MSH5-SAPCD1,SAPCD1,MSH5-SAPCD1,SAPCD1,SAPCD1-AS1,VWA7,VARS,LSM2,HSPA1L,HSPA1A | 0.368998 | 301.806 | 573 | 360.711 |
| 3 | 3 | 1.46E+08 | 148757033 | PLSCR5,ZIC4,ZIC1,AGTR1,CPB1,CPA3,GYG1,HLTF | 0.366998 | 338.18 | 81 | 49.4457 |
| 3 | 6 | 12750511 | 15368402 | PHACTR1,PHACTR1,TBC1D7-LOC100130357,PHACTR1,TBC1D7-LOC100130357,LOC100130357,TBC1D7-LOC100130357,LOC100130357,TBC1D7-LOC100130357,TBC1D7,GFOD1,SIRT5,NOL7,NOL7,RANBP9,RANBP9,MCUR1,RNF182,CD83,JARID2 | 0.3553 | 265.189 | 119 | 72.3704 |

| | | | | | | | | |
|---|---|---|---|---|---|---|---|---|
| 3 | 6 | 25813107 | 27878502 | SLC17A1,SLC17A3,SLC17A2,TRIM38,HIST1H1A,HIST1H3A,HIST1H4A,HIST1H4B,HIST1H3B,HIST1H2AB,HIST1H2BB,HIST1H3C,HIST1H1C,LOC108783645,HFE,HFE,HIST1H4C,HIST1H1T,HIST1H2BC,HIST1H2AC,HIST1H1E,HIST1H2BD,HIST1H2BE,HIST1H4D,HIST1H3D,HIST1H3D,HIST1H2AD,HIST1H2BF,HIST1H4E,HIST1H2BG,HIST1H2AE,HIST1H3E,HIST1H1D,HIST1H4F,HIST1H4G,HIST1H3F,HIST1H2BH,HIST1H3G,HIST1H2BI,HIST1H4H,BTN3A2,BTN2A2,BTN3A1,BTN2A3P,BTN3A3,BTN2A1,BTN2A1,LOC285819,BTN1A1,HMGN4,ABT1,ZNF322,HIST1H2BJ,HIST1H2AG,HIST1H2BK,HIST1H4I,HIST1H2BK,HIST1H2AH,HIST1H2AH,MIR3143,PRSS16,POM121L2,VN1R10P,ZNF204P,ZNF391,ZNF184,HIST1H2BL,HIST1H2AI,HIST1H3H,HIST1H2AJ,HIST1H2BM,HIST1H4J,HIST1H4K,HIST1H2AK,HIST1H2BN,HIST1H2AL,HIST1H1B,HIST1H3I,HIST1H4L,HIST1H3J,HIST1H2AM,HIST1H2BO | 0.353297 | 327.023 | 343 | 198.543 |
| 3 | 6 | 32337602 | 34725692 | LOC101929163,C6orf10,LOC101929163,BTNL2,HLA-DRA,HLA-DRB5,HLA-DRB1,HLA-DQA1,HLA-DQB1,HLA-DQB1-AS1,HLA-DQB1,HLA-DQA2,HLA-DQB2,HLA-DOB,TAP2,PSMB8,PSMB8,PSMB8-AS1,PSMB8-AS1,TAP1,TAP1,PSMB9,HLA-DMB,HLA-DMA,BRD2,HLA-DOA,HLA-DPA1,HLA-DPA1,HLA-DPB1,HLA-DPB1,HLA-DPB2,COL11A2,RXRB,RXRB,SLC39A7,SLC39A7,HSD17B8,MIR219A1,RING1,HCG25,VPS52,VPS52,RPS18,B3GALT4,WDR46,WDR46,MIR6873,PFDN6,PFDN6,MIR6834,RGL2,TAPBP,ZBTB22,DAXX,KIFC1,PHF1,CUTA,SYNGAP1,SYNGAP1,MIR5004,ZBTB9,BAK1,GGNBP1,GGNBP1,LINC00336,LINC00336,ITPR3,UQCC2,UQCC2,MIR3934,IP6K3,LEMD2,MLN,MIR1275,GRM4,HMGA1,SMIM29,NUDT3,RPS10-NUDT3,RPS10-NUDT3,RPS10,PACSIN1,SPDEF,C6orf106,C6orf106,LOC101929243,SNRPC | 0.34942 | 278.524 | 628 | 383.11 |
| 3 | 11 | 1.19E+08 | 120096521 | NLRX1,PDZD3,CCDC153,CBL,MCAM,MCAM,MIR6756,RNF26,C1QTNF5,MFRP,MFRP,C1QTNF5,USP2,USP2-AS1,USP2-AS1,THY1,NECTIN1,TRIM29,OAF | 0.348301 | 272.981 | 168 | 108.997 |
| 3 | 6 | 32012152 | 32261672 | TNXB,ATF6B,FKBPL,PRRT1,LOC100507547,PPT2,PPT2,PPT2-EGFL8,PPT2-EGFL8,EGFL8,AGPAT1,AGPAT1,MIR6721,RNF5,RNF5P1,RNF5,RNF5P1,MIR6833,AGER,PBX2,GPSM3,GPSM3,NOTCH4,NOTCH4,LOC101929163,LOC101929163,C6orf10 | 0.335154 | 259.091 | 190 | 113.637 |

| | | | | | | | | |
|---|---|---|---|---|---|---|---|---|
| 3 | 21 | 30444220 | 38459151 | CCT8,MAP3K7CL,BACH1,GRIK1,GRIK1,GRIK1-AS2,CLDN17,CLDN8,KRTAP24-1,KRTAP25-1,KRTAP26-1,KRTAP27-1,KRTAP23-1,KRTAP13-2,MIR4327,KRTAP13-1,KRTAP13-3,KRTAP13-4,KRTAP15-1,KRTAP19-1,KRTAP19-2,KRTAP19-3,KRTAP19-4,KRTAP19-5,KRTAP19-6,KRTAP19-7,KRTAP22-2,KRTAP6-3,KRTAP6-2,KRTAP22-1,KRTAP6-1,KRTAP20-1,KRTAP20-4,KRTAP20-2,KRTAP20-3,KRTAP21-3,KRTAP21-2,KRTAP21-1,KRTAP8-1,KRTAP7-1,KRTAP11-1,KRTAP19-8,TIAM1,LOC150051,SOD1,SCAF4,HUNK,MIS18A,MRAP,MRAP,URB1,URB1,URB1,SNORA80A,URB1-AS1,EVA1C,C21orf59-TCP10L,TCP10L,C21orf59-TCP10L,C21orf59-TCP10L,C21orf59,SYNJ1,SYNJ1,PAXBP1-AS1,PAXBP1-AS1,PAXBP1,PAXBP1,C21orf62-AS1,C21orf62-AS1,C21orf62,C21orf62,OLIG2,OLIG1,LINC01548,IFNAR2,IL10RB-AS1,IL10RB,IFNAR1,IFNGR2,IFNGR2,TMEM50B,TMEM50B,DNAJC28,GART,SON,SON,MIR6501,DONSON,CRYZL1,ITSN1,ATP5PO,SLC5A3,MRPS6,MRPS6,KCNE2,SMIM11A,SMIM11B,C21orf140,KCNE1,KCNE1B,RCAN1,CLIC6,RUNX1,RUNX1,RUNX1-IT1,MIR802,SETD4,LOC100133286,CBR1,CBR3-AS1,CBR3,DOPEY2,MORC3,CHAF1B,CLDN14,SIM2,HLCS,RIPPLY3,PIGP | 0.332439 | 302.367 | 905 | 542.419 |
| 3 | 5 | 20957651 | 34715622 | GUSBP1,CDH12,PRDM9,C5orf17,CDH10,LINC02228,CDH9,CDH6,DROSHA,C5orf22,PDZD2,PDZD2,MIR4279,GOLPH3,MTMR12,ZFR,ZFR,MIR579,SUB1,NPR3,TARS,ADAMTS12,RXFP3,SLC45A2,AMACR,C1QTNF3-AMACR,C1QTNF3-AMACR,C1QTNF3,RAI14 | 0.330964 | 256.014 | 464 | 281.468 |
| 3 | 11 | 61323474 | 62781576 | SYT7,RPLP0P2,DAGLA,MYRF,MYRF,DKFZP434K028,TMEM258,TMEM258,MIR611,FEN1,FADS1,FADS1,MIR1908,FADS1,FADS2,FADS2,FADS3,FADS3,MIR6746,RAB3IL1,BEST1,FTH1,INCENP,SCGB1D1,SCGB2A1,SCGB1D2,SCGB2A2,SCGB1D4,ASRGL1,SCGB1A1,AHNAK,EEF1G,EEF1G,MIR3654,EEF1G,MIR6747,TUT1,MTA2,EML3,ROM1,B3GAT3,GANAB,INTS5,C11orf98,LBHD1,LBHD1,CSKMT,SNORA57,LBHD1,CSKMT,LBHD1,LBHD1,UQCC3,UQCC3,UBXN1,LRRN4CL,BSCL2,HNRNPUL2-BSCL2,BSCL2,HNRNPUL2-BSCL2,GNG3,HNRNPUL2-BSCL2,HNRNPUL2,TTC9C,ZBTB3,POLR2G,TAF6L,TAF6L,TMEM179B,TMEM179B,TMEM179B,MIR6748,TMEM223,NXF1,MIR6514,NXF1,STX5,LOC105369332,WDR74,WDR74,SNHG1,SNORD22,SNHG1,SNORD31,SNHG1,SNORD30,SNHG1,SNORD29,SNHG1,SNORD28,SNHG1,SNORD27,SNORD26,SNHG1,SNORD25,SLC3A2,CHRM1,SLC22A6,SLC22A8 | 0.324562 | 298.02 | 566 | 356.973 |

| | | | | | | | | |
|---|---|---|---|---|---|---|---|---|
| 3 | 1 | 1.76E+08 | 185069140 | RFWD2,PAPPA2,ASTN1,ASTN1,MIR488,BRINP2,SEC16B,CRYZL2P-SEC16B,RASAL2-AS1,RASAL2,TEX35,C1orf220,MIR4424,RALGPS2,RALGPS2,ANGPTL1,FAM20B,TOR3A,ABL2,SOAT1,AXDND1,AXDND1,NPHS2,NPHS2,TDRD5,FAM163A,TOR1AIP2,TOR1AIP1,CEP350,QSOX1,FLJ23867,LHX4,LHX4,LHX4-AS1,ACBD6,XPR1,KIAA1614,KIAA1614-AS1,STX6,MR1,IER5,CACNA1E,ZNF648,GLUL,TEDDM1,RGSL1,RNASEL,RGS16,RGS8,NPL,DHX9,SHCBP1L,LAMC1,LAMC1,LAMC1-AS1,LAMC2,NMNAT2,SMG7,NCF2,ARPC5,RGL1,APOBEC4,RGL1,COLGALT2,TSEN15,C1orf21,EDEM3,FAM129A,RNF2 | 0.320214 | 303.537 | 1017 | 613.49 |
| 3 | 11 | 63533321 | 65119169 | [illegible]UB1,MACROD1,MACROD1,FLRT1,STIP1,FERMT3,FERMT3,TRPT1,TRPT1,NUDT22,DNAJC4,VEGFB,FKBP2,PPP1R14B,PLCB3,BAD,BAD,GPR137,GPR137,KCNK4,KCNK4-TEX40,KCNK4-TEX40,CATSPERZ,ESRRA,TRMT112,PRDX5,CCDC88B,RPS6KA4,RPS6KA4,MIR1237,LOC100996455,SLC22A11,SLC22A12,NRXN2,RASGRP2,PYGM,SF1,MAP4K2,MEN1,CDC42BPG,EHD1,MIR194-2HG,MIR194-2HG,MIR192,MIR194-2,ATG2A,ATG2A,MIR6750,ATG2A,MIR6749,PPP2R5B,GPHA2,MAJIN,BATF2,ARL2,ARL2-SNX15,ARL2,ARL2-SNX15,MIR6879,ARL2-SNX15,SNX15,SAC3D1,NAALADL1,CDCA5,ZFPL1,ZFPL1,TMEM262,TMEM262,VPS51,TM7SF2,ZNHIT2,FAU,MRPL49,SYVN1,SYVN1,MIR6751,SPDYC,LOC728975,LOC728975,CAPN1,CAPN1,SLC22A20P,POLA2,CDC42EP2,DPF2 | 0.318898 | 270.72 | 723 | 462.188 |
| 3 | 8 | 94829255 | 95735247 | MIR378D2,PDP1,CDH17,GEM,RAD54B,RAD54B,FSBP,VIRMA,LOC100288748,ESRP1,DPY19L4 | 0.316417 | 309.76 | 113 | 71.1168 |
| 3 | 3 | 1.84E+08 | 184542551 | HTR3E,EIF2B5,DVL3,AP2M1,ABCF3,VWA5B2,VWA5B2,MIR1224,ALG3,EEF1AKMT4,EEF1AKMT4-ECE2,EEF1AKMT4-ECE2,CAMK2N2,EEF1AKMT4-ECE2,ECE2,PSMD2,EIF4G1,EIF4G1,SNORD66,FAM131A,CLCN2,POLR2H,THPO,CHRD,LINC02054,EPHB3,MAGEF1,VPS8 | 0.302424 | 272.261 | 284 | 183.8 |
| 3 | 8 | 1.04E+08 | 109795868 | LOC105375690,RIMS2,RIMS2,DCSTAMP,DPYS,LRP12,ZFPM2,ZFPM2,ZFPM2-AS1,OXR1,ABRA,ANGPT1,RSPO2,EIF3E,EMC2 | 0.299893 | 278.66 | 251 | 142.827 |
| 3 | 22 | 20609931 | 21188945 | ZNF74,SCARF2,KLHL22,MED15,POM121L4P,TMEM191A,PI4KA,PI4KA,SERPIND1 | 0.297806 | 269.88 | 151 | 87.7765 |
| 3 | 4 | 5981939 | 10509695 | JAKMIP1,WFS1,PPP2R2C,MAN2B2,MRFAP1,LOC93622,S100P,MRFAP1L1,BLOC1S4,KIAA0232,TBC1D14,LOC100129931,CCDC96,LOC100129931,TADA2B,TADA2B,GRPEL1,SORCS2,SORCS2,MIR4798,SORCS2,PSAPL1,SORCS2,MIR4274,AFAP1-AS1,AFAP1,AFAP1,AFAP1,LOC389199,ABLIM2,ABLIM2,MIR95,SH3TC1,HTRA3,ACOX3,TRMT44,GPR78,CPZ,HMX1,USP17L6P,DRD5,SLC2A9,WDR1,WDR1,MIR3138,ZNF518B,CLNK | 0.296847 | 253.574 | 459 | 279.787 |

| | | | | | | | | |
|---|---|---|---|---|---|---|---|---|
| 3 | 7 | 1.42E+08 | 143884193 | MGAM,MGAM2,PRSS58,PRSS1,PRSS3P2,EPHB6,TRPV6,TRPV5,LLCFC1,KEL,OR9A2,OR6V1,OR6W1P,PIP,TAS2R39,TAS2R40,LOC105375545,LOC105375545,GSTK1,LOC105375545,TMEM139,TMEM139,CASP2,CLCN1,FAM131B,ZYX,ZYX,MIR6892,EPHA1,EPHA1,EPHA1-AS1,EPHA1-AS1,TAS2R60,EPHA1-AS1,TAS2R41,TCAF2,LOC154761,TCAF1,OR2F2,OR2F1,OR6B1,OR2A5,OR2A25,OR2A12,OR2A2,OR2A14,ARHGEF35 | 0.295434 | 318.879 | 542 | 316.645 |
| 3 | 9 | 34497015 | 35956694 | DNAI1,ENHO,CNTFR,CNTFR,CNTFR-AS1,RPP25L,DCTN3,ARID3C,SIGMAR1,GALT,IL11RA,IL11RA,CCL27,CCL27,LOC730098,CCL19,CCL21,FAM205A,FAM205BP,PHF24,DNAJB5,C9orf131,VCP,FANCG,PIGO,STOML2,FAM214B,UNC13B,RUSC2,RUSC2,FAM166B,FAM166B,TESK1,TESK1,MIR4667,CD72,SIT1,CCDC107,CCDC107,ARHGEF39,ARHGEF39,CA9,TPM2,TLN1,TLN1,MIR6852,CREB3,MIR6853,CREB3,GBA2,RGP1,RGP1,MSMP,MSMP,NPR2,NPR2,SPAG8,SPAG8,HINT2,FAM221B,TMEM8B,OR13J1,HRCT1,SPAAR | 0.290247 | 271.942 | 516 | 335.918 |
| 3 | 1 | 1.74E+08 | 175956156 | SLC9C2,ANKRD45,ANKRD45,TEX50,KLHL20,CENPL,DARS2,GAS5,SNORD81,GAS5,SNORD47,GAS5,SNORD80,GAS5,SNORD79,GAS5,SNORD78,GAS5,SNORD44,GAS5,SNORA103,SNORD77,GAS5,SNORD76,GAS5,SNORD75,GAS5,SNORD74,ZBTB37,SERPINC1,RC3H1,RABGAP1L,RABGAP1L,GPR52,CACYBP,MRPS14,TNN,KIAA0040,TNR,RFWD2,RFWD2,SCARNA3 | 0.279107 | 315.678 | 237 | 141.721 |
| 3 | 9 | 33290480 | 33534483 | NFX1,AQP7,AQP3,NOL6,ANKRD18B | 0.27718 | 259.508 | 76 | 46.9645 |
| 3 | 7 | 74167958 | 76871162 | NCF1,GTF2IRD2,RCC1L,GTF2IRD2,GTF2IRD2B,NCF1C,GTF2IP4,GTF2IP1,GTF2IP1,PMS2P5,GTF2IP1,PMS2P5,CASTOR2,TRIM73,NSUN5P1,NSUN5P1,POM121C,POM121C,PMS2P3,HIP1,CCL26,CCL24,RHBDD2,POR,MIR4651,POR,SNORA14A,POR,TMEM120A,STYXL1,MDH2,SRRM3,HSPB1,YWHAG,SSC4D,SSC4D,ZP3,ZP3,DTX2,UPK3B,LOC100133091,POMZP3,DTX2P1-UPK3BP1-PMS2P11,FAM185BP,CCDC146,CCDC146,FGL2 | 0.274123 | 234.048 | 368 | 198.51 |
| 3 | 11 | 65715492 | 67434513 | TSGA10IP,SART1,EIF1AD,BANF1,CST6,CATSPER1,GAL3ST3,SF3B2,PACS1,KLC2,RAB1B,CNIH2,YIF1A,TMEM151A,CD248,RIN1,BRMS1,B4GAT1,LOC102724064,SLC29A2,SLC29A2,NPAS4,MRPL11,PELI3,PELI3,LOC101928069,DPP3,BBS1,BBS1,ZDHHC24,ZDHHC24,ACTN3,CTSF,CCDC87,CCS,RBM14,RBM14-RBM4,RBM14-RBM4,RBM4,RBM4,RBM4,RBM4B,RBM4B,SPTBN2,C11orf80,C11orf80,RCE1,RCE1,PC,PC,LRFN4,PC,MIR3163,C11orf86,SYT12,SYT12,MIR6860,RHOD,KDM2A,GRK2,ANKRD13D,SSH3,LOC100130987,POLD4,LOC100130987,CLCF1,RAD9A,PPP1CA,TBC1D10C,CARNS1,RPS6KB2,PTPRCAP,CORO1B,GPR152,GPR152,CABP4,CABP4,TMEM134,AIP,AIP,MIR6752,PITPNM1,CDK2AP2,CABP2,GSTP1,C11orf72,NDUFV1,NUDT8,TBX10,ACY3,ALDH3B2 | 0.272239 | 270.44 | 749 | 476.037 |

| | | | | | | | | |
|---|---|---|---|---|---|---|---|---|
| 3 | 8 | 95906097 | 104432777 | CCNE2,NDUFAF6,TP53INP1,NDUFAF6,NDUFAF6,MIR3150BHG,MIR3150B,MIR3150A,PLEKHF2,C8orf37,C8orf37,C8orf37-AS1,GDF6,UQCRB,MTERF3,PTDSS1,LOC102724804,SDC2,CPQ,CPQ,LOC101927066,LOC101927066,TSPYL5,MTDH,LAPTM4B,MATN2,RPL30,RPL30,SNORA72,ERICH5,RIDA,POP1,NIPAL2,KCNS2,STK3,OSR2,VPS13B,VPS13B,MIR599,MIR875,COX6C,RGS22,RGS22,MIR1273A,FBXO43,POLR2K,SPAG1,RNF19A,ANKRD46,SNX31,PABPC1,YWHAZ,ZNF706,NACAP1,GRHL2,NCALD,MIR5680,RRM2B,UBR5,ODF1,KLF10,GASAL1,AZIN1,AZIN1-AS1,ATP6V1C1,LINC01181,BAALC-AS2,BAALC-AS2,BAALC,BAALC,MIR3151,BAALC,FZD6,CTHRC1,SLC25A32,SLC25A32,DCAF13,DCAF13 | 0.270689 | 278.598 | 727 | 423.122 |
| 3 | 19 | 28263182 | 32847734 | UQCRFS1,VSTM2B,POP4,PLEKHF1,C19orf12,CCNE1,URI1,ZNF536,TSHZ3,THEG5,ZNF507 | 0.263245 | 249.313 | 140 | 89.1581 |
| 3 | 19 | 49863031 | 50657962 | TEAD2,DKKL1,DKKL1,LOC101928295,CCDC155,PTH2,GFY,SLC17A7,PIH1D1,ALDH16A1,FLT3LG,RPL13A,RPL13AP5,RPL13A,RPL13AP5,SNORD32A,RPL13A,RPL13AP5,SNORD33,RPL13A,RPL13AP5,SNORD34,RPL13A,RPL13AP5,SNORD35A,RPS11,RPS11,SNORD35B,MIR150,FCGRT,RCN3,NOSIP,PRRG2,PRR12,RRAS,SCAF1,IRF3,IRF3,BCL2L12,BCL2L12,PRMT1,PRMT1,MIR5088,ADM5,CPT1C,TSKS,AP2A1,AP2A1,MIR6799,AP2A1,FUZ,FUZ,MED25,MED25,MIR6800,PTOV1-AS1,PTOV1-AS1,PTOV1,PTOV1,MIR4749,PTOV1,PTOV1,PTOV1-AS2,PNKP,AKT1S1,AKT1S1,TBC1D17,TBC1D17,TBC1D17,MIR4750,IL4I1,IL4I1,NUP62,ATF5,ATF5,MIR4751,SIGLEC11,SIGLEC16,VRK3,ZNF473,LOC400710,IZUMO2 | 0.261227 | 282.085 | 423 | 267.562 |
| 3 | 12 | 570251 | 752910 | B4GALNT3,NINJ2,NINJ2,LOC100049716 | 0.260175 | 281.115 | 33 | 21.0589 |
| 3 | 3 | 1.54E+08 | 157921020 | DHX36,GPR149,MME,PLCH1,PLCH1,PLCH1-AS2,C3orf33,SLC33A1,GMPS,KCNAB1,SSR3,TIPARP,LEKR1,CCNL1,VEPH1,VEPH1,PTX3,PQLC2L,SHOX2,RSRC1 | 0.259489 | 274.012 | 272 | 159.104 |
| 3 | 15 | 73925460 | 75517674 | NPTN,CD276,C15orf59,TBC1D21,LOXL1-AS1,LOXL1,LOXL1,STOML1,PML,LOC283731,ISLR2,ISLR,STRA6,CCDC33,CYP11A1,SEMA7A,SEMA7A,MIR6881,UBL7,ARID3B,CLK3,EDC3,CYP1A1,CYP1A2,CSK,MIR4513,CSK,LMAN1L,CPLX3,ULK3,SCAMP2,MPI,FAM219B,COX5A,RPP25,SCAMP5,PPCDC,C15orf39 | 0.259226 | 249.641 | 368 | 227.673 |
| 3 | 6 | 42657274 | 44361395 | UBR2,PRPH2,TBCC,BICRAL,RPL7L1,C6orf226,PTCRA,CNPY3,CNPY3-GNMT,CNPY3-GNMT,CNPY3-GNMT,GNMT,PEX6,PPP2R5D,MEA1,MEA1,KLHDC3,KLHDC3,RRP36,CUL7,MRPL2,KLC4,PTK7,SRF,CUL9,DNPH1,TTBK1,SLC22A7,CRIP3,ZNF318,ABCC10,DLK2,TJAP1,LRRC73,YIPF3,POLR1C,POLR1C,XPO5,XPO5,POLH,GTPBP2,MAD2L1BP,RSPH9,MRPS18A,VEGFA,LOC101929705,C6orf223,MRPL14,TMEM63B,CAPN11,MYMX,SLC29A1,HSP90AB1,SLC35B2,MIR4647,SLC35B2,NFKBIE,TMEM151B,TCTE1,AARS2,SPATS1,CDC5L | 0.254528 | 273.495 | 672 | 426.246 |

| | | | | | | | | |
|---|---|---|---|---|---|---|---|---|
| 3 | 11 | 79442211 | 88961154 | MIR4300HG,MIR4300,FAM181B,PRCP,DDIAS,RAB30,RAB30,SNORA70E,PCF11,PCF11,LOC100506282,ANKRD42,CCDC90B,DLG2,TMEM126B,TMEM126A,CREBZF,CCDC89,SYTL2,CCDC83,PICALM,EED,EED,MIR6755,HIKESHI,CCDC81,ME3,PRSS23,PRSS23,FZD4,FZD4,TMEM135,RAB38,MIR3166,CTSC,GRM5-AS1,GRM5,GRM5,TYR | 0.24855 | 264.398 | 479 | 286.946 |
| 3 | 17 | 74303474 | 76128251 | QRICH2,PRPSAP1,SPHK1,UBE2O,AANAT,RHBDF2,CYGB,PRCD,PRCD,SNHG16,SNORD1C,SNHG16,SNHG16,SNORD1B,SNHG16,SNORD1A,ST6GALNAC2,ST6GALNAC1,MXRA7,JMJD6,METTL23,SRSF2,SRSF2,MIR636,SRSF2,MFSD11,MFSD11,LINC00868,MGAT5B,SNHG20,SEC14L1,SCARNA16,SNHG20,SEC14L1,SCARNA16,MIR6516,SEC14L1,LOC105371907,SEPT9,SEPT9,SEPT9,MIR4316,LINC01973,TNRC6C,TNRC6C,TNRC6C-AS1,TNRC6C-AS1,TMC6,TMC6,TMC8 | 0.245363 | 244.97 | 314 | 191.944 |
| 3 | 1 | 6614429 | 6880398 | NOL9,TAS1R1,ZBTB48,KLHL21,PHF13,THAP3,THAP3,DNAJC11,DNAJC11,CAMTA1 | 0.243586 | 278.866 | 82 | 48.258 |
| 3 | 7 | 99454439 | 102120766 | CYP3A43,OR2AE1,TRIM4,GJC3,AZGP1,AZGP1P1,ZKSCAN1,ZSCAN21,ZSCAN21,ZNF3,ZNF3,COPS6,MCM7,MCM7,MIR25,MIR93,MCM7,MIR106B,MCM7,AP4M1,AP4M1,TAF6,TAF6,CNPY4,CNPY4,MBLAC1,LAMTOR4,C7orf43,C7orf43,MIR4658,GAL3ST4,GPC2,STAG3,STAG3,GATS,GATS,GATS,PVRIG,SPDYE3,STAG3L5P-PVRIG2P-PILRB,PVRIG2P,STAG3L5P-PVRIG2P-PILRB,MIR6840,STAG3L5P-PVRIG2P-PILRB,PILRB,PILRA,ZCWPW1,MEPCE,PPP1R35,C7orf61,TSC22D4,NYAP1,AGFG2,SAP25,LRCH4,ZASP,LRCH4,FBXO24,FBXO24,PCOLCE-AS1,PCOLCE-AS1,PCOLCE,PCOLCE,MOSPD3,TFR2,ACTL6B,GNB2,GIGYF1,POP7,EPO,ZAN,EPHB4,SLC12A9,TRIP6,TRIP6,MIR6875,SRRT,SRRT,UFSP1,UFSP1,ACHE,MUC3A,MUC12,MUC12,LOC102724094,MUC17,TRIM56,SERPINE1,AP1S1,AP1S1,MIR4653,VGF,NAT16,MOGAT3,PLOD3,ZNHIT1,CLDN15,FIS1,LOC101927746,IFT22,COL26A1,MYL10,CUX1,SH2B2,SH2B2,MIR4285,LOC100289561,LOC100630923,LOC100630923,LOC100630923,PRKRIP1,LOC100630923,PRKRIP1,MIR548O,ORAI2,ALKBH4,LRWD1,LRWD1,MIR5090,LRWD1,MIR4467,POLR2J | 0.236262 | 302.49 | 877 | 541.045 |
| 3 | 11 | 94229984 | 102826377 | ANKRD49,C11orf97,FUT4,FUT4,LOC105369438,LOC105369438,PIWIL4,LOC105369438,AMOTL1,CWC15,KDM4D,KDM4E,SRSF8,ENDOD1,SESN3,SESN3,LOC100129203,FAM76B,CEP57,MTMR2,MAML2,MAML2,MIR1260B,CCDC82,JRKL,CNTN5,LOC100128386,ARHGAP42,ARHGAP42,PGR,PGR,PGR-AS1,TRPC6,TRPC6,MIR3920,ANGPTL5,ANGPTL5,CEP126,CEP126,C11orf70,YAP1,BIRC3,BIRC2,TMEM123,LOC102723838,MMP7,MMP20,MMP27,MMP8,MMP10,WTAPP1,MMP1,WTAPP1,MMP3,MMP3,MMP12,MMP13 | 0.235465 | 285.005 | 550 | 328.599 |
| 3 | 1 | 2.47E+08 | 247265220 | AHCTF1,ZNF695,ZNF670-ZNF695,ZNF670-ZNF695,ZNF670,ZNF669 | 0.233986 | 273.036 | 31 | 17.3032 |

| | | | | | | | | |
|---|---|---|---|---|---|---|---|---|
| 3 | 9 | 1.29E+08 | 132979610 | MVB12B,LMX1B,ZBTB43,ZBTB34,RALGPS1,RALGPS1,ANGPTL2,GARNL3,SLC2A8,ZNF79,RPL12,RPL12,SNORA65,LRSAM1,FAM129B,STXBP1,STXBP1,MIR3911,CFAP157,CFAP157,PTRH1,PTRH1,TTC16,TOR2A,SH2D3C,MIR3960,MIR2861,CDK9,CDK9,FPGS,ENG,ENG,LOC102723566,AK1,AK1,MIR4672,ST6GALNAC6,ST6GALNAC4,PIP5KL1,DPM2,FAM102A,NAIF1,SLC25A25,PTGES2,PTGES2-AS1,LCN2,C9orf16,CIZ1,CIZ1,DNM1,DNM1,DNM1,MIR199B,MIR3154,GOLGA2,GOLGA2,SWI5,SWI5,TRUB2,COQ4,SLC27A4,SLC27A4,MIR1268A,MIR1268A,URM1,MIR1268A,MIR219A2,MIR219B,MIR1268A,CERCAM,MIR1268A,ODF2,MIR1268A,GLE1,MIR1268A,SPTAN1,MIR1268A,WDR34,SET,PKN3,ZDHHC12,LOC100506100,ZER1,ZER1,TBC1D13,ENDOG,ENDOG,SPOUT1,SPOUT1,KYAT1,LRRC8A,PHYHD1,DOLK,DOLK,NUP188,NUP188,SH3GLB2,MIGA2,DOLPP1,CRAT,PTPA,IER5L,C9orf106,NTMT1,C9orf50,NTMT1,NTMT1,ASB6,ASB6,PRRX2,PTGES,TOR1B,TOR1A,C9orf78,USP20,USP20,MIR6855,FNBP1,GPR107,LOC401554,NCS1 | 0.233025 | 269.582 | 1049 | 648.167 |
| 3 | 3 | 50677715 | 52570962 | MAPKAPK3,MIR4787,DOCK3,DOCK3,MANF,RBM15B,DCAF1,RAD54L2,TEX264,GRM2,IQCF6,IQCF3,IQCF2,IQCF5-AS1,IQCF5,IQCF1,RRP9,PARP3,GPR62,PCBP4,ABHD14B,ABHD14A,ABHD14A-ACY1,ABHD14A-ACY1,ACY1,RPL29,DUSP7,LINC00696,POC1A,ALAS1,TLR9,TWF2,PPM1M,WDR82,WDR82,MIRLET7G,GLYCTK,GLYCTK,MIR135A1,DNAH1,BAP1,PHF7,SEMA3G,TNNC1,NISCH,STAB1,STAB1,NT5DC2,NT5DC2,SMIM4 | 0.227489 | 275.826 | 606 | 387.485 |
| 3 | 1 | 1.62E+08 | 173494116 | UHMK1,UAP1,DDR2,HSD17B7,CCDC190,RGS4,RGS5,RGS5,LOC101928404,NUF2,PBX1,LMX1A,RXRG,LOC400794,LRRC52,MGST3,ALDH9A1,TMCO1,UCK2,UCK2,MIR3658,FAM78B,FAM78B,MIR921,FMO9P,POGK,TADA1,ILDR2,MAEL,GPA33,DUSP27,POU2F1,CD247,CREG1,RCSD1,MPZL1,ADCY10,MPC2,MPC2,DCAF6,DCAF6,DCAF6,MIR1255B2,GPR161,TIPRL,SFT2D2,ANKRD36BP1,TBX19,MIR557,XCL2,XCL1,DPT,ATP1B1,ATP1B1,NME7,NME7,BLZF1,BLZF1,CCDC181,CCDC181,SLC19A2,F5,SELP,SELL,SELE,METTL18,C1orf112,SCYL3,KIFAP3,METTL11B,METTL11B,MIR3119-1,MIR3119-2,LOC101928650,GORAB,GORAB,PRRX1,MROH9,FMO3,FMO3,MIR1295A,MIR1295B,FMO6P,FMO2,FMO1,FMO4,PRRC2C,MYOC,VAMP4,METTL13,DNM3,DNM3,DNM3OS,MIR214,MIR3120,DNM3,DNM3OS,MIR214,DNM3,DNM3OS,MIR199A2,C1orf105,C1orf105,PIGC,SUCO,FASLG,TNFSF18,TNFSF4,PRDX6,SLC9C2 | 0.227082 | 287.856 | 1033 | 617.952 |

| | | | | | | | | |
|---|---|---|---|---|---|---|---|---|
| 3 | 14 | 19057400 | 21679808 | DUXAP10,BMS1P22,OR4Q3,OR4M1,OR4N2,OR4K2,OR4K5,OR4K1,OR4K15,OR4Q2,OR4K14,OR4K13,OR4L1,OR4K17,OR4N5,OR11G2,OR11H6,OR11H7,OR11H4,TTC5,CCNB1IP1,CCNB1IP1,SNORD126,PARP2,TEP1,KLHL33,OSGEP,APEX1,PIP4P1,PNP,RNASE10,RNASE9,RNASE11,LOC254028,RNASE12,LOC254028,OR6S1,ANG,RNASE4,RNASE4,EDDM3A,EDDM3B,RNASE6,RNASE1,RNASE3,ECRP,RNASE2,METTL17,METTL17,LOC101929718,SLC39A2,NDRG2,NDRG2,MIR6717,NDRG2,TPPP2,NDRG2,RNASE13,NDRG2,RNASE7,NDRG2,RNASE8,NDRG2,ARHGEF40,ARHGEF40,ARHGEF40,ZNF219,ZNF219,ZNF219,TMEM253,OR5AU1,HNRNPC | 0.226919 | 281.865 | 401 | 241.109 |
| 3 | 22 | 45809322 | 51244066 | SMC1B,RIBC2,FBLN1,ATXN10,ATXN10,MIR4762,WNT7B,LOC730668,PRR34,PRR34,PRR34-AS1,MIRLET7BHG,MIR3619,MIRLET7BHG,MIRLET7BHG,MIRLET7A3,MIRLET7BHG,MIR4763,MIRLET7B,PPARA,CDPF1,PKDREJ,TTC38,GTSE1,TRMU,CELSR1,GRAMD4,CERK,TBC1D22A,TBC1D22A,TBC1D22A-AS1,LINC01644,LINC00898,MIR3201,FAM19A5,FAM19A5,LOC284933,MIR4535,C22orf34,C22orf34,MIR3667,BRD1,ZBED4,ALG12,CRELD2,PIM3,PIM3,MIR6821,IL17REL,TTLL8,MLC1,MOV10L1,PANX2,TRABD,SELENOO,SELENOO,TUBGCP6,TUBGCP6,HDAC10,MAPK12,MAPK11,PLXNB2,DENND6B,PPP6R2,SBF1,ADM2,MIOX,LMF2,NCAPH2,NCAPH2,SCO2,SCO2,TYMP,TYMP,ODF3B,KLHDC7B,SYCE3,CPT1B,CHKB-CPT1B,CHKB-CPT1B,CHKB,MAPK8IP2,ARSA,SHANK3,ACR,RPL23AP82,RABL2B | 0.225331 | 260.984 | 902 | 562.865 |
| 3 | 2 | 95770270 | 96504630 | MRPS5,ZNF514,ZNF2,PROM2,KCNIP3,FAHD2A,TRIM43B,TRIM43 | 0.217368 | 234.952 | 109 | 60.6934 |
| 3 | 16 | 31473707 | 31580552 | ARMC5,TGFB1I1,SLC5A2,SLC5A2,C16orf58,C16orf58,AHSP,FRG2KP,YBX3P1 | 0.216635 | 264.232 | 53 | 33.9031 |
| 3 | 7 | 43846580 | 45927701 | BLVRA,MRPS24,URGCP-MRPS24,URGCP-MRPS24,URGCP,UBE2D4,UBE2D4,POLR2J4,POLR2J4,RASA4CP,LINC00957,DBNL,PGAM2,POLM,MIR6838,POLM,AEBP1,AEBP1,MIR4649,POLD2,MYL7,GCK,YKT6,CAMK2B,NUDCD3,NPC1L1,DDX56,TMED4,OGDH,ZMIZ2,PPIA,H2AFV,PURB,MIR4657,PURB,MYO1G,SNHG15,SNHG15,SNORA9,CCM2,NACAD,TBRG4,TBRG4,SNORA5A,TBRG4,SNORA5C,TBRG4,SNORA5B,RAMP3,ADCY1,SEPT7P2 | 0.211755 | 261.921 | 421 | 253.18 |
| 3 | 6 | 39902671 | 42559343 | LRFN2,UNC5CL,TSPO2,APOBEC2,OARD1,OARD1,NFYA,ADCY10P1,TREML1,TREM2,TREML2,TREML4,TREM1,NCR2,LINC01276,FOXP4,FOXP4,MIR4641,MDFI,TFEB,PGC,FRS3,PRICKLE4,TOMM6,USP49,MED20,BYSL,CCND3,TAF8,C6orf132,GUCA1A,GUCA1B,MRPS10,TRERF1,UBR2 | 0.20877 | 226.056 | 292 | 181.976 |
| 3 | 1 | 86894055 | 91406853 | CLCA2,CLCA1,CLCA4,CLCA4,LOC105378828,LOC105378828,CLCA3P,SH3GLB1,SELENOF,HS2ST1,LINC01140,LMO4,PKN2-AS1,PKN2,PKN2,GTF2B,KYAT3,KYAT3,RBMXL1,GBP3,GBP1,GBP2,GBP7,GBP4,GBP5,GBP6,GBP1P1,LRRC8B,LRRC8C,LRRC8D,ZNF326,BARHL2,ZNF644 | 0.208633 | 315.539 | 362 | 216.972 |

| | | | | | | | | |
|---|---|---|---|---|---|---|---|---|
| 3 | 2 | 96652782 | 97783970 | ANKRD36C,FAHD2CP,FAHD2CP,GPAT2,GPAT2,ADRA2B,ASTL,DUSP2,STARD7,STARD7,STARD7-AS1,STARD7-AS1,TMEM127,CIAO1,SNRNP200,ITPRIPL1,NCAPH,NEURL3,ARID5A,KANSL3,FER1L5,LMAN2L,CNNM4,CNNM4,MIR3127,CNNM3,ANKRD23,ANKRD39,SEMA4C,FAM178B,FAM178B,LOC101927053,FAHD2B,ANKRD36 | 0.207313 | 257.686 | 364 | 224.19 |
| 3 | 4 | 1.71E+08 | 184562095 | HPF1,MFAP3L,AADAT,GALNTL6,LOC101930370,GALNT7,GALNT7,HMGB2,SAP30,SCRG1,HAND2,HAND2,HAND2-AS1,HAND2-AS1,FBXO8,CEP44,MIR4276,HPGD,GLRA3,ADAM29,GPM6A,GPM6A,LOC101928590,WDR17,SPATA4,ASB5,SPCS3,VEGFC,NEIL3,AGA,LINC01098,MIR1305,TENM3,DCTD,WWC2-AS2,WWC2,WWC2,WWC2-AS1,WWC2,CLDN22,CLDN24,CDKN2AIP,ING2 | 0.204785 | 222.715 | 454 | 275.213 |
| 3 | 2 | 72968392 | 75874362 | EXOC6B,SPR,EMX1,SFXN5,RAB11FIP5,NOTO,SMYD5,PRADC1,CCT7,FBXO41,EGR4,ALMS1,NAT8,ALMS1P1,NAT8B,TPRKB,DUSP11,C2orf78,STAMBP,ACTG2,DGUOK,DGUOK,DGUOK-AS1,TET3,BOLA3,MOB1A,MTHFD2,SLC4A5,DCTN1,DCTN1,DCTN1-AS1,C2orf81,WDR54,RTKN,INO80B,INO80B-WBP1,INO80B-WBP1,WBP1,MOGS,MRPL53,CCDC142,CCDC142,TTC31,TTC31,LBX2,LBX2,LBX2-AS1,PCGF1,TLX2,DQX1,AUP1,AUP1,HTRA2,HTRA2,HTRA2,LOXL3,LOXL3,LOXL3,DOK1,DOK1,M1AP,SEMA4F,HK2,POLE4,TACR1,TACR1,MIR5000,EVA1A,MRPL19 | 0.203754 | 264.053 | 628 | 390.419 |
| 3 | 12 | 4919497 | 6948331 | KCNA6,KCNA1,KCNA5,NTF3,ANO2,VWF,CD9,PLEKHG6,TNFRSF1A,SCNN1A,SCNN1A,LTBR,LTBR,CD27-AS1,CD27-AS1,CD27,TAPBPL,TAPBPL,VAMP1,VAMP1,MRPL51,NCAPD2,NCAPD2,SCARNA10,GAPDH,IFFO1,NOP2,CHD4,CHD4,SCARNA11,LPAR5,ACRBP,ING4,ZNF384,PIANP,COPS7A,MLF2,PTMS,LAG3,CD4,GPR162,P3H3 | 0.1995 | 247.006 | 480 | 298.087 |
| 3 | 12 | 1.19E+08 | 122958727 | SUDS3,SRRM4,HSPB8,LINC00934,CCDC60,TMEM233,PRKAB1,CIT,CIT,MIR1178,BICDL1,RAB35,GCN1,GCN1,MIR4498,RPLP0,PXN-AS1,PXN,PXN,SIRT4,PLA2G1B,MSI1,COX6A1,TRIAP1,TRIAP1,GATC,GATC,GATC,SRSF9,SRSF9,DYNLL1,COQ5,RNF10,POP5,CABP1,MLEC,UNC119B,UNC119B,MIR4700,ACADS,SPPL3,HNF1A,HNF1A,C12orf43,C12orf43,OASL,P2RX7,P2RX4,CAMKK2,ANAPC5,RNF34,KDM2B,KDM2B,MIR7107,ORAI1,MORN3,TMEM120B,TMEM120B,RHOF,RHOF,LINC01089,SETD1B,HPD,PSMD9,WDR66,BCL7A,MLXIP,LRRC43,IL31,LRRC43,B3GNT4,B3GNT4,DIABLO,DIABLO,VPS33A,CLIP1,ZCCHC8 | 0.196799 | 263.887 | 754 | 462.972 |
| 3 | 8 | 1.36E+08 | 135812202 | ZFAT,ZFAT,ZFAT-AS1 | 0.195985 | 239.43 | 29 | 17.2295 |

| | | | | | | | | |
|---|---|---|---|---|---|---|---|---|
| 3 | 7 | 65139852 | 74103469 | LOC441242,INTS4P2,LOC441242,CCT6P1,SNORA22,LOC441242,VKORC1L1,GUSB,ASL,CRCP,TPST1,LINC00174,KCTD7,RABGEF1,TMEM248,SBDS,TYW1,TYW1,MIR4650-1,MIR4650-2,PMS2P4,STAG3L4,AUTS2,GALNT17,GALNT17,MIR3914-1,MIR3914-2,CALN1,TYW1B,TYW1B,MIR4650-1,MIR4650-2,SBDSP1,POM121,POM121,NSUN5P2,TRIM74,LOC541473,LOC100101148,GTF2IP4,GTF2IP1,NCF1B,NSUN5,TRIM50,FKBP6,FZD9,BAZ1B,BCL7B,TBL2,MLXIPL,VPS37D,DNAJC30,BUD23,STX1A,STX1A,MIR4284,ABHD11,CLDN3,CLDN4,METTL27,TMEM270,ELN,LIMK1,EIF4H,EIF4H,MIR590,LAT2,RFC2,CLIP2,GTF2IRD1,GTF2I,LOC101926943 | 0.195641 | 233.47 | 653 | 373.81 |
| 3 | 2 | 2.16E+08 | 220585821 | FN1,MREG,PECR,TMEM169,XRCC5,MARCH4,SMARCAL1,RPL37A,IGFBP2,IGFBP5,TNP1,LINC01921,DIRC3,TNS1,TNS1,MIR6809,LOC105373878,RUFY4,CXCR2P1,RUFY4,CXCR2,CXCR1,ARPC2,GPBAR1,AAMP,PNKD,PNKD,TMBIM1,PNKD,TMBIM1,MIR6513,PNKD,CATIP-AS2,PNKD,CATIP-AS2,MIR6810,CATIP-AS2,CATIP,CATIP,CATIP,CATIP-AS1,SLC11A1,CTDSP1,CTDSP1,MIR26B,VIL1,USP37,CNOT9,PLCD4,ZNF142,BCS1L,RNF25,RNF25,STK36,STK36,TTLL4,CYP27A1,PRKAG3,WNT6,WNT10A,CDK5R2,FEV,CRYBA2,MIR375,LOC100129175,CFAP65,CFAP65,IHH,IHH,MIR3131,NHEJ1,SLC23A3,CNPPD1,RETREG2,ZFAND2B,ABCB6,ATG9A,ANKZF1,GLB1L,STK16,STK16,TUBA4A,TUBA4A,TUBA4A,TUBA4B,TUBA4B,DNAJB2,PTPRN,PTPRN,MIR153-1,RESP18,DNPEP,DES,SPEG,LOC100996693,GMPPA,ASIC4,CHPF,TMEM198,TMEM198,MIR3132,OBSL1,INHA,STK11IP,SLC4A3 | 0.19362 | 251.034 | 994 | 623.633 |
| 3 | 22 | 37962389 | 38945392 | CDC42EP1,LGALS2,GGA1,SH3BP1,SH3BP1,LOC101927051,PDXP,LGALS1,NOL12,TRIOBP,H1F0,GCAT,GALR3,ANKRD54,ANKRD54,MIR658,ANKRD54,MIR659,ANKRD54,EIF3L,EIF3L,MICALL1,C22orf23,POLR2F,POLR2F,MIR6820,POLR2F,SOX10,POLR2F,MIR4534,PICK1,SLC16A8,BAIAP2L2,PLA2G6,MAFF,TMEM184B,CSNK1E,LOC400927-CSNK1E,KCNJ4,KDELR3,DDX17,DMC1 | 0.190968 | 253.477 | 337 | 205.326 |
| 3 | 22 | 20043434 | 20508931 | TANGO2,DGCR8,MIR3618,DGCR8,MIR1306,DGCR8,TRMT2A,TRMT2A,MIR6816,TRMT2A,RANBP1,RANBP1,RANBP1,SNORA77B,ZDHHC8,CCDC188,LINC00896,RTN4R,RTN4R,MIR1286,DGCR6L,LOC101927859,TMEM191B,LOC101927859,PI4KAP1 | 0.1811 | 221.156 | 106 | 60.9041 |

| | | | | | | | | |
|---|---|---|---|---|---|---|---|---|
| 3 | 19 | 13317371 | 19904847 | CACNA1A,CCDC130,MRI1,C19orf53,ZSWIM4,LOC284454,MIR24-2,MIR27A,MIR23A,NANOS3,MIR181C,MIR181D,NANOS3,C19orf57,CC2D1A,PODNL1,PODNL1,DCAF15,DCAF15,RFX1,RLN3,IL27RA,PALM3,MISP3,MISP3,MIR1199,C19orf67,SAMD1,PRKACA,ASF1B,LOC100507373,ADGRL1,ADGRL1,ADGRE5,DDX39A,PKN1,PTGER1,GIPC1,DNAJB1,MIR639,TECR,TECR,NDUFB7,CLEC17A,ADGRE3,ZNF333,ADGRE2,OR7C1,OR7A5,OR7A10,OR7A17,OR7C2,SLC1A6,CCDC105,CASP14,OR1I1,SYDE1,ILVBL,NOTCH3,NOTCH3,MIR6795,EPHX3,BRD4,AKAP8,AKAP8L,WIZ,RASAL3,PGLYRP2,CYP4F22,CYP4F8,CYP4F3,CYP4F12,OR10H2,OR10H3,CYP4F24P,OR10H5,OR10H1,CYP4F2,CYP4F11,OR10H4,LINC00661,LINC00905,TPM4,RAB8A,HSH2D,CIB3,FAM32A,AP1M1,KLF2,EPS15L1,CALR3,C19orf44,C19orf44,CHERP,CHERP,SLC35E1,MED26,SMIM7,TMEM38A,NWD1,SIN3B,F2RL3,CPAMD8,HAUS8,MYO9B,USE1,OCEL1,NR2F6,USHBP1,BABAM1,ANKLE1,ABHD8,MRPL34,DDA1,ANO8,GTPBP3,PLVAP,BST2,MVB12A,TMEM221,NXNL1,SLC27A1,SLC27A1,LOC100507551,LOC100507551,PGLS,PGLS,FAM129C,COLGALT1,UNC13A,MAP1S,FCHO1,B3GNT3,INSL3,JAK3,RPL18A,RPL18A,SNORA68,SLC5A5,CCDC124,KCNN1,ARRDC2,IL12RB1,IL12RB1,MAST3,MAST3,PIK3R2,IFI30,MPV17L2,RAB3A,PDE4C,IQCN,JUND,MIR3188,LSM4,PGPEP1,GDF15,GDF15,MIR3189,LRRC25,SSBP4,ISYNA1,ELL,FKBP8,KXD1,UBA52,REX1BD,CRLF1,TMEM59L,KLHL26,CRTC1,COMP,UPF1,CERS1,GDF1,COPE,DDX49,HOMER3,SUGP2,SUGP2,ARMC6,ARMC6,SLC25A42,TMEM161A,MEF2B,BORCS8-MEF2B,BORCS8-MEF2B,BORCS8,RFXANK,RFXANK,NR2C2AP,NR2C2AP,NCAN,HAPLN4,TM6SF2,SUGP1,MAU2,GATAD2A,MIR640,GATAD2A,TSSK6,NDUFA13,YJEFN3,CILP2,PBX4,LPA | 0.172451 | 262.386 | 2178 | 1343.47 |
| 3 | 1 | 2.1E+08 | 225139863 | DIEXF,SYT14,SERTAD4-AS1,SERTAD4-AS1,SERTAD4,SERTAD4,HHAT,KCNH1,RCOR3,TRAF5,LINC00467,RD3,SLC30A1,NEK2,LPGAT1,INTS7,DTL,DTL,MIR3122,PPP2R5A,PPP2R5A,SNORA16B,TMEM206,NENF,ATF3,FAM71A,BATF3,NSL1,TATDN3,SPATA45,FLVCR1,VASH2,ANGEL2,RPS6KC1,PROX1,SMYD2,PTPN14,CENPF,KCNK2,KCTD3,USH2A,USH2A,LOC102723833,ESRRG,GPATCH2,SPATA17,SPATA17,SPATA17-AS1,RRP15,TGFB2,TGFB2,MIR548F3,MIR548F3,C1orf143,LYPLAL1,LOC102723886,ZC3H11B,RNU5F-1,SLC30A10,RNU5F-1,EPRS,RNU5F-1,BPNT1,RNU5F-1,IARS2,RNU5F-1,IARS2,MIR215,RNU5F-1,IARS2,MIR194-1,IARS2,RAB3GAP2,RAB3GAP2,MIR664A,SNORA36B,MARK1,C1orf115,MARC2,MARC1,HLX-AS1,HLX,HLX,C1orf140,DUSP10,HHIPL2,TAF1A,MIA3,AIDA,BROX,FAM177B,DISP1,TLR5,SUSD4,CCDC185,CAPN8,CAPN2,TP53BP2,LOC100287497,FBXO28,DEGS1,LOC101927164,NVL,NVL,NVL,MIR320B2,CNIH4,WDR26,WDR26,MIR4742,CNIH3,DNAH14 | 0.169549 | 273.45 | 1259 | 758.43 |

| | | | | | | | | |
|---|---|---|---|---|---|---|---|---|
| 3 | 14 | 21851714 | 24624755 | SUPT16H,CHD8,CHD8,SNORD9,CHD8,SNORD8,RAB2B,TOX4,TOX4,METTL3,METTL3,SALL2,OR10G3,OR10G2,OR4E2,LOC105370401,LINC02332,DAD1,ABHD4,OR6J1,OXA1L,SLC7A7,MRPL52,MMP14,LRP10,REM2,RBM23,PRMT5-AS1,PRMT5,PRMT5,LOC101926933,HAUS4,HAUS4,HAUS4,MIR4707,AJUBA,C14orf93,PSMB5,PSMB11,CDH24,ACIN1,C14orf119,LMLN2,CEBPE,SLC7A8,RNF212B,HOMEZ,PPP1R3E,BCL2L2,BCL2L2-PABPN1,BCL2L2-PABPN1,BCL2L2-PABPN1,PABPN1,SLC22A17,EFS,IL25,CMTM5,MYH6,MYH6,MIR208A,MYH7,MYH7,MHRT,MYH7,MIR208B,NGDN,THTPA,ZFHX2,THTPA,AP1G2,AP1G2,LOC102724814,JPH4,DHRS2,DHRS4-AS1,DHRS4-AS1,DHRS4,DHRS4,DHRS4L2,DHRS4L1,CARMIL3,CPNE6,NRL,NRL,PCK2,DCAF11,FITM1,PSME1,EMC9,PSME2,MIR7703,PSME2,RNF31 | 0.168373 | 251.457 | 1008 | 619.534 |
| 3 | 16 | 88816421 | 90294253 | PIEZO1,CDT1,APRT,GALNS,GALNS,TRAPPC2L,TRAPPC2L,PABPN1L,CBFA2T3,CBFA2T3,LOC101927793,CBFA2T3,LOC100129697,ACSF3,LINC02138,CDH15,SLC22A31,ZNF778,ANKRD11,ANKRD11,LOC100287036,SPG7,RPL13,RPL13,SNORD68,CPNE7,DPEP1,CHMP1A,CHMP1A,SPATA33,SPATA33,CDK10,SPATA2L,VPS9D1,VPS9D1,VPS9D1-AS1,VPS9D1,ZNF276,ZNF276,ZNF276,FANCA,FANCA,SPIRE2,TCF25,MC1R,TUBB3,DEF8,CENPBD1,AFG3L1P,DBNDD1,DBNDD1,GAS8,GAS8,GAS8,GAS8-AS1,GAS8,URAHP,PRDM7 | 0.161196 | 257.464 | 474 | 291.125 |
| 3 | 3 | 56716629 | 57231704 | FAM208A,ARHGEF3,ARHGEF3,ARHGEF3-AS1,ARHGEF3,SPATA12,IL17RD | 0.157853 | 209.125 | 60 | 35.426 |

| | | | | | | | | |
|---|---|---|---|---|---|---|---|---|
| 3 | 19 | 1592511 | 12768991 | MBD3,UQCR11,TCF3,ONECUT3,ATP8B3,REXO1,REXO1,MIR1909,REXO1,LOC100288123,KLF16,ABHD17A,ADAT3,SCAMP4,SCAMP4,CSNK1G2,CSNK1G2-AS1,CSNK1G2,BTBD2,MKNK2,MOB3A,IZUMO4,AP3D1,DOT1L,PLEKHJ1,MIR1227,PLEKHJ1,MIR6789,PLEKHJ1,SF3A2,AMH,AMH,MIR4321,JSRP1,OAZ1,PEAK3,LINGO3,LSM7,SPPL2B,TMPRSS9,TMPRSS9,TIMM13,TIMM13,LMNB2,LMNB2,MIR7108,GADD45B,GNG7,DIRAS1,SLC39A3,SGTA,THOP1,ZNF554,ZNF555,ZNF556,ZNF57,ZNF77,TLE6,TLE2,AES,GNA11,GNA15,GNA15,LOC100996351,S1PR4,NCLN,CELF5,NFIC,SMIM24,DOHH,FZR1,C19orf71,MFSD12,HMG20B,GIPC3,TBXA2R,CACTIN-AS1,CACTIN,CACTIN,PIP5K1C,TJP3,TJP3,MIR1268A,MIR1268A,APBA3,MIR1268A,MRPL54,MIR1268A,RAX2,MIR1268A,MATK,MIR1268A,ZFR2,MIR1268A,ATCAY,MIR1268A,NMRK2,MIR1268A,DAPK3,MIR1268A,DAPK3,MIR637,MIR1268A,EEF2,MIR1268A,EEF2,SNORD37,PIAS4,ZBTB7A,MAP2K2,CREB3L3,SIRT6,ANKRD24,EBI3,CCDC94,SHD,TMIGD2,FSD1,STAP2,MPND,SH3GL1,CHAF1A,UBXN6,UBXN6,MIR4746,HDGFL2,HDGFL2,PLIN4,PLIN4,PLIN5,LRG1,SEMA6B,TNFAIP8L1,MYDGF,DPP9,DPP9,DPP9-AS1,MIR7-3HG,MIR7-3HG,MIR7-3,FEM1A,TICAM1,PLIN3,ARRDC5,UHRF1,UHRF1,MIR4747,KDM4B,PTPRS,ZNRF4,SAFB2,SAFB,C19orf70,HSD11B1L,RPL36,LONP1,CATSPERD,PRR22,DUS3L,NRTN,FUT6,FUT3,FUT5,NDUFA11,VMAC,CAPS,RANBP3,LOC100128568,RFX2,RFX2,ACSBG2,MLLT1,ACER1,CLPP,ALKBH7,PSPN,GTF2F1,GTF2F1,MIR6885,GTF2F1,MIR6790,LOC390877,KHSRP,KHSRP,MIR3940,SLC25A41,SLC25A23,CRB3,DENND1C,TUBB4A,TNFSF9,CD70,TNFSF14,C3,GPR108,GPR108,MIR6791,TRIP10,SH2D3A,VAV1,ADGRE1,ADGRE4P,FLJ25758,MBD3L2,ZNF557,INSR,ARHGEF18,LOC100128573,PEX11G,TEX45,ZNF358,MCOLN1,P | 0.1569 | 259.1 | 3843 | 2368.43 |
| 3 | 1 | 31845742 | 33583672 | FABP3,SERINC2,LINC01225,TINAGL1,HCRTR1,PEF1,COL16A1,ADGRB2,ADGRB2,MIR4254,SPOCD1,PTP4A2,KHDRBS1,TMEM39B,TMEM39B,MIR5585,KPNA6,TXLNA,CCDC28B,IQCC,DCDC2B,DCDC2B,TMEM234,TMEM234,TMEM234,EIF3I,EIF3I,FAM167B,LCK,HDAC1,MARCKSL1,FAM229A,TSSK3,BSDC1,ZBTB8B,ZBTB8A,ZBTB8OS,RBBP4,RBBP4,SYNC,SYNC,KIAA1522,YARS,S100PBP,FNDC5,HPCA,TMEM54,RNF19B,AK2,AZIN2 | 0.149294 | 241.93 | 474 | 293.924 |
| 3 | 16 | 3254180 | 4415106 | OR1F1,OR1F2P,ZNF200,MEFV,ZNF263,TIGD7,ZNF75A,OR2C1,ZSCAN32,ZNF174,ZNF597,NAA60,NAA60,MIR6126,C16orf90,CLUAP1,NLRC3,SLX4,DNASE1,TRAP1,CREBBP,ADCY9,SRL,TFAP4,GLIS2,PAM16,CORO7-PAM16,CORO7-PAM16,CORO7 | 0.149197 | 243.387 | 330 | 210.436 |
| 3 | 2 | 27688587 | 27898601 | IFT172,FNDC4,GCKR,C2orf16,ZNF512,CCDC121,CCDC121,GPN1,GPN1,SUPT7L,SUPT7L,SLC4A1AP,SLC4A1AP | 0.147536 | 267.105 | 132 | 84.5356 |

| | | | | | | | | |
|---|---|---|---|---|---|---|---|---|
| 3 | 20 | 58498018 | 62959313 | FAM217B,PPP1R3D,FAM217B,CDH26,C20orf197,MIR646HG,MIR646,MIR4533,MIR548AG2,CDH4,CDH4,LOC100128310,MIR1257,TAF4,LSM14B,PSMA7,SS18L1,MTG2,HRH3,OSBPL2,ADRM1,LAMA5,LAMA5,MIR4758,LAMA5,LAMA5-AS1,RPS21,CABLES2,RBBP8NL,GATA5,MIR1-1HG-AS1,MIR1-1HG,MIR1-1HG,MIR1-1,MIR1-1HG,MIR133A2,SLCO4A1,SLCO4A1,SLCO4A1-AS1,NTSR1,MRGBP,OGFR-AS1,OGFR,OGFR,COL9A3,TCFL5,TCFL5,DPH3P1,DIDO1,GID8,SLC17A9,BHLHE23,LINC01749,MIR124-3,YTHDF1,BIRC7,BIRC7,MIR3196,NKAIN4,NKAIN4,FLJ16779,ARFGAP1,ARFGAP1,MIR4326,COL20A1,CHRNA4,CHRNA4,LOC100130587,KCNQ2,EEF1A2,PPDPF,PTK6,SRMS,FNDC11,HELZ2,GMEB2,STMN3,RTEL1,RTEL1-TNFRSF6B,RTEL1-TNFRSF6B,TNFRSF6B,ARFRP1,ZGPAT,ZGPAT,LIME1,LIME1,SLC2A4RG,ZBTB46,ABHD16B,TPD52L2,DNAJC5,UCKL1,UCKL1,MIR1914,UCKL1,MIR647,UCKL1,UCKL1-AS1,ZNF512B,SAMD10,PRPF6,C20orf204,SOX18,TCEA2,RGS19,RGS19,MIR6813,OPRL1,LKAAEAR1,OPRL1,NPBWR2,MYT1,PCMTD2 | 0.146164 | 259.074 | 913 | 560.761 |
| 3 | 12 | 1.23E+08 | 133841395 | HCAR2,HCAR3,HCAR1,DENR,CCDC62,HIP1R,VPS37B,ABCB9,OGFOD2,ARL6IP4,PITPNM2,PITPNM2,MIR4304,MPHOSPH9,C12orf65,CDK2AP1,SBNO1,KMT5A,RILPL2,SNRNP35,RILPL1,MIR3908,TMED2,DDX55,EIF2B1,GTF2H3,TCTN2,ATP6V0A2,DNAH10,CCDC92,ZNF664-RFLNA,ZNF664,ZNF664-RFLNA,RFLNA,NCOR2,NCOR2,MIR6880,SCARB1,UBC,MIR5188,DHX37,BRI3BP,AACS,TMEM132B,LOC283435,LINC02347,LINC02347,LINC00944,LINC00943,TMEM132C,TMEM132C,MIR3612,SLC15A4,GLT1D1,TMEM132D,TMEM132D,TMEM132D-AS2,LINC02418,FZD10,PIWIL1,RIMBP2,STX2,RAN,ADGRD1,ADGRD1,ADGRD1-AS1,LINC01257,SFSWAP,MMP17,ULK1,PUS1,EP400,EP400,SNORA49,EP400P1,DDX51,NOC4L,GALNT9,GALNT9,LOC100130238,FBRSL1,LRCOL1,P2RX2,POLE,PXMP2,PGAM5,ANKLE2,GOLGA3,CHFR,ZNF605,ZNF26,ZNF84,ZNF140,ZNF891,ZNF10,ZNF268,ANHX | 0.145151 | 238.549 | 1287 | 780.18 |
| 3 | 22 | 38962547 | 39636883 | DMC1,FAM227A,CBY1,TOMM22,JOSD1,GTPBP1,SUN2,DNAL4,NPTXR,CBX6,APOBEC3A,APOBEC3A_B,APOBEC3A_B,APOBEC3A_B,APOBEC3B,APOBEC3A_B,APOBEC3B,APOBEC3B-AS1,APOBEC3C,APOBEC3D,APOBEC3F,APOBEC3G,APOBEC3H,CBX7,PDGFB | 0.142182 | 233.116 | 183 | 105.022 |
| 3 | 9 | 10500 | 2719107 | CBWD1,C9orf66,C9orf66,DOCK8,DOCK8,KANK1,DMRT1,DMRT3,DMRT2,SMARCA2,VLDLR-AS1,VLDLR,VLDLR,KCNV2 | 0.140266 | 241.697 | 203 | 125.359 |

| | | | | | | | | |
|---|---|---|---|---|---|---|---|---|
| 3 | 9 | 88937271 | 93627033 | ZCCHC6,GAS1,C9orf170,DAPK1,CTSL,CTSL3P,CTSLP8,LOC392364,LOC497256,SPATA31E1,LOC497256,SPATA31C1,CDK20,SPATA31C2,SPIN1,NXNL2,MIR4289,C9orf47,C9orf47,S1PR3,S1PR3,SHC3,CKS2,CKS2,MIR3153,SECISBP2,SEMA4D,GADD45G,MIR4290HG,MIR4290,DIRAS2,SYK | 0.13937 | 247.84 | 265 | 160.189 |
| 3 | 3 | 45877082 | 48448358 | LZTFL1,LZTFL1,CCR9,FYCO1,FYCO1,CXCR6,XCR1,CCR1,CCR3,CCR2,LOC102724297,CCR5,CCRL2,LTF,RTP3,LRRC2,TDGF1,FAM240A,ALS2CL,TMIE,PRSS50,PRSS46P,PRSS45,PRSS42,MYL3,PTH1R,CCDC12,NBEAL2,NRADDP,SETD2,KIF9-AS1,KIF9,KIF9,KLHL18,PTPN23,SCAP,ELP6,CSPG5,SMARCC1,DHX30,DHX30,MIR1226,MAP4,CDC25A,MIR4443,CAMP,ZNF589,NME6,SPINK8,SPINK8,MIR2115,FBXW12,PLXNB1 | 0.131398 | 250.566 | 610 | 391.458 |
| 3 | 6 | 27879002 | 30630871 | OR2B2,OR2B6,ZNF165,ZSCAN12P1,ZSCAN16-AS1,ZSCAN16-AS1,ZSCAN16,ZKSCAN8,ZNF192P1,ZSCAN9,ZKSCAN4,NKAPL,ZSCAN26,PGBD1,ZSCAN31,ZSCAN31,ZKSCAN3,ZKSCAN3,ZSCAN12,ZSCAN23,GPX6,GPX5,ZBED9,TRIM27,LINC01556,ZNF311,LOC100129636,OR2W1,LOC100129636,OR2B3,OR2J1,OR2J3,OR2J2,LOC101929006,OR14J1,OR5V1,OR12D3,OR12D2,OR12D1,OR11A1,OR10C1,OR2H1,MAS1L,UBD,SNORD32B,OR2H2,GABBR1,MOG,ZFP57,HLA-F,HLA-F,HLA-F-AS1,HLA-F-AS1,HCG4,HCG4,HLA-V,HLA-V,HLA-G,HLA-H,HLA-A,HCG9,ZNRD1ASP,HLA-J,ZNRD1ASP,ZNRD1,PPP1R11,RNF39,TRIM31,TRIM31,TRIM31-AS1,TRIM40,TRIM10,TRIM15,TRIM26,HCG17,HLA-L,HCG17,HLA-L,MIR6891,TRIM39,TRIM39-RPP21,TRIM39-RPP21,RPP21,HLA-E,GNL1,GNL1,PRR3,PRR3,ABCF1,ABCF1,MIR877,PPP1R10,MRPS18B,ATAT1,C6orf136,DHX16 | 0.123974 | 259.823 | 594 | 361.74 |
| 3 | 4 | 438820 | 2194326 | ABCA11P,ZNF721,PIGG,PDE6B,ATP5ME,MYL5,MYL5,SLC49A3,SLC49A3,PCGF3,LOC100129917,CPLX1,GAK,TMEM175,DGKQ,SLC26A1,SLC26A1,IDUA,IDUA,FGFRL1,RNF212,SPON2,SPON2,LOC100130872,CTBP1-AS,CTBP1,CTBP1,CTBP1-AS2,MAEA,UVSSA,CRIPAK,NKX1-1,FAM53A,SLBP,TMEM129,TACC3,FGFR3,LETM1,NSD2,NSD2,SCARNA22,NELFA,NELFA,MIR943,C4orf48,NAT8L,POLN | 0.120583 | 252.934 | 444 | 274.159 |

| | | | | | | | | |
|---|---|---|---|---|---|---|---|---|
| 3 | 22 | 40030566 | 45757898 | CACNA1I,ENTHD1,GRAP2,FAM83F,TNRC6B,ADSL,SGSM3,MKL1,MCHR1,SLC25A17,SLC25A17,MIR4766,ST13,XPNPEP3,XPNPEP3,DNAJB7,RBX1,MIR1281,EP300,EP300,L3MBTL2,L3MBTL2,LOC100506544,L3MBTL2,CHADL,CHADL,RANGAP1,ZC3H7B,TEF,TOB2,PHF5A,ACO2,ACO2,POLR3H,POLR3H,CSDC2,PMM1,DESI1,XRCC6,SNU13,C22orf46,MEI1,CCDC134,SREBF2,SREBF2,MIR33A,SHISA8,MIR378I,TNFRSF13C,CENPM,LINC00634,SEPT3,WBP2NL,NAGA,FAM109B,SMDT1,NDUFA6,LOC101929829,CYP2D6,CYP2D7,TCF20,NFAM1,SERHL,SERHL,RRP7A,RRP7A,SERHL2,SERHL2,RRP7BP,RRP7BP,POLDIP3,CYB5R3,CYB5R3,ATP5MGL,A4GALT,ARFGAP3,PACSIN2,LOC100506679,TTLL1,TTLL1,BIK,MCAT,TSPO,TTLL12,FLJ30901,SCUBE1,MPPED1,EFCAB6-AS1,EFCAB6,EFCAB6,SULT4A1,PNPLA5,PNPLA3,SAMM50,PARVB,PARVG,SHISAL1,RTL6,LINC00207,PRR5,PRR5,PRR5-ARHGAP8,PRR5-ARHGAP8,ARHGAP8,PHF21B,NUP50,NUP50,LOC105373064,KIAA0930,KIAA0930,MIR1249,UPK3A,FAM118A,SMC1B | 0.119409 | 233.505 | 1107 | 660.981 |
| 3 | 12 | 1.12E+08 | 118650185 | ATXN2,BRAP,ACAD10,ALDH2,ALDH2,MIR6761,MAPKAPK5-AS1,MAPKAPK5,MAPKAPK5,ADAM1A,TMEM116,ERP29,NAA25,NAA25,MIR3657,TRAFD1,HECTD4,HECTD4,MIR6861,RPL6,PTPN11,RPH3A,MIR1302-1,RPH3A,OAS1,OAS3,OAS2,DTX1,RASAL1,CFAP73,CFAP73,DDX54,CFAP73,DDX54,MIR7106,DDX54,RITA1,IQCD,TPCN1,TPCN1,MIR6762,SLC8B1,PLBD2,SDS,SDSL,LHX5,RBM19,TBX5,TBX3,MED13L,MED13L,MIR620,LINC00173,MAP1LC3B2,C12orf49,RNFT2,HRK,FBXW8,FBXW8,LOC100506551,TESC,FBXO21,NOS1,KSR2,RFC5,WSB2,VSIG10,PEBP1,TAOK3 | 0.101433 | 233.467 | 835 | 505.779 |
| 3 | 7 | 1.51E+08 | 159128163 | NOB1,WDR86,WDR86,WDR86-AS1,WDR86-AS1,CRYGN,CRYGN,MIR3907,RHEB,PRKAG2,PRKAG2,LOC644090,GALNTL5,GALNT11,KMT2C,XRCC2,ACTR3B,DPP6,PAXIP1-AS2,PAXIP1-AS2,PAXIP1,PAXIP1,HTR5A-AS1,HTR5A-AS1,HTR5A,HTR5A,INSIG1,BLACE,LOC100286906,EN2,CNPY1,LOC100506302,RBM33,SHH,LOC389602,C7orf13,RNF32,RNF32,LMBR1,LMBR1,NOM1,MNX1,MNX1,MNX1-AS2,UBE3C,DNAJB6,LOC101927914,PTPRN2,PTPRN2,MIR153-2,PTPRN2,MIR595,LINC01022,MIR5707,NCAPG2,ESYT2,WDR60,VIPR2 | 0.0926546 | 226.078 | 711 | 419.084 |
| 3 | 2 | 84921381 | 86334415 | DNAH6,TRABD2A,TMSB10,KCMF1,TCF7L1,TGOLN2,RETSAT,ELMOD3,CAPG,SH2D6,MAT2A,GGCX,VAMP8,VAMP5,RNF181,TMEM150A,USP39,C2orf68,USP39,SFTPB,GNLY,ATOH8,ST3GAL5,POLR1A,PTCD3 | 0.0904795 | 235.944 | 278 | 170.183 |

| | | | | | | | | |
|---|---|---|---|---|---|---|---|---|
| 3 | 7 | 1.27E+08 | 141720880 | ZNF800,GCC1,ARF5,FSCN3,PAX4,SND1,SND1,SND1-IT1,SND1,LRRC4,SND1,MIR593,MIR129-1,LEP,RBM28,PRRT4,IMPDH1,HILPDA,METTL2B,LINC01000,FAM71F2,FAM71F1,CALU,CALU,OPN1SW,OPN1SW,CCDC136,FLNC,FLNC,FLNC-AS1,ATP6V1F,LOC100130705,KCP,IRF5,TNPO3,LOC407835,TSPAN33,SMO,AHCYL2,STRIP2,SMKR1,NRF1,MIR182,MIR96,MIR183,UBE2H,ZC3HC1,KLHDC10,TMEM209,SSMEM1,CPA2,CPA4,CPA5,CPA1,CEP41,MEST,MEST,MIR335,COPG2,COPG2,TSGA13,TSGA13,KLF14,MIR29A,MIR29B1,LINC-PINT,MKLN1,PODXL,PLXNA4,FLJ40288,CHCHD3,EXOC4,EXOC4,LOC101928861,LRGUK,SLC35B4,AKR1B1,AKR1B10,AKR1B15,BPGM,CALD1,AGBL3,AGBL3,C7orf49,C7orf49,C7orf49,TMEM140,WDR91,WDR91,MIR6509,STRA8,CNOT4,NUP205,STMP1,SLC13A4,FAM180A,LUZP6,MTPN,CHRM2,LOC349160,MIR490,CHRM2,LOC349160,PTN,DGKI,CREB3L2,CREB3L2,LOC100130880,AKR1D1,MIR4468,TRIM24,SVOPL,ATP6V0A4,ATP6V0A4,TMEM213,TMEM213,KIAA1549,ZC3HAV1L,ZC3HAV1,TTC26,UBN2,LUC7L2,FMC1,C7orf55-LUC7L2,LUC7L2,C7orf55-LUC7L2,LUC7L2,C7orf55-LUC7L2,LOC100129148,KLRG2,CLEC2L,HIPK2,TBXAS1,PARP12,KDM7A,SLC37A3,RAB19,MKRN1,DENND2A,ADCK2,NDUFB2-AS1,NDUFB2-AS1,NDUFB2,NDUFB2,BRAF,MRPS33,TMEM178B,AGK,KIAA1147,WEE2-AS1,WEE2,SSBP1,TAS2R3,TAS2R4,TAS2R5,PRSS37,OR9A4,CLEC5A,TAS2R38,MGAM | 0.0904218 | 235.997 | 1767 | 1052.51 |
| 3 | 10 | 42379857 | 51877836 | CCNYL2,ZNF33B,BMS1,MIR5100,RET,CSGALNACT2,RASGEF1A,FXYD4,HNRNPF,ZNF487,ZNF239,ZNF485,ZNF32-AS3,ZNF32,ZNF32-AS1,ZNF32-AS3,ZNF32,ZNF32-AS2,HNRNPA3P1,LINC00619,CXCL12,TMEM72-AS1,TMEM72,RASSF4,RASSF4,DEPP1,C10orf25,C10orf25,ZNF22,ZNF22,RSU1P2,ANKRD30BP3,MIR3156-1,OR13A1,ALOX5,ALOX5,LOC102724323,MARCH8,ZFAND4,WASHC2C,PTPN20,FAM35BP,LOC102724593,LOC102724593,SYT15,SYT15,GPRIN2,NPY4R,NPY4R2,ANXA8,FAM35DP,ANTXRL,ANXA8L1,ZNF488,RBP3,GDF2,GDF10,FRMPD2,MAPK8,ARHGAP22,WDFY4,WDFY4,LRRC18,MIR4294,VSTM4,FAM170B-AS1,FAM170B,C10orf128,C10orf71,DRGX,ERCC6,ERCC6,PGBD3,CHAT,SLC18A3,CHAT,C10orf53,OGDHL,PARG,PARG,PARGP1,TIMM23B,TIMM23B,MSMB,TIMM23B,NCOA4,TIMM23B,TIMM23,WASHC2A | 0.0882613 | 245.852 | 829 | 477.423 |
| 3 | 3 | 49210257 | 49568304 | KLHDC8B,C3orf84,CCDC36,C3orf62,C3orf62,MIR4271,USP4,GPX1,RHOA,TCTA,AMT,AMT,NICN1,NICN1,DAG1 | 0.0877403 | 238.104 | 102 | 60.6524 |

| | | | | | | | | |
|---|---|---|---|---|---|---|---|---|
| 3 | 3 | 5220234 | 15252429 | ARL8B,EDEM1,MIR4790,GRM7-AS3,GRM7,LMCD1-AS1,LMCD1,SSUH2,CAV3,OXTR,RAD18,SRGAP3,THUMPD3,SETD5,LHFPL4,MTMR14,CPNE9,BRPF1,OGG1,OGG1,CAMK1,CAMK1,TADA3,TADA3,ARPC4,ARPC4-TTLL3,ARPC4,ARPC4-TTLL3,ARPC4-TTLL3,TTLL3,RPUSD3,CIDEC,JAGN1,IL17RE,IL17RC,CRELD1,PRRT3,PRRT3,PRRT3-AS1,EMC3,EMC3,EMC3-AS1,FANCD2,FANCD2,FANCD2OS,FANCD2OS,BRK1,VHL,IRAK2,TATDN2,GHRLOS,LINC00852,GHRL,GHRLOS,GHRL,SEC13,ATP2B2,ATP2B2,MIR378B,ATP2B2,MIR885,SLC6A11,SLC6A1,SLC6A1-AS1,SLC6A1,HRH1,ATG7,ATG7,VGLL4,VGLL4,TAMM41,SYN2,SYN2,TIMP4,PPARG,TSEN2,MKRN2OS,MKRN2,RAF1,TMEM40,CAND2,RPL32,RPL32,SNORA7A,IQSEC1,NUP210,HDAC11,FBLN2,WNT7A,TPRXL,CHCHD4,TMEM43,XPC,LSM3,SLC6A6,GRIP2,CCDC174,C3orf20,FGD5,NR2C2,NR2C2,MRPS25,MRPS25,RBSN,COL6A4P1,CAPN7 | 0.0857664 | 235.457 | 1182 | 722.078 |
| 3 | 2 | 2.39E+08 | 243188873 | UBE2F,UBE2F-SCLY,UBE2F-SCLY,SCLY,ESPNL,KLHL30,ERFE,ILKAP,LOC151174,LOC643387,HES6,PER2,TRAF3IP1,ASB1,TWIST2,HDAC4,HDAC4,MIR4440,HDAC4,MIR4269,HDAC4,MIR2467,LOC101928111,LOC150935,NDUFA10,NDUFA10,MIR4786,OR6B2,OR6B3,COPS9,OTOS,GPC1,GPC1,PP14571,GPC1,PP14571,MIR149,ANKMY1,ANKMY1,DUSP28,DUSP28,RNPEPL1,CAPN10,GPR35,AQP12B,LOC285191,AQP12A,KIF1A,AGXT,C2orf54,CROCC2,CROCC2,LOC200772,SNED1,SNED1,MTERF4,MTERF4,PASK,PASK,PPP1R7,PPP1R7,ANO7,HDLBP,SEPT2,FARP2,FARP2,MIR3133,STK25,BOK,THAP4,ATG4B,DTYMK,ING5,D2HGDH,GAL3ST2,NEU4,PDCD1,RTP5,LINC01237,LOC285095,LINC01237,LOC285097,LINC01881 | 0.0853213 | 233.523 | 784 | 480.792 |
| 3 | 12 | 1.06E+08 | 111946806 | APPL2,C12orf75,NUAK1,CKAP4,TCP11L2,POLR3B,POLR3B,LOC100287944,LOC100287944,RFX4,LOC100287944,RFX4,LOC100505978,LOC100287944,RIC8B,RIC8B,TMEM263,MTERF2,CRY1,BTBD11,PWP1,PRDM4,PRDM4,LOC101929162,ASCL4,WSCD2,CMKLR1,FICD,SART3,ISCU,TMEM119,SELPLG,MIR4496,CORO1C,SSH1,SSH1,MIR619,DAO,SVOP,USP30,USP30-AS1,USP30,ALKBH2,UNG,ACACB,FOXN4,MYO1H,KCTD10,UBE3B,MMAB,MVK,FAM222A,FAM222A-AS1,TRPV4,TRPV4,MIR4497,GLTP,TCHP,GIT2,ANKRD13A,C12orf76,IFT81,ATP2A2,ANAPC7,ARPC3,GPN3,GPN3,FAM216A,FAM216A,VPS29,RAD9B,PPTC7,TCTN1,TCTN1,HVCN1,HVCN1,PPP1CC,CCDC63,MYL2,LINC01405,CUX2,FAM109A,SH2B3,ATXN2 | 0.0845005 | 231.66 | 959 | 581.652 |

| | | | | | | | | |
|---|---|---|---|---|---|---|---|---|
| 3 | 1 | 1.1E+08 | 112240172 | TAF13,TMEM167B,SCARNA2,C1orf194,KIAA1324,SARS,CELSR2,PSRC1,MYBPHL,SORT1,PSMA5,SYPL2,ATXN7L2,CYB561D1,AMIGO1,GPR61,GNAI3,MIR197,GNAT2,AMPD2,GSTM4,GSTM2,GSTM5,GSTM3,EPS8L3,CSF1,AHCYL1,STRIP1,ALX3,UBL4B,SLC6A17,KCNC4,RBM15,SLC16A4,LAMTOR5,LAMTOR5,LAMTOR5-AS1,PROK1,KCNA10,KCNA2,KCNA3,CD53,LRIF1,DRAM2,CEPT1,DENND2D,CHI3L2,CHIAP2,CHIA,PIFO,OVGP1,WDR77,WDR77,ATP5PB,ATP5PB,C1orf162,TMIGD3,TMIGD3,ADORA3,RAP1A | 0.083849 | 255.787 | 582 | 363.891 |
| 3 | 3 | 37408124 | 44283963 | C3orf35,ITGA9,ITGA9,ITGA9-AS1,CTDSPL,CTDSPL,MIR26A1,VILL,PLCD1,DLEC1,ACAA1,MYD88,OXSR1,SLC22A13,SLC22A14,XYLB,ACVR2B-AS1,ACVR2B,ACVR2B,EXOG,SCN5A,SCN10A,SCN11A,SCN11A,WDR48,WDR48,GORASP1,GORASP1,TTC21A,TTC21A,TTC21A,MIR6822,CSRNP1,XIRP1,CX3CR1,CCR8,SLC25A38,RPSA,RPSA,SNORA6,RPSA,SNORA62,MOBP,MYRIP,MYRIP,EIF1B-AS1,EIF1B,ENTPD3,ENTPD3,ENTPD3-AS1,RPL14,ZNF619,ZNF620,ZNF621,CTNNB1,ULK4,TRAK1,CCK,LYZL4,VIPR1,VIPR1,VIPR1-AS1,SEC22C,SEC22C,SS18L2,SEC22C,NKTR,NKTR,LOC101928323,ZBTB47,KLHL40,HHATL,CCDC13,CCDC13,CCDC13-AS1,HIGD1A,ACKR2,CYP8B1,ZNF662,KRBOX1,FAM198A,POMGNT2,SNRK,SNRK,SNRK-AS1,ANO10,ANO10,ABHD5,ABHD5,MIR138-1,TOPAZ1 | 0.0811165 | 236.994 | 918 | 567.203 |
| 3 | 19 | 60500 | 1272496 | OR4F17,PLPP2,MIER2,THEG,C2CD4C,SHC2,ODF3L2,MADCAM1,TPGS1,CDC34,GZMM,BSG,HCN2,POLRMT,FGF22,RNF126,FSTL3,PRSS57,PALM,MISP,PTBP1,PTBP1,MIR4745,PLPPR3,PLPPR3,MIR3187,AZU1,PRTN3,ELANE,CFD,MED16,R3HDM4,KISS1R,ARID3A,WDR18,GRIN3B,GRIN3B,TMEM259,TMEM259,CNN2,ABCA7,ARHGAP45,POLR2E,GPX4,SBNO2,STK11,CBARP,ATP5F1D,MIDN,CIRBP-AS1,CIRBP-AS1,CIRBP,CIRBP | 0.0802733 | 249.766 | 477 | 284.097 |
| 3 | 2 | 86406652 | 92267022 | IMMT,IMMT,MIR4779,MRPL35,REEP1,KDM3A,CHMP3,RNF103-CHMP3,RNF103-CHMP3,RNF103,RNF103-CHMP3,RMND5A,RMND5A,CD8A,CD8B,ANAPC1P1,LOC285074,MIR4771-1,MIR4771-2,MIR4435-1,MIR4435-2,RGPD1,KRCC1,SMYD1,SMYD1,MIR4780,FABP1,THNSL2,FOXI3,TEX37,LOC101928371,EIF2AK3,EIF2AK3,RPIA,ANKRD36BP2,MIR4436A | 0.0772576 | 253.658 | 411 | 216.922 |
| 3 | 16 | 10574694 | 11857517 | ATF7IP2,EMP2,TEKT5,NUBP1,NUBP1,TVP23A,TVP23A,CIITA,DEXI,CLEC16A,SOCS1,TNP2,PRM3,PRM2,PRM1,RMI2,LITAF,SNN,TXNDC11,ZC3H7A | 0.0771915 | 224.824 | 182 | 107.9 |

| | | | | | | | | |
|---|---|---|---|---|---|---|---|---|
| 3 | 11 | 55035775 | 61319028 | TRIM48,OR4A16,OR4A15,OR4C15,OR4C16,OR4C11,OR4P4,OR4S2,OR4C6,OR5D13,OR5D14,OR5L1,OR5D18,OR5L2,OR5D16,TRIM51,OR5W2,OR5I1,OR10AG1,OR7E5P,OR5F1,OR5AS1,OR8I2,OR8H2,OR8H3,OR8J3,OR8K5,OR5J2,OR5T2,OR5T3,OR5T1,OR8H1,OR8K3,OR8K1,OR8J1,OR8U8,OR8U1,OR5R1,OR5M9,OR5M3,OR5M8,OR5M11,OR5M10,OR5M1,OR5AP2,OR5AR1,OR9G9,OR9G1,OR9G4,OR5AK2,OR5AK4P,LRRC55,APLNR,TNKS1BP1,SSRP1,P2RX3,PRG3,PRG2,SLC43A3,RTN4RL2,SLC43A1,TIMM10,SMTNL1,UBE2L6,SERPING1,MIR130A,YPEL4,CLP1,ZDHHC5,MED19,TMX2,TMX2-CTNND1,TMX2-CTNND1,SELENOH,TMX2-CTNND1,BTBD18,TMX2-CTNND1,CTNND1,OR9Q1,OR6Q1,OR9Q1,OR9Q1,OR9I1,OR9Q2,OR1S2,OR1S1,OR10Q1,OR10W1,OR5B17,OR5B3,OR5B2,OR5B12,OR5B21,LPXN,ZFP91,ZFP91-CNTF,ZFP91-CNTF,CNTF,GLYAT,GLYATL2,GLYATL1,GLYATL1,LOC283194,FAM111B,FAM111A,DTX4,MPEG1,OR5AN1,OR5A2,OR5A1,OR4D6,OR4D10,OR4D11,OR4D9,OSBP,OSBP,MIR3162,PATL1,OR10V1,OR10V2P,STX3,MRPL16,GIF,TCN1,OOSP1,OOSP2,MS4A3,MS4A2,MS4A6A,MS4A4E,MS4A4A,MS4A6E,MS4A7,MS4A14,MS4A5,MS4A1,MS4A12,MS4A13,MS4A8,MS4A18,MS4A15,MS4A10,CCDC86,PTGDR2,ZP1,PRPF19,TMEM109,TMEM132A,SLC15A3,CD6,CD5,VPS37C,PGA3,PGA4,PGA5,VWCE,DDB1,TKFC,TKFC,CYB561A3,CYB561A3,TMEM138,TMEM216,CPSF7,SDHAF2,PPP1R32,MIR4488,LRRC10B,SYT7 | 0.0742335 | 255.845 | 1169 | 707.042 |
| 3 | 9 | 33568663 | 34493316 | ANKRD18B,ANXA2P2,PTENP1,PRSS3,UBE2R2,UBAP2,UBAP2,SNORD121B,UBAP2,SNORD121A,DCAF12,UBAP1,KIF24,NUDT2,MYORG,C9orf24,FAM219A,DNAI1 | 0.0721085 | 249.198 | 180 | 105.8 |
| 3 | 12 | 1770066 | 4609494 | ADIPOR2,CACNA2D4,CACNA2D4,LRTM2,LINC00940,DCP1B,CACNA1C,CACNA1C,CACNA1C-AS2,CACNA1C,CACNA1C-AS1,ITFG2-AS1,ITFG2-AS1,FKBP4,ITFG2-AS1,ITFG2,ITFG2,ITFG2,NRIP2,ITFG2,FOXM1,FOXM1,RHNO1,TULP3,TEAD4,TSPAN9,LOC100128253,PRMT8,PRMT8,THCAT155,CRACR2A,PARP11,CCND2-AS1,CCND2,CCND2,TIGAR,FGF23,FGF6,C12orf4 | 0.070033 | 234.702 | 369 | 220.511 |
| 3 | 9 | 1.15E+08 | 123785816 | HSDL2,C9orf147,C9orf147,C9orf147,KIAA1958,KIAA1958,INIP,SNX30,SLC46A2,ZNF883,ZFP37,FAM225B,FAM225A,SLC31A2,FKBP15,SLC31A1,CDC26,PRPF4,RNF183,WDR31,BSPRY,HDHD3,ALAD,POLE3,C9orf43,RGS3,ZNF618,AMBP,KIF12,COL27A1,COL27A1,MIR455,ORM1,ORM2,AKNA,WHRN,ATP6V1G1,TMEM268,TEX48,TNFSF15,TNFSF8,TNC,DEC1,PAPPA,PAPPA,PAPPA-AS1,ASTN2,ASTN2,TRIM32,ASTN2,SNORA70C,TLR4,BRINP1,MIR147A,CDK5RAP2,MEGF9,FBXW2,PSMD5,CUTALP,PHF19,TRAF1,C5-OT1,C5,C5 | 0.0696201 | 222.561 | 806 | 490.704 |

| | | | | | | | | |
|---|---|---|---|---|---|---|---|---|
| 3 | 1 | 29391497 | 31792071 | EPB41,TMEM200B,SRSF4,MECR,PTPRU,MATN1,MATN1,MATN1-AS1,LAPTM5,LAPTM5,MIR4420,SDC3,PUM1,PUM1,SNORD103A,SNORD103B,PUM1,SNORD103C,NKAIN1,SNRNP40,ZCCHC17 | 0.0693845 | 225.574 | 184 | 109.526 |
| 3 | 10 | 70948966 | 71914905 | SUPV3L1,LOC101928994,HKDC1,HKDC1,HK1,TACR2,TSPAN15,NEUROG3,FAM241B,COL13A1,H2AFY2,AIFM2,TYSND1,SAR1A | 0.0665582 | 219.254 | 174 | 105.389 |
| 3 | 20 | 55919010 | 58422336 | RAE1,LOC100291105,RBM38,RBM38,CTCFL,PCK1,ZBP1,PMEPA1,PMEPA1,NKILA,MIR4532,C20orf85,ANKRD60,PPP4R1L,RAB22A,VAPB,APCDD1L,LINC01711,STX16,STX16-NPEPL1,STX16-NPEPL1,NPEPL1,MIR296,MIR298,GNAS-AS1,GNAS,GNAS,NELFCD,CTSZ,TUBB1,ATP5F1E,SLMO2-ATP5E,SLMO2-ATP5E,PRELID3B,ZNF831,EDN3,PHACTR3 | 0.0647943 | 236.057 | 310 | 184.142 |
| 3 | 16 | 32858994 | 35193080 | SLC6A10P | 0.0647631 | 233.996 | 129 | 61.1607 |
| 3 | 19 | 50661513 | 51305751 | IZUMO2,MYH14,KCNC3,NAPSB,NAPSA,NR1H2,POLD1,SPIB,MYBPC2,FAM71E1,FAM71E1,EMC10,EMC10,JOSD2,ASPDH,LRRC4B,SYT3,C19orf81,SHANK1,CLEC11A,GPR32,ACP4,C19orf48,C19orf48,SNORD88B,C19orf48,SNORD88A,C19orf48,SNORD88C | 0.0540683 | 243.822 | 266 | 163.172 |
| 3 | 1 | 2.35E+08 | 247014562 | COA6,TARBP1,IRF2BP2,TOMM20,TOMM20,SNORA14B,RBM34,ARID4B,ARID4B,MIR4753,GGPS1,TBCE,TBCE,B3GALNT2,B3GALNT2,MIR5096,GNG4,MIR5096,LYST,LYST,LYST,MIR1537,NID1,GPR137B,ERO1B,EDARADD,LGALS8,LGALS8-AS1,LGALS8,LGALS8,HEATR1,HEATR1,ACTN2,MTR,RYR2,RYR2,MIR4428,LOC100130331,ZP4,CHRM3,FMN2,MIR1273E,MIR1273E,GREM2,RGS7,RGS7,MIR3123,FH,KMO,KMO,OPN3,OPN3,OPN3,CHML,WDR64,EXO1,MAP1LC3C,PLD5,CEP170,SDCCAG8,SDCCAG8,MIR4677,SDCCAG8,AKT3,AKT3,ZBTB18,C1orf100,ADSS,CATSPERE,DESI2,COX20,HNRNPU,LOC101928068,EFCAB2,EFCAB2,KIF26B,SMYD3,TFB2M,CNST,SCCPDH,AHCTF1 | 0.0463984 | 258.045 | 1124 | 671.944 |
| 3 | 17 | 67084242 | 67309407 | ABCA6,ABCA6,MIR4524B,MIR4524A,ABCA10,ABCA5 | 0.0455223 | 298.347 | 111 | 63.628 |
| 3 | 4 | 2241705 | 5977736 | POLN,HAUS3,MXD4,MIR4800,MXD4,ZFYVE28,CFAP99,RNF4,FAM193A,TNIP2,SH3BP2,ADD1,MFSD10,NOP14-AS1,NOP14,NOP14,GRK4,HTT,MSANTD1,RGS12,HGFAC,DOK7,LRPAP1,LINC00955,ADRA2C,OTOP1,TMEM128,LYAR,ZBTB49,NSG1,STX18,MSX1,CYTL1,STK32B,LINC01587,EVC2,EVC,CRMP1,MIR378D1 | 0.0443788 | 239.694 | 558 | 335.497 |

| | | | | | | | | |
|---|---|---|---|---|---|---|---|---|
| 3 | 11 | 1.2E+08 | 134946016 | OAF,POU2F3,TMEM136,ARHGEF12,GRIK4,GRIK4,LOC105369532,GRIK4,LOC101929227,GRIK4,LOC101929208,TBCEL,TECTA,SC5D,SORL1,MIR100HG,MIR125B1,MIR100HG,BLID,MIR100HG,MIRLET7A2,MIR100HG,MIR100,MIR100HG,UBASH3B,CRTAM,JHY,BSX,HSPA8,CLMP,MIR4493,LOC100128242,GRAMD1B,SCN3B,ZNF202,OR6X1,OR6M1,TMEM225,OR8D4,OR4D5,OR6T1,OR10S1,OR10G4,OR10G9,OR10G8,OR10G7,VWA5A,OR10D3,OR8G2P,OR8G1,OR8G1,OR8G5,OR8D1,OR8D2,OR8B2,OR8B3,OR8B4,OR8B8,OR8B12,OR8A1,PANX3,TBRG1,SIAE,SIAE,SPA17,SPA17,NRGN,VSIG2,ESAM,ESAM,LOC101929340,MSANTD2,ROBO3,ROBO4,HEPN1,HEPACAM,HEPACAM,CCDC15,SLC37A2,TMEM218,PKNOX2,FEZ1,FEZ1,LOC403312,LOC403312,EI24,STT3A-AS1,STT3A-AS1,STT3A,CHEK1,CHEK1,ACRV1,ACRV1,PATE1,PATE2,PATE3,PATE4,HYLS1,PUS3,DDX25,CDON,RPUSD4,FAM118B,SRPRA,FOXRED1,TIRAP,DCPS,DCPS,GSEC,ST3GAL4,KIRREL3,KIRREL3,LOC101929427,KIRREL3-AS3,LOC101929473,ETS1,ETS1,LOC101929517,LOC101929538,FLI1,FLI1,SENCR,FLI1,KCNJ1,KCNJ5,C11orf45,KCNJ5,TP53AIP1,ARHGAP32,BARX2,TMEM45B,NFRKB,PRDM10,LINC00167,APLP2,ST14,ZBTB44,LOC646383,ADAMTS8,ADAMTS15,C11orf44,LINC02551,SNX19,NTM,OPCML,SPATA19,MIR4697HG,MIR4697,IGSF9B,JAM3,NCAPD3,VPS26B,THYN1,ACAD8,GLB1L3,GLB1L2,B3GAT1,LOC100507548 | 0.044239 | 230.82 | 1471 | 908.788 |
| 3 | 1 | 1.45E+08 | 146012180 | NBPF20,NBPF19,NBPF10,LIX1L,NBPF20,NBPF19,NBPF10,LIX1L,LIX1L-AS1,NBPF20,NBPF19,NBPF10,LIX1L-AS1,RBM8A,NBPF20,NBPF19,NBPF10,RBM8A,NBPF20,NBPF19,NBPF10,RBM8A,GNRHR2,NBPF20,NBPF19,NBPF10,GNRHR2,NBPF20,NBPF19,NBPF10,PEX11B,NBPF20,NBPF19,NBPF10,ITGA10,NBPF20,NBPF19,NBPF10,ANKRD35,NBPF20,NBPF19,NBPF10,PIAS3,NBPF20,NBPF19,NBPF10,PIAS3,MIR6736,NBPF20,NBPF19,NBPF10,PIAS3,NUDT17,NBPF20,NBPF19,NBPF10,NUDT17,NBPF20,NBPF19,NBPF10,POLR3C,NBPF20,NBPF19,NBPF10,RNF115,NBPF20,NBPF19,NBPF10,CD160,NBPF20,NBPF19,NBPF10,PDZK1,NBPF20,NBPF19,NBPF10,GPR89A,NBPF20,NBPF19,NBPF10,NBPF25P,NBPF20,NBPF19,NBPF10,NBPF25P,PDE4DIPP1 | 0.043673 | 235.562 | 148 | 91.7065 |

| | | | | | | | | |
|---|---|---|---|---|---|---|---|---|
| 3 | 21 | 40652061 | 48119395 | BRWD1,BRWD1-AS2,HMGN1,WRB,WRB-SH3BGR,WRB-SH3BGR,WRB,LCA5L,WRB-SH3BGR,SH3BGR,B3GALT5-AS1,B3GALT5,IGSF5,PCP4,DSCAM,DSCAM,MIR4760,MIR3197,BACE2,BACE2,PLAC4,FAM3B,MX2,MX1,TMPRSS2,LINC00479,RIPK4,RIPK4,MIR6814,PRDM15,C2CD2,ZBTB21,ZNF295-AS1,UMODL1,UMODL1,UMODL1-AS1,ABCG1,TFF3,TFF2,TFF1,TMPRSS3,UBASH3A,RSPH1,SLC37A1,PDE9A,WDR4,NDUFV3,ERVH48-1,MIR5692B,PKNOX1,CBS,CBSL,CBSL,CBS,U2AF1,U2AF1L5,CRYAA,CRYAA2,SIK1B,SIK1,HSF2BP,HSF2BP,H2BFS,RRP1B,PDXK,CSTB,RRP1,AGPAT3,TRAPPC10,PWP2,C21orf33,ICOSLG,DNMT3L,DNMT3L,LOC105372833,AIRE,PFKL,C21orf2,TRPM2,TRPM2,TRPM2-AS,LRRC3,TSPEAR,TSPEAR,TSPEAR-AS1,TSPEAR,TSPEAR-AS2,TSPEAR,KRTAP10-1,TSPEAR,KRTAP10-2,TSPEAR,KRTAP10-3,TSPEAR,KRTAP10-4,TSPEAR,KRTAP10-5,TSPEAR,KRTAP10-6,TSPEAR,KRTAP10-7,TSPEAR,KRTAP10-8,TSPEAR,KRTAP10-9,TSPEAR,KRTAP10-10,TSPEAR,KRTAP10-11,TSPEAR,KRTAP12-4,TSPEAR,KRTAP12-3,TSPEAR,KRTAP12-2,TSPEAR,KRTAP12-1,TSPEAR,KRTAP10-12,UBE2G2,SUMO3,PTTG1IP,ITGB2,LINC01547,FAM207A,ADARB1,POFUT2,COL18A1,COL18A1,COL18A1-AS1,COL18A1,MIR6815,SLC19A1,PCBP3,COL6A1,COL6A2,FTCD,FTCD,FTCD-AS1,SPATC1L,LSS,MCM3AP-AS1,MCM3AP-AS1,MCM3AP,MCM3AP,YBEY,C21orf58,PCNT,DIP2A,S100B,PRMT2 | 0.042795 | 238.93 | 1356 | 814.207 |
| 3 | 9 | 95274304 | 99286090 | CENPP,ECM2,CENPP,ECM2,MIR4670,CENPP,CENPP,IPPK,IPPK,IPPK,LOC100128361,BICD2,ANKRD19P,ZNF484,FGD3,FGD3,LOC101927954,SUSD3,CARD19,NINJ1,WNK2,WNK2,C9orf129,C9orf129,FAM120AOS,FAM120AOS,FAM120A,FAM120A,PHF2,PHF2,MIR548AU,MIR4291,BARX1,PTPDC1,MIRLET7A1,MIRLET7F1,MIRLET7DHG,MIRLET7D,ZNF169,NUTM2F,MFSD14B,PCAT7,FBP2,FBP2,FBP1,C9orf3,C9orf3,MIR2278,C9orf3,LOC101928119,C9orf3,MIR23B,MIR27B,C9orf3,MIR3074,MIR24-1,FANCC,PTCH1,PTCH1,LOC100507346,ERCC6L2,LINC00092,LOC158434,HSD17B3,HSD17B3,HSD17B3-AS1,SLC35D2,ZNF367,HABP4,CDC14B | 0.0383176 | 219.595 | 521 | 306.06 |
| 3 | 20 | 3955501 | 5538708 | SMOX,ADRA1D,PRNP,PRND,PRNT,RASSF2,SLC23A2,TMEM230,PCNA,PCNA,PCNA-AS1,CDS2,PROKR2,LOC643406,LINC00654,GPCPD1 | 0.037332 | 233.661 | 113 | 70.0721 |
| 3 | 2 | 71649970 | 72411383 | ZNF638,DYSF,CYP26B1,EXOC6B | 0.0372538 | 238.25 | 93 | 58.45 |

| | | | | | | | | |
|---|---|---|---|---|---|---|---|---|
| 3 | 10 | 71978404 | 89505778 | PPA1,NPFFR1,LRRC20,EIF4EBP2,NODAL,PALD1,PRF1,ADAMTS14,TBATA,SGPL1,PCBD1,UNC5B,SLC29A3,CDH23,CDH23,CDH23-AS1,CDH23,C10orf105,CDH23,VSIR,PSAP,CHST3,SPOCK2,ASCC1,ASCC1,ANAPC16,ANAPC16,DDIT4,DNAJB12,MICU1,MCU,MCU,MIR4676,OIT3,PLA2G12B,P4HA1,NUDT13,ECD,FAM149B1,DNAJC9,DNAJC9,DNAJC9-AS1,DNAJC9-AS1,DNAJC9-AS1,MRPS16,DNAJC9-AS1,CFAP70,CFAP70,ANXA7,MSS51,PPP3CB,PPP3CB,PPP3CB-AS1,PPP3CB-AS1,USP54,USP54,MYOZ1,SYNPO2L,GLUD1P3,SEC24C,FUT11,CHCHD1,ZSWIM8,ZSWIM8,ZSWIM8-AS1,NDST2,CAMK2G,C10orf55,PLAU,VCL,AP3M1,ADK,ADK,LOC102723439,KAT6B,DUPD1,DUSP13,SAMD8,VDAC2,COMTD1,ZNF503,ZNF503,ZNF503-AS2,ZNF503-AS2,LRMDA,LRMDA,MIR606,KCNMA1,KCNMA1,KCNMA1-AS1,DLG5,DLG5-AS1,POLR3A,RPS24,ZMIZ1-AS1,ZMIZ1,PPIF,ZCCHC24,LOC729815,EIF5AL1,SFTPA2,SFTPA1,BMS1P21,MBL1P,SFTPD,TMEM254-AS1,TMEM254,TMEM254,PLAC9,ANXA11,LOC100130698,MAT1A,DYDC1,DYDC1,DYDC2,DYDC2,FAM213A,TSPAN14,SH2D4B,NRG3,NRG3,NRG3-AS1,GHITM,C10orf99,CDHR1,LRIT2,LRIT1,RGR,CCSER2,GRID1-AS1,GRID1,GRID1,GRID1,MIR346,WAPL,OPN4,LDB3,BMPR1A,MMRN2,MMRN2,SNCG,SNCG,ADIRF,ADIRF,AGAP11,AGAP11,GLUD1,FAM35A,MIR4678,MINPP1,PAPSS2 | 0.0366546 | 231.202 | 1616 | 972.35 |
| 3 | 16 | 31196804 | 31447600 | FUS,PYCARD,PYCARD,PYCARD-AS1,TRIM72,TRIM72,PYDC1,ITGAM,ITGAX,ITGAD,COX6A2,ZNF843 | 0.0363042 | 250.51 | 115 | 70.5439 |
| 3 | 3 | 1.85E+08 | 188924629 | VPS8,C3orf70,EHHADH-AS1,EHHADH,EHHADH,MIR5588,MAP3K13,TMEM41A,LIPH,SENP2,IGF2BP2,IGF2BP2,IGF2BP2-AS1,TRA2B,ETV5,DGKG,CRYGS,TBCCD1,DNAJB11,AHSG,FETUB,HRG,KNG1,EIF4A2,EIF4A2,SNORD2,EIF4A2,MIR1248,SNORA81,EIF4A2,SNORA63,EIF4A2,SNORA4,RFC4,ADIPOQ,ADIPOQ-AS1,ST6GAL1,RPL39L,LOC101929106,RTP1,MASP1,MASP1,LOC101929130,RTP4,SST,RTP2,RTP2,LOC100131635,LOC100131635,LOC100131635,BCL6,BCL6,LPP,FLJ42393,LPP,LPP,MIR28 | 0.0346188 | 212.696 | 394 | 237.491 |

| | | | | | | | | |
|---|---|---|---|---|---|---|---|---|
| 3 | 20 | 29420069 | 55905066 | FRG1BP,FRG1DP,FRG1BP,DEFB115,DEFB116,DEFB118,DEFB119,DEFB121,DEFB123,DEFB124,REM1,HM13,HM13,HM13-AS1,ID1,MIR3193,COX4I2,BCL2L1,BCL2L1,ABALON,TPX2,MYLK2,FOXS1,DUSP15,DUSP15,TTLL9,TTLL9,PDRG1,XKR7,CCM2L,HCK,TM9SF4,TSPY26P,PLAGL2,POFUT1,POFUT1,MIR1825,KIF3B,ASXL1,NOL4L,NOL4L,LOC101929698,NOL4L-DT,C20orf203,COMMD7,DNMT3B,MAPRE1,EFCAB8,SUN5,BPIFB2,BPIFB6,BPIFB3,BPIFB4,BPIFA2,BPIFA3,BPIFA1,BPIFB1,CDK5RAP1,SNTA1,CBFA2T2,NECAB3,NECAB3,C20orf144,NECAB3,ACTL10,E2F1,PXMP4,ZNF341,ZNF341,ZNF341-AS1,CHMP4B,RALY,MIR4755,RALY,EIF2S2,ASIP,AHCY,ITCH,ITCH,MIR644A,DYNLRB1,MAP1LC3A,PIGU,TP53INP2,NCOA6,GGT7,ACSS2,GSS,MYH7B,MYH7B,MIR499A,MIR499B,TRPC4AP,EDEM2,MMP24-AS1-EDEM2,MMP24-AS1-EDEM2,MMP24-AS1-EDEM2,PROCR,MMP24-AS1-EDEM2,MMP24,EIF6,FAM83C,UQCC1,GDF5OS,GDF5OS,GDF5,GDF5,GDF5,MIR1289-1,CEP250,C20orf173,ERGIC3,FER1L4,SPAG4,CPNE1,CPNE1,RBM12,NFS1,NFS1,ROMO1,ROMO1,RBM39,PHF20,SCAND1,CNBD2,NORAD,EPB41L1,EPB41L1,LOC100130373,AAR2,DLGAP4,DLGAP4,DLGAP4-AS1,DLGAP4-AS1,MYL9,TGIF2,TGIF2-C20orf24,TGIF2-C20orf24,C20orf24,SLA2,NDRG3,DSN1,SOGA1,TLDC2,TLDC2,SAMHD1,SAMHD1,RBL1,MROH8,MROH8,RPN2,RPN2,GHRH,MANBAL,SRC,BLCAP,BLCAP,NNAT,CTNNBL1,VSTM2L,TTI1,RPRD1B,TGM2,KIAA1755,KIAA1755,LOC149684,BPI,LBP,SNHG17,SNORA71B,SNHG17,SNORA71A,SNHG17,SNORA71C,SNHG17,SNORA71D,SNHG11,SNORA71E,SNHG11,SNORA60,RALGAPB,RALGAPB, | 0.0325518 | 231.315 | 3533 | 2179.7 |

| | | | | | | | | |
|---|---|---|---|---|---|---|---|---|
| 3 | 14 | 69908761 | 89341485 | SLC39A9,PLEKHD1,CCDC177,SUSD6,LOC100289511,SRSF5,SRSF5,SLC10A1,SMOC1,SLC8A3,LOC646548,ADAM21P1,COX16,SYNJ2BP-COX16,SYNJ2BP-COX16,SYNJ2BP,ADAM21,ADAM20P1,ADAM20,MED6,TTC9,MAP3K9,PCNX1,SIPA1L1,RGS6,DPF3,DCAF4,ZFYVE1,RBM25,PSEN1,PAPLN,PAPLN,LOC101928123,NUMB,HEATR4,HEATR4,RIOX1,HEATR4,ACOT1,ACOT2,ACOT4,ACOT6,DNAL1,PNMA1,ELMSAN1,ELMSAN1,MIR4505,PTGR2,ZNF410,FAM161B,FAM161B,COQ6,COQ6,COQ6,ENTPD5,ENTPD5,BBOF1,BBOF1,ALDH6A1,ALDH6A1,LIN52,VSX2,ABCD4,VRTN,SYNDIG1L,NPC2,MIR4709,NPC2,ISCA2,LTBP2,AREL1,AREL1,FCF1,FCF1,YLPM1,PROX2,DLST,RPS6KL1,PGF,EIF2B2,MLH3,ACYP1,ZC2HC1C,NEK9,TMED10,FOS,JDP2,BATF,LOC102724153,FLVCR2,FLVCR2,ERG28,TTLL5,TGFB3,IFT43,GPATCH2L,ESRRB,VASH1,ANGEL1,LRRC74A,IRF2BPL,CIPC,ZDHHC22,TMEM63C,NGB,MIR1260A,NGB,POMT2,GSTZ1,TMED8,SAMD15,NOXRED1,VIPAS39,AHSA1,ISM2,SPTLC2,ALKBH1,ALKBH1,SLIRP,SLIRP,SLIRP,SNW1,SNW1,SNW1,C14orf178,C14orf178,ADCK1,NRXN3,DIO2,DIO2,DIO2-AS1,CEP128,TSHR,GTF2A1,GTF2A1,SNORA79,STON2,SEL1L,LINC02301,FLRT2,LINC01148,GALC,GPR65,KCNK10,SPATA7,PTPN21,ZC3H14,EML5,TTC8 | 0.0301916 | 230.57 | 1788 | 1091.05 |
| 3 | 12 | 10765344 | 15087986 | MAGOHB,STYK1,YBX3,TAS2R7,TAS2R8,TAS2R9,TAS2R10,PRR4,PRH1-PRR4,PRH1-PRR4,PRH1,PRH1-PRR4,PRH1,TAS2R13,PRH1-PRR4,PRH1,PRH2,PRH1-PRR4,PRH1,PRH1-TAS2R14,TAS2R14,PRH1-PRR4,PRH1,PRH1-TAS2R14,PRH1-PRR4,PRH1,PRH1-TAS2R14,TAS2R50,PRH1-PRR4,PRH1,PRH1-TAS2R14,TAS2R20,PRH1-PRR4,PRH1,PRH1-TAS2R14,TAS2R19,PRH1-PRR4,PRH1,PRH1-TAS2R14,TAS2R31,PRH1-PRR4,PRH1,PRH1-TAS2R14,TAS2R46,PRH1-PRR4,PRH1,PRH1-TAS2R14,TAS2R43,PRH1-PRR4,PRH1,PRH1-TAS2R14,TAS2R30,TAS2R42,PRB3,PRB4,PRB1,PRB2,ETV6,BCL2L14,MIR1244-1,MIR1244-2,MIR1244-3,MIR1244-4,LRP6,MANSC1,LOH12CR2,LOH12CR2,BORCS5,BORCS5,DUSP16,CREBL2,GPR19,CDKN1B,APOLD1,APOLD1,MIR613,DDX47,RPL13AP20,GPRC5A,MIR614,GPRC5D-AS1,GPRC5D,GPRC5D-AS1,HEBP1,GPRC5D-AS1,HTR7P1,FAM234B,GSG1,EMP1,LINC01559,GRIN2B,ATF7IP,PLBD1,PLBD1-AS1,GUCY2C,GUCY2C,HIST4H4,H2AFJ,WBP11,C12orf60,SMCO3,C12orf60,ART4,MGP,ERP27 | 0.0292711 | 242.809 | 494 | 289.538 |

| | | | | | | | | |
|---|---|---|---|---|---|---|---|---|
| 3 | 12 | 7169137 | 9913546 | C1S,C1R,C1RL,C1RL,C1RL-AS1,C1RL-AS1,RBP5,CLSTN3,PEX5,ACSM4,CD163L1,CD163,APOBEC1,GDF3,DPPA3,CLEC4C,NANOGNB,NANOG,SLC2A14,SLC2A3,FOXJ2,C3AR1,NECAP1,CLEC4A,CLEC4A,POU5F1P3,FAM90A1,LINC00937,CLEC6A,CLEC4D,CLEC4E,AICDA,MFAP5,RIMKLB,A2ML1,PHC1,PHC1,M6PR,M6PR,KLRG1,LINC00612,A2M-AS1,A2M,A2M,PZP,LOC642846,LINC02367,LOC728715,LOC728715,DDX12P,KLRB1,CLEC2D,CLECL1,CD69 | 0.0231356 | 242.966 | 589 | 342.675 |
| 3 | 14 | 64561806 | 69707690 | SYNE2,MIR548H1,SYNE2,ESR2,TEX21P,MTHFD1,MTHFD1,ZBTB25,ZBTB25,AKAP5,ZBTB25,ZBTB1,LOC102723809,HSPA2,PPP1R36,PLEKHG3,SPTB,SPTB,MIR7855,CHURC1,CHURC1-FNTB,CHURC1-FNTB,GPX2,CHURC1-FNTB,CHURC1-FNTB,RAB15,CHURC1-FNTB,FNTB,CHURC1-FNTB,FNTB,MAX,CHURC1-FNTB,FNTB,MAX,MIR4706,MAX,MAX,LOC100506321,MIR4708,FUT8,FUT8-AS1,FUT8,MIR625,FUT8,CCDC196,GPHN,FAM71D,MPP5,ATP6V1D,EIF2S1,PLEK2,MIR5694,TMEM229B,PLEKHH1,PIGH,ARG2,ARG2,VTI1B,VTI1B,RDH11,RDH12,ZFYVE26,RAD51B,ZFP36L1,ACTN1,DCAF5,EXD2 | 0.0223905 | 226.466 | 669 | 405.829 |
| 3 | 10 | 35427339 | 37422982 | CREM,CCNY,GJD4,FZD8,FZD8,MIR4683,ANKRD30A | 0.0223464 | 207.913 | 71 | 41.9306 |
| 3 | 9 | 1.24E+08 | 128728260 | CNTRL,RAB14,GSN,GSN-AS1,GSN,STOM,GGTA1P,DAB2IP,TTLL11,MIR4478,NDUFA8,MORN5,LHX6,RBM18,MRRF,PTGS1,OR1J1,OR1J2,OR1J4,OR1N1,OR1N2,OR1L8,OR1Q1,OR1B1,OR1L1,OR1L3,OR1L4,OR1L6,OR5C1,OR1K1,PDCL,RC3H2,RC3H2,SNORD90,ZBTB6,ZBTB26,RABGAP1,RABGAP1,GPR21,MIR600HG,MIR600HG,MIR600,STRBP,CRB2,DENND1A,DENND1A,MIR601,LOC100505588,LHX2,NEK6,PSMB7,LOC100129034,PSMB7,NR5A1,NR6A1,NR6A1,MIR181A2HG,MIR181A2,NR6A1,MIR181A2HG,MIR181B2,OLFML2A,WDR38,RPL35,ARPC5L,GOLGA1,SCAI,PPP6C,RABEPK,HSPA5,GAPVD1,MAPKAP1,PBX3 | 0.0207948 | 226.995 | 717 | 432.454 |
| 3 | 2 | 70131926 | 71577454 | MXD1,ASPRV1,PCBP1-AS1,PCBP1,C2orf42,TIA1,PCYOX1,SNRPG,FAM136A,TGFA,ADD2,FIGLA,CLEC4F,CD207,VAX2,ATP6V1B1,ATP6V1B1,ATP6V1B1-AS1,ANKRD53,TEX261,NAGK,MCEE,MPHOSPH10,PAIP2B,ZNF638 | 0.0196802 | 228.754 | 218 | 131.741 |
| 3 | 3 | 52574400 | 56591389 | SMIM4,PBRM1,GNL3,GNL3,SNORD136,SNORD19,GNL3,SNORD19B,GNL3,SNORD69,GLT8D1,GLT8D1,SPCS1,SPCS1,NEK4,ITIH1,ITIH3,ITIH4,ITIH4,ITIH4-AS1,MUSTN1,TMEM110-MUSTN1,TMEM110-MUSTN1,TMEM110-MUSTN1,STIMATE,SFMBT1,RFT1,PRKCD,TKT,DCP1A,CACNA1D,CHDH,IL17RB,ACTR8,SELENOK,CACNA2D3,CACNA2D3,CACNA2D3-AS1,CACNA2D3,LRTM1,WNT5A,ERC2,ERC2,MIR3938,CCDC66 | 0.0168759 | 232.272 | 479 | 293.014 |

| | | | | | | | | |
|---|---|---|---|---|---|---|---|---|
| 3 | 8 | 27691217 | 30601907 | SCARA5,SCARA5,MIR4287,NUGGC,ELP3,PNOC,ZNF395,FBXO16,FZD3,FZD3,MIR4288,EXTL3,INTS9,HMBOX1,KIF13B,DUSP4,MIR3148,SARAF,LEPROTL1,LEPROTL1,MBOAT4,MBOAT4,DCTN6,RBPMS-AS1,RBPMS,RBPMS,GTF2E2,GTF2E2,SMIM18,GSR,UBXN8 | 0.0156953 | 221.726 | 314 | 185.758 |
| 3 | 3 | 1.83E+08 | 183823816 | DCUN1D1,MCCC1,LAMP3,MCF2L2,MCF2L2,B3GNT5,KLHL6,KLHL24,YEATS2,YEATS2,YEATS2-AS1,MAP6D1,PARL,ABCC5,ABCC5,ABCC5-AS1,HTR3D,HTR3C,HTR3E-AS1,HTR3E,HTR3E | 0.00291313 | 228.48 | 231 | 136.111 |
| 3 | 9 | 99525320 | 102677670 | ZNF510,ZNF782,NUTM2G,MFSD14C,CTSV,ANKRD18CP,LOC100499484,LOC100499484-C9ORF174,LOC100499484-C9ORF174,LOC100499484-C9ORF174,CCDC180,LOC100499484-C9ORF174,CCDC180,MIR1302-8,TDRD7,TMOD1,TMOD1,TSTD2,TSTD2,NCBP1,XPA,FOXE1,TRMO,HEMGN,ANP32B,NANS,TRIM14,CORO2A,TBC1D2,TBC1D2,MIR6854,GABBR2,ANKS6,GALNT12,COL15A1,TGFBR1,ALG2,SEC61B,NR4A3,STX17 | -0.00015744 | 222.052 | 429 | 256.503 |
| 3 | 11 | 36616228 | 43964882 | C11orf74,LRRC4C,LOC100507205,API5,TTC17,MIR670,MIR129-2,HSD17B12,ALKBH3,ALKBH3,SEC14L1P1,ALKBH3,ALKBH3-AS1,C11orf96 | -0.00257291 | 187.356 | 172 | 101.51 |
| 3 | 6 | 34725697 | 38690985 | SNRPC,UHRF1BP1,TAF11,ANKS1A,TCP11,SCUBE3,ZNF76,DEF6,PPARD,FANCE,RPL10A,RPL10A,MIR7111,TEAD3,TULP1,FKBP5,FKBP5,MIR5690,ARMC12,CLPSL2,CLPSL1,CLPS,LHFPL5,SRPK1,SLC26A8,MAPK14,MAPK13,BRPF3,PNPLA1,C6orf222,ETV7,PXT1,KCTD20,STK38,SRSF3,MIR3925,CDKN1A,DINOL,CDKN1A,RAB44,CPNE5,PPIL1,PPIL1,C6orf89,C6orf89,PI16,MTCH1,FGD2,PIM1,TMEM217,TMEM217,TBC1D22B,TBC1D22B,RNF8,CMTR1,CCDC167,LINC02520,MIR4462,MDGA1,ZFAND3,BTBD9,GLO1,DNAH8 | -0.0077167 | 223.572 | 705 | 425.411 |

| | | | | | | | | |
|---|---|---|---|---|---|---|---|---|
| 3 | 5 | 1.34E+08 | 170837509 | DDX46,C5orf24,TXNDC15,PCBD2,CATSPER3,PITX1,PITX1,C5orf66,C5orf66,C5orf66-AS2,C5orf66,C5orf66,H2AFY,H2AFY,DCANP1,TIFAB,NEUROG1,CXCL14,SLC25A48,LINC01959,SLC25A48,MIR5692C1,SLC25A48,IL9,FBXL21,LECT2,TGFBI,VTRNA2-1,SMAD5-AS1,SMAD5,SMAD5,SMIM32,TRPC7,TRPC7,TRPC7-AS2,SPOCK1,KLHL3,KLHL3,MIR874,HNRNPA0,NPY6R,MYOT,PKD2L2,PKD2L2,FAM13B,FAM13B,LOC100130172,WNT8A,NME5,BRD8,KIF20A,CDC23,GFRA3,CDC25C,CDC25C,FAM53C,FAM53C,KDM3B,REEP2,EGR1,ETF1,HSPA9,HSPA9,SNORD63,CTNNA1,CTNNA1,LRRTM2,SIL1,SNHG4,MATR3,SNORA74D,SNHG4,MATR3,SNORA74A,MATR3,PAIP2,PAIP2,SLC23A1,SLC23A1,MZB1,PROB1,SPATA24,DNAJC18,ECSCR,TMEM173,UBE2D2,CXXC5,PSD2,NRG2,PURA,IGIP,CYSTM1,PFDN1,HBEGF,SLC4A9,ANKHD1,ANKHD1-EIF4EBP3,ANKHD1-EIF4EBP3,ANKHD1-EIF4EBP3,EIF4EBP3,SRA1,APBB3,APBB3,MIR6831,SLC35A4,CD14,TMCO6,NDUFA2,IK,MIR3655,IK,WDR55,DND1,HARS,HARS,HARS2,HARS2,ZMAT2,PCDHA1,PCDHA1,PCDHA2,PCDHA1,PCDHA2,PCDHA3,PCDHA1,PCDHA2,PCDHA3,PCDHA4,PCDHA1,PCDHA2,PCDHA3,PCDHA4,PCDHA5,PCDHA1,PCDHA2,PCDHA3,PCDHA4,PCDHA5,PCDHA6,PCDHA1,PCDHA2,PCDHA3,PCDHA4,PCDHA5,PCDHA6,PCDHA7,PCDHA1,PCDHA2,PCDHA3,PCDHA4,PCDHA5,PCDHA6,PCDHA7,PCDHA8,PCDHA1,PCDHA2,PCDHA3,PCDHA4,PCDHA5,PCDHA6,PCDHA7,PCDHA8,PCDHA9,PCDHA1,PCDHA2,PCDHA3,PCDHA4,PCDHA5,PCDHA6,PCDHA7,PCDHA8,PCDHA9,PCDHA10,PCDHA1,PCDHA2,PCDHA3,PCDHA4,PCDHA5,PCDHA6,PCDHA7,PCDHA8,PCDHA9,PCDHA10,PCDHA11,PCDHA1,PCDHA2,PCDHA3,PCDHA4,PCDHA5,PCDHA6,PCDHA7,PC | -0.0137869 | 232.672 | 3883 | 2394.49 |
| 3 | 18 | 18690689 | 19112633 | ROCK1,GREB1L,ESCO1 | -0.0160364 | 242.809 | 41 | 26.7125 |
| 3 | 21 | 39471368 | 40574470 | DSCR4,DSCR8,DSCR10,KCNJ15,ERG,ETS2,LOC400867,PSMG1,BRWD1 | -0.0208594 | 214.048 | 78 | 47.4321 |
| 3 | 11 | 62782076 | 63532661 | SLC22A8,SLC22A24,SLC22A25,SLC22A10,SLC22A9,HRASLS5,LGALS12,RARRES3,HRASLS2,PLA2G16,ATL3,RTN3,C11orf95 | -0.0261101 | 218.483 | 151 | 88.8384 |
| 3 | 9 | 1.03E+08 | 104500268 | ERP44,INVS,INVS,TEX10,TEX10,MSANTD3,MSANTD3-TMEFF1,MSANTD3-TMEFF1,TMEFF1,CAVIN4,PLPPR1,BAAT,MRPL50,ZNF189,ALDOB,TMEM246-AS1,TMEM246,RNF20,GRIN3A,GRIN3A,PPP3R2 | -0.0279503 | 217.8 | 169 | 103.007 |

| | | | | | | | | |
|---|---|---|---|---|---|---|---|---|
| 3 | 17 | 25268559 | 59112171 | MIR4522,WSB1,TBC1D3P5,KSR1,LGALS9,NOS2,LYRM9,NLK,PPY2P,KRT18P55,TMEM97,TMEM97,IFT20,IFT20,TNFAIP1,POLDIP2,TMEM199,TMEM199,MIR4723,SEBOX,VTN,SARM1,SARM1,SLC46A1,SLC46A1,SLC13A2,FOXN1,UNC119,PIGS,ALDOC,SPAG5,SPAG5-AS1,SGK494,SPAG5-AS1,KIAA0100,KIAA0100,SDF2,SUPT6H,PROCA1,RAB34,RPL23A,RPL23A,SNORD42B,RPL23A,SNORD4A,RPL23A,SNORD42A,RPL23A,SNORD4B,TLCD1,NEK8,TRAF4,FAM222B,ERAL1,MIR451A,MIR451B,MIR144,MIR4732,FLOT2,DHRS13,PHF12,PHF12,LOC101927018,SEZ6,PIPOX,MYO18A,TIAF1,MYO18A,CRYBA1,NUFIP2,MIR4523,TAOK1,ABHD15,TP53I13,GIT1,ANKRD13B,CORO6,SSH2,SSH2,EFCAB5,EFCAB5,NSRP1,MIR423,MIR3184,NSRP1,SLC6A4,BLMH,TMIGD1,CPD,GOSR1,TBC1D29,LOC107133515,SH3GL1P2,LOC107133515,SUZ12P1,SUZ12P1,CRLF3,CRLF3,ATAD5,TEFM,ADAP2,RNF135,LOC646030,MIR4733,NF1,NF1,OMG,NF1,EVI2B,NF1,EVI2A,RAB11FIP4,RAB11FIP4,MIR4724,MIR193A,MIR4725,MIR365B,COPRS,UTP6,SUZ12,LRRC37B,LRRC37B,SH3GL1P1,LOC105371730,LOC105371730,RHOT1,RHOT1,RHBDL3,C17orf75,MIR632,ZNF207,ZNF207,PSMD11,CDK5R1,MYO1D,TMEM98,SPACA3,ASIC2,CCL2,CCL7,CCL11,CCL8,CCL13,CCL1,C17orf102,TMEM132E,CCT6B,ZNF830,LIG3,RFFL,RAD51L3-RFFL,RAD51L3-RFFL,RAD51D,RAD51L3-RFFL,FNDC8,NLE1,UNC45B,SLC35G3,SLFN5,SLFN11,SLFN12,SLFN13,SLFN12L,SLFN14,SLFN14,LOC107985033,LINC02001,SNORD7,PEX12,AP2B1,RASL10B,GAS2L2,MMP28,C17orf50,MMP28,TAF15,HEATR9,CCL5,RDM1,LYZL6,CCL16,CCL14,CCL15-CCL14,CCL15-CCL14,CCL15,CCL23,CCL18,CCL3,CCL4,CCL3L3,CCL3L1,CCL4L1,CCL4L2,ZNHIT3,ZNHIT3,MYO19,MYO19,PIGW, | -0.0285021 | 236.74 | 6715 | 4051.79 |
| 3 | 18 | 9134687 | 9235760 | ANKRD12 | -0.0299195 | 182.173 | 11 | 5.9875 |

| | | | | | | | | |
|---|---|---|---|---|---|---|---|---|
| 3 | 16 | 11992404 | 28332251 | GSPT1,NPIPB2,TNFRSF17,SNX29,CPPED1,CPPED1,MIR4718,SHISA9,ERCC4,MKL2,MIR193BHG,MIR193B,MIR193BHG,MIR365A,PARN,BFAR,PLA2G10,ABCC6P2,NOMO1,PDXDC1,PDXDC1,NTAN1,PDXDC1,RRN3,MPV17L,C16orf45,MARF1,MARF1,MIR6506,NDE1,MIR484,NDE1,NDE1,MYH11,MYH11,FOPNL,ABCC1,ABCC6,NOMO3,PKD1P4-NPIPA8,XYLT1,XYLT1,LOC102723692,PKD1P5-LOC105376752,NOMO2,ABCC6P1,RPS15A,ARL6IP1,SMG1,TMC7,LOC102723385,COQ7,COQ7,ITPRIPL2,SYT17,CLEC19A,TMC5,GDE1,CCP110,C16orf62,KNOP1,IQCK,GPRC5B,GPR139,GP2,UMOD,PDILT,ACSM5,ACSM2A,ACSM2B,ACSM1,THUMPD1,ACSM3,ACSM3,ERI2,ERI2,ERI2,REXO5,REXO5,DCUN1D3,LYRM1,DNAH3,DNAH3,TMEM159,TMEM159,ZP2,ANKS4B,CRYM,CRYM-AS1,SNX29P1,MIR3680-2,MIR3680-1,SLC7A5P2,LOC101927814,METTL9,METTL9,METTL9,IGSF6,OTOA,RRN3P1,UQCRC2,PDZD9,C16orf52,VWA3A,EEF2K,POLR3E,CDR2,RRN3P3,LOC653786,HS3ST2,USP31,SCNN1G,SCNN1B,COG7,GGA2,EARS2,UBFD1,NDUFAB1,PALB2,DCTN5,PLK1,ERN2,CHP2,PRKCB,CACNG3,RBBP6,TNRC6A,LINC01567,TNRC6A,SLC5A11,ARHGAP17,LOC554206,LCMT1,AQP8,ZKSCAN2,HS3ST4,HS3ST4,MIR548W,C16orf82,KDM8,NSMCE1,FLJ21408,IL4R,IL21R,IL21R,IL21R-AS1,GTF3C1,KIAA0556,KIAA0556,LOC100128079,GSG1L,XPO6,SBK1 | -0.0327711 | 227.034 | 1954 | 1172.86 |
| 3 | 9 | 35957194 | 47317179 | OR2S2,RECK,GLIPR2,CCIN,CLTA,GNE,RNF38,MELK,MIR4475,PAX5,PAX5,MIR4540,PAX5,MIR4476,ZCCHC7,GRHPR,ZBTB5,POLR1E,FBXO10,TOMM5,FRMPD1,TRMT10B,EXOSC3,DCAF10,SLC25A51,SHB,ALDH1B1,IGFBPL1,ANKRD18A,FAM201A,CNTNAP3,ZNF658B,ZNF658,CNTNAP3B,CNTNAP3P2 | -0.0340214 | 197.053 | 403 | 235.094 |
| 3 | 10 | 70706177 | 70884084 | DDX50,DDX21,KIF1BP,SRGN,VPS26A | -0.0359659 | 228.491 | 36 | 21.3686 |
| 3 | 9 | 65468179 | 72915143 | LINC01410,PTGER4P2-CDK2AP2P2,LOC403323,LINC00537,MIR4477A,MIR4477B,FRG1JP,MIR1299,PGM5P2,CBWD6,CBWD5,PGM5,TMEM252,PIP5K1B,FAM122A,PIP5K1B,PIP5K1B,LOC101927069,PRKACG,FXN,TJP2,FAM189A2,APBA1,PTAR1,C9orf135,MAMDC2,MAMDC2,MAMDC2-AS1,MAMDC2,SMC5-AS1,SMC5 | -0.0379452 | 207.592 | 228 | 128.998 |
| 3 | 2 | 27917984 | 31754594 | LINC01460,MRPL33,RBKS,RBKS,BABAM2,BRE-AS1,BABAM2,BABAM2,MIR4263,BABAM2,LOC100505716,FLJ31356,FOSL2,FOSL2,PLB1,PPP1CB,SPDYA,SPDYA,TRMT61B,TRMT61B,WDR43,WDR43,SNORD92,WDR43,SNORD53,TOGARAM2,C2orf71,CLIP4,ALK,YPEL5,LBH,LCLAT1,CAPN13,GALNT14,CAPN14,EHD3,XDH,SRD5A2 | -0.0388103 | 220.328 | 435 | 266.675 |

| | | | | | | | | |
|---|---|---|---|---|---|---|---|---|
| 3 | 15 | 20044632 | 41797800 | CHEK2P2,HERC2P3,NBEAP1,POTEB,POTEB2,POTEB3,MIR5701-1,MIR5701-2,MIR5701-3,FAM30C,LOC101927079,LINC02203,LOC101927079,LINC02203,OR4M2,LOC101927079,OR4N4,OR4N3P,IGHV1OR15-1,LOC102724760,IGHV1OR15-3,LOC642131,REREP3,TUBGCP5,CYFIP1,NIPA2,NIPA1,WHAMMP3,GOLGA8IP,HERC2P2,GOLGA6L2,MIR4508,MKRN3,MAGEL2,NDN,NPAP1,SNRPN,SNHG14,SNURF,SNHG14,SNHG14,SNORD107,PWARSN,SNHG14,PWAR5,SNORD64,SNHG14,PWAR5,SNORD108,SNHG14,SNORD109B,SNORD109A,SNHG14,SNORD116-1,SNHG14,SNORD116-2,SNHG14,SNORD116-3,SNORD116-9,SNHG14,SNORD116-4,SNHG14,SNORD116-5,SNORD116-7,SNHG14,SNORD116-2,SNORD116-6,SNHG14,SNORD116-8,SNHG14,SNORD116-10,SNHG14,SNORD116-11,SNHG14,SNORD116-12,SNHG14,SNORD116-13,SNHG14,SNORD116-14,SNHG14,SNORD116-19,SNORD116-17,SNHG14,SNORD116-22,SNHG14,SNORD116-23,SNHG14,SNORD116-24,SNHG14,SNORD116-25,SNHG14,SNORD116-26,SNHG14,SNORD116-27,SNHG14,SNORD116-28,SNHG14,SNORD116-29,SNHG14,SNORD115-1,SNHG14,SNORD115-5,SNORD115-9,SNORD115-10,SNORD115-12,SNHG14,SNORD115-6,SNHG14,SNORD115-7,SNHG14,SNORD115-8,SNHG14,SNORD115-9,SNORD115-10,SNORD115-12,SNORD115-5,SNHG14,SNORD115-10,SNHG14,SNORD115-13,SNHG14,SNORD115-14,SNHG14,SNORD115-16,SNHG14,SNORD115-17,SNORD115-18,SNORD115-19,SNHG14,SNORD115-20,SNORD115-15,SNORD115-21,SNHG14,SNORD115-15,SNORD115- | -0.039354 | 239.12 | 2088 | 1218.69 |
| 3 | 8 | 39695533 | 42698364 | ADAM2,IDO1,IDO2,TCIM,ZMAT4,SFRP1,SFRP1,MIR548AO,GOLGA7,GINS4,GINS4,LOC102723729,GPAT4,NKX6-3,ANK1,ANK1,MIR486-1,MIR486-2,KAT6A,AP3M2,PLAT,IKBKB,POLB,DKK4,VDAC3,SLC20A2,SMIM19,CHRNB3,CHRNA6,THAP1 | -0.041802 | 212.364 | 335 | 204.855 |
| 3 | 8 | 1.21E+08 | 121535620 | MTBP | -0.0422575 | 310.591 | 22 | 13.4846 |
| 3 | 3 | 44449680 | 45875922 | ZNF445,ZNF852,ZKSCAN7,ZNF660,ZNF660-ZNF197,ZNF660-ZNF197,ZNF197,ZNF35,ZNF502,ZNF501,KIAA1143,KIF15,MIR564,TMEM42,TMEM42,TGM4,ZDHHC3,EXOSC7,CLEC3B,CDCP1,TMEM158,LARS2,LARS2,LARS2-AS1,LIMD1,SACM1L,SLC6A20,LZTFL1 | -0.0429524 | 209.501 | 281 | 172.145 |
| 3 | 20 | 25405742 | 26256532 | GINS1,NINL,NANP,ZNF337-AS1,ZNF337,ZNF337,FAM182B,LOC101926935,FAM182A,NCOR1P1,MIR663AHG,MIR663A | -0.0514122 | 246.833 | 76 | 42.1717 |
| 3 | 11 | 47493794 | 48998678 | CELF1,PTPMT1,PTPMT1,KBTBD4,KBTBD4,KBTBD4,NDUFS3,NDUFS3,FAM180B,C1QTNF4,MTCH2,AGBL2,FNBP4,NUP160,PTPRJ,PTPRJ,MIR3161,OR4B1,OR4X2,OR4X1,OR4S1,OR4C3,OR4C45,OR4C5,OR4A47 | -0.052246 | 249.994 | 221 | 136.04 |
| 3 | 1 | 2.25E+08 | 225700510 | DNAH14,LBR,ENAH | -0.0553462 | 260.124 | 56 | 33.5815 |
| 3 | 9 | 93627223 | 95048136 | SYK,AUH,NFIL3,MIR3910-1,MIR3910-2,ROR2,SPTLC1,LINC00475,PRSS47,IARS | -0.0576622 | 220.784 | 124 | 75.2513 |

| | | | | | | | | |
|---|---|---|---|---|---|---|---|---|
| 3 | 18 | 6824723 | 9104348 | ARHGAP28,LINC00668,LAMA1,LAMA1,LOC101927188,LRRC30,PTPRM,RAB12,GACAT2,MTCL1,NDUFV2 | -0.0671706 | 228.218 | 209 | 126.628 |
| 3 | 1 | 1.17E+08 | 121484934 | ATP1A1-AS1,CD58,IGSF3,MIR320B1,C1orf137,CD2,PTGFRN,CD101,CD101,LOC101929099,TTF2,TTF2,MIR942,TRIM45,VTCN1,MAN1A2,FAM46C,GDAP2,WDR3,WDR3,SPAG17,SPAG17,TBX15,WARS2,WARS2,LOC101929147,HAO2,HSD3B2,HSD3B1,ZNF697,PHGDH,HMGCS2,REG4,NBPF7,ADAM30,NOTCH2,FCGR1B,SRGAP2-AS1,SRGAP2D,SRGAP2B,SRGAP2C,EMBP1 | -0.0702252 | 230.08 | 467 | 281.021 |
| 3 | 7 | 1.02E+08 | 102664939 | RASA4DP,FAM185A,FAM185A,FBXL13,FBXL13,FBXL13,LRRC17 | -0.0706928 | 214.13 | 45 | 25.1207 |
| 3 | 3 | 49999992 | 50154746 | RBM6,RBM5,RBM5,RBM5-AS1,RBM5,SEMA3F-AS1 | -0.0726399 | 206.681 | 56 | 34.014 |
| 3 | 8 | 6914630 | 8866679 | DEFB4B,DEFB103B,DEFB103A,SPAG11B,DEFB104A,DEFB104B,DEFB106B,DEFB106A,DEFB105B,DEFB105A,DEFB107A,DEFB107B,PRR23D1,PRR23D2,PRR23D2,PRR23D1,SPAG11B,SPAG11A,SPAG11A,DEFB4A,PRAG1,CLDN23,MFHAS1,ERI1 | -0.0741097 | 236.268 | 121 | 68.1767 |
| 3 | 8 | 31007828 | 38965410 | WRN,NRG1,FUT10,MAK16,MAK16,TTI2,TTI2,RNF122,DUSP26,UNC5D,UNC5D,LOC101929550,KCNU1,LINC01605,ZNF703,LOC102723701,ERLIN2,ERLIN2,LOC728024,PLPBP,ADGRA2,BRF2,RAB11FIP1,GOT1L1,ADRB3,EIF4EBP1,ASH2L,STAR,LSM1,BAG4,DDHD2,PLPP5,NSD3,LETM2,FGFR1,C8orf86,RNF5P1,TACC1,PLEKHA2,HTRA4,TM2D2,ADAM9,ADAM32 | -0.0805148 | 209.929 | 546 | 331.402 |
| 3 | 7 | 35058793 | 35870555 | DPY19L1,DPY19L2P1,TBX20,LOC401324,HERPUD2,SEPT7 | -0.0844296 | 186.13 | 47 | 26.0339 |
| 3 | 7 | 26193268 | 33045014 | 04,LOC441204,KIAA0087,C7orf71,SKAP2,HOXA1,HOXA2,HOXA3,HOXA4,HOXA-AS3,HOXA5,HOXA-AS3,HOXA6,HOXA-AS3,HOXA-AS3,HOXA7,HOXA7,HOXA9,HOXA10-HOXA9,HOXA10-HOXA9,HOXA10-AS,HOXA10-HOXA9,HOXA10-AS,MIR196B,HOXA10-HOXA9,HOXA10-AS,HOXA10,HOXA10-HOXA9,HOXA10,HOXA11,HOXA13,EVX1-AS,EVX1,HIBADH,HIBADH,TSL,TAX1BP1,JAZF1,JAZF1,JAZF1-AS1,JAZF1-AS1,CREB5,TRIL,LOC100506497,CPVL,CPVL,CPVL,CHN2,CHN2,PRR15,LOC646762,LOC646762,MIR550A3,WIPF3,SCRN1,FKBP14,PLEKHA8,MTURN,LOC105375218,ZNRF2,ZNRF2,MIR550A1,MIR550B1,ZNRF2,NOD1,GGCT,GARS,CRHR2,INMT,INMT-MINDY4,INMT-MINDY4,INMT-MINDY4,MINDY4,AQP1,GHRHR,ADCYAP1R1,NEUROD6,CCDC129,PPP1R17,PDE1C,LSM5,AVL9,AVL9,DPY19L1P1,DPY19L1P1,MIR550A2,MIR550B2,DPY19L1P2,KBTBD2,RP9P,FKBP9 | -0.088101 | 219.589 | 662 | 385.249 |

| | | | | | | | | |
|---|---|---|---|---|---|---|---|---|
| 3 | 15 | 62352431 | 73924960 | VPS13C,C2CD4A,C2CD4B,GOLGA2P11,MGC15885,TLN2,TLN2,MIR190A,TPM1,TPM1,TPM1-AS,LACTB,RPS27L,RAB8B,APH1B,CA12,USP3,USP3,USP3-AS1,USP3-AS1,FBXL22,FBXL22,HERC1,MIR422A,DAPK2,DAPK2,LOC101928988,FAM96A,SNX1,SNX22,SNX22,PPIB,PPIB,CSNK1G1,PCLAF,TRIP4,ZNF609,OAZ2,RBPMS2,RBPMS2,MIR1272,PIF1,PLEKHO2,ANKDD1A,SPG21,MTFMT,SLC51B,RASL12,KBTBD13,UBAP1L,PDCD7,CLPX,CILP,PARP16,IGDCC3,IGDCC4,DPP8,HACD3,INTS14,SLC24A1,SLC24A1,DENND4A,DENND4A,RAB11A,MEGF11,MEGF11,MIR4311,DIS3L,TIPIN,TIPIN,SCARNA14,MAP2K1,MAP2K1,SNAPC5,SNAPC5,RPL4,RPL4,SNORD18C,RPL4,SNORD18B,RPL4,SNORD16,RPL4,SNORD18A,ZWILCH,ZWILCH,LCTL,LCTL,LINC01169,SMAD6,LINC02206,LOC102723493,SMAD3,AAGAB,AAGAB,IQCH,IQCH,IQCH,IQCH-AS1,IQCH-AS1,C15orf61,C15orf61,MAP2K5,SKOR1,PIAS1,CALML4,CLN6,FEM1B,ITGA11,CORO2B,ANP32A,ANP32A,MIR4312,MIR548H4,SPESP1,NOX5,MIR548H4,NOX5,GLCE,PAQR5,PAQR5,LOC145694,LOC145694,KIF23,KIF23,RPLP1,DRAIC,TLE3,TLE3,MIR629,UACA,LARP6,LARP6,LRRC49,LRRC49,LRRC49,THAP10,CT62,THSD4,NR2E3,MYO9A,SENP8,GRAMD2A,PKM,PARP6,CELF6,HEXA,HEXA-AS1,TMEM202,TMEM202,TMEM202-AS1,TMEM202-AS1,ARIH1,MIR630,HIGD2B,BBS4,ADPGK,ADPGK,ADPGK-AS1,ADPGK-AS1,NEO1,HCN4,REC114,NPTN | -0.0906873 | 218.361 | 1697 | 1017.7 |
| 3 | 10 | 33197413 | 35300956 | ITGB1,NRP1,PARD3,CUL2 | -0.0937287 | 188.6 | 101 | 59.6979 |
| 3 | 16 | 5517209 | 10525187 | RBFOX1,TMEM114,METTL22,ABAT,TMEM186,TMEM186,PMM2,PMM2,LOC100130283,CARHSP1,CARHSP1,USP7,C16orf72,GRIN2A,ATF7IP2 | -0.094419 | 181.36 | 194 | 117.139 |
| 3 | 3 | 60500 | 5211565 | CHL1,CHL1,CHL1-AS1,CNTN6,CNTN4,CNTN4,CNTN4-AS1,IL5RA,TRNT1,CRBN,LRRN1,SETMAR,SUMF1,ITPR1,BHLHE40-AS1,BHLHE40-AS1,BHLHE40,BHLHE40,ARL8B | -0.102497 | 212.341 | 288 | 176.768 |
| 3 | 7 | 45928201 | 48289004 | IGFBP1,IGFBP3,LOC730338,TNS3,C7orf65,PKD1L1,PKD1L1,C7orf69,HUS1,SUN3,C7orf57,UPP1,ABCA13 | -0.105548 | 194.491 | 208 | 128.76 |
| 3 | 7 | 48320920 | 57938684 | ABCA13,CDC14C,VWC2,ZPBP,SPATA48,IKZF1,FIGNL1,DDC,DDC,DDC-AS1,GRB10,COBL,POM121L12,VSTM2A,VSTM2A,VSTM2A-OT1,SEC61G,EGFR,EGFR,EGFR-AS1,LANCL2,VOPP1,FKBP9P1,SEPT14,ZNF713,MRPS17,NIPSNAP2,PSPH,CCT6A,CCT6A,SNORA15,SUMF2,PHKG1,CHCHD2,NUPR2,LOC401357,MIR4283-1,MIR4283-2,ZNF479,GUSBP10,MIR3147,ZNF716 | -0.110949 | 199.844 | 482 | 273.962 |

| | | | | | | | | |
|---|---|---|---|---|---|---|---|---|
| 3 | 1 | 70555384 | 86808869 | LRRC7,LRRC40,SRSF11,ANKRD13C,HHLA3,CTH,PTGER3,PTGER3,ZRANB2-AS1,ZRANB2-AS1,ZRANB2,ZRANB2,MIR186,ZRANB2,NEGR1,LRRIQ3,FPGT,FPGT-TNNI3K,FPGT-TNNI3K,FPGT-TNNI3K,TNNI3K,ERICH3,ERICH3,ERICH3-AS1,CRYZ,CRYZ,TYW3,TYW3,LHX8,SLC44A5,ACADM,RABGGTB,RABGGTB,SNORD45C,RABGGTB,SNORD45A,RABGGTB,SNORD45B,MSH4,ASB17,ST6GALNAC3,ST6GALNAC5,PIGK,AK5,ZZZ3,USP33,MIGA1,NEXN,FUBP1,DNAJB4,GIPC2,PTGFR,IFI44L,IFI44,ADGRL4,ADGRL2,TTLL7,PRKACB,SAMD13,DNASE2B,RPF1,GNG5,SPATA1,SPATA1,CTBS,CTBS,LINC01555,SSX2IP,LPAR3,MCOLN2,MCOLN3,WDR63,MIR4423,SYDE2,C1orf52,BCL10,BCL10,LOC646626,DDAH1,CYR61,ZNHIT6,COL24A1 | -0.111902 | 244.27 | 983 | 579.788 |
| 3 | 14 | 96756380 | 97347571 | ATG2B,GSKIP,AK7,PAPOLA,VRK1 | -0.117208 | 237.899 | 108 | 64.1709 |
| 3 | 3 | 93519633 | 98217474 | PROS1,ARL13B,ARL13B,STX19,DHFR2,DHFR2,NSUN3,NSUN3,EPHA6,ARL6,CRYBG3,CRYBG3,RIOX2,RIOX2,GABRR3,OR5AC2,OR5H1,OR5H14,OR5H15,OR5H6,OR5H2,OR5K4,OR5K3,OR5K1,OR5K2 | -0.121496 | 247.048 | 242 | 139.237 |
| 3 | 7 | 94257456 | 99453429 | SGCE,PEG10,PPP1R9A,PON1,PON3,PON2,ASB4,PDK4,DYNC1I1,SLC25A13,SLC25A13,MIR591,SEM1,DLX6-AS1,DLX6,DLX5,SDHAF3,TAC1,ASNS,CZ1P-ASNS,CZ1P-ASNS,MIR5692C2,CZ1P-ASNS,CCZ1P-OR7E38P,OCM2,LMTK2,BHLHA15,TECPR1,BRI3,BRI3,BAIAP2L1,BAIAP2L1,NPTX2,TMEM130,TRRAP,TRRAP,MIR3609,SCARNA28,TRRAP,SCARNA28,LOC101927550,SMURF1,SMURF1,KPNA7,MYH16,ARPC1A,ARPC1B,PDAP1,BUD31,BUD31,PTCD1,ATP5MF-PTCD1,PTCD1,ATP5MF-PTCD1,ATP5MF-PTCD1,CPSF4,ATP5MF-PTCD1,ATP5MF,ZNF789,ZNF789,ZNF394,ZNF394,ZKSCAN5,FAM200A,ZNF655,TMEM225B,ZSCAN25,ZSCAN25,CYP3A5,CYP3A5,CYP3A7-CYP3A51P,CYP3A7-CYP3A51P,CYP3A7,CYP3A4,CYP3A43 | -0.127807 | 232.621 | 655 | 393.879 |
| 3 | 6 | 44364042 | 54025376 | CDC5L,CDC5L,MIR4642,SUPT3H,SUPT3H,MIR586,SUPT3H,RUNX2,RUNX2,CLIC5,ENPP4,ENPP5,RCAN2,RCAN2,LOC101926915,CYP39A1,SLC25A27,SLC25A27,LOC101926934,LOC101926934,TDRD6,TDRD6,PLA2G7,ANKRD66,MEP1A,ADGRF5,ADGRF5,LOC101926962,ADGRF1,TNFRSF21,CD2AP,ADGRF2,ADGRF4,OPN5,PTCHD4,MUT,CENPQ,GLYATL3,C6orf141,RHAG,CRISP2,CRISP3,PGK2,CRISP1,DEFB133,DEFB114,DEFB113,DEFB110,DEFB112,TFAP2D,TFAP2B,PKHD1,MIR206,LINCMD1,MIR133B,IL17A,IL17F,MCM3,PAQR8,EFHC1,TRAM2,TMEM14A,GSTA2,GSTA1,GSTA5,GSTA3,GSTA4,ICK,FBXO9,GCM1,ELOVL5,ELOVL5,MIR5685,GCLC,KLHL31,LRRC1,LOC101927189,MLIP | -0.128409 | 217.94 | 810 | 488.383 |

| | | | | | | | | |
|---|---|---|---|---|---|---|---|---|
| 3 | 7 | 36012743 | 43843466 | EEPD1,KIAA0895,KIAA0895,ANLN,ANLN,AOAH,ELMO1,ELMO1,MIR1200,ELMO1,ELMO1-AS1,GPR141,NME8,SFRP4,EPDR1,STARD3NL,TARP,TRG-AS1,AMPH,FAM183BP,VPS41,POU6F2,POU6F2-AS2,POU6F2,YAE1D1,RALA,CDK13,MPLKIP,SUGCT,INHBA,INHBA,INHBA-AS1,GLI3,C7orf25,PSMA2,MRPL32,HECW1,HECW1,HECW1-IT1,MIR3943,HECW1,LUARIS,STK17A,STK17A,COA1,COA1,BLVRA | -0.128964 | 201.093 | 548 | 321.906 |
| 3 | 3 | 57857978 | 78767056 | SLMAP,FLNB,FLNB,FLNB-AS1,DNASE1L3,ABHD6,HTD2,RPP14,PXK,PDHB,KCTD6,ACOX2,FAM107A,FAM107A,FAM3D-AS1,FAM3D-AS1,FAM3D,FAM3D,C3orf67,C3orf67,C3orf67-AS1,FHIT,PTPRG,PTPRG,PTPRG-AS1,C3orf14,FEZF2,CADPS,SYNPR,SYNPR,SYNPR-AS1,SNTN,C3orf49,C3orf49,THOC7,THOC7,ATXN7,PSMD6-AS2,PSMD6,PSMD6,PRICKLE2-AS1,PRICKLE2,PRICKLE2,PRICKLE2,PRICKLE2-AS3,ADAMTS9,ADAMTS9,ADAMTS9-AS1,ADAMTS9,ADAMTS9-AS2,MAGI1,SLC25A26,LRIG1,KBTBD8,MIR4272,SUCLG2,FAM19A1,FAM19A4,EOGT,TMF1,TMF1,MIR3136,UBA3,ARL6IP5,LMOD3,FRMD4B,MITF,FOXP1,FOXP1,MIR1284,EIF4E3,EIF4E3,GPR27,GPR27,PROK2,RYBP,SHQ1,GXYLT2,PPP4R2,PPP4R2,EBLN2,PDZRN3,PDZRN3,LOC101927296,PDZRN3,PDZRN3-AS1,CNTN3,MIR1324,FRG2C,FRG2EP,ZNF717,ZNF717,MIR4273,ROBO2,ROBO1 | -0.136281 | 198.317 | 1088 | 645.252 |
| 3 | 9 | 1.07E+08 | 114454520 | SMC2,OR13F1,OR13C4,OR13C3,OR13C8,OR13C5,OR13C2,OR13C9,OR13D1,NIPSNAP3A,NIPSNAP3B,ABCA1,SLC44A1,FSD1L,FKTN,TAL2,TMEM38B,ZNF462,ZNF462,LOC340512,RAD23B,KLF4,ACTL7B,ACTL7A,ELP1,FAM206A,CTNNAL1,TMEM245,TMEM245,MIR32,FRRS1L,EPB41L4B,PTPN3,MIR3927,PALM2,PALM2,PALM2-AKAP2,PALM2-AKAP2,PALM2-AKAP2,AKAP2,C9orf152,TXN,TXNDC8,SVEP1,MUSK,LPAR1,OR2K2,KIAA0368,ZNF483,ZNF483,PTGR1,PTGR1,LRRC37A5P,DNAJC25,DNAJC25-GNG10,DNAJC25-GNG10,GNG10,C9orf84 | -0.142381 | 214.564 | 747 | 452.895 |
| 3 | 6 | 38835815 | 39902171 | DNAH8,DNAH8,LOC100131047,GLP1R,SAYSD1,KCNK5,KCNK17,KCNK16,KIF6,DAAM2,DAAM2,LOC100505635,DAAM2,MOCS1,MOCS1 | -0.142384 | 208.902 | 176 | 108.071 |
| 3 | 16 | 47494690 | 49764206 | ITFG1,PHKB,ABCC12,ABCC11,LONP2,LONP2,MIR5095,LONP2,MIR5095,SIAH1,MIR5095,SIAH1,MIR5095,N4BP1,CBLN1,C16orf78,ZNF423 | -0.149029 | 206.072 | 185 | 114.206 |
| 3 | 4 | 10515046 | 17814648 | CLNK,HS3ST1,RAB28,LINC01097,NKX3-2,LINC01096,BOD1L1,MIR5091,CPEB2-AS1,CPEB2,CPEB2,LOC101929095,LOC101929095,C1QTNF7,C1QTNF7,CC2D2A,FBXL5,FAM200B,BST1,CD38,FGFBP1,FGFBP2,PROM1,TAPT1,TAPT1-AS1,LDB2,QDPR,CLRN2,LAP3,MED28,FAM184B,DCAF16,NCAPG | -0.151022 | 194.748 | 412 | 249.734 |

| | | | | | | | | |
|---|---|---|---|---|---|---|---|---|
| 3 | 10 | 27434367 | 32740889 | YME1L1,MASTL,MASTL,ACBD5,ACBD5,LRRC37A6P,ARMC4P1,PTCHD3,RAB18,MKX,ARMC4,MPP7,WAC,BAMBI,C10orf126,LYZL1,SVIL-AS1,SVIL,SVIL,SVIL,MIR604,SVIL,MIR938,JCAD,MTPAP,GOLGA2P6,MAP3K8,LYZL2,SVILP1,ZNF438,ZEB1-AS1,ZEB1,ZEB1,ARHGAP12,KIF5B,EPC1,CCDC7 | -0.152059 | 219.813 | 401 | 241.561 |
| 3 | 3 | 48789041 | 49019190 | PRKAR2A,SLC25A20,ARIH2OS,ARIH2OS,ARIH2,ARIH2 | -0.154596 | 198.648 | 51 | 29.1304 |
| 3 | 9 | 1.39E+08 | 140638023 | SEC16A,C9orf163,NOTCH1,NOTCH1,MIR4673,MIR4674,EGFL7,EGFL7,MIR126,AGPAT2,FAM69B,SNHG7,SNORA17B,SNHG7,SNORA17A,LCN10,LCN6,LCN6,LOC100128593,LCN8,LCN15,TMEM141,CCDC183,CCDC183,CCDC183-AS1,CCDC183-AS1,RABL6,RABL6,RABL6,MIR4292,AJM1,PHPT1,MAMDC4,EDF1,TRAF2,TRAF2,MIR4479,FBXW5,C8G,LCN12,PRR31,PTGDS,LCNL1,PAXX,CLIC3,ABCA2,ABCA2,C9orf139,C9orf139,FUT7,C9orf139,NPDC1,ENTPD2,SAPCD2,UAP1L1,MAN1B1-AS1,MAN1B1,DPP7,GRIN1,LRRC26,LRRC26,MIR3621,TMEM210,ANAPC2,SSNA1,TPRN,TMEM203,NDOR1,RNF208,CYSRT1,RNF224,SLC34A3,TUBB4B,TUBB4B,FAM166A,FAM166A,STPG3-AS1,STPG3,NELFB,TOR4A,NRARP,EXD3,NOXA1,NOXA1,ENTPD8,ENTPD8,NSMF,NSMF,MIR7114,PNPLA7,MRPL41,DPH7,ZMYND19,ARRDC1,ARRDC1-AS1,EHMT1 | -0.157204 | 207.166 | 674 | 427.005 |
| 3 | 9 | 72967111 | 79814290 | SMC5,KLF9,TRPM3,TRPM3,MIR204,TMEM2,ABHD17B,C9orf85,C9orf57,GDA,ZFAND5,TMC1,ALDH1A1,ANXA1,RORB-AS1,RORB,RORB,TRPM6,C9orf40,C9orf41-AS1,CARNMT1,CARNMT1,NMRK1,OSTF1,PCSK5,RFK,GCNT1,PRUNE2,FOXB2,VPS13A-AS1,VPS13A | -0.158752 | 206.609 | 468 | 282.15 |
| 3 | 3 | 1.41E+08 | 146303985 | RASA2,RNF7,GRK7,ATP1B3,TFDP2,GK5,XRN1,ATR,PLS1,TRPC1,PCOLCE2,PAQR9,U2SURP,CHST2,SLC9A9,C3orf58,PLOD2,PLSCR4,PLSCR2,PLSCR1,PLSCR5 | -0.159474 | 228.455 | 426 | 248.234 |
| 3 | 18 | 9256633 | 14763934 | ANKRD12,TWSG1,RALBP1,PPP4R1,RAB31,TXNDC2,VAPA,APCDD1,NAPG,PIEZO2,PIEZO2,MIR6788,SLC35G4,GNAL,GNAL,CHMP1B,GNAL,MPPE1,MPPE1,IMPA2,ANKRD62,CIDEA,TUBB6,TUBB6,AFG3L2,AFG3L2,PRELID3A,SPIRE1,PSMG2,CEP76,PSMG2,PTPN2,SEH1L,CEP192,LDLRAD4,LDLRAD4,MIR5190,LDLRAD4,MIR4526,FAM210A,RNMT,MC5R,MC2R,ZNF519,ANKRD20A5P,POTEC,ANKRD30B | -0.168988 | 214.609 | 520 | 301.143 |
| 3 | 18 | 14851503 | 15381134 | ANKRD30B | -0.169416 | 139.54 | 15 | 7.68813 |
| 3 | 10 | 37508221 | 39076015 | ANKRD30A,ZNF248,ZNF25,ZNF33A,ZNF37A,HSD17B7P2,SEPT7P9,LINC00999 | -0.171599 | 195.729 | 88 | 45.9858 |
| 3 | 15 | 57025696 | 60720911 | ZNF280D,LOC145783,TCF12,TCF12,CGNL1,MYZAP,GCOM1,GCOM1,GCOM1,POLR2M,ALDH1A2,ALDH1A2,LOC283665,AQP9,LIPC,LIPC,LIPC-AS1,ADAM10,ADAM10,HSP90AB4P,MINDY2,SLTM,RNF111,CCNB2,MYO1E,MYO1E,MIR2116,MYO1E,LDHAL6B,FAM81A,GCNT3,GTF2A2,BNIP2,FOXB1,ANXA2,ICE2 | -0.171987 | 207.248 | 363 | 216.026 |

| | | | | | | | | |
|---|---|---|---|---|---|---|---|---|
| 3 | 6 | 18264143 | 25812009 | DEK,RNF144B,MIR548A1HG,MIR548A1,ID4,MBOAT1,E2F3,CDKAL1,SOX4,CASC15,NBAT1,PRL,HDGFL1,NRSN1,DCDC2,DCDC2,KAAG1,MRS2,GPLD1,ALDH5A1,KIAA0319,TDP2,ACOT13,C6orf62,GMNN,C6orf229,RIPOR2,CMAHP,CARMIL1,SCGN,HIST1H2AA,HIST1H2BA,SLC17A4,SLC17A1 | -0.172783 | 193.826 | 396 | 239.889 |
| 3 | 3 | 1.12E+08 | 120449024 | SLC9C1,CD200,BTLA,ATG3,ATG3,SLC35A5,SLC35A5,CCDC80,LOC100129297,CD200R1L,CD200R1L,CD200R1,GTPBP8,NEPRO,BOC,BOC,CFAP44,CFAP44,CFAP44,CFAP44-AS1,SPICE1,SIDT1,SIDT1,MIR4446,USF3,NAA50,ATP6V1A,GRAMD1C,ZDHHC23,CCDC191,QTRT2,DRD3,ZNF80,TIGIT,ZBTB20,MIR568,ZBTB20,ZBTB20,ZBTB20-AS1,ZBTB20,MIR4796,GAP43,LSAMP,MIR4447,LOC105374060,IGSF11,C3orf30,UPK1B,B4GALT4,B4GALT4,B4GALT4-AS1,ARHGAP31,TMEM39A,POGLUT1,TIMMDC1,CD80,ADPRH,PLA1A,POPDC2,COX17,MAATS1,NR1I2,GSK3B,GPR156,LRRC58,FSTL1,MIR198,FSTL1,NDUFB4,HGD,RABL3 | -0.173911 | 211.986 | 704 | 429.648 |
| 3 | 2 | 1.8E+08 | 180008530 | TTN,TTN,LOC101927055,CCDC141,SESTD1 | -0.174537 | 244.253 | 122 | 81.2817 |
| 3 | 12 | 4665495 | 4919427 | RAD51AP1,DYRK4,AKAP3,AKAP3,NDUFA9,NDUFA9,GALNT8,KCNA6 | -0.175023 | 218.507 | 71 | 43.1832 |
| 3 | 1 | 16254597 | 16332841 | SPEN,ZBTB17,SRARP | -0.176371 | 211.618 | 56 | 39.7854 |
| 3 | 10 | 60500 | 27284161 | TUBB8,ZMYND11,DIP2C,DIP2C,MIR5699,DIP2C,PRR26,LARP4B,GTPBP4,IDI2,IDI2,IDI2-AS1,IDI2-AS1,IDI2-AS1,IDI1,IDI1,WDR37,ADARB2,ADARB2,ADARB2-AS1,PFKP,PITRM1,PITRM1,PITRM1-AS1,KLF6,AKR1E2,AKR1C1,AKR1C2,AKR1C3,AKR1C8P,AKR1C4,UCN3,TUBAL3,NET1,CALML5,CALML3-AS1,CALML3-AS1,CALML3,ASB13,FAM208B,GDI2,ANKRD16,FBXO18,IL15RA,IL2RA,RBM17,PFKFB3,PFKFB3,MIR3155A,MIR3155B,LOC399715,LOC399716,PRKCQ,SFMBT2,ITIH5,ITIH2,KIN,ATP5F1C,TAF3,GATA3-AS1,GATA3,LOC101928272,CELF2,CELF2,CELF2-AS1,USP6NL,ECHDC3,PROSER2,PROSER2-AS1,UPF2,DHTKD1,SEC61A2,SEC61A2,NUDT5,NUDT5,CDC123,CAMK1D,CAMK1D,MIR4480,CAMK1D,MIR4481,CAMK1D,MIR548Q,CCDC3,OPTN,MCM10,UCMA,PHYH,SEPHS1,BEND7,PRPF18,FRMD4A,MIR4293,FAM107B,CDNF,HSPA14,SUV39H2,SUV39H2,DCLRE1C,DCLRE1C,MEIG1,DCLRE1CP1,ACBD7-DCLRE1CP1,ACBD7-DCLRE1CP1,OLAH,ACBD7-DCLRE1CP1,ACBD7,C10orf111,RPP38,NMT2,NMT2,PPIAP30,FAM171A1,ITGA8,MINDY3,PTER,C1QL3,RSU1,CUBN,TRDMT1,VIM-AS1,VIM,VIM,ST8SIA6,ST8SIA6,ST8SIA6-AS1,HACD1,STAM,TMEM236,MRC1,MRC1,MIR511,MRC1,TMEM236,SLC39A12,SLC39A12,SLC39A12-AS1,CACNB2,NSUN6,ARL5B,MALRD1,PLXDC2,MIR4675,NEBL,NEBL,C10orf113,CASC10,CASC10,MIR1915,SKIDA1,MLLT10,DNAJC1,EBLN1,LOC100130992,COMMD3,COMMD3-BMI1,COMMD3-BMI1,BMI1,SPAG6,PIP4K2A,ARMC3,MSRB2,PTF1A,C10orf67,OTUD1,KIAA1217,KIAA1217,MIR603,ARHGAP21,PRTFDC1,ENKUR,ENKUR,THNSL1,GPR158- | -0.17916 | 197.353 | 2221 | 1303.04 |

| | | | | | | | |
|---|---|---|---|---|---|---|---|
| 3 | 13 | 25442693 | 32893507 | RNF17,CENPJ,TPTE2P1,PABPC3,AMER2,MTMR6,NUP58,ATP8A2,SHISA2,RNF6,CDK8,WASF3,GPR12,USP12,RPL21,RPL21P28,RPL21,RPL21P28,SNORD102,RPL21,RPL21P28,SNORA27,RASL11A,GTF3A,GTF3A,MTIF3,MTIF3,LNX2,POLR1D,GSX1,PDX1,CDX2,URAD,FLT3,PAN3-AS1,PAN3,PAN3,FLT1,POMP,SLC46A3,MTUS2,MTUS2,MTUS2-AS1,SLC7A1,UBL3,LINC00544,KATNAL1,HMGB1,USPL1,ALOX5AP,TEX26-AS1,MEDAG,TEX26,HSPH1,B3GLCT,RXFP2,FRY,ZAR1L,BRCA2 | -0.181997 | 209.521 | 694 | 416.696 |
| 3 | 9 | 27062612 | 33280799 | IFT74,TEK,EQTN,MOB3B,MOB3B,IFNK,C9orf72,LINGO2,LINGO2,MIR876,LINGO2,MIR873,ACO1,DDX58,TOPORS,TOPORS,SMIM27,SMIM27,SMIM27,NDUFB6,NDUFB6,TAF1L,TMEM215,APTX,DNAJA1,SMU1,B4GALT1,B4GALT1,B4GALT1-AS1,SPINK4,BAG1,CHMP5 | -0.183493 | 220.55 | 273 | 165.117 |
| 3 | 7 | 1.44E+08 | 148507547 | NOBOX,TPK1,CNTNAP2,CNTNAP2,MIR548F4,CNTNAP2,MIR548T,C7orf33,CUL1,EZH2 | -0.186643 | 160.333 | 147 | 83.9493 |
| 3 | 9 | 2719607 | 15468751 | KCNV2,PUM3,RFX3,GLIS3,GLIS3,GLIS3-AS1,SLC1A1,SPATA6L,SPATA6L,PLPP6,CDC37L1,AK3,RCL1,RCL1,MIR101-2,JAK2,INSL6,INSL4,RLN2,RLN1,PLGRKT,CD274,PDCD1LG2,RIC1,ERMP1,MLANA,KIAA2026,KIAA2026,MIR4665,RANBP6,IL33,TPD52L3,UHRF2,GLDC,KDM4C,DMAC1,PTPRD,TYRP1,TYRP1,LURAP1L-AS1,LURAP1L-AS1,LURAP1L,LURAP1L,MPDZ,LINC01235,LINC00583,NFIB,ZDHHC21,CER1,FREM1,LOC389705,TTC39B,SNAPC3,PSIP1 | -0.187607 | 200.278 | 742 | 436.111 |
| 3 | 21 | 9411693 | 30442751 | MIR3648-1,MIR3648-2,TEKT4P2,TPTE,BAGE2,BAGE4,BAGE3,BAGE5,BAGE2,BAGE4,BAGE3,BAGE5,BAGE,ANKRD20A11P,LIPI,RBM11,ABCC13,HSPA13,SAMSN1,SAMSN1,SAMSN1-AS1,SAMSN1-AS1,SAMSN1-AS1,LOC388813,LOC388813,NRIP1,USP25,MIR99AHG,MIR99AHG,MIR99A,MIR99AHG,MIRLET7C,MIR99AHG,MIR125B2,LINC01549,CXADR,BTG3,C21orf91,CHODL,TMPRSS15,MIR548XHG,NCAM2,MIR155HG,MIR155,MRPL39,JAM2,ATP5PF,ATP5PF,GABPA,GABPA,APP,CYYR1-AS1,CYYR1,CYYR1,ADAMTS1,ADAMTS5,ADAMTS5,MIR4759,N6AMT1,LTN1,RWDD2B,USP16,CCT8 | -0.193303 | 225.833 | 606 | 339.551 |
| 3 | 20 | 13756487 | 24646159 | ESF1,NDUFAF5,SEL1L2,MACROD2,MACROD2,FLRT3,MACROD2,LOC613266,KIF16B,SNRPB2,OTOR,PCSK2,BFSP1,DSTN,RRBP1,BANF2,SNX5,SNX5,SNORD17,MGME1,OVOL2,PET117,PET117,KAT14,KAT14,ZNF133,LINC00851,DZANK1,POLR3F,POLR3F,MIR3192,RBBP9,SEC23B,SMIM26,DTD1,C20orf78,C20orf78,SCP2D1,SLC24A3,SLC24A3,LOC100130264,RIN2,NAA20,CRNKL1,CRNKL1,CFAP61,CFAP61,INSM1,RALGAPA2,KIZ,KIZ,KIZ-AS1,XRN2,NKX2-4,NKX2-2,PAX1,FOXA2,SSTR4,THBD,CD93,NXT1,GZF1,NAPB,CSTL1,CST11,CST8,CST9L,CST9,CST3,CST4,CST1,CST2,CST5,GGTLC1,SYNDIG1 | -0.199839 | 210.556 | 752 | 448.954 |

| | | | | | | | | |
|---|---|---|---|---|---|---|---|---|
| 3 | 5 | 54683800 | 61642392 | MTREX,MTREX,PLPP1,PLPP1,PLPP1,MIR5687,PLPP1,RNF138P1,SLC38A9,DDX4,IL31RA,IL6ST,FLJ31104,ANKRD55,LINC01948,C5orf67,MAP3K1,SETD9,SETD9,MIER3,MIER3,GPBP1,ACTBL2,PLK2,GAPT,MIR548AE2,LINC02108,RAB3C,PDE4D,DEPDC1B,ELOVL7,ERCC8,NDUFAF2,SMIM15,ZSWIM6,C5orf64,C5orf64,LOC101928651,KIF2A | -0.199978 | 188.233 | 416 | 240.657 |
| 3 | 2 | 32429661 | 37310556 | SLC30A6,NLRC4,YIPF4,BIRC6,BIRC6,MIR558,TTC27,TTC27,MIR4765,LTBP1,MIR4430,RASGRP3,RASGRP3,LOC105374454,FAM98A,LINC01317,MYADML,CRIM1,FEZ2,VIT,STRN,HEATR5B | -0.200701 | 216.057 | 398 | 238.172 |
| 3 | 9 | 19102432 | 26982397 | HAUS6,PLIN2,DENND4C,RPS6,ACER2,SLC24A2,MLLT3,MLLT3,MIR4473,MLLT3,MIR4474,FOCAD,FOCAD,MIR491,HACD4,IFNB1,IFNW1,IFNA21,IFNA7,IFNA16,IFNA14,IFNA5,KLHL9,IFNA6,IFNA13,IFNA2,IFNA8,IFNA1,MIR31HG,IFNE,MIR31HG,MIR31,MTAP,CDKN2A-AS1,CDKN2A,CDKN2B-AS1,CDKN2B,CDKN2B-AS1,DMRTA1,ELAVL2,IZUMO3,TUSC1,LOC100506422,CAAP1,PLAA,PLAA,IFT74,IFT74 | -0.200774 | 215.808 | 361 | 211.491 |
| 3 | 9 | 80030815 | 86354052 | VPS13A,GNA14,GNAQ,CEP78,PSAT1,TLE4,TLE1,SPATA31D5P,SPATA31D4,SPATA31D3,SPATA31D1,RASEF,FRMD3,IDNK,UBQLN1,LOC105376114 | -0.203018 | 208.648 | 290 | 162.395 |
| 3 | 13 | 32930568 | 35619239 | BRCA2,N4BP2L1,N4BP2L2,PDS5B,KL,STARD13,RFC3,NBEA | -0.208253 | 205.079 | 157 | 97.04 |
| 3 | 1 | 16976597 | 17272784 | MST1P2,FAM231AP,FAM231A,FAM231C,ESPNP,FAM231C,FAM231AP,FAM231A,MST1L,LOC102724562,MST1L,LOC440570,CROCC | -0.20833 | 240.206 | 59 | 28.1704 |
| 3 | 17 | 65211891 | 65346500 | HELZ,PSMD12 | -0.208591 | 264.843 | 17 | 9.4384 |
| 3 | 2 | 2.14E+08 | 216289975 | SPAG16,SPAG16,MIR4438,VWC2L,BARD1,LOC101928103,ABCA12,ABCA12,ATIC,FN1 | -0.210698 | 203.531 | 183 | 110.608 |
| 3 | 1 | 35452708 | 35879754 | ZMYM6,ZMYM1,SFPQ,ZMYM4,ZMYM4,ZMYM4-AS1 | -0.216672 | 259.289 | 93 | 56.5201 |
| 3 | 10 | 60154767 | 70666515 | TFAM,BICC1,PHYHIPL,PHYHIPL,FAM13C,FAM13C,SLC16A9,CCDC6,LINC01553,ANK3,CDK1,RHOBTB1,TMEM26,TMEM26,TMEM26-AS1,CABCOCO1,ARID5B,RTKN2,ZNF365,ADO,EGR2,NRBF2,JMJD1C,JMJD1C,MIR1296,JMJD1C,JMJD1C-AS1,JMJD1C,REEP3,REEP3,CTNNA3,CTNNA3,LOC101928961,LRRTM3,CTNNA3,LRRTM3,DNAJC12,SIRT1,HERC4,MYPN,ATOH7,PBLD,HNRNPH3,HNRNPH3,RUFY2,RUFY2,DNA2,SLC25A16,TET1,CCAR1,CCAR1,SNORD98,CCAR1,MIR1254-1,STOX1,DDX50 | -0.218809 | 210.818 | 695 | 421.481 |
| 3 | 5 | 1.33E+08 | 134133006 | FSTL4,FSTL4,MIR1289-2,C5orf15,VDAC1,TCF7,SKP1,PPP2CA,PPP2CA,MIR3661,CDKL3,UBE2B,CDKN2AIPNL,JADE2,SAR1B,SEC24A,CAMLG,DDX46 | -0.223375 | 221.529 | 177 | 102.527 |

| | | | | | | | | |
|---|---|---|---|---|---|---|---|---|
| 3 | 2 | 2.02E+08 | 214149545 | C2CD6,TMEM237,MPP4,ALS2,CDK15,FZD7,KIAA2012,KIAA2012,LOC729224,SUMO1,NOP58,NOP58,SNORD70,NOP58,SNORD70B,NOP58,SNORD11B,NOP58,SNORD11,BMPR2,FAM117B,ICA1L,WDR12,CARF,NBEAL1,CYP20A1,ABI2,RAPH1,CD28,CTLA4,ICOS,PARD3B,NRP2,INO80D,NDUFS1,EEF1B2,EEF1B2,SNORD51,EEF1B2,SNORA41,GPR1,ZDBF2,ADAM23,FAM237A,DYTN,MDH1B,FASTKD2,FASTKD2,MIR3130-1,MIR3130-2,CPO,KLF7,KLF7,MIR2355,LINC01802,MIR1302-4,CREB1,CREB1,METTL21A,METTL21A,CCNYL1,CCNYL1,MIR4775,FZD5,PLEKHM3,LOC100507443,CRYGD,LOC100507443,CRYGC,LOC100507443,CRYGB,CRYGA,C2orf80,IDH1,PIKFYVE,PTH2R,LOC101927960,MAP2,UNC80,RPE,KANSL1L,KANSL1L,LOC101928020,ACADL,MYL1,LANCL1-AS1,LANCL1,LANCL1,CPS1,ERBB4,ERBB4,MIR548F2,MIR4776-1,MIR4776-2,IKZF2,LOC100130451,SPAG16 | -0.22549 | 204 | 1168 | 698.526 |
| 3 | 5 | 49555074 | 54558744 | EMB,PARP8,ISL1,LINC02118,PELO,ITGA1,ITGA1,ITGA2,MOCS2,FST,NDUFS4,ARL15,ARL15,MIR581,ARL15,MIR4459,HSPB3,SNX18,ESM1,GZMK,GZMA,CDC20B,CDC20B,GPX8,CDC20B,MIR449A,MIR449B,CDC20B,MIR449C,MCIDAS,CCNO,DHX29 | -0.22941 | 198.636 | 262 | 157.543 |
| 3 | 20 | 5573997 | 13698190 | GPCPD1,C20orf196,CHGB,TRMT6,MCM8,MCM8,MCM8-AS1,CRLS1,LRRN4,FERMT1,BMP2,HAO1,TMX4,PLCB1,PLCB4,LAMP5-AS1,LAMP5,LAMP5,PAK5,SNAP25-AS1,ANKEF1,SNAP25,MKKS,SLX4IP,JAG1,JAG1,MIR6870,C20orf187,LINC00687,BTBD3,SPTLC3,ISM1,TASP1,ESF1 | -0.231234 | 195.712 | 419 | 254.374 |
| 3 | 12 | 1.01E+08 | 105570002 | GAS2L3,ANO4,SLC5A8,UTP20,ARL1,SPIC,MYBPC1,CHPT1,CHPT1,SYCP3,SYCP3,GNPTAB,DRAM1,WASHC3,NUP37,PARPBP,PARPBP,PMCH,IGF1,PAH,PAH,ASCL1,ASCL1,LOC101929058,C12orf42,STAB2,NT5DC3,TTC41P,HSP90B1,MIR3652,HSP90B1,C12orf73,TDG,GLT8D2,HCFC2,NFYB,TXNRD1,TXNRD1,EID3,CHST11,CHST11,MIR3922,SLC41A2,C12orf45,ALDH1L2,WASHC4,APPL2 | -0.231449 | 217.095 | 671 | 401.808 |

| | | | | | | | | |
|---|---|---|---|---|---|---|---|---|
| 3 | 2 | 39606761 | 55536143 | MAP4K3,TMEM178A,THUMPD2,SLC8A1-AS1,SLC8A1,SLC8A1,C2orf91,PKDCC,LOC102723824,EML4,EML4,COX7A2L,KCNG3,MTA3,OXER1,HAAO,ZFP36L2,LINC01126,THADA,PLEKHH2,PLEKHH2,C1GALT1C1L,DYNC2LI1,DYNC2LI1,ABCG5,ABCG5,ABCG8,LRPPRC,PPM1B,SLC3A1,SLC3A1,PREPL,PREPL,CAMKMT,SIX3,SIX2,SRBD1,PRKCE,EPAS1,TMEM247,ATP6V1E2,RHOQ,RHOQ,LOC100506142,PIGF,CRIPT,SOCS5,LINC01118,LINC01119,MCFD2,MCFD2,TTC7A,TTC7A,STPG4,CALM2,LOC101927043,EPCAM,EPCAM,MIR559,MSH2,KCNK12,MSH6,FBXO11,FOXN2,PPP1R21,STON1-GTF2A1L,STON1-GTF2A1L,STON1,STON1-GTF2A1L,GTF2A1L,STON1-GTF2A1L,LHCGR,FSHR,NRXN1,MIR4431,ASB3,GPR75-ASB3,ASB3,GPR75-ASB3,CHAC2,GPR75-ASB3,ERLEC1,GPR75-ASB3,MIR3682,GPR75-ASB3,GPR75,PSME4,ACYP2,ACYP2,TSPYL6,C2orf73,SPTBN1,SPTBN1,RPL23AP32,EML6,RTN4,CLHC1,RPS27A,RPS27A,MIR4426,MTIF2,CCDC88A | -0.232589 | 199.654 | 1191 | 719 |
| 3 | 6 | 1.28E+08 | 134031460 | PTPRK,PTPRK,LOC101928140,LAMA2,ARHGAP18,TMEM244,L3MBTL3,SAMD3,TMEM200A,SMLR1,EPB41L2,AKAP7,ARG1,ARG1,MED23,MED23,ENPP3,ENPP3,OR2A4,MIR548H5,ENPP1,CTGF,MIR548AJ1,MOXD1,STX7,TAAR9,TAAR8,TAAR6,TAAR5,TAAR3P,TAAR2,TAAR1,VNN1,VNN3,VNN2,SLC18B1,RPS12,RPS12,SNORD101,RPS12,SNORD100,RPS12,SNORA33,EYA4,EYA4,TARID | -0.234771 | 219.858 | 512 | 307.631 |
| 3 | 2 | 95326671 | 95767550 | LOC442028,TEKT4,MAL,MRPS5 | -0.241674 | 230.988 | 25 | 14.106 |
| 3 | 2 | 2.21E+08 | 227368182 | MIR4268,EPHA4,PAX3,PAX3,CCDC140,CCDC140,SGPP2,FARSB,MOGAT1,ACSL3,KCNE4,SCG2,AP1S3,WDFY1,MRPL44,SERPINE2,FAM124B,CUL3,DOCK10,DOCK10,MIR4439,NYAP2 | -0.244021 | 186.931 | 308 | 184.987 |
| 3 | 2 | 37450339 | 39431110 | CEBPZ,CEBPZ,NDUFAF7,NDUFAF7,NDUFAF7,PRKD3,PRKD3,QPCT,CDC42EP3,RMDN2,RMDN2,RMDN2-AS1,CYP1B1,ATL2,LOC101929596,HNRNPLL,GALM,SRSF7,GEMIN6,DHX57,MORN2,ARHGEF33,ARHGEF33,LOC375196,SOS1,CDKL4 | -0.256115 | 212.888 | 253 | 154.406 |

| | | | | | | | | |
|---|---|---|---|---|---|---|---|---|
| 3 | 2 | 97788579 | 179395177 | ANKRD36,ANKRD36B,COX5B,ACTR1B,ZAP70,TMEM131,VWA3B,CNGA3,INPP4A,COA5,UNC50,MGAT4A,KIAA1211L,TSGA10,TSGA10,C2orf15,TSGA10,LIPT1,LIPT1,MITD1,MRPL30,LYG2,LYG1,TXNDC9,EIF5B,REV1,AFF3,LONRF2,CHST10,NMS,PDCL3,NPAS2,NPAS2,LOC101927142,RPL31,RPL31,TBC1D8,TBC1D8,CNOT11,SNORD89,RNF149,MIR5696,CREG2,RFX8,MAP4K4,IL1R2,IL1R1,IL1RL2,IL1RL1,IL18R1,IL18RAP,IL18RAP,MIR4772,SLC9A4,SLC9A2,MFSD9,TMEM182,LOC100287010,POU3F3,LINC01159,LOC102724691,MRPS9,MRPS9,MRPS9,UTAT33,GPR45,TGFBRAP1,C2orf49,FHL2,NCK2,C2orf40,UXS1,PLGLA,CD8B2,ST6GAL2,SLC5A7,SULT1C3,SULT1C2,SULT1C4,GCC2,GCC2,GCC2-AS1,LIMS1,RANBP2,CCDC138,EDAR,SH3RF3-AS1,SH3RF3-AS1,SH3RF3,SH3RF3,MIR4265,SH3RF3,MIR4266,SH3RF3,SEPT10,SOWAHC,RGPD5,RGPD6,LIMS3,LIMS4,LIMS3-LOC440895,MIR4267,MALL,MALL,MIR4436B1,MIR4436B2,NPHP1,MIR4436B1,MIR4436B2,LIMS3-LOC440895,LIMS3,LIMS4,RGPD6,RGPD5,BUB1,ACOXL,BCL2L11,MIR4435-2HG,MIR4435-2HG,MIR4435-1,MIR4435-2,ANAPC1,ANAPC1,MIR4771-1,MIR4771-2,MERTK,TMEM87B,FBLN7,ZC3H8,ZC3H6,RGPD8,RGPD5,TTL,LOC105373562,POLR1B,POLR1B,CHCHD5,SLC20A1,NT5DC4,NT5DC4,CKAP2L,CKAP2L,IL1A,IL1B,IL37,IL36G,IL36A,IL36B,IL36RN,IL1F10,IL1RN,PSD4,PAX8,PAX8,PAX8-AS1,CBWD2,RABL2A,SLC35F5,SLC35F5,MIR4782,LOC101060091,ACTR3,ACTR3,DPP10,DDX18,CCDC93,INSIG2,EN1,MARCO,C1QL2,STEAP3,STEAP3,STEAP3-AS1,C2orf76,DBI,TMEM37,SCTR,SCTR,LOC107105282,CFAP221,TMEM177,PTPN4,EPB41L5,TMEM185B,RALB | -0.258393 | 204.752 | 6288 | 3735.47 |
| 3 | 5 | 61898215 | 68485610 | IPO11,IPO11-LRRC70,HTR1A,RNF180,RGS7BP,SHISAL2B,SREK1IP1,CWC27,ADAMTS6,CENPK,PPWD1,TRIM23,TRAPPC13,SGTB,NLN,ERBIN,SREK1,MAST4,CD180,LINC02219,PIK3R1,SLC30A5,CCNB1,CENPH | -0.264687 | 195.025 | 408 | 242.393 |
| 3 | 5 | 68709931 | 72144239 | RAD17,MARVELD2,OCLN,GTF2H2C_2,GTF2H2C,SERF1A,SERF1B,SMN1,SMN2,GUSBP3,GTF2H2B,GUSBP3,NAIP,GTF2H2,LOC647859,BDP1,MCCC2,CARTPT,MAP1B,MAP1B,MIR4803,MRPS27,PTCD2,ZNF366,TNPO1 | -0.271225 | 194.091 | 268 | 149.086 |
| 3 | 9 | 15509991 | 19058795 | PSIP1,CCDC171,C9orf92,BNC2,CNTLN,SH3GL2,ADAMTSL1,ADAMTSL1,MIR3152,SAXO1,RRAGA,HAUS6 | -0.282433 | 175.951 | 228 | 132.715 |
| 3 | 8 | 1.45E+08 | 145749963 | SCRIB,MIR937,SCRIB,PUF60,NRBP2,NRBP2,MIR6845,EPPK1,PLEC,PLEC,MIR661,PARP10,GRINA,SPATC1,OPLAH,OPLAH,MIR6846,EXOSC4,EXOSC4,MIR6847,GPAA1,CYC1,SHARPIN,MAF1,WDR97,HGH1,MROH1,MIR7112,SCX,BOP1,BOP1,MIR7112,BOP1,SCX,HSF1,DGAT1,DGAT1,MIR6848,SCRT1,TMEM249,TMEM249,FBXL6,FBXL6,SLC52A2,ADCK5,CPSF1,CPSF1,MIR939,CPSF1,MIR6849,SLC39A4,VPS28,TONSL,TONSL,TONSL-AS1,MIR6893,TONSL,TONSL-AS1,CYHR1,KIFC2,FOXH1,PPP1R16A,GPT,MFSD3,RECQL4,LRRC14,LRRC14,LRRC24 | -0.282779 | 169.6 | 599 | 380.406 |

| | | | | | | | | |
|---|---|---|---|---|---|---|---|---|
| 3 | 16 | 70852277 | 71405469 | HYDIN,CMTR2,CALB2 | -0.290602 | 200.394 | 126 | 77.3669 |
| 3 | 15 | 51250719 | 56657956 | AP4E1,MIR4713HG,TNFAIP8L3,MIR4713HG,CYP19A1,MIR4713HG,CYP19A1,MIR4713,GLDN,DMXL2,SCG3,LYSMD2,TMOD2,TMOD3,LEO1,MAPK6,BCL2L10,GNB5,GNB5,CERNA1,CERNA1,MYO5C,MYO5C,MYO5C,MIR1266,MYO5A,ARPP19,FAM214A,ONECUT1,WDR72,UNC13C,RSL24D1,RAB27A,PIGB,PIGB,CCPG1,DNAAF4-CCPG1,CCPG1,DNAAF4-CCPG1,CCPG1,DNAAF4-CCPG1,MIR628,DNAAF4-CCPG1,C15orf65,DNAAF4-CCPG1,C15orf65,DNAAF4,DNAAF4-CCPG1,DNAAF4,PYGO1,PRTG,NEDD4,RFX7,TEX9 | -0.292662 | 206.534 | 591 | 353.87 |
| 3 | 1 | 1.86E+08 | 200817711 | TPR,ODR4,ODR4,OCLM,LOC102724919,PDC,PTGS2,PLA2G4A,BRINP3,RGS18,RGS21,RGS1,RGS13,RGS2,UCHL5,UCHL5,TROVE2,TROVE2,GLRX2,CDC73,CDC73,MIR1278,CDC73,B3GALT2,KCNT2,KCNT2,MIR4735,CFH,CFHR3,CFHR1,CFHR4,CFHR2,CFHR5,F13B,ASPM,ZBTB41,CRB1,DENND1B,C1orf53,LHX9,NEK7,ATP6V1G3,PTPRC,MIR181A1HG,MIR181B1,MIR181A1,NR5A2,LINC00862,ZNF281,KIF14,DDX59,CAMSAP2 | -0.293269 | 235.072 | 705 | 417.594 |
| 3 | 3 | 1.08E+08 | 111870774 | DZIP3,RETNLB,TRAT1,GUCA1C,MORC1,MORC1,MORC1-AS1,C3orf85,DPPA2,DPPA4,LINC01205,NECTIN3,CD96,CD96,ZBED2,PLCXD2,PLCXD2,PHLDB2,PHLDB2,ABHD10,TAGLN3,TMPRSS7,C3orf52,C3orf52,MIR567,GCSAM,SLC9C1 | -0.293332 | 196.478 | 261 | 157.477 |
| 3 | 11 | 31085958 | 32861801 | DCDC1,DNAJC24,DNAJC24,IMMP1L,IMMP1L,ELP4,PAX6,PAX6,PAX6-AS1,RCN1,WT1,WT1-AS,EIF3M,EIF3M,CCDC73,CCDC73,PRRG4 | -0.293357 | 199.531 | 158 | 88.9157 |
| 3 | 3 | 15253612 | 37407624 | CAPN7,SH3BP5-AS1,SH3BP5,SH3BP5,METTL6,EAF1,COLQ,COLQ,MIR4270,HACL1,HACL1,BTD,BTD,ANKRD28,ANKRD28,MIR3134,MIR563,GALNT15,DPH3,OXNAD1,OXNAD1,RFTN1,RFTN1,DAZL,PLCL2,PLCL2,MIR3714,TBC1D5,LOC339862,SATB1,KCNH8,KCNH8,MIR4791,EFHB,RAB5A,RAB5A,PP2D1,PP2D1,KAT2B,KAT2B,MIR3135A,SGO1,SGO1,SGO1-AS1,ZNF385D,ZNF385D,ZNF385D-AS1,UBE2E2,UBE2E2,MIR548AC,UBE2E1,NKIRAS1,RPL15,NR1D2,THRB,THRB,LOC101927854,MIR4792,RARB,TOP2B,MIR4442,NGLY1,OXSM,LINC00692,LRRC3B,NEK10,SLC4A7,EOMES,CMC1,AZI2,ZCWPW2,RBMS3,TGFBR2,GADL1,MIR466,STT3B,OSBPL10,ZNF860,GPD1L,CMTM8,CMTM7,CMTM6,DYNC1LI1,CNOT10,TRIM71,CCR4,GLB1,GLB1,TMPPE,CRTAP,SUSD5,FBXL2,FBXL2,UBP1,UBP1,CLASP2,PDCD6IP,ARPP21,ARPP21,MIR128-2,STAC,DCLK3,TRANK1,EPM2AIP1,MLH1,LRRFIP2,LOC152048,GOLGA4,GOLGA4 | -0.29792 | 188.548 | 1356 | 810.479 |
| 3 | 6 | 54025376 | 58745455 | MLIP,TINAG,FAM83B,HCRTR2,GFRAL,HMGCLL1,BMP5,COL21A1,DST,DST,LOC101930010,BEND6,KIAA1586,ZNF451,ZNF451,LOC101927211,BAG2,RAB23,PRIM2,PRIM2,MIR548U,LINC00680-GUSBP4,LINC00680 | -0.303502 | 220.68 | 414 | 253.797 |
| 3 | 10 | 51885769 | 55996729 | WASHC2A,ASAH2,SGMS1,SGMS1-AS1,ASAH2B,A1CF,PRKG1,PRKG1,MIR605,PRKG1,CSTF2T,DKK1,MBL2,PCDH15 | -0.312919 | 176.533 | 176 | 104.857 |

| | | | | | | | | |
|---|---|---|---|---|---|---|---|---|
| 3 | 1 | 2.04E+08 | 203839001 | ZC3H11A,ZBED6,ZC3H11A,SNRPE | -0.313634 | 261.336 | 29 | 15.4506 |
| 3 | 14 | 21680058 | 21841672 | HNRNPC,RPGRIP1,SUPT16H | -0.321846 | 191.984 | 68 | 40.6194 |
| 3 | 3 | 1.6E+08 | 180321113 | KPNA4,ARL14,PPM1L,B3GALNT1,NMD3,SPTSSB,LINC02067,OTOL1,MIR1263,SI,SLITRK3,BCHE,ZBBX,SERPINI2,WDR49,PDCD10,SERPINI1,GOLIM4,EGFEM1P,MIR551B,EGFEM1P,MECOM,TERC,ACTRT3,MYNN,LRRC34,LRRIQ4,LRRC31,SAMD7,SEC62,GPR160,PHC3,PRKCI,SKIL,CLDN11,SLC7A14,LOC101928583,LOC101928583,RPL22L1,EIF5A2,SLC2A2,TNIK,TNIK,MIR569,PLD1,TMEM212,FNDC3B,GHSR,TNFSF10,NCEH1,ECT2,SPATA16,NLGN1,NAALADL2,NAALADL2,NAALADL2-AS3,NAALADL2,NAALADL2-AS2,NAALADL2,MIR4789,TBL1XR1,KCNMB2-AS1,KCNMB2,ZMAT3,PIK3CA,PIK3CA,KCNMB3,KCNMB3,ZNF639,MFN1,GNB4,ACTL6A,MRPL47,NDUFB5,USP13,PEX5L,PEX5L,PEX5L-AS2,TTC14 | -0.321941 | 186.656 | 980 | 588.438 |
| 3 | 15 | 43748041 | 44029073 | TP53BP1,MAP1A,PPIP5K1,CKMT1B,STRC,CATSPER2,PPIP5K1P1-CATSPER2,PPIP5K1P1-CATSPER2,CKMT1A,STRCP1,CATSPER2P1 | -0.323019 | 186.987 | 258 | 149.766 |
| 3 | 2 | 1.98E+08 | 202319063 | PGAP1,ANKRD44,SF3B1,COQ10B,HSPD1,HSPE1,HSPE1-MOB4,HSPE1-MOB4,MOB4,RFTN2,MARS2,BOLL,PLCL1,SATB2,FTCDNL1,C2orf69,TYW5,TYW5,MAIP1,MAIP1,SPATS2L,KCTD18,SGO2,AOX1,BZW1,CLK1,PPIL3,NIF3L1,ORC2,FAM126B,NDUFB3,CFLAR,CFLAR,CFLAR-AS1,CASP10,CASP8,ALS2CR12,TRAK2 | -0.330349 | 194.898 | 510 | 302.04 |
| 3 | 2 | 1.91E+08 | 197737351 | HIBCH,INPP1,MFSD6,NEMP2,NAB1,GLS,STAT1,LOC105373805,STAT4,STAT4,MYO1B,NABP1,CAVIN2,TMEFF2,SLC39A10,DNAH7,STK17B,HECW2,CCDC150,CCDC150,LOC100130452,GTF3C3,C2orf66,PGAP1 | -0.332866 | 195.037 | 434 | 264.635 |
| 3 | 5 | 34716122 | 45907753 | RAI14,TTC23L,RAD1,BRIX1,DNAJC21,AGXT2,PRLR,SPEF2,IL7R,CAPSL,UGT3A1,UGT3A2,LMBRD2,LMBRD2,MIR580,SKP2,NADK2,RANBP3L,SLC1A3,NIPBL,C5orf42,NUP155,WDR70,GDNF,EGFLAM,LIFR,LIFR,LIFR-AS1,MIR3650,OSMR-AS1,OSMR,RICTOR,FYB1,C9,DAB2,PTGER4,TTC33,PRKAA1,RPL37,SNORD72,RPL37,CARD6,C7,MROH2B,C6,PLCXD3,OXCT1,OXCT1,OXCT1-AS1,C5orf51,FBXO4,GHR,CCDC152,CCDC152,SELENOP,SELENOP,LOC648987,ANXA2R,LOC648987,LOC100132356,ZNF131,NIM1K,HMGCS1,CCL28,TMEM267,C5orf34,PAIP1,NNT,FGF10,MRPS30,HCN1 | -0.336101 | 194.931 | 1047 | 630.416 |

| | | | | | | | | |
|---|---|---|---|---|---|---|---|---|
| 3 | 12 | 21623127 | 34431528 | PYROXD1,RECQL,RECQL,GOLT1B,SPX,GYS2,LDHB,KCNJ8,ABCC9,CMAS,ST8SIA1,C2CD5,ETNK1,SOX5,SOX5,MIR920,LINC00477,BCAT1,C12orf77,LRMP,CASC1,ETFRF1,KRAS,LMNTD1,MIR4302,RASSF8,BHLHE41,SSPN,ITPR2,INTS13,FGFR1OP2,TM7SF3,MED21,C12orf71,STK38L,ARNTL2,ARNTL2,ARNTL2-AS1,SMCO2,PPFIBP1,REP15,MRPS35,MANSC4,KLHL42,PTHLH,CCDC91,FAR2,FAR2,LOC100506606,ERGIC2,OVCH1-AS1,OVCH1-AS1,OVCH1,OVCH1,TMTC1,IPO8,CAPRIN2,LINC00941,TSPAN11,DDX11,SINHCAF,SINHCAF,FLJ13224,DENND5B,DENND5B,DENND5B-AS1,ETFBKMT,AMN1,H3F3C,KIAA1551,BICD1,FGD4,DNM1L,YARS2,PKP2,SYT10,ALG10 | -0.338546 | 190.823 | 1100 | 644.13 |
| 3 | 7 | 33069212 | 34981501 | NT5C3A,RP9,BBS9,BMPER,NPSR1-AS1,NPSR1-AS1,NPSR1,NPSR1,NPSR1-AS1,NPSR1,DPY19L1 | -0.339666 | 167.941 | 111 | 63.1614 |
| 3 | 16 | 46597745 | 47252242 | ANKRD26P1,SHCBP1,VPS35,ORC6,MYLK3,C16orf87,GPT2,DNAJA2,NETO2,ITFG1-AS1,ITFG1 | -0.341345 | 207.46 | 123 | 71.3715 |
| 3 | 1 | 63027248 | 64038210 | DOCK7,DOCK7,ANGPTL3,ATG4C,FOXD3-AS1,FOXD3,ALG6,ITGB3BP,EFCAB7,EFCAB7,DLEU2L | -0.343041 | 212.157 | 103 | 58.1613 |
| 3 | 14 | 53251943 | 56104645 | FERMT2,DDHD1,MIR5580,BMP4,CDKN3,CNIH1,GMFB,CGRRF1,SAMD4A,GCH1,GCH1,MIR4308,WDHD1,SOCS4,MAPK1IP1L,LGALS3,DLGAP5,FBXO34,ATG14,TBPL2,KTN1-AS1,KTN1 | -0.344029 | 200.598 | 257 | 153.043 |

| | | | | | | | | |
|---|---|---|---|---|---|---|---|---|
| 3 | 5 | 72359596 | 116733567 | FCHO2,TMEM171,TMEM174,FOXD1,BTF3,ANKRA2,UTP15,ARHGEF28,ENC1,HEXB,HEXB,GFM2,GFM2,GFM2,NSA2,NSA2,FAM169A,GCNT4,ANKRD31,HMGCR,COL4A3BP,COL4A3BP,POLK,POLK,ANKDD1B,POC5,SV2C,IQGAP2,IQGAP2,F2RL2,F2R,F2RL1,S100Z,CRHBP,AGGF1,ZBED3,ZBED3,SNORA47,PDE8B,WDR41,OTP,TBCA,AP3B1,SCAMP1,LHFPL2,ARSB,DMGDH,BHMT2,BHMT,JMY,HOMER1,PAPD4,CMYA5,MTX3,THBS4,THBS4,CTD-2201I18.1,SERINC5,LOC644936,SPZ1,ZFYVE16,FAM151B,ANKRD34B,DHFR,DHFR,MTRNR2L2,DHFR,MSH3,MSH3,RASGRF2-AS1,RASGRF2,RASGRF2,RASGRF2,RNU5E-1,RNU5D-1,RNU5E-1,RNU5D-1,CKMT2,CKMT2-AS1,RNU5E-1,RNU5D-1,ZCCHC9,RNU5E-1,RNU5D-1,ACOT12,RNU5E-1,RNU5D-1,SSBP2,SSBP2,ATG10,RPS23,ATP6AP1L,MIR3977,TMEM167A,TMEM167A,SCARNA18,TMEM167A,XRCC4,XRCC4,VCAN,VCAN,VCAN-AS1,HAPLN1,EDIL3,NBPF22P,COX7C,COX7C,SNORD138,MIR4280,LOC101929380,LINC01949,RASA1,CCNH,TMEM161B,LINC00461,MIR9-2,LINC00461,MEF2C-AS2,MEF2C,MEF2C,MEF2C-AS1,MIR3660,CETN3,LOC731157,MBLAC2,POLR3G,LYSMD3,ADGRV1,ARRDC3,ARRDC3,ARRDC3-AS1,NR2F1,FAM172A,MIR2277,FAM172A,FAM172A,POU5F2,KIAA0825,SLF1,MCTP1,FAM81B,TTC37,ARSK,GPR150,RFESD,SPATA9,SPATA9,SPATA9,RHOBTB3,RHOBTB3,GLRX,LINC01554,ELL2,LOC101929710,MIR583,LOC101929710,PCSK1,LOC101929710,CAST,CAST,ERAP1,ERAP1,ERAP2,LNPEP,LIX1,RIOK2,RGMB,RGMB-AS1,RGMB,CHD1,FAM174A,ST8SIA4,ST8SIA4,MIR548P,SLCO4C1,SLCO6A1,PAM,GIN1,PPIP5K2,C5orf30,NUDT12,EFNA5,FBXL17,FER,PJA2,MAN2A1,LINC01848,TME | -0.344889 | 188.212 | 2693 | 1606.1 |
| 3 | 14 | 91927672 | 92628196 | PPP4R3A,CATSPERB,TC2N,FBLN5,TRIP11,ATXN3,NDUFB1,CPSF2 | -0.346415 | 225.609 | 157 | 88.821 |
| 3 | 14 | 60952291 | 64560254 | SIX6,SIX1,SIX4,MNAT1,TRMT5,TRMT5,SLC38A6,SLC38A6,TMEM30B,PRKCH,LINC01303,FLJ22447,FLJ22447,HIF1A-AS1,HIF1A,HIF1A,HIF1A,LOC105370526,HIF1A,LOC105370526,HIF1A-AS2,SNAPC1,SYT16,LINC00643,KCNH5,RHOJ,GPHB5,PPP2R5E,WDR89,SGPP1,SYNE2 | -0.346998 | 193.981 | 306 | 183.445 |
| 3 | 15 | 60770053 | 62238089 | ICE2,RORA-AS1,RORA,RORA,VPS13C | -0.350824 | 189.037 | 81 | 48.1246 |
| 3 | 6 | 61944508 | 76330662 | KHDRBS2,LGSN,PTP4A1,PHF3,EYS,EYS,LOC441155,ADGRB3,LMBRD1,COL19A1,COL9A1,FAM135A,SDHAF4,SMAP1,SMAP1,B3GAT2,B3GAT2,OGFRL1,MIR30C2,MIR30A,RIMS1,KCNQ5,KCNQ5,MIR4282,KHDC1L,KHDC1,DPPA5,KHDC3L,OOEP,DDX43,CGAS,MTO1,EEF1A1,EEF1A1,SNORD141A,SNORD141B,SLC17A5,LOC101928489,CD109,CD109,COL12A1,COX7A2,TMEM30A,FILIP1,FILIP1,LOC101928540,FILIP1,LOC101928540,MIR4463,SENP6 | -0.351778 | 184.742 | 779 | 456.505 |

| | | | | | | | | |
|---|---|---|---|---|---|---|---|---|
| 3 | 3 | 99568630 | 108270213 | MIR548G,CMSS1,FILIP1L,MIR548G,CMSS1,FILIP1L,MIR3921,CMSS1,TMEM30CP,TBC1D23,NIT2,TOMM70,LNP1,TMEM45A,ADGRG7,TFG,ABI3BP,IMPG2,SENP7,FAM172BP,TRMT10C,PCNP,ZBTB11,ZBTB11,ZBTB11-AS1,RPL24,CEP97,NXPE3,NFKBIZ,ZPLD1,ALCAM,CBLB,CCDC54,BBX,LINC00636,CD47,IFT57,HHLA2,MYH15,CIP2A | -0.359527 | 187.122 | 555 | 336.707 |
| 3 | 2 | 2.38E+08 | 238940980 | MLPH,PRLH,RAB17,LRRFIP1,RBM44,RAMP1,UBE2F,UBE2F-SCLY | -0.361583 | 167.809 | 100 | 58.0837 |
| 3 | 9 | 1.15E+08 | 115204076 | UGCG,UGCG,MIR4668,SUSD1,PTBP3,HSDL2 | -0.369167 | 160.719 | 74 | 42.297 |
| 3 | 9 | 1.41E+08 | 141152931 | EHMT1,EHMT1,LOC651337,MIR602,LOC100133077,CACNA1B,CACNA1B,CACNA1B,LOC101928786,TUBBP5 | -0.37012 | 183.887 | 100 | 59.8442 |
| 3 | 2 | 75928325 | 84915738 | GCFC2,LRRTM4,REG3G,REG1B,REG1A,REG3A,CTNNA2,CTNNA2,MIR4264,CTNNA2,LRRTM1,FUNDC2P2,SUCLG1,DNAH6 | -0.370536 | 153.685 | 201 | 125.102 |
| 3 | 3 | 1.58E+08 | 158970638 | RSRC1,MLF1,GFM1,GFM1,LXN,RARRES1,MFSD1,IQCJ,IQCJ-SCHIP1 | -0.371427 | 192.216 | 103 | 57.9472 |
| 3 | 9 | 86407814 | 88934648 | GKAP1,KIF27,C9orf64,HNRNPK,HNRNPK,MIR7-1,RMI1,SLC28A3,NTRK2,AGTPBP1,LOC389765,NAA35,GOLM1,C9orf153,ISCA1,ZCCHC6 | -0.375595 | 204.407 | 232 | 136.15 |
| 3 | 11 | 88961654 | 90601745 | TYR,NOX4,FOLH1B,TRIM77,TRIM49,TRIM64B,TRIM64,TRIM49C,UBTFL1,NAALAD2,CHORDC1,DISC1FP1,MIR4490 | -0.377348 | 231.391 | 131 | 68.1013 |
| 3 | 4 | 1.65E+08 | 170675058 | MARCH1,MARCH1,ANP32C,TRIM61,FAM218A,TRIM61,TRIM60,TMEM192,KLHL2,KLHL2,GK3P,MSMO1,CPE,CPE,MIR578,TLL1,SPOCK3,ANXA10,DDX60,DDX60L,PALLD,CBR4,SH3RF1,NEK1,CLCN3,HPF1 | -0.385525 | 177.038 | 392 | 226.013 |
| 3 | X | 1.54E+08 | 155260060 | RAB39B,CLIC2,TMLHE-AS1,LOC101927830,TMLHE-AS1,LOC101927830,TMLHE,TMLHE,SPRY3,VAMP7,IL9R | -0.388027 | 125.505 | 54 | 26.7772 |
| 3 | 14 | 50092260 | 53101810 | DNAAF2,POLE2,KLHDC1,KLHDC2,NEMF,ARF6,LINC01588,LINC01588,LINC01599,LINC01599,VCPKMT,SOS2,L2HGDH,L2HGDH,MIR4504,ATP5S,CDKL1,MAP4K5,ATL1,SAV1,NIN,NIN,LOC105370489,ABHD12B,PYGL,TRIM9,TMX1,FRMD6,GNG2,GNG2,LOC102723604,RTRAF,NID2,PTGDR,PTGER2,TXNDC16,GPR137C | -0.388533 | 201.12 | 504 | 297.156 |
| 3 | 16 | 66861886 | 68344972 | NAE1,CA7,PDP2,CDH16,RRAD,FAM96B,CES2,CES3,CES4A,CBFB,C16orf70,B3GNT9,TRADD,FBXL8,FBXL8,HSF4,HSF4,NOL3,KIAA0895L,EXOC3L1,E2F4,ELMO3,ELMO3,MIR328,LRRC29,LRRC29,TMEM208,TMEM208,FHOD1,SLC9A5,PLEKHG4,PLEKHG4,KCTD19,KCTD19,LRRC36,TPPP3,ZDHHC1,HSD11B2,ATP6V0D1,AGRP,RIPOR1,CTCF,CARMIL2,CARMIL2,ACD,ACD,PARD6A,ENKD1,C16orf86,GFOD2,RANBP10,TSNAXIP1,CENPT,CENPT,THAP11,NUTF2,EDC4,NRN1L,PSKH1,CTRL,PSMB10,LCAT,LCAT,SLC12A4,SLC12A4,DPEP3,DPEP2,DDX28,DDX28,DUS2,DUS2,NFATC3,ESRP2,ESRP2,MIR6773,PLA2G15,SLC7A6,SLC7A6,SLC7A6OS,SLC7A6OS,SLC7A6OS,PRMT7 | -0.38944 | 181.401 | 696 | 456.234 |

| | | | | | | | |
|---|---|---|---|---|---|---|---|
| 3 | 7 | 1.03E+08 | 127017353 | FBXL13,ARMC10,NAPEPLD,DPY19L2P2,PMPCB,PMPCB,DNAJC2,DNAJC2,PSMC2,PSMC2,SLC26A5,SLC26A5,LOC101927870,RELN,RELN,ORC5,LHFPL3,LHFPL3,LHFPL3-AS2,KMT2E,SRPK2,PUS7,RINT1,RINT1,EFCAB10,EFCAB10,ATXN7L1,CDHR3,SYPL1,NAMPT,CCDC71L,PIK3CG,PRKAR2B,HBP1,COG5,COG5,GPR22,COG5,DUS4L,DUS4L,BCAP29,SLC26A4-AS1,SLC26A4,SLC26A4,LOC101927974,CBLL1,CBLL1,SLC26A3,DLD,LAMB1,LAMB4,NRCAM,PNPLA8,THAP5,DNAJB9,C7orf66,EIF3IP1,IMMP2L,IMMP2L,LRRN3,DOCK4,DOCK4,DOCK4-AS1,ZNF277,IFRD1,LSMEM1,TMEM168,BMT2,GPR85,SMIM30,PPP1R3A,FOXP2,FOXP2,MIR3666,MDFIC,TFEC,TES,CAV2,CAV1,MET,CAPZA2,ST7-AS1,ST7,ST7,ST7-OT4,ST7,ST7,ST7-AS2,ST7,ST7-OT3,WNT2,ASZ1,CFTR,CFTR,CFTR-AS1,CTTNBP2,LSM8,ANKRD7,KCND2,TSPAN12,ING3,CPED1,WNT16,FAM3C,PTPRZ1,AASS,FEZF1,FEZF1,FEZF1-AS1,CADPS2,CADPS2,RNF133,CADPS2,RNF148,TAS2R16,SLC13A1,IQUB,NDUFA5,ASB15,LOC102724555,ASB15,LMOD2,WASL,HYAL4,SPAM1,TMEM229A,GPR37,C7orf77,POT1,GRM8,GRM8,MIR592,ZNF800 | -0.390805 | 200.328 | 1689 | 1020.93 |
| 3 | 7 | 61657308 | 65139352 | ZNF733P,LOC102724738,LOC100287704,LOC100287834,LOC100287834,MIR4283-1,MIR4283-2,ZNF727,ZNF735,ZNF679,ZNF736,ZNF680,ZNF107,ZNF138,ZNF273,ZNF117,ERV3-1-ZNF117,ERV3-1-ZNF117,ERV3-1,CCT6P3,SNORA22C,CCT6P3,INTS4P1,ZNF92,LOC441242,INTS4P2 | -0.394538 | 151.606 | 177 | 82.4329 |
| 3 | 2 | 55920195 | 70131426 | PNPT1,EFEMP1,MIR217HG,MIR217,MIR217HG,MIR216A,MIR217HG,MIR216B,LOC100129434,CCDC85A,CCDC85A,VRK2,VRK2,FANCL,FANCL,MIR4432HG,MIR4432,BCL11A,PAPOLG,REL,PUS10,PUS10,PEX13,PEX13,KIAA1841,C2orf74,AHSA2P,AHSA2P,USP34,USP34,USP34,SNORA70B,XPO1,FAM161A,CCT4,COMMD1,B3GNT2,MIR5192,B3GNT2,TMEM17,EHBP1,EHBP1,LOC100132215,OTX1,WDPCP,MDH1,UGP2,VPS54,PELI1,MIR4433B,MIR4433A,LGALSL,AFTPH,MIR4434,AFTPH,SERTAD2,LINC02245,SLC1A4,CEP68,RAB1A,ACTR2,SPRED2,MIR4778,MEIS1,MEIS1,MEIS1-AS2,ETAA1,C1D,WDR92,WDR92,PNO1,PNO1,PPP3R1,CNRIP1,PLEK,FBXO48,APLF,PROKR1,ARHGAP25,BMP10,GKN2,GKN1,ANTXR1,ANTXR1,MIR3126,GFPT1,NFU1,AAK1,AAK1,SNORA36C,ANXA4,GMCL1,SNRNP27 | -0.400645 | 183.867 | 959 | 569.635 |
| 3 | 8 | 47458041 | 48685233 | LINC00293,LOC100287846,SPIDR,CEBPD | -0.402375 | 137.445 | 61 | 31.9567 |

| | | | | | | | | |
|---|---|---|---|---|---|---|---|---|
| 3 | X | 1.5E+08 | 154275782 | CD99L2,HMGB3,MIR4330,GPR50-AS1,GPR50,GPR50,VMA21,PASD1,PRRG3,FATE1,CNGA2,MAGEA4,GABRE,GABRE,MIR224,GABRE,MIR452,MAGEA5,MAGEA10-MAGEA5,MAGEA10-MAGEA5,MAGEA10,GABRA3,GABRA3,MIR105-1,GABRA3,MIR767,GABRA3,MIR105-2,GABRQ,MAGEA6,CSAG3,MAGEA2,MAGEA2B,CSAG4,MAGEA12,CSAG1,MAGEA3,CETN2,NSDHL,ZNF185,PNMA5,PNMA3,MAGEA1,ZNF275,ZFP92,TREX2,HAUS7,BGN,ATP2B3,CCNQ,DUSP9,PNCK,SLC6A8,BCAP31,ABCD1,PLXNB3,SRPK3,IDH3G,IDH3G,SSR4,SSR4,PDZD4,L1CAM,L1CAM,LCA10,LCA10,AVPR2,ARHGAP4,NAA10,RENBP,HCFC1,TMEM187,MIR3202-1,MIR3202-2,TMEM187,IRAK1,MECP2,OPN1LW,OPN1MW2,OPN1MW,OPN1MW3,TKTL1,FLNA,EMD,RPL10,RPL10,SNORA70,DNASE1L1,DNASE1L1,TAZ,TAZ,ATP6AP1,GDI1,FAM50A,FAM50A,MIR6858,PLXNA3,LAGE3,UBL4A,SLC10A3,FAM3A,G6PD,G6PD,IKBKG,IKBKG,CTAG1B,CTAG1A,CTAG2,GAB3,DKC1,DKC1,SNORA36A,MIR664B,DKC1,SNORA56,MPP1,SMIM9,F8,FUNDC2 | -0.405702 | 174.753 | 952 | 588.509 |
| 3 | 14 | 24624760 | 25347355 | RNF31,IRF9,REC8,REC8,IPO4,IPO4,TM9SF1,TSSK4,CHMP4A,MDP1,NEDD8-MDP1,NEDD8-MDP1,NEDD8,GMPR2,TINF2,TGM1,RABGGTA,DHRS1,NOP9,NOP9,CIDEB,CIDEB,LTB4R2,CIDEB,LTB4R2,LTB4R,LTB4R2,LTB4R,LTB4R,ADCY4,RIPK3,NFATC4,NYNRIN,CBLN3,CBLN3,KHNYN,KHNYN,KHNYN,SDR39U1,SDR39U1,LOC101927045,CMA1,CTSG,GZMH,GZMB,STXBP6 | -0.41545 | 168.898 | 316 | 206.377 |
| 3 | X | 1.47E+08 | 149760468 | FMR1,FMR1NB,AFF2,IDS,LINC00893,CXorf40A,CXorf40A,HSFX3,HSFX4,MAGEA9,MAGEA9B,HSFX2,HSFX1,TMEM185A,MAGEA11,HSFX1,HSFX2,MAGEA9B,MAGEA9,MAGEA8-AS1,MAGEA8,CXorf40A,HSFX4,CXorf40A,CXorf40B,LINC00894,MIR2114,MAMLD1 | -0.419345 | 160.42 | 175 | 103.385 |
| 3 | X | 55478779 | 58505576 | MAGEH1,USP51,FOXR2,RRAGB,KLF8,UBQLN2,SPIN3,SPIN2B,SPIN2A,FAAH2,ZXDB,ZXDA | -0.421755 | 174.464 | 114 | 67.6394 |
| 3 | 17 | 64001788 | 64066145 | CEP112 | -0.430507 | 218.032 | 11 | 6.44974 |
| 3 | 11 | 48999653 | 51566742 | TRIM49B,TRIM64C,FOLH1,LOC440040,OR4C13,OR4C12,LOC441601,OR4A5,OR4C46 | -0.441132 | 214.681 | 121 | 60.3126 |
| 3 | 19 | 32851336 | 34699417 | ZNF507,DPY19L3,PDCD5,ANKRD27,RGS9BP,NUDT19,TDRD12,SLC7A9,CEP89,FAAP24,RHPN2,GPATCH1,WDR88,LRP3,LRP3,SLC7A10,SLC7A10,CEBPA,CEBPG,PEPD,CHST8,KCTD15,LSM14A | -0.44312 | 186.057 | 302 | 175.645 |
| 3 | 1 | 1.08E+08 | 109617735 | PRMT6,NTNG1,VAV3,VAV3,VAV3-AS1,SLC25A24,NBPF4,NBPF6,FAM102B,HENMT1,PRPF38B,FNDC7,STXBP3,AKNAD1,GPSM2,GPSM2,CLCC1,CLCC1,WDR47,TAF13 | -0.450395 | 199.166 | 228 | 136.862 |
| 3 | 7 | 76871662 | 87514515 | CCDC146,GSAP,GSAP,LOC101927243,PTPN12,APTR,RSBN1L,RSBN1L,TMEM60,PHTF2,MAGI2,MAGI2,MAGI2-AS3,GNAI1,GNAT3,CD36,SEMA3C,HGF,CACNA2D1,CACNA2D1,LOC101927356,PCLO,SEMA3E,SEMA3A,SEMA3D,GRM3,KIAA1324L,LOC101927420,DMTF1,DMTF1,TMEM243,CROT,ABCB4,ABCB1,ABCB1,RUNDC3B,RUNDC3B,SLC25A40,DBF4 | -0.453747 | 189.46 | 680 | 404.439 |

| | | | | | | | | |
|---|---|---|---|---|---|---|---|---|
| 3 | 4 | 13322 | 438320 | ZNF595,ZNF718,ZNF718,ZNF732,ZNF141,ZNF141,MIR571,ABCA11P,ABCA11P,ZNF721 | -0.45939 | 202.465 | 68 | 36.2423 |
| 3 | 8 | 1.46E+08 | 146303522 | LRRC14,LRRC24,C8orf82,ARHGAP39,ZNF251,ZNF34,RPL8,RPL8,MIR6850,ZNF517,ZNF517,LOC100130027,ZNF7,COMMD5,ZNF250,ZNF16,ZNF252P,ZNF252P,TMED10P1,ZNF252P-AS1,C8orf33 | -0.459589 | 140.753 | 136 | 85.4639 |
| 3 | 11 | 1.19E+08 | 119031823 | DDX6,CXCR5,BCL9L,BCL9L,MIR4492,UPK2,FOXR1,CCDC84,RPS25,TRAPPC4,MIR3656,TRAPPC4,SLC37A4,HYOU1,VPS11,HMBS,H2AFX,DPAGT1,C2CD2L,HINFP,ABCG4 | -0.469687 | 165.026 | 181 | 115.516 |
| 3 | 6 | 76640702 | 82462493 | IMPG1,HTR1B,IRAK1BP1,PHIP,HMGN3,HMGN3,HMGN3-AS1,LCA5,SH3BGRL2,LINC01621,ELOVL4,TTK,BCKDHB,FAM46A | -0.472926 | 168.812 | 222 | 133.723 |
| 3 | 15 | 75518174 | 76136903 | LOC105376731,COMMD4,NEIL1,NEIL1,MIR631,MAN2C1,SIN3A,PTPN9,SNUPN,IMP3,SNX33,CSPG4,ODF3L1,DNM1P35,MIR4313,UBE2Q2 | -0.476948 | 140.321 | 183 | 106.09 |
| 3 | 12 | 9914046 | 10763366 | KLRF1,CLEC2B,KLRF2,CLEC2A,CLEC12A-AS1,CLEC12A,CLEC12A,CLEC1B,CLEC12B,CLEC12B,LOC102724020,CLEC9A,CLEC1A,CLEC7A,OLR1,OLR1,TMEM52B,TMEM52B,GABARAPL1,KLRD1,LOC101928100,KLRK1,KLRC4-KLRK1,KLRC4-KLRK1,KLRC4,KLRC3,KLRC2,KLRC1,EIF2S3L,KLRA1P,MAGOHB | -0.479997 | 198.917 | 173 | 95.3822 |
| 3 | 9 | 1.33E+08 | 139357968 | NCS1,HMCN2,ASS1,FUBP3,PRDM12,EXOSC2,ABL1,QRFP,FIBCD1,LAMC3,AIF1L,NUP214,FAM78A,PLPP7,PRRC2B,PRRC2B,SNORD62A,SNORD62B,POMT1,UCK1,RAPGEF1,MED27,NTNG2,SETX,TTF1,CFAP77,BARHL1,DDX31,DDX31,GTF3C4,GTF3C4,AK8,SPACA9,TSC1,GFI1B,MIR548AW,GFI1B,GTF3C5,GTF3C5,MIR6877,CEL,CELP,RALGDS,GBGT1,OBP2B,ABO,SURF6,MED22,MED22,RPL7A,RPL7A,RPL7A,SNORD24,RPL7A,SNORD36B,RPL7A,SNORD36A,RPL7A,SNORD36C,SURF1,SURF1,SURF2,SURF2,SURF4,SURF4,STKLD1,STKLD1,REXO4,REXO4,ADAMTS13,ADAMTS13,CACFD1,SLC2A6,MYMK,ADAMTSL2,FAM163B,DBH,DBH,DBH-AS1,SARDH,VAV2,BRD3,WDR5,RXRA,MIR4669,RXRA,COL5A1,COL5A1,COL5A1-AS1,COL5A1,LOC101448202,LOC101448202,MIR3689A,MIR3689B,MIR3689D2,LOC101448202,MIR3689A,MIR3689E,MIR3689F,FCN2,FCN1,OLFM1,C9orf62,PPP1R26,C9orf116,MRPS2,MRPS2,LOC101928525,LCN1,OBP2A,PAEP,GLT6D1,LCN9,SOHLH1,KCNT1,CAMSAP1,UBAC1,NACC2,TMEM250,LHX3,QSOX2,DKFZP434A062,GPSM1,DNLZ,CARD9,SNAPC4,SDCCAG3,PMPCA,INPP5E,SEC16A | -0.48507 | 161.588 | 1414 | 867.744 |

| | | | | | | | | |
|---|---|---|---|---|---|---|---|---|
| 3 | X | 46618095 | 53245233 | SLC9A7,RP2,LINC01545,JADE3,RGN,NDUFB11,RBM10,UBA1,UBA1,INE1,CDK16,USP11,ZNF157,ZNF157,SNORA11C,ZNF41,LINC01560,ARAF,SYN1,SYN1,TIMP1,SYN1,MIR4769,CFP,ELK1,UXT,UXT,UXT-AS1,UXT-AS1,ZNF81,ZNF182,SPACA5,SPACA5B,ZNF630-AS1,ZNF630-AS1,ZNF630,ZNF630,SSX6,SSX5,SSX1,SSX9,SSX3,SSX4B,SSX4,SSX4,SSX4B,SLC38A5,FTSJ1,PORCN,EBP,TBC1D25,RBM3,WDR13,WAS,SUV39H1,GLOD5,GATA1,HDAC6,ERAS,PCSK1N,TIMM17B,PQBP1,SLC35A2,PIM2,OTUD5,KCND1,GRIPAP1,TFE3,CCDC120,PRAF2,WDR45,GPKOW,MAGIX,PLP2,PRICKLE3,SYP,SYP,SYP-AS1,CACNA1F,CCDC22,CCDC22,FOXP3,FOXP3,PPP1R3F,GAGE10,GAGE1,PAGE1,PAGE4,USP27X-AS1,USP27X,CLCN5,CLCN5,MIR532,CLCN5,MIR188,CLCN5,MIR500A,CLCN5,MIR362,CLCN5,MIR501,CLCN5,MIR500B,CLCN5,MIR660,CLCN5,MIR502,AKAP4,CCNB3,DGKK,SHROOM4,BMP15,NUDT10,CXorf67,NUDT11,GSPT2,MAGED1,MAGED4B,MAGED4,MAGED4B,MAGED4,SNORA11D,SNORA11E,XAGE2,SSX8,SSX7,SSX2B,SSX2,SSX2,SSX2B,SPANXN5,XAGE5,XAGE3,FAM156B,FAM156A,GPR173,TSPYL2,KDM5C,KDM5C,MIR6895,KDM5C,MIR6894 | -0.485116 | 167.027 | 1058 | 651.982 |
| 3 | X | 1.39E+08 | 146993762 | ATP11C,MIR505,ATP11C,CXorf66,LOC728660,SOX3,LINC00632,CDR1,MIR320D2,SPANXB1,LDOC1,SPANXA2-OT1,LOC645188,SPANXD,SPANXC,MAGEC3,MAGEC1,MAGEC2,SPANXN4,SPANXN3,SLITRK4,SPANXN2,UBE2NL,SPANXN1,SLITRK2,MIR890,MIR888,MIR892A,MIR892B,MIR891B,MIR891A,CXorf51A,CXorf51B,MIR513C,MIR513B,MIR513A1,MIR513A2,MIR506,MIR506,MIR507,MIR508,MIR514B,MIR509-1,MIR509-2,MIR509-3,MIR509-3,MIR510,FMR1 | -0.485995 | 131.916 | 199 | 113.419 |
| 3 | 17 | 65718635 | 65739894 | NOL11,NOL11,SNORA38B | -0.486932 | 235.825 | 21 | 9.97574 |
| 3 | 8 | 1.25E+08 | 124798048 | FAM91A1 | -0.491065 | 190.463 | 11 | 5.5489 |
| 3 | 8 | 1.42E+08 | 144894625 | GPR20,PTP4A3,MROH5,MIR1302-7,MIR4472-1,TSNARE1,ADGRB1,ARC,JRK,PSCA,LY6K,LY6K,LOC100288181,THEM6,SLURP1,LYPD2,SLURP2,LYNX1-SLURP2,LYNX1-SLURP2,LYNX1,LY6D,GML,CYP11B1,CYP11B2,LY6E-DT,LY6E,C8orf31,LY6H,GPIHBP1,ZFP41,GLI4,ZNF696,TOP1MT,RHPN1,MAFA,ZC3H3,GSDMD,MROH6,NAPRT,EEF1D,TIGD5,PYCR3,TSTA3,ZNF623,ZNF707,CCDC166,MAPK15,FAM83H,FAM83H,MIR4664,FAM83H-AS1,SCRIB | -0.507965 | 143.645 | 472 | 293.131 |
| 3 | 3 | 78767556 | 90311186 | ROBO1,ROBO1,MIR3923,GBE1,CADM2,CADM2,MIR5688,CADM2,CADM2-AS2,VGLL3,MIR4795,CHMP2B,POU1F1,HTR1F,CGGBP1,CGGBP1,ZNF654,C3orf38,EPHA3 | -0.512095 | 134.894 | 217 | 128.495 |
| 3 | 2 | 1.8E+08 | 179572573 | MIR548N,TTN,TTN | -0.512768 | 206.99 | 96 | 59.1276 |

| | | | | | | | | |
|---|---|---|---|---|---|---|---|---|
| 3 | 14 | 56129561 | 60900146 | KTN1,PELI2,TMEM260,OTX2,EXOC5,AP5M1,NAA30,CCDC198,SLC35F4,C14orf37,ACTR10,PSMA3,PSMA3,PSMA3-AS1,PSMA3-AS1,ARID4A,TOMM20L,TOMM20L,TIMM9,TIMM9,TIMM9,KIAA0586,KIAA0586,DACT1,DAAM1,GPR135,L3HYPDH,JKAMP,JKAMP,CCDC175,CCDC175,RTN1,RTN1,MIR5586,LRRC9,PCNX4,DHRS7,PPM1A | -0.513848 | 178.866 | 441 | 264.978 |
| 3 | 16 | 85145883 | 88815921 | FAM92B,MIR5093,GSE1,GINS2,C16orf74,C16orf74,MIR1910,EMC8,COX4I1,IRF8,LINC00917,FOXF1,MTHFSD,FLJ30679,FOXC2-AS1,FOXC2,FOXC2,FOXL1,C16orf95,FBXO31,MAP1LC3B,ZCCHC14,JPH3,KLHDC4,SLC7A5,CA5A,BANP,LOC400553,ZNF469,ZFPM1,ZFPM1,MIR5189,ZFPM1,LOC100128882,ZC3H18,IL17C,CYBA,MVD,SNAI3-AS1,SNAI3,RNF166,CTU2,PIEZO1,PIEZO1,MIR4722,PIEZO1,LOC100289580,PIEZO1,LOC339059 | -0.515954 | 143.034 | 460 | 285.201 |
| 3 | 12 | 37857194 | 43750404 | ALG10B,CPNE8,KIF21A,ABCD2,C12orf40,C12orf40,SLC2A13,SLC2A13,LRRK2,MUC19,CNTN1,PDZRN4,GXYLT1,YAF2,ZCRB1,PPHLN1,PRICKLE1,ADAMTS20 | -0.520692 | 180.496 | 465 | 276.515 |
| 3 | 12 | 15090834 | 20968171 | ERP27,ARHGDIB,PDE6H,RERG,PTPRO,EPS8,STRAP,DERA,SLC15A5,MGST1,LMO3,SKP1P2,MIR3974,RERGL,PIK3C2G,PLCZ1,CAPZA3,PLEKHA5,AEBP2,PDE3A,SLCO1C1 | -0.526261 | 169.276 | 340 | 198.105 |
| 3 | 1 | 10500 | 3392682 | FAM87B,LINC00115,LINC01128,SAMD11,SAMD11,NOC2L,NOC2L,KLHL17,PLEKHN1,PERM1,HES4,ISG15,AGRN,RNF223,C1orf159,LINC01342,MIR200B,MIR200A,MIR429,TTLL10,TNFRSF18,TNFRSF4,SDF4,B3GALT6,C1QTNF12,UBE2J2,SCNN1D,ACAP3,ACAP3,MIR6726,PUSL1,PUSL1,INTS11,INTS11,INTS11,MIR6727,CPTP,TAS1R3,DVL1,DVL1,MIR6808,MXRA8,AURKAIP1,CCNL2,LOC148413,MRPL20,ANKRD65,TMEM88B,VWA1,ATAD3C,ATAD3B,ATAD3A,TMEM240,SSU72,FNDC10,LOC105378586,MIB2,MMP23B,MMP23B,MMP23A,CDK11B,CDK11B,SLC35E2B,CDK11B,MMP23A,CDK11B,CDK11A,SLC35E2,NADK,GNB1,CALML6,TMEM52,CFAP74,GABRD,PRKCZ,PRKCZ,PRKCZ-AS1,PRKCZ,FAAP20,FAAP20,SKI,MORN1,RER1,PEX10,PLCH2,PANK4,HES5,TNFRSF14-AS1,TNFRSF14,TNFRSF14,FAM213B,FAM213B,MMEL1,MMEL1,TTC34,ACTRT2,LINC00982,PRDM16,PRDM16,MIR4251,ARHGEF16 | -0.527633 | 156.723 | 910 | 547.789 |

| | | | | | | | | |
|---|---|---|---|---|---|---|---|---|
| 3 | 19 | 34960722 | 36832061 | WTIP,ZNF807,SCGB1B2P,SCGB2B2,ZNF302,ZNF181,ZNF599,ZNF30,ZNF792,GRAMD1A,SCN1B,HPN,HPN,HPN-AS1,HPN-AS1,FXYD3,LGI4,FXYD1,FXYD7,FXYD5,FAM187B,LSR,USF2,HAMP,MAG,CD22,CD22,MIR5196,FFAR1,FFAR3,GPR42,LINC01531,FFAR2,KRTDAP,DMKN,SBSN,GAPDHS,GAPDHS,TMEM147-AS1,TMEM147-AS1,TMEM147,TMEM147,ATP4A,HAUS5,RBM42,ETV2,COX6B1,UPK1A,UPK1A,UPK1A-AS1,ZBTB32,KMT2B,IGFLR1,IGFLR1,U2AF1L4,U2AF1L4,PSENEN,LIN37,HSPB6,PROSER3,ARHGAP33,ARHGAP33,LINC01529,PRODH2,NPHS1,KIRREL2,APLP1,NFKBID,HCST,TYROBP,LRFN3,SDHAF1,SYNE4,ALKBH6,LOC101927572,CLIP3,LOC101927572,THAP8,THAP8,WDR62,OVOL3,OVOL3,POLR2I,POLR2I,TBCB,CAPNS1,COX7A1,ZNF565,ZNF146,ZFP14 | -0.529546 | 163.981 | 686 | 435.028 |
| 3 | 11 | 1.03E+08 | 110102175 | DCUN1D5,DYNC2H1,MIR4693,PDGFD,PDGFD,DDI1,CASP12,CASP4,CASP5,CASP1,CARD16,CARD17,CARD18,GRIA4,MSANTD4,KBTBD3,KBTBD3,AASDHPPT,AASDHPPT,GUCY1A2,CWF19L2,ALKBH8,ELMOD1,SLN,SLC35F2,RAB39A,CUL5,ACAT1,NPAT,ATM,ATM,C11orf65,C11orf65,KDELC2,EXPH5,DDX10,C11orf87,ZC3H12C,RDX | -0.533979 | 180.435 | 607 | 360.855 |
| 3 | 1 | 2.27E+08 | 227504224 | CDC42BPA | -0.535496 | 210.599 | 28 | 15.3652 |

| | | | | | | | | |
|---|---|---|---|---|---|---|---|---|
| 3 | 17 | 6003 | 9394549 | DOC2B,RPH3AL,RPH3AL,LOC100506388,LOC105371430,C17orf97,RFLNB,VPS53,FAM57A,GEMIN4,GLOD4,GLOD4,MRM3,MRM3,NXN,TIMM22,ABR,ABR,MIR3183,BHLHA9,TUSC5,YWHAE,CRK,MYO1C,INPP5K,PITPNA,SLC43A2,SCARF1,RILP,PRPF8,TLCD2,MIR22HG,MIR22HG,MIR22,WDR81,SERPINF2,SERPINF1,SMYD4,RPA1,RTN4RL1,DPH1,DPH1,OVCA2,HIC1,SMG6,SRR,SRR,TSR1,TSR1,TSR1,SNORD91B,TSR1,SNORD91A,SGSM2,MNT,LOC284009,METTL16,PAFAH1B1,CLUH,CLUH,MIR6776,CCDC92B,MIR1253,RAP1GAP2,RAP1GAP2,LOC101927911,OR1D5,OR1D2,OR1G1,OR1A2,OR1A1,OR1D4,OR3A2,OR3A1,OR3A4P,OR1E1,OR3A3,OR1E2,SPATA22,SPATA22,ASPA,TRPV3,TRPV1,SHPK,CTNS,TAX1BP3,P2RX5-TAX1BP3,P2RX5-TAX1BP3,EMC6,P2RX5-TAX1BP3,P2RX5-TAX1BP3,P2RX5,ITGAE,ITGAE,HASPIN,NCBP3,CAMKK1,P2RX1,ATP2A3,ZZEF1,CYB5D2,ANKFY1,UBE2G1,SPNS3,SPNS2,SPNS2,MYBBP1A,MYBBP1A,GGT6,SMTNL2,LINC01996,ALOX15,PELP1,PELP1,LOC101559451,ARRB2,MED11,CXCL16,ZMYND15,TM4SF5,VMO1,GLTPD2,PSMB6,PLD2,MINK1,CHRNE,CHRNE,C17orf107,GP1BA,SLC25A11,RNF167,PFN1,ENO3,SPAG7,CAMTA2,INCA1,KIF1C,KIF1C,LOC102724009,SLC52A1,ZFP3,ZNF232,USP6,ZNF594,LOC100130950,SCIMP,RABEP1,RABEP1,NUP88,NUP88,RPAIN,C1QBP,DHX33,DERL2,MIS12,LOC728392,NLRP1,WSCD1,AIPL1,PIMREG,PITPNM3,KIAA0753,TXNDC17,TXNDC17,MED31,MED31,C17orf100,MIR4520-1,MIR4520-2,ALOX15P1,SLC13A5,XAF1,FBXO39,TEKT1,ALOX12-AS1,ALOX12-AS1,ALOX12,RNASEK,RNASEK-C17orf49,RNASEK-C17orf49,C17orf49,RNASEK-C17orf49,C17orf49,MIR497HG,MIR497HG,MIR195,MIR497HG,MIR497,BCL6B,SLC16A13,SLC16A11,CLEC10A | -0.535815 | 158.065 | 2754 | 1720.67 |
| 3 | 19 | 34718741 | 34900409 | KIAA0355,GPI,PDCD2L | -0.536258 | 167.471 | 48 | 31.112 |
| 3 | 17 | 16677361 | 19681064 | KRT16P2,TBC1D27P,TNFRSF13B,LINC02090,MPRIP,PLD6,FLCN,COPS3,NT5M,MED9,RASD1,PEMT,RAI1,SMCR5,RAI1,RAI1,SREBF1,SREBF1,SREBF1,MIR6777,SREBF1,MIR33B,TOM1L2,DRC3,ATPAF2,GID4,DRG2,MYO15A,ALKBH5,LLGL1,FLII,MIEF2,TOP3A,SMCR8,SHMT1,EVPLL,LINC02076,LGALS9C,CCDC144B,TBC1D28,ZNF286B,ZNF286B,FOXO3B,TRIM16L,FBXW10,TVP23B,PRPSAP2,SLC5A10,SLC5A10,FAM83G,GRAP,LOC388436,LOC79999,GRAPL,GRAPL,LOC388436,LOC79999,EPN2,EPN2,B9D1,B9D1,B9D1,MIR1180,MAPK7,MFAP4,RNF112,SLC47A1,SLC47A1,SNORA59B,SNORA59A,ALDH3A2,SLC47A2,ALDH3A1,ULK2 | -0.53758 | 161.325 | 770 | 467.597 |
| 3 | 1 | 16342001 | 16976400 | HSPB7,CLCNKA,CLCNKB,FAM131C,EPHA2,ARHGEF19,RSG1,FBXO42,SZRD1,SPATA21,NECAP2,CROCCP3,LOC440570,CROCCP2,MST1P2 | -0.540518 | 168.767 | 183 | 103.698 |

| | | | | | | | | |
|---|---|---|---|---|---|---|---|---|
| 3 | 11 | 60500 | 8934151 | SCGB1C2,SCGB1C1,ODF3,BET1L,RIC8A,RIC8A,MIR6743,SIRT3,PSMD13,NLRP6,PGGHG,IFITM5,IFITM2,IFITM1,IFITM3,B4GALNT4,PKP3,SIGIRR,ANO9,PTDSS2,RNH1,HRAS,LRRC56,LMNTD2,LMNTD2,LOC692247,RASSF7,MIR210HG,MIR210,PHRF1,IRF7,CDHR5,SCT,DRD4,DEAF1,DEAF1,TMEM80,TMEM80,EPS8L2,TALDO1,GATD1,LOC171391,CEND1,SLC25A22,PIDD1,RPLP2,RPLP2,SNORA52,PNPLA2,CRACR2B,CD151,POLR2L,TSPAN4,CHID1,AP2A2,MUC6,MUC2,MUC5AC,MUC5B,MUC5B,MIR6744,TOLLIP,BRSK2,MOB2,DUSP8,KRTAP5-AS1,KRTAP5-1,KRTAP5-AS1,KRTAP5-2,KRTAP5-AS1,KRTAP5-3,KRTAP5-5,KRTAP5-6,IFITM10,CTSD,SYT8,TNNI2,LSP1,LSP1,MIR4298,LSP1,MIR7847,TNNT3,MRPL23,MRPL23-AS1,HOTS,H19,H19,H19,MIR675,IGF2,INS-IGF2,IGF2,INS-IGF2,MIR483,IGF2,INS-IGF2,IGF2-AS,INS-IGF2,INS-IGF2,INS,TH,MIR4686,ASCL2,C11orf21,C11orf21,TSPAN32,TSPAN32,CD81-AS1,CD81-AS1,CD81,CD81,TSSC4,TRPM5,KCNQ1,KCNQ1,KCNQ1OT1,KCNQ1,KCNQ1-AS1,CDKN1C,SLC22A18AS,SLC22A18AS,SLC22A18,SLC22A18,PHLDA2,NAP1L4,NAP1L4,SNORA54,CARS,CARS,CARS-AS1,OSBPL5,MRGPRG,MRGPRG,MRGPRG-AS1,MRGPRG-AS1,MRGPRE,ZNF195,ART5,ART1,CHRNA10,NUP98,PGAP2,RHOG,STIM1,MIR4687,STIM1,RRM1,OR52B4,TRIM21,OR52K2,OR52K1,OR52M1,C11orf40,OR52I2,OR52I1,TRIM68,OR51D1,OR51E1,OR51E2,OR51F1,OR52R1,OR51F2,OR51S1,OR51H1,OR51T1,OR51A7,OR51G2,OR51G1,OR51A4,OR51A2,MMP26,OR51L1,OR52J3,OR52E2,OR52A5,OR52A1,OR52Z1,OR51V1,HBB,HBD,HBBP1,HBG1,HBG2,HBE1,OR51B4,OR51B2,OR51B5,OR51 | -0.564807 | 165.738 | 2237 | 1401.75 |
| 3 | X | 73045726 | 76813120 | TSIX,XIST,XIST,FTX,FTX,MIR421,MIR374B,MIR374C,FTX,MIR545,MIR374A,ZCCHC13,SLC16A2,RLIM,NEXMIF,ABCB7,UPRT,ZDHHC15,TTC3P1,MAGEE2,PBDC1,MAGEE1,MIR325HG,MIR384,MIR325HG,MIR325,FGF16,ATRX | -0.566119 | 144.596 | 226 | 140.481 |
| 3 | 2 | 72411883 | 72960267 | EXOC6B | -0.569189 | 131.3 | 28 | 16.106 |
| 3 | 8 | 87498730 | 94828755 | RMDN1,CPNE3,CNGB3,CNBD1,DCAF4L2,MMP16,RIPK2,OSGIN2,NBN,DECR1,CALB1,TMEM64,NECAB1,NECAB1,C8orf88,C8orf88,PIP4P2,OTUD6B,LRRC69,LRRC69,MIR4661,LRRC69,SLC26A7,SLC26A7,RUNX1T1,TRIQK,C8orf87,LINC00535,FAM92A,RBM12B,RBM12B,RBM12B-AS1,TMEM67 | -0.572318 | 171.867 | 391 | 223.436 |
| 3 | 2 | 26688576 | 27688439 | OTOF,C2orf70,CIB4,KCNK3,SLC35F6,CENPA,DPYSL5,MAPRE3,TMEM214,AGBL5,OST4,EMILIN1,KHK,KHK,CGREF1,CGREF1,ABHD1,PREB,PRR30,TCF23,SLC5A6,SLC5A6,ATRAID,ATRAID,CAD,SLC30A3,DNAJC5G,TRIM54,UCN,MPV17,GTF3C2,GTF3C2,GTF3C2-AS1,EIF2B4,SNX17,SNX17,ZNF513,ZNF513,PPM1G,NRBP1,KRTCAP3,KRTCAP3,IFT172,IFT172 | -0.5727 | 152.804 | 432 | 278.51 |

| | | | | | | | | |
|---|---|---|---|---|---|---|---|---|
| 3 | 1 | 25701807 | 29387087 | RHCE,MACO1,LDLRAP1,MAN1C1,SELENON,MTFR1L,LOC646471,MTFR1L,MTFR1L,AUNIP,AUNIP,PAQR7,STMN1,STMN1,MIR3917,PAFAH2,EXTL1,SLC30A2,TRIM63,PDIK1L,FAM110D,ZNF593,CNKSR1,CATSPER4,CEP85,SH3BGRL3,UBXN11,UBXN11,CD52,CD52,CRYBG2,ZNF683,LIN28A,DHDDS,DHDDS,LOC101928324,HMGN2,RPS6KA1,RPS6KA1,MIR1976,ARID1A,PIGV,ZDHHC18,SFN,GPN2,GPATCH3,NR0B2,NUDC,KDF1,TRNP1,FAM46B,SLC9A1,WDTC1,TMEM222,SYTL1,MAP3K6,FCN3,CD164L2,GPR3,WASF2,AHDC1,FGR,IFI6,FAM76A,STX12,PPP1R8,PPP1R8,SCARNA1,THEMIS2,RPA2,SMPDL3B,XKR8,EYA3,PTAFR,DNAJC8,ATP5IF1,SESN2,MED18,PHACTR4,SNHG3,RCC1,SNHG3,RCC1,SNORA73B,RCC1,TRNAU1AP,SNHG12,SNORD99,SNHG12,SNHG12,SNORA61,SNHG12,SNORA44,SNHG12,SNORA16A,RAB42,TAF12,GMEB1,YTHDF2,OPRD1,EPB41 | -0.575426 | 153.239 | 826 | 504.786 |
| 3 | 20 | 68259 | 468297 | DEFB125,DEFB126,DEFB127,DEFB128,DEFB129,DEFB132,C20orf96,ZCCHC3,SOX12,NRSN2,TRIB3,RBCK1,TBC1D20,CSNK2A1 | -0.589427 | 149.099 | 83 | 50.6451 |
| 3 | 19 | 38314683 | 49862859 | LOC644554,LOC100631378,WDR87,SIPA1L3,DPF1,PPP1R14A,SPINT2,YIF1B,C19orf33,YIF1B,KCNK6,CATSPERG,PSMD8,GGN,SPRED3,FAM98C,RASGRP4,RYR1,MAP4K1,MAP4K1,LOC105372397,EIF3K,ACTN4,CAPN12,LGALS7,LGALS7B,LGALS4,ECH1,HNRNPL,RINL,SIRT2,NFKBIB,CCER2,SARS2,SARS2,MRPS12,MRPS12,FBXO17,FBXO27,ACP7,PAK4,NCCRP1,SYCN,IFNL3,IFNL2,IFNL1,LRFN1,GMFG,SAMD4B,PAF1,MED29,ZFP36,MIR4530,PLEKHG2,RPS16,SUPT5H,TIMM50,DLL3,SELENOV,EID2B,EID2,LGALS13,LGALS16,LGALS17A,LGALS14,CLC,LEUTX,DYRK1B,FBL,FCGBP,PSMC4,ZNF546,ZNF780B,ZNF780A,MAP3K10,TTC9B,CNTD2,AKT2,AKT2,MIR641,C19orf47,C19orf47,PLD3,PLD3,PLD3,MIR6796,HIPK4,PRX,SERTAD1,SERTAD3,BLVRB,SPTBN4,SHKBP1,LTBP4,NUMBL,COQ8B,ITPKC,C19orf54,SNRPA,MIA,MIA-RAB4B,MIA-RAB4B,RAB4B,RAB4B-EGLN2,RAB4B-EGLN2,EGLN2,CYP2T1P,CYP2A6,CYP2A7,CYP2G1P,CYP2B7P,CYP2B6,CYP2A13,CYP2F1,CYP2S1,AXL,HNRNPUL1,CCDC97,TGFB1,B9D2,TMEM91,EXOSC5,BCKDHA,B3GNT8,DMAC2,ERICH4,PCAT19,CEACAM21,CEACAM4,CEACAM7,CEACAM5,CEACAM6,CEACAM3,LYPD4,DMRTC2,RPS19,RPS19,MIR6797,CD79A,ARHGEF1,LOC100505585,RABAC1,ATP1A3,GRIK5,ZNF574,POU2F2,LOC100505622,MIR4323,DEDD2,ZNF526,GSK3A,ERF,CIC,PAFAH1B3,PAFAH1B3,PRR19,PRR19,TMEM145,MEGF8,MEGF8,MIR8077,CNFN,LOC101930071,LIPE-AS1,LIPE,LIPE-AS1,LIPE,LIPE-AS1,CXCL17,LIPE-AS1,LIPE-AS1,CEACAM1,LIPE-AS1,CEACAM8,PSG3,PSG8,PSG10P,PSG1,PSG6,PSG7,PSG11,PSG2,PSG5,PSG4,PSG9,CD177,TEX101,LYPD3,PHLDB3,ETHE1,ZNF575,XRCC1,PINLYP,IRGQ,ZNF576,ZNF428,ZNF428,SRRM5,CADM4,PLAUR,IRGC,SMG9,KCNN4,LYPD5,ZNF283,ZNF404,ZNF45,ZNF221,ZNF155,ZNF1 | -0.593029 | 158.424 | 3845 | 2356.83 |
| 3 | 15 | 42867891 | 43269095 | STARD9,CDAN1,TTBK2,UBR1 | -0.593441 | 147.222 | 149 | 96.8355 |

| | | | | | | | | |
|---|---|---|---|---|---|---|---|---|
| 3 | 1 | 94645278 | 103548546 | ARHGAP29,ABCD3,F3,SLC44A3-AS1,MIR378G,SLC44A3,CNN3,LOC729970,ALG14,ALG14,LOC101928098,TMEM56,TMEM56-RWDD3,TMEM56,TMEM56-RWDD3,LOC101928118,TMEM56-RWDD3,LOC101928118,TMEM56-RWDD3,RWDD3,RWDD3,PTBP2,DPYD,DPYD,DPYD-AS1,MIR137HG,MIR137HG,MIR2682,MIR137HG,MIR137,SNX7,PLPPR5,PLPPR5,LOC100129620,PLPPR4,PALMD,PALMD,MIR548AA1,MIR548AA1,FRRS1,FRRS1,AGL,SLC35A3,MFSD14A,SASS6,TRMT13,TRMT13,LRRC39,LRRC39,DBT,RTCA,RTCA,MIR553,CDC14A,GPR88,VCAM1,EXTL2,SLC30A7,DPH5,S1PR1,OLFM3,COL11A1 | -0.595499 | 173.82 | 560 | 329.776 |
| 3 | 12 | 48105406 | 50821753 | ENDOU,RAPGEF3,SLC48A1,HDAC7,VDR,TMEM106C,COL2A1,SENP1,SENP1,PFKM,PFKM,ASB8,CCDC184,OR10AD1,H1FNT,ZNF641,ANP32D,C12orf54,OR8S1,LALBA,KANSL2,KANSL2,SNORA2C,MIR1291,KANSL2,SNORA2A,KANSL2,SNORA2B,CCNT1,TEX49,ADCY6,ADCY6,MIR4701,CACNB3,DDX23,RND1,CCDC65,FKBP11,ARF3,WNT10B,WNT1,DDN,DDN,DDN-AS1,DDN-AS1,PRKAG1,KMT2D,RHEBL1,DHH,LMBR1L,TUBA1B,TUBA1A,TUBA1C,LOC101927267,PRPH,LOC101927267,TROAP,TROAP,C1QL4,DNAJC22,SPATS2,KCNH3,MCRS1,FAM186B,PRPF40B,PRPF40B,FMNL3,FMNL3,TMBIM6,NCKAP5L,BCDIN3D-AS1,BCDIN3D,BCDIN3D,FAIM2,LINC02396,AQP2,AQP2,LOC101927318,LOC101927318,AQP5,AQP5,AQP6,RACGAP1,ASIC1,SMARCD1,GPD1,COX14,CERS5,LIMA1,FAM186A,LARP4 | -0.596441 | 145.362 | 933 | 584.479 |
| 3 | 20 | 488972 | 1210717 | CSNK2A1,TCF15,SRXN1,SCRT2,SLC52A3,FAM110A,ANGPT4,RSPO4,PSMF1,TMEM74B,C20orf202,RAD21L1 | -0.601612 | 129.428 | 76 | 45.0728 |
| 3 | 3 | 1.21E+08 | 120969692 | STXBP5L | -0.60224 | 150.388 | 14 | 7.78176 |

| | | | | | | | | |
|---|---|---|---|---|---|---|---|---|
| 3 | 12 | 50854882 | 59284583 | LARP4,DIP2B,ATF1,TMPRSS12,METTL7A,HIGD1C,SLC11A2,LETMD1,LETMD1,CSRNP2,CSRNP2,TFCP2,POU6F1,DAZAP2,DAZAP2,SMAGP,SMAGP,BIN2,CELA1,GALNT6,SLC4A8,SCN8A,FIGNL2,ANKRD33,ACVRL1,ACVR1B,GRASP-AS1,GRASP,GRASP,NR4A1,ATG101,OR7E47P,KRT80,C12orf80,KRT7,KRT7,KRT7-AS,KRT87P,KRT86,KRT81,KRT86,KRT83,KRT85,KRT84,KRT82,KRT75,KRT6B,KRT6C,KRT6A,KRT5,KRT71,KRT74,KRT72,KRT73,KRT73,KRT73-AS1,KRT2,KRT1,KRT77,KRT76,KRT3,KRT4,KRT79,KRT78,KRT8,KRT8,KRT18,KRT18,EIF4B,LOC283335,TNS2,TNS2,TNS2,MIR6757,SPRYD3,IGFBP6,SOAT2,CSAD,CSAD,ZNF740,ZNF740,ITGB7,RARG,MFSD5,ESPL1,PFDN5,C12orf10,AAAS,SP7,SP1,AMHR2,PRR13,PCBP2,PCBP2,PCBP2-OT1,PCBP2,MAP3K12,MAP3K12,TARBP2,NPFF,ATF7,LOC100652999,ATF7,ATP5MC2,CALCOCO1,HOXC13-AS,HOXC13,HOXC13,HOXC12,HOTAIR,HOXC11,HOXC11,HOXC-AS3,HOXC10,HOXC10,MIR196A2,HOXC9,HOXC8,HOXC6,HOXC5,HOXC4,HOXC5,HOXC4,HOXC5,HOXC4,MIR615,HOXC4,FLJ12825,LOC100240734,LINC02381,SMUG1,CBX5,MIR3198-2,CBX5,HNRNPA1,HNRNPA1P10,NFE2,COPZ1,COPZ1,MIR148B,LOC102724050,GPR84,LOC102724050,ZNF385A,LOC102724050,ITGA5,LOC102724050,GTSF1,GTSF1,NCKAP1L,PDE1B,PPP1R1A,GLYCAM1,LACRT,DCD,MUCL1,TESPA1,NEUROD4,OR9K2,OR10A7,OR6C74,OR6C6,OR6C1,OR6C3,OR6C75,OR6C65,OR6C76,OR6C2,OR6C70,OR6C68,OR6C4,OR2AP1,OR10P1,METTL7B,ITGA7,BLOC1S1,BLOC1S1-RDH5,BLOC1S1-RDH5,RDH5,CD63,GDF11,SARNP,ORMDL2,DNAJC14,T | -0.602365 | 153.645 | 2605 | 1606.42 |
| 3 | 15 | 41797933 | 42503973 | LTK,RPAP1,TYRO3,MGA,MGA,MIR626,MAPKBP1,JMJD7,JMJD7-PLA2G4B,JMJD7-PLA2G4B,PLA2G4B,SPTBN5,SPTBN5,MIR4310,SPTBN5,LOC105370792,LOC105370792,EHD4,PLA2G4E-AS1,PLA2G4E,PLA2G4E,PLA2G4D,PLA2G4F,VPS39,VPS39,MIR627,TMEM87A | -0.60572 | 140.597 | 356 | 228.042 |
| 3 | 20 | 1234923 | 3955001 | RAD21L1,SNPH,SDCBP2,FKBP1A-SDCBP2,FKBP1A-SDCBP2,SDCBP2-AS1,FKBP1A-SDCBP2,SDCBP2-AS1,FKBP1A,FKBP1A-SDCBP2,FKBP1A,MIR6869,NSFL1C,SIRPB2,SIRPD,SIRPB1,SIRPG,SIRPG,SIRPG-AS1,SIRPA,PDYN-AS1,PDYN,STK35,TGM3,TGM6,SNRPB,SNRPB,SNORD119,ZNF343,TMC2,NOP56,NOP56,MIR1292,NOP56,SNORD110,NOP56,SNORA51,NOP56,SNORD86,NOP56,SNORD56,NOP56,SNORD57,NOP56,IDH3B,IDH3B,EBF4,CPXM1,C20orf141,TMEM239,PCED1A,PCED1A,VPS16,VPS16,VPS16,PTPRA,PTPRA,GNRH2,MRPS26,OXT,AVP,UBOX5-AS1,UBOX5,UBOX5-AS1,UBOX5,FASTKD5,LZTS3,DDRGK1,ITPA,SLC4A11,C20orf194,ATRN,GFRA4,ADAM33,SIGLEC1,HSPA12B,C20orf27,SPEF1,CENPB,CDC25B,AP5S1,MAVS,PANK2,PANK2,MIR103A2,MIR103B2,RNF24 | -0.609422 | 149.703 | 616 | 378.52 |

| | | | | | | | | |
|---|---|---|---|---|---|---|---|---|
| 3 | 19 | 19905347 | 24364012 | ZNF56,ZNF506,ZNF56,ZNF253,ZNF93,ZNF682,ZNF90,ZNF486,MIR1270,ZNF826P,MIR1270,ZNF737,ZNF626,ZNF66,ZNF85,ZNF430,ZNF714,ZNF431,ZNF708,ZNF738,ZNF493,ZNF429,LOC400682,ZNF100,ZNF100,LOC641367,ZNF43,ZNF208,ZNF257,ZNF676,ZNF729,ZNF98,GOLGA2P9,ZNF492,ZNF99,ZNF728,ZNF730,ZNF724,IPO5P1,ZNF91,ZNF675,ZNF681,ZNF726,ZNF254,HAVCR1P1 | -0.615694 | 165.503 | 455 | 207.175 |
| 3 | 10 | 89514536 | 91465271 | ATAD1,KLLN,PTEN,RNLS,LIPJ,LIPF,LIPK,LIPN,LIPM,LIPM,ANKRD22,ANKRD22,STAMBPL1,ACTA2-AS1,ACTA2,ACTA2,ACTA2,FAS,FAS,CH25H,LIPA,IFIT2,IFIT3,IFIT1B,IFIT1,IFIT5,SLC16A12,SLC16A12,SLC16A12-AS1,PANK1,PANK1,MIR107,KIF20B | -0.61801 | 170.161 | 237 | 142.52 |
| 3 | 8 | 42705875 | 43398986 | RNF170,RNF170,MIR4469,HOOK3,FNTA,POMK,HGSNAT,POTEA | -0.619477 | 158.521 | 95 | 53.1511 |
| 3 | 7 | 87536565 | 94252789 | DBF4,ADAM22,SRI,SRI,LOC102723885,STEAP4,ZNF804B,ZNF804B,TEX47,STEAP2-AS1,STEAP2-AS1,STEAP1,STEAP2,CFAP69,LOC101927446,GTPBP10,CLDN12,CDK14,FZD1,MTERF1,AKAP9,CYP51A1,CYP51A1-AS1,CYP51A1-AS1,LRRD1,KRIT1,ANKIB1,GATAD1,PEX1,RBM48,FAM133B,FAM133DP,CDK6,SAMD9,SAMD9L,HEPACAM2,VPS50,CALCR,CALCR,MIR653,CALCR,MIR489,MIR4652,TFPI2,GNGT1,GNG11,BET1,COL1A2,CASD1,SGCE | -0.620324 | 177.464 | 635 | 386.97 |
| 3 | 16 | 1887762 | 1918190 | FAHD1,MEIOB,MEIOB | -0.628527 | 202.454 | 12 | 7.57558 |
| 3 | X | 62042080 | 72783463 | SPIN4,ARHGEF9,MIR1468,AMER1,ASB12,MTMR8,ZC4H2,ZC3H12B,LAS1L,FRMD8P1,MSN,MIR223,VSIG4,HEPH,EDA2R,AR,OPHN1,YIPF6,STARD8,EFNB1,PJA1,LINC00269,FAM155B,EDA,EDA,MIR676,AWAT2,OTUD6A,IGBP1,DGAT2L6,AWAT1,P2RY4,ARR3,RAB41,PDZD11,KIF4A,GDPD2,DLG3,DLG3,DLG3-AS1,TEX11,SLC7A3,SNX12,FOXO4,CXorf65,IL2RG,MED12,NLGN3,BCYRN1,GJB1,BCYRN1,ZMYM3,BCYRN1,NONO,BCYRN1,ITGB1BP2,BCYRN1,BCYRN1,TAF1,BCYRN1,OGT,BCYRN1,GCNA,BCYRN1,CXCR3,BCYRN1,CXorf49B,CXorf49,CXorf49B,CXorf49,NHSL2,NHSL2,RTL5,FLJ44635,PIN4,PIN4,ERCC6L,RPS4X,CITED1,HDAC8,PHKA1,PHKA1,PHKA1-AS1,FAM236B,FAM236A,DMRTC1,DMRTC1B,FAM226B,FAM226A,PABPC1L2B,PABPC1L2A,NAP1L6,NAP1L2,CDX4,CHIC1 | -0.637338 | 140.269 | 1000 | 599.149 |
| 3 | 18 | 76953831 | 78016748 | ATP9B,NFATC1,LOC284241,CTDP1,KCNG2,PQLC1,HSBP1L1,TXNL4A,RBFA,ADNP2,PARD6G-AS1,PARD6G-AS1,PARD6G,PARD6G | -0.648008 | 144.365 | 147 | 85.702 |
| 3 | 10 | 1.32E+08 | 135524247 | MIR378C,TCERG1L,LINC01164,PPP2R2D,BNIP3,JAKMIP3,DPYSL4,STK32C,LRRC27,PWWP2B,C10orf91,INPP5A,NKX6-2,CFAP46,ADGRA1,KNDC1,UTF1,VENTX,MIR202HG,MIR202,ADAM8,TUBGCP2,TUBGCP2,ZNF511,ZNF511,CALY,PRAP1,FUOM,ECHS1,ECHS1,MIR3944,PAOX,MTG1,SPRN,SCART1,CYP2E1,SYCE1,SYCE1,SPRNP1,FRG2B | -0.64817 | 132.262 | 481 | 288.267 |

| | | | | | | | | |
|---|---|---|---|---|---|---|---|---|
| 3 | 11 | 1.12E+08 | 118622147 | BCO2,PTS,PLET1,PLET1,LOC100132686,LOC101928847,NCAM1,NCAM1,NCAM1,NCAM1-AS1,TTC12,ANKK1,DRD2,DRD2,MIR4301,TMPRSS5,ZW10,CLDN25,USP28,HTR3B,HTR3A,ZBTB16,NNMT,C11orf71,RBM7,REXO2,NXPE1,NXPE4,NXPE2,CADM1,BUD13,ZPR1,APOA5,APOA4,APOC3,APOA1,APOA1,APOA1-AS,APOA1-AS,SIK3,SIK3,PAFAH1B2,SIDT2,SIDT2,LOC100652768,TAGLN,TAGLN,PCSK7,PCSK7,RNF214,BACE1,BACE1,BACE1-AS,CEP164,DSCAML1,FXYD2,FXYD6-FXYD2,FXYD6-FXYD2,FXYD6-FXYD2,FXYD6,TMPRSS13,IL10RA,SMIM35,SMIM35,TMPRSS4,TMPRSS4,SCN4B,SCN2B,JAML,MPZL3,MPZL2,CD3E,CD3D,CD3G,UBE4A,UBE4A,LOC100131626,LOC100131626,ATP5MG,KMT2A,KMT2A,LOC101929089,LOC101929089,TTC36,TTC36,TMEM25,TMEM25,TMEM25,IFT46,IFT46,ARCN1,PHLDB1,PHLDB1,MIR6716,TREH | -0.649525 | 140.861 | 920 | 567.692 |
| 3 | 7 | 10500 | 6845723 | FAM20C,PDGFA,PRKAR1B,PRKAR1B,LOC101926963,PRKAR1B,DNAAF5,DNAAF5,SUN1,GET4,ADAP1,COX19,CYP2W1,C7orf50,C7orf50,MIR339,C7orf50,GPR146,C7orf50,GPER1,ZFAND2A,UNCX,MICALL2,INTS1,MAFK,LOC100128653,MAFK,TMEM184A,PSMG3,PSMG3-AS1,ELFN1,MAD1L1,MAD1L1,MIR4655,MRM2,MRM2,NUDT1,NUDT1,SNX8,SNX8,MIR6836,EIF3B,CHST12,GRIFIN,LFNG,LFNG,MIR4648,BRAT1,IQCE,TTYH3,AMZ1,AMZ1,GNA12,GNA12,CARD11,LOC100129603,SDK1,FOXK1,AP5Z1,AP5Z1,MIR4656,RADIL,RADIL,PAPOLB,MMD2,RNF216P1,RBAK,RBAK-RBAKDN,RBAK-RBAKDN,RBAKDN,ZNF890P,WIPI2,SLC29A4,TNRC18,LOC100129484,FBXL18,FBXL18,MIR589,ACTB,FSCN1,RNF216,RNF216,MIR6874,ZNF815P,OCM,CCZ1,RSPH10B2,RSPH10B,PMS2,AIMP2,AIMP2,EIF2AK1,EIF2AK1,EIF2AK1,ANKRD61,USP42,CYTH3,FAM220A,RAC1,DAGLB,KDELR2,GRID2IP,ZDHHC4,C7orf26,ZNF853,ZNF12,PMS2CL,RSPH10B,RSPH10B2,CCZ1B | -0.651049 | 142.662 | 1163 | 684.941 |
| 3 | 3 | 1.27E+08 | 130648702 | MGLL,KBTBD12,SEC61A1,SEC61A1,RUVBL1,RUVBL1,RUVBL1,EEFSEC,EEFSEC,DNAJB8,GATA2,LINC01565,RPN1,RAB7A,LOC653712,ACAD9,ACAD9,KIAA1257,KIAA1257,KIAA1257,EFCC1,EFCC1,GP9,RAB43,ISY1-RAB43,ISY1-RAB43,ISY1,CNBP,COPG1,COPG1,MIR6826,HMCES,H1FX,RPL32P3,RPL32P3,SNORA7B,EFCAB12,MBD4,IFT122,RHO,H1FOO,PLXND1,TMCC1,TRH,ALG1L2,LINC02014,COL6A5,COL6A6,PIK3R4,ATP2C1 | -0.651997 | 142.323 | 509 | 312.304 |
| 3 | 12 | 753071 | 1769566 | NINJ2,LOC100049716,NINJ2,WNK1,RAD52,ERC1,FBXL14,WNT5B,MIR3649 | -0.654836 | 140.433 | 125 | 77.6841 |
| 3 | 19 | 1274255 | 1585213 | CIRBP,C19orf24,EFNA2,MUM1,NDUFS7,GAMT,DAZAP1,RPS15,APC2,C19orf25,PCSK4,REEP6,ADAMTSL5,PLK5,MEX3D,MBD3 | -0.656365 | 151.995 | 163 | 101.023 |

| | | | | | | | | |
|---|---|---|---|---|---|---|---|---|
| 3 | 22 | 16050500 | 20041345 | DUXAP8,BMS1P22,CCT8L2,ANKRD62P1-PARP4P3,XKR3,GAB4,IL17RA,TMEM121B,LINC01664,HDHD5,HDHD5,HDHD5-AS1,ADA2,CECR2,SLC25A18,SLC25A18,LOC101929372,ATP6V1E1,BCL2L13,BID,BID,MIR3198-1,MICAL3,MICAL3,MIR648,PEX26,TUBA8,USP18,GGT3P,DGCR6,PRODH,DGCR2,DGCR2,DGCR11,ESS2,TSSK2,ESS2,GSC2,SLC25A1,CLTCL1,HIRA,MRPL40,C22orf39,UFD1,CDC45,CLDN5,SEPT5,SEPT5,SEPT5-GP1BB,SEPT5-GP1BB,GP1BB,TBX1,GNB1L,GNB1L,RTL10,TXNRD2,TXNRD2,COMT,COMT,COMT,MIR4761,ARVCF,TANGO2,MIR185,TANGO2 | -0.656557 | 143.307 | 583 | 343.975 |
| 3 | 4 | 1.86E+08 | 189068463 | CCDC110,CCDC110,LOC105377590,LOC105377590,PDLIM3,SORBS2,TLR3,FAM149A,CYP4V2,KLKB1,F11,F11,F11-AS1,F11-AS1,MTNR1A,FAT1,ZFP42,TRIML2,TRIML1 | -0.657389 | 150.556 | 245 | 158.727 |
| 3 | 12 | 1.19E+08 | 118839696 | TAOK3,SUDS3 | -0.661916 | 152.106 | 27 | 15.1182 |
| 3 | 1 | 17273204 | 25598565 | CROCC,MFAP2,ATP13A2,SDHB,PADI2,PADI1,PADI3,PADI3,MIR3972,PADI4,PADI6,RCC2,ARHGEF10L,ACTL8,IGSF21,KLHDC7A,PAX7,TAS1R2,ALDH4A1,ALDH4A1,MIR4695,IFFO2,UBR4,LOC101927895,EMC1,EMC1,MRTO4,AKR7L,AKR7A3,AKR7A2,PQLC2,CAPZB,MINOS1,MINOS1-NBL1,MINOS1,MINOS1-NBL1,RPS14P3,MINOS1-NBL1,NBL1,HTR6,TMCO4,RNF186,OTUD3,PLA2G2E,PLA2G2A,PLA2G5,PLA2G2D,PLA2G2F,PLA2G2C,UBXN10,VWA5B1,CAMK2N1,MUL1,FAM43B,CDA,PINK1,MIR6084,PINK1,PINK1,PINK1-AS,DDOST,KIF17,SH2D5,HP1BP3,EIF4G3,EIF4G3,MIR1256,ECE1,ECE1,LOC100506801,NBPF3,ALPL,RAP1GAP,USP48,LDLRAD2,LDLRAD2,HSPG2,HSPG2,CELA3B,CELA3A,CDC42,WNT4,MIR4418,ZBTB40,EPHA8,C1QA,C1QC,C1QB,EPHB2,MIR4684,EPHB2,EPHB2,MIR4253,LACTBL1,TEX46,KDM1A,KDM1A,MIR3115,LUZP1,HTR1D,HNRNPR,ZNF436,ZNF436,ZNF436-AS1,ZNF436-AS1,TCEA3,ASAP3,E2F2,E2F2,LOC101928163,ID3,MDS2,RPL11,ELOA,PITHD1,LYPLA2,GALE,HMGCL,FUCA1,CNR2,MIR378F,PNRC2,SRSF10,MYOM3,IL22RA1,IFNLR1,GRHL3,GRHL3,STPG1,STPG1,NIPAL3,RCAN3,NCMAP,SRRM1,CLIC4,RUNX3,RUNX3,MIR6731,MIR4425,SYF2,RSRP1 | -0.673678 | 137.604 | 1544 | 935.42 |
| 3 | 16 | 54281460 | 58750794 | IRX3,IRX5,IRX6,MMP2,LPCAT2,LPCAT2,CAPNS2,SLC6A2,CES1P1,CES1,CES5A,GNAO1,GNAO1,DKFZP434H168,GNAO1,MIR3935,AMFR,NUDT21,OGFOD1,BBS2,MT4,MT3,MT2A,MT1E,MT1M,MT1A,MT1B,MT1F,MT1G,MT1H,MT1IP,MT1X,NUP93,MIR138-2,SLC12A3,SLC12A3,MIR6863,HERPUD1,CETP,NLRC5,CPNE2,FAM192A,RSPRY1,ARL2BP,PLLP,CCL22,CX3CL1,CCL17,CIAPIN1,CIAPIN1,COQ9,COQ9,POLR2C,DOK4,CCDC102A,ADGRG5,ADGRG1,ADGRG3,DRC7,KATNB1,KIFC3,KIFC3,MIR6772,LOC388282,CNGB1,TEPP,ZNF319,USB1,MMP15,CFAP20,CSNK2A2,CCDC113,CCDC113,PRSS54,PRSS54,GINS3,NDRG4,SETD6,CNOT1,CNOT1,SNORA46,CNOT1,SNORA50A,SLC38A7,GOT2 | -0.673832 | 150.674 | 861 | 525.878 |
| 3 | 2 | 86335408 | 86406647 | PTCD3,PTCD3,SNORD94,IMMT | -0.676097 | 158.131 | 44 | 25.25 |

| | | | | | | | | |
|---|---|---|---|---|---|---|---|---|
| 3 | 6 | 42559843 | 42656079 | UBR2 | -0.676681 | 196.924 | 42 | 25.6297 |
| 3 | 11 | 92881756 | 93141608 | SLC36A4,DEUP1 | -0.682317 | 151.003 | 30 | 17.2754 |
| 3 | 15 | 77240775 | 79168994 | RCN2,PSTPIP1,TSPAN3,PEAK1,HMG20A,LINGO1,LINGO1,LINGO1-AS1,LOC645752,TBC1D2B,SH2D7,CIB2,IDH3A,ACSBG1,DNAJA4,WDR61,CRABP1,IREB2,HYKK,PSMA4,CHRNA5,CHRNA5,CHRNA3,CHRNA3,CHRNB4,LOC646938,ADAMTS7,MORF4L1 | -0.683189 | 133.734 | 305 | 179.204 |
| 3 | 1 | 1.85E+08 | 185259257 | RNF2,TRMT1L,SWT1 | -0.685792 | 202.426 | 41 | 23.4268 |
| 3 | 1 | 6885142 | 16248878 | CAMTA1,VAMP3,PER3,UTS2,TNFRSF9,PARK7,ERRFI1,SLC45A1,RERE,ENO1,ENO1,MIR6728,CA6,SLC2A7,SLC2A5,GPR157,MIR34AHG,MIR34A,MIR34AHG,LINC01759,H6PD,SPSB1,SLC25A33,TMEM201,PIK3CD,PIK3CD-AS1,PIK3CD,CLSTN1,CTNNBIP1,LZIC,NMNAT1,MIR5697,NMNAT1,RBP7,UBE4B,KIF1B,PGD,CENPS,CENPS-CORT,CENPS-CORT,CORT,DFFA,PEX14,CASZ1,C1orf127,TARDBP,MASP2,SRM,EXOSC10,EXOSC10,LOC105376736,MTOR,MTOR,MTOR-AS1,MTOR,ANGPTL7,UBIAD1,DISP3,FBXO2,FBXO2,FBXO44,FBXO44,FBXO6,MAD2L2,DRAXIN,AGTRAP,C1orf167,C1orf167,LOC102724659,C1orf167,MTHFR,MTHFR,CLCN6,NPPA-AS1,NPPA,NPPB,KIAA2013,PLOD1,MFN2,MIIP,MIIP,MIR6729,TNFRSF8,MIR7846,TNFRSF1B,TNFRSF1B,TNFRSF1B,MIR4632,VPS13D,VPS13D,SNORA59B,SNORA59A,DHRS3,AADACL4,AADACL3,C1orf158,PRAMEF12,PRAMEF1,PRAMEF11,HNRNPCL4,HNRNPCL3,HNRNPCL1,PRAMEF2,PRAMEF18,HNRNPCL2,PRAMEF18,PRAMEF22,PRAMEF18,PRAMEF22,PRAMEF19,PRAMEF18,PRAMEF20,PRAMEF18,PRAMEF19,PRAMEF20,LRRC38,PDPN,PRDM2,KAZN,KAZN,TMEM51-AS1,TMEM51,C1orf195,TMEM51,FHAD1,FHAD1,LOC101927417,EFHD2,CTRC,CELA2A,CELA2B,CELA2B,CASP9,CASP9,DNAJC16,AGMAT,DDI2,DDI2,RSC1A1,RSC1A1,PLEKHM2,SLC25A34,SLC25A34-AS1,TMEM82,FBLIM1,UQCRHL,FLJ37453,SPEN,SPEN,MIR5096,SPEN | -0.685874 | 142.163 | 1412 | 855.251 |
| 3 | 2 | 24550861 | 26453245 | ITSN2,NCOA1,PTRHD1,PTRHD1,CENPO,CENPO,CENPO,ADCY3,ADCY3,DNAJC27,EFR3B,POMC,DNMT3A,DNMT3A,MIR1301,DTNB,ASXL2,KIF3C,RAB10,GAREM2,HADHA | -0.688693 | 135.562 | 262 | 156.763 |
| 3 | 15 | 44029086 | 44614547 | CATSPER2P1,PDIA3,ELL3,ELL3,SERF2,SERF2,SERF2-C15ORF63,SERF2,SERF2-C15ORF63,MIR1282,SERF2,SERF2-C15ORF63,SERINC4,SERF2-C15ORF63,SERINC4,SERF2-C15ORF63,HYPK,MFAP1,WDR76,FRMD5,CASC4 | -0.689058 | 154.106 | 103 | 61.5937 |
| 3 | 16 | 81061785 | 85144152 | CENPN,ATMIN,C16orf46,GCSH,PKD1L2,BCO1,GAN,MIR4720,CMIP,PLCG2,SDR42E1,HSD17B2,MPHOSPH6,CDH13,CDH13,MIR3182,HSBP1,MLYCD,OSGIN1,NECAB2,SLC38A8,MBTPS1,HSDL1,DNAAF1,DNAAF1,TAF1C,TAF1C,ADAD2,ADAD2,LOC654780,KCNG4,WFDC1,ATP2C2,ATP2C2,ATP2C2-AS1,TLDC1,COTL1,KLHL36,USP10,CRISPLD2,ZDHHC7,KIAA0513,FAM92B | -0.689406 | 138.306 | 545 | 327.392 |

| | | | | | | | | |
|---|---|---|---|---|---|---|---|---|
| 3 | 15 | 49936316 | 51242381 | ATP8B4,SLC27A2,HDC,GABPB1,GABPB1,GABPB1-IT1,GABPB1-AS1,MIR4712,USP8,USP8,USP50,USP50,TRPM7,SPPL2A,AP4E1 | -0.689695 | 170.919 | 208 | 117.856 |
| 3 | 18 | 72022373 | 76870591 | FAM69C,CNDP2,CNDP1,LINC00909,ZNF407,ZADH2,TSHZ1,SMIM21,ZNF516,C18orf65,ZNF236,MBP,GALR1,SALL3,ATP9B | -0.698897 | 132.874 | 236 | 146.953 |
| 3 | 1 | 3394471 | 6614424 | ARHGEF16,MEGF6,MEGF6,MIR551A,TPRG1L,WRAP73,TP73,TP73-AS1,CCDC27,SMIM1,LRRC47,CEP104,DFFB,C1orf174,AJAP1,MIR4417,MIR4689,NPHP4,NPHP4,KCNAB2,CHD5,RPL22,RNF207,ICMT,HES3,GPR153,ACOT7,HES2,ESPN,ESPN,MIR4252,TNFRSF25,TNFRSF25,PLEKHG5,PLEKHG5,NOL9 | -0.702802 | 128.612 | 422 | 255.707 |
| 3 | 2 | 1.8E+08 | 191175850 | SESTD1,ZNF385B,ZNF385B,MIR1258,CWC22,UBE2E3,LINC01934,MIR4437,ITGA4,ITGA4,CERKL,CERKL,NEUROD1,SSFA2,PPP1R1C,PDE1A,DNAJC10,FRZB,NCKAP1,DUSP19,NUP35,MIR548AE1,ZNF804A,LOC101927196,FSIP2,FSIP2,FSIP2,FSIP2-AS1,ZC3H15,ITGAV,FAM171B,ZSWIM2,CALCRL,TFPI,GULP1,DIRC1,COL3A1,COL3A1,MIR1245A,MIR1245B,COL5A2,COL5A2,MIR3129,WDR75,SLC40A1,ASDURF,ASNSD1,ASNSD1,ANKAR,ANKAR,OSGEPL1,OSGEPL1,ORMDL1,PMS1,MSTN,C2orf88,HIBCH | -0.708342 | 155.101 | 791 | 479.514 |
| 3 | 8 | 8869017 | 15555800 | ERI1,ERI1,MIR4660,PPP1R3B,TNKS,TNKS,MIR597,MIR124-1,MSRA,PRSS55,RP1L1,MIR4286,C8orf74,SOX7,SOX7,LOC102723313,LOC102723313,PINX1,PINX1,PINX1,MIR1322,XKR6,XKR6,MIR598,XKR6,LOC101929269,MTMR9,SLC35G5,TDH,LOC100129129,FAM167A-AS1,FAM167A,FAM167A,BLK,GATA4,C8orf49,NEIL2,FDFT1,CTSB,DEFB136,DEFB135,DEFB134,LOC729732,MIR5692A1,MIR5692A2,LONRF1,LONRF1,MIR3926-1,MIR3926-2,KIAA1456,DLC1,DLC1,C8orf48,SGCZ,SGCZ,MIR383,TUSC3 | -0.709743 | 125.966 | 399 | 240.899 |
| 3 | 16 | 68349789 | 70843922 | PRMT7,SMPD3,ZFP90,CDH3,CDH1,TANGO6,HAS3,HAS3,CHTF8,CHTF8,CHTF8,UTP4,UTP4,SNTB2,VPS4A,PDF,COG8,COG8,COG8,NIP7,NIP7,TMED6,TERF2,CYB5B,MIR1538,NFAT5,NQO1,NOB1,WWP2,WWP2,MIR140,CLEC18A,PDXDC2P-NPIPB14P,PDXDC2P-NPIPB14P,MIR1972-1,MIR1972-2,PDPR,PDPR,LOC400541,CLEC18C,EXOSC6,AARS,DDX19B,DDX19B,LOC100506083,DDX19A,ST3GAL2,FUK,COG4,SF3B3,SF3B3,SNORD111B,SF3B3,SNORD111,IL34,MTSS1L,VAC14,VAC14,VAC14-AS1,HYDIN | -0.711604 | 143.657 | 573 | 339.632 |
| 3 | 8 | 1.42E+08 | 142266863 | PTK2,DENND3,SLC45A4,SLC45A4,LOC105375787,LOC105375787 | -0.715016 | 136.823 | 56 | 34.2539 |

| | | | | | | | | |
|---|---|---|---|---|---|---|---|---|
| 3 | 15 | 79189824 | 99800155 | CTSH,RASGRF1,RASGRF1,LOC100129540,ANKRD34C-AS1,MIR184,ANKRD34C,TMED3,KIAA1024,MTHFS,ST20-MTHFS,ST20-MTHFS,ST20,ST20-MTHFS,ST20,ST20-AS1,ST20,ST20-AS1,BCL2A1,ZFAND6,FAH,ARNT2,ARNT2,LOC101929586,ARNT2,MIR5572,ABHD17C,CEMIP,MIR549A,CEMIP,CEMIP,MESD,MESD,MIR4514,TLNRD1,CFAP161,IL16,STARD5,TMC3-AS1,TMC3,MEX3B,LINC01583,EFL1,SAXO2,ADAMTS7P1,GOLGA6L10,UBE2Q2P2,GOLGA6L10,GOLGA6L17P,GOLGA6L9,RPS17,GOLGA6L10,GOLGA6L17P,GOLGA6L9,GOLGA6L10,GOLGA6L17P,GOLGA6L9,ADAMTS7P1,GOLGA6L17P,GOLGA6L9,UBE2Q2P2,RPS17,CPEB1,CPEB1,CPEB1-AS1,CPEB1-AS1,AP3B2,AP3B2,ACTG1P17,SNHG21,FSD2,SCARNA15,FSD2,WHAMM,HOMER2,FAM103A1,C15orf40,C15orf40,BTBD1,BTBD1,MIR4515,TM6SF1,HDGFL3,BNC1,SH3GL3,ADAMTSL3,EFL1P1,LOC440300,GOLGA2P7,ZSCAN2,SCAND2P,WDR73,NMB,SEC11A,ZNF592,ALPK3,SLC28A1,PDE8A,AKAP13,KLHL25,KLHL25,MIR1276,MIR548AP,AGBL1,AGBL1,LOC102724452,NTRK3,NTRK3,NTRK3-AS1,MRPL46,MRPS11,DET1,MIR1179,MIR7-2,MIR3529,AEN,ISG20,ACAN,HAPLN3,MFGE8,ABHD2,RLBP1,FANCI,FANCI,POLG,POLG,POLG,MIR6766,MIR9-3HG,MIR9-3,RHCG,TICRR,KIF7,PLIN1,PEX11A,WDR93,MESP1,MESP2,ANPEP,AP3S2,C15orf38-AP3S2,AP3S2,C15orf38-AP3S2,MIR5094,AP3S2,C15orf38-AP3S2,MIR5009,C15orf38-AP3S2,ARPIN,ZNF710,MIR3174,ZNF710,ZNF710,ZNF710-AS1,IDH2,IDH2,IDH2-DT,SEMA4B,CIB1,CIB1,GDPGP1,CIB1,TTLL13P,CIB1,NGRN,NGRN,GABARAPL3,ZNF774,IQGAP1,CRTC3,CRTC3, | -0.715497 | 137.422 | 1893 | 1159.17 |
| 3 | 16 | 64981519 | 66789025 | CDH11,LINC00922,CDH5,BEAN1,TK2,CKLF,CKLF-CMTM1,CKLF-CMTM1,CMTM1,CMTM2,CMTM3,CMTM4,DYNC1LI2,TERB1 | -0.716305 | 129.934 | 118 | 71.0379 |
| 3 | X | 9000484 | 13726274 | FAM9B,TBL1X,GPR143,SHROOM2,CLDN34,WWC3,CLCN4,MID1,HCCS,ARHGAP6,ARHGAP6,AMELX,ARHGAP6,MIR548AX,MSL3,FRMPD4,PRPS2,TLR7,TLR8-AS1,TLR8,TMSB4X,FAM9C,GS1-600G8.3,ATXN3L,EGFL6,TCEANC | -0.720344 | 143.354 | 325 | 199.777 |
| 3 | X | 1.18E+08 | 122770028 | DOCK11,IL13RA1,ZCCHC12,LONRF3,KIAA1210,PGRMC1,SLC25A43,SLC25A5-AS1,SLC25A5,SLC25A5,CXorf56,UBE2A,NKRF,SEPT6,SEPT6,MIR766,SOWAHD,RPL39,RPL39,SNORA69,UPF3B,RNF113A,NDUFA1,AKAP14,NKAP,RHOXF1-AS1,RHOXF2B,RHOXF2,RHOXF1-AS1,RHOXF1,RHOXF2,RHOXF2B,ZBTB33,TMEM255A,ATP1B4,LAMP2,CUL4B,MCTS1,C1GALT1C1,CT47B1,GLUD2,MIR3672,GRIA3,THOC2 | -0.724613 | 132.575 | 395 | 234.187 |

| | | | | | | | | |
|---|---|---|---|---|---|---|---|---|
| 3 | X | 96330837 | 114357828 | DIAPH2,PCDH19,TNMD,TSPAN6,SRPX2,SYTL4,CSTF2,NOX1,XKRX,ARL13A,TRMT2B,TMEM35A,CENPI,DRP2,TAF7L,TIMM8A,BTK,RPL36A,RPL36A-HNRNPH2,RPL36A-HNRNPH2,GLA,RPL36A-HNRNPH2,HNRNPH2,ARMCX4,ARMCX1,ARMCX6,ARMCX3,ARMCX2,NXF5,ZMAT1,TCEAL2,TCEAL6,BEX5,TCP11X2,NXF2B,NXF2,TMSB15A,ARMCX5,ARMCX5-GPRASP2,ARMCX5-GPRASP2,GPRASP1,ARMCX5-GPRASP2,GPRASP2,ARMCX5-GPRASP2,BHLHB9,ARMCX5-GPRASP2,LINC00630,LINC00630,LINC00630,RAB40AL,BEX1,NXF3,BEX4,TCEAL8,TCEAL5,BEX2,TCEAL7,TCEAL9,BEX3,RAB40A,TCEAL4,TCEAL3,TCEAL1,MORF4L2,GLRA4,GLRA4,TMEM31,RAB9B,PLP1,RAB9B,TMSB15B,H2BFXP,LOC100101478,H2BFWT,H2BFM,SLC25A53,SLC25A53,ZCCHC18,FAM199X,ESX1,IL1RAPL2,IL1RAPL2,TEX13A,NRK,SERPINA7,MUM1L1,CXorf57,RNF128,TBC1D8B,RIPPLY1,CLDN2,CLDN2,MORC4,RBM41,NUP62CL,PIH1D3,FRMPD3-AS1,FRMPD3,FRMPD3,PRPS1,TSC22D3,NCBP2L,MID2,MID2,LOC101928335,TEX13B,VSIG1,PSMD10,ATG4A,COL4A6,COL4A5,IRS4,GUCY2F,NXT2,KCNE5,ACSL4,TMEM164,TMEM164,MIR652,TMEM164,MIR3978,AMMECR1,AMMECR1,SNORD96B,RTL9,TDGF1P3,CHRDL1,PAK3,CAPN6,DCX,ALG13,TRPC5,TRPC5,TRPC5OS,RTL4,LHFPL1,AMOT,AMOT,MIR4329,HTR2C,SNORA35,HTR2C,MIR764,HTR2C,MIR1912,HTR2C,MIR1264,HTR2C,MIR1298,HTR2C,HTR2C,MIR1911,HTR2C,MIR448,IL13RA2,LRCH2 | -0.724682 | 131.272 | 1404 | 838.271 |
| 3 | 15 | 1E+08 | 102520892 | MEF2A,LYSMD4,DNM1P46,LOC400464,ADAMTS17,SPATA41,CERS3-AS1,CERS3,CERS3,LINS1,ASB7,ALDH1A3,ALDH1A3,LOC101927751,LRRK1,CHSY1,SELENOS,SNRPA1,PCSK6,PCSK6,LOC100507472,TM2D3,TARSL2,LOC100128108,OR4F6,OR4F15,OR4F13P,OR4F4 | -0.726206 | 140.856 | 304 | 176.228 |
| 3 | 9 | 95048771 | 95272410 | IARS,IARS,MIR3651,SNORA84,NOL8,CENPP,CENPP,OGN,CENPP,OMD,CENPP,ASPN,CENPP,ECM2 | -0.727055 | 192.264 | 73 | 42.8621 |
| 3 | 8 | 30608848 | 31005018 | UBXN8,PPP2CB,TEX15,PURG,WRN | -0.729868 | 177.67 | 103 | 64.1488 |
| 3 | 10 | 95452311 | 96087718 | FRA10AC1,LGI1,SLC35G1,PIPSL,PLCE1,PLCE1,PLCE1-AS2,PLCE1,PLCE1-AS1 | -0.730947 | 153.473 | 87 | 53.5534 |
| 3 | 8 | 95738471 | 95905243 | DPY19L4,INTS8,INTS8,CCNE2,CCNE2 | -0.735954 | 202.605 | 61 | 33.7345 |

| | | | | | | | | |
|---|---|---|---|---|---|---|---|---|
| 3 | 10 | 98369486 | 115312335 | PIK3AP1,MIR607,LCOR,SLIT1,SLIT1,ARHGAP19-SLIT1,ARHGAP19-SLIT1,ARHGAP19,FRAT1,FRAT2,RRP12,PGAM1,EXOSC1,ZDHHC16,MMS19,UBTD1,ANKRD2,HOGA1,HOGA1,C10orf62,MORN4,PI4K2A,AVPI1,MARVELD1,ZFYVE27,SFRP5,GOLGA7B,GOLGA7B,CRTAC1,CRTAC1,R3HCC1L,LOXL4,PYROXD2,PYROXD2,MIR1287,HPS1,HPS1,MIR4685,HPSE2,CNNM1,GOT1,NKX2-3,SLC25A28,ENTPD7,ENTPD7,COX15,COX15,COX15,CUTC,CUTC,ABCC2,DNMBP,DNMBP,DNMBP-AS1,CPN1,ERLIN1,CHUK,CWF19L1,CWF19L1,SNORA12,BLOC1S2,PKD2L1,SCD,WNT8B,SEC31B,NDUFB8,HIF1AN,PAX2,SLF2,SEMA4G,SEMA4G,MIR608,SEMA4G,MRPL43,MRPL43,TWNK,LZTS2,LZTS2,PDZD7,PDZD7,SFXN3,KAZALD1,TLX1NB,TLX1NB,TLX1,LBX1,LBX1-AS1,BTRC,POLL,POLL,DPCD,DPCD,DPCD,MIR3158-1,MIR3158-2,FBXW4,FGF8,NPM3,MGEA5,KCNIP2-AS1,KCNIP2,KCNIP2,C10orf76,HPS6,LDB1,PPRC1,NOLC1,ELOVL3,PITX3,GBF1,NFKB2,PSD,FBXL15,CUEDC2,MIR146B,RPARP-AS1,C10orf95,RPARP-AS1,MFSD13A,ACTR1A,SUFU,TRIM8,ARL3,SFXN2,WBP1L,CYP17A1,BORCS7,BORCS7-ASMT,BORCS7-ASMT,AS3MT,CNNM2,NT5C2,RPEL1,INA,PCGF6,TAF5,ATP5MD,ATP5MD,MIR1307,PDCD11,CALHM2,CALHM1,CALHM3,NEURL1-AS1,NEURL1,NEURL1,SH3PXD2A,STN1,SLK,COL17A1,COL17A1,MIR936,SFR1,CFAP43,CFAP43,MIR609,GSTO1,MIR4482,GSTO2,ITPRIP,CFAP58,SORCS3,SORCS1,XPNPEP1,ADD3-AS1,ADD3,MXI1,SMNDC1,DUSP5,SMC3,RBM20,PDCD4,PDCD4,MIR4680,BBIP1,SHOC2,RPL13AP6,SHOC2,ADRA2A,GPAM,TECTB,ACSL5,ZDHHC6,ZDHHC6,VTI1A,VTI1A,VTI1A,MIR4295,TCF7L2 | -0.73746 | 136.116 | 2042 | 1249.4 |
| 3 | 8 | 1.36E+08 | 141745591 | MIR30B,MIR30D,LINC01591,KHDRBS3,FAM135B,COL22A1,KCNK9,TRAPPC9,CHRAC1,AGO2,PTK2 | -0.739767 | 110.874 | 265 | 154.7 |
| 3 | X | 53245233 | 55478014 | KDM5C,IQSEC2,SMC1A,SMC1A,MIR6857,RIBC1,HSD17B10,HUWE1,HUWE1,MIR98,HUWE1,MIRLET7F2,PHF8,FAM120C,WNK3,TSR2,TSR2,FGD1,FGD1,GNL3L,ITIH6,MAGED2,MAGED2,SNORA11,TRO,PFKFB1,APEX2,ALAS2,PAGE2B,PAGE2,FAM104B,MTRNR2L10,PAGE5,PAGE3,MIR4536-1,MIR4536-2 | -0.740615 | 129.029 | 467 | 285.669 |
| 3 | 15 | 43350536 | 43739665 | UBR1,TMEM62,CCNDBP1,EPB42,TGM5,TGM7,LCMT2,LCMT2,ADAL,ADAL,ZSCAN29,TUBGCP4,TUBGCP4,TP53BP1,TP53BP1 | -0.745955 | 151.798 | 158 | 99.1094 |
| 3 | 3 | 57232204 | 57857478 | HESX1,APPL1,APPL1,ASB14,APPL1,ASB14,LOC105377102,ASB14,LOC105377102,ASB14,DNAH12,PDE12,PDE12,ARF4,PDE12,DENND6A,DENND6A,SLMAP | -0.747426 | 186.487 | 156 | 92.311 |
| 3 | X | 60500 | 8758794 | PLCXD1,GTPBP6,PPP2R3B,SHOX,CRLF2,CSF2RA,IL3RA,SLC25A6,ASMTL-AS1,ASMTL,ASMTL,P2RY8,AKAP17A,AKAP17A,ASMT,ASMT,DHRSX,DHRSX,ZBED1,CD99,XG,XG,XGY2,GYG2,ARSD,ARSD-AS1,ARSD,ARSE,ARSH,ARSF,LINC01546,MXRA5,PRKX,LOC389906,FAM239B,NLGN4X,MIR4770,PUDP,PUDP,STS,MIR4767,PUDP,STS,STS,PNPLA4,MIR651,VCX2,ANOS1 | -0.748647 | 104.483 | 460 | 240.758 |

| | | | | | | | | |
|---|---|---|---|---|---|---|---|---|
| 3 | 16 | 71405969 | 81010118 | CALB2,ZNF23,ZNF19,CHST4,TAT-AS1,TAT,TAT,MARVELD3,PHLPP2,PHLPP2,SNORA70D,AP1G1,AP1G1,SNORD71,ATXN1L,ZNF821,IST1,IST1,PKD1L3,PKD1L3,DHODH,HP,HPR,TXNL4B,DHX38,PMFBP1,ZFHX3,HCCAT5,C16orf47,PSMD7,LOC283922,NPIPB15,CLEC18B,GLG1,RFWD3,MLKL,FA2H,WDR59,ZNRF1,LDHD,ZFP1,CTRB2,CTRB1,BCAR1,CFDP1,TMEM170A,CHST6,CHST5,TMEM231,GABARAPL2,ADAT1,KARS,TERF2IP,DUXB,CNTNAP4,LINC02125,MIR4719,MON1B,MON1B,SYCE1L,SYCE1L,ADAMTS18,NUDT7,VAT1L,CLEC3A,WWOX,MAF,LOC102724084,DYNLRB2,CDYL2,CMC2 | -0.752759 | 132.104 | 866 | 526.82 |
| 3 | 8 | 17143858 | 27690717 | VPS37A,MTMR7,SLC7A2,PDGFRL,MTUS1,MTUS1,MIR548V,FGL1,PCM1,ASAH1,NAT1,NAT2,PSD3,SH2D4A,CSGALNACT1,INTS10,LPL,SLC18A1,ATP6V1B2,LZTS1,GFRA2,DOK2,XPO7,NPM2,FGF17,DMTN,FAM160B2,NUDT18,HR,REEP4,LGI3,SFTPC,BMP1,PHYHIP,MIR320A,POLR3D,POLR3D,PIWIL2,SLC39A14,PPP3CC,SORBS3,PDLIM2,C8orf58,CCAR2,BIN3,EGR3,PEBP4,RHOBTB2,TNFRSF10B,TNFRSF10B,LOC286059,LOC254896,TNFRSF10C,TNFRSF10C,TNFRSF10D,TNFRSF10A,CHMP7,R3HCC1,LOXL2,LOXL2,LOC100507156,ENTPD4,SLC25A37,NKX3-1,NKX2-6,STC1,ADAM28,ADAM28,LOC101929294,LOC101929294,ADAMDEC1,LOC101929294,ADAM7,LOC101929294,ADAM7,LOC101929315,LOC101929294,NEFM,NEFL,NEFL,MIR6841,DOCK5,DOCK5,MIR6876,GNRH1,KCTD9,CDCA2,EBF2,PPP2R2A,BNIP3L,PNMA2,DPYSL2,ADRA1A,STMN4,TRIM35,PTK2B,CHRNA2,EPHX2,CLU,CLU,MIR6843,SCARA3,MIR3622B,MIR3622A,CCDC25,ESCO2,PBK | -0.754707 | 137.818 | 1269 | 780.054 |
| 3 | 2 | 26508314 | 26688571 | HADHB,ADGRF3,ADGRF3,SELENOI,SELENOI,DRC1,OTOF | -0.755593 | 130.008 | 67 | 41.2925 |
| 3 | 10 | 1.17E+08 | 118099717 | ATRNL1,GFRA1,CCDC172 | -0.756407 | 92.446 | 29 | 16.3395 |
| 3 | X | 86635412 | 96166812 | KLHL4,CPXCR1,TGIF2LX,PABPC5,PCDH11X,NAP1L3,FAM133A,MIR548M,DIAPH2,DIAPH2,RPA4 | -0.756493 | 104.557 | 146 | 86.4834 |
| 3 | 6 | 15368902 | 18258633 | JARID2,DTNBP1,MYLIP,MYLIP,MIR4639,GMPR,ATXN1,ATXN1,LOC101928433,STMND1,RBM24,CAP2,CAP2,LOC101928491,FAM8A1,NUP153,NUP153,LOC105374952,KIF13A,NHLRC1,TPMT,KDM1B,DEK | -0.763163 | 137.243 | 282 | 167.014 |

| | | | | | | | | |
|---|---|---|---|---|---|---|---|---|
| 3 | 6 | 60500 | 12750011 | LOC285766,DUSP22,IRF4,EXOC2,EXOC2,HUS1B,LINC01622,FOXQ1,FOXF2,FOXF2,MIR6720,FOXC1,GMDS,LINC01600,MYLK4,MYLK4,WRNIP1,WRNIP1,SERPINB1,MIR4645,LOC101927730,SERPINB9,SERPINB6,NQO2,RIPK1,BPHL,TUBB2A,TUBB2B,LOC100422781,PSMG4,SLC22A23,SLC22A23,LOC643327,PXDC1,FAM50B,PRPF4B,FAM217A,C6orf201,C6orf201,ECI2,ECI2,CDYL,RPP40,RPP40,LYRM4-AS1,LYRM4-AS1,LYRM4,PPP1R3G,LYRM4-AS1,LYRM4,LYRM4-AS1,LYRM4,MIR3691,LYRM4,FARS2,NRN1,F13A1,F13A1,MIR7853,MIR5683,LY86-AS1,LY86,LY86,RREB1,SSR1,CAGE1,RIOK1,DSP,SNRNP48,BMP6,BLOC1S5-TXNDC5,TXNDC5,BLOC1S5-TXNDC5,PIP5K1P1,BLOC1S5-TXNDC5,BLOC1S5,EEF1E1-BLOC1S5,EEF1E1-BLOC1S5,EEF1E1,SLC35B3,TFAP2A,TFAP2A,TFAP2A-AS2,TFAP2A,TFAP2A-AS1,LINC00518,MIR5689HG,MIR5689,GCNT2,C6orf52,PAK1IP1,TMEM14C,TMEM14B,MAK,GCM2,SYCP2L,SYCP2L,LOC101928191,ELOVL2,ELOVL2,ELOVL2-AS1,SMIM13,SMIM13,ERVFRD-1,NEDD9,TMEM170B,ADTRP,HIVEP1,EDN1,PHACTR1 | -0.768411 | 130.576 | 947 | 564.454 |
| 3 | 2 | 17963724 | 21266735 | MSGN1,KCNS3,RDH14,NT5C1B-RDH14,NT5C1B-RDH14,NT5C1B,MIR4757,OSR1,TTC32,WDR35,LOC101928222,MATN3,MATN3,LAPTM4A,SDC1,PUM2,RHOB,HS1BP3,GDF7,LDAH,APOB | -0.772964 | 137.1 | 248 | 154.141 |
| 3 | 2 | 14621363 | 15601492 | FAM84A,NBAS | -0.778287 | 130.802 | 57 | 35.1438 |
| 3 | 10 | 1.24E+08 | 131934107 | DMBT1,C10orf120,CUZD1,FAM24B-CUZD1,FAM24B-CUZD1,FAM24B,LOC399815,FAM24A,C10orf88,PSTK,IKZF5,ACADSB,HMX3,HMX2,BUB3,GPR26,CPXM2,CHST15,OAT,NKX1-2,LHPP,FAM53B,FAM53B,FAM53B-AS1,EEF1AKMT2,ABRAXAS2,ZRANB1,CTBP2,CTBP2,MIR4296,TEX36-AS1,TEX36,TEX36,EDRF1,EDRF1,EDRF1-AS1,MMP21,UROS,UROS,MIR4484,BCCIP,BCCIP,DHX32,DHX32,FANK1,ADAM12,C10orf90,LOC728158,C10orf90,DOCK1,DOCK1,FAM196A,NPS,FOXI2,CLRN3,PTPRE,PTPRE,AS-PTPRE,MKI67,MGMT,EBF3,EBF3,MIR4297,C10orf143,CTAGE7P | -0.780017 | 134.095 | 643 | 385.622 |
| 3 | 11 | 1.1E+08 | 112013780 | ARHGAP20,C11orf53,COLCA1,COLCA2,MIR4491,POU2AF1,BTG4,LOC728196,MIR34B,LOC728196,MIR34C,C11orf88,LAYN,SIK2,SIK2,PPP2R1B,PPP2R1B,ALG9,FDXACB1,C11orf1,CRYAB,CRYAB,HSPB2,HSPB2-C11orf52,HSPB2,HSPB2-C11orf52,HSPB2-C11orf52,HSPB2-C11orf52,C11orf52,DIXDC1,DLAT,DLAT,PIH1D2,PIH1D2,NKAPD1,TIMM8B,TIMM8B,SDHD,SDHD | -0.782511 | 134.086 | 247 | 148.354 |
| 3 | 1 | 31792571 | 31842458 | ZCCHC17,FABP3 | -0.783278 | 119.801 | 11 | 6.11742 |
| 3 | X | 30864666 | 31221550 | TAB3,FTHL17,DMD | -0.784811 | 117.338 | 29 | 17.9229 |

| | | | | | | | | |
|---|---|---|---|---|---|---|---|---|
| 3 | 17 | 12016569 | 16676861 | MAP2K4,MYOCD,MYOCD,LOC101928418,MYOCD,LOC100128006,LOC100128006,ARHGAP44,ARHGAP44,ARHGAP44,MIR1269B,ELAC2,HS3ST3A1,CDRT15P1,COX10,CDRT15,HS3ST3B1,PMP22,PMP22,MIR4731,TEKT3,CDRT4,TVP23C-CDRT4,TVP23C-CDRT4,TVP23C,CDRT1,TRIM16,ZNF286A,TBC1D26,CDRT15P2,MEIS3P1,ADORA2B,ZSWIM7,ZSWIM7,TTC19,TTC19,NCOR1,PIGL,PIGL,MIR1288,CENPV,UBB,TRPV2,LRRC75A-AS1,LRRC75A-AS1,SNORD49B,LRRC75A-AS1,SNORD49A,LRRC75A-AS1,SNORD65,LRRC75A-AS1,LRRC75A,LRRC75A,ZNF287,ZNF624,CCDC144A | -0.787522 | 135.876 | 424 | 247.199 |
| 3 | 4 | 52660617 | 56316420 | DCUN1D4,LRRC66,SGCB,SPATA18,USP46,DANCR,MIR4449,DANCR,SNORA26,ERVMER34-1,RASL11B,SCFD2,FIP1L1,LNX1,LNX1,LNX1-AS1,RPL21P44,CHIC2,GSX2,PDGFRA,KIT,KDR,SRD5A3,SRD5A3,SRD5A3-AS1,TMEM165,CLOCK | -0.790783 | 127.158 | 284 | 173.518 |
| 3 | 18 | 2785334 | 6824223 | SMCHD1,EMILIN2,LPIN2,LPIN2,LOC727896,MYOM1,MYL12A,MYL12A,LOC104968399,MYL12B,TGIF1,DLGAP1,DLGAP1,DLGAP1-AS1,DLGAP1,DLGAP1-AS2,DLGAP1,DLGAP1-AS3,AKAIN1,LINC00667,ZBTB14,EPB41L3,MIR3976,TMEM200C,L3MBTL4,L3MBTL4,MIR4317,LINC01387,LOC101927168 | -0.791308 | 130.73 | 265 | 154.475 |
| 3 | 17 | 19683791 | 22220573 | ULK2,AKAP10,SPECC1,CCDC144CP,LGALS9B,CDRT15L2,LOC100287072,CCDC144NL,CCDC144NL,CCDC144NL-AS1,USP22,DHRS7B,TMEM11,NATD1,MAP2K3,KCNJ12,KCNJ18,C17orf51,UBBP4,FAM27E5 | -0.79321 | 134.425 | 216 | 117.774 |
| 3 | 16 | 11858838 | 11991904 | ZC3H7A,BCAR4,RSL1D1,GSPT1 | -0.793378 | 156.419 | 47 | 27.8511 |
| 3 | X | 1.23E+08 | 138914461 | STAG2,SH2D1A,TENM1,TEX13C,DCAF12L2,DCAF12L1,PRR32,ACTRT1,SMARCA1,OCRL,APLN,XPNPEP2,SASH3,ZDHHC9,UTP14A,BCORL1,ELF4,AIFM1,RAB33A,ZNF280C,SLC25A14,GPR119,RBMX2,FAM45BP,ENOX2,ARHGAP36,IGSF1,OR13H1,STK26,FRMD7,RAP2C,RAP2C-AS1,MBNL3,MBNL3,HS6ST2,HS6ST2,HS6ST2-AS1,USP26,TFDP3,GPC4,GPC3,MIR363,MIR92A2,MIR19B2,MIR20B,MIR18B,MIR106A,CCDC160,PHF6,HPRT1,MIR450B,MIR450A1,MIR450A1,MIR450A2,MIR542,MIR503HG,MIR503,MIR503HG,MIR424,PLAC1,FAM122B,FAM122C,MOSPD1,SMIM10,RTL8B,RTL8C,RTL8A,SMIM10L2B-AS1,CT55,ZNF75D,ZNF449,INTS6L,CT45A10,SAGE1,MMGT1,SLC9A6,FHL1,MAP7D3,ADGRG4,BRS3,HTATSF1,VGLL1,VGLL1,MIR934,CD40LG,ARHGEF6,RBMX,RBMX,SNORD61,GPR101,ZIC3,FGF13,FGF13,MIR504,F9,MCF2,ATP11C | -0.794746 | 132.521 | 1048 | 630.247 |
| 3 | 2 | 11598303 | 13043698 | E2F6,GREB1,MIR4429,GREB1,NTSR2,LPIN1,LPIN1,MIR548S,MIR4262,MIR3681HG,MIR3681,TRIB2,TRIB2,MIR3125 | -0.798505 | 140.014 | 100 | 61.0897 |

| | | | | | | | | |
|---|---|---|---|---|---|---|---|---|
| 3 | 8 | 10500 | 6914130 | ZNF596,FAM87A,FBXO25,TDRP,ERICH1,DLGAP2,DLGAP2,DLGAP2-AS1,CLN8,MIR3674,MIR596,ARHGEF10,KBTBD11,MYOM2,CSMD1,MCPH1,MCPH1,ANGPT2,MCPH1,MCPH1-AS1,AGPAT5,AGPAT5,MIR4659A,MIR4659B,XKR5,XKR5,GS1-24F4.2,DEFB1,DEFA6,DEFA4,DEFA5 | -0.799785 | 113.744 | 388 | 226.555 |
| 3 | 4 | 1.86E+08 | 186357376 | CENPU,ACSL1,MIR3945HG,MIR3945,MIR4455,HELT,LINC02436,SLC25A4,CFAP97,SNX25,LRP2BP,ANKRD37,ANKRD37,UFSP2,UFSP2,C4orf47 | -0.800432 | 125.611 | 110 | 66.2345 |
| 3 | 3 | 1.85E+08 | 184711902 | VPS8 | -0.818053 | 167.586 | 53 | 29.9737 |
| 3 | 3 | 1.49E+08 | 148793821 | HLTF | -0.818799 | 167.712 | 21 | 12.9571 |
| 3 | 13 | 96684755 | 115109378 | UGGT2,HS6ST3,HS6ST3,MIR4501,OXGR1,MBNL2,RAP2A,IPO5,FARP1,RNF113B,FARP1,STK24,SLC15A1,DOCK9,UBAC2,UBAC2,GPR18,UBAC2,GPR183,UBAC2,FKSG29,UBAC2,MIR623,TM9SF2,CLYBL,CLYBL,MIR4306,CLYBL,LOC101927437,ZIC5,ZIC2,PCCA,PCCA,GGACT,GGACT,TMTC4,NALCN-AS1,NALCN,NALCN,ITGBL1,FGF14,FGF14,MIR2681,FGF14,MIR4705,TPP2,METTL21C,CCDC168,TEX30,KDELC1,BIVM,BIVM-ERCC5,BIVM-ERCC5,ERCC5,SLC10A2,DAOA-AS1,DAOA,EFNB2,ARGLU1,FAM155A,FAM155A,MIR1267,LIG4,ABHD13,TNFSF13B,MYO16,MYO16,MYO16-AS1,IRS2,COL4A1,COL4A2,COL4A2,COL4A2-AS2,COL4A2,COL4A2-AS1,RAB20,NAXD,CARS2,ING1,LINC00346,ANKRD10,LINC00431,ARHGEF7-AS2,ARHGEF7,ARHGEF7,TEX29,LINC02337,SOX1-OT,SOX1,SPACA7,TUBGCP3,ATP11AUN,ATP11A,MCF2L-AS1,MCF2L,F7,F10,F10,F10-AS1,PROZ,PCID2,PCID2,CUL4A,CUL4A,LAMP1,GRTP1,GRTP1,GRTP1-AS1,ADPRHL1,DCUN1D2,DCUN1D2,TMCO3,TMCO3,TFDP1,ATP4B,GRK1,LINC00552,TMEM255B,GAS6-AS1,GAS6,GAS6,LINC00452,RASA3,CDC16,CDC16,MIR548AR,MIR4502,UPF3A,CHAMP1 | -0.8248 | 129.096 | 1477 | 896.081 |
| 3 | 3 | 44284366 | 44449180 | TOPAZ1,TCAIM | -0.829701 | 143.812 | 44 | 27.1179 |
| 3 | X | 15873485 | 30683185 | GRPR,MAGEB17,CTPS2,CTPS2,MIR548AM,CTPS2,S100G,SYAP1,TXLNG,RBBP7,REPS2,NHS,NHS,MIR4768,SCML1,RAI2,BEND2,SCML2,CDKL5,CDKL5,RS1,RS1,PPEF1,PHKA2-AS1,PHKA2,PHKA2,ADGRG2,PDHA1,PDHA1,MAP3K15,MAP3K15,SH3KBP1,BCLAF3,MAP7D2,MAP7D2,MIR23C,EIF1AX,RPS6KA3,CNKSR2,KLHL34,SMPX,MBTPS2,MBTPS2,YY2,SMS,PHEX,PHEX,PHEX-AS1,PHEX,PTCHD1-AS,PTCHD1-AS,ZNF645,PTCHD1-AS,DDX53,PTCHD1,PRDX4,ACOT9,SAT1,APOO,CXorf58,KLHL15,EIF2S3,ZFX,SUPT20HL2,SUPT20HL1,PDK3,PCYT1B,POLA1,POLA1,SCARNA23,ARX,MAGEB18,MAGEB6,MAGEB5,VENTXP1,PPP4R3C,DCAF8L2,MAGEB10,DCAF8L1,IL1RAPL1,IL1RAPL1,MIR4666B,MAGEB2,MAGEB3,MAGEB4,MAGEB1,NR0B1,CXorf21,GK | -0.839721 | 125.776 | 978 | 582.877 |

| | | | | | | | | |
|---|---|---|---|---|---|---|---|---|
| 3 | 17 | 9395049 | 11924355 | STX8,CFAP52,USP43,DHRS7C,GSG1L2,GLP2R,RCVRN,GAS7,MYH13,MYHAS,MYH8,MYHAS,MYH4,MYHAS,MYH1,MYHAS,MYH2,MYH3,SCO1,ADPRM,TMEM220,TMEM220,TMEM220-AS1,PIRT,SHISA6,DNAH9,ZNF18,MAP2K4 | -0.847114 | 137.187 | 511 | 306.927 |
| 3 | 1 | 92428272 | 94009780 | BRDT,EPHX4,BTBD8,KIAA1107,C1orf146,GLMN,RPAP2,GFI1,EVI5,RPL5,RPL5,FAM69A,RPL5,FAM69A,SNORD21,RPL5,FAM69A,SNORA66,FAM69A,MTF2,TMED5,TMED5,CCDC18,CCDC18,DR1,FNBP1L | -0.850257 | 145.337 | 239 | 138.525 |
| 3 | 12 | 1.23E+08 | 123110903 | ZCCHC8,RSRC2,KNTC1 | -0.853685 | 187.616 | 88 | 52.5926 |
| 3 | 10 | 94716040 | 95421901 | EXOC6,CYP26C1,CYP26A1,MYOF,CEP55,FFAR4,RBP4,PDE6C | -0.856004 | 137.411 | 135 | 83.7484 |
| 3 | 16 | 31580677 | 32858494 | YBX3P1,ZNF720,ZNF267 | -0.859506 | 124.773 | 59 | 30.0713 |
| 3 | 12 | 1.01E+08 | 101016189 | SCYL2,SLC17A8,NR1H4,GAS2L3 | -0.859898 | 140.543 | 44 | 26.5068 |
| 3 | 1 | 2.26E+08 | 226488992 | LIN9 | -0.86548 | 127.5 | 15 | 8.21874 |
| 3 | 8 | 1.29E+08 | 135524883 | CCDC26,MIR3686,GSDMC,FAM49B,FAM49B,MIR5194,ASAP1,ADCY8,EFR3A,OC90,HHLA1,KCNQ3,LRRC6,TMEM71,PHF20L1,TG,TG,SLA,WISP1,NDRG1,ST3GAL1,ZFAT | -0.869055 | 114.927 | 414 | 242.872 |
| 3 | 3 | 1.89E+08 | 197961930 | TPRG1,TPRG1,TPRG1-AS2,TP63,TP63,MIR944,P3H2,CLDN1,CLDN16,TMEM207,IL1RAP,GMNC,OSTN,OSTN,OSTN-AS1,UTS2B,UTS2B,CCDC50,CCDC50,LINCR-0002,PYDC2,FGF12,FGF12,FGF12-AS1,MB21D2,HRASLS,ATP13A5,ATP13A5,ATP13A5-AS1,ATP13A4,ATP13A4,ATP13A4-AS1,OPA1,OPA1,OPA1-AS1,HES1,CPN2,LRRC15,GP5,ATP13A3,TMEM44-AS1,TMEM44,TMEM44,LSG1,FAM43A,XXYLT1,XXYLT1,MIR3137,ACAP2,PPP1R2,APOD,LOC105374297,SDHAP2,SDHAP2,MIR570HG,MIR570HG,MIR570,MUC20,MUC4,TNK2,TNK2-AS1,SDHAP1,TFRC,ZDHHC19,SLC51A,PCYT1A,TCTEX1D2,TCTEX1D2,TM4SF19-TCTEX1D2,TM4SF19-TCTEX1D2,TM4SF19-AS1,TM4SF19,TM4SF19-TCTEX1D2,TM4SF19,UBXN7,UBXN7,UBXN7-AS1,RNF168,SMCO1,WDR53,FBXO45,NRROS,CEP19,PIGX,PAK2,SENP5,NCBP2,NCBP2,NCBP2-AS1,NCBP2-AS2,PIGZ,MELTF,MELTF-AS1,MELTF,DLG1,DLG1,MIR4797,BDH1,RUBCN,MIR922,RUBCN,RUBCN,FYTTD1,FYTTD1,LRCH3,IQCG,IQCG,RPL35A,LMLN,LMLN,LMLN-AS1 | -0.871317 | 126.929 | 918 | 546.534 |
| 3 | 11 | 8936346 | 9305180 | AKIP1,C11orf16,ASCL3,TMEM9B,NRIP3,SCUBE2,SCUBE2,MIR5691,DENND5A,TMEM41B | -0.871652 | 115.906 | 95 | 57.774 |
| 3 | 4 | 1.14E+08 | 128811372 | ANK2,ANK2,MIR1243,CAMK2D,ARSJ,UGT8,UGT8,MIR577,NDST4,TRAM1L1,NDST3,SNHG8,SNORA24,PRSS12,CEP170P1,METTL14,SEC24D,SYNPO2,MYOZ2,LOC101929762,USP53,C4orf3,FABP2,LOC645513,PDE5A,PDE5A,MAD2L1,PRDM5,NDNF,TNIP3,QRFPR,ANXA5,TMEM155,PP12613,EXOSC9,EXOSC9,CCNA2,CCNA2,BBS7,TRPC3,KIAA1109,ADAD1,IL2,IL21,IL21,IL21-AS1,BBS12,FGF2,FGF2,NUDT6,NUDT6,SPATA5,SPRY1,ANKRD50,FAT4,MIR2054,INTU,SLC25A31,HSPA4L,PLK4 | -0.87388 | 136.101 | 922 | 570.233 |

| | | | | | | | | |
|---|---|---|---|---|---|---|---|---|
| 3 | X | 1.14E+08 | 117677015 | LRCH2,RBMXL3,LRCH2,LUZP4,PLS3,AGTR2,SLC6A14,CT83,KLHL13,WDR44,WDR44,MIR1277,DOCK11 | -0.874032 | 125.478 | 137 | 81.5164 |
| 3 | 13 | 22097333 | 25440410 | MICU2,FGF9,SGCG,SACS,TNFRSF19,MIPEP,MIPEP,PCOTH,PCOTH,C1QTNF9B,C1QTNF9B,SPATA13,MIR2276,SPATA13,C1QTNF9,PARP4,TPTE2P6,ATP12A,RNF17 | -0.876032 | 133.692 | 293 | 175.418 |
| 3 | 2 | 10500 | 11312163 | FAM110C,SH3YL1,ACP1,ALKAL2,TMEM18,LOC101060391,SNTG2,SNTG2,TPO,PXDN,MYT1L,EIPR1,TRAPPC12,ADI1,RNASEH1,RPS7,COLEC11,ALLC,DCDC2C,SOX11,CMPK2,RSAD2,RNF144A,RNF144A,LOC101929452,LINC00299,ID2,KIDINS220,MBOAT2,ASAP2,ITGB1BP1,ITGB1BP1,CPSF3,CPSF3,IAH1,ADAM17,YWHAQ,TAF1B,GRHL1,KLF11,CYS1,RRM2,C2orf48,C2orf48,MIR4261,HPCAL1,ODC1,ODC1,SNORA80B,LOC101929715,NOL10,ATP6V1C2,ATP6V1C2,PDIA6,PDIA6,KCNF1,FLJ33534,C2orf50,PQLC3 | -0.876914 | 124.7 | 731 | 435.623 |
| 3 | 10 | 1.18E+08 | 124343959 | PNLIPRP3,PNLIP,PNLIPRP1,PNLIPRP2,C10orf82,HSPA12A,HSPA12A,ENO4,ENO4,SHTN1,VAX1,MIR3663HG,MIR3663,KCNK18,SLC18A2,PDZD8,EMX2OS,EMX2,EMX2,RAB11FIP2,FAM204A,PRLHR,CACUL1,NANOS1,EIF3A,EIF3A,SNORA19,FAM45A,FAM45BP,SFXN4,PRDX3,GRK5,GRK5,MIR4681,RGS10,TIAL1,BAG3,INPP5F,MCMBP,SEC23IP,MIR4682,PLPP4,LINC01561,WDR11,FGFR2,ATE1,ATE1,ATE1-AS1,NSMCE4A,TACC2,BTBD16,PLEKHA1,PLEKHA1,MIR3941,ARMS2,HTRA1,DMBT1 | -0.87731 | 126.935 | 648 | 393.872 |
| 3 | 4 | 1.29E+08 | 141295437 | LARP1B,PGRMC2,JADE1,SCLT1,C4orf33,PCDH10,PABPC4L,PCDH18,SLC7A11-AS1,SLC7A11,SLC7A11,NOCT,ELF2,MGARP,NDUFC1,NDUFC1,NAA15,NAA15,RAB33B,SETD7,MGST2,MGST2,MAML3,MAML3,MAML3,LOC101927516,SCOC,SCOC-AS1,SCOC | -0.884619 | 106.196 | 320 | 191.66 |
| 3 | 10 | 1.15E+08 | 116702558 | CASP7,PLEKHS1,PLEKHS1,MIR4483,DCLRE1A,NHLRC2,ADRB1,CCDC186,CCDC186,MIR2110,TDRD1,VWA2,AFAP1L2,ABLIM1,ABLIM1,LOC101927692,FAM160B1,TRUB1 | -0.891202 | 131.598 | 216 | 133.58 |
| 3 | 3 | 56592736 | 56707747 | CCDC66,CCDC66,FAM208A,FAM208A | -0.892753 | 180.677 | 57 | 34.9573 |
| 3 | 3 | 1.6E+08 | 160254708 | TRIM59-IFT80,IFT80,TRIM59-IFT80,SMC4,TRIM59-IFT80,SMC4,MIR15B,MIR16-2,TRIM59-IFT80,TRIM59,TRIM59-IFT80,KPNA4,KPNA4,SCARNA7 | -0.893313 | 147.368 | 87 | 47.383 |
| 3 | 8 | 1.26E+08 | 125535312 | TATDN1,TATDN1,MIR6844 | -0.895689 | 156.997 | 16 | 7.8584 |

| | | | | | | | | |
|---|---|---|---|---|---|---|---|---|
| 3 | 4 | 1.46E+08 | 164774623 | OTUD4,SMAD1,SMAD1-AS1,SMAD1,MMAA,C4orf51,ZNF827,LSM6,REELD1,SLC10A7,POU4F2,TTC29,EDNRA,TMEM184C,PRMT9,ARHGAP10,ARHGAP10,MIR4799,NR3C2,DCLK2,LRBA,LRBA,LOC729558,LRBA,MAB21L2,RPS3A,RPS3A,SNORD73B,RPS3A,SNORD73A,SH3D19,PRSS48,FAM160A1,GATB,FBXW7,FBXW7,FBXW7-AS1,FBXW7,MIR3140,MIR4453HG,MIR4453,TMEM154,TIGD4,ARFIP1,FHDC1,TRIM2,TRIM2,ANXA2P1,MND1,TMEM131L,TLR2,RNF175,SFRP2,DCHS2,PLRG1,FGB,FGA,FGG,LRAT,RBM46,NPY2R,MAP9,MAP9,LOC102724776,GUCY1A1,GUCY1B1,ASIC5,TDO2,CTSO,PDGFC,GLRB,GRIA2,FAM198B,FAM198B,FAM198B-AS1,TMEM144,RXFP1,C4orf46,ETFDH,PPID,FNIP2,FNIP2,C4orf45,C4orf45,RAPGEF2,FSTL5,FSTL5,LOC101928052,NAF1,NPY1R,NPY5R,TKTL2,TMA16,MARCH1 | -0.907938 | 117.536 | 1149 | 696.001 |
| 3 | 6 | 1.23E+08 | 123539924 | HSF2,SERINC1,PKIB,FABP7,SMPDL3A,CLVS2,TRDN | -0.912494 | 136.909 | 45 | 26.729 |
| 3 | 14 | 31210295 | 35032409 | COCH,COCH,LOC100506071,STRN3,STRN3,MIR624,STRN3,AP4S1,AP4S1,HECTD1,HEATR5A,LOC101927124,LOC101927124,DTD2,DTD2,GPR33,NUBPL,LINC02313,ARHGAP5,AKAP6,NPAS3,EGLN3,SPTSSA,EAPP,SNX6 | -0.92178 | 124.101 | 313 | 187.357 |
| 3 | 9 | 99296129 | 99522759 | CDC14B,AAED1,ZNF510 | -0.926862 | 153.997 | 29 | 15.7356 |
| 3 | 18 | 31158539 | 35388792 | ASXL3,NOL4,DTNA,MAPRE2,ZNF397,ZNF397,ZSCAN30,ZSCAN30,ZNF271P,ZNF24,ZNF396,INO80C,MIR3975,GALNT1,MIR187,MIR3929,C18orf21,RPRD1A,SLC39A6,ELP2,MOCOS,FHOD3,TPGS2,TPGS2,KIAA1328,KIAA1328,CELF4,CELF4,LOC105372068,MIR4318 | -0.93279 | 124.385 | 364 | 217.812 |
| 3 | 11 | 9467174 | 31085458 | ZNF143,WEE1,SWAP70,SBF2-AS1,SBF2,SBF2,SBF2,LOC101928008,ADM,AMPD3,RNF141,MRVI1-AS1,LYVE1,MRVI1-AS1,MRVI1,MRVI1,CTR9,EIF4G2,EIF4G2,SNORD97,ZBED5,GALNT18,GALNT18,CSNK2A3,MIR4299,USP47,DKK3,MICAL2,MICALCL,PARVA,TEAD1,RASSF10,ARNTL,BTBD10,PTH,FAR1,SPON1,RRAS2,COPB1,PSMA1,PDE3B,CYP2R1,CALCA,CALCB,INSC,SOX6,C11orf58,PLEKHA7,OR7E14P,RPS13,PIK3C2A,NUCB2,NCR3LG1,KCNJ11,ABCC8,USH1C,OTOG,MYOD1,KCNC1,SERGEF,TPH1,SAAL1,MRGPRX3,MRGPRX4,LOC494141,SAA4,SAA2-SAA4,SAA2-SAA4,SAA2,SAA1,HPS5,GTF2H1,LDHA,LDHC,LDHAL6A,TSG101,UEVLD,SPTY2D1OS,SPTY2D1,SPTY2D1,TMEM86A,TMEM86A,IGSF22,IGSF22,PTPN5,MRGPRX1,MRGPRX2,ZDHHC13,CSRP3,E2F8,NAV2,NAV2,MIR4486,NAV2,LOC100126784,NAV2,MIR4694,NAV2,NAV2-AS2,DBX1,HTATIP2,PRMT3,SLC6A5,NELL1,ANO5,SLC17A6,FANCF,GAS2,SVIP,CCDC179,LUZP2,ANO3,ANO3,MUC15,SLC5A12,FIBIN,BBOX1,BBOX1-AS1,CCDC34,LGR4,LGR4,LOC105376671,LIN7C,LIN7C,BDNF-AS,BDNF-AS,BDNF,BDNF,KIF18A,KIF18A,MIR610,METTL15,KCNA4,FSHB,ARL14EP,MPPED2,DCDC1 | -0.940971 | 123.96 | 1705 | 1019.37 |

| | | | | | | | | |
|---|---|---|---|---|---|---|---|---|
| 3 | 6 | 32261677 | 32336732 | LOC101929163,C6orf10 | -0.943509 | 133.104 | 40 | 22.8874 |
| 3 | 13 | 35619420 | 48878244 | NBEA,NBEA,MAB21L1,DCLK1,SOHLH2,CCDC169-SOHLH2,CCDC169-SOHLH2,CCDC169,SPART,SPART,SPART-AS1,CCNA1,SERTM1,RFXAP,SMAD9,ALG5,EXOSC8,SUPT20H,CSNK1A1L,POSTN,TRPC4,UFM1,FREM2,STOML3,PROSER1,NHLRC3,LHFPL6,COG6,COG6,MIR4305,FOXO1,MIR320D1,MRPS31,SLC25A15,SLC25A15,TPTE2P5,SLC25A15,TPTE2P5,MIR621,TPTE2P5,TPTE2P5,SUGT1P3,ELF1,WBP4,MIR3168,KBTBD6,LOC101929140,KBTBD7,LOC101929140,MTRF1,MTRF1,MTRF1,NAA16,NAA16,RGCC,VWA8,MIR5006,VWA8,DGKH,AKAP11,TNFSF11,FAM216B,EPSTI1,DNAJC15,ENOX1,CCDC122,LACC1,LINC00390,SMIM2-AS1,SMIM2,SMIM2,SERP2,TSC22D1,TSC22D1,TSC22D1-AS1,NUFIP1,GPALPP1,GTF2F2,GTF2F2,KCTD4,TPT1,SNORA31,TPT1,SLC25A30,COG3,ERICH6B,SPERT,SIAH3,ZC3H13,CPB2-AS1,CPB2,CPB2,LCP1,LRRC63,RUBCNL,LRCH1,ESD,HTR2A,SUCLA2,NUDT15,MED4,MED4-AS1,MED4,ITM2B,RB1 | -0.945992 | 122.21 | 1111 | 655.547 |
| 3 | 15 | 44816844 | 49800538 | EIF3J-AS1,EIF3J,SPG11,PATL2,B2M,TRIM69,SORD2P,TERB2,SORD,DUOX2,DUOXA2,DUOXA2,DUOXA1,DUOXA1,DUOX1,SHF,LOC101928414,SLC28A2,GATM,SPATA5L1,C15orf48,C15orf48,MIR147B,SLC30A4,SLC30A4,HMGN2P46,HMGN2P46,BLOC1S6,SQOR,SEMA6D,SLC24A5,SLC24A5,MYEF2,MYEF2,CTXN2,SLC12A1,DUT,FBN1,CEP152,SHC4,SHC4,EID1,SECISBP2L,COPS2,GALK2,GALK2,MIR4716,GALK2,FAM227B,FAM227B,FAM227B,FGF7 | -0.946404 | 132.058 | 621 | 374.509 |
| 3 | 4 | 1.04E+08 | 113524863 | CENPE,TACR3,CXXC4,CXXC4,CXXC4-AS1,TET2,TET2-AS1,PPA2,ARHGEF38,INTS12,GSTCD,GSTCD,LOC101929529,NPNT,LOC101929577,TBCK,TBCK,AIMP1,AIMP1,GIMD1,DKK2,PAPSS1,SGMS2,LOC101929595,LOC101929595,CYP2U1,CYP2U1,HADH,LEF1,LEF1,LEF1-AS1,RPL34-AS1,RPL34,OSTC,ETNPPL,COL25A1,SEC24B,MCUB,CASP6,PLA2G12A,CFI,GAR1,RRH,LRIT3,EGF,ELOVL6,ENPEP,PITX2,FAM241A,AP1AR,TIFA,ALPK1,NEUROG2,NEUROG2,LOC105377372,LOC105377372,ZGRF1,ZGRF1 | -0.948066 | 116.813 | 617 | 368.01 |
| 3 | 19 | 36832130 | 38314183 | ZFP14,ZFP82,ZNF566,ZNF566,LOC728752,LOC728752,ZNF260,ZNF529,ZNF529,ZNF529-AS1,ZNF382,ZNF461,ZNF567,ZNF850,ZNF790-AS1,ZNF790,ZNF345,ZNF345,ZNF829,ZNF829,ZNF568,ZNF420,ZNF585A,ZNF585B,ZNF383,HKR1,ZNF527,ZNF569,ZNF570,ZNF793,ZNF571-AS1,ZNF571-AS1,ZNF540,ZNF571-AS1,ZNF540,ZNF571,ZNF540,ZFP30,ZNF781,ZNF607,ZNF573 | -0.948348 | 119.078 | 341 | 196.718 |
| 3 | 4 | 1.41E+08 | 145916699 | UCP1,TBC1D9,RNF150,ZNF330,IL15,INPP4B,USP38,GAB1,GAB1,MIR3139,SMARCA5,SMARCA5-AS1,SMARCA5,FREM3,GYPE,GYPB,GYPA,HHIP,ANAPC10 | -0.955112 | 118.728 | 293 | 170.558 |

| | | | | | | | | |
|---|---|---|---|---|---|---|---|---|
| 3 | 4 | 71067907 | 84378159 | ODAM,FDCSP,CSN3,CABS1,SMR3A,SMR3B,OPRPN,MUC7,AMTN,AMBN,ENAM,JCHAIN,UTP3,RUFY3,GRSF1,MOB1B,DCK,SLC4A4,GC,NPFFR2,ADAMTS3,COX18,ANKRD17,ALB,AFP,AFM,RASSF6,CXCL8,CXCL6,PF4V1,CXCL1,PF4,PPBP,CXCL5,CXCL3,CXCL2,MTHFD2L,EPGN,EREG,AREG,BTC,PARM1,RCHY1,THAP6,ODAPH,CDKL2,G3BP2,USO1,PPEF2,NAAA,SDAD1,SDAD1,LOC101928809,LOC101928809,CXCL9,CXCL9,ART3,CXCL10,ART3,CXCL11,ART3,NUP54,SCARB2,FAM47E,FAM47E,FAM47E-STBD1,FAM47E-STBD1,FAM47E-STBD1,STBD1,CCDC158,SHROOM3,SHROOM3,MIR4450,SOWAHB,SEPT11,CCNI,CCNG2,CXCL13,CNOT6L,MRPL1,FRAS1,ANXA3,BMP2K,PAQR3,NAA11,GK2,ANTXR2,PRDM8,FGF5,C4orf22,BMP3,PRKG2,PRKG2,LOC101928942,RASGEF1B,HNRNPD,HNRNPDL,ENOPH1,TMEM150C,SCD5,SCD5,MIR575,SEC31A,THAP9-AS1,THAP9,THAP9,LIN54,COPS4,PLAC8,COQ2,HPSE,HELQ,MRPS18C | -0.956536 | 125.906 | 1329 | 783.71 |
| 3 | 3 | 1.8E+08 | 182683535 | TTC14,TTC14,CCDC39,CCDC39,LOC101928882,FXR1,DNAJC19,SOX2-OT,SOX2,ATP11B,DCUN1D1 | -0.956685 | 137.91 | 135 | 76.5601 |
| 3 | X | 31697475 | 46617595 | DMD,DMD,MIR3915,DMD,MIR548F5,FAM47A,TMEM47,FAM47B,MAGEB16,CFAP47,CFAP47,LOC101928627,FAM47C,PRRG1,LANCL3,XK,CYBB,DYNLT3,HYPM,SYTL5,MIR548AJ2,SYTL5,SRPX,RPGR,OTC,TSPAN7,MID1IP1,MIR3937,BCOR,ATP6AP2,MPC1L,CXorf38,MED14,MED14,MED14OS,MED14OS,USP9X,DDX3X,NYX,CASK,CASK,GPR34,CASK,GPR82,PPP1R2P9,MAOA,MAOB,NDP,NDP-AS1,EFHC2,FUNDC1,DUSP21,KDM6A,CXorf36,MIR221,MIR222,KRBOX4,ZNF674,CHST7,SLC9A7 | -0.967783 | 117.762 | 814 | 470.638 |
| 3 | 10 | 55997229 | 60154354 | PCDH15,PCDH15,LOC105378311,PCDH15,LOC105378311,MIR548F1,ZWINT,MIR3924,IPMK,CISD1,UBE2D1,TFAM | -0.968612 | 90.2605 | 87 | 49.7188 |
| 3 | 4 | 1.85E+08 | 185550705 | RWDD4,TRAPPC11,STOX2,ENPP6,IRF2,CASP3 | -0.969661 | 127.063 | 74 | 46.1692 |
| 3 | 21 | 40578007 | 40650808 | BRWD1 | -0.970748 | 162.204 | 27 | 16.5604 |
| 3 | 5 | 1.17E+08 | 132569313 | LINC02147,LINC02208,LINC02215,DTWD2,DMXL1,DMXL1,MIR5706,TNFAIP8,HSD17B4,FAM170A,PRR16,FTMT,SRFBP1,LOX,ZNF474,LOC100505841,SNCAIP,SNCAIP,MGC32805,LINC02201,SNX2,SNX24,PPIC,LOC105379152,PRDM6,PRDM6,CEP120,CSNK1G3,ZNF608,GRAMD2B,ALDH7A1,PHAX,TEX43,LMNB1,MARCH3,C5orf63,MEGF10,PRRC1,CTXN3,SLC12A2,FBN2,SLC27A6,ISOC1,ISOC1,MIR4633,MIR4460,ADAMTS19-AS1,ADAMTS19,ADAMTS19,KIAA1024L,CHSY3,HINT1,LYRM7,CDC42SE2,RAPGEF6,FNIP1,ACSL6,IL3,CSF2,P4HA2,P4HA2,MIR6830,PDLIM4,SLC22A4,SLC22A4,MIR3936HG,MIR3936HG,MIR3936,SLC22A5,C5orf56,IRF1,IL5,RAD50,RAD50,TH2LCRR,IL13,IL4,IL4,LOC105379176,KIF3A,CCNI2,CCNI2,SEPT8,SEPT8,SOWAHA,SHROOM1,GDF9,GDF9,UQCRQ,UQCRQ,LEAP2,AFF4,ZCCHC10,HSPA4,FSTL4 | -0.975502 | 120.988 | 1090 | 656.742 |

| | | | | | | | | |
|---|---|---|---|---|---|---|---|---|
| 3 | 18 | 42281305 | 65181867 | SETBP1,SETBP1,MIR4319,SLC14A2,SLC14A1,SIGLEC15,EPG5,PSTPIP2,ATP5F1A,HAUS1,C18orf25,RNF165,LOXHD1,ST8SIA5,PIAS2,PIAS2,KATNAL2,KATNAL2,KATNAL2,ELOA2,HDHD2,IER3IP1,SKOR2,MIR4527HG,MIR4527,SMAD2,ZBTB7C,CTIF,CTIF,MIR4743,SMAD7,LOC100129878,DYM,DYM,MIR4744,DYM,C18orf32,RPL17-C18orf32,RPL17-C18orf32,MIR1539,RPL17-C18orf32,RPL17,RPL17-C18orf32,RPL17,SNORD58C,RPL17-C18orf32,RPL17,SNORD58A,RPL17-C18orf32,RPL17,SNORD58B,LIPG,ACAA2,SCARNA17,SNHG22,SNHG22,MYO5B,MYO5B,MYO5B,MIR4320,CFAP53,MBD1,CXXC1,SKA1,MAPK4,MRO,ME2,ELAC1,SMAD4,MEX3C,LINC01630,DCC,DCC,MIR4528,MBD2,MBD2,SNORA37,POLI,STARD6,C18orf54,DYNAP,RAB27B,CCDC68,TCF4,TCF4,TCF4-AS1,TCF4,TCF4-AS1,MIR4529,LOC642484,LOC642484,LINC01905,TXNL1,WDR7,BOD1L2,ST8SIA3,ONECUT2,FECH,NARS,LOC100505549,LOC100505549,ATP8B1,ATP8B1,NEDD4L,MIR122,MIR3591,ALPK2,LOC101927322,MALT1,MALT1,ZNF532,OACYLP,SEC11C,GRP,RAX,CPLX4,LMAN1,CCBE1,PMAIP1,MC4R,CDH20,RNF152,PIGN,KIAA1468,TNFRSF11A,ZCCHC2,PHLPP1,BCL2,KDSR,VPS4B,SERPINB5,SERPINB12,SERPINB13,SERPINB4,SERPINB3,SERPINB11,SERPINB7,SERPINB2,SERPINB10,HMSD,SERPINB8,LINC00305,CDH7,CDH19,MIR5011,DSEL | -0.975974 | 123.918 | 1570 | 940.203 |
| 3 | 2 | 31756413 | 32422889 | SRD5A2,MEMO1,DPY30,SPAST,SLC30A6 | -0.993116 | 113.132 | 65 | 32.3927 |
| 3 | 6 | 38690985 | 38834654 | DNAH8 | -0.998031 | 145.704 | 47 | 29.3928 |
| 3 | 12 | 88588624 | 100676960 | TMTC3,KITLG,DUSP6,POC1B,POC1B,GALNT4,POC1B-GALNT4,POC1B,POC1B-GALNT4,POC1B,POC1B-GALNT4,POC1B-AS1,ATP2B1,LINC00615,CCER1,EPYC,KERA,LUM,DCN,LINC01619,BTG1,CLLU1OS,CLLU1OS,CLLU1,C12orf74,PLEKHG7,EEA1,LOC643339,LOC643339,LINC02412,NUDT4,NUDT4P2,NUDT4B,UBE2N,MRPL42,SOCS2-AS1,SOCS2,SOCS2,CRADD,PLXNC1,CEP83,MIR5700,TMCC3,MIR492,MIR492,KRT19P2,NDUFA12,NR2C1,FGD6,VEZT,MIR331,MIR3685,METAP2,USP44,PGAM1P5,NTN4,NTN4,LOC105369921,LINC02410,SNRPF,CCDC38,AMDHD1,HAL,LTA4H,ELK3,CDK17,CFAP54,NEDD1,RMST,MIR1251,MIR135A2,MIR4495,MIR4303,SLC9A7P1,LINC02453,TMPO-AS1,TMPO,TMPO,SLC25A3,SLC25A3,SNORA53,IKBIP,APAF1,ANKS1B,ANKS1B,FAM71C,UHRF1BP1L,GOLGA2P5,ACTR6,ACTR6,DEPDC4,DEPDC4,SCYL2 | -1.00014 | 118.38 | 901 | 534.981 |
| 3 | 6 | 1.22E+08 | 122733995 | TBC1D32,GJA1,HSF2 | -1.00209 | 116.061 | 23 | 13.4372 |

| | | | | | | | | |
|---|---|---|---|---|---|---|---|---|
| 3 | 13 | 48956089 | 53036581 | RB1,LPAR6,RB1,RCBTB2,CYSLTR2,FNDC3A,MLNR,CDADC1,CAB39L,SETDB2,SETDB2-PHF11,SETDB2-PHF11,PHF11,RCBTB1,ARL11,EBPL,KPNA3,CTAGE10P,SPRYD7,DLEU2,MIR3613,DLEU2,TRIM13,DLEU2,TRIM13,KCNRG,DLEU2,KCNRG,DLEU2,MIR16-1,MIR15A,DLEU2,DLEU1,DLEU1,ST13P4,DLEU7,DLEU7,DLEU7-AS1,RNASEH2B-AS1,RNASEH2B,RNASEH2B,GUCY1B2,FAM124A,SERPINE3,SERPINE3,MIR5693,SERPINE3,INTS6,INTS6,MIR4703,WDFY2,DHRS12,TMEM272,CCDC70,ATP7B,ALG11,ALG11,UTP14C,UTP14C,NEK5,NEK3,MRPS31P5,THSD1,VPS36,CKAP2 | -1.00536 | 122.883 | 473 | 281.822 |
| 3 | 13 | 19020500 | 21949354 | TUBA3C,LOC101928697,TPTE2,MPHOSPH8,PSPC1,ZMYM5,ZMYM2,GJA3,GJB2,GJB6,CRYL1,CRYL1,MIR4499,IFT88,IL17D,EEF1AKMT1,XPO4,LATS2,SAP18,SKA3,SKA3,MRPL57,MRPL57,MIPEPP3,ZDHHC20 | -1.0056 | 117.098 | 268 | 150.783 |
| 3 | 6 | 1.5E+08 | 149862732 | PPIL4 | -1.00922 | 137.223 | 13 | 6.87271 |
| 3 | 9 | 1.24E+08 | 123877547 | C5,CNTRL | -1.01144 | 135.263 | 22 | 12.606 |
| 3 | 4 | 84406139 | 104032182 | ABRAXAS1,GPAT3,LOC101928978,NKX6-1,CDS1,WDFY3,ARHGAP24,ARHGAP24,MIR4451,MAPK10,MAPK10,LOC101929064,PTPN13,SLC10A6,C4orf36,AFF1,KLHL8,HSD17B13,HSD17B11,NUDT9,SPARCL1,DSPP,DMP1,IBSP,MEPE,SPP1,PKD2,ABCG2,PPM1K,HERC6,HERC5,PYURF,PIGY,HERC3,HERC3,NAP1L5,FAM13A-AS1,FAM13A,FAM13A,TIGD2,GPRIN3,SNCA,MMRN1,CCSER1,GRID2,ATOH1,SMARCAD1,HPGDS,PDLIM5,BMPR1B,UNC5C,PDHA2,STPG2,RAP1GDS1,TSPAN5,EIF4E,METAP1,METAP1,MIR3684,ADH5,LOC100507053,ADH4,LOC100507053,ADH6,LOC100507053,ADH1A,ADH1B,ADH1C,ADH7,C4orf17,TRMT10A,MTTP,LOC285556,DAPP1,LAMTOR3,DNAJB14,H2AFZ,DDIT4L,DDIT4L,LOC101929353,EMCN,PPP3CA,PPP3CA,MIR1255A,FLJ20021,BANK1,SLC39A8,NFKB1,MANBA,LOC102723704,UBE2D3,UBE2D3,CISD2,CISD2,SLC9B1,SLC9B1,SLC9B2,BDH2,CENPE | -1.01422 | 115.539 | 1309 | 777.19 |

| | | | | | | | | |
|---|---|---|---|---|---|---|---|---|
| 3 | 4 | 17814730 | 49632814 | NCAPG,NCAPG,LCORL,LCORL,SLIT2,SLIT2,MIR218-1,PACRGL,PACRGL,KCNIP4,KCNIP4,ADGRA3,GBA3,PPARGC1A,MIR573,DHX15,SOD3,CCDC149,LGI2,SEPSECS,PI4K2B,ZCCHC4,ANAPC4,SLC34A2,SEL1L3,SMIM20,RBPJ,CCKAR,TBC1D19,STIM2,MIR4275,PCDH7,ARAP2,LOC439933,DTHD1,MIR1255B1,MIR4801,NWD2,C4orf19,C4orf19,RELL1,RELL1,PGM2,TBC1D1,TBC1D1,PTTG2,KLF3,TLR10,TLR1,TLR6,FAM114A1,MIR574,FAM114A1,TMEM156,KLHL5,WDR19,RFC1,KLB,RPL9,LIAS,UGDH,UGDH-AS1,SMIM14,SMIM14,UBE2K,PDS5A,LOC344967,N4BP2,RHOH,CHRNA9,RBM47,RBM47,MIR4802,NSUN7,APBB2,UCHL1,LIMCH1,PHOX2B,TMEM33,DCAF4L1,SLC30A9,BEND4,SHISA3,ATP8A1,GRXCR1,KCTD8,YIPF7,GUF1,GNPDA2,GABRG1,GABRA2,COX7B2,GABRA4,GABRB1,COMMD8,ATP10D,CORIN,CORIN,LOC101927179,LOC101927179,NFXL1,NFXL1,NFXL1,LOC101927157,LOC101927157,CNGA1,NIPAL1,TXK,TEC,SLAIN2,SLC10A4,ZAR1,FRYL,OCIAD1,OCIAD1,OCIAD1-AS1,OCIAD2,CWH43 | -1.01447 | 114.201 | 1708 | 1023.52 |
| 3 | 15 | 42509923 | 42858951 | TMEM87A,GANC,CAPN3,ZNF106,SNAP23,LRRC57,HAUS2 | -1.03114 | 121.235 | 145 | 89.8619 |
| 3 | 3 | 1.31E+08 | 134369981 | ATP2C1,ATP2C1,ASTE1,ASTE1,NEK11,NEK11,LOC339874,NUDT16,MRPL3,MRPL3,SNORA58,CPNE4,CPNE4,MIR5704,ACPP,DNAJC13,ACAD11,NPHP3-ACAD11,ACAD11,NPHP3-ACAD11,ACKR4,ACAD11,NPHP3-ACAD11,UBA5,NPHP3-ACAD11,UBA5,NPHP3-ACAD11,NPHP3,TMEM108,BFSP2,BFSP2,BFSP2-AS1,CDV3,TOPBP1,TF,SRPRB,RAB6B,C3orf36,SLCO2A1,RYK,AMOTL2,AMOTL2,MIR6827,MIR4788,ANAPC13,CEP63,KY | -1.03594 | 120.569 | 471 | 282.211 |
| 3 | 16 | 49764706 | 54280960 | ZNF423,CNEP1R1,HEATR3,PAPD5,ADCY7,ADCY7,MIR6771,BRD7,NKD1,SNX20,SNX20,LOC101927272,NOD2,CYLD,MIR3181,CYLD,LINC02168,SALL1,C16orf97,LINC00919,TOX3,LOC105371267,CHD9,LOC643802,RBL2,AKTIP,RPGRIP1L,FTO,LINC02169 | -1.03646 | 116.94 | 391 | 233.641 |
| 3 | 10 | 96367745 | 98363870 | CYP2C18,CYP2C19,CYP2C9,CYP2C8,ACSM6,PDLIM1,SORBS1,ALDH18A1,TCTN3,ENTPD1,ENTPD1,ENTPD1-AS1,ENTPD1-AS1,CC2D2B,ENTPD1-AS1,CCNJ,ENTPD1-AS1,MIR3157,ZNF518A,ZNF518A,BLNK,BLNK,DNTT,OPALIN,TLL2,TM9SF3,PIK3AP1 | -1.03755 | 119.583 | 332 | 195.86 |
| 3 | 13 | 88636973 | 96508586 | MIR17HG,MIR17,MIR18A,MIR17HG,MIR19A,MIR20A,MIR17HG,MIR19B1,MIR92A1,GPC5,GPC5,MIR548AS,GPC6,GPC6,GPC6-AS2,DCT,TGDS,GPR180,SOX21,ABCC4,CLDN10,DZIP1,DNAJC3,UGGT2 | -1.04123 | 90.5248 | 224 | 134.065 |
| 3 | X | 1.5E+08 | 149999808 | MTM1,MTMR1,CD99L2 | -1.04155 | 117.02 | 53 | 31.7423 |

| | | | | | | | |
|---|---|---|---|---|---|---|---|
| 3 | 18 | 19115991 | 30518068 | ESCO1,SNRPD1,ABHD3,ABHD3,MIR320C1,MIB1,MIB1,MIR133A1HG,MIR133A1,MIB1,MIR133A1HG,MIR1-2,GATA6,CTAGE1,RBBP8,MIR4741,RBBP8,CABLES1,TMEM241,RIOK3,RMC1,RMC1,NPC1,NPC1,ANKRD29,LAMA3,TTC39C,TTC39C-AS1,TTC39C,CABYR,OSBPL1A,OSBPL1A,MIR320C2,IMPACT,HRH4,ZNF521,SS18,PSMA8,TAF4B,KCTD1,AQP4,AQP4,AQP4-AS1,AQP4-AS1,CHST9,CHST9,CDH2,MIR302F,DSC3,DSC2,DSC2,DSCAS,DSCAS,DSC1,DSG1,DSG1,DSG1-AS1,DSG1-AS1,DSG4,DSG3,DSG2,DSG2,DSG2-AS1,TTR,B4GALT6,SLC25A52,TRAPPC8,RNF125,RNF138,MEP1B,GAREM1,WBP11P1,KLHL14,CCDC178 | -1.04319 | 119.88 | 849 | 511.096 |
| 3 | 7 | 74105344 | 74167458 | GTF2I,LOC101926943,GTF2I | -1.04489 | 181.886 | 14 | 6.65245 |
| 3 | 4 | 57366803 | 68337860 | SRP72,ARL9,THEGL,HOPX,SPINK2,REST,NOA1,POLR2B,IGFBP7,IGFBP7,IGFBP7-AS1,MIR548AG1,ADGRL3,TECRL,EPHA5,MIR1269A | -1.04569 | 87.4187 | 249 | 152.355 |
| 3 | 11 | 94153276 | 94229910 | MRE11,MRE11,MIR548L,ANKRD49 | -1.04635 | 132.341 | 30 | 17.1285 |
| 3 | 7 | 48289504 | 48319514 | ABCA13 | -1.05169 | 142.68 | 27 | 19.6569 |
| 3 | 6 | 86284641 | 121433831 | SNX14,SYNCRIP,SNHG5,SNORD50A,SNHG5,SNORD50B,HTR1E,CGA,ZNF292,GJB7,SMIM8,C6orf163,LINC01590,CFAP206,SLC35A1,RARS2,ORC3,AKIRIN2,SPACA1,CNR1,RNGTT,PNRC1,SRSF12,PM20D2,GABRR1,GABRR2,UBE2J1,RRAGD,ANKRD6,LYRM2,LYRM2,LOC101929057,LOC101929057,MDN1,MDN1,CASP8AP2,GJA10,BACH2,MIR4464,MAP3K7,MIR4643,EPHA7,MANEA,FUT9,UFL1,FHL5,GPR63,NDUFAF4,KLHL32,KLHL32,MIR548H3,MIR548H3,MMS22L,MIR2113,POU3F2,FBXL4,MIR548AI,FAXC,COQ3,PNISR,USP45,TSTD3,CCNC,PRDM13,MCHR2,SIM1,ASCC3,GRIK2,HACE1,LIN28B-AS1,LIN28B,BVES,BVES-AS1,BVES-AS1,POPDC3,PREP,PRDM1,ATG5,LOC105377924,CRYBG1,RTN4IP1,RTN4IP1,QRSL1,QRSL1,LINC02532,MIR587,C6orf203,BEND3,PDSS2,SOBP,SCML4,SEC63,OSTM1,NR2E1,SNX3,AFG1L,FOXO3,ARMC2,ARMC2,ARMC2-AS1,SESN1,CEP57L1,CCDC162P,C6orf183,CCDC162P,CD164,PPIL6,PPIL6,SMPD2,SMPD2,MICAL1,MICAL1,ZBTB24,ZBTB24,AK9,FIG4,GPR6,WASF1,CDC40,METTL24,DDO,SLC22A16,CDK19,CDK19,AMD1,AMD1,GTF3C6,RPF2,GSTM2P1,SLC16A10,MFSD4B,REV3L,REV3L,TRAF3IP2-AS1,TRAF3IP2-AS1,TRAF3IP2-AS1,TRAF3IP2,FYN,WISP3,TUBE1,FAM229B,LAMA4,LAMA4,LOC101927640,RFPL4B,MARCKS,HDAC2,HDAC2,HDAC2-AS2,HDAC2-AS2,HS3ST5,FRK,NT5DC1,NT5DC1,COL10A1,TSPYL4,DSE,DSE,TSPYL1,DSE,LOC100287467,CALHM6,TRAPPC3L,TRAPPC3L,CALHM5,TRAPPC3L,CALHM4,CALHM4,RWDD1,RSPH4A,ZUFSP,KPNA5,FAM162B,GPRC6A,RFX6,VGLL2,ROS1,DCBLD1,DCBLD1,GOPC,GOPC,NUS1,SLC35F1,CEP85L,CEP85L,PLN,MCM9,MCM9,ASF1A,FAM184A,FAM184A,MIR548B,MAN1A1,TBC1D32 | -1.05901 | 118.052 | 2289 | 1356.56 |
| 3 | 1 | 2.25E+08 | 225488715 | DNAH14 | -1.06248 | 122.356 | 69 | 39.2667 |
| 3 | 9 | 1.05E+08 | 106864442 | LINC00587,CYLC2,SMC2 | -1.08305 | 64.6871 | 35 | 21.5083 |

| | | | | | | | | |
|---|---|---|---|---|---|---|---|---|
| 3 | 5 | 61642892 | 61897715 | KIF2A,DIMT1,IPO11,IPO11,LRRC70,IPO11-LRRC70,IPO11,IPO11-LRRC70 | -1.08383 | 155.974 | 76 | 43.2983 |
| 3 | 12 | 64895039 | 85407733 | TBK1,RASSF3,RASSF3,MIR548C,MIR548Z,GNS,TBC1D30,WIF1,LEMD3,MSRB3,LOC100507065,RPSAP52,RPSAP52,HMGA2,HMGA2,HMGA2,LOC100129940,LLPH,TMBIM4,IRAK3,HELB,GRIP1,CAND1,DYRK2,IFNG,IL26,IL22,MDM1,RAP1B,SNORA70G,RAP1B,NUP107,SLC35E3,MDM2,CPM,CPSF6,CPSF6,MIR1279,LYZ,YEATS4,FRS2,CCT2,LRRC10,BEST3,RAB3IP,MYRFL,CNOT2,KCNMB4,PTPRB,PTPRR,TSPAN8,LGR5,ZFC3H1,THAP2,TMEM19,RAB21,TBC1D15,TPH2,TRHDE-AS1,TRHDE-AS1,TRHDE,TRHDE,ATXN7L3B,KCNC2,CAPS2,CAPS2,GLIPR1L1,GLIPR1L2,GLIPR1,GLIPR1,KRR1,KRR1,PHLDA1,NAP1L1,BBS10,OSBPL8,ZDHHC17,CSRP2,E2F7,NAV3,SYT1,SYT1,MIR1252,PAWR,PPP1R12A,OTOGL,PTPRQ,MYF6,MYF5,LIN7A,LIN7A,MIR617,LIN7A,MIR618,ACSS3,ACSS3,MIR4699,PPFIA2,PPFIA2,PPFIA2-AS1,CCDC59,CCDC59,METTL25,METTL25,TMTC2,SLC6A15 | -1.08407 | 115.947 | 1414 | 844.097 |
| 3 | 7 | 6851534 | 26192768 | CCZ1B,MIR3683,LOC100131257,C1GALT1,COL28A1,MIOS,RPA3,RPA3,UMAD1,UMAD1,GLCCI1,ICA1,NXPH1,NDUFA4,PHF14,THSD7A,TMEM106B,VWDE,SCIN,ARL4A,ETV1,DGKB,AGMO,MEOX2,ISPD,ISPD,ISPD-AS1,SOSTDC1,LRRC72,ANKMY2,BZW2,TSPAN13,AGR2,AGR3,AHR,SNX13,PRPS1L1,HDAC9,MIR1302-6,HDAC9,TWIST1,FERD3L,TWISTNB,TWISTNB,MIR3146,TMEM196,MACC1,ITGB8,ABCB5,SP8,SP4,SP4,MIR1183,DNAH11,DNAH11,CDCA7L,CDCA7L,RAPGEF5,STEAP1B,LOC541472,IL6,IL6,TOMM7,SNHG26,SNORD93,FAM126A,KLHL7,NUPL2,GPNMB,MALSU1,IGF2BP3,RPS2P32,TRA2A,CLK2P1,CCDC126,FAM221A,STK31,NPY,MPP6,GSDME,OSBPL3,CYCS,C7orf31,NPVF,MIR148A,NFE2L3 | -1.09482 | 113.76 | 1152 | 675.235 |
| 3 | X | 76814120 | 86635412 | ATRX,MAGT1,COX7B,ATP7A,ATP7A,PGAM4,PGK1,TAF9B,CYSLTR1,RTL3,LPAR4,MIR4328,P2RY10,GPR174,ITM2A,TBX22,FAM46D,BRWD3,HMGN5,SH3BGRL,POU3F4,CYLC1,RPS6KA6,MIR548I4,HDX,UBE2DNL,APOOL,APOOL,SATL1,SATL1,ZNF711,POF1B,MIR1321,CHM,CHM,MIR361,DACH2 | -1.12137 | 108.485 | 457 | 267.509 |
| 3 | 13 | 53262521 | 88484001 | CNMD,MIR759,PCDH8,OLFM4,MIR1297,MIR5007,PCDH17,DIAPH3,DIAPH3,DIAPH3-AS1,TDRD3,MIR3169,PCDH20,MIR548X2,MIR4704,PCDH9,PCDH9,PCDH9-AS2,KLHL1,KLHL1,ATXN8OS,DACH1,MZT1,BORA,DIS3,PIBF1,KLF5,KLF12,LOC100288208,TBC1D4,COMMD6,UCHL3,UCHL3,LMO7-AS1,LMO7-AS1,LMO7,LMO7,LMO7DN,KCTD12,ACOD1,CLN5,FBXL3,MYCBP2,MYCBP2,MYCBP2-AS1,SCEL,SCEL,SCEL-AS1,SLAIN1,MIR3665,SLAIN1,EDNRB-AS1,EDNRB,EDNRB,RNF219-AS1,POU4F1,RNF219-AS1,RNF219,RNF219,RBM26,NDFIP2-AS1,NDFIP2,NDFIP2,SPRY2,SLITRK1,SLITRK6,MIR4500HG,MIR4500,SLITRK5 | -1.12542 | 94.7636 | 876 | 537.107 |

| | | | | | | | | |
|---|---|---|---|---|---|---|---|---|
| 3 | 10 | 92503278 | 94653279 | HTR7,RPP30,ANKRD1,NUDT9P1,PCGF5,HECTD2-AS1,HECTD2,PPP1R3C,TNKS2,FGFBP3,BTAF1,CPEB3,MARCH5,IDE,KIF11,HHEX,EXOC6 | -1.12734 | 107.72 | 254 | 149.433 |
| 3 | 1 | 1.76E+08 | 176175142 | RFWD2 | -1.14078 | 92.8323 | 32 | 16.417 |
| 3 | 1 | 2.47E+08 | 247081765 | AHCTF1 | -1.14857 | 101.279 | 42 | 20.3621 |
| 3 | 15 | 76146675 | 77239955 | UBE2Q2,FBXO22,FBXO22,FBXO22-AS1,NRG4,TMEM266,TMEM266,LOC101929439,ETFA,ISL2,SCAPER,SCAPER,MIR3713,RCN2 | -1.15023 | 108.373 | 136 | 74.6782 |
| 3 | X | 13726774 | 15872985 | RAB9A,TRAPPC2,OFD1,GPM6B,GEMIN8,UBE2E4P,GLRA2,FANCB,MOSPD2,ASB9,ASB11,PIGA,VEGFD,PIR-FIGF,PIR-FIGF,PIR,BMX,ACE2,TMEM27,CA5BP1,CA5B,ZRSR2,AP1S2 | -1.15296 | 118.713 | 266 | 153.023 |
| 3 | 8 | 48685733 | 87497250 | PRKDC,MCM4,UBE2V2,EFCAB1,SNAI2,PPDPFL,SNTG1,PXDNL,PCMTD1,ST18,ALKAL1,RB1CC1,NPBWR1,OPRK1,ATP6V1H,RGS20,TCEA1,LYPLA1,MRPL15,SOX17,RP1,XKR4,TMEM68,TGS1,LYN,RPS20,RPS20,SNORD54,MOS,PLAG1,CHCHD7,SDR16C5,SDR16C6P,PENK,PENK,LOC101929415,IMPAD1,FAM110B,UBXN2B,CYP7A1,SDCBP,NSMAF,TOX,CA8,RAB2A,CHD7,LOC100130298,CLVS1,ASPH,MIR4470,NKAIN3,NKAIN3,UG0898H09,GGH,TTPA,YTHDF3,MIR124-2HG,MIR124-2HG,MIR124-2,LOC401463,BHLHE22,CYP7B1,LINC01299,ARMC1,MTFR1,PDE7A,DNAJC5B,TRIM55,CRH,LINC00967,RRS1,ADHFE1,C8orf46,MYBL1,VCPIP1,C8orf44,C8orf44-SGK3,C8orf44-SGK3,SGK3,PTTG3P,C8orf44-SGK3,SGK3,MCMDC2,SNHG6,SNORD87,TCF24,PPP1R42,COPS5,CSPP1,ARFGEF1,LOC102724708,CPA6,CPA6,PREX2,C8orf34-AS1,C8orf34-AS1,C8orf34,C8orf34,SULF1,SLCO5A1,PRDM14,NCOA2,TRAM1,LACTB2-AS1,LACTB2,LACTB2,XKR9,EYA1,MSC,MSC,MSC-AS1,MSC-AS1,MSC-AS1,TRPA1,TRPA1,LOC392232,KCNB2,TERF1,SBSPON,C8orf89,RPL7,RDH10,RDH10,RDH10-AS1,STAU2-AS1,STAU2-AS1,STAU2,STAU2,UBE2W,ELOC,TMEM70,LY96,JPH1,GDAP1,MIR5681A,MIR5681B,MIR2052HG,MIR2052HG,MIR2052,PI15,CRISPLD1,HNF4G,ZFHX4,PEX2,PKIA,ZC2HC1A,IL7,STMN2,HEY1,MRPS28,TPD52,MIR5708,ZBTB10,ZNF704,PAG1,FABP5,PMP2,FABP9,FABP4,FABP12,IMPA1,SLC10A5,ZFAND1,CHMP4C,SNX16,RALYL,LRRCC1,E2F5,E2F5,C8orf59,C8orf59,CA13,CA1,CA3,CA3,CA3-AS1,CA3-AS1,CA2,CA2,PSKH2,ATP6V0D2,SLC7A13,WWP1,RMDN1 | -1.15623 | 107.153 | 2175 | 1290.27 |
| 3 | 9 | 1.03E+08 | 102822552 | STX17,ERP44 | -1.15821 | 115.778 | 24 | 13.0448 |
| 3 | 12 | 59285083 | 64803971 | LRIG3,SLC16A7,FAM19A2,USP15,MIR6125,USP15,MON2,LINC01465,MIRLET7I,PPM1H,AVPR1A,DPY19L2,TMEM5,TMEM5,TMEM5-AS1,SRGAP1,C12orf66,C12orf56,XPOT | -1.16452 | 103.364 | 260 | 149.742 |
| 3 | 14 | 94706923 | 94733399 | PPP4R4 | -1.1735 | 193.87 | 15 | 9.24548 |

| | | | | | | | | |
|---|---|---|---|---|---|---|---|---|
| 3 | 6 | 1.24E+08 | 128322478 | TRDN,TRDN-AS1,TRDN,NKAIN2,RNF217-AS1,RNF217-AS1,RNF217,RNF217,TPD52L1,HDDC2,HEY2,NCOA7,NCOA7-AS1,NCOA7,HINT3,TRMT11,CENPW,RSPO3,RNF146,ECHDC1,KIAA0408,SOGA3,SOGA3,C6orf58,THEMIS,PTPRK | -1.18175 | 117.773 | 271 | 161.538 |
| 3 | 10 | 35302527 | 35426839 | CUL2,CUL2,MIR3611,CREM | -1.18683 | 138.796 | 21 | 13.2986 |
| 3 | 12 | 85450461 | 88373515 | LRRIQ1,ALX1,RASSF9,NTS,MGAT4C,LINC02258,MKRN9P | -1.19021 | 94.897 | 79 | 47.4171 |
| 3 | 1 | 1.73E+08 | 173569368 | SLC9C2 | -1.1931 | 145.884 | 20 | 10.9471 |
| 3 | 22 | 45758675 | 45804789 | SMC1B | -1.19703 | 154.839 | 16 | 10.0268 |
| 3 | 21 | 38459651 | 38564608 | TTC3,TTC3,TTC3-AS1 | -1.19923 | 151.787 | 63 | 32.3746 |
| 3 | 6 | 82462993 | 86215109 | IBTK,TPBG,UBE3D,DOPEY1,DOPEY1,PGM3,PGM3,RWDD2A,ME1,PRSS35,SNAP91,RIPPLY2,CYB5R4,MRAP2,CEP162,TBX18,NT5E | -1.19935 | 109.573 | 293 | 174.669 |
| 3 | 18 | 66382152 | 72021873 | TMX3,CCDC102B,DOK6,CD226,RTTN,SOCS6,CBLN2,NETO1,NETO1,MIR548AV,FBXO15,TIMM21,CYB5A,C18orf63 | -1.20814 | 95.8206 | 200 | 119.819 |
| 3 | 12 | 43750904 | 48104695 | ADAMTS20,PUS7L,IRAK4,TWF1,TMEM117,NELL2,DBX2,PLEKHA8P1,ANO6,LINC00938,ARID2,SCAF11,SLC38A1,SLC38A2,SLC38A4,AMIGO2,PCED1B,MIR4698,PCED1B,MIR4494,RPAP3,ENDOU | -1.20967 | 115.287 | 350 | 209.062 |
| 3 | 4 | 2194826 | 2240810 | POLN,POLN,HAUS3 | -1.21255 | 115.863 | 12 | 7.62104 |
| 3 | 18 | 18520842 | 18690189 | ROCK1 | -1.21312 | 156.091 | 37 | 21.7852 |
| 3 | 4 | 68547794 | 70800523 | UBA6,UBA6-AS1,GNRHR,TMPRSS11D,TMPRSS11A,TMPRSS11F,LOC550113,TMPRSS11F,LOC550113,SYT14P1,TMPRSS11F,FTLP10,TMPRSS11BNL,TMPRSS11BNL,TMPRSS11B,YTHDC1,TMPRSS11E,UGT2B17,UGT2B15,UGT2B10,UGT2A3,UGT2B7,LOC105377267,UGT2B11,UGT2B28,UGT2B4,UGT2A2,UGT2A1,UGT2A1,SULT1B1,SULT1E1,CSN1S1 | -1.21844 | 129.179 | 227 | 119.299 |
| 3 | 6 | 76331162 | 76633501 | SENP6,MYO6,IMPG1 | -1.22779 | 126.89 | 63 | 38.7197 |
| 3 | 8 | 38975487 | 39694801 | ADAM32,ADAM3A,ADAM18,ADAM2 | -1.23721 | 114.765 | 96 | 50.5654 |
| 3 | 2 | 37315391 | 37449657 | GPATCH11,EIF2AK2,SULT6B1,CEBPZOS,CEBPZOS,CEBPZ,CEBPZ | -1.23896 | 147.051 | 50 | 28.0424 |
| 3 | 2 | 96521007 | 96648124 | ANKRD36C | -1.2444 | 249.628 | 24 | 10.4524 |
| 3 | 12 | 4611535 | 4662392 | C12orf4,RAD51AP1 | -1.24742 | 130.498 | 21 | 12.0889 |
| 3 | 2 | 21267235 | 24549626 | TDRD15,KLHL29,ATAD2B,UBXN2A,MFSD2B,WDCP,FKBP1B,SF3B6,FAM228B,TP53I3,FAM228B,PFN4,FAM228B,FAM228A,ITSN2 | -1.2545 | 102.15 | 225 | 139.425 |
| 3 | X | 1.54E+08 | 154467146 | FUNDC2,CMC4,CMC4,MTCP1,BRCC3,VBP1 | -1.26639 | 100.912 | 30 | 16.5155 |
| 3 | 12 | 1.12E+08 | 111993775 | ATXN2 | -1.30479 | 124.161 | 14 | 8.33592 |
| 3 | 1 | 91407353 | 91866661 | ZNF644,HFM1 | -1.31913 | 90.3009 | 41 | 24.0223 |
| 3 | 18 | 10500 | 2784834 | ROCK1P1,USP14,THOC1,COLEC12,CETN1,CLUL1,CLUL1,TYMSOS,TYMSOS,TYMS,TYMS,TYMS,ENOSF1,ENOSF1,YES1,ADCYAP1,LINC00470,METTL4,NDC80,SMCHD1 | -1.3464 | 111.913 | 217 | 127.588 |
| 3 | 2 | 2.02E+08 | 202469425 | STRADB,C2CD6 | -1.36814 | 113.07 | 47 | 27.2304 |
| 3 | 3 | 1.54E+08 | 154033126 | DHX36 | -1.37422 | 181.104 | 14 | 8.69173 |
| 3 | 5 | 54562927 | 54675052 | DHX29,MTREX | -1.37738 | 132.266 | 49 | 27.2037 |
| 3 | 13 | 32899142 | 32929465 | BRCA2 | -1.38181 | 110.977 | 33 | 22.7954 |
| 3 | 10 | 27294428 | 27431433 | ANKRD26,YME1L1 | -1.39192 | 127.127 | 59 | 33.0352 |
| 3 | 16 | 58752100 | 64981019 | GOT2,LOC729159,CDH8 | -1.40867 | 61.9765 | 78 | 48.9243 |
| 3 | 5 | 68487522 | 68706460 | CENPH,MRPS36,CDK7,CCDC125,AK6,AK6,TAF9,AK6,TAF9,RAD17,RAD17 | -1.4113 | 122.892 | 71 | 38.6545 |
| 3 | 3 | 1.22E+08 | 121544959 | IQCB1 | -1.42854 | 76.1133 | 10 | 4.4314 |

| | | | | | | | | |
|---|---|---|---|---|---|---|---|---|
| 3 | 14 | 35036807 | 49431910 | SNX6,CFL2,BAZ1A,SRP54,FAM177A1,FAM177A1,LOC101927178,LOC101927178,PPP2R3C,PPP2R3C,KIAA0391,PSMA6,NFKBIA,INSM2,RALGAPA1,RALGAPA1P1,RALGAPA1,RALGAPA1P1,SNORA101B,BRMS1L,MBIP,SFTA3,SFTA3,NKX2-1,SFTA3,NKX2-1,NKX2-1-AS1,NKX2-1,NKX2-1-AS1,NKX2-8,PAX9,SLC25A21,SLC25A21,SLC25A21-AS1,MIPOL1,FOXA1,TTC6,SSTR1,CLEC14A,SEC23A,GEMIN2,TRAPPC6B,PNN,MIA2,FBXO33,LRFN5,FSCB,C14orf28,LOC101927418,KLHL28,TOGARAM1,PRPF39,PRPF39,SNORD127,FKBP3,FANCM,MIS18BP1,RPL10L,MDGA2,MIR548Y | -1.44692 | 92.5562 | 727 | 429.677 |
| 3 | 15 | 56665563 | 57017227 | TEX9,TEX9,MNS1,MNS1,ZNF280D | -1.45721 | 113.011 | 52 | 30.8569 |
| 3 | 20 | 55906878 | 55918510 | SPO11 | -1.47142 | 98.324 | 13 | 7.44615 |
| 3 | 2 | 55536143 | 55914912 | CCDC88A,CFAP36,PPP4R3B,PNPT1 | -1.47544 | 111.65 | 96 | 55.8537 |
| 3 | 16 | 46457168 | 46594033 | ANKRD26P1 | -1.4937 | 109.882 | 23 | 12.4725 |
| 3 | 2 | 71582792 | 71645853 | ZNF638 | -1.50207 | 131.696 | 26 | 15.2869 |
| 3 | 2 | 27900113 | 27917484 | SLC4A1AP | -1.50344 | 105.767 | 13 | 6.68611 |
| 3 | 2 | 75879224 | 75923533 | MRPL19,GCFC2 | -1.51475 | 106.083 | 19 | 11.2112 |
| 3 | 15 | 62238501 | 62351931 | VPS13C | -1.51484 | 113.937 | 44 | 26.3488 |
| 3 | 14 | 25347855 | 31209795 | STXBP6,NOVA1,MIR4307HG,MIR4307HG,MIR4307,FOXG1,LINC01551,PRKD1,G2E3-AS1,G2E3,G2E3,SCFD1 | -1.51866 | 79.725 | 151 | 88.9514 |
| 3 | 16 | 47252742 | 47493168 | ITFG1 | -1.53197 | 80.8494 | 20 | 10.5885 |
| 3 | 12 | 20968671 | 21621704 | SLCO1B3,SLCO1B7,SLCO1B1,SLCO1A2,SLCO1A2,IAPP,PYROXD1 | -1.5412 | 86.0006 | 102 | 52.7005 |
| 3 | 4 | 56319173 | 57361620 | CLOCK,PDCL2,NMU,EXOC1L,EXOC1,CEP135,KIAA1211,AASDH,PPAT,PAICS,SRP72 | -1.54208 | 101.9 | 169 | 99.1189 |
| 3 | 9 | 1.14E+08 | 114553803 | C9orf84 | -1.55271 | 117.587 | 29 | 17.4509 |
| 3 | 7 | 35871055 | 36012243 | 7-Sep | -1.55305 | 97.6674 | 20 | 9.77004 |
| 3 | 3 | 1.08E+08 | 108361485 | CIP2A,DZIP3 | -1.55468 | 116.876 | 34 | 20.8775 |
| 3 | 2 | 39431610 | 39605278 | CDKL4,MAP4K3 | -1.56876 | 88.0481 | 39 | 22.0718 |
| 3 | 8 | 1.42E+08 | 141900129 | PTK2 | -1.57509 | 80.9677 | 30 | 15.1905 |
| 3 | 8 | 1.04E+08 | 104455023 | DCAF13 | -1.5786 | 125.904 | 13 | 7.18364 |
| 3 | 14 | 53110194 | 53251443 | ERO1A,PSMC6,STYX,GNPNAT1 | -1.57957 | 87.1168 | 50 | 26.8772 |
| 3 | 2 | 15601808 | 17963224 | NBAS,DDX1,LINC01804,MYCNOS,MYCN,MYCN,FAM49A,RAD51AP2,VSNL1,SMC6,GEN1 | -1.58059 | 86.6568 | 151 | 92.596 |
| 3 | 18 | 35388792 | 42280805 | MIR924HG,MIR924,MIR924HG,MIR5583-2,MIR5583-1,PIK3C3,RIT2,SYT4 | -1.58613 | 57.3488 | 91 | 57.6568 |
| 3 | 10 | 71917440 | 71977779 | SAR1A,PPA1 | -1.58684 | 91.5866 | 16 | 8.07838 |
| 3 | 10 | 70892605 | 70947513 | VPS26A,SUPV3L1 | -1.59172 | 118.775 | 20 | 11.1045 |
| 3 | 9 | 79814790 | 80022620 | VPS13A | -1.59256 | 98.1757 | 75 | 43.3229 |
| 3 | 8 | 15556300 | 17143853 | TUSC3,MSR1,FGF20,MICU3,ZDHHC2,CNOT7,VPS37A | -1.60206 | 85.4622 | 96 | 53.7476 |
| 3 | 1 | 1.04E+08 | 107598836 | COL11A1,RNPC3,AMY2B,AMY2A | -1.60248 | 55.7557 | 56 | 29.8711 |
| 3 | 9 | 72920176 | 72965395 | SMC5 | -1.6216 | 95.2808 | 15 | 8.11198 |
| 3 | 1 | 86814439 | 86891222 | ODF2L,CLCA2 | -1.69324 | 85.1777 | 21 | 11.7231 |
| 3 | 15 | 99800655 | 100243011 | LRRC28,MEF2A | -1.70803 | 74.1934 | 29 | 15.6014 |
| 3 | 15 | 43269996 | 43348633 | UBR1 | -1.70941 | 103.064 | 28 | 17.4779 |
| 3 | 2 | 13043698 | 14621363 | - | -1.76359 | 5.99107 | 13 | 8.00505 |
| 3 | 5 | 72147148 | 72354387 | TNPO1,TNPO1,MIR4804,FCHO2 | -1.78022 | 89.2333 | 51 | 25.9735 |
| 3 | 10 | 1.17E+08 | 117486201 | TRUB1,ATRNL1 | -1.78104 | 66.9397 | 53 | 28.9345 |
| 3 | 2 | 2.14E+08 | 214239890 | SPAG16 | -1.80078 | 69.8797 | 14 | 7.65888 |
| 3 | X | 1.23E+08 | 123217423 | THOC2,XIAP,STAG2 | -1.8107 | 76.0473 | 57 | 33.8887 |
| 3 | 15 | 60724015 | 60768436 | ICE2 | -1.82373 | 108.106 | 22 | 11.6809 |
| 3 | 10 | 96087720 | 96361470 | PLCE1,NOC3L,TBC1D12,HELLS | -1.82822 | 89.07 | 65 | 38.2056 |
| 3 | 9 | 33536823 | 33567306 | ANKRD18B | -1.83728 | 184.85 | 11 | 6.09821 |
| 3 | 9 | 19058941 | 19096854 | HAUS6,HAUS6,SCARNA8 | -1.84498 | 70.9321 | 18 | 8.47488 |
| 3 | 18 | 9104885 | 9134187 | NDUFV2,NDUFV2,NDUFV2-AS1 | -1.8514 | 84.6242 | 10 | 5.02986 |

| | | | | | | | | |
|---|---|---|---|---|---|---|---|---|
| 3 | 20 | 469392 | 485964 | CSNK2A1 | -1.85213 | 105.79 | 11 | 5.87349 |
| 3 | 19 | 34904625 | 34960222 | PDCD2L,UBA2 | -1.85422 | 81.3919 | 26 | 14.0314 |
| 3 | X | 72783963 | 73045726 | CHIC1,TSIX,XIST | -1.86998 | 74.0171 | 29 | 18.918 |
| 3 | 2 | 26455040 | 26507870 | HADHA,HADHB | -1.87437 | 76.5118 | 22 | 12.7188 |
| 3 | 10 | 70666591 | 70703093 | DDX50 | -1.89622 | 69.8347 | 15 | 7.89515 |
| 3 | 9 | 26984295 | 27061103 | IFT74,IFT74,LRRC19 | -1.90553 | 84.7726 | 21 | 13.2702 |
| 3 | 11 | 1.19E+08 | 118651963 | DDX6 | -1.90706 | 58.1136 | 18 | 9.36038 |
| 3 | X | 8759294 | 9000268 | FAM9A,FAM9B | -1.90754 | 121.186 | 16 | 8.09925 |
| 3 | 4 | 1.14E+08 | 113825743 | ZGRF1,LARP7,LARP7,LOC109864269,LARP7,LOC109864269,MIR367,MIR302D,LARP7,LOC109864269,MIR302A,MIR302C,LARP7,LOC109864269,MIR302C,MIR302B,ANK2 | -1.91253 | 67.2863 | 39 | 22.38 |
| 3 | 20 | 5538713 | 5573992 | GPCPD1 | -1.92227 | 76.4728 | 22 | 12.2012 |
| 3 | 2 | 11312739 | 11597301 | PQLC3,ROCK2,E2F6 | -1.92767 | 90.0654 | 48 | 27.8784 |
| 3 | 4 | 1.29E+08 | 129043425 | PLK4,MFSD8,ABHD18,LARP1B | -1.93227 | 71.6976 | 55 | 32.2714 |
| 3 | X | 1.47E+08 | 147022222 | FMR1 | -1.93741 | 93.5621 | 14 | 7.97539 |
| 3 | 3 | 5212065 | 5216226 | ARL8B | -1.95312 | 69.6573 | 10 | 5.3846 |
| 3 | 16 | 81012218 | 81060236 | CMC2,CENPN | -1.95605 | 69.9168 | 18 | 10.1358 |
| 3 | 10 | 37425469 | 37508143 | ANKRD30A | -1.97416 | 96.7025 | 18 | 8.62165 |
| 3 | X | 30683685 | 30864663 | GK,TAB3 | -1.98056 | 76.3971 | 34 | 19.1616 |
| 3 | X | 1.18E+08 | 117810768 | DOCK11 | -2.02107 | 65.8029 | 62 | 31.9998 |
| 3 | 18 | 76873231 | 76953331 | ATP9B | -2.02417 | 57.8904 | 14 | 6.92321 |
| 3 | 1 | 25599065 | 25697101 | RSRP1,RHD,TMEM50A,TMEM50A,RHCE,RHCE | -2.03811 | 77.9732 | 24 | 11.0927 |
| 3 | 20 | 1212073 | 1229463 | RAD21L1 | -2.04952 | 71.8418 | 10 | 6.24576 |
| 3 | 1 | 1.15E+08 | 115537456 | SYCP1 | -2.05981 | 41.7859 | 28 | 14.507 |
| 3 | 10 | 32742230 | 33197337 | CCDC7,ITGB1 | -2.06169 | 44.609 | 57 | 29.5453 |
| 3 | 4 | 1.86E+08 | 185652200 | CASP3,PRIMPOL,PRIMPOL,CENPU,CENPU | -2.07349 | 73.1891 | 40 | 21.7169 |
| 3 | 9 | 15468756 | 15506699 | PSIP1 | -2.08459 | 55.073 | 18 | 9.37817 |
| 3 | 11 | 1.12E+08 | 112042669 | IL18,TEX12 | -2.08785 | 54.3801 | 10 | 5.43653 |
| 3 | 1 | 1.51E+08 | 150692062 | HORMAD1 | -2.10119 | 63.1795 | 17 | 8.68101 |
| 3 | 11 | 9307934 | 9466674 | TMEM41B,IPO7,IPO7,SNORA23 | -2.1033 | 60.9268 | 39 | 20.6153 |
| 3 | 18 | 14763968 | 14850398 | ANKRD30B,ANKRD30B,MIR3156-2 | -2.10371 | 96.587 | 22 | 10.6104 |
| 3 | 9 | 86354552 | 86403657 | GKAP1 | -2.11066 | 41.1762 | 11 | 6.27251 |
| 3 | 4 | 1.46E+08 | 146095810 | ANAPC10,ABCE1,OTUD4 | -2.12195 | 77.7346 | 62 | 30.702 |
| 3 | 12 | 88374015 | 88586620 | C12orf50,C12orf29,C12orf29,CEP290,CEP290,TMTC3 | -2.12535 | 82.398 | 92 | 53.8317 |
| 3 | 19 | 34699917 | 34718241 | LSM14A | -2.1376 | 67.3451 | 10 | 5.24679 |
| 3 | 12 | 64808565 | 64891882 | XPOT,TBK1 | -2.14125 | 73.5919 | 49 | 28.1788 |
| 3 | X | 96167312 | 96330337 | DIAPH2 | -2.14936 | 42.3219 | 16 | 9.40169 |
| 3 | 14 | 56105877 | 56128384 | KTN1 | -2.15023 | 63.0689 | 22 | 13.8752 |
| 3 | 7 | 33054413 | 33066612 | NT5C3A | -2.15057 | 72.0562 | 10 | 5.65356 |
| 3 | 3 | 1.12E+08 | 111936390 | SLC9C1 | -2.18624 | 45.1841 | 15 | 8.76216 |
| 3 | 4 | 1.41E+08 | 141471498 | SCOC,CLGN,MGAT4D,ELMOD2 | -2.20003 | 68.7045 | 41 | 22.7205 |
| 3 | 18 | 9239420 | 9256628 | ANKRD12 | -2.20568 | 39.5105 | 11 | 6.80585 |
| 3 | 15 | 44615047 | 44816344 | CASC4,CTDSPL2 | -2.21692 | 51.0092 | 31 | 15.5452 |
| 3 | 17 | 11924855 | 12013819 | MAP2K4,MAP2K4,MIR744 | -2.22695 | 53.859 | 16 | 7.8976 |
| 3 | 10 | 1.32E+08 | 131977594 | GLRX3 | -2.24198 | 44.1978 | 14 | 6.67483 |
| 3 | 20 | 58425361 | 58497518 | SYCP2 | -2.25309 | 63.27 | 41 | 24.2602 |
| 3 | 20 | 13699450 | 13755981 | ESF1 | -2.2875 | 39.0826 | 15 | 7.50633 |
| 3 | 10 | 91468950 | 92502413 | KIF20B,HTR7 | -2.31018 | 45.7021 | 40 | 25.0646 |
| 3 | 16 | 66792669 | 66861580 | TERB1,NAE1 | -2.31525 | 69.6065 | 33 | 19.7776 |
| 3 | 12 | 60500 | 569751 | IQSEC3,IQSEC3,LOC574538,SLC6A12,SLC6A12,LOC101929384,SLC6A13,KDM5A,CCDC77,B4GALNT3 | -2.32952 | 42.7208 | 113 | 68.6007 |
| 3 | 4 | 1.86E+08 | 186384217 | C4orf47,C4orf47,CCDC110,CCDC110 | -2.34106 | 58.2881 | 18 | 11.9963 |
| 3 | 4 | 68338360 | 68547398 | CENPC,STAP1,UBA6 | -2.35313 | 60.7039 | 74 | 40.771 |
| 3 | 18 | 65182367 | 66381272 | TMX3 | -2.3674 | 35.3268 | 22 | 13.7405 |
| 3 | 7 | 34982191 | 35058293 | DPY19L1 | -2.37388 | 45.0689 | 19 | 9.23999 |
| 3 | X | 31222050 | 31696975 | DMD | -2.39135 | 34.8157 | 26 | 14.7547 |

| | | | | | | | | |
|---|---|---|---|---|---|---|---|---|
| | | | | CSN1S1,CSN2,STATH,HTN3,HTN1,CSN1S2AP,CSN1S2B | | | | |
| 3 | 4 | 70801612 | 71067325 | P,PRR27,ODAM | -2.41412 | 56.743 | 60 | 33.5929 |
| 3 | 18 | 30518568 | 31158039 | CCDC178 | -2.44675 | 36.7386 | 38 | 19.654 |
| 3 | 4 | 1.04E+08 | 104118088 | CENPE | -2.46161 | 60.6141 | 45 | 27.9329 |
| 3 | 6 | 1.21E+08 | 121625954 | TBC1D32 | -2.47595 | 44.7197 | 31 | 16.8653 |
| 3 | 10 | 1.15E+08 | 115469278 | HABP2,HABP2,NRAP,NRAP,CASP7 | -2.49582 | 42.2755 | 68 | 42.2537 |
| 3 | 22 | 38945892 | 38958409 | DMC1 | -2.53508 | 27.8408 | 4 | 2.03817 |
| 3 | 11 | 1.1E+08 | 110447979 | RDX,FDX1,ARHGAP20 | -2.53692 | 41.0014 | 27 | 13.288 |
| 3 | 15 | 79170435 | 79189324 | MORF4L1 | -2.5431 | 77.4943 | 14 | 7.86229 |
| 3 | X | 1.14E+08 | 114422944 | LRCH2 | -2.55095 | 42.262 | 12 | 7.02982 |
| 3 | 13 | 53036582 | 53262021 | CKAP2,SUGT1 | -2.6023 | 59.1199 | 23 | 12.7096 |
| 3 | 14 | 60903574 | 60951791 | C14orf39 | -2.60291 | 35.3787 | 16 | 8.44049 |
| 3 | 6 | 1.23E+08 | 122752699 | HSF2 | -2.60794 | 52.361 | 13 | 6.62372 |
| 3 | 13 | 96508866 | 96684255 | UGGT2 | -2.62423 | 39.9118 | 41 | 23.1741 |
| 3 | 6 | 86215609 | 86284141 | SNX14 | -2.65047 | 53.2532 | 26 | 15.0263 |
| 3 | 16 | 10525192 | 10567925 | ATF7IP2 | -2.68739 | 39.0981 | 14 | 7.34803 |
| 3 | 13 | 21950728 | 22096833 | MIPEPP3,ZDHHC20,ZDHHC20,MICU2 | -2.69058 | 50.804 | 29 | 15.5464 |
| 3 | 15 | 49801038 | 49935816 | FAM227B,DTWD1 | -2.69878 | 37.963 | 24 | 12.6256 |
| 3 | 10 | 94654481 | 94715540 | EXOC6 | -2.70396 | 48.8971 | 13 | 8.01749 |
| 3 | 12 | 1.01E+08 | 100729623 | SCYL2 | -2.74583 | 43.5175 | 20 | 11.155 |
| 3 | 2 | 1.98E+08 | 197762011 | PGAP1 | -2.75664 | 38.2816 | 10 | 6.06867 |
| 3 | 6 | 1.24E+08 | 123760270 | TRDN | -2.81909 | 33.3638 | 34 | 18.127 |
| 3 | 12 | 50822684 | 50848230 | LARP4 | -2.82685 | 32.1858 | 16 | 8.59941 |
| 3 | 12 | 85408233 | 85450461 | TSPAN19,LRRIQ1 | -2.90628 | 50.596 | 17 | 10.6555 |
| 3 | 13 | 48881413 | 48955589 | RB1 | -2.94443 | 35.2689 | 17 | 9.35482 |
| 3 | 7 | 87516041 | 87533724 | DBF4 | -3.00182 | 35.5337 | 13 | 6.26016 |
| 3 | 10 | 95422275 | 95451861 | PDE6C,FRA10AC1 | -3.02741 | 43.3521 | 16 | 8.46324 |
| 3 | 10 | 1.18E+08 | 118187635 | CCDC172,PNLIPRP3 | -3.28571 | 12.4797 | 10 | 5.4631 |
| 3 | 4 | 84379417 | 84403434 | MRPS18C,MRPS18C,ABRAXAS1,ABRAXAS1 | -3.42062 | 29.1691 | 18 | 9.56343 |
| 3 | 10 | 1.24E+08 | 124351472 | DMBT1 | -3.6072 | 23.9528 | 10 | 5.41279 |
| 3 | Y | 2656174 | 10058894 | TGIF2LY,PCDH11Y,TBL1Y,PRKY | -4.22921 | 14.4614 | 90 | 49.0956 |
| | | | | GYG2P1,UTY,VCY1B,VCY,VCY,VCY1B,NLGN4Y,TTTY14, | | | | |
| 3 | Y | 13105053 | 59106039 | CD24 | -5.68429 | 3.49659 | 151 | 86.6763 |
