# Supplementary material for: Multiomics analysis of serial PARP inhibitor treated metastatic TNBC inform on rational combination therapies
Source: NPJ Precis Oncol. 2021 Oct 19;5:92. doi: 10.1038/s41698-021-00232-w (PMC8526613; doi:10.1038/s41698-021-00232-w)
Supplement: Supplementary file 2 — Reporting Summary [file 41698_2021_232_MOESM2_ESM.pdf]

nature research

Corresponding author(s): NPJPRECISIONONCOLOGY-00821R

Last updated by author(s): Jan 22, 2021

Reporting Summary

Nature Research wishes to improve the reproducibility of the work that we publish. This form provides structure for consistency and transparency in reporting. For further information on Nature Research policies, see our Editorial Policies and the Editorial Policy Checklist.

Statistics

For all statistical analyses, confirm that the following items are present in the figure legend, table legend, main text, or Methods section.

n/a	Confirmed	
☒	☐	The exact sample size (*n*) for each experimental group/condition, given as a discrete number and unit of measurement
☒	☐	A statement on whether measurements were taken from distinct samples or whether the same sample was measured repeatedly
☒	☐	The statistical test(s) used AND whether they are one- or two-sided<br>*Only common tests should be described solely by name; describe more complex techniques in the Methods section.*
☒	☐	A description of all covariates tested
☒	☐	A description of any assumptions or corrections, such as tests of normality and adjustment for multiple comparisons
☒	☐	A full description of the statistical parameters including central tendency (e.g. means) or other basic estimates (e.g. regression coefficient) AND variation (e.g. standard deviation) or associated estimates of uncertainty (e.g. confidence intervals)
☒	☐	For null hypothesis testing, the test statistic (e.g. *F*, *t*, *r*) with confidence intervals, effect sizes, degrees of freedom and *P* value noted<br>*Give P values as exact values whenever suitable.*
☒	☐	For Bayesian analysis, information on the choice of priors and Markov chain Monte Carlo settings
☒	☐	For hierarchical and complex designs, identification of the appropriate level for tests and full reporting of outcomes
☒	☐	Estimates of effect sizes (e.g. Cohen's *d*, Pearson's *r*), indicating how they were calculated

Our web collection on statistics for biologists contains articles on many of the points above.

Software and code

Policy information about availability of computer code

Data collection	No software was used
Data analysis	Somatic variants were called following the GATK Best Practices Somatic Short Variant Discovery, including GATK4 MuTect2 (2.1-beta, Broad Institute).<br>All variants were hand-curated with Samtools mpileup (1.2, https://github.com/samtools/samtools) and Interactive Genome Viewer (2.3.82, Broad Institute) to assess their validity in context.<br>The Cyc-IF data analysis was performed on python and the codes are available on Github (https://github.com/biodev/cycIF-workflow/tree/v1.0).

For manuscripts utilizing custom algorithms or software that are central to the research but not yet described in published literature, software must be made available to editors and reviewers. We strongly encourage code deposition in a community repository (e.g. GitHub). See the Nature Research guidelines for submitting code & software for further information.

Data

Policy information about availability of data

All manuscripts must include a data availability statement. This statement should provide the following information, where applicable:

- Accession codes, unique identifiers, or web links for publicly available datasets
- A list of figures that have associated raw data
- A description of any restrictions on data availability

The raw DNA sequencing data generated during the current study, is available in the dbGaP repository: (not available yet). As these files are controlled access, researchers must request access to the dbGaP data. The repository also includes clinical and phenotypic metadata and molecular data (including protein expression). The reverse phase protein array data (protein expression data), is publicly available in the Synapse repository under the following project accession:

(not available yet). HER2 immunohistochemistry data, Intracellular Signaling Protein Panel assay data, and data from the GeneTrails Solid Tumor Panel assay, are not publicly available, but will be made available on reasonable request. Please contact the Knight Diagnostic Laboratories at Oregon Health and Science University (OHSU), email: KDLClientServices@ohsu.edu, for more information on these datasets. The data generated and analyzed during this study are described in the following metadata record: (not available yet)."
dbGaP (not available yet) (2021).

Field-specific reporting

Please select the one below that is the best fit for your research. If you are not sure, read the appropriate sections before making your selection.

☒ Life sciences ☐ Behavioural & social sciences ☐ Ecological, evolutionary & environmental sciences

For a reference copy of the document with all sections, see nature.com/documents/nr-reporting-summary-flat.pdf

Life sciences study design

All studies must disclose on these points even when the disclosure is negative.

Sample size	This study was done on 3 human subjects. The small patient number is due to the fact that this was a pilot study to test the feasibility of our clinical trial design.
Data exclusions	No data was excluded.
Replication	This is not applicable to this study.
Randomization	Randomization was not relevant to this study since the outcome was the feasibility and not the biological findings.
Blinding	Blinding was not relevant to this study since the outcome was the feasibility and not the biological findings.

Reporting for specific materials, systems and methods

We require information from authors about some types of materials, experimental systems and methods used in many studies. Here, indicate whether each material, system or method listed is relevant to your study. If you are not sure if a list item applies to your research, read the appropriate section before selecting a response.

Materials & experimental systems

n/a	Involved in the study	
☐	☒	Antibodies
☒	☐	Eukaryotic cell lines
☒	☐	Palaeontology and archaeology
☒	☐	Animals and other organisms
☐	☒	Human research participants
☐	☒	Clinical data
☒	☐	Dual use research of concern

Methods

n/a	Involved in the study	
☒	☐	ChIP-seq
☒	☐	Flow cytometry
☒	☐	MRI-based neuroimaging

Antibodies

Antibodies used	All antibodies used are described in supplementary tables 1 and 3, which includes the manufacturer and catalogue number.
Validation	All antibodies are commercially available and validation is decribed on the manufacturers products' webpage.

Human research participants

Policy information about studies involving human research participants

Population characteristics	Eligibility criteria included biopsy proven metastatic triple negative breast cancer, ECOG PS ≤2, received ≤2 chemotherapeutic agents in the metastatic setting. Patients with prior PARP inhibtior or immune checkpoint blockade exposure in the metastatic setting were excluded.
Recruitment	Patients for this study were enrolled at Oregon Health and Science University Center for Health and Healing and Community Hematology and Oncology clinics
Ethics oversight	The study was reviewed and approved by the Oregon Health and Science University Institutional Review Board. All

Ethics oversight	participants signed written informed consent prior to any study related procedures (IRB#18239)

Note that full information on the approval of the study protocol must also be provided in the manuscript.

Clinical data

Policy information about clinical studies

All manuscripts should comply with the ICMJE guidelines for publication of clinical research and a completed CONSORT checklist must be included with all submissions.

Clinical trial registration	NCT03544125
Study protocol	Attached
Data collection	Study was IRB approved 4/6/2018, and completed 11/18/2020 after all patients had completed all study procedures including follow up. Data was collected using paper case report forms.
Outcomes	The primary endpoint of the study is feasibility of completing all CLIA assays within 28 days of pre-treatment biopsy
